# Visible‐Light‐Mediated Energy Transfer Enables the Synthesis of β‐Lactams via Intramolecular Hydrogen Atom Transfer

**DOI:** 10.1002/anie.202213086

**Published:** 2022-10-26

**Authors:** Meghan J. Oddy, Daniel A. Kusza, Ryan G. Epton, Jason M. Lynam, William P. Unsworth, Wade F. Petersen

**Affiliations:** ^1^ Department of Chemistry University of Cape Town Rondebosch, Cape Town 7700 South Africa; ^2^ Department of Chemistry University of York York YO10 5DD UK

**Keywords:** C−H Functionalization, Energy Transfer, Heterocycles, Hydrogen Atom Transfer, Photocatalysis

## Abstract

The synthesis of 2‐azetidinones (β‐lactams) from simple acrylamide starting materials by visible‐light‐mediated energy transfer catalysis is reported. The reaction features a C(sp^3^)−H functionalization via a variation of the Norrish–Yang photocyclization involving a carbon‐to‐carbon 1,5‐hydrogen atom transfer (supported by deuterium labelling and DFT calculations) and can be used for the construction of a diverse range of β‐lactam products.

## Graphical abstract

A visible‐light‐mediated, 2‐chlorothioxanthone (**2‐CTX**) triplet energy sensitization of simple acrylamides enables facile access to a diverse range of β‐lactam products. The reaction proceeds through triplet sensitization of the C−C olefin moiety and features a C(sp^3^)−H functionalization through a biradical carbon‐to‐carbon 1,5‐hydrogen atom transfer. The proposed mechanism is supported by deuterium labelling experiments and DFT calculations.

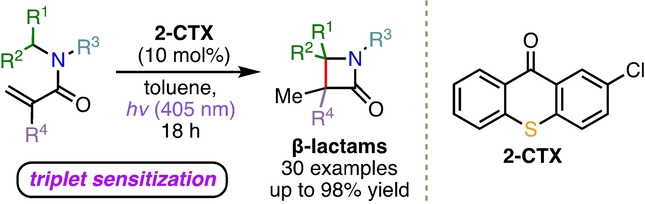

β‐Lactams are amongst the most important compound classes in medicinal and biological chemistry—most famously in frontline penicillin antibiotics, but also in a range of other compounds of wide interest in medicinal chemistry and chemical biology (Figure 1A).^[1]^ Reduction of the lactam carbonyl group also enables their conversion into azetidines, another privileged scaffold with broad utility in medicinal chemistry (Figure 1B).[^2^, ^3^] Effective strategies for the synthesis of β‐lactams are therefore highly desirable, and various methods have been developed over the years.^[4]^ However, most require the use of relatively reactive precursors (e.g. ketenes and nitrones),^[5]^ which can limit their utility in some applications. In this study, we wanted to investigate whether β‐lactams could be accessed from simpler, more benign precursors, by harnessing visible‐light‐mediated energy transfer to enable novel C−H bond functionalization reactions.[^6^, ^7^] Direct C−H bond functionalization reactions are amongst the most powerful methods in modern synthetic chemistry, especially when they enable functionalization of unactivated C(sp^3^)−H bonds.[^8^, ^9^, ^10^] Such reactions are usually based on transition metal‐catalyzed activation^[9]^ and/or the use of directing groups.^[10]^ However, in the spirit of developing more sustainable synthetic methods, complementary metal‐free, low‐energy‐consuming, and atom‐economical strategies for C−H functionalization are of high importance.

**Figure 1:**
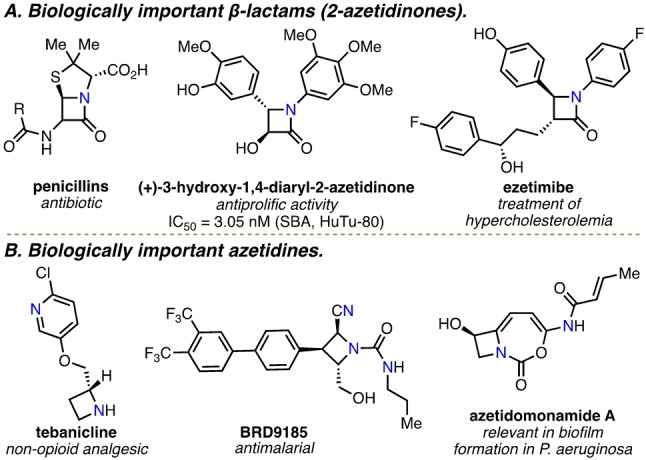
Representative set of biologically important β‐lactam (2‐azetidinones) and azetidine derivatives.

Hydrogen atom transfer (HAT) processes are important in this regard,[^11^, ^12^] and within this reaction class, Norrish–Yang‐type photocyclization reactions were considered well‐suited to the goals of this project. Norrish–Yang reactions can be performed under relatively mild conditions and without the use of metal catalysts. C(sp^3^)−H bond functionalization is enabled through the 1,5‐HAT of a diradical triplet excited state intermediate, itself generated under irradiation, typically with UV light (Scheme 1A).[^13^, ^14^] Early reports of the application of this chemistry in β‐lactam synthesis can be found in the works of Aoyama^[14a]^ (1978) and Wehrli (1980, Scheme 1B, left).^[14c]^ And in a related process, Hasegawa reported a β‐lactam synthesis via a rare C‐to‐C 1,5‐HAT variation (1977, Scheme 1B, right).^[14d]^ However, all of these methods require the use of high power UV light sources, which tends to limit the reaction scope/yields and reduce its selectivity. Related methods from other groups have emerged for β‐lactam or azetidine synthesis in subsequent years, again using UV light to form the excited triplet states.^[14]^

**Scheme 1:**
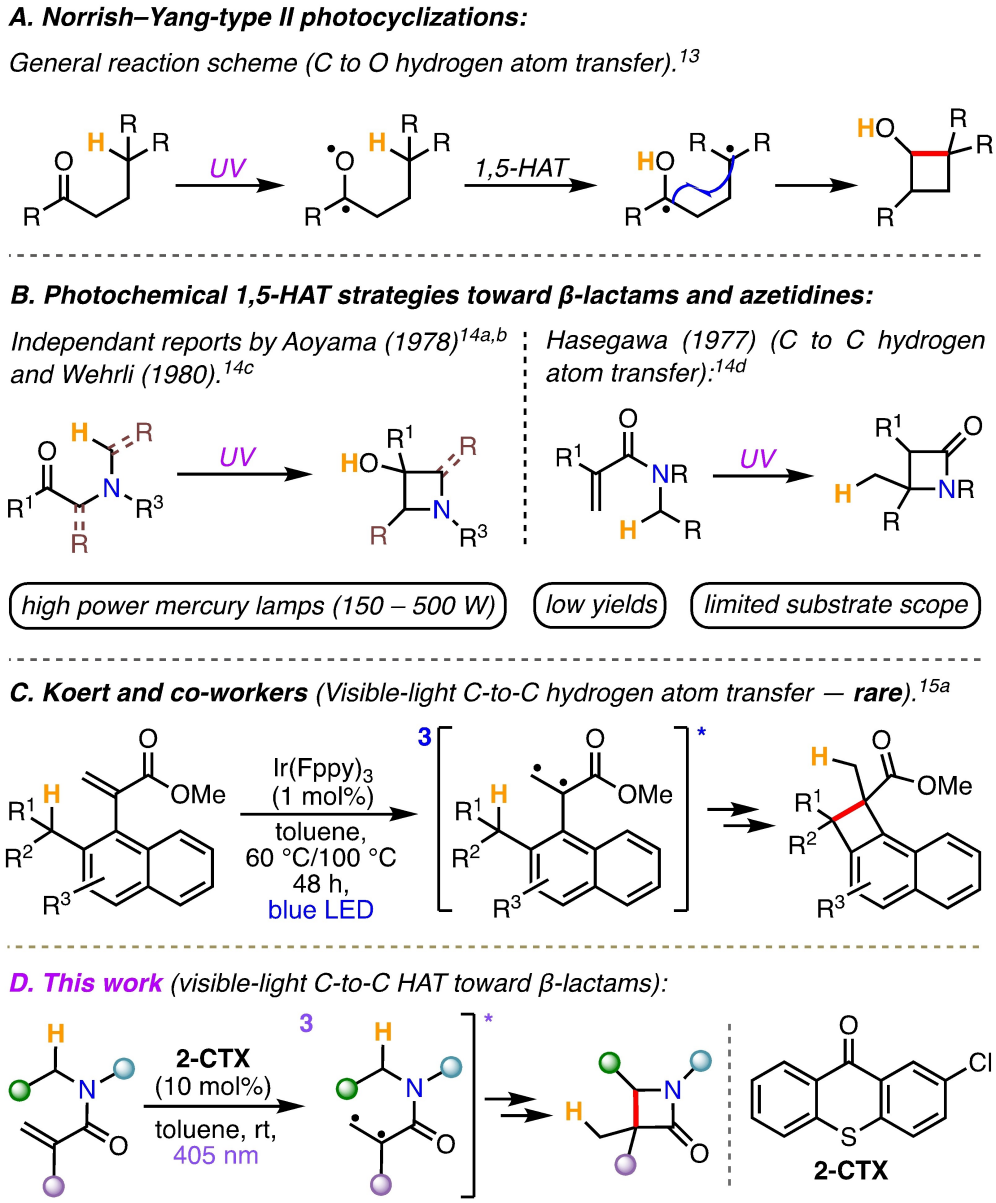
A–C) Previous literature reports on Norrish–Yang type chemistry and related variations. D) This work.

Visible‐light‐mediated Norrish–Yang‐type reactions are rare. To the best of our knowledge, the first published report to make β‐lactam products feature in Sarpong and co‐worker's study, which is focused on keto‐amide cyclization under blue light irradiation.^[14f]^ But, a notable variation using visible‐light by Koert and co‐workers must be highlighted (Scheme 1C).^[15a]^ Here they reported carbon‐to‐carbon 1,5‐HAT (rather than the typical carbon to oxygen 1,5‐HAT of the Norrish‐Yang cyclization)^[15]^ through EnT mediated olefin triplet sensitization (rather than a triplet sensitized ketone).^[16]^ In fact, catalytic carbon‐to‐carbon HAT by any method are still relatively underexplored.^[11]^ Also, Bach and co‐workers recently reported a related β‐lactam forming reaction, to form β spirocyclic azetidine‐3,3′‐indolines via energy transfer triplet‐sensitized excitation of 3‐oxo‐indole precursors.^[15b]^

Considering the remarkable advances in visible‐light‐mediated energy transfer (EnT) catalysis in recent years,^[17]^ we recognized an opportunity to develop a novel, visible‐light EnT method for β‐lactam synthesis. Herein, we report a convenient room‐temperature and metal‐free synthesis of β‐lactams from simple and easily obtained acrylamide precursors, under visible‐light irradiation. The reaction involves an energy transfer mediated C(sp^3^)−H functionalization and proceeds via a rare carbon‐to‐carbon HAT (Scheme 1D). Notably, the hydrogen atom donor moiety was modifiable to include heteroaromatic as well as non‐aromatic moieties—enabling facile access to a wide range of valuable β‐lactam products.

Our studies commenced using model acrylamide **1 a**, based on conditions reported in our previous work (Table 1, entry 1).^[18]^ Thus, reacting acrylamide **1 a** and photosensitizer 2‐chlorothioxanthone (**2‐CTX**) under irradiation at 405 nm, the corresponding β‐lactam **4 a** was obtained in 84 % yield. Switching the solvent to ethanol afforded **4 a** in a reduced 72 % yield (entry 2) while the use of toluene as a solvent was optimal, affording **4 a** in quantitative yield (entry 3), and hence these conditions were taken forward. Other photosensitizers were also investigated but produced inferior results comparatively; the unsubstituted thioxanthone (**TX**) afforded **4 a** in 75 % yield (entry 4) and **4‐CzIPN** produced **4 a** in 84 % yield (entry 5). Diastereomeric ratios were determined by ^1^H NMR (see later for discussion) and were found to be between 1.1 : 1–1.7 : 1 dr. It is noteworthy that the formation of the corresponding benzoazepinone (Table 1, **7**), which may have been expected to form via radical addition to the phenyl substituent,^[18]^ or polymerization of the acrylamide starting material, was not observed in any case.

**Table 1:** Optimization studies.

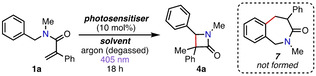				
entry	photosensitizer	solvent	yield [%]^[a]^	dr^[b]^
1	**2‐CTX**	TFE : CHCl_3_	84	1.2 : 1
2	**2‐CTX**	EtOH	72	1.1 : 1
3	**2‐CTX**	toluene	>99	1.5 : 1
4	**TX**	toluene	75	1.5 : 1
5	**4‐CzIPN**	toluene	84	1.7 : 1

[a] Determined by ^1^H NMR using 1, 3, 5‐trimethoxybenzene as an internal standard. [b] Determined by ^1^H NMR analysis of the crude reaction mixture.

We next explored the scope of the reaction (Scheme 2). In exploring the R^1^/R^2^ scope, monosubstituted aromatics **4 a**–**4 h**, bearing alkyl groups, halogens, electron‐releasing groups, as well as electron‐withdrawing groups, were obtained in 49–97 % yield. Products featuring aromatic boron esters could also be synthesized, producing **4 i** in 63 % yield. Di‐ and tri‐substituted products were also suitable, affording **4 j**–**4 l** in 81–97 % yield. The naphthyl derived β‐lactam **4 m** was obtained in 75 % yield and product **4 n**, featuring a 1,5‐HAT from a tertiary carbon centre, was produced in 88 % yield. Modification of the aromatic heterocycle was also possible, with indole‐, furan‐, and thiophene‐based products **4 o**–**4 q** formed in 52 %, 77 % and 97 % yields, respectively. Pleasingly, extending the 1,5‐HAT beyond benzylic amines is possible, with allylic, propargylic, and nitrile products **4 r**–**4 t** obtained in 60 %, 40 %, and 85 % yield, respectively. No potential allylic rearrangement products (such as a δ‐lactam) were observed. The reaction is also possible using an amine lacking a radical‐stabilising π‐system (diisopropylamine), with product **4 u** formed in 52 % yield. This case presumably proceeds via a tertiary radical intermediate, and apparently marks the limit of the method, as the related product **4 v** (utilising diethylamine—which would require reaction via an unstabilized secondary radical intermediate) was not obtained. Modification of the protecting group on the nitrogen atom was also suitable (R^3^ substituent), with products **5 a**–**5 d** obtained in 52–98 % yield. On the other hand, unprotected nitrogen product **5 e** was not obtained. Variation of the acrylic acid moiety (R^4^/R^5^) was also compatible with this chemistry, successfully affording β‐lactams **6 a**–**6 e** in 53–95 % yield. Some limitations of the method were also observed; for example, ferrocene‐substituted and cyclic modifications to R^1^/R^2^ did not lead to formation of the corresponding products **4 w**–**4 y**. Acrylic acid moieties featuring nonaromatic substituents (**6 f** and **6 g**), were also not obtained. Additionally, variation to the benzylic ester did not produce β‐butyrolactone **8**. In all of these cases, unreacted starting material was recovered.

**Scheme 2:**
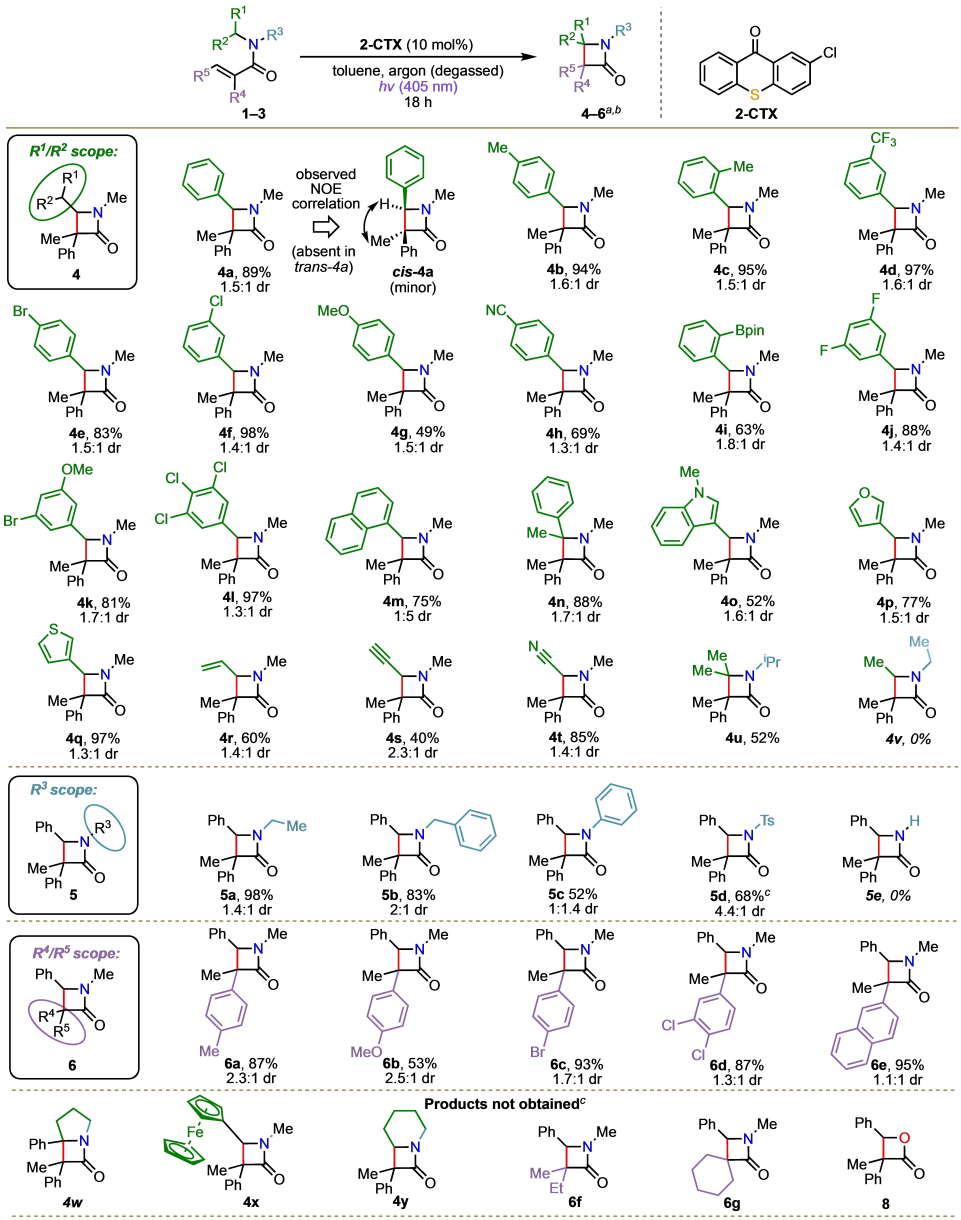
Reaction scope. ^[a]^ Isolated yields. ^[b]^ Diastereomeric ratio determined by ^1^H NMR analysis of the crude reaction mixture and are given as the *trans:cis* ratio. ^[c]^ Starting material recovered.

Notably, in most cases the diastereomeric products were fully separable using standard silica gel chromatography remarkably easily, with the *trans*‐isomer typically obtained in modest excess. The presence (*cis*) or absence (*trans*) of an NOE correlation between the C3‐methyl group and C4‐hydrogen atom proved to be a reliable method to assign the relative stereochemistry, exemplified in Scheme 2 for β‐lactam ***cis‐*** **4 a**. In addition, the *trans*/*cis* stereochemistry could usually be assigned reliably based on the chemical shift of the C3‐methyl resonances in the ^1^H NMR data, with characteristic shifts of the methyl group observed, proposed to be as a result shielding/deshielding effects of the adjacent C4‐aryl group (see the Supporting Information).

A proposed mechanism of the reaction is shown in Scheme 3A. Following energy transfer from the photoexcited 2‐CTX*, acrylamide **1 a** is promoted to its corresponding triplet excited state ^**3**^ A*_s‐trans_. A subsequent carbon‐to‐carbon 1,5‐HAT affords triplet intermediate ^**3**^ B* which, following intersystem crossing (ISC) back to its singlet state ^**1**^ B* and radical‐radical coupling produces β‐lactam **4 a**. Deuterium labelling experiments support this mechanistic proposal, based on 100 % deuterium incorporation in product ***d***_***2***_ **‐4 a** when deuterated starting material ***d***_***2***_ **‐1 a** was reacted under the standard conditions, in both toluene as well as ethanol (Scheme 3B). A competition experiment was also performed (between **1 a** and **d_2_‐1 a**), which indicated a kinetic isotope effect of ≈2.2 (see the Supporting Information); this primary kinetic isotope effect is also consistent with the proposed mechanism (Scheme 3C). Additionally, this latter experiment (Scheme 3C)—also representing a cross‐over experiment—suggests that a chain mechanism proceeding via intermediate **4‐exo‐*I*** is unlikely as this would lead to mixtures of mono‐ and di‐deuterated products as a result of HAT from competing starting material and/or possibly the solvent. This was not observed.

**Scheme 3:**
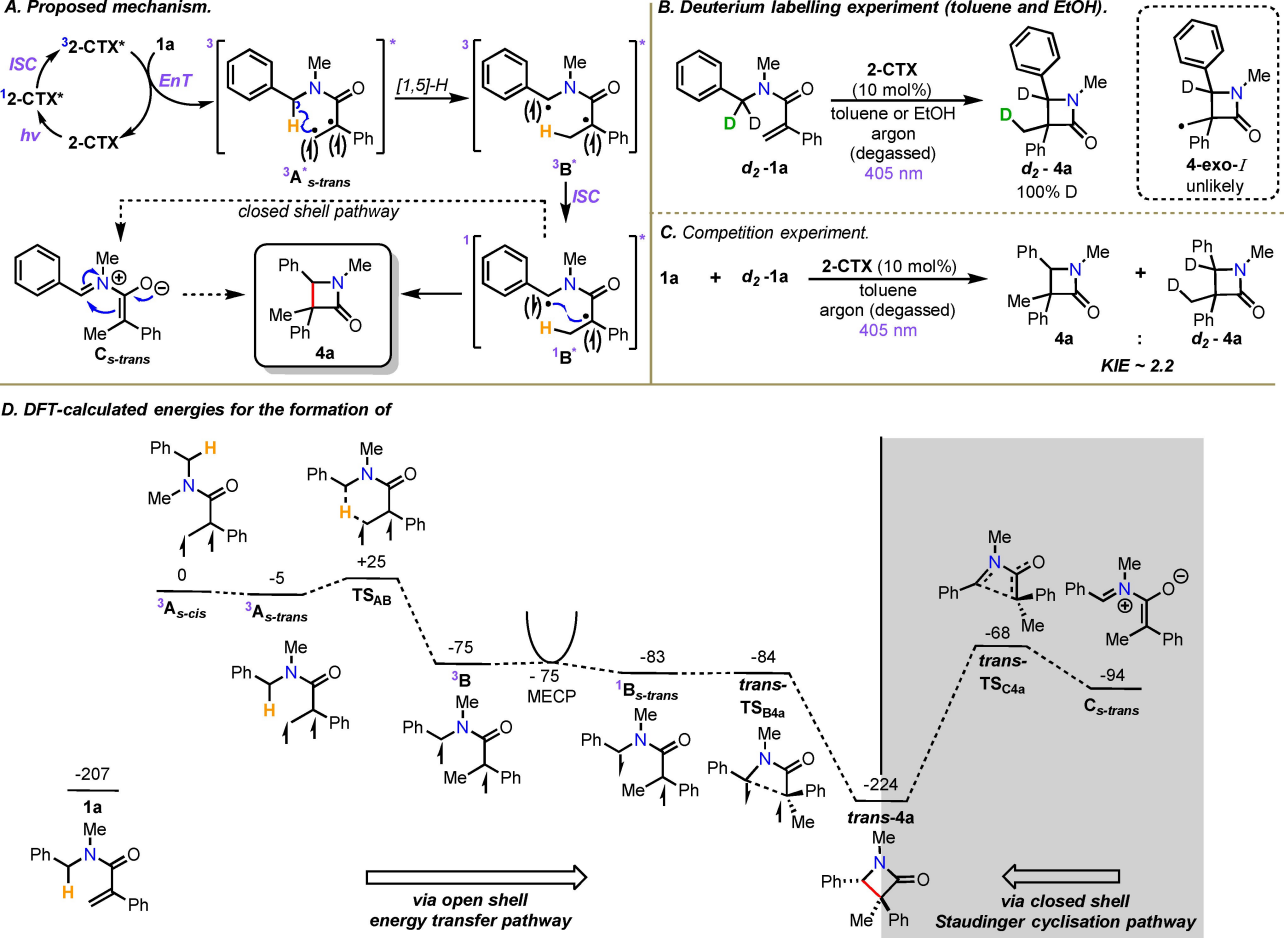
A) Proposed mechanism. B–C) Deuterium labelling studies. D) DFT studies. Energies are Gibbs free energies at 298.15 K in kJ mol^−1^ calculated at the D3(BJ)‐UB3LYP/def2‐TZVPP//UB3LYP/def2‐SVP level of theory, with PCM solvent correction in toluene. The MECP structure and energy was found using Orca, at the D3(BJ)‐UB3LYP/G/def2‐TZVPP//UB3LYP/G/def2‐SVP level of theory, which was compared with Orca calculated energies of ^3^B and ^1^B_*s‐trans*_ (see Supporting Information for further details).

An alternative closed‐shell pathway, via zwitterionic intermediate **C_s‐*trans*_** (i.e. a Staudinger cyclization adduct), was also considered to be a potentially viable pathway (Scheme 3A, dotted arrow).[^4c^, ^19^] Therefore, to gain further insight into both possibilities, DFT calculations were performed (see the Supporting Information for full details). First, the open‐shell energy transfer pathway was considered (Scheme 3D, unshaded part). The conversion of **1 a** into **4 a** was found to be thermodynamically favourable, with the two potential diastereomers ***trans*** **‐4 a** (Scheme 3D) and ***cis*** **‐4 a** (not shown) being very close in energy. It is proposed that energy transfer from the excited state of the **2‐CTX** photocatalyst occurs to give ^**3**^ **A_s‐*cis*_**: this state was located at 207 kJ mol^−1^ higher in energy than **1 a**. Although care must be taken with quantitative comparisons, the triplet energy of **2‐CTX** ^[20]^ is 260 kJ mol^−1^ implying that it is indeed capable of energy transfer to generate ^**3**^ **A**. A 1,5‐hydrogen migration can occur from ^**3**^ **A_s‐*trans*_** through a low energy transition state (**TS_AB_**) to give ^**3**^ **B**. No viable routes to form the lactam ring directly from ^**3**^ **B** were obtained and such a process would be spin‐forbidden. Instead, conversion into the singlet is proposed to occur, and an MECP was located at −75 kJ mol^−1^. The singlet diradical ^**1**^ **B_s‐*trans*_**, was located at −83 kJ mol^−1^ (with respect ^**3**^ **A_s‐*cis*_**), which can then undergo an essentially barrierless carbon‐carbon bond formation through ***trans*** **‐TS_B4a_** to give ***trans*** **‐4 a** (Scheme 3D).^[21]^ The formation of ***cis*** **‐4 a** was found to occur through an almost identical and isoenergetic process (not shown, see the Supporting Information). These data would suggest that in this case, lactam formation is expected to proceed without significant diastereoselectivity, which is in line with the experimental results. Although the formation of ***trans‐4 a*** from ^**3**^ **B** is predicted to be essentially barrierless, a mechanistic route corresponding to the closed‐shell Staudinger cyclization pathway was also investigated (Scheme 3D, shaded part). In this case, the closed‐shell singlet (**C_s‐*trans*_**) undergoes lactam formation through ***trans*** **‐TS_C4a_** which lies uphill at −68 kJ mol^−1^. This is consistent with the calculated open‐shell pathway being the dominant mechanistic pathway.^[22]^

In conclusion, we have developed a convenient and straightforward procedure for the synthesis of β‐lactams from simple and easily obtained acrylamide precursors using visible‐light‐mediated energy transfer. The reaction is proposed to proceed via a rare carbon‐to‐carbon 1,5‐HAT and enables a C(sp^3^)−H functionalization. The proposed mechanism is supported by deuterium labelling experiments and computational studies. The reaction has broad scope and is typically high yielding, offering a fast and atom‐economical route to a wide range of biologically important β‐lactams.

## Conflict of interest

The authors declare no conflict of interest.

## Supporting information

As a service to our authors and readers, this journal provides supporting information supplied by the authors. Such materials are peer reviewed and may be re‐organized for online delivery, but are not copy‐edited or typeset. Technical support issues arising from supporting information (other than missing files) should be addressed to the authors.

Supporting Information

## Data Availability

The data that support the findings of this study are available in the supplementary material of this article.
