# Supplementary material for: Visible‐Light‐Mediated Energy Transfer Enables the Synthesis of β‐Lactams via Intramolecular Hydrogen Atom Transfer
Source: Angew Chem Int Ed Engl. 2022 Oct 26;61(48):e202213086. doi: 10.1002/anie.202213086 (PMC9828223; doi:10.1002/anie.202213086)
Supplement: Supplementary file 1 — Supporting Information [file ANIE-61-0-s001.pdf]

Angewandte Chemie
Eine Zeitschrift der Gesellschaft Deutscher Chemiker

Supporting Information

Visible-Light-Mediated Energy Transfer Enables the Synthesis of β-Lactams via Intramolecular Hydrogen Atom Transfer

*M. J. Oddy, D. A. Kusza, R. G. Epton, J. M. Lynam, W. P. Unsworth, W. F. Petersen**

Supporting Information

Table of Contents

1. General information……S3
2. Deuterium labelling studies……S4
3. DFT calculations……S6
4. General procedure A: Synthesis of acrylamides (**1–3**)……S47
5. General procedure B: Synthesis of acrylamides (**1–3**) via reductive amination……S47
6. Characterization data of acrylamides **1**……S47
7. Characterization data of acrylamides **2**……S59
8. Characterization data of acrylamides **3**……S62
9. General procedure C: Synthesis of β-lactams (**4–6**) via **2-CTX** mediated energy transfer catalysis……S66
10. Stereochemical assignment of *trans/cis* β-lactam products by ^{1}H NMR……S66
11. Characterization data of β-lactams **4**……S66
12. Characterization data of β-lactams **5**……S78
13. Characterization data of β-lactams **6**……S80
14. References……S83
15. NMR Spectra……S85
    - *Acrylamides **1***……S85
    - *Acrylamides **2***……S112

- *Acrylamides **3***………………………………………………………………………………………….S117
- β-*lactams **4***………………………………………………………………………………………….S125
- β-*lactams **5***………………………………………………………………………………………….S165
- β-*lactams **6***………………………………………………………………………………………….S171

1. General information

Unless otherwise specified, all reagents and photoinitators were purchased from commercial sources and used without further purification. Anhydrous solvents were obtained using a solvent purification system drying over 3Å molecular sieves. Photochemical reactions were carried out using an EvoluChem™ PhotoRedOx Box under irradiation at 450 nm using an EvoluChem™ LED (30 W). Standard borosilicate glass vessels were used. Where required, reactions were heated using a standard stirrer/metal heating block combination fitted with a temperature probe. ^{1}H NMR and ^{13}C NMR spectra were recorded on Varian Mercury 300 MHz (75 MHz for ^{13}C), Bruker 400 MHz (101 MHz for ^{13}C), Bruker 600 MHz (151 MHz for ^{13}C) instruments. All spectral data were acquired at 295 K. Chemical shifts are reported in parts per million (ppm, δ), downfield from tetramethylsilane (TMS, δ = 0.00 ppm), and are referenced to residual solvent [$CDCl_3$, δ = 7.26 ppm (^{1}H) and 77.16 ppm (^{13}C)]. Coupling constants (*J*) are reported in Hertz (Hz). The multiplicity abbreviations used are br broad, s singlet, d doublet, t triplet, q quartet, m multiplet, app apparent. Infrared (IR) spectra were recorded on a PerkinElmer Spectrum 100 FT-IR spectrometer. High-resolution mass spectra were obtained from the University of Stellenbosch Mass Spectrometry Service and recorded in electrospray positive mode with a time-of-flight analyzer system on a Waters Synapt G2 machine. Melting points were determined using a Reichert-Jung Thermovar hot-stage microscope and are uncorrected. Thin-layer chromatography was carried out on silica gel $60F_{254}$ precoated aluminum foil sheets (unless otherwise stated) and were visualized using UV light (254 nm) or staining with acidic *p*-anisaldehyde or ninhydrin solutions. Flash column chromatography was carried out using silica gel 60 (unless otherwise stated), eluting with the specified solvent system.

2. Deuterium experiments.

A. Labelling study.

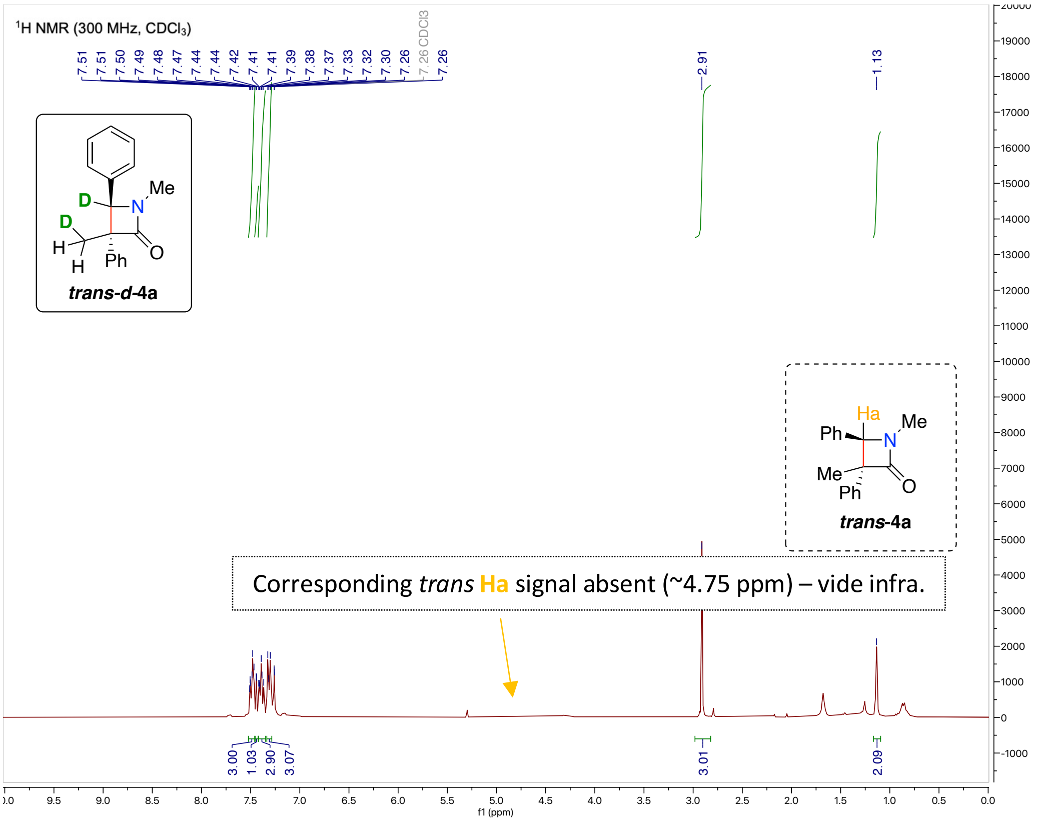

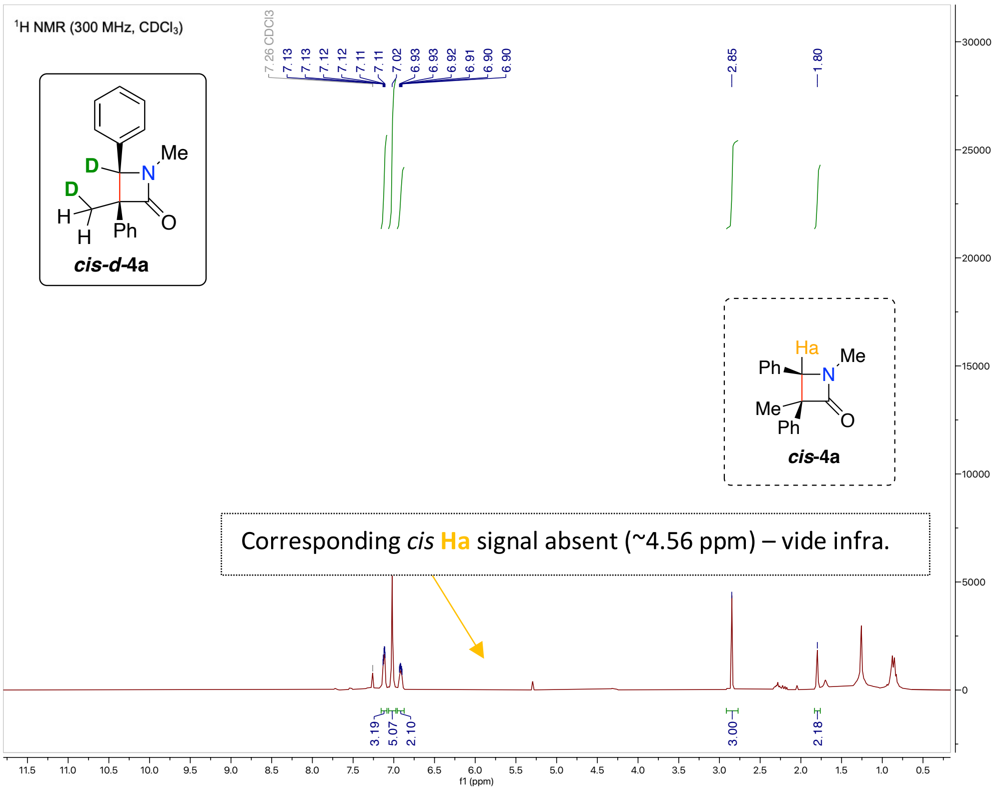

Figure S1. ^{1}H NMR spectra of *trans*-*d*-**4a** and *cis*-*d*-**4a**.

B. Competition experiment.

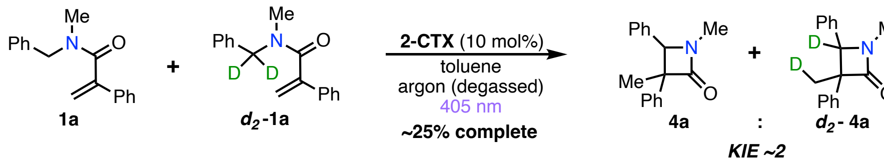

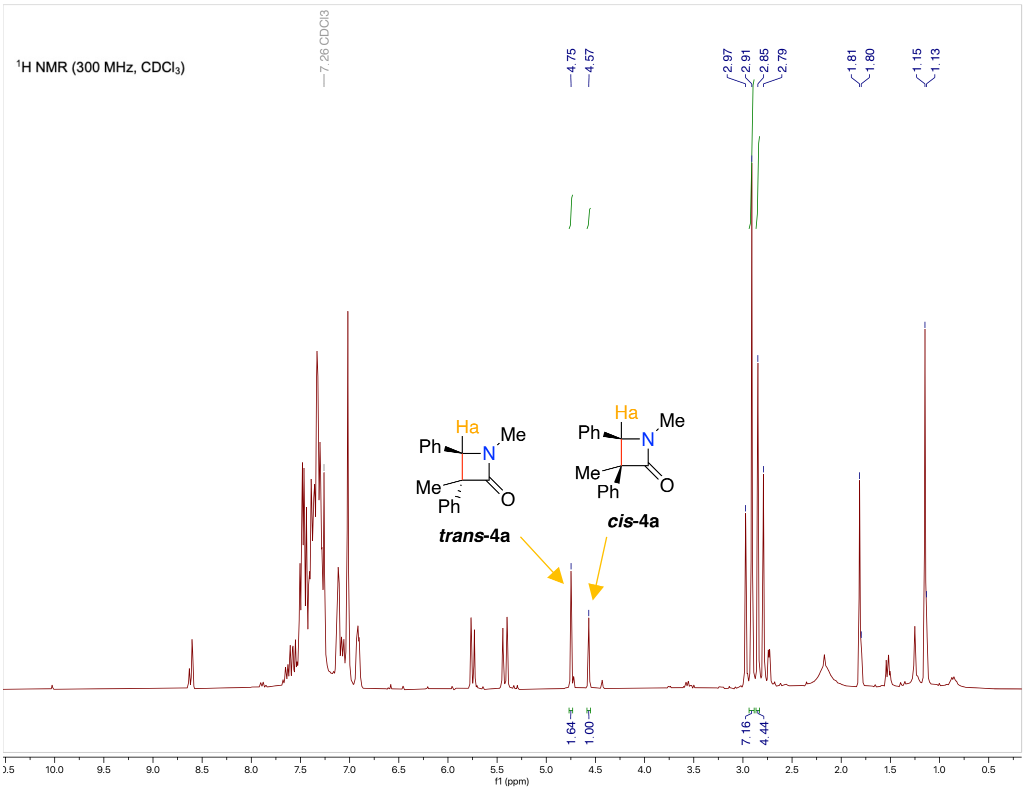

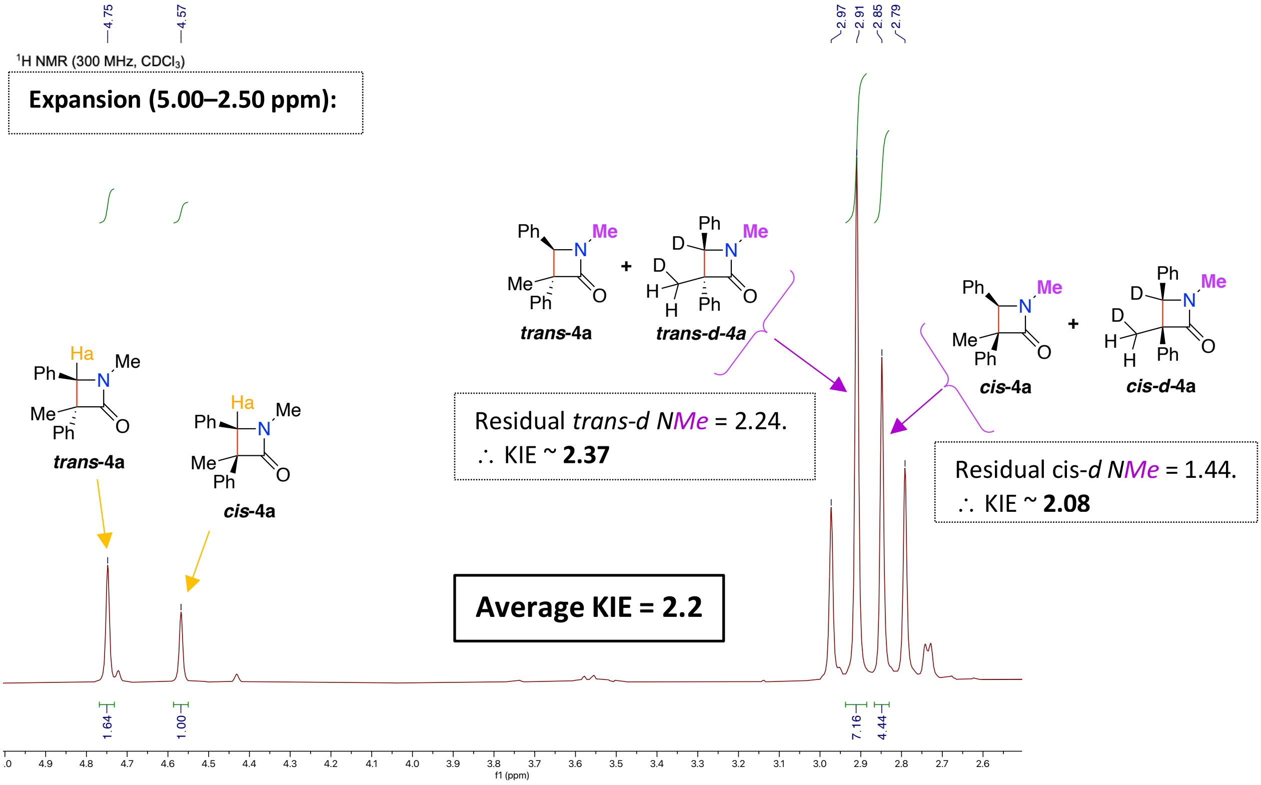

Figure S2. ^{1}H NMR analysis for KIE estimation.

3. DFT calculations — computational method.

All calculations were performed using the Gaussian 09, Revision D.01 package.[1]
Initial geometry optimisations were performed at the UB3LYP/def2-SVP level,[2–6] followed by frequency calculations at the same level. Transition states were located by performing a relaxed bond distance scan of the atoms involved in the transition state. The geometry of the structure close to the anticipated transition state was then resubmitted for an unconstrained transition state. A frequency calculation was then performed on the optimised transition-state structure. All minima were confirmed as such by the absence of imaginary frequencies and all transition states were identified by the presence of only one imaginary frequency. Intrinsic Reaction Coordinate (IRC) analysis confirmed that transition states were connected to the appropriate minima. Single-point calculations on the UB3LYP/def2-SVP optimised geometries were performed at the UB3LYP/def2-TZVPP level of theory,[2–6] with dispersion effects modelled with Grimme's D3 method with additional Becke-Johnson damping. [7] The D3(BJ)-UB3LYP/def2-TZVPP SCF energies were corrected for their zero-point energies, thermal energies, and entropies at 298.15 K (obtained from the UB3LYP/def2-SVP-level frequency calculations). Optimisations were performed with tight convergence criteria and no symmetry constraints were applied. An ultrafine integral grid was used for all calculations. Where used, solvent corrections were applied with the Polarisable Continuum Model (PCM) using the integral equation formalism variant (IEFPCM) in toluene.[8] In the geometry optimisations and single-point calculations of the singlet diradical species (**^{1}B*$_{s\text{-}trans}$***, **^{1}B*$_{s\text{-}cis}$***, ***trans*-TS$_{B4a}$** and ***cis*-TS$_{B4a}$**), "geom=mix" was required in the input.

Energies in Hartrees, spin expectation (<S*S>) values (after annihilation of spin-contaminant), xyz coordinates and the imaginary frequency present in the transition state structures are reported.

1a

SCF Energy: UB3LYP/def2-SVP = -787.450325

SCF Energy: D3(BJ)-UB3LYP/def2-TZVPP = -788.389789

SCF Energy: D3(BJ)-UB3LYP/def2-TZVPP (Toluene correction) = -788.393752

Zero-point correction = 0.298317

Thermal correction to Gibbs Free Energy = 0.251157

<S*S> Value: UB3LYP/def2-SVP = 0.0000

<S*S> Value: D3(BJ)-UB3LYP/def2-TZVPP = 0.0000

<S*S> Value: D3(BJ)-UB3LYP/def2-TZVPP (Toluene correction) = 0.0000

C	-4.75022500	0.71462400	-0.95965500
C	-3.99215300	-0.39236800	-0.56130600
C	-2.85892600	-0.23332000	0.24854100
C	-2.49652300	1.06221400	0.65761100
C	-3.25029000	2.16751400	0.25893400
C	-4.37929500	1.99759200	-0.55136700
H	-5.63194800	0.57211800	-1.59003600
H	-4.29087200	-1.39580900	-0.88019500
H	-1.62044800	1.18963800	1.29825200
H	-2.95922600	3.16898800	0.58686100
H	-4.96914200	2.86446400	-0.86041700
C	-2.03074800	-1.42916700	0.68245700
H	-1.87872800	-1.40413300	1.76944000
H	-2.56266800	-2.36120200	0.42739600
N	-0.69820400	-1.48957300	0.07116100
C	-0.65350500	-1.88048100	-1.32769800
H	0.33436600	-1.68226600	-1.75626400
H	-0.88049700	-2.95462300	-1.45576800
H	-1.39976500	-1.30374600	-1.89896300
C	0.37585300	-1.07587900	0.81576000
O	0.24407200	-0.56187300	1.92204800
C	1.76724100	-1.31273500	0.26037300
C	2.19473900	-2.57628600	0.09178100
H	3.23187400	-2.79807900	-0.17385400
C	2.63937400	-0.12794900	0.02486000
C	3.54109800	-0.12670000	-1.05753800
C	2.59547800	1.00548600	0.86027000
C	4.38011700	0.96404000	-1.29205700
H	3.56865600	-0.98340000	-1.73523300
C	3.43693100	2.09441600	0.62217500
H	1.90838900	1.00825300	1.70668200
C	4.33225900	2.08020600	-0.45148500
H	5.06753200	0.94546500	-2.14188600
H	3.39467700	2.96125200	1.28698900

H	4.98640600	2.93636100	-0.63552300
H	1.52770600	-3.42852100	0.24806900

***trans*-4a**

SCF Energy: UB3LYP/def2-SVP = -787.458673
SCF Energy: D3(BJ)-UB3LYP/def2-TZVPP = -788.397679
SCF Energy: D3(BJ)-UB3LYP/def2-TZVPP (Toluene correction) = -788.402222

Zero-point correction = 0.298457
Thermal correction to Gibbs Free Energy = 0.253260

<S*S> Value: UB3LYP/def2-SVP = 0.0000
<S*S> Value: D3(BJ)-UB3LYP/def2-TZVPP = 0.0000
<S*S> Value: D3(BJ)-UB3LYP/def2-TZVPP (Toluene correction) = 0.0000

C	4.10874700	-0.14919000	0.69860200
C	2.87077300	0.43204700	0.41075600
C	1.88593800	-0.28471300	-0.28828000
C	2.17996100	-1.59379600	-0.70240400
C	3.41387300	-2.18028900	-0.40963800
C	4.38325100	-1.45888900	0.29354500
H	4.86393400	0.42541600	1.24125900
H	1.43438000	-2.15869800	-1.26983900
H	3.62224300	-3.20128200	-0.73976600
H	5.35163900	-1.91290900	0.51838500
C	0.53235700	0.31186900	-0.59637400
H	0.12238000	-0.17316600	-1.49946600
H	2.66556700	1.45776000	0.72655500
C	-0.58901400	0.40827700	0.54051700
C	-0.55197700	1.92705900	0.22503500
O	-1.16022400	2.90356000	0.59566700
C	-0.14957700	0.07593000	1.97033600
H	-0.92860500	0.40497000	2.67456100
H	-0.01050800	-1.00902600	2.09601600
H	0.79106400	0.57705400	2.24065600
C	-1.90104100	-0.28221500	0.19999100
C	-1.91381400	-1.63778100	-0.17053500
C	-3.12613800	0.39719500	0.30049600
C	-3.11527100	-2.29571400	-0.44439900
H	-0.97229900	-2.18952700	-0.24146800
C	-4.32891200	-0.26219800	0.02650900
H	-3.13019600	1.44929600	0.59224200
C	-4.32925700	-1.60829500	-0.34767300
H	-3.10302900	-3.34997400	-0.73367900
H	-5.27257300	0.28413000	0.10567500
H	-5.27045000	-2.12095200	-0.56235000
N	0.46122800	1.77263800	-0.68206600
C	1.02705000	2.70146500	-1.62688100
H	0.66572100	3.70712400	-1.36688100

H	2.12881600	2.69747400	-1.58935900
H	0.71342700	2.47078900	-2.66102900

***cis*-4a**

SCF Energy: UB3LYP/def2-SVP = -787.457606

SCF Energy: D3(BJ)-UB3LYP/def2-TZVPP = -788.398236

SCF Energy: D3(BJ)-UB3LYP/def2-TZVPP (Toluene correction) = -788.402882

Zero-point correction = 0.298332
Thermal correction to Gibbs Free Energy = 0.253157

<S*S> Value: UB3LYP/def2-SVP = 0.0000

<S*S> Value: D3(BJ)-UB3LYP/def2-TZVPP = 0.0000
<S*S> Value: D3(BJ)-UB3LYP/def2-TZVPP (Toluene correction) = 0.0000

C	2.59855500	-1.16468100	-1.44783900
C	1.63460000	-0.22933400	-1.06621600
C	1.59564100	0.26237900	0.24866000
C	2.55087100	-0.19962200	1.16669000
C	3.51340500	-1.14047200	0.78916800
C	3.53957500	-1.62585300	-0.52102100
H	2.61425500	-1.53810400	-2.47502300
H	2.54290700	0.18608200	2.19073300
H	4.24925200	-1.48894800	1.51857000
H	4.29361900	-2.35793300	-0.82122800
C	0.55620300	1.26614800	0.68579700
H	0.88155400	1.72275900	1.63840100
H	0.90059900	0.12329300	-1.79434900
C	-0.99889300	0.88467300	0.75444500
C	-1.16336100	2.02464900	-0.28472200
O	-2.09306200	2.55191300	-0.84961300
C	-1.65251100	1.24957600	2.09507000
H	-2.74765900	1.24799200	1.98727200
H	-1.38584700	0.52044300	2.87630500
H	-1.34450800	2.25255400	2.43307600
C	-1.42050300	-0.50053800	0.29727200
C	-0.94155800	-1.64495500	0.95819000
C	-2.34086900	-0.66818000	-0.75024800
C	-1.35922800	-2.92102700	0.57584500
H	-0.22484500	-1.53996900	1.77635200
C	-2.76067900	-1.94714900	-1.13183500
H	-2.73884100	0.21184800	-1.25919300
C	-2.27112700	-3.07722700	-0.47327900
H	-0.96884200	-3.79819900	1.09862800
H	-3.47827500	-2.05691600	-1.94931300
H	-2.59909900	-4.07591100	-0.77319000
N	0.18007900	2.27641000	-0.30695900
C	0.93772400	3.33089700	-0.92914300
H	0.25913400	3.88585900	-1.59323400

H	1.35182900	4.03184400	-0.18205500
H	1.77163600	2.92743500	-1.52713700

^{3}A$_{s\text{-}cis}$

SCF Energy: UB3LYP/def2-SVP = -787.366905

SCF Energy: D3(BJ)-UB3LYP/def2-TZVPP = -788.305112

SCF Energy: D3(BJ)-UB3LYP/def2-TZVPP (Toluene correction) = -788.309248

Zero-point correction = 0.293826

Thermal correction to Gibbs Free Energy = 0.245615

<S*S> Value: UB3LYP/def2-SVP = 2.0004

<S*S> Value: D3(BJ)-UB3LYP/def2-TZVPP = 2.0003

<S*S> Value: D3(BJ)-UB3LYP/def2-TZVPP (Toluene correction) = 2.0003

C	-4.93313500	0.47111700	-0.89834200
C	-4.08643300	-0.54278200	-0.43553100
C	-2.94382400	-0.23722800	0.31724500
C	-2.66425000	1.11052500	0.60466300
C	-3.50730600	2.12290600	0.14292200
C	-4.64387700	1.80682000	-0.61113800
H	-5.82073000	0.21464400	-1.48277400
H	-4.32310500	-1.58821800	-0.65681300
H	-1.78060000	1.34985200	1.20142000
H	-3.28072300	3.16646900	0.37739300
H	-5.30358700	2.60119900	-0.96986400
C	-2.01742500	-1.33148400	0.81747900
H	-1.80548600	-1.18040400	1.88419700
H	-2.50652000	-2.31329000	0.69745000
N	-0.72245200	-1.38658100	0.13445800
C	-0.71661400	-1.93002300	-1.21073600
H	0.19519100	-1.63155800	-1.74315100
H	-0.78706700	-3.03283600	-1.21662600
H	-1.57423800	-1.52759400	-1.77502200
C	0.37069100	-0.82165400	0.74780000
O	0.26147000	-0.14241600	1.76601100
C	1.73040200	-1.13275500	0.17667800
C	2.06623400	-2.55120700	0.00060100
H	2.04702300	-3.05031500	-0.97475200
C	2.71252800	-0.09617100	0.00420700
C	4.02533000	-0.42606800	-0.44194000
C	2.42770400	1.28347800	0.22670200
C	4.98588000	0.55564000	-0.65570200
H	4.27323200	-1.47548100	-0.61820400
C	3.39571200	2.25603700	0.00838500
H	1.44384400	1.57085400	0.59524200
C	4.68011400	1.90561600	-0.43436700
H	5.98418700	0.27005600	-0.99779800
H	3.15111200	3.30604900	0.18959800

H	5.43545300	2.67727500	-0.60238600
H	2.32703000	-3.16791100	0.86923700

^{3}A$_{s\text{-}trans}$

SCF Energy: UB3LYP/def2-SVP = -787.364855
SCF Energy: D3(BJ)-UB3LYP/def2-TZVPP = -788.304875
SCF Energy: D3(BJ)-UB3LYP/def2-TZVPP (Toluene correction) = -788.309413

Zero-point correction = 0.293216
Thermal correction to Gibbs Free Energy = 0.243935

<S*S> Value: UB3LYP/def2-SVP = 2.0004
<S*S> Value: D3(BJ)-UB3LYP/def2-TZVPP = 2.0004
<S*S> Value: D3(BJ)-UB3LYP/def2-TZVPP (Toluene correction) = 2.0004

C	-2.78765800	-2.23612100	-0.55133600
C	-1.92434900	-1.17978100	-0.25485700
C	-2.37252900	-0.07695300	0.49029400
C	-3.70273000	-0.05757900	0.92952100
C	-4.56967100	-1.11633900	0.63741300
C	-4.11405900	-2.20803300	-0.10498900
H	-2.42336100	-3.08841500	-1.13082300
H	-0.88882200	-1.21054500	-0.60507300
H	-4.06643000	0.79688800	1.50828400
H	-5.60446800	-1.08431500	0.98827100
H	-4.78922300	-3.03566500	-0.33695500
C	-1.42147700	1.06012800	0.83338400
H	-1.96192200	1.81740600	1.42980600
H	-0.61422100	0.66691100	1.46591900
N	-0.81428700	1.69486100	-0.32812900
C	-1.71739100	2.31919400	-1.28193600
H	-1.11785700	2.81177700	-2.05604200
H	-2.37750100	1.57616500	-1.75984200
H	-2.35220900	3.06471800	-0.77184600
C	0.53189700	1.66024500	-0.61246900
O	0.98575900	2.04938500	-1.68109200
C	1.47576200	1.18337200	0.47024500
C	1.57629800	2.02664600	1.66641500
H	1.09146800	1.77232600	2.61647700
C	2.37368200	0.09327100	0.21290400
C	3.32647600	-0.30334600	1.19554000
C	2.33635400	-0.65683700	-1.00013900
C	4.17585200	-1.38310300	0.98362100
H	3.38095100	0.25691800	2.13202300
C	3.19080300	-1.73429300	-1.20030500
H	1.64818000	-0.35729700	-1.79149000
C	4.11504100	-2.11021500	-0.21368400
H	4.89561900	-1.66420200	1.75706500
H	3.14485000	-2.28835100	-2.14167000

H	4.78396900	-2.95830600	-0.37894200
H	2.11111300	2.98334500	1.62273700

TS_{AB}

SCF Energy: UB3LYP/def2-SVP = -787.355941

SCF Energy: D3(BJ)-UB3LYP/def2-TZVPP = -788.292443

SCF Energy: D3(BJ)-UB3LYP/def2-TZVPP (Toluene correction) = -788.296488

Zero-point correction = 0.289844

Thermal correction to Gibbs Free Energy = 0.242416

<S*S> Value: UB3LYP/def2-SVP = 2.0005

<S*S> Value: D3(BJ)-UB3LYP/def2-TZVPP = 2.0005

<S*S> Value: D3(BJ)-UB3LYP/def2-TZVPP (Toluene correction) = 2.0005

Imaginary Frequency (cm^{-1}) = -1178.16

C	-3.19286900	-1.41536100	-1.65506500
C	-2.33743900	-0.52304900	-1.00525900
C	-2.62964300	-0.06547500	0.29299400
C	-3.80215300	-0.53039300	0.91908100
C	-4.65380500	-1.42537100	0.27071400
C	-4.35280100	-1.87245000	-1.02083900
H	-2.95182800	-1.75617800	-2.66542500
H	-1.43528800	-0.16917900	-1.50982700
H	-4.04794900	-0.18023900	1.92629100
H	-5.56099800	-1.77204800	0.77265200
H	-5.02098500	-2.57053700	-1.53125400
C	-1.70385400	0.84031700	1.03432000
H	-2.22048200	1.38810000	1.84131500
H	-0.86885700	0.14715400	1.63754900
N	-0.85330000	1.70967300	0.26966600
C	-1.47728400	2.90516400	-0.27834900
H	-0.72151100	3.46520200	-0.84084200
H	-2.31168300	2.63803800	-0.94847600
H	-1.87773900	3.53953500	0.53264700
C	0.50233200	1.49430000	0.02982500
O	1.13065200	2.28082800	-0.67476500
C	1.15176800	0.30742900	0.67271900
C	0.48409100	-0.33749400	1.82860100
H	0.30076300	-1.41873900	1.77704100
C	2.44939300	-0.16862300	0.24297400
C	3.09496300	-1.20000900	0.98798600
C	3.13219400	0.30883100	-0.91575100
C	4.32938000	-1.71390000	0.60863200
H	2.61317500	-1.59393200	1.88433700
C	4.36267300	-0.21961100	-1.29048100
H	2.67745200	1.10582400	-1.49832300
C	4.97494700	-1.22998500	-0.53670300

H	4.79439900	-2.50008100	1.20926700
H	4.85698300	0.16553700	-2.18638000
H	5.94403600	-1.63580800	-0.83793100
H	0.76704300	0.00709600	2.83541100

^{3}B

SCF Energy: UB3LYP/def2-SVP = -787.396876

SCF Energy: D3(BJ)-UB3LYP/def2-TZVPP = -788.335052

SCF Energy: D3(BJ)-UB3LYP/def2-TZVPP (Toluene correction) = -788.339131

Zero-point correction = 0.294896
Thermal correction to Gibbs Free Energy = 0.247075

<S*S> Value: UB3LYP/def2-SVP = 2.0016

<S*S> Value: D3(BJ)-UB3LYP/def2-TZVPP = 2.0015

<S*S> Value: D3(BJ)-UB3LYP/def2-TZVPP (Toluene correction) = 2.0014

C	4.96912700	0.07301600	0.44658300
C	3.64619600	0.50038400	0.39196200
C	2.65273900	-0.27019800	-0.28428900
C	3.08063100	-1.49679500	-0.87926900
C	4.40410700	-1.90924200	-0.81926700
C	5.36603000	-1.12648300	-0.16040000
H	5.70493900	0.68114600	0.97992900
H	2.34249500	-2.11525300	-1.39754400
H	4.69705600	-2.85161800	-1.28987400
H	6.40785400	-1.45176700	-0.11445800
C	1.27486400	0.07092700	-0.37273500
H	0.57618000	-0.70040900	-0.70515900
H	3.36795500	1.42198800	0.90295200
C	-1.35554800	0.12323200	0.73928000
C	-0.67774900	1.37016600	0.28078700
O	-1.26042400	2.44732600	0.22966000
C	-0.69492800	-0.68950700	1.82132600
H	-1.34019100	-0.73936000	2.71614400
H	-0.50168200	-1.73212700	1.51174000
H	0.26486000	-0.25532000	2.12917800
C	-2.66910800	-0.21223900	0.25951800
C	-3.34861400	-1.36439900	0.75367500
C	-3.33943900	0.55036400	-0.74367700
C	-4.60455200	-1.72624800	0.27846300
H	-2.87981900	-1.98313400	1.51976000
C	-4.59460400	0.17949300	-1.20994100
H	-2.86563200	1.45064700	-1.13237500
C	-5.23991600	-0.95949800	-0.70705200
H	-5.09634900	-2.61620300	0.68004900
H	-5.08228900	0.78766100	-1.97634500
H	-6.22717500	-1.24516700	-1.07828300
N	0.68381100	1.28822600	-0.05230600
C	1.35855500	2.56489000	-0.30922600
H	1.71504800	3.03298100	0.62267400

H	2.20785200	2.40038900	-0.98168000
H	0.63887100	3.25217100	-0.77226500

[1]B*s-trans*

SCF Energy: UB3LYP/def2-SVP = -787.401321

SCF Energy: D3(BJ)-UB3LYP/def2-TZVPP = -788.339952

SCF Energy: D3(BJ)-UB3LYP/def2-TZVPP (Toluene correction) = -788.344429

Zero-point correction = 0.295511
Thermal correction to Gibbs Free Energy = 0.249036

<S*S> Value: UB3LYP/def2-SVP = 0.1493

<S*S> Value: D3(BJ)-UB3LYP/def2-TZVPP = 0.1326
<S*S> Value: D3(BJ)-UB3LYP/def2-TZVPP (Toluene correction) = 0.1141

C	4.90512600	-0.02994000	0.66096000
C	3.59257000	0.41877700	0.54840000
C	2.67313600	-0.22640900	-0.32619200
C	3.14670900	-1.35346700	-1.05656700
C	4.45933800	-1.79242200	-0.93677400
C	5.35297000	-1.13110100	-0.08131500
H	5.58918000	0.47917800	1.34510900
H	2.45809900	-1.87469600	-1.72723500
H	4.79483100	-2.65833000	-1.51341900
H	6.38541300	-1.47591500	0.01198400
C	1.30494200	0.15671000	-0.49068300
H	0.63935300	-0.54236600	-1.00047700
H	3.26381100	1.25824200	1.16170800
C	-1.30212200	0.07640400	0.59317000
C	-0.68575400	1.35027800	0.18164600
O	-1.29189300	2.41147600	0.05292400
C	-0.58711400	-0.81920300	1.57837900
H	-1.18325800	-0.91286800	2.50391900
H	-0.43309000	-1.84483000	1.20031600
H	0.39562100	-0.42141100	1.85886600
C	-2.67119500	-0.22983400	0.23205500
C	-3.28114600	-1.43411800	0.68686500
C	-3.45448600	0.60235300	-0.62075600
C	-4.57470300	-1.78459200	0.31094800
H	-2.72879200	-2.11106200	1.33885500
C	-4.74472700	0.24156700	-0.99208000
H	-3.03543700	1.54661000	-0.96271100
C	-5.31906100	-0.95126700	-0.53193000
H	-5.00776700	-2.71842500	0.67934900
H	-5.31743300	0.90472400	-1.64599200
H	-6.33583100	-1.22567000	-0.82403200
N	0.71475100	1.31475600	-0.04630800
C	1.38832300	2.61460600	-0.11157400
H	1.62968000	2.99640000	0.89363200

H	2.30605700	2.53015700	-0.70419800
H	0.69461900	3.32511400	-0.58222300

***trans*-TS$_{B4a}$**

SCF Energy: UB3LYP/def2-SVP = -787.399619

SCF Energy: D3(BJ)-UB3LYP/def2-TZVPP = -788.339930

SCF Energy: D3(BJ)-UB3LYP/def2-TZVPP (Toluene correction) = -788.344600

Zero-point correction = 0.294630
Thermal correction to Gibbs Free Energy = 0.249064

<S*S> Value: UB3LYP/def2-SVP = 0.1033

<S*S> Value: D3(BJ)-UB3LYP/def2-TZVPP = 0.0922

<S*S> Value: D3(BJ)-UB3LYP/def2-TZVPP (Toluene correction) = 0.0877

Imaginary Frequency (cm^{-1}) = -86.74

C	4.50662000	-0.29898900	0.73232300
C	3.26512100	0.29420600	0.52330900
C	2.35236800	-0.24170400	-0.42619200
C	2.74734400	-1.40877700	-1.13587900
C	3.98768600	-1.99663800	-0.91657400
C	4.87912100	-1.44434200	0.01518700
H	5.19226000	0.12912200	1.46835800
H	2.05853400	-1.84166900	-1.86651400
H	4.26905000	-2.89304300	-1.47533600
H	5.85451400	-1.90663000	0.18465900
C	1.06340000	0.32775100	-0.66865200
H	0.39271400	-0.17740700	-1.36833500
H	2.97894400	1.17360000	1.10397400
C	-1.09681700	0.28016600	0.72897200
C	-0.63815400	1.62849200	0.27693600
O	-1.30362300	2.65398400	0.28891800
C	-0.37304700	-0.35673500	1.88910100
H	-0.97088900	-0.25634500	2.81498900
H	-0.19480000	-1.43391400	1.73846600
H	0.59881300	0.11992100	2.07034700
C	-2.38392000	-0.22712400	0.30536800
C	-2.90296000	-1.44132200	0.83247600
C	-3.16370300	0.43374400	-0.68585400
C	-4.11347600	-1.96628800	0.38747800
H	-2.34570500	-1.98189000	1.59847300
C	-4.37191200	-0.09715800	-1.12335300
H	-2.81831500	1.38730800	-1.08525200
C	-4.85719900	-1.30113000	-0.59419000
H	-4.48329900	-2.90324100	0.81239900
H	-4.94956700	0.43671700	-1.88256800
H	-5.80875400	-1.71284400	-0.93965200
N	0.66875900	1.58051700	-0.18371900

C	1.34108600	2.83141100	-0.53000400
H	1.45465600	3.47010000	0.35899600
H	2.32843200	2.61094700	-0.95326200
H	0.74174100	3.39039700	-1.26775700

^{1}B$_{s\text{-}cis}$

SCF Energy: UB3LYP/def2-SVP = -787.398464

SCF Energy: D3(BJ)-UB3LYP/def2-TZVPP = -788.339382

SCF Energy: D3(BJ)-UB3LYP/def2-TZVPP (Toluene correction) = -788.343965

Zero-point correction = 0.294706
Thermal correction to Gibbs Free Energy = 0.247865

<S*S> Value: UB3LYP/def2-SVP = 0.1927

<S*S> Value: D3(BJ)-UB3LYP/def2-TZVPP = 0.1754

<S*S> Value: D3(BJ)-UB3LYP/def2-TZVPP (Toluene correction) = 0.1612

C	-3.82487800	0.37627100	-0.93344200
C	-2.60575800	-0.28113200	-0.79837100
C	-1.93389500	-0.32474400	0.45684200
C	-2.55125100	0.34793800	1.55095700
C	-3.76817400	1.00036600	1.40446100
C	-4.42049400	1.01589700	0.16200600
H	-4.31618500	0.39987200	-1.90986300
H	-2.05044700	0.34258500	2.52290600
H	-4.21795900	1.50521300	2.26340300
H	-5.37848500	1.52848400	0.04764400
C	-0.67708700	-0.96215000	0.67978000
H	-0.14629300	-0.73652800	1.60574900
H	-2.15289300	-0.74479400	-1.67524300
C	2.16134300	-0.82117800	0.41815200
C	1.38092100	-1.95146400	-0.12919900
O	1.91681000	-2.99179400	-0.49843600
C	3.35219400	-1.21133300	1.24981100
H	4.29039500	-0.79370800	0.84539800
H	3.26158300	-0.83474400	2.28580100
H	3.45164300	-2.30323900	1.28700200
C	1.98295400	0.56076600	0.01171500
C	2.65264300	1.60568200	0.70362200
C	1.20799300	0.93598500	-1.12075100
C	2.52989200	2.93613900	0.30801700
H	3.26483800	1.36958800	1.57527300
C	1.09506600	2.26426500	-1.51412400
H	0.70595800	0.16384400	-1.70522600
C	1.75021800	3.27863300	-0.80166600
H	3.05074000	3.71480400	0.87166200
H	0.49576400	2.51459700	-2.39338300
H	1.65954200	4.32156500	-1.11463300
N	-0.02171100	-1.81105100	-0.19056100
C	-0.73218100	-2.84870400	-0.94475000
H	-0.71145900	-2.64802100	-2.02807800

H	-0.21554800	-3.80368600	-0.77351700
H	-1.77072500	-2.91153700	-0.60183700

***cis*-TS$_{B4a}$**

SCF Energy: UB3LYP/def2-SVP = -787.396798

SCF Energy: D3(BJ)-UB3LYP/def2-TZVPP = -788.339424

SCF Energy: D3(BJ)-UB3LYP/def2-TZVPP (Toluene correction) = -788.344405

Zero-point correction = 0.293978

Thermal correction to Gibbs Free Energy = 0.247804

<S*S> Value: UB3LYP/def2-SVP = 0.1046

<S*S> Value: D3(BJ)-UB3LYP/def2-TZVPP = 0.0922

<S*S> Value: D3(BJ)-UB3LYP/def2-TZVPP (Toluene correction) = 0.0869

Imaginary Frequency (cm^{-1}) = -90.21

C	-3.37985500	0.59476400	-1.00812000
C	-2.21848000	-0.15479400	-0.84440400
C	-1.77965200	-0.54279300	0.45101600
C	-2.55751900	-0.12332000	1.56563700
C	-3.71214200	0.62918700	1.39200800
C	-4.13531800	0.99040800	0.10358100
H	-3.69860600	0.88333100	-2.01319900
H	-2.23395600	-0.40382100	2.57180800
H	-4.29354200	0.93991000	2.26396500
H	-5.04504600	1.58043600	-0.03118900
C	-0.59320000	-1.30530900	0.66990800
H	-0.29045900	-1.51290600	1.69931900
H	-1.62861100	-0.43882400	-1.71714900
C	1.93162100	-0.68157600	0.58287600
C	1.50347000	-1.85941400	-0.22705100
O	2.26954400	-2.69381800	-0.68391000
C	2.88426400	-0.97237600	1.70999500
H	3.86960500	-0.50077300	1.54651500
H	2.50413600	-0.58098100	2.67072000
H	3.04307400	-2.05362700	1.81950300
C	1.74601600	0.67463200	0.10739400
C	2.11899000	1.78441300	0.91258700
C	1.23823400	0.96105300	-1.18951700
C	1.96674100	3.09209100	0.45964200
H	2.51988900	1.61533900	1.91330000
C	1.09321800	2.27086500	-1.63662100
H	0.98409900	0.13748400	-1.85857300
C	1.45161800	3.34833000	-0.81681200
H	2.25513700	3.92315900	1.10890700
H	0.70710200	2.45561500	-2.64258400
H	1.33960700	4.37536000	-1.17253000
N	0.12515900	-1.93518900	-0.35446200

C	-0.44359200	-2.97172100	-1.21519900
H	-0.19376100	-2.78501600	-2.27110500
H	-0.02432900	-3.95409000	-0.94222500
H	-1.53329100	-2.99037400	-1.09448600

C$_{s\text{-}trans}$

SCF Energy: UB3LYP/def2-SVP = -787.401046

SCF Energy: D3(BJ)-UB3LYP/def2-TZVPP = -788.341780

SCF Energy: D3(BJ)-UB3LYP/def2-TZVPP (Toluene correction) = -788.350514

Zero-point correction = 0.297093

Thermal correction to Gibbs Free Energy = 0.250957

<S*S> Value: UB3LYP/def2-SVP = 0.0000

<S*S> Value: D3(BJ)-UB3LYP/def2-TZVPP = 0.0000

<S*S> Value: D3(BJ)-UB3LYP/def2-TZVPP (Toluene correction) = 0.0000

C	5.15345600	0.10725200	0.69320700
C	3.80376800	0.45245800	0.67658700
C	2.91034000	-0.18655600	-0.21691500
C	3.42563500	-1.19617800	-1.06633300
C	4.77667500	-1.52921300	-1.05137500
C	5.64802800	-0.87500500	-0.17285400
H	5.82679100	0.60484000	1.39520100
H	2.74670700	-1.71455400	-1.74833300
H	5.15278900	-2.30498200	-1.72231500
H	6.70861800	-1.13707200	-0.15508300
C	1.48398900	0.07229100	-0.32491200
H	0.90444000	-0.68663400	-0.85780800
H	3.44343200	1.19803300	1.38273700
C	-1.40827000	-0.07378500	0.37939200
C	-0.74682600	1.07232500	-0.10638400
O	-1.15652300	2.10441300	-0.64512200
C	-0.75083900	-1.11970800	1.25842900
H	-1.35387900	-1.28513300	2.16784600
H	-0.65601300	-2.11019000	0.77376400
H	0.25115300	-0.83308700	1.60282300
C	-2.84882800	-0.23719400	0.13565100
C	-3.52367300	-1.40648400	0.58137500
C	-3.64175500	0.71916300	-0.55777500
C	-4.88531800	-1.60532800	0.35507900
H	-2.97574600	-2.18856600	1.10694200
C	-5.00249000	0.51383200	-0.77546700
H	-3.16308600	1.62806100	-0.91431700
C	-5.64179800	-0.64621300	-0.32375500
H	-5.35861400	-2.52406800	0.71373700
H	-5.57490900	1.27783000	-1.30987900
H	-6.70954700	-0.80078500	-0.49982400
N	0.76942500	1.07874500	0.10484500
C	1.30767300	2.35017900	0.58284600
H	1.47057700	2.31476600	1.67183600

H	2.24735100	2.59897400	0.07314600
H	0.54754300	3.10504100	0.34581900

***trans*-TS$_{C4a}$**

SCF Energy: UB3LYP/def2-SVP = -787.395453

SCF Energy: D3(BJ)-UB3LYP/def2-TZVPP = -788.336050

SCF Energy: D3(BJ)-UB3LYP/def2-TZVPP (Toluene correction) = -788.341503

Zero-point correction = 0.296333
Thermal correction to Gibbs Free Energy = 0.252118

<S*S> Value: UB3LYP/def2-SVP = 0.0000

<S*S> Value: D3(BJ)-UB3LYP/def2-TZVPP = 0.0000
<S*S> Value: D3(BJ)-UB3LYP/def2-TZVPP (Toluene correction) = 0.0000

Imaginary Frequency (cm^{-1}) = -75.50

C	4.63884700	-0.31037300	0.76059900
C	3.38041500	0.26369700	0.60297700
C	2.48263500	-0.21846800	-0.38427300
C	2.89952700	-1.31526400	-1.18161000
C	4.15689600	-1.88632600	-1.01544300
C	5.03668200	-1.38356400	-0.04744500
H	5.31551600	0.07369900	1.52819400
H	2.21644900	-1.70877300	-1.93915300
H	4.45787000	-2.72916600	-1.64264200
H	6.02484400	-1.83141000	0.08238800
C	1.16792000	0.32625200	-0.59373100
H	0.50581100	-0.20390700	-1.27836700
H	3.07597300	1.08014900	1.25975800
C	-1.15629000	0.23375900	0.59891100
C	-0.68145400	1.53939000	0.13813000
O	-1.33902500	2.55172100	-0.09473600
C	-0.43212800	-0.47627100	1.72502500
H	-1.03204800	-0.41811000	2.65329900
H	-0.26532400	-1.54862300	1.52828500
H	0.54229100	-0.02171900	1.94051800
C	-2.49801500	-0.21564300	0.26150400
C	-3.01913300	-1.42098600	0.80728900
C	-3.32558300	0.47797400	-0.66719600
C	-4.26950400	-1.91006300	0.43562200
H	-2.43122100	-1.99114900	1.52677800
C	-4.57218400	-0.01842300	-1.03288300
H	-2.97867300	1.42797200	-1.07045900
C	-5.05693200	-1.21485000	-0.48788800
H	-4.63461800	-2.84284900	0.87396200
H	-5.18227800	0.54128200	-1.74711000
H	-6.04053100	-1.59587800	-0.77418700
N	0.72065700	1.51088000	-0.08794400

C	1.45358900	2.77377100	-0.11646500
H	1.69476000	3.12645100	0.89941100
H	2.37609900	2.66690800	-0.69975500
H	0.79096300	3.51753100	-0.58616500

C*s-cis*

SCF Energy: UB3LYP/def2-SVP = -787.398805

SCF Energy: D3(BJ)-UB3LYP/def2-TZVPP = -788.343037

SCF Energy: D3(BJ)-UB3LYP/def2-TZVPP (Toluene correction) = -788.351518

Zero-point correction = 0.295920

Thermal correction to Gibbs Free Energy = 0.249182

<S*S> Value: UB3LYP/def2-SVP = 0.0000

<S*S> Value: D3(BJ)-UB3LYP/def2-TZVPP = 0.0000

<S*S> Value: D3(BJ)-UB3LYP/def2-TZVPP (Toluene correction) = 0.0000

C	4.10905600	0.31492700	0.84981500
C	2.82079500	-0.21697400	0.85689300
C	2.04935900	-0.23638300	-0.32754100
C	2.60677100	0.32131900	-1.50138000
C	3.89955200	0.83783400	-1.50624500
C	4.65638500	0.83284500	-0.32935500
H	4.68958000	0.33257900	1.77515100
H	2.01073800	0.33917700	-2.41728900
H	4.31614800	1.25415400	-2.42622100
H	5.66865400	1.24442100	-0.32727700
C	0.68848100	-0.74511300	-0.44459800
H	0.11696500	-0.38823000	-1.30697700
H	2.40737700	-0.58400200	1.79438100
C	-2.30387100	-0.93371700	-0.33809900
C	-1.41876900	-1.98416100	-0.09587900
O	-1.53822200	-3.20720200	-0.17404800
C	-3.61539700	-1.34545200	-0.97261700
H	-4.46902900	-0.80493700	-0.53025000
H	-3.64275400	-1.15056200	-2.06280300
H	-3.77243100	-2.42520000	-0.83874700
C	-2.11994100	0.48381700	0.01252400
C	-2.65880300	1.50074500	-0.81399500
C	-1.48780700	0.91635000	1.20590600
C	-2.52883300	2.85392200	-0.49637800
H	-3.17740600	1.21933000	-1.73330300
C	-1.34635700	2.26833200	1.51659000
H	-1.13376700	0.16909400	1.91996900
C	-1.86070600	3.25343900	0.66516900
H	-2.95355000	3.60596200	-1.16783000
H	-0.85557000	2.55694500	2.45093000
H	-1.76149800	4.31260600	0.91530500
N	0.02836000	-1.56114400	0.31805500
C	0.59491700	-2.35544000	1.40390100
H	0.52629600	-1.80590700	2.35612200

H	-0.01253400	-3.26822100	1.45503200
H	1.64104400	-2.61931200	1.20154300

***cis*-TS$_{C4a}$**

SCF Energy: UB3LYP/def2-SVP = -787.393038

SCF Energy: D3(BJ)-UB3LYP/def2-TZVPP = -788.336983

SCF Energy: D3(BJ)-UB3LYP/def2-TZVPP (Toluene correction) = -788.342841

Zero-point correction = 0.295297

Thermal correction to Gibbs Free Energy = 0.250496

<S*S> Value: UB3LYP/def2-SVP = 0.0000

<S*S> Value: D3(BJ)-UB3LYP/def2-TZVPP = 0.0000

<S*S> Value: D3(BJ)-UB3LYP/def2-TZVPP (Toluene correction) = 0.0000

Imaginary Frequency (cm^{-1}) = -88.01

C	-3.46051600	0.35502500	-0.94093200
C	-2.21343600	-0.25556700	-0.83234100
C	-1.71966400	-0.66653100	0.43145700
C	-2.51821200	-0.41314800	1.57593200
C	-3.76096300	0.19852600	1.45974800
C	-4.24118300	0.58089700	0.19908900
H	-3.82512500	0.66923900	-1.92209500
H	-2.14355900	-0.70855700	2.55964000
H	-4.36280000	0.38289800	2.35303200
H	-5.21831700	1.06147500	0.10767500
C	-0.43642700	-1.28800500	0.60873700
H	-0.07377500	-1.41394000	1.62843700
H	-1.60272200	-0.39747500	-1.72405900
C	2.08779200	-0.54272700	0.56071600
C	1.72103200	-1.77495500	-0.12463900
O	2.45623800	-2.72128100	-0.38942900
C	3.25182400	-0.64458500	1.50893000
H	4.12201400	-0.05743400	1.16311300
H	2.99110300	-0.25051800	2.50811100
H	3.56521400	-1.69085200	1.62035500
C	1.67431000	0.78223500	0.10001300
C	1.84432500	1.92122200	0.92894700
C	1.18053600	1.01739800	-1.21082000
C	1.49690800	3.19826000	0.49427700
H	2.23619300	1.79647600	1.94013600
C	0.83234900	2.29774600	-1.63924700
H	1.11732400	0.18543500	-1.91416300
C	0.98203900	3.40018000	-0.79166700
H	1.63090300	4.04902000	1.16824700
H	0.46407100	2.43952400	-2.65924000
H	0.71944500	4.40402100	-1.13395600
N	0.32044900	-1.81570000	-0.39074900

C	-0.16886700	-2.69751000	-1.44662700
H	-0.05688800	-2.23952900	-2.44280800
H	0.45377500	-3.60580700	-1.41619600
H	-1.22099200	-2.95459400	-1.27498100

Minimum Energy Crossing Point (MECP) Calculations

To generate structures and energies for the minimum energy crossing point (MECP) between triplet diradical **^{3}B** and both singlet diradical **^{1}B*s-trans*** and zwitterion **C*s-trans***, the MECP optimisation routine was used as implemented in Orca 5.0.2.[9,10] The energies of the neighbouring states (namely **^{3}B**, **^{1}B*s-trans*** and **C*s-trans***) were also reoptimized to compare the energies with the Gaussian calculations, which were indeed comparable.

Geometry optimisations were performed, using the geometries from the Gaussian calculations, at the UB3LYP/G/def2-SVP level,[2–6] followed by frequency calculations at the same level. All minima were confirmed as such by the absence of imaginary frequencies. Single-point calculations on the UB3LYP/G/def2-SVP optimised geometries were performed at the UB3LYP/G/def2-TZVPP level of theory,[2–6] with dispersion effects modelled with Grimme's D3 method with additional Becke-Johnson damping.[7] The D3(BJ)-B3LYP/def2-TZVPP SCF energies were corrected for their thermal, enthalpic and entropic corrections at 298.15 K (obtained from the B3LYP/G/def2-SVP level frequency calculations). Optimisations were performed with TightSCF convergence criteria, and no symmetry constraints were applied. Where used, solvent corrections were applied with the conductor-like polarizable continuum model (C-PCM) in toluene.[11] In the geometry optimisations and single-point calculations of the singlet diradical species (**^{1}B*s-trans***, **MECP(^{3}B→^{1}B*s-trans*)**), broken symmetry was required to be specified in the input.

Energies in Hartrees, spin expectation (<S*S>) values and xyz coordinates are reported.

^{3}B

SCF Energy: UB3LYP/G/def2-SVP = -787.39765188
SCF Energy: UB3LYP/G/def2-TZVPP = -788.25932253
SCF Energy: UB3LYP/G/def2-TZVPP (Toluene correction) = -788.26704721

Zero-point correction = 0.2948184
Total Thermal correction = 0.31220176
Entropic Correction = -0.06296159

Dispersion Correction = -0.076463568

<S*S> Value: UB3LYP/G/def2-SVP = 2.050144
<S*S> Value: D3(BJ)-UB3LYP/G/def2-TZVPP = 2.048263
<S*S> Value: D3(BJ)-UB3LYP/G/def2-TZVPP (Toluene correction) = 2.047389

C	4.979304	0.071599	0.395463
C	3.657884	0.506167	0.366665
C	2.644220	-0.267448	-0.275909
C	3.051604	-1.505249	-0.862646
C	4.373458	-1.925021	-0.828034
C	5.355208	-1.138552	-0.203734
H	5.731213	0.682663	0.902308
H	2.297914	-2.126596	-1.354348
H	4.649627	-2.875785	-1.291874
H	6.396172	-1.468792	-0.178648
C	1.266190	0.077767	-0.341403
H	0.560543	-0.694745	-0.655244
H	3.397326	1.436147	0.871700
C	-1.360518	0.135944	0.767116
C	-0.680782	1.383247	0.310987
O	-1.261697	2.461186	0.261666
C	-0.714003	-0.664324	1.866847
H	-1.369353	-0.700528	2.755136
H	-0.520670	-1.711629	1.573728
H	0.243877	-0.228652	2.178906
C	-2.662430	-0.210679	0.265322
C	-3.339616	-1.370084	0.745840
C	-3.322573	0.547808	-0.747751
C	-4.582118	-1.744790	0.245851
H	-2.878560	-1.984267	1.520290
C	-4.564715	0.164500	-1.238198
H	-2.851646	1.454887	-1.124308
C	-5.206479	-0.983264	-0.750794
H	-5.071937	-2.640805	0.636211
H	-5.044932	0.769813	-2.011545
H	-6.182728	-1.279326	-1.142444

N	0.680106	1.298263	-0.024101
C	1.356134	2.572738	-0.287043
H	1.720433	3.040295	0.642119
H	2.200042	2.406034	-0.965562
H	0.634654	3.261516	-0.745034

^{1}B$_{s\text{-}trans}$

SCF Energy: UB3LYP/G/def2-SVP = -787.40212783

SCF Energy: UB3LYP/G/def2-TZVPP = -788.26391834

SCF Energy: UB3LYP/G/def2-TZVPP (Toluene correction) = -788.27211547

Zero-point correction = 0.29543122
Total Thermal correction = 0.31254881
Entropic Correction = -0.06124786

Dispersion Correction = -0.076795665

<S*S> Value: UB3LYP/G/def2-SVP = 0.764536

<S*S> Value: D3(BJ)-UB3LYP/G/def2-TZVPP = 0.739049
<S*S> Value: D3(BJ)-UB3LYP/G/def2-TZVPP (Toluene correction) = 0.683657

C	4.901826	-0.007986	0.671346
C	3.590560	0.434200	0.523343
C	2.673535	-0.266999	-0.309857
C	3.148196	-1.441203	-0.960774
C	4.459736	-1.872778	-0.806871
C	5.350853	-1.156887	0.006118
H	5.584045	0.544469	1.322943
H	2.461511	-2.004845	-1.598286
H	4.796335	-2.775489	-1.323290
H	6.382459	-1.495909	0.126030
C	1.306932	0.106931	-0.506726
H	0.640999	-0.624622	-0.968266
H	3.260524	1.313598	1.077158
C	-1.310602	0.118784	0.560809
C	-0.682853	1.354827	0.060864
O	-1.283244	2.405510	-0.152396
C	-0.610741	-0.703975	1.617737
H	-1.209825	-0.714583	2.546146
H	-0.470710	-1.758742	1.323807
H	0.376889	-0.298615	1.868266
C	-2.679744	-0.201624	0.213410
C	-3.310819	-1.352916	0.765839
C	-3.443169	0.561928	-0.718085
C	-4.605399	-1.718391	0.408119
H	-2.774689	-1.976043	1.481895
C	-4.734121	0.186176	-1.071021
H	-3.008602	1.466682	-1.138541
C	-5.329827	-0.953217	-0.512989
H	-5.055395	-2.609758	0.853369
H	-5.290698	0.796093	-1.787688
H	-6.347488	-1.238951	-0.790797

N	0.719500	1.296574	-0.151113
C	1.400771	2.584887	-0.306132
H	1.645992	3.033774	0.669969
H	2.317502	2.454218	-0.891954
H	0.710915	3.264920	-0.824846

MECP(^{3}B→^{1}B$_{s\text{-}trans}$)

SCF Energy: UB3LYP/G/def2-SVP = -787.39576642

SCF Energy: UB3LYP/G/def2-TZVPP = -788.25771245

SCF Energy: UB3LYP/G/def2-TZVPP (Toluene correction) = -788.26584877

Zero-point correction = 0.29273491
Total Thermal correction = 0.30979965
Entropic Correction = -0.06225652

Dispersion Correction = -0.0760754

C	5.032541	0.166289	-0.412720
C	3.760572	0.646869	-0.116643
C	2.614557	-0.207656	-0.171113
C	2.863408	-1.572708	-0.534500
C	4.135772	-2.035329	-0.830249
C	5.241185	-1.170273	-0.776460
H	5.883007	0.851211	-0.352888
H	2.016241	-2.262504	-0.584170
H	4.275229	-3.083857	-1.107195
H	6.244744	-1.533961	-1.008122
C	1.265386	0.122488	0.130917
H	0.555326	-0.705786	0.151397
H	3.663408	1.687789	0.180743
C	-1.434568	0.150541	0.996600
C	-0.721301	1.416141	0.625378
O	-1.322146	2.482193	0.612972
C	-1.014215	-0.525661	2.273480
H	-1.772335	-0.380223	3.064691
H	-0.890703	-1.615417	2.148276
H	-0.063564	-0.125366	2.652244
C	-2.595419	-0.260883	0.262723
C	-3.379303	-1.372978	0.688394
C	-3.008488	0.403664	-0.930405
C	-4.492573	-1.790663	-0.032745
H	-3.107929	-1.910775	1.598106
C	-4.124087	-0.021010	-1.641207
H	-2.448924	1.271134	-1.281082
C	-4.875056	-1.121645	-1.203516
H	-5.072637	-2.647255	0.320646
H	-4.416425	0.511264	-2.550122
H	-5.749829	-1.453296	-1.768022
N	0.651050	1.352332	0.350765
C	1.342926	2.624478	0.165852
H	2.067693	2.805911	0.974786
H	1.869111	2.636984	-0.799013

H	0.588498	3.417997	0.179735

C*s-trans*

SCF Energy: UB3LYP/G/def2-SVP = -787.40183824
SCF Energy: UB3LYP/G/def2-TZVPP = -788.26629521
SCF Energy: UB3LYP/G/def2-TZVPP (Toluene correction) = -788.27987379

Zero-point correction = 0.29704643
Total Thermal correction = 0.31385542
Entropic Correction = -0.06068029

Dispersion Correction = -0.076233667

<S*S> Value: UB3LYP/G/def2-SVP = 0.000000
<S*S> Value: D3(BJ)-UB3LYP/G/def2-TZVPP = 0.000000
<S*S> Value: D3(BJ)-UB3LYP/G/def2-TZVPP (Toluene correction) = 0.000000

C	5.185102	0.202253	0.572834
C	3.833728	0.537916	0.526730
C	2.930882	-0.224178	-0.253883
C	3.439640	-1.343166	-0.958014
C	4.792256	-1.667028	-0.915315
C	5.672358	-0.891971	-0.151400
H	5.865787	0.796877	1.186725
H	2.754020	-1.955072	-1.549841
H	5.162787	-2.529269	-1.474494
H	6.734423	-1.145597	-0.112325
C	1.502454	0.013043	-0.382243
H	0.923524	-0.811727	-0.806923
H	3.480638	1.376553	1.123373
C	-1.395916	-0.045416	0.329755
C	-0.732316	1.028289	-0.296420
O	-1.139156	1.983424	-0.964149
C	-0.741663	-0.972356	1.335529
H	-1.345851	-1.019657	2.257862
H	-0.649045	-2.016551	0.980386
H	0.261012	-0.647443	1.641963
C	-2.836520	-0.236851	0.106115
C	-3.514417	-1.336928	0.699207
C	-3.626638	0.622461	-0.707112
C	-4.876433	-1.560190	0.499701
H	-2.968477	-2.044985	1.322618
C	-4.987760	0.393701	-0.897516
H	-3.145441	1.475864	-1.178832
C	-5.630252	-0.695856	-0.298790
H	-5.352261	-2.423232	0.974576
H	-5.557898	1.082889	-1.527553
H	-6.698329	-0.869418	-0.453929

N	0.784382	1.063316	-0.084121
C	1.318709	2.386343	0.230521
H	1.500082	2.480626	1.313044
H	2.247898	2.579740	-0.321060
H	0.549412	3.103021	-0.082402

MECP(^{3}B→C$_{s\text{-}trans}$)

SCF Energy: UB3LYP/G/def2-SVP = -787.39521890

SCF Energy: UB3LYP/G/def2-TZVPP = -788.25645499

SCF Energy: UB3LYP/G/def2-TZVPP (Toluene correction) = -788.2636193

Zero-point correction = 0.29239586
Total Thermal correction = 0.3094763
Entropic Correction = -0.06231732

Dispersion Correction = -0.076724351

C	4.953679	0.023826	0.618787
C	3.638355	0.461453	0.497236
C	2.698091	-0.261288	-0.291997
C	3.157825	-1.452144	-0.925519
C	4.473681	-1.877865	-0.798618
C	5.386588	-1.140665	-0.029781
H	5.652756	0.593293	1.237038
H	2.454800	-2.033263	-1.528583
H	4.796715	-2.793055	-1.301586
H	6.421936	-1.474941	0.069057
C	1.324587	0.096644	-0.462875
H	0.657368	-0.655974	-0.887862
H	3.325054	1.353262	1.039956
C	-1.331431	0.116234	0.551144
C	-0.693022	1.335433	0.039776
O	-1.284091	2.383066	-0.219430
C	-0.638418	-0.709530	1.609780
H	-1.246526	-0.728542	2.531965
H	-0.489975	-1.762909	1.312912
H	0.344713	-0.301975	1.874239
C	-2.700131	-0.199334	0.203093
C	-3.339151	-1.343055	0.764790
C	-3.459484	0.558370	-0.737891
C	-4.634694	-1.705123	0.408549
H	-2.807870	-1.963192	1.486905
C	-4.751510	0.185475	-1.088865
H	-3.019088	1.455415	-1.168273
C	-5.354387	-0.945325	-0.520906
H	-5.089413	-2.590338	0.861303
H	-5.303456	0.790884	-1.812944
H	-6.372912	-1.228818	-0.797761
N	0.722644	1.280169	-0.135953
C	1.404603	2.571416	-0.254877
H	1.668536	2.983047	0.732852
H	2.310383	2.462082	-0.861937

4. General procedure A: Synthesis of acrylamides (1–3) using Mukaiyama's reagent.

To a mixture of amine (1.1 equiv.), carboxylic acid (1 equiv.) and 2-chloro-1-methylpyridinium iodide (1.2–1.4 equiv.) in CH_2Cl_2 (5 mL/mmol of aniline) at 0 °C under argon, was added triethylamine (3 equiv.) dropwise and the reaction mixture allowed to warm slowly to room temperature. Once the reaction was completed (by TLC) the organic phase was washed with aqueous HCl (1.0 M), followed by sat. aqueous Na_2CO_3 and dried over $MgSO_4$. The organic solvents were removed in vacuo and the resulting crude product was purified by column chromatography on silica gel or neutral alumina (petroleum ether:EtOAc gradient) to give acrylamide **1–3**.

5. General procedure B: Synthesis of acrylamides (1–3) including reductive amination.

To a mixture of aldehyde (1 equiv.), $MgSO_4$ (3 equiv.) in CH_2Cl_2 (5 mL/mmol of aldehyde), was added methylamine 33% in abs. ethanol (3 equiv.) at room temperature and the reaction was stirred overnight. The solvent was evaporated, followed by the addition of $NaBH_4$ (1 equiv.) in MeOH (3.3 mL/mmol of aldehyde) at room temperature and the reaction mixture was stirred for a further 3 hrs, before the addition of water. Excess MeOH was removed under reduced pressure and the product was extracted with 3 x CH_2Cl_2. The combined organic layers were dried over $MgSO_4$ and the organic solvents were removed in vacuo. The resulting crude product was used directly in the subsequent coupling reaction (general procedure A).

6. Characterization data of acrylamides 1.

N-benzyl-*N*-methyl-2-phenylacrylamide (1a)

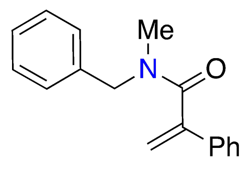

1a

Prepared according to general procedure A using *N*-methyl-1-phenylmethanamine (0.500 g, 4.12 mmol), 2-phenylacrylic acid (0.856 g, 5.77 mmol), 2-chloro-1-methylpyridinium iodide (1.264 g, 4.95 mmol) and triethylamine (1.72 mL, 12.37 mmol) in CH_2Cl_2 (50 mL). Chromatography on silica gel with EtOAc:petroleum ether (1:9 to 3:7) afforded **1a** in a 0.55:0.45 ratio of rotamers as a clear oil (0.685 g, 66% yield). $\mathbf{R_f}$ = 0.15 (2:8 EtOAc:petroleum ether); **IR** (film, ν_{max}/cm^{-1}) 3059, 3029, 2925, 1710, 1684, 1634, 1575, 1492, 1449, 1402, 1253, 1211, 1117, 1075, 1027; ^{1}**H NMR** (300 MHz, $CDCl_3$) 7.50–7.44 (m, 2H), 7.39–7.28 (m, 7H), 7.09–7.06 (m, 1H), 5.77 (s, 0.55 H), 5.73 (s, 0.45H), 5.45 (s, 0.45H), 5.41 (s,

0.55H) 4.73 (s, 1.1H), 4.43 (s, 0.9H), 2.98 (s, 1.35H), 2.80 (s, 1.65H); **^{13}C NMR** (101 MHz, $CDCl_3$) δ 171.4, 171.0, 145.2, 145.0, 137.0, 136.3, 135.7, 135.6, 129.0, 128.9, 128.8, 128.8, 128.7, 128.4, 128.3, 128.1, 127.7, 127.6, 127.1, 125.8, 125.7, 114.4, 114.3, 54.5, 50.3, 36.0, 32.3; **HRMS** (ESI^+) *m/z*: [M + H] calcd for $C_{17}H_{18}NO^+$ 252.1383; found 252.1396.

_N_-methyl-_N_-(4-methylbenzyl)-2-phenylacrylamide (1b)

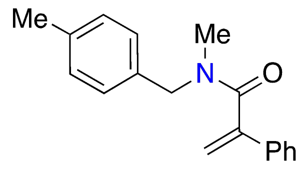

1b

Prepared according to general procedure A using *N*-methyl-1-(*p*-tolyl)methanamine (0.297 g, 2.2 mmol), 2-phenylacrylic acid (0.297 g, 2 mmol), 2-chloro-1-methylpyridinium iodide (0.665 g, 2.6 mmol) and triethylamine (0.836 mL, 6 mmol) in CH_2Cl_2 (25 mL). Chromatography on silica gel with EtOAc:petroleum ether (1:9 to 4:6) afforded **1b** in a 0.55:0.45 ratio of rotamers as a clear oil (0.355 g, 67% yield). **R_f** = 0.26 (3:7 EtOAc:petroleum ether); **IR** (film, ν_{max}/cm^{-1}) 3053, 3024, 2922, 2864, 1712, 1682, 1635, 1574, 1514, 1490, 1445, 1401, 1252, 1210, 1118, 1075, 1025; **^{1}H NMR** (300 MHz, $CDCl_3$) 7.49–7.43 (m, 2H), 7.37–7.33 (m, 3H), 7.24–7.09 (m, 3H), 6.97–6.94 (m, 1H), 5.75 (s, 0.55H), 5.73 (s, 0.45H), 5.44 (s, 0.45H), 5.38 (s, 0.55H), 4.68 (s, 1.1H), 4.39 (s, 0.9H), 2.96 (s, 1.35H), 2.78 (s, 1.65H), 2.35 (s, 1.65H), 2.32 (s, 1.35H); **^{13}C NMR** (101 MHz, $CDCl_3$) δ 171.3, 170.9, 145.3, 145.1, 137.5, 137.3, 135.8, 135.6, 134.0, 133.3, 129.5, 129.4, 129.0, 128.9, 128.8, 128.6, 128.4, 127.1, 125.8, 125.8, 114.3, 114.1, 54.3, 50.0, 35.9, 32.2, 21.2, 21.2; **HRMS** (ESI^+) *m/z*: [M + H] calcd for $C_{18}H_{20}NO^+$ 266.1539; found 266.1548.

_N_-methyl-_N_-(2-methylbenzyl)-2-phenylacrylamide (1c)

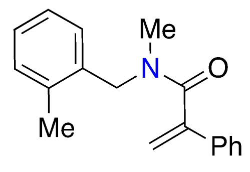

1c

Prepared according to general procedure A using *N*-methyl-1-(*o*-tolyl)methanamine (0.297 g, 2.2 mmol), 2-phenylacrylic acid (0.297 g, 2 mmol), 2-chloro-1-methylpyridinium iodide (0.665 g, 2.6 mmol) and triethylamine (0.836 mL, 6 mmol) in CH_2Cl_2 (25 mL). Chromatography on silica gel with EtOAc:petroleum ether (1:9 to 4:6) afforded **1c** in a 0.58:0.42 ratio of rotamers as a clear oil (0.393 g, 74% yield). **R_f** = 0.33 (3:7 EtOAc:petroleum ether); **IR** (film, ν_{max}/cm^{-1}) 3057, 3023, 2923, 1636, 1548, 1490, 1453, 1400, 1307, 1284, 1245, 1217, 1125, 1076; **^{1}H NMR** (300 MHz, $CDCl_3$) δ 7.51–7.45 (m, 2H), 7.41–7.32 (m, 3H), 7.20–7.06 (m, 4H), 5.77 (s, 0.58H), 5.64 (s, 0.42H), 5.41 (s, 0.58H), 5.38 (s, 0.42H), 4.76 (s, 1.16H), 4.40 (s, 0.84H), 3.02 (s, 1.26H), 2.77 (s, 1.74H), 2.36 (s, 1.74H), 2.14 (s, 1.26H);

^{13}C NMR (101 MHz, $CDCl_3$) δ 171.6, 170.8, 145.5, 144.8, 136.7, 135.7, 135.7, 135.6, 134.5, 134.3, 130.6, 130.6, 128.9, 128.7, 128.3, 127.6, 127.4, 126.3, 126.2, 125.9, 125.8, 114.3, 114.3, 114.2, 52.3, 48.0, 35.8, 32.8, 19.3, 19.1; **HRMS** (ESI$^+$) *m/z*: [M + H] calcd for $C_{18}H_{20}NO^+$ 266.1539; found 266.1551

_N_-methyl-2-phenyl-_N_-(3-(trifluoromethyl)benzyl)acrylamide (1d)

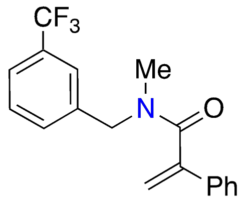

1d

Prepared according to general procedure A using *N*-methyl-1-(3-(trifluoromethyl)phenyl)methanamine (0.416 g, 2.2 mmol), 2-phenylacrylic acid (0.297 g, 2 mmol), 2-chloro-1-methylpyridinium iodide (0.665 g, 2.6 mmol) and triethylamine (0.836 mL, 6 mmol) in CH_2Cl_2 (25 mL). Chromatography on silica gel with EtOAc:petroleum ether (1:9 to 4:6) afforded **1d** in a 0.6:0.4 ratio of rotamers as a clear oil (0.491 g, 77% yield). **R_f** = 0.23 (3:7 EtOAc:petroleum ether; **IR** (film, $ν_{max}$/cm^{-1}) 2926, 1658, 1679, 1612, 1519, 1425, 1364, 1322, 1271, 1165, 1115, 1069, 1017; **^{1}H NMR** (300 MHz, $CDCl_3$) δ 7.57–7.23 (m, 9H), 5.78 (s, 0.6H), 5.73 (s, 0.4H), 5.43 (s, 0.4H), 5.41 (s, 0.6H), 4.77 (s, 1.2H), 4.48 (s, 0.8H), 2.99 (s, 1.2H), 2.81 (s, 1.8H); **^{13}C NMR** (151 MHz, $CDCl_3$) δ 171.4, 171.2, 145.2, 138.3, 137.6, 135.6, 135.6, 131.6, 131.3 (q, J_{C-F} = 32.0 Hz), 131.2 (q, J_{C-F} = 32.2 Hz), 130.4, 129.3, 129.1, 129.0, 129.0, 128.8, 125.9, 125.8, 124.7 (q, J_{C-F} = 3.9 Hz), 124.7, 124.6, 124.5 (q, J_{C-F} = 3.9 Hz), 124.2 (q, J_{C-F} = 272.7 Hz), 124.0 (q, J_{C-F} = 272.1 Hz), 123.9, 123.9, 114.6, 114.5, 54.2, 50.0, 36.2, 32.5; **^{19}F NMR** (377 MHz, $CDCl_3$) *δ* -62.62; **HRMS** (ESI$^+$) *m/z*: [M + H] calcd for $C_{18}H_{17}F_3NO^+$ 320.1257; found 320.1274.

_N_-(4-bromobenzyl)-_N_-methyl-2-phenylacrylamide (1e)

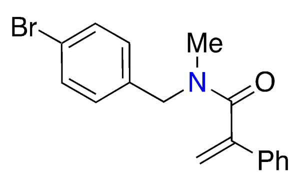

1e

Prepared according to general procedure A using 1-(4-bromophenyl)-*N*-methylmethanamine (0.440 g, 2.2 mmol), 2-phenylacrylic acid (0.297 g, 2 mmol), 2-chloro-1-methylpyridinium iodide (0.665 g, 2.6 mmol) and triethylamine (0.836 mL, 6 mmol) in CH_2Cl_2 (25 mL). Chromatography on silica gel with EtOAc:petroleum ether (1:9 to 3:7) afforded **1e** in a 0.6:0.4 ratio of rotamers as a clear oil (0.495 g, 75% yield). **R_f** = 0.14 (2:8 EtOAc:petroleum ether); **IR** (film, $ν_{max}$/cm^{-1}) 3056, 3028, 2925, 1698, 1634, 1486, 1441, 1399, 1282, 1253, 1209, 1120, 1071, 1010; **^{1}H NMR** (300 MHz, $CDCl_3$) δ 7.47–7.29 (m, 7H), 7.21–7.18 (m, 1.2H), 6.91– 6.89 (m, 0.8H), 5.75 (s, 0.6H),

5.70 (s, 0.4H), 5.39 (s, 0.4H), 5.37 (s, 0.6H), 4.64 (s, 1.2H), 4.36 (s, 0.8H), 2.94 (s, 1.2H), 2.77 (s, 1.8H); **^{13}C NMR** (101 MHz, $CDCl_3$) δ 171.1, 170.9, 144.9, 144.9, 136.0, 135.5, 135.4, 135.3, 131.8, 131.7, 129.9, 128.9, 128.8, 128.7, 128.7, 128.6, 125.7, 125.6, 121.4, 121.4, 114.3, 114.3, 53.8, 49.6, 35.9, 32.2; **HRMS** (ESI^+) *m/z*: [M + H] calcd for $C_{17}H_{17}{}^{79}BrNO^+$ 330.0488; found 330.0484.

***N*-(3-chlorobenzyl)-*N*-methyl-2-phenylacrylamide (1f)**

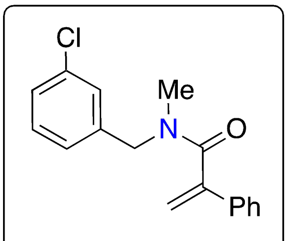

1f

Prepared according to general procedure A using 1-(3-chlorophenyl)-*N*-methylmethanamine (0.342 g, 2.2 mmol), 2-phenylacrylic acid (0.297 g, 2 mmol), 2-chloro-1-methylpyridinium iodide (0.665 g, 2.6 mmol) and triethylamine (0.836 mL, 6 mmol) in CH_2Cl_2 (25 mL). Chromatography on silica gel with EtOAc:petroleum ether (1:9 to 3:7) afforded **1f** in a 0.6:0.4 ratio of rotamers as a clear oil (0.335 g, 59% yield). **R_f** = 0.19 (2:8 EtOAc:petroleum ether); **IR** (film, ν_{max}/cm^{-1}) 3058, 3027, 2926, 1638, 1565, 1568, 1478, 1429, 1400, 1357, 1287, 1253, 1205, 1122, 1078, 1028; **^{1}H NMR** (300 MHz, $CDCl_3$) δ 7.47–7.19 (m, 8.2H), 7.00–6.93 (m, 0.8H), 5.77 (s, 0.6H), 5.72 (s, 0.4H), 5.42 (s, 0.4H), 5.40 (s, 0.6H), 4.68 (s, 1.2H), 4.39 (s, 0.8H), 2.97 (s, 1.2H), 2.79 (s, 1.8H); **^{13}C NMR** (101 MHz, $CDCl_3$) δ 171.3, 171.1, 145.1, 145.0, 139.1, 138.5, 135.6, 135.5, 134.8, 134.6, 130.1 (2C), 129.0, 129.0, 128.9, 128.8, 128.2, 127.9, 127.8, 127.2, 126.4, 125.8, 125.8, 125.2, 114.6, 114.5, 54.0, 49.8, 36.2, 32.5; **HRMS** (ESI^+) *m/z*: [M + H] calcd for $C_{17}H_{17}{}^{35}ClNO^+$ 286.0993; found 286.1004.

***N*-(4-methoxybenzyl)-*N*-methyl-2-phenylacrylamide (1g)**

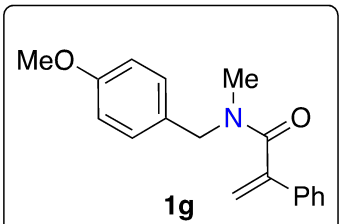

1g

Prepared according to general procedure A using 1-(4-methoxyphenyl)-*N*-methylmethanamine (201 mg, 1.33 mmol), 2-phenylacrylic acid (179 mg, 1.21 mmol), 2-chloro-1-methylpyridinium iodide (402 mg, 1.57 mmol) and triethylamine (0.51 mL, 3.63 mmol) in CH_2Cl_2 (7 mL). Chromatography on silica gel with EtOAc:petroleum ether (1:9 to 1:4) afforded **1g** in a 0.55:0.45 ratio of rotamers as a clear oil (276 mg, 81% yield). **R_f** = 0.38 (3:7 EtOAc:petroleum ether); **IR** (film, ν_{max}/cm^{-1}) 2922, 2851, 1632, 1511, 1443, 1400, 1299, 1245, 1176, 1119, 1076, 1031; **^{1}H NMR** (300 MHz, $CDCl_3$) δ 7.49–7.25 (m, 6H), 6.99–6.80 (m, 3H), 5.75 (s, 1H), 5.44 (s, 0.45H), 5.37 (s, 0.55H), 4.65 (s, 1.1H), 4.36 (s, 0.9H), 3.81 (s, 1.65H), 3.78 (s, 1.35H), 2.94 (s, 1.35H), 2.77 (s, 1.65H); **^{13}C NMR**

(101 MHz, $CDCl_3$) 171.2, 170.9, 159.2, 159.2, 145.2, 145.2, 135.8, 135.6, 129.8, 129.1, 129.0, 128.9, 128.8, 128.7, 128.6, 128.2, 125.9, 125.8, 114.5, 114.2, 114.1, 55.4, 55.4, 54.0, 49.7, 35.8, 35.8, 32.1; **HRMS** (ESI^+) *m/z*: [M + H] calcd for $C_{18}H_{20}NO_2^+$ 282.1498; found 282.1495.

_N_-(4-cyanobenzyl)-_N_-methyl-2-phenylacrylamide (1h)

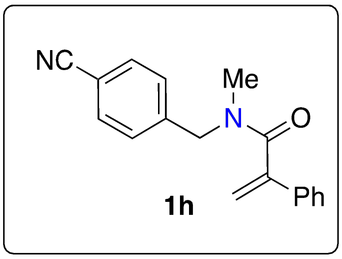

Prepared according to general procedure A using 4-((methylamino)methyl)benzonitrile (322 mg, 2,20 mmol), 2-phenylacrylic acid (296 mg, 2.00 mmol), 2-chloro-1-methylpyridinium iodide (664 mg, 2.60 mmol) and triethylamine (0.84 mL, 6.00 mmol) in CH_2Cl_2 (10 mL). Chromatography on silica gel with EtOAc:petroleum ether (1:9 to 1:4) afforded **1h** in a 0.65:0.35 ratio of rotamers as a clear oil (426 mg, 77% yield). $\mathbf{R_f}$ = 0.48 (6:4 EtOAc:petroleum ether); **IR** (film, ν_{max}/cm^{-1}) 3010, 2929, 2228, 1633, 1493, 1446, 1401, 1214, 1123, 1024; **^{1}H NMR** (300 MHz, $CDCl_3$) δ 7.65 (d, *J* = 8.0 Hz, 1.3H), 7.58 (d, *J* = 8.0 Hz, 0.7H), 7.44–7.35 (m, 6.30H), 7.15 (d, *J* = 8.0 Hz, 0.7H), 5.78 (s, 0.65H), 5.71 (s, 0.35H), 5.41 (s, 0.65H), 5.40 (s, 0.35H), 4.75 (s, 1.30H), 4.48 (s, 0.65H), 2.99 (s, 1.05H), 2.83 (s, 1.95H); **^{13}C NMR** (101 MHz, $CDCl_3$) δ 171.4, 171.3, 145.0, 142.7, 142.1, 135.6, 132.7, 129.1, 129.1, 128.9, 128.8, 127.7, 125.9, 125.8, 118.8, 118.5, 114.8, 111.8, 111.7, 54.3, 50.3, 36.5, 32.8; **HRMS** (ESI^+) *m/z*: [M + H] calcd for $C_{18}H_{17}N_2O^+$ 277.1335; found 277.1353.

_N_-methyl-2-phenyl-_N_-(2-(4,4,5,5-tetramethyl-1,3,2-dioxaborolan-2-yl)benzyl)acrylamide (1i)

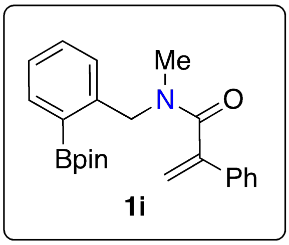

Prepared according to general procedure A using *N*-methyl-1 (2-(4,4,5,5-tetramethyl-1,3,2-dioxaborolan-2-yl)phenyl)methanamine (408 mg, 1.65 mmol), 2-phenylacrylic acid (222 mg, 1.50 mmol), 2-chloro-1-methylpyridinium iodide (498 mg, 1.95 mmol) and triethylamine (0.63 mL, 4.50 mmol) in CH_2Cl_2 (8 mL). Chromatography on silica gel with EtOAc:petroleum ether (1:9 to 1:4) afforded **1i** in a 0.55:0.45 ratio of rotamers as a clear oil (385 mg, 68% yield). $\mathbf{R_f}$ = 0.4 (3:7 EtOAc:petroleum ether); **IR** (film, ν_{max}/cm^{-1}) 2981, 2933, 1634, 1492, 1444, 1378, 1345, 1317, 1265, 1216, 1143, 1114, 1068, 1042; **^{1}H NMR** (300 MHz, $CDCl_3$) δ 7.82 (dd, *J* = 13.9, 7.6 Hz, 1H), 7.50–7.21 (m, 7.55H), 7.09 (d, *J* = 7.6 Hz, 0.45H), 5.76 (s, 0.55H), 5.60 (s, 0.45H), 5.41 (s, 0.55H), 5.33 (s,

0.45H), 5.09 (s, 1.1H), 4.75 (s, 0.9H), 3.04 (s, 1.35H), 2.81 (s, 1.65H), 1.34 (s, 6.6H), 1.25 (s, 5.4H); **^{13}C NMR** (101 MHz, $CDCl_3$) δ 171.7, 171.1, 145.7, 145.2, 143.8, 143.5, 136.5, 136.3, 136.2, 135.9, 131.7, 131.5, 128.9, 128.8, 128.6, 128.6, 127.7, 126.6, 126.5, 126.1, 126.0, 125.7, 114.4, 114.0, 83.9, 83.9, 53.7, 48.7, 36.1, 33.1, 25.1, 24.9; **HRMS** (ESI$^+$) *m/z*: [M + H] calcd for $C_{23}H_{29}BNO_3^+$ 378.2235; found 378.2247.

***N*-(3,5-difluorobenzyl)-*N*-methyl-2-phenylacrylamide (1j)**

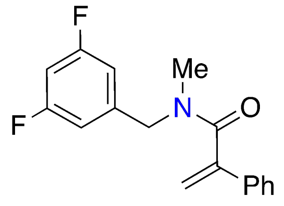

1j

Prepared according to general procedure A using 1-(3,5-difluorophenyl)-*N*-methylmethanamine (0.246 g, 1.57 mmol), 2-phenylacrylic acid (0.212 g, 1.43 mmol), 2-chloro-1-methylpyridinium iodide (0.475, 1.85 mmol) and triethylamine (0.6 mL, 4.29 mmol) in CH_2Cl_2 (15 mL). Chromatography on silica gel with EtOAc:petroleum ether (1:9 to 3:7) afforded **1j** in a 0.6:0.4 ratio of rotamers as a yellow oil (0.270 g, 66% yield). **R_f** = 0.20 (3:7 EtOAc:petroleum ether); **IR** (film, $ν_{max}$/cm^{-1}) 3059, 2926, 1627, 1599, 1550, 1492, 1453, 1340, 1354, 1315, 1202, 1118, 1079; **^{1}H NMR** (300 MHz, $CDCl_3$) δ 7.46–7.33 (m, 5H), 6.85– 6.55 (m, 3H), 5.78 (s, 0.6H), 5.72 (s, 0.4H), 5.42 (s, 1H), 4.68 (s, 1.2H), 4.39 (s, 0.8H), 2.99 (s, 1.2H), 2.82 (s, 1.8H); **^{13}C NMR** (151 MHz, $CDCl_3$) δ 171.4, 171.2, 163.5 (d, $J_{C\text{-}F}$ = 249.8 Hz), 163.4 (d, $J_{C\text{-}F}$ = 249.2 Hz), 145.1, 145.1, 141.2 (t, $J_{C\text{-}F}$ = 8.8Hz), 140.8 (t, $J_{C\text{-}F}$ = 8.2 Hz), 135.6, 135.6, 129.1, 129.1, 129.0, 128.9, 125.9, 125.8, 114.8, 110.9 (dd, $J_{C\text{-}F}$ = 20.6, 5.2 Hz), 109.9 (dd, $J_{C\text{-}F}$ = 20.6, 4.8 Hz), 103.3 (t, $J_{C\text{-}F}$ = 25.1 Hz), 103.2 (t, $J_{C\text{-}F}$ = 25.2 Hz), 54.0, 49.9, 36.4, 32.7; **^{19}F NMR** (377 MHz, $CDCl_3$) *δ* -108.81, -109.30; **HRMS** (ESI$^+$) *m/z*: [M + H] calcd for $C_{17}H_{16}F_2NO^+$ 288.1194; found 288.1203.

***N*-(3-bromo-5-methoxybenzyl)-*N*-methyl-2-phenylacrylamide (1k)**

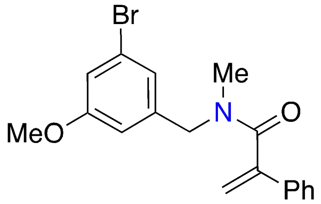

1k

Prepared according to general procedure A using 1-(3-bromo-5-methoxyphenyl)-*N*-methylmethanamine (759 mg, 3.30 mmol), 2-phenylacrylic acid (444 mg, 3.00 mmol), 2-chloro-1-methylpyridinium iodide (996 mg, 3.90 mmol) and triethylamine (1.25 mL, 9.00 mmol) in CH_2Cl_2 (17 mL). Chromatography on silica gel with EtOAc:petroleum ether (1:9 to 1:4) afforded **1k** in a 0.6:0.4 ratio of rotamers as a yellow solid (367 mg, 34% yield). **R_f** = 0.31 (3:7 EtOAc:petroleum ether); **M.P.** 72–75 °C; **IR** (film, $ν_{max}$/cm^{-1}) 3002, 2932, 1633, 1601, 1571, 1456, 1427, 1399, 1268,

1050; **^{1}H NMR** (300 MHz, $CDCl_3$) δ 7.46–7.30 (m, 5H), 7.03 (s, 0.6H), 6.97 (s, 0.6H), 6.92 (s, 0.4H), 6.79 (s, 0.6H), 6.76 (s, 0.4H), 6.48 (s, 0.4H), 5.77 (s, 0.6H), 5.72 (s, 0.4H), 5.42 (s, 0.4H), 5.40 (s, 0.6H), 4.64 (s, 1.2H), 4.36 (s, 0.8H), 3.78 (s, 1.8H), 3.69 (s, 1.2H), 2.97 (s, 1.2H), 2.80 (s, 1.8H); **^{13}C NMR** (101 MHz, $CDCl_3$) δ 171.4, 171.2, 160.8, 160.8, 145.1, 145.0, 140.3, 139.7, 135.7, 135.6, 129.1, 129.0, 128.9, 128.8, 125.9, 125.8, 123.4, 123.3, 123.1, 122.5, 116.6, 116.5, 114.8, 114.6, 112.8, 111.9, 55.6, 55.6, 54.1, 49.9, 36.2, 32.5; **HRMS** (ESI^+) *m/z*: [M + H] calcd for $C_{18}H_{19}{}^{79}BrNO_2^+$ 360.0594; found 360.0601.

_N_-methyl-2-phenyl-_N_-(3,4,5-trichlorobenzyl)acrylamide (1l)

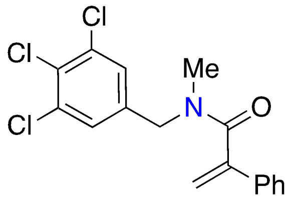

1l

Prepared according to general procedure A using *N*-methyl-1-(3,4,5-trichlorophenyl)methanamine (0.186 g, 0.83 mmol), 2-phenylacrylic acid (0.112 g, 0.753 mmol), 2-chloro-1-methylpyridinium iodide (250 mg, 0.98 mmol) and triethylamine (0.31 mL, 2.26 mmol) in CH_2Cl_2 (10 mL). Chromatography on silica gel with EtOAc:petroleum ether (1:9 to 3:7) afforded **1l** in a 0.65:0.35 ratio of rotamers as a white solid (0.202 g, 91% yield). **R_f** = 0.24 (2:8 EtOAc:petroleum ether); **M.P.** 101–102 °C; **IR** (film, ν_{max}/cm^{-1}) 3059, 2935, 1620, 1554, 1492, 1431, 1397, 1346, 1275, 1250, 1202, 1157, 1115, 1075, 1030; **^{1}H NMR** (300 MHz, $CDCl_3$) δ 7.44–7.34 (m, 6.35H), 6.99 (s, 0.65H), 5.78 (s, 0.65H), 5.72 (s, 0.35H), 5.42 (s, 1H), 4.65 (s, 1.3H), 4.35 (s, 0.7H), 2.98 (s, 1.05H), 2.82 (1.95H); **^{13}C NMR** (151 MHz, $CDCl_3$) δ 171.3, 171.2, 145.1, 145.0, 137.7, 137.0, 135.6, 135.5, 134.7, 134.6, 130.8, 130.7, 129.2, 129.1, 128.9, 128.3, 127.3, 125.9, 125.8, 115.0, 114.9, 53.3, 49.3, 36.4, 32.7; **HRMS** (ESI^+) *m/z*: [M + H] calcd for $C_{17}H_{15}{}^{35}Cl_3NO^+$ 354.0214; found 354.0223.

_N_-methyl-_N_-(naphthalen-1-ylmethyl)-2-phenylacrylamide (1m)

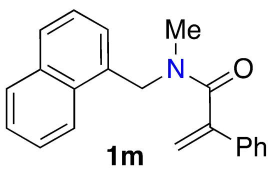

1m

Prepared according to general procedure A using *N*-methyl-1-(naphthalen-1-yl)methanamine (377 mg, 2.20 mmol), 2-phenylacrylic acid (296 mg, 2.00 mmol), 2-chloro-1-methylpyridinium iodide (664 mg, 2.60 mmol) and triethylamine (0.84 mL, 6.00 mmol) in CH_2Cl_2 (11 mL). Chromatography on silica gel with EtOAc:petroleum ether (1:9 to 1:4) afforded **1m** in a 0.7:0.3 ratio of rotamers as a white solid (476 mg, 79%

yield). $\mathbf{R_f}$ = 0.46 (3:7 EtOAc:petroleum ether); **M.P.** 105–107 °C; **IR** (film, ν_{max}/cm^{-1}) 2920, 2864, 1689, 1459, 1377, 1077, 1049; **^{1}H NMR** (300 MHz, $CDCl_3$) δ 8.26 (d, *J* = 8.2 Hz, 0.7H), 7.90–7.30 (m, 11.3H), 5.75 (s, 0.7H), 5.63 (s, 0.3H), 5.42 (s, 0.3H), 5.39 (s, 0.7H), 5.22 (s, 1.4H), 4.92 (s, 0.6H), 3.10 (s, 0.9H), 2.71 (s, 2.1H); **^{13}C NMR** (101 MHz, $CDCl_3$) δ 171.8, 170.7, 145.5, 144.9, 135.8, 135.7, 134.1, 133.8, 132.5, 131.9, 131.8, 131.0, 129.1, 129.0, 128.9, 128.8, 128.8, 128.7, 128.3, 127.7, 126.7, 126.5, 126.2, 126.1, 126.0, 125.9, 125.5, 125.3, 124.2, 124.2, 122.4, 114.3, 114.2, 52.4, 48.3, 35.6, 33.2 (one rotameric signal not reliably assigned due to overlapping signals); **HRMS** (ESI$^+$) *m/z*: [M + H] calcd for $C_{21}H_{20}NO^+$ 302.1539; found 302.1550.

_N_-methyl-2-phenyl-_N_-(1-phenylethyl)acrylamide (1n)

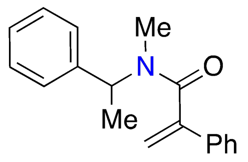

1n

Prepared according to general procedure A using *N*-methyl-1-phenylethan-1-amine (0.297 g, 2.2 mmol), 2-phenylacrylic acid (0.297 g, 2 mmol), 2-chloro-1-methylpyridinium iodide (0.665 g, 2.6 mmol) and triethylamine (0.836 mL, 6 mmol) in CH_2Cl_2 (25 mL). Chromatography on silica gel with EtOAc:petroleum ether (1:9 to 4:6) afforded **1o** in a 0.5:0.5 ratio of rotamers as a clear oil (0.481 g, 59% yield). $\mathbf{R_f}$ = 0.37 (3:7 EtOAc:petroleum ether); **IR** (film, ν_{max}/cm^{-1}) 3057, 3029, 2976, 2936, 1630, 1492, 1445, 1398, 1340, 1124, 1078, 1028; **^{1}H NMR** (300 MHz, $CDCl_3$) δ 7.53–7.51 (m, 1H), 7.46–7.23 (m, 8H), 7.10–7.07 (m, 1H), 6.20 (q, *J* = 6.0 Hz, 0.5H), 5.75 (s, 1H), 5.47 (s, 0.5H), 5.37 (s, 0.5H), 5.12 (q, *J* = 6.0 Hz, 0.5H), 2.75 (s, 1.5H), 2.54 (s, 1.5H), 1.57 (d, *J* = 6.0 Hz, 1.5H), 1.38 (d, *J* = 6.0 Hz, 1.5H); **^{13}C NMR** (151 MHz, $CDCl_3$) δ 170.9, 170.8, 145.8, 145.7, 140.2, 139.7, 135.9, 135.6, 129.1, 128.9, 128.8, 128.7, 128.4, 128.6, 127.5, 127.4, 127.4, 126.8, 126.0, 125.7, 113.9, 113.7, 56.0, 50.2, 30.6, 27.1, 16.8, 15.5; **HRMS** (ESI$^+$) *m/z*: [M + H] calcd for $C_{18}H_{20}NO^+$ 266.1539; found 266.1547.

_N_-methyl-_N_-((1-methyl-1H-indol-3-yl)methyl)-2-phenylacrylamide (1o)

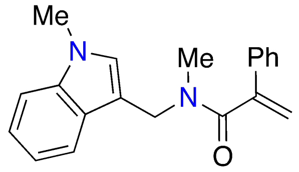

1o

Prepared according to general procedure A using *N*-methyl-1-(1-methyl-1*H*-indol-3-yl)methanamine (0.061 g, 0.35 mmol), 2-phenylacrylic acid (0.47 g, 0.32 mmol), 2-chloro-1-methylpyridinium iodide (0.106 g, 0.41 mmol) and triethylamine (0.133 mL, 0.95 mmol) in CH_2Cl_2 (10 mL). Chromatography on silica gel with EtOAc:petroleum ether (1:9 to 3:7) afforded **1o** in a 0.65:0.35 ratio of rotamers

as a clear oil (0.057 g, 59% yield). **R$_f$** = 0.14 (3:7 EtOAc:petroleum ether); **IR** (film, ν_{max}/cm^{-1}) 3055, 2923, 1681, 1625, 1552, 1475, 1445, 1401, 1377, 1331, 1253, 1125, 1071; **^{1}H NMR** (300 MHz, $CDCl_3$) δ 7.82–7.79 (m, 0.65H), 7.53–6.62 (m, 9H), 6.62 (s, 0.35H), 5.78 (s, 0.35H), 5.72 (s, 0.65H), 5.52 (s, 0.35H), 5.33 (s, 0.65H), 4.87 (s, 1.3H), 4.59 (s, 0.7H), 3.78 (s, 1.95H), 3.70 (s, 1.05), 3.02 (s, 1.05H), 2.80 (s, 1.95H); **^{13}C NMR** (101 MHz, $CDCl_3$) δ 168.0, 168.0, 144.7, 144.7, 137.3, 137.3, 136.1, 136.1, 128.9, 128.9, 128.7, 128.7, 128.4, 128.4, 125.7, 125.7, 122.7, 122.7, 120.9, 120.9, 119.0, 119.0, 117.4, 117.4, 109.0, 109.0, 107.1, 107.1, 34.0, 29.8, 26.8, 26.3, 20.6, 20.6; **HRMS** (ESI$^+$) *m/z*: [M + H] calcd for $C_{20}H_{21}N_2O^+$ 305.1648; found 305.1646.

_N_-(furan-3-ylmethyl)-_N_-methyl-2-phenylacrylamide (1p)

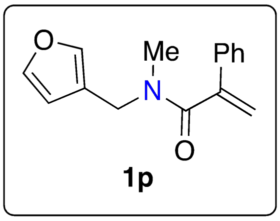

Prepared according to general procedure A using 1-(furan-3-yl)-*N*-methylmethanamine (0.556 g, 5 mmol), 2-phenylacrylic acid (0.674 g, 4.54 mmol), 2-chloro-1-methylpyridinium iodide (1.507 g, 5.90 mmol) and triethylamine (1.89 mL, 13.62 mmol) in CH_2Cl_2 (20 mL). Chromatography on silica gel with EtOAc:petroleum ether (1:9 to 4:6) afforded **1p** in a 0.55:0.45 ratio of rotamers as a clear oil (0.457 g, 42% yield). **R$_f$** = 0.23 (3:7 EtOAc:petroleum ether); **IR** (film, ν_{max}/cm^{-1}) 2925, 1692, 1632, 1493, 1442, 1400, 1205, 1158, 1120, 1071, 1022; **^{1}H NMR** (300 MHz, $CDCl_3$) δ 7.48–7.30 (m, 6.55H), 7.16 (s, 0.45H), 6.44 (s, 0.55H), 6.00 (s, 0.45H), 5.74 (s, 1H), 5.41 (s, 0.45H), 5.35 (s, 0.55H), 4.53 (s, 1.1H), 4.25 (s, 0.9H), 2.98 (s, 1.35H), 2.81 (s, 1.65H); **^{13}C NMR** (101 MHz, $CDCl_3$) δ 170.9 (2C), 145.2 (2C) 143.7, 143.7, 140.9, 140.5, 135.8, 135.6, 129.0, 129.0, 128.9, 128.7, 125.9, 125.7, 120.8, 120.7, 114.5, 114.3, 110.8, 109.8, 45.7, 41.3, 35.9, 32.0; **HRMS** (ESI$^+$) *m/z*: [M + H] calcd for $C_{15}H_{16}NO_2^+$ 242.1176; found 242.1188.

_N_-methyl-2-phenyl-_N_-(thiophen-3-ylmethyl)acrylamide (1q)

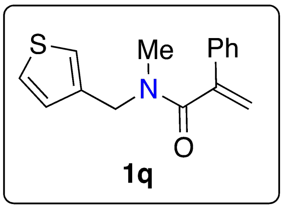

Prepared according to general procedure A using *N*-methyl-1-(thiophen-3-yl)methanamine (0.200 g, 1.57 mmol), 2-phenylacrylic acid (0.212 g, 1.43 mmol), 2-chloro-1-methylpyridinium iodide (0.473 g, 1.85 mmol) and triethylamine (0.597 mL, 4.29 mmol) in CH_2Cl_2 (20 mL). Chromatography on silica gel with EtOAc:petroleum ether (1:9 to 4:6) afforded **1q** in a 0.55:0.45 ratio of rotamers as a clear oil (0.245 g, 67% yield). **R$_f$** = 0.29 (3:7 EtOAc:petroleum ether); **IR** (film, ν_{max}/cm^{-1}) 3088, 2924, 1631, 1549, 1488, 1442, 1399, 1281, 1259, 1233, 1203,

1120, 11076, 1028; **^{1}H NMR** (300 MHz, $CDCl_3$) δ 7.48–7.18 (m, 6H), 7.19 (br s, 0.55H), 7.10 (d, *J* = 4.8 Hz, 0.55H), 6.95–6.94 (br s, 0.45H), 6.73 (d, *J* = 4.8 Hz, 0.45H), 5.75 (s, 0.55H), 5.74 (s, 0.45H), 5.42 (s, 0.45H), 5.37 (s, 0.55H), 4.70 (s, 1.1H), 4.41 (s, 0.9H), 2.99 (s, 1.35H), 2.81 (s, 1.65H); **^{13}C NMR** (101 MHz, $CDCl_3$) δ 171.0, 170.8, 145.4, 145.3, 137.8, 137.6, 135.9, 135.7, 129.0, 129.0, 128.9, 128.7, 127.9, 126.7, 126.7, 126.4, 125.9, 125.8, 123.2, 122.6, 114.4, 114.2, 50.1, 45.6, 36.1, 32.3; **HRMS** (ESI$^+$) *m/z*: [M + H] calcd for $C_{15}H_{16}NOS^+$ 258.0947; found 258.0966.

_N_-allyl-_N_-methyl-2-phenylacrylamide (1r)

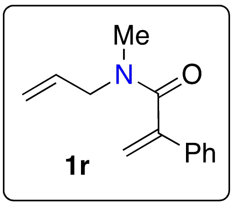

Prepared according to general procedure A using *N*-methylprop-2-en-1-amine (156 mg, 2.20 mmol), 2-phenylacrylic acid (296 mg, 2.00 mmol), 2-chloro-1-methylpyridinium iodide (664 mg, 2.60 mmol) and triethylamine (0.84 mL, 6.00 mmol) in CH_2Cl_2 (11 mL). Chromatography on silica gel with EtOAc:petroleum ether (1:9 to 1:3) afforded **1r** in a 0.55:0.45 ratio of rotamers as a clear oil (105 mg, 26% yield). **R_f** = 0.52 (6:4 EtOAc:petroleum ether); **IR** (film, $ν_{max}$/cm^{-1}) 2986, 2924, 1708, 1630, 1491, 1441, 1400, 1217, 1142, 1075; **^{1}H NMR** (300 MHz, $CDCl_3$) δ 7.43–7.29 (m, 5H), 5.88–5.75 (m, 0.45H), 5.73 (s, 0.45H), 5.69 (s, 0.55H), 5.60–5.47 (m, 0.55H), 5.35 (s, 0.55H), 5.34 (s, 0.45H), 5.22–5.06 (m, 2H), 4.11 (d, *J* = 5.8Hz, 0.9H), 3.80 (d, *J* = 5.8 Hz, 1.1H), 2.99 (s, 1.65H), 2.82 (s, 1.35H); **^{13}C NMR** (101 MHz, $CDCl_3$) δ 171.1, 170.7, 145.4, 145.1, 135.7, 135.6, 132.8, 132.6, 128.9, 128.9, 128.7, 128.6, 125.8, 125.7, 117.9, 117.8, 114.1, 113.9, 53.5, 49.4, 36.0, 32.2; **HRMS** (ESI$^+$) *m/z*: [M + H] calcd for $C_{13}H_{16}NO^+$ 202.1226; found 202.1238.

_N_-methyl-2-phenyl-_N_-(prop-2-yn-1-yl)acrylamide (1s)

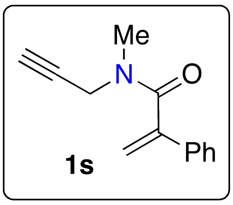

Prepared according to general procedure A using *N*-methylprop-2-yn-1-amine (228 mg, 3.30 mmol), 2-phenylacrylic acid (444 mg, 3.00 mmol), 2-chloro-1-methylpyridinium iodide (996 mg, 3.90 mmol) and triethylamine (1.25 mL, 9.00 mmol) in CH_2Cl_2 (17 mL). Chromatography on silica gel with EtOAc:petroleum ether (1:9 to 1:3) afforded **1s** in a 0.6:0.4 ratio of rotamers as a white solid (287 mg, 48% yield). **R_f** = 0.58 (6:4 EtOAc:petroleum ether); **M.P.** 60–62 °C; **IR** (film, $ν_{max}$/cm^{-1}) 3288, 3237, 2925, 1635, 1488, 1444, 1397, 1256, 1216, 1124, 1076, 1029; **^{1}H NMR** (300 MHz, $CDCl_3$) δ 7.44–7.29 (m, 5H), 5.79 (s, 0.4H), 5.77 (s, 0.6H), 5.45 (s, 0.4H), 5.39

(s, 1H), 4.35 (d, *J* = 2.5 Hz, 1.2H), 4.01 (d, *J* = 2.5 Hz, 0.8H), 3.13 (s, 1.2H), 2.95 (s, 1.8H), 2.26–2.23 (m, 1H); **^{13}C NMR** (101 MHz, $CDCl_3$) δ 170.7, 170.6, 144.7, 144.2, 153.3, 129.0, 128.9, 128.8, 128.7, 125.9, 125.9, 125.7, 114.6, 114.6, 78.3, 78.0, 72.3, 72.2, 40.8, 35.9, 35.7, 32.1; **HRMS** (ESI$^+$) *m/z*: [M + H] calcd for $C_{13}H_{14}NO^+$ 200.1070; found 200.1084.

***N*-(cyanomethyl)-*N*-methyl-2-phenylacrylamide (1t)**

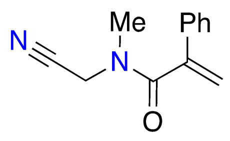

1t

Prepared according to general procedure A using 2-(methylamino)acetonitrile (0.154 g, 2.2 mmol), 2-phenylacrylic acid (0.297 g, 2 mmol), 2-chloro-1-methylpyridinium iodide (0.665 g, 2.6 mmol) and triethylamine (0.836 mL, 6 mmol) in CH_2Cl_2 (25 mL). Chromatography on silica gel with EtOAc:petroleum ether (1:9 to 4:6) afforded **1t** in a 0.75:0.25 ratio of rotamers as a yellow oil (0.358 g, 90% yield). **R_f** = 0.39 (3:7 EtOAc:petroleum ether); **IR** (film, ν_{max}/cm^{-1}) 3058, 3027, 2984, 2931, 1647, 1558, 1486, 1448, 1395, 1343, 1263, 1200, 1128, 1077, 1028; **^{1}H NMR** (300 MHz, $CDCl_3$) δ 7.39–7.33 (m, 5H), 5.87 (s, 0.25H), 5.83 (s, 0.75H), 5.52 (s, 0.25H), 5.45 (s, 0.75H), 4.48 (s, 1.5H), 4.15 (s, 0.5H), 3.18 (s, 0.75H), 3.00 (s, 2.25H); **^{13}C NMR** (101 MHz, $CDCl_3$) δ (major rotamer) 171.0, 143.7, 134.8, 129.4, 129.2, 129.1, 125.7, 116.0, 36.6, 34.9 (diagnostic signals for the minor rotamer can be seen at 115.0, 39.6, 33.0); **HRMS** (ESI$^+$) *m/z*: [M + H] calcd for $C_{12}H_{13}N_2O^+$ 201.1022; found 201.1035.

***N,N*-diisopropyl-2-phenylacrylamide (1u)**

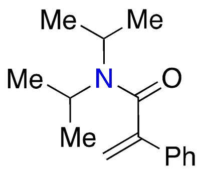

1u

Prepared according to general procedure A using diisopropylamine (278 mg, 2.75 mmol), 2-phenylacrylic acid (370 mg, 2.50 mmol), 2-chloro-1-methylpyridinium iodide (830 mg, 3.25 mmol) and triethylamine (1.05 mL, 7.50 mmol) in CH_2Cl_2 (14 mL). Chromatography on silica gel with EtOAc:petroleum ether (1:9 to 1:4) afforded **1u** as a white solid (538 mg, 93% yield). **R_f** = 0.45 (2:8 EtOAc:petroleum ether); **M.P.** 70–73 °C; **IR** (film, ν_{max}/cm^{-1}) 2967, 2928, 1630, 1442, 1372, 1345, 1256, 1214, 1155, 1135, 1039; **^{1}H NMR** (300 MHz, $CDCl_3$) δ 7.45–7.28 (m, 5H), 5.61 (s, 1H), 5.24 (s, 1H), 3.97 (h, *J* = 6.3 Hz, 1H), 3.42 (h, *J* = 6.3 Hz, 1H), 1.53 (d, *J* = 6.8 Hz, 6H), 0.99 (d, *J* = 6.8 Hz, 6H); **^{13}C NMR** (101 MHz, $CDCl_3$) δ 170.2, 146.8, 135.9, 128.8, 128.5, 125.7, 111.6, 50.8, 45.6, 20.5, 20.5; **HRMS** (ESI$^+$) *m/z*: [M + H] calcd for $C_{15}H_{22}NO^+$ 232.1696; found 232.1708.

***N*,*N*-diethyl-2-phenylacrylamide (1v)**

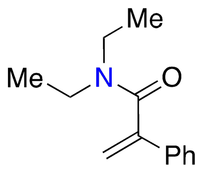

1v

Prepared according to general procedure A using diethylamine (241 mg, 3.30 mmol), 2-phenylacrylic acid (444 mg, 3.00 mmol), 2-chloro-1-methylpyridinium iodide (996 mg, 3.90 mmol) and triethylamine (1.25 mL, 9.00 mmol) in CH_2Cl_2 (17 mL). Chromatography on silica gel with EtOAc:petroleum ether (1:9 to 1:4) afforded **1v** as a clear oil (506 mg, 83% yield). **R_f** = 0.32 (3:7 EtOAc:petroleum ether); **IR** (film, ν_{max}/cm^{-1}) 2976, 2935, 1626, 1433, 1378, 1312, 1284, 1247, 1220, 1144, 1091, 908, 780, 753, 699; **^{1}H NMR** (300 MHz, $CDCl_3$) δ 7.43–7.28 (m, 5H), 5.68 (s, 1H), 5.31 (s, 1H), 3.49 (q, *J* = 7.1 Hz, 2H), 3.21 (q, *J* = 7.1 Hz, 2H), 1.20 (t, *J* = 7.1 Hz, 3H), 0.98 (d, *J* = 7.1 Hz, 3H); **^{13}C NMR** (101 MHz, $CDCl_3$) δ 170.3, 145.6, 135.8, 128.8, 128.5, 125.6, 113.0, 42.8, 38.8, 14.0, 12.8; **HRMS** (ESI^+) *m/z*: [M + H] calcd for $C_{13}H_{18}NO^+$ 204.1383; found 204.1396.

2-phenyl-1-(2-phenylpyrrolidin-1-yl)prop-2-en-1-one (1w)

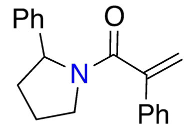

1w

Prepared according to general procedure A using 2-phenylpyrrolidine (0.324 g, 2.2 mmol), 2-phenylacrylic acid (0.297 g, 2 mmol), 2-chloro-1-methylpyridinium iodide (0.665 g, 2.6 mmol) and triethylamine (0.836 mL, 6 mmol) in CH_2Cl_2 (25 mL). Chromatography on silica gel with EtOAc:petroleum ether (1:9 to 3:7) afforded **1w** in a 1:1 ratio of rotamers as a yellow oil (0.313 g, 56% yield). **R_f** = 0.19 (3:7 EtOAc:petroleum ether); **IR** (film, ν_{max}/cm^{-1}) 3057, 3028, 2970, 2877, 1711, 1634, 1517, 1493, 1424, 1313, 1255, 1206, 1173, 1076, 1027; **^{1}H NMR** (300 MHz, $CDCl_3$) δ 7.49–7.19 (m, 9H), 7.05–7.02 (m, 1H), 5.75 (s, 0.5H), 5.52 (s, 0.5H), 5.38 (s, 0.5H), 5.35–5.31 (m, 0.5H), 4.98 (s, 0.5H), 4.74 (d, *J* = 6.6 Hz, 0.5H), 3.94–3.88 (m, 1H), 3.53–3.42 (m, 1H), 2.41–2.28 (m, 0.5H), 2.25–2.18 (m, 0.5H), 1.97–1.80 (m, 3H); **^{13}C NMR** (101 MHz, $CDCl_3$) δ 170.2, 169.3, 146.3, 145.1, 143.5, 143.2, 135.9, 135.6, 128.8, 128.6, 128.5, 128.4, 128.3, 128.0, 128.0, 127.0, 126.9, 126.2, 125.7, 125.7, 125.6, 115.3, 62.4, 60.5, 49.3, 46.8, 35.7, 34.5, 24.1, 21.7; **HRMS** (ESI^+) *m/z*: [M + H] calcd for $C_{19}H_{20}NO^+$ 278.1539; found 278.1553.

***N*-methyl-2-phenyl-*N*-(ferrocene)acrylamide (1x)**

Prepared according to general procedure A using *N*-methyl-1-(ferrocene)methanamine (1.145 g, 5 mmol), 2-phenylacrylic acid (0.674, 4.54 mmol), 2-chloro-1-methylpyridinium iodide (1.507 g, 5.90 mmol) and triethylamine (1.9 mL, 13.62 mmol) in CH_2Cl_2 (25 mL).

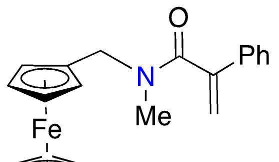
1x

Chromatography on silica gel with EtOAc:petroleum ether (1:9 to 3:7) afforded **1x** in a 1:1 ratio of rotamers as an orange oil (0.340 g, 21% yield). **R$_f$** = 0.19 (3:7 EtOAc:petroleum ether); **IR** (film, ν_{max}/cm^{-1}) 3086, 2922, 2860, 1583, 1631, 1488, 1440, 1396, 1301, 1280, 1257, 1233, 1182, 1104, 1075, 1029, 1001; **^{1}H NMR** (300 MHz, $CDCl_3$) δ 7.51–7.29 (m, 5H), 5.82 (s, 0.5H), 5.72 (s, 0.5H), 5.44 (s, 0.5H), 5.28 (s, 0.5H), 4.60 (m, 5H), 4.18–3.92 (m, 6H), 2.94–2.87 (m, 3H); **^{13}C NMR** (151 MHz, $CDCl_3$) δ 170.3, 145.4, 136.2, 135.5, 129.0, 128.9, 128.5, 126.0, 125.7, 114.6, 114.0, 69.5, 69.0, 68.7, 50.4, 46.3, 36.3, 31.6; **HRMS** (ESI$^+$) *m/z*: [M + H] calcd for $C_{21}H_{22}FeNO^+$ 360.1045; found 360.1053.

2-phenyl-1-(piperidin-1-yl)prop-2-en-1-one (6y)[11]

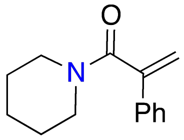
1y

Prepared according to general procedure A using piperidine (0.187 g, 2.2 mmol), 2-phenylacrylic acid (0.297 g, 2 mmol), 2-chloro-1-methylpyridinium iodide (0.665 g, 2.6 mmol) and triethylamine (0.836 mL, 6 mmol) in CH_2Cl_2 (25 mL). Chromatography on silica gel with EtOAc:petroleum ether (1:9 to 4:6) afforded **1y** as a yellow oil (0.115 g, 27% yield). **R$_f$** = 0.21 (3:7 EtOAc:petroleum ether); **IR** (film, ν_{max}/cm^{-1}) 2932, 2857, 1635, 1441, 1279, 1248, 1218, 1132, 1069, 1013; **^{1}H NMR** (300 MHz, $CDCl_3$) δ 7.45–7.41 (m, 2H), 7.37–7.30 (m, 3H), 5.71 (s, 1H), 5.33 (s, 1H), 3.69–3.66 (m, 2H), 3.30 (app t, *J* = 5.7 Hz, 2H), 1.63–1.59 (m, 4H), 1.38–1.34 (m, 2H); **HRMS** (ESI$^+$) *m/z*: [M + H] calcd for $C_{14}H_{18}NO^+$ 216.1383; found 216.1391. Data in accordance with those previously reported.

7. Characterization data of acrylamides 2.

N-benzyl-*N*-ethyl-2-phenylacrylamide (2a)

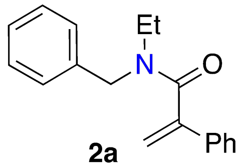
2a

Prepared according to general procedure A using *N*-benzylethanamine (0.297 g, 2.2 mmol), 2-phenylacrylic acid (0.297 g, 2 mmol), 2-chloro-1-methylpyridinium iodide (0.665 g, 2.6 mmol) and triethylamine (0.836 mL, 6 mmol) in CH_2Cl_2 (25 mL). Chromatography on silica gel with EtOAc:petroleum ether (1:9 to 3:7) afforded **2a** in a 0.55:0.45 ratio of as a colourless oil (0.510 g, 96% yield). **R$_f$** = 0.51 (3:7 EtOAc:petroleum ether); **IR** (film, ν_{max}/cm^{-1}) 3059, 3029, 2973, 2931, 1692, 1634, 1549, 1495, 1430, 1366, 1309, 1285, 1235, 1207, 1178,

1125, 1077, 1028; **^{1}H NMR** (300 MHz, $CDCl_3$) δ 7.49–7.45 (m, 2H), 7.40–7.25 (m, 7H), 7.10–7.08 (m, 1H), 5.73 (s, 0.55H), 5.70 (s, 0.45H), 5.40 (s, 1H), 4.73 (s, 1.1H), 4.42 (s, 0.9H), 3.47 (q, *J* = 7.1 Hz, 0.9H), 3.18 (q, *J* = 7.1 Hz, 1.1H), 1.17 (t, *J* = 7.1 Hz, 1.35H), 0.97 (t, *J* = 7.1 Hz, 1.65H); **^{13}C NMR** (101 MHz, $CDCl_3$) δ 171.0, 170.8, 145.4, 145.1, 137.5, 136.7, 135.8, 135.7, 129.0, 128.9, 128.7, 128.7, 128.2, 127.6, 127.5, 127.1, 125.8, 125.7, 113.9, 113.6, 51.5, 46.5, 42.3, 38.9, 13.4, 12.1; **HRMS** (ESI$^+$) *m/z*: [M + H] calcd for $C_{18}H_{20}NO^+$ 266.1539; found 266.1550.

***N*,*N*-dibenzyl-2-phenylacrylamide (2b)**

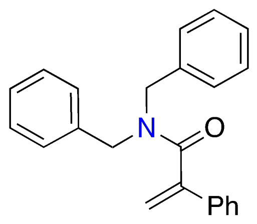

2b

Prepared according to general procedure A using dibenzylamine (434 mg, 2.20 mmol), 2-phenylacrylic acid (296 mg, 2.00 mmol), 2-chloro-1-methylpyridinium iodide (664 mg, 2.60 mmol) and triethylamine (0.84 mL, 6.00 mmol) in CH_2Cl_2 (11 mL). Chromatography on silica gel with EtOAc:petroleum ether (1:9 to 1:4) afforded **2b** as a white solid (524 mg, 80% yield). **R_f** = 0.59 (3:7 EtOAc:petroleum ether); **M.P.** 54–55 °C; **IR** (film, ν_{max}/cm^{-1}) 3060, 2926, 1635, 1495, 1423, 1362, 1322, 1206, 1077, 1022; **^{1}H NMR** (300 MHz, $CDCl_3$) δ 7.51–7.48 (m, 2H), 7.40–7.26 (m, 11H), 7.09–7.06 (m, 2H), 5.74 (s, 1H), 5.50 (s, 1H), 4.66 (s, 2H), 4.34 (s, 2H); **^{13}C NMR** (101 MHz, $CDCl_3$) δ 171.3, 145.1, 136.9, 136.2, 135.9, 129.0, 128.8, 128.8, 128.7, 128.7, 127.8, 127.7, 127.4, 125.9, 114.5, 51.0, 46.4; **HRMS** (ESI$^+$) *m/z*: [M + H] calcd for $C_{23}H_{22}NO^+$ 328.1696; found 328.1701.

***N*-benzyl-*N*,2-diphenylacrylamide (2c)**

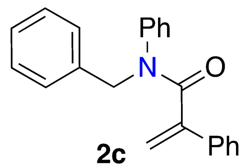

2c

Prepared according to general procedure A using *N*-benzylaniline (0.200 mg, 1.09 mmol), 2-phenylacrylic acid (0.226 mg, 1.53 mmol), 2-chloro-1-methylpyridinium iodide (0.334 mg, 1.31 mmol) and triethylamine (0.456 mL, 3.27 mmol) in CH_2Cl_2 (10 mL). Chromatography on silica gel with EtOAc:petroleum ether (1:9 to 3:7) afforded **2c** as a colourless oil (0.222 g, 65% yield). **R_f** = 0.42 (2:8 EtOAc:petroleum ether); **IR** (film, ν_{max}/cm^{-1}) 3058, 3030, 2929, 1645, 1592, 1493, 1448, 1390, 1329, 1284, 1240, 1205, 1076, 1027, 1004; **^{1}H NMR** (300 MHz, $CDCl_3$) δ 7.29–7.06 (m, 13H), 6.76–6.73 (m, 2H), 5.46 (s, 1H), 5.38 (s, 1H), 5.02 (s, 2H); **^{13}C NMR** (101 MHz, $CDCl_3$) δ 170.6, 145.9, 142.2, 137.5, 137.2, 128.9, 128.8, 128.7, 128.6, 128.4, 128.0, 127.6, 127.2, 126.2, 117.9, 53.2; **HRMS** (ESI$^+$) *m/z*: [M + H] calcd for $C_{22}H_{20}NO^+$ 314.1539; found 314.1551.

_N_-benzyl-2-phenyl-_N_-tosylacrylamide (2d)

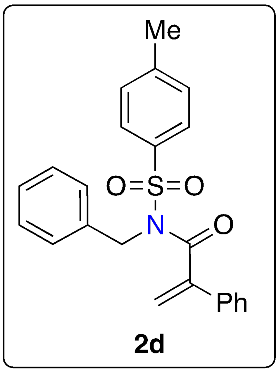

Prepared according to general procedure A using *N*-benzyl-4-methylbenzenesulfonamide (862 mg, 3.30 mmol), 2-phenylacrylic acid (444 mg, 3.00 mmol), 2-chloro-1-methylpyridinium iodide (996 mg, 3.90 mmol) and triethylamine (1.25 mL, 9.00 mmol) in CH_2Cl_2 (17 mL). Chromatography on silica gel with EtOAc:petroleum ether (1:9 to 1:4) afforded **2d** as a clear oil (446 mg, 38% yield). $\mathbf{R_f}$ = 0.58 (2:8 EtOAc:petroleum ether); **M.P.** 120–122 °C; **IR** (film, ν_{max}/cm^{-1}) 3060, 3032, 2923, 1687, 1598, 1495, 1449, 1405, 1355, 1163, 1123, 1082, 1030; **^{1}H NMR** (300 MHz, $CDCl_3$) δ 7.65 (d, *J* = 8.1 Hz, 2H), 7.46–6.98 (m, 12H), 5.65 (s, 1H), 5.28 (s, 1H), 4.88 (s, 2H), 2.42 (s, 3H); **^{13}C NMR** (101 MHz, $CDCl_3$) δ 170.5, 145.0, 144.4, 136.6, 136.1, 134.8, 129.4, 129.0, 129.0, 129.0, 128.7, 127.8, 127.7, 125.9, 118.2, 50.5, 21.8; **HRMS** (ESI$^+$) *m/z*: [M + H] calcd for $C_{23}H_{22}NO_3S^+$ 392.1315; found 392.1324.

_N_-benzyl-2-phenylacrylamide (2e)[14]

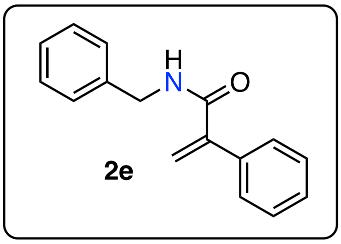

Prepared according to general procedure A using phenylmethanamine (490 mg, 4.58 mmol), 2-phenylacrylic acid (616 mg, 4.16 mmol), 2-chloro-1-methylpyridinium iodide (1382 mg, 5.41 mmol) and triethylamine (1.74 mL, 12.5 mmol) in CH_2Cl_2 (23 mL). Chromatography on silica gel with EtOAc:petroleum ether (1:9 to 3:10) afforded **2e** as a clear oil (612 mg, 62% yield). $\mathbf{R_f}$ = 0.66 (4:6 EtOAc:petroleum ether); **M.P.** 70–72 °C; **IR** (film, ν_{max}/cm^{-1}) 3315, 3062, 3032, 2970, 2926, 1734, 1704, 1655, 1607, 1528, 1449, 1356, 1305, 1328, 1229, 1077, 1047; **^{1}H NMR** (300 MHz, $CDCl_3$) δ 7.37–7.24 (m, 10H), 6.18 (s, 1H), 6.01 (brs, 1H), 5.63 (s, 1H), 4.52 (d, *J* = 5,8 Hz, 2H); **^{13}C NMR** (101 MHz, $CDCl_3$) δ 167.3, 144.8, 138.2, 137.1, 128.9, 128.9, 128.7, 128.3, 127.8, 127.7, 122.7, 44.1; **HRMS** (ESI$^+$) *m/z*: [M + H] calcd for $C_{16}H_{16}NO^+$ 238.1226; found 238.1228. Reported data in accordance with previous literature.

8. Characterization data of acrylamides 3.

N-benzyl-*N*-methyl-2-(p-tolyl)acrylamide (3a)

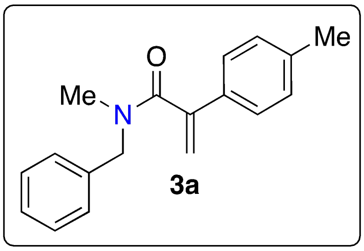

Prepared according to general procedure A using *N*-methyl-1-phenylmethanamine (0.152 g, 1.25 mmol), 2-(*p*-tolyl)acrylic acid[12] (0.185 g, 1.14 mmol), 2-chloro-1-methylpyridinium iodide (0.379 g, 1.482 mmol) and triethylamine (0.476 mL, 3.42 mmol) in CH_2Cl_2 (10 mL). Chromatography on silica gel with EtOAc:petroleum ether (1:9 to 3:7) afforded **3a** in a 0.55:0.45 ratio of rotamers as a clear oil (0.187 g, 62% yield). **R$_f$** = 0.26 (2:8 EtOAc:petroleum ether); **IR** (film, ν_{max}/cm^{-1}) 3027, 2921, 1637, 1564, 1511, 1488, 1450, 1361, 1305, 1256, 1212, 1185, 1119, 1077, 1028; **^{1}H NMR** (300 MHz, $CDCl_3$) δ 7.38–7.26 (m, 6H), 7.19 –7.15 (m, 2H), 7.10–7.07 (m, 1H), 5.72 (s, 0.55H), 5.69 (s, 0.45H), 5.38 (s, 0.45H), 5.34 (s, 0.55H), 4.72 (s, 1.1H), 4.42 (s, 0.9H), 2.97 (s, 1.35H), 2.79 (s, 1.65H), 2.36 (s, 1.35H), 2.35 (s, 1.65H); **^{13}C NMR** (151 MHz, $CDCl_3$) δ 171.6, 171.2, 145.3, 145.1, 138.8, 138.7, 137.2, 136.6, 133.0, 132.9, 129.7, 129.7, 128.8, 128.8, 128.4, 127.8, 127.6, 127.2, 125.8, 125.7, 113.5, 113.3, 54.6, 50.3, 36.0, 32.4, 21.3, 21.3; **HRMS** (ESI$^+$) *m/z*: [M + H] calcd for $C_{18}H_{20}NO^+$ 266.1539; found 266.1553.

N-benzyl-2-(4-methoxyphenyl)-*N*-methylacrylamide (3b)

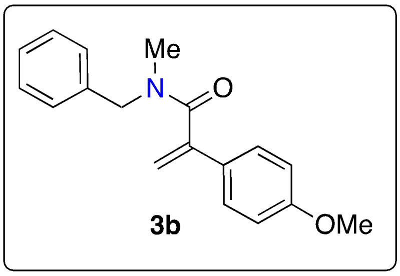

Prepared according to general procedure A using *N*-methyl-1-phenylmethanamine (44.0 mg, 0.36 mmol), 2-(4-methoxyphenyl)acrylic acid[12] (58.8 mg, 0.33 mmol), 2-chloro-1-methylpyridinium iodide (110 mg, 0.43 mmol) and triethylamine (0.14 mL, 0.99 mmol) in CH_2Cl_2 (2 mL). Chromatography on silica gel with EtOAc:petroleum ether (1:9 to 1:5) afforded **3b** in a 0.55:0.45 ratio of rotamers as a clear oil (23.2 mg, 25% yield). **R$_f$** = 0.21 (2:8 EtOAc:petroleum ether); **IR** (film, ν_{max}/cm^{-1}) 3031, 2923, 2855, 1789, 1740, 1638, 1498, 1456, 1367, 1248, 1169, 1087, 1055, 1030; **^{1}H NMR** (300 MHz, $CDCl_3$) δ 7.42–7.26 (m, 6H), 7.09–6.86 (m, 3H), 5.65 (s, 0.55H), 5.61 (s, 0.45H), 5.32 (s, 0.45H), 5.27 (s, 0.55H), 4.71 (s, 1.1H), 4.43 (s, 0.9H), 3.82 (s, 1.35H), 3.81 (s, 1.65H), 2.97 (s, 1.35H), 2.80 (s, 1.65H); **^{13}C NMR** (101 MHz, $CDCl_3$) δ 171.6, 171.3, 160.0, 159.9, 144.6, 144.3, 137.0, 136.4, 128.8, 128.8, 128.4, 128.2, 128.1, 127.7, 127.6, 127.1, 127.1, 114.3, 114.2, 112.3, 112.1, 55.4, 55.4, 54.5, 50.2, 36.0, 32.3 (one

rotameric signal not reliably assigned due to overlapping signals); **HRMS** (ESI$^+$) *m/z*: [M + H] calcd for $C_{18}H_{20}NO_2^+$ 282.1489; found 282.1502.

N-benzyl-2-(4-bromophenyl)-N-methylacrylamide (3c)

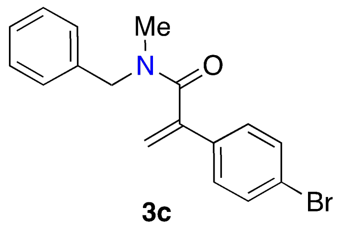

3c

Prepared according to general procedure A using *N*-methyl-1-phenylmethanamine (595 mg, 4.91 mmol), 2-(4-bromophenyl)acrylic acid[12] (1013 mg, 4.46 mmol), 2-chloro-1-methylpyridinium iodide (1481 mg, 5.80 mmol) and triethylamine (1.86 mL, 13.38 mmol) in CH_2Cl_2 (25 mL). Chromatography on silica gel with EtOAc:petroleum ether (1:9 to 1:4) afforded **8c** in a 0.55:0.45 ratio of rotamers as a clear oil (560 mg, 38% yield). $\mathbf{R_f}$ = 0.56 (3:7 EtOAc:petroleum ether); **IR** (film, ν_{max}/cm^{-1}) 2923, 2859, 1635, 1487, 1450, 1397, 1259, 1210, 1185, 1113, 1075, 1009; **^{1}H NMR** (300 MHz, $CDCl_3$) δ 7.50–7.46 (m, 2H), 7.38–7.24 (m, 6H), 7.08–7.05 (m, 1H), 5.76 (s, 0.55H), 5.72 (s, 0.45H), 5.45 (s, 0.45H), 5.41 (s, 0.55H), 4.70 (s, 1.1H), 4.42 (s, 0.9H), 2.97 (s, 1.35H), 2.79 (s, 1.65H); **^{13}C NMR** (101 MHz, $CDCl_3$) δ 170.8, 170.4, 144.3, 144.1, 136.9, 136.2, 134.8, 134.7, 132.1 (2C), 128.9, 128.8, 128.3, 127.9, 127.7, 127.5, 127.5, 127.1, 123.0, 122.8, 115.0, 114.8, 54.6, 50.4, 36.1, 32.5; **HRMS** (ESI$^+$) *m/z*: [M + H] calcd for $C_{17}H_{17}{}^{79}BrNO^+$ 330.0488; found 330.0496.

N-benzyl-2-(3,4-dichlorophenyl)-N-methylacrylamide (3d)

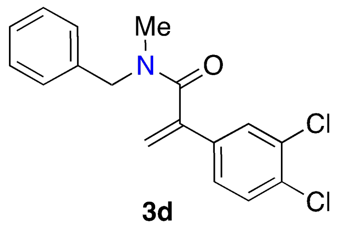

3d

Prepared according to general procedure A using *N*-methyl-1-phenylmethanamine (0.046 g, 0.38 mmol), 2-(3,4-dichlorophenyl)acrylic acid[13] (0.075 g, 0.35 mmol), 2-chloro-1-methylpyridinium iodide (0.114 g, 0.45 mmol) and triethylamine (0.144 mL, 1.04 mmol) in CH_2Cl_2 (10 mL). Chromatography on silica gel with EtOAc:petroleum ether (1:9 to 4:6) afforded **3d** in a 0.55:0.45 ratio of rotamers as a clear oil (0.053 g, 48% yield). $\mathbf{R_f}$ = 0.17 (2:8 EtOAc:petroleum ether); **IR** (film, ν_{max}/cm^{-1}) 3062, 3029, 2924, 1716, 1636, 1549, 1472, 1399, 1263, 1208, 1136, 1080, 1029; **^{1}H NMR** (300 MHz, $CDCl_3$) δ 7.56–7.53 (m, 1H), 7.44–7.26 (m, 7H), 7.09–7.06 (m, 1H), 5.77 (s, 0.55H), 5.72 (s, 0.45H), 5.48 (s, 0.45H), 5.45 (s, 0.55H), 4.70 (s, 1.1H), 4.44 (s, 0.9H), 3.00 (s, 1.35H), 2.80 (s, 1.65H); **^{13}C NMR** (151 MHz, $CDCl_3$) δ 170.3, 169.9, 143.3, 143.0, 136.8, 136.1, 135.9, 135.8, 133.3, 132.9, 132.8, 130.9, 129.2, 129.0, 128.9, 128.8, 128.4,

128.1, 128.0, 127.9, 127.9, 127.0, 125.3, 125.2, 116.0, 115.8, 54.6, 50.5, 36.1, 32.7; **HRMS** (ESI$^+$) *m/z*: [M + H] calcd for $C_{17}H_{16}{}^{35}Cl_2NO^+$ 320.0603; found 320.0622.

N-benzyl-N-methyl-2-(naphthalen-2-yl)acrylamide (3e)

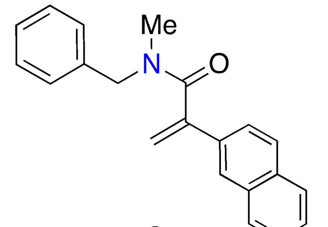

3e

Prepared according to general procedure A using *N*-methyl-1-phenylmethanamine (0.151 g, 0.76 mmol), 2-(naphthalen-2-yl)acrylic acid[12] (0.101 g, 0.84 mmol), 2-chloro-1-methylpyridinium iodide (0.252 g, 0.99 mmol) and triethylamine (0.32 mL, 2.28 mmol) in CH_2Cl_2 (10 mL). Chromatography on silica gel with EtOAc:petroleum ether (1:9 to 3:7) afforded **3e** in a 0.55:0.45 ratio of rotamers as a clear oil (0.144 g, 48% yield). **R$_f$** = 0.63 (2:8 EtOAc:petroleum ether); **IR** (film, ν_{max}/cm^{-1}) 355, 3030, 2924, 1716, 1685, 1632, 1575, 1489, 1451, 1400, 1360, 1317, 1267, 1191, 1107, 1073, 1026; **^{1}H NMR** (300 MHz, $CDCl_3$) δ 7.87–7.74 (m, 4H), 7.66–7.62 (m, 1H), 7.51– 7.46 (m, 2H), 7.39–7.24 (m, 4H), 7.10–7.08 (m, 1H), 5.90 (s, 0.55H), 5.87 (s, 0.45H), 5.54 (s, 0.45H), 5.50 (s, 0.55H), 4.78 (s,1.1H), 4.45 (s, 0.9H), 3.04 (s, 1.35H), 2.82 (s, 1.65H); **^{13}C NMR** (151 MHz, $CDCl_3$) δ 171.5, 171.1, 145.4, 145.2, 137.2, 136.5, 133.5, 133.5, 133.5, 133.4, 133.1, 132.9, 128.9, 128.8, 128.8, 128.6, 128.5, 128.5, 127.8, 127.7, 127.2, 126.7, 126.6, 126.6, 125.6, 125.5, 123.2, 123.1, 114.8, 114.4, 54.6, 50.5, 36.1, 32.5 (overlapping peaks in the ^{13}C NMR spectrum meant that some rotameric signals could not be reliably assigned); **HRMS** (ESI$^+$) *m/z*: [M + H] calcd for $C_{21}H_{20}NO^+$ 302.1539; found 302.1550.

(E)-N-benzyl-N,2-dimethylbut-2-enamide (3f)

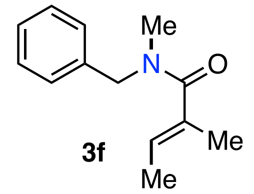

3f

Prepared according to general procedure A using *N*-methyl-1-phenylmethanamine (235 mg, 1.55 mmol), (*E*)-2-methylbut-2-enoic acid (141 mg, 1.41 mmol), 2-chloro-1-methylpyridinium iodide (468 mg, 1.83 mmol) and triethylamine (0.59 mL, 4.23 mmol) in CH_2Cl_2 (8 mL). Chromatography on silica gel with EtOAc:petroleum ether (1:9 to 1:4) afforded **3f** as a clear oil (166 mg, 58% yield). **R$_f$** = 0.41 (3:7 EtOAc:petroleum ether); **IR** (film, ν_{max}/cm^{-1}) 2926, 2859, 1769, 1712, 1647, 1440, 1383, 1277, 1220, 1010; **^{1}H NMR** (300 MHz, $CDCl_3$) δ 7.35–7.21 (m, 5H), 5.68 (q, *J* = 6.6 Hz, 1H), 4.58 (s, 2H), 2.87 (s, 3H), 1.86 (s, 3H), 1.67 (d, *J* = 6.6 Hz, 3H); **^{13}C NMR** (101 MHz, $CDCl_3$) δ 174.3, 137.1, 132.2, 128.8, 127.5, 54.6,

50.4, 36.2, 32.8, 126.0, 14.3, 13.3 (overlapping peaks in the ^{13}C NMR spectrum meant that some rotameric signals could not be reliably assigned); **HRMS** (ESI^+) *m/z*: [M + H] calcd for $C_{13}H_{18}NO^+$ 204.1383; found 204.1398.

N-benzyl-*N*-methylcyclohex-1-ene-1-carboxamide (3g)

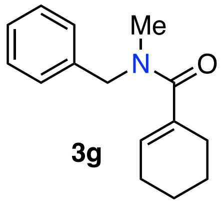

3g

Prepared according to general procedure A using *N*-methyl-1-phenylmethanamine (267 mg, 2.20 mmol), cyclohex-1-ene-1-carboxylic acid (252 mg, 2.00 mmol), 2-chloro-1-methylpyridinium iodide (664 mg, 2.60 mmol) and triethylamine (0.84 mL, 6.00 mmol) in CH_2Cl_2 (11 mL). Chromatography on silica gel with EtOAc:petroleum ether (1:9 to 1:3) afforded **3g** as a clear oil (271 mg, 59% yield). $\mathbf{R_f}$ = 0.30 (4:6 EtOAc:petroleum ether); **IR** (film, v_{max}/cm^{-1}) 2927, 2860, 1611, 1493, 1449, 1397, 1242, 1062, 1032, 734, 699; ^{1}**H NMR** (300 MHz, $CDCl_3$) δ 7.36–7.19 (m, 5H), 5.88–5.85 (m, 1H), 4.59 (s, 2H), 2.88 (s, 3H), 2.28-2.19 (m, 2H), 2.13–2.02 (m, 2H), 1.68–1.60 (m, 4H); ^{13}**C NMR** (101 MHz, $CDCl_3$) δ 173.7, 137.1, 134.5, 128.8, 127.9, 127.5, 54.7, 50.3, 36.3, 32.8, 26.1, 24.7, 22.2, 21.7 (overlapping peaks in the ^{13}C NMR spectrum meant that some rotameric signals could not be reliably assigned); **HRMS** (ESI^+) *m/z*: [M + H] calcd for $C_{15}H_{20}NO^+$ 230.1539; found 230.1554.

Synthesis and characterisation data of benzyl 2-phenylacrylate (9)[15]

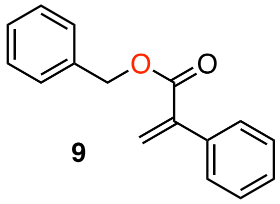

9

To a mixture of phenylmethanol (433 mg, 4.00 mmol, 2 equiv.), 2-phenylacrylic acid (296 mg, 2.00 mmol, 1 equiv.), *N*,*N'*-dicyclohexylmethanediimine (908 mg, 2.2 mmol, 1.1 equiv.) and *N*,*N*-dimethylpyridin-4-amine (33.7 mg, 0.20 mmol, 0.1 equiv.) in CH_2Cl_2 (40 mL) at 0 °C under argon and the reaction mixture allowed to warm slowly to room temperature. Once the reaction was completed (by TLC) the mixture was cooled to -20 °C for 2 hours then filtered through Celite. The organic solvents were removed in vacuo and the resulting crude product was purified by column chromatography on silica gel with EtOAc:petroleum ether (1:9 to 1:3) and afforded **9** as a clear oil (162 mg, 34% yield). $\mathbf{R_f}$ = 0.48 (3:7 EtOAc:petroleum ether); **IR** (film, v_{max}/cm^{-1}) 3033, 2930, 2856, 2117, 1718, 1615, 1496, 1451, 1400, 1378, 1309, 1277, 1177, 1086; ^{1}**H NMR** (300 MHz, $CDCl_3$) δ 7.45–7.36 (m, 10H), 6.42 (s, 1H), 5.94 (s, 1H), 5.29 (s, 2H); ^{13}**C NMR** (101 MHz, $CDCl_3$)

δ 166.7, 141.4, 136.8, 136.1, 128.7, 128.4, 128.3, 128.3, 128.3, 128.2, 127.1, 66.9. Reported data in accordance with those previously reported.

9. General procedure C: Synthesis of β-lactams (4–6) via 2-CTX mediated photocatalysis.

A stirring mixture of acrylamide **1–3** (0.10–0.5 mmol), **2-CTX** (10 mol%) in 5 mL PhMe was degassed with argon for 10 min before irradiation with 405 nm LEDs (18 W) overnight, with fan-cooling. The solvent was then removed in vacuo and the resulting residue purified by silica column chromatography (petroleum ether/EtOAc gradient) to afford β-lactam **4–6**. *Diastereomeric ratios reported were determined by ^{1}H NMR analysis of the crude reaction mixture prior to purification.*

10. Stereochemical assignment of *trans/cis* β-lactam products by ^{1}H NMR.

Trans and *cis* β-lactams diastereomers could be assigned from the ^{1}H NMR by comparison of the shielding/deshielding effects experienced by the C3 methyl group shown by the asterisk (Figure S3, *). In the *trans* product, shielding effects by the adjacent phenyl substituent — rotated perpendicular to the C3-methyl due to steric — results in the C3 methyl group occurring at ~1.2 ppm. On the other hand, due to the lack of this shielding effect in the *cis* product, the corresponding C3 methyl resonance is found relatively downfield at ~1.8 ppm. NOE experiments for a random selected set of compounds (**4f**, **4j**, **4l**) are consistent with this hypothesis. Additionally, this behaviour is similarly consistent with previous literature.[16]

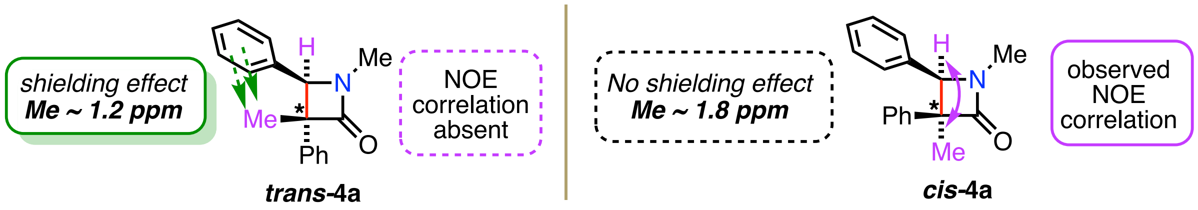

Figure S3. Shield effects observed in the ^{1}H NMR used to elucidate the *trans/cis* stereochemistry of the β-lactam products.

11. Characterization data for β-lactams (4).

1,3-dimethyl-3,4-diphenylazetidin-2-one (4a)

Prepared according to general procedure B using **1a** (0.050 g, 0.20 mmol) and **2-CTX** (0.0049 g, 0.02 mmol). Chromatography on silica gel with EtOAc:petroleum ether (1:9 to 2:8) afforded

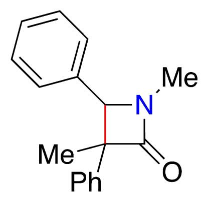

4a

4a as 2 separable diastereomers (0.044 g, 89% yield, 1.5:1 dr). **HRMS** (ESI+) *m/z*: [M + H] calcd for $C_{17}H_{18}NO^+$ 252.1383; found 252.1388.

***trans*-Diastereomer:** 0.026 g; white solid; **R$_f$** = 0.61 (4:6 EtOAc:petroleum ether); **M.P.** 101–102 °C; **IR** (film, ν_{max}/cm^{-1}) 2969, 2903, 1742, 1494, 1448, 1420, 1390, 1357, 1179, 1027; **^{1}H NMR** (300 MHz, $CDCl_3$) δ 7.51–7.45 (m, 3H), 7.44 (m, 1H), 7.42–7.37 (m, 3H), 7.33–7.29 (m, 3H), 4.75 (s, 1H), 2.91 (s, 3H), 1.15 (s, 3H); **^{13}C NMR** (101 MHz, $CDCl_3$) δ 172.1, 142.4, 135.6, 129.0, 128.9, 128.4, 127.2, 127.2, 126.1, 69.1, 63.8, 27.1, 19.8.

***cis*-Diastereomer:** 0.018 g; yellow oil; **R$_f$** = 0.33 (4:6 EtOAc:petroleum ether); **IR** (film, ν_{max}/cm^{-1}) 2958, 2922, 1744, 1673, 1451, 1422, 1390, 1362, 1282, 1027; **^{1}H NMR** (300 MHz, $CDCl_3$) δ 7.13–7.10 (m, 3H), 7.03–6.99 (m, 5H), 6.93–6.90 (m, 2H), 4.56 (s, 1H), 2.85 (s, 3H), 1.81 (s, 3H); **^{13}C NMR** (101 MHz, $CDCl_3$) δ 172.0, 138.3, 135.7, 128.3, 128.1, 128.0, 127.6, 127.3, 126.6, 70.8, 66.2, 27.1, 24.5.

1-methyl-3-(methyl-*d*)-3,4-diphenylazetidin-2-one-4-*d* (*d*-4a)

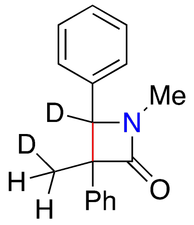

***d*-4a**

Prepared according to general procedure B using *d*-**1a** (0.050 g, 0.20 mmol) and **2-CTX** (0.0049 g, 0.02 mmol). Chromatography on silica gel with EtOAc:petroleum ether (1:9 to 2:8) afforded *d*-**4a** as 2 separable diastereomers (0.043 g, 86% yield, 1.1:1 dr).

***trans*-Diastereomer:** 0.022 g; white solid; **R$_f$** = 0.61 (4:6 EtOAc:petroleum ether); **^{1}H NMR** (300 MHz, $CDCl_3$) δ 7.51–7.45 (m, 3H), 7.44 (m, 1H), 7.42–7.37 (m, 3H), 7.33–7.30 (m, 3H), 2.91 (s, 3H), 1.13 (s, 2H).

***cis*-Diastereomer:** 0.021 g; yellow oil; **R$_f$** = 0.33 (4:6 EtOAc:petroleum ether); **^{1}H NMR** (300 MHz, $CDCl_3$) δ 7.13–7.11 (m, 3H), 7.03–6.99 (m, 5H), 6.93–6.90 (m, 2H), 2.85 (s, 3H), 1.80 (s, 2H).

1,3-dimethyl-3-phenyl-4-(*p*-tolyl)azetidin-2-one (4b)

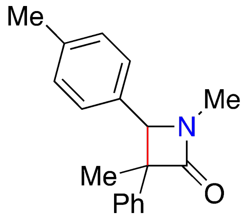

4b

Prepared according to general procedure B using **1b** (90 mg, 0.34 mmol) and **2-CTX** (8.3 mg, 0.03 mmol). Chromatography on silica gel with EtOAc:petroleum ether (1:9 to 2:8) afforded **4b** as 2 separable diastereomers (85 mg, 94% yield, 1.6:1 dr). **HRMS** (ESI+) *m/z*: [M + H] calcd for $C_{18}H_{20}NO^+$ 266.1539; found 266.1542.

***trans*-Diastereomer:** 0.053 g; yellow oil; **R$_f$** = 0.68 (3:7 EtOAc:petroleum ether); **IR** (film, ν_{max}/cm^{-1}) 2966, 2922, 1443, 1426, 1387, 1279, 1178, 1027; **^{1}H NMR** (300 MHz, $CDCl_3$) δ 7.51–7.50 (m, 2H), 7.41 (t, *J* = 7.5 Hz, 2H), 7.31–7.26 (m, 3H), 7.22–7.19 (m, 2H), 4.72 (s, 1H), 2.90 (s, 3H), 2.40 (s, 3H), 1.16 (s, 3H); **^{13}C NMR** (101 MHz, $CDCl_3$) δ 172.1, 142.5, 138.2, 132.4, 129.6, 128.8, 127.2, 127.2, 126.1, 68.9, 63.7, 27.0, 21.3, 19.8.

***cis*-Diastereomer:** 0.032 g; white solid; **R$_f$** = 0.48 (3:7 EtOAc:petroleum ether); **M.P.** 105–107 °C; **IR** (film, ν_{max}/cm^{-1}) 2955, 2924, 1742, 1638, 1446, 1391, 1280, 1122, 1069, 1027; **^{1}H NMR** (300 MHz, $CDCl_3$) δ 7.08–7.03 (m, 5H), 6.92 (d, *J* = 7.8 Hz, 2H), 6.79 (d, *J* = 7.8 Hz, 2H), 4.53 (s, 1H), 2.82 (s, 3H), 2.21 (s, 3H), 1.79 (s, 3H); **^{13}C NMR** (101 MHz, $CDCl_3$) δ 172.1, 138.5, 137.8, 132.5, 129.0, 127.9, 127.5, 127.3, 126.5, 70.6, 65.8, 27.0, 24.6, 21.2.

1,3-dimethyl-3-phenyl-4-(*o*-tolyl)azetidin-2-one (4c)

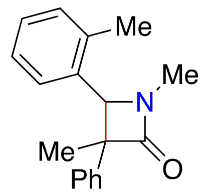

4c

Prepared according to general procedure B using **1c** (100 mg, 0.38 mmol) and **2-CTX** (9.3 mg, 0.04 mmol). Chromatography on silica gel with EtOAc:petroleum ether (1:9 to 2:8) afforded **4c** as 2 separable diastereomers (95 mg, 95% yield, 1.5:1 dr). **HRMS** (ESI$^+$) *m/z*: [M + H] calcd for $C_{18}H_{20}NO^+$ 266.1539; found 266.1549.

***trans*-Diastereomer:** 0.057 g; white solid; **R$_f$** = 0.42 (4:6 EtOAc:petroleum ether); **M.P.** 92–94 °C; **IR** (film, ν_{max}/cm^{-1}) 2961, 2927, 1747, 1454, 1415, 1389, 1027; **^{1}H NMR** (300 MHz, $CDCl_3$) δ 7.45–7.37 (m, 4H), 7.33–7.20 (m, 5H), 4.82 (s, 1H), 3.01 (s, 3H), 2.12 (s, 3H), 1.19 (s, 3H); **^{13}C NMR** (101 MHz, $CDCl_3$) δ 172.9, 141.1, 136.5, 134.1, 131.0, 128.9, 127.9, 127.4, 126.2, 126.0, 125.9, 67.5, 63.4, 27.4, 19.8, 16.2.

***cis*-Diastereomer:** 0.038 g; colourless oil; **R$_f$** = 0.28 (4:6 EtOAc:petroleum ether); **IR** (film, ν_{max}/cm^{-1}) 2961, 2924, 1744, 1448, 1422, 1388, 1352, 1028; **^{1}H NMR** (300 MHz, $CDCl_3$) δ 7.05–6.96 (m, 7H), 6.89 (t, *J* = 7.5 Hz, 1H), 6.72 (d, *J* = 7.8Hz, 1H), 4.84 (s, 1H), 2.91 (s, 1H), 2.39 (s, 3H), 1.89 (s, 3H); **^{13}C NMR** (101 MHz, $CDCl_3$) δ 172.4, 137.8, 135.7, 133.9, 130.3, 128.0, 127.5, 127.0, 126.8, 126.0, 125.8, 67.2, 65.9, 27.6, 23.6, 19.8.

1,3-dimethyl-3-phenyl-4-(3-(trifluoromethyl)phenyl)azetidin-2-one (4d)

Prepared according to general procedure B using **1d** (100 mg, 0.31 mmol) and **2-CTX** (7.7 mg, 0.03 mmol). Chromatography on silica gel with EtOAc:petroleum ether (1:9 to 2:8) afforded

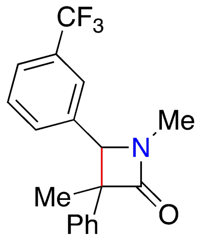

4d

4d as 2 separable diastereomers (97 mg, 97% yield, 1.6:1 dr). **HRMS** (ESI+) *m/z*: [M + H] calcd for $C_{18}H_{17}F_3NO^+$ 320.1257; found 320.1273.

***trans*-Diastereomer:** 0.062 g; yellow soild; **Rf** = 0.32 (3:7 EtOAc:petroleum ether); **M.P.** 108–109 °C; **IR** (film, ν_{max}/cm^{-1}) 2955, 2922, 1741, 1448, 1326, 1284, 1260, 1163, 1123, 1072, 1032; **^{1}H NMR** (300 MHz, $CDCl_3$) δ 7.68–7.58 (m, 2H), 7.55–7.44 (m, 4H), 7.41 (t, *J* = 7.5 Hz, 2H), 7.34–7.29 (m, 1H), 4.80 (s, 1H), 2.92 (s, 3H), 1.14 (s, 3H); **^{13}C NMR** (101 MHz, $CDCl_3$) δ 171.9, 141.8, 137.1, 131.6 (q, $J_{C\text{-}F}$ = 32.6 Hz), 130.5, 129.6, 129.1, 127.5, 126.0, 125.3 (q, $J_{C\text{-}F}$ = 3.9 Hz), 124.0 (q, $J_{C\text{-}F}$ = 273.4 Hz), 123.8 (q, $J_{C\text{-}F}$ = 3.8 Hz), 68.7, 64.2, 27.2, 19.8; **^{19}F NMR** (377 MHz, $CDCl_3$) *δ* –62.64.

***cis*-Diastereomer:** 0.035 g; oil; **Rf** = 0.19 (3:7 EtOAc:petroleum ether); **IR** (film, ν_{max}/cm^{-1}) 2968, 2927, 1738, 1392, 1326, 1156, 1116, 1071, 1083; **^{1}H NMR** (300 MHz, $CDCl_3$) δ 7.36 (d, *J* = 7.8 Hz, 1H), 7.22 (t, *J* = 7.8 Hz, 1H), 7.16 (s, 1H), 7.11–6.93 (m, 6H), 4.61 (s, 1H), 2.86 (s, 3H), 1.84 (s, 3H); **^{13}C NMR** (101 MHz, $CDCl_3$) δ 171.7, 137.6, 137.1, 130.8 (q, $J_{C\text{-}F}$ = 32.5 Hz), 130.6, 128.8, 128.2, 127.2, 126.9, 126.6 (q, $J_{C\text{-}F}$ = 273.5 Hz), 124.9, 124.4, 70.2, 66.8, 27.3, 24.0; **^{19}F NMR** (377 MHz, $CDCl_3$) *δ* –62.90.

4-(4-bromophenyl)-1,3-dimethyl-3-phenylazetidin-2-one (4e)

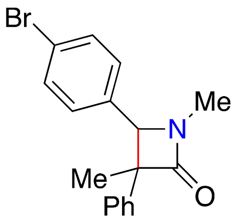

4e

Prepared according to general procedure B using **1e** (120 mg, 0.36 mmol) and **2-CTX** (8.9 mg, 0.04 mmol). Chromatography on silica gel with EtOAc:petroleum ether (1:9 to 2:8) afforded **4e** as 2 separable diastereomers (100 mg, 83% yield, 1.5:1 dr). **HRMS** (ESI+) *m/z*: [M + H] calcd for $C_{17}H_{17}{}^{79}BrNO^+$ 330.0488; found 330.0499.

***trans*-Diastereomer:** 0.060 g; oil; **Rf** = 0.65 (3:7 EtOAc:petroleum ether); **IR** (film, ν_{max}/cm^{-1}) 2965, 2921, 1746, 1487, 1443, 1424, 1386, 1069, 1028, 1008; **^{1}H NMR** (300 MHz, $CDCl_3$) δ 7.59 (d, *J* = 8.1 Hz, 2H), 7.47–7.44 (m, 2H), 7.39 (t, *J* = 7.2 Hz, 2H), 7.32–7.27 (m, 1H), 7.19 (d, *J* = 8.1 Hz, 2H), 4.69 (s, 1H), 2.89 (s, 3H), 1.14 (s, 3H); **^{13}C NMR** (101 MHz, $CDCl_3$) δ 171.9, 142.0, 134.7, 132.2, 129.0, 128.8, 127.4, 126.0, 122.4, 68.6, 63.9, 27.1, 19.8.

***cis*-Diastereomer:** 0.040 g; white solid; **Rf** = 0.35 (3:7 EtOAc:petroleum ether); **M.P.** 105–106 °C; **IR** (film, ν_{max}/cm^{-1}) 2956, 2923, 1742, 1640, 1487, 1445, 1423, 1389, 1278, 1068, 1008; **^{1}H NMR** (300 MHz, $CDCl_3$) δ 7.28 (d, *J* = 7.5 Hz, 2H), 7.10–7.06 (m, 3H), 7.04–7.00 (m, 2H), 6.81

(d, J = 8.4 Hz, 2H), 4.54 (s, 1H), 2.84 (s, 3H), 1.83 (s, 3H); **^{13}C NMR** (101 MHz, $CDCl_3$) δ 171.8, 137.9, 134.8, 131.5, 129.2, 128.2, 127.3, 126.9, 122.1, 70.1, 66.3, 27.1, 24.4;

4-(3-chlorophenyl)-1,3-dimethyl-3-phenylazetidin-2-one (4f)

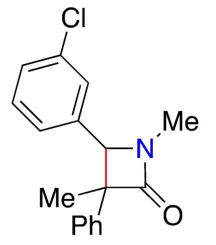

4f

Prepared according to general procedure B using **1f** (100 mg, 0.35 mmol) and **2-CTX** (8.6 mg, 0.04 mmol). Chromatography on silica gel with EtOAc:petroleum ether (1:9 to 2:8) afforded **4f** as 2 separable diastereomers (98 mg, 98% yield, 1.4:1 dr). **HRMS** (ESI$^+$) *m/z*: [M + H] calcd for $C_{17}H_{17}{}^{35}ClNO^+$ 286.0993; found 286.0999.

***trans*-Diastereomer:** 0.057 g; white solid; **R$_f$** = 0.30 (3:7 EtOAc:petroleum ether); **M.P.** 104–106 °C; **IR** (film, ν_{max}/cm^{-1}) 2958, 2924, 1737, 1435, 1390, 1274, 1125, 1073,, 1030; **^{1}H NMR** (300 MHz, $CDCl_3$) δ 7.49–7.45 (m, 2H), 7.43–7.26 (m, 6H), 7.20 (d, J = 7.2 Hz, 1H), 4.70 (s, 1H), 2.90 (s, 3H), 1.16 (s, 3H); **^{13}C NMR** (151 MHz, $CDCl_3$) δ 171.8, 142.0, 138.0, 135.2, 130.3, 129.0, 128.6, 127.4, 127.3, 126.0, 125.3, 68.6, 64.1, 27.2, 19.8.

***cis*-Diastereomer:** 0.042 g; yellow oil; **R$_f$** = 0.17 (3:7 EtOAc:petroleum ether); **IR** (film, ν_{max}/cm^{-1}) 2960, 2922, 1744, 1435, 1388, 1278, 1072, 1030; **^{1}H NMR** (300 MHz, $CDCl_3$) δ 7.10–6.99 (m, 7H), 6.93 (s, 1H), 6.76 (d, J = 7.5 Hz, 1H), 4.51 (s, 1H), 2.84 (s, 3H), 1.81 (s, 3H); **^{13}C NMR** (151 MHz, $CDCl_3$) δ 171.8, 138.0, 137.8, 134.4, 129.6, 128.2, 128.1, 127.8, 127.3, 126.9, 125.5, 70.1, 66.5, 27.2, 24.2.

4-(4-methoxyphenyl)-1,3-dimethyl-3-phenylazetidin-2-one (4g)

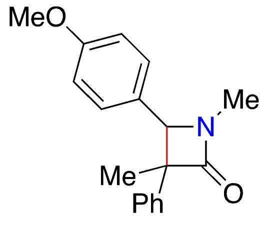

4g

Prepared according to general procedure B using **1g** (174 mg, 0.62 mmol) and **2-CTX** (15.2 mg, 0.06 mmol). Chromatography on silica gel with EtOAc:petroleum ether (0:1 to 1:7) afforded **4g** as 2 separable diastereomers (67.7 mg, 39% yield, 1.5:1 dr). **HRMS** (ESI$^+$) *m/z*: [M + H] calcd for $C_{18}H_{20}NO_2^+$ 282.1489; found 282.1502.

***trans*-Diastereomer:** 40.6 mg; clear oil; **R$_f$** = 0.46 (3:7 EtOAc:petroleum ether); **IR** (film, ν_{max}/cm^{-1}) 2957, 2924, 1745, 1611, 1513, 1444, 1389, 1298, 1248, 1176, 1028; **^{1}H NMR** (300 MHz, $CDCl_3$) 7.47 (d, J = 7.8 Hz, 2H), 7.38 (t, J = 7.5 Hz, 2H), 7.32–7.19 (m, 3H), 6.99 (d, J = 8.3 Hz, 2H), 4.69 (s, 1H), 3.85 (s, 3H), 2.88 (s, 3H), 1.16 (s, 3H); **^{13}C NMR** (101 MHz, $CDCl_3$) δ 172.2, 159.8, 142.6, 128.9, 128.5, 127.5, 127.2, 126.1, 114.5, 68.8, 63.8, 55.5, 27.0, 19.8.

***cis*-Diastereomer:** 27.1 mg; clear oil; **R_f** = 0.34 (3:7 EtOAc:petroleum ether; **IR** (film, ν_{max}/cm^{-1}) 2956, 2923, 2856, 1744, 1612, 1513, 1445, 1390, 1248, 1176, 1030; **^{1}H NMR** (300 MHz, $CDCl_3$) 7.11–6.96 (m, 5H), 6.82 (d, *J* = 8.5 Hz, 2H), 6.65 (d, *J* = 8.5 Hz, 2H), 4.51 (s, 1H), 3.70 (s, 3H), 2.81 (s, 3H), 1.78 (s, 3H); **^{13}C NMR** (101 MHz, $CDCl_3$) δ 172.1, 159.5, 138.6, 128.8, 128.0, 127.6, 127.4, 126.6, 113.8, 70.4, 65.9, 55.3, 26.9, 24.7.

4-(1,3-dimethyl-4-oxo-3-phenylazetidin-2-yl)benzonitrile (4h)

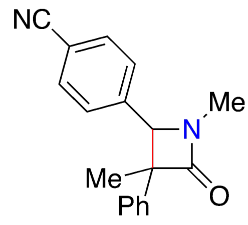

4h

Prepared according to general procedure B using **1h** (77.4 mg, 0.28 mmol) and **2-CTX** (6.9 mg, 0.03 mmol). Chromatography on silica gel with EtOAc:petroleum ether (0:1 to 1:7) afforded **4h** as 2 separable diastereomers (53.4 mg, 69% yield, 1.25:1 dr). **HRMS** (ESI$^+$) *m/z*: [M + H] calcd for $C_{18}H_{17}N_2O^+$ 277.1335; found 277.1340.

***trans*-Diastereomer:** 29.7 mg, clear oil; **R_f** = 0.43 (3:7 EtOAc:petroleum ether); **IR** (film, ν_{max}/cm^{-1}) 2965, 2923, 2855, 2228, 1748, 1607, 1443, 1426, 1389, 1346, 1171, 1028; **^{1}H NMR** (300 MHz, $CDCl_3$) δ 7.77 (d, *J* = 7.9 Hz, 2H), 7.46–7.28 (m, 7H), 4.78 (s, 1H), 2.92 (d, *J* = 1.2 Hz, 3H), 1.12 (s, 3H); **^{13}C NMR** (101 MHz, $CDCl_3$) δ 171.6, 141.5, 135.8, 132.8, 129.1, 127.9, 127.6, 126.0, 118.5, 112.5, 68.7, 64.6, 27.4, 19.7.

***cis*-Diastereomer:** 23.7 mg, white solid; **R_f** = 0.22 (3:7 EtOAc:petroleum ether; **M.P.** 180–182 °C; **IR** (film, ν_{max}/cm^{-1}) 3016, 2966, 2924, 2227, 1744, 1609, 1498, 1425, 1390, 1353, 1216, 1030; **^{1}H NMR** (300 MHz, $CDCl_3$) δ 7.40 (d, *J* = 8.0 Hz, 2H), 7.04–6.95 (m, 7H), 4.60 (s, 1H), 2.85 (s, 3H), 1.83 (s, 3H).; **^{13}C NMR** (101 MHz, $CDCl_3$) δ 171.5, 141.6, 137.4, 132.1, 128.3, 128.1, 127.2, 127.2, 118.5, 111.9, 70.2, 67.1, 27.4, 24.1.

1,3-dimethyl-3-phenyl-4-(2-(4,4,5,5-tetramethyl-1,3,2-dioxaborolan-2-yl)phenyl)azetidin-2-one (4i)

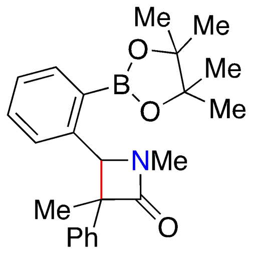

4i

Prepared according to general procedure B using **1i** (166 mg, 0.44 mmol) and **2-CTX** (10.9 mg, 0.04 mmol). Chromatography on silica gel with EtOAc:petroleum ether (0:1 to 1:7) afforded **4i** as 2 inseparable diastereomers (105 mg, 63% yield, 1.8:1 dr). **HRMS** (ESI$^+$) *m/z*: [M + H] calcd for $C_{23}H_{29}BNO_3^+$ 378.2235; found 378.2254.

Combined Diastereomers (~2:1 after chromatography): clear oil; **R_f** = 0.53 (3:7 EtOAc:petroleum ether); **IR** (film, ν_{max}/cm^{-1}) 2979, 2932, 1749, 1445, 1422, 1382,

1347, 1320, 1263, 1144, 1115, 1069, 1027; **^{1}H NMR** (300 MHz, $CDCl_3$) 5.47 (s, 0.6H), 5.38 (s, 0.3H), 2.96 (s, 2H), 2.87 (s, 1H), 1.89 (s, 1H), 1.44 (s, 4H), 1.14 (s, 2H), 1.10 (s, 4H), 1.03 (s, 4H); **^{13}C NMR** (101 MHz, $CDCl_3$) 173.1, 172.8, 142.3, 141.7, 141.7, 138.7, 136.8, 136.2, 131.0, 130.98, 128.5, 127.8, 127.3, 127.3, 127.0, 126.7, 126.4, 126.3, 126.3, 125.8, 84.2, 83.9, 68.6, 68.1, 66.5, 63.8, 27.2, 26.9, 25.2, 25.0, 24.8, 24.4, 24.3, 16.2. Overlapping peaks in the ^{13}C NMR spectrum meant that some of the signals in this diastereomeric mixture could not be reliably assigned.

4-(3,5-difluorophenyl)-1,3-dimethyl-3-phenylazetidin-2-one (4j)

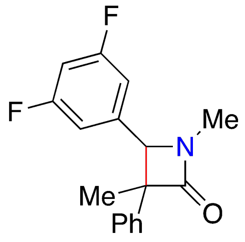

4j

Prepared according to general procedure B using **1j** (101 mg, 0.35 mmol) and **2-CTX** (8.7 mg, 0.035 mmol). Chromatography on silica gel with EtOAc:petroleum ether (1:9 to 2:8) afforded **4j** as 2 separable diastereomers (89 mg, 88% yield, 1.4:1 dr). **HRMS** (ESI$^+$) *m/z*: [M + H] calcd for $C_{17}H_{16}F_2NO^+$ 288.1194; found 288.1205.

***trans*-Diastereomer:** 0.052 g; white solid **R_f** = 0.31 (2:8 EtOAc:petroleum ether); **M.P.** 153–155 °C; **IR** (film, ν_{max}/cm^{-1}) 3059, 2916, 1744, 1624, 1596, 1460, 1443, 1393, 1341, 1115, 1038; **^{1}H NMR** (300 MHz, $CDCl_3$) δ 7.47–7.37 (m, 4H), 7.33–7.28 (m, 1H), 6.86–6.83 (m, 3H), 4.69 (s, 1H), 2.92 (s, 3H), 1.18 (s, 3H); **^{13}C NMR** (151 MHz, $CDCl_3$) δ 171.7, 163.6 (dd, $J_{C\text{-}F}$ = 250.5, 12.6 Hz), 141.6, 140.3 (t, $J_{C\text{-}F}$ = 8.2 Hz), 129.1, 127.6, 126.0, 110.0 (dd, $J_{C\text{-}F}$ = 20.4, 5.6 Hz), 104.0 (t, $J_{C\text{-}F}$ = 25.4 Hz), 68.4, 64.4, 27.4, 19.6; **^{19}F NMR** (377 MHz, CDCl3) *δ* -108.04.

***cis*-Diastereomer::** 0.037 g; white solid; **R_f** = 0.11 (2:8 EtOAc:petroleum ether); **M.P.** 113–115 °C; **IR** (film, ν_{max}/cm^{-1}) 2958, 2924, 2867, 1748, 1625, 1597, 1459, 1390, 1346, 1318, 1117; **^{1}H NMR** (300 MHz, $CDCl_3$) δ 7.12–7.00 (m, 5H), 6.57–6.51 (m, 1H), 6.47–6.44 (m, 2H), 4.50 (s, 1H), 2.86 (s, 3H), 1.81 (s, 3H); **^{13}C NMR** (151 MHz, $CDCl_3$) δ 171.5, 163.0 (dd, $J_{C\text{-}F}$ = 249.7, 12.8 Hz), 140.4 (t, $J_{C\text{-}F}$ = 8.5 Hz), 137.5, 128.3, 127.2, 127.1, 110.3 (d, $J_{C\text{-}F}$ = 20.5, 5.4 Hz), 103.5 (t, $J_{C\text{-}F}$ = 25.4 Hz), 69.9, 66.9, 27.4, 24.0; **^{19}F NMR** (377 MHz, CDCl3) *δ* -109.37;

4-(3-bromo-5-methoxyphenyl)-1,3-dimethyl-3-phenylazetidin-2-one (4k)

Prepared according to general procedure B using **1k** (155 mg, 0.43 mmol) and **2-CTX** (10.6 mg, 0.04 mmol). Chromatography on silica gel with EtOAc:petroleum ether (0:1 to 1:5) afforded **4k** as 2 separable diastereomers (107 mg, 69% yield, 1.7:1 dr). **IR** (film, ν_{max}/cm^{-1})

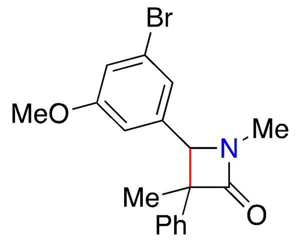

4k

2965, 2926, 1746, 1598, 1570, 1458, 1431, 1389, 1339, 1314, 1270, 1156, 1051, 1029; **HRMS** (ESI+) *m/z*: [M + H] calcd for $C_{18}H_{19}{}^{79}BrNO_2^+$ 360.0594; found 360.0610.

***trans*-Diastereomer:** 67.4 mg; $\mathbf{R_f}$ = 0.50 (4:6 EtOAc:petroleum ether); **^{1}H NMR** (300 MHz, $CDCl_3$) 7.45 (d, *J* = 7.7 Hz, 2H), 7.38 (t, *J* = 7.5 Hz, 2H), 7.33–7.27 (m, 1H), 7.07–7.00 (m, 2H), 6.75 (s, 1H), 4.64 (s, 1H), 3.83 (s, 3H), 2.89 (s, 3H), 1.19 (s, 3H); **^{13}C NMR** (101 MHz, $CDCl_3$) 171.8, 160.9, 141.9, 139.2, 129.0, 127.4, 126.0, 123.6, 122.4, 116.6, 112.4, 68.5, 64.2, 55.8, 27.3, 19.6.

***cis*-Diastereomer:** 39.6 mg; $\mathbf{R_f}$ = 0.29 (4:6 EtOAc:petroleum ether; **^{1}H NMR** (300 MHz, $CDCl_3$) 7.12–6.02 (m, 5H), 6.79 (s, 1H), 6.73 (s, 1H), 6.24 (s, 1H), 4.44 (s, 1H), 3.54 (s, 3H), 2.83 (s, 3H), 1.80 (s, 3H); **^{13}C NMR** (101 MHz, $CDCl_3$) 171.7, 160.3, 139.1, 137.9, 128.2, 127.2, 127.0, 123.4, 122.6, 117.3, 111.7, 70.0, 66.5, 55.6, 27.3, 24.2.

1,3-dimethyl-3-phenyl-4-(3,4,5-trichlorophenyl)azetidin-2-one (4l)

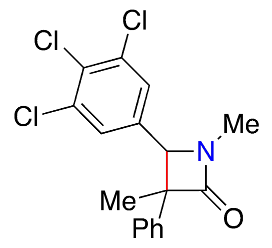

4l

Prepared according to general procedure B using **1l** (100 mg, 0.28 mmol) and **2-CTX** (7.0 mg, 0.03 mmol). Chromatography on silica gel with EtOAc:petroleum ether (1:9 to 2:8) afforded **4l** as 2 separable diastereomers (97 mg, 97% yield, 1.3:1 dr). **HRMS** (ESI+) *m/z*: [M + H] calcd for $C_{17}H_{15}{}^{35}Cl_3NO^+$ 354.0214; found 354.0225.

***trans*-Diastereomer:** 0.054 g; white solid; $\mathbf{R_f}$ = 0.26 (3:7 EtOAc:petroleum ether); **M.P.** 187–189 °C; **IR** (film, ν_{max}/cm^{-1}) 2957, 2923, 1746, 1641, 1425, 1392, 1265, 1033; **^{1}H NMR** (300 MHz, $CDCl_3$) δ 7.42–7.35 (m, 4H), 7.30–7.23 (m, 3H), 4.62 (s, 1H), 2.88 (s, 3H), 1.17 (s, 3H); **^{13}C NMR** (151 MHz, $CDCl_3$) δ 171.6, 141.4, 136.7, 135.2, 131.6, 129.1, 127.7, 127.2, 126.0, 67.9, 64.6, 27.4, 19.8.

***cis*-Diastereomer:** 0.043 g; white solid; $\mathbf{R_f}$ = 0.18 (3:7 EtOAc: petroleum ether); **M.P.** 138–140 °C; **IR** (film, ν_{max}/cm^{-1}) 2955, 2925, 2856, 1742, 1639, 1426, 1388, 1333, 1271, 1224, 1032; **^{1}H NMR** (300 MHz, $CDCl_3$) δ 7.16–7.09 (m, 3H), 7.02 (d, *J* = 7.8 Hz, 2H), 6.92 (s, 2H), 4.44 (s, 1H), 2.84 (s, 3H), 1.82 (s, 3H); **^{13}C NMR** (151 MHz, $CDCl_3$) δ 171.4, 137.2, 136.7, 134.3, 130.9, 128.5, 127.6, 127.5, 127.1, 69.3, 67.1, 27.4, 23.9.

1,3-dimethyl-4-(naphthalen-1-yl)-3-phenylazetidin-2-one (4m)

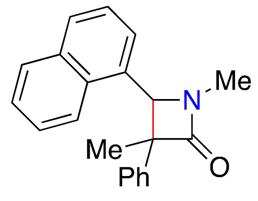

4m

Prepared according to general procedure B using **1m** (101 mg, 0.34 mmol) and **2-CTX** (8.3 mg, 0.03 mmol). Chromatography on silica gel with EtOAc:petroleum ether (0:1 to 1:7) afforded **2m** as 2 separable diastereomers (76.0 mg, 75% yield, 1:5 dr). **HRMS** (ESI$^+$) *m/z*: [M + H] calcd for $C_{21}H_{20}NO^+$ 302.1539; found 302.1541.

***trans*-Diastereomer:** 12.7 mg, clear oil, **R$_f$** = 0.54 (3:7 EtOAc:petroleum ether); **IR** (film, ν_{max}/cm^{-1}) 2919, 2855, 1457, 1376, 1067; **^{1}H NMR** (300 MHz, $CDCl_3$) 7.92 (d, *J* = 8.1 Hz, 1H), 7.86 (d, *J* = 8.1 Hz, 1H), 7.59–7.36 (m, 10H), 5.32 (s, 1H), 3.12 (s, 3H), 1.61 (s, 3H), 1.13 (s, 3H); **^{13}C NMR** (101 MHz, $CDCl_3$) 173.1, 140.9, 134.0, 132.0, 131.7, 129.2, 129.1, 128.6, 127.7, 126.7, 126.2, 126.2, 125.4, 123.8, 123.2, 67.7, 64.1, 27.8, 16.0.

***cis*-Diastereomer:** 63.3 mg, clear oil, **R$_f$** = 0.46 (3:7 EtOAc:petroleum ether; **IR** (film, ν_{max}/cm^{-1}) 2959, 2922, 2858, 1748, 1447, 1386, 1259 1231, 1049; **^{1}H NMR** (300 MHz, $CDCl_3$) 8.06 (d, *J* = 8.7 Hz, 1H), 7.80 (d, *J* = 8.1 Hz, 1H), 7.64–7.50 (m, 3H), 7.18 (t, *J* = 8.1 Hz, 1H), 6.98 (d, *J* = 7.4 Hz, 1H), 6.92–6.82 (m, 5H), 5.42 (s, 1H), 3.00 (s, 3H), 2.02 (s, 3H); **^{13}C NMR** (101 MHz, $CDCl_3$) 172.5, 137.7, 133.6, 131.7, 131.6, 129.2, 128.1, 127.7, 126.9, 126.7, 126.6, 125.8, 125.1, 123.9, 122.4, 67.0, 66.6, 27.7, 23.4.

1,3,4-trimethyl-3,4-diphenylazetidin-2-one (4n)

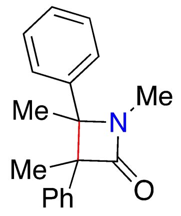

4n

Prepared according to general procedure B using **1n** (100 mg, 0.37 mmol) and **2-CTX** (9.3 mg, 0.037 mmol). Chromatography on silica gel with EtOAc:petroleum ether (1:9 to 2:8) afforded **4n** as 2 separable diastereomers (88 mg, 88% yield, 1.7:1 dr). **HRMS** (ESI$^+$) *m/z*: [M + H] calcd for $C_{18}H_{20}NO^+$ 266.1539; found 266.1552.

***trans*-Diastereomer:** 0.056 g; white solid; **R$_f$** = 0.39 (3:7 EtOAc:petroleum ether); **M.P.** 138–139 °C; **IR** (film, ν_{max}/cm^{-1}) 2972, 2925, 1735, 1492, 1445, 1419, 1386, 1260, 1064, 1023; **^{1}H NMR** (300 MHz, $CDCl_3$) δ 7.47–7.28 (m, 10H), 2.94 (s, 3H), 1.27 (s, 3H), 1.12 (s, 3H); **^{13}C NMR** (101 MHz, $CDCl_3$) δ 172.2, 141.0, 140.0, 128.8, 128.6, 127.5, 127.2, 126.9, 126.1, 69.0, 66.4, 24.8, 22.7, 21.7.

***cis*-Diastereomer:** 0.032 g; white solid; **R$_f$** = 0.25 (3:7 EtOAc:petroleum ether); **M.P.** 138–140 °C; **IR** (film, ν_{max}/cm^{-1}) 2975, 2941, 1727, 1494, 1447, 1422, 1392, 1266, 1285, 1069, 1028; **^{1}H NMR** (300 MHz, $CDCl_3$) δ 7.09–6.89 (m, 10H), 2.84 (s, 3H), 1.90 (s, 3H), 1.71 (s, 3H); **^{13}C NMR**

(101 MHz, $CDCl_3$) δ 172.0, 140.6, 140.1, 127.9, 127.7, 127.1, 126.9, 126.6, 126.2, 68.5, 67.9, 24.3, 20.5, 19.8.

1,3-dimethyl-4-(1-methyl-1*H*-indol-3-yl)-3-phenylazetidin-2-one (4o)

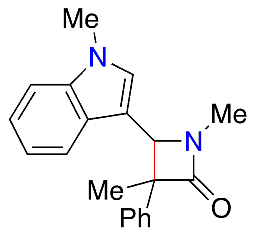

4o

Prepared according to general procedure B using **1o** (48 mg, 0.16 mmol) and **2-CTX** (3.87 mg, 0.02 mmol). Chromatography on silica gel with EtOAc:petroleum ether (1:9 to 2:8) afforded colourless oil **4o** as 2 inseparable diastereomers (25 mg, 52% yield, 1.6:1 dr).

Combined diastereomers: $\mathbf{R_f}$ = 0.14 (3:7 EtOAc:petroleum ether); **IR** (film, ν_{max}/cm^{-1}) 3057, 2960, 2922, 1737, 1664, 1545, 1668, 1424, 1387, 1241, 1068; **^{1}H NMR** (300 MHz, $CDCl_3$) δ 7.65–7.59 (m, 0.6H), 7.54–7.51 (m, 1.2H), 7.43–7.26 (m, 3.8H), 7.21–6.96 (m, 4H), 6.50 (s, 0.4H), 5.00 (s, 0.6H), 4.90 (s, 0.4H), 3.85 (s, 1.8H), 3.60 (s, 1.2H), 2.90 (s, 1.8H), 2.83 (s, 1.2H), 1.84 (s, 1.2H), 1.32 (s, 1.8H); **^{13}C NMR** (101 MHz, $CDCl_3$) δ 172.4, 172.2, 142.8, 139.5, 137.5, 137.0, 128.9, 128.2, 127.9, 127.8, 127.3, 127.2, 127.1, 127.0, 126.5, 126.0, 122.3, 121.8, 120.1, 119.6, 119.5, 118.9, 109.9, 109.4, 109.0, 65.3, 64.1, 63.9, 63.8, 33.2, 32.9, 26.9, 24.8, 19.6; **HRMS** (ESI^+) *m/z*: [M + H] calcd for $C_{20}H_{21}N_2O^+$ 305.1648; found 305.1660.

4-(furan-3-yl)-1,3-dimethyl-3-phenylazetidin-2-one (4p)

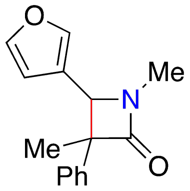

4p

Prepared according to general procedure B using **1p** (100 mg, 0.41 mmol) and **2-CTX** (10.2 mg, 0.04 mmol). Chromatography on silica gel with EtOAc:petroleum ether (1:9 to 2:8) afforded **4p** as 2 separable diastereomers (77 mg, 77% yield, 1.5:1 dr). **HRMS** (ESI^+) *m/z*: [M + H] calcd for $C_{15}H_{16}NO_2^+$ 242.1176; found 242.1192.

***trans*-Diastereomer:** 0.046 g; colourless oil; $\mathbf{R_f}$ = 0.40 (3:7 EtOAc:petroleum ether); **IR** (film, ν_{max}/cm^{-1}) 3124, 2905, 1743, 1497, 1445, 1425, 1389, 1163, 1026; **^{1}H NMR** (300 MHz, $CDCl_3$) δ 7.52–7.48 (m, 2H), 7.45–7.34 (m, 4H), 7.30–7.25 (m, 1H), 6.42 (s, 1H), 4.65 (s, 1H), 2.85 (s, 3H), 1.32 (s, 3H); **^{13}C NMR** (101 MHz, $CDCl_3$) δ 171.9, 144.1, 142.1, 140.9, 128.9, 127.3, 126.0, 120.9, 109.6, 63.2, 61.7, 26.8, 20.2.

***cis*-Diastereomer:** 0.031 g; white solid; $\mathbf{R_f}$ = 0.23 (3:7 EtOAc:petroleum ether); **M.P.** 104–106 °C; **IR** (film, ν_{max}/cm^{-1}) 2960, 2914, 1743, 1444, 1423, 1392, 1372, 1154, 1022; **^{1}H NMR** (300 MHz, $CDCl_3$) δ 7.26 (s, 1H), 7.18–7.12 (m, 5H), 7.09 (m, 1H), 5.61 (s, 1H), 4.49 (s, 1H), 2.78 (s,

3H), 1.76 (s, 3H); ^{13}**C NMR** (101 MHz, $CDCl_3$) δ 171.6, 143.5, 141.4, 138.7, 128.2, 127.2, 127.0, 121.5, 109.0, 64.8, 62.5, 26.8, 24.2;

1,3-dimethyl-3-phenyl-4-(thiophen-3-yl)azetidin-2-one (4q)

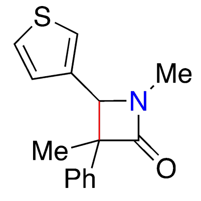

4q

Prepared according to general procedure B using **1q** (100 mg, 0.39 mmol) and **2-CTX** (9.6 mg, 0.04 mmol). Chromatography on silica gel with EtOAc:petroleum ether (1:9 to 2:8) afforded **4q** as 2 separable diastereomers (97 mg, 97% yield, 1.3:1 dr). **HRMS** (ESI^+) *m/z*: [M + H] calcd for $C_{15}H_{16}NOS^+$ 258.0947; found 258.0961.

***trans*-Diastereomer:** 0.056 g; yellow soild; $\mathbf{R_f}$ = 0.20 (3:7 EtOAc:petroleum ether); **M.P.** 96–97 °C; **IR** (film, ν_{max}/cm^{-1}) 3083, 2965, 2907, 1735, 1647, 1492, 1442, 1423, 1388, 1321, 1257, 1027; ^{1}**H NMR** (300 MHz, $CDCl_3$) δ 7.48–7.36 (m, 5H), 7.31–7.23 (m, 2H), 7.07 (d, *J* = 5.1 Hz, 1H), 4.81 (s, 1H), 2.90 (s, 3H), 1.22 (s, 3H); ^{13}**C NMR** (101 MHz, $CDCl_3$) δ 172.0, 142.2, 137.5, 128.9, 127.3, 127.0, 126.5, 126.0, 122.8, 65.4, 63.8, 27.1, 19.9.

***cis*-Diastereomer:** 0.042 g; yellow solid; $\mathbf{R_f}$ = 0.10 (3:7 EtOAc:petroleum ether); **M.P.** 103–104 °C; **IR** (film, ν_{max}/cm^{-1}) 2959, 2922, 1740, 1641, 1444, 1423, 1390, 1372, 1261, 1073, 1026; ^{1}**H NMR** (300 MHz, $CDCl_3$) δ 7.12–7.06 (m, 5H), 7.03–7.01 (m, 1H), 6.95–6.93 (m, 1H), 6.46 (d, *J* = 4.8 Hz, 1H), 4.66 (s, 1H), 2.83 (s, 3H), 1.78 (s, 3H); ^{13}**C NMR** (101 MHz, $CDCl_3$) δ 171.8, 138.7, 137.9, 128.1, 127.1, 126.8, 126.4, 126.0, 123.5, 66.5, 65.7, 27.1, 24.4.

1,3-dimethyl-3-phenyl-4-vinylazetidin-2-one (4r)

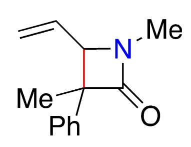

4r

Prepared according to general procedure B using **1r** (50.3 mg, 0.25 mmol) and **2-CTX** (6.2 mg, 0.03 mmol). Chromatography on silica gel with EtOAc:petroleum ether (0:1 to 1:4) afforded **4r** as 2 inseparable diastereomers (30.2 mg, 60% yield, 1.4:1 dr). **IR** (film, ν_{max}/cm^{-1}) 2969, 2921, 1742, 1494, 1423, 1389, 1251, 1177, 1067, 1028; **HRMS** (ESI^+) *m/z*: [M + H] calcd for $C_{13}H_{16}NO^+$ 202.1226; found 202.1236.

Combined diastereomers (~1.5:1): $\mathbf{R_f}$ = 0.64 (6:4 EtOAc:petroleum ether); ^{1}**H NMR** (300 MHz, $CDCl_3$) 7.41–7.15 (m, 5H), 5.96–5.79 (m, 0.6H), 5.49–5.37 (m, 1.2H), 5.33–5.20 (m, 0.4H), 5.18–5.02 (m, 0.8H), 4.04 (d, *J* = 7.7 Hz, 0.6H), 3.84 (d, *J* = 7.4 Hz, 0.4H), 2.77 (s, 3H), 1.68 (s, 1.2H), 1.43 (s, 1.8H); ^{13}**C NMR** (101 MHz, $CDCl_3$) 171.7, 171.4, 142.3, 138.4, 135.0, 133.5,

128.8, 128.4, 127.3, 127.1, 127.1, 125.9, 121.4, 120.6, 69.3, 67.7, 63.6, 62.7, 26.7, 26.7, 23.7, 19.9.

4-ethynyl-1,3-dimethyl-3-phenylazetidin-2-one (4s)

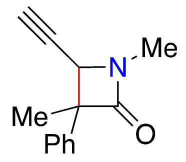

4s

Prepared according to general procedure B using **1s** (93.6 mg, 0.47 mmol) and **2-CTX** (11.6 mg, 0.05 mmol). Chromatography on silica gel with EtOAc:petroleum ether (0:1 to 1:4) afforded **4s** as 2 separable diastereomers (37.5 mg, 40% yield, 2.3:1 dr). **HRMS** (ESI$^+$) *m/z*: [M + H] calcd for $C_{13}H_{14}NO^+$ 200.1070; found 200.1085.

***trans*-Diastereomer:** 26.1 mg, $\mathbf{R_f}$ = 0.70 (3:7 EtOAc:petroleum ether); **IR** (film, ν_{max}/cm^{-1}) 3283, 3237, 2972, 2923, 1746, 1495, 1444, 1421, 1388, 1331, 1248, 1166, 1035; **^{1}H NMR** (300 MHz, $CDCl_3$) δ 7.41–7.24 (m, 5H), 4.28 (d, *J* = 2.1 Hz, 1H), 2.86 (s, 3H), 2.66 (d, *J* = 2.1 Hz, 1H), 1.67 (s, 3H); **^{13}C NMR** (101 MHz, $CDCl_3$) δ 170.7, 140.8, 128.9, 127.5, 126.0, 77.7, 76.9, 63.2, 56.1, 26.6, 21.1.

***cis*-Diastereomer:** 11.4 mg, $\mathbf{R_f}$ = 0.46 (3:7 EtOAc:petroleum ether; **IR** (film, ν_{max}/cm^{-1}) 3287, 3235, 2964, 2923, 1744, 1445, 1423, 1389, 1384, 1257, 1071, 1036; **^{1}H NMR** (300 MHz, $CDCl_3$) δ 7.41–7.28 (m, 5H), 4.16 (d, *J* = 1.8 Hz, 1H), 2.91 (s, 3H), 2.30 (d, *J* = 1.8 Hz, 1H), 1.74 (s, 3H); **^{13}C NMR** (101 MHz, $CDCl_3$) δ 170.7, 137.9, 128.3, 127.6, 127.2, 78.5, 78.0, 64.9, 57.2, 26.9, 22.9.

1,3-dimethyl-4-oxo-3-phenylazetidine-2-carbonitrile (4t)

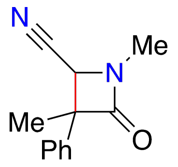

4t

Prepared according to general procedure B using **1t** (100 mg, 0.50 mmol) and **2-CTX** (12.3 mg, 0.05 mmol). Chromatography on silica gel with EtOAc:petroleum ether (1:9 to 2:8) afforded **4t** as 2 separable diastereomers (85 mg, 85% yield, 1.4:1 dr). **HRMS** (ESI$^+$) *m/z*: [M + H] calcd for $C_{12}H_{13}N_2O^+$ 201.1022; found 201.1026.

Diastereomer 1: 0.050 g; yellow oil; $\mathbf{R_f}$ = 0.48 (3:7 EtOAc:petroleum ether); **IR** (film, ν_{max}/cm^{-1}) 2973, 2934, 2540, 1753, 1655, 1448, 1424, 1383, 1033; **^{1}H NMR** (300 MHz, $CDCl_3$) δ 7.41–7.30 (m, 5H), 4.33 (s, 1H), 2.97 (s, 3H), 1.82 (s, 3H); **^{13}C NMR** (101 MHz, $CDCl_3$) δ 169.3, 138.6, 129.2, 128.3, 125.8, 115.3, 64.8, 54.5, 27.8, 21.6.

Diastereomer 2: 0.035 g; yellow solid; $\mathbf{R_f}$ = 0.18 (3:7 EtOAc:petroleum ether); **M.P.** 111–113 °C; **IR** (film, ν_{max}/cm^{-1}) 2959, 2922, 2243, 1740, 1641, 1491, 1444, 1422, 1390, 13721261, 1026;

^{1}H NMR (300 MHz, $CDCl_3$) δ 7.45–7.34 (m, 5H), 4.23 (s, 1H), 3.00 (s, 3H), 1.80 (s, 3H); **^{13}C NMR** (101 MHz, $CDCl_3$) δ 169.4, 136.1, 129.1, 128.8, 126.6, 115.1, 66.2, 55.7, 28.0, 22.5.

1-isopropyl-3,4,4-trimethyl-3-phenylazetidin-2-one (4u)

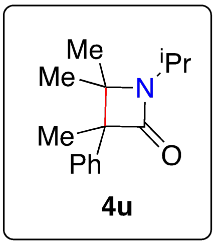

Prepared according to general procedure B using **1u** (139 mg, 0.60 mmol) and **2-CTX** (14.8 mg, 0.06 mmol). Chromatography on silica gel with EtOAc:petroleum ether (0:1 to 1:7) afforded **4u** as a clear oil (72.2 mg, 52% yield). **R_f** = 0.73 (3:7 EtOAc:petroleum ether); **IR** (film, $ν_{max}/cm^{-1}$) 2972, 2927, 1729, 1453, 1405, 1379, 1343, 1244, 1127, 1068, 1030; **^{1}H NMR** (300 MHz, $CDCl_3$) δ 7.34–7.17 (m, 5H), 3.62 (hept, *J* = 6.7 Hz, 1H), 1.58 (s, 3H), 1.45 (s, 3H), 1.34 (s, 3H), 1.36 (s,3H), 0.90 (s, 3H); **^{13}C NMR** (101 MHz, $CDCl_3$) δ 170.6, 141.1, 128.5, 127.0, 126.9, 64.1, 63.2, 43.9, 26.1, 23.1, 22.2, 22.1, 20.0; **HRMS** (ESI^+) *m/z*: [M + H] calcd for $C_{15}H_{22}NO^+$ 232.1696; found 232.1712.

12. Characterization data for β-lactams 5.

1-ethyl-3-methyl-3,4-diphenylazetidin-2-one (5a)

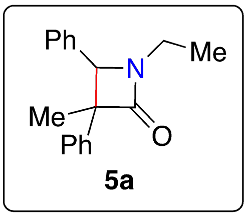

Prepared according to general procedure B using **2a** (100 mg, 0.38 mmol) and **2-CTX** (9.3 mg, 0.038 mmol). Chromatography on silica gel with EtOAc:petroleum ether (1:9 to 2:8) afforded **5a** as 2 separable diastereomers (98 mg, 98% yield, 1.4:1 dr). **HRMS** (ESI^+) *m/z*: [M + H] calcd for $C_{18}H_{20}NO^+$ 266.1539; found 266.1555.

***trans*-Diastereomer:** 0.057 g; white solid; **R_f** = 0.44 (3:7 EtOAc:petroleum ether); **M.P.** 96–98 °C; **IR** (film, $ν_{max}/cm^{-1}$) 2973, 2929, 1739, 1643, 1494, 1451, 1400, 1354, 1311, 1272, 1272, 1169, 1049; **^{1}H NMR** (300 MHz, $CDCl_3$) δ 7.51–7.27 (m, 10H), 4.80 (s, 1H), 3.78–3.70 (m 1H), 3.08–3.01 (m, 1H), 1.16 (s, 3H), 1.14 (t, *J* = 7.2 Hz, 3H); **^{13}C NMR** (151 MHz, $CDCl_3$) δ 171.9, 142.4, 136.0, 128.9, 128.9, 128.4, 127.3, 127.2, 126.0, 66.9, 62.9, 35.3, 19.5, 13.0.

***cis*-Diastereomer:** 0.041 g; colourless oil; **R_f** = 0.36 (3:7 EtOAc:petroleum ether); **IR** (film, $ν_{max}/cm^{-1}$) 2963, 2926, 1733, 1494, 1451, 1405, 1356, 1300, 1065; **^{1}H NMR** (300 MHz, $CDCl_3$) δ 7.12–7.09 (m, 3H), 7.02 (s, 5H), 6.95–6.92 (m, 2H), 4.63 (s, 1H), 3.66–3.59 (m, 1H), 3.01–2.94 (m, 1H), 1.80 (s, 3H), 1.14, (t, *J* = 7.3 Hz, 3H); **^{13}C NMR** (151 MHz, $CDCl_3$) δ 171.8, 138.4, 136.1, 128.2, 128.0, 128.0, 127.7, 127.4, 126.6, 68.5, 65.2, 35.2, 24.5, 12.9.

1-benzyl-3-methyl-3,4-diphenylazetidin-2-one (5b)

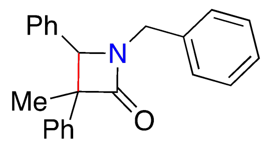

5b

Prepared according to general procedure B using 2**b** (115 mg, 0.35 mmol) and **2-CTX** (8.6 mg, 0.04 mmol). Chromatography on silica gel with EtOAc:petroleum ether (0:1 to 1:7) afforded **5b** as 2 inseparable diastereomers (95.1 mg, 83% yield, 2:1 dr). **IR** (film, ν_{max}/cm^{-1}) 3029, 2969, 2923, 1742, 1495, 1452, 1398, 1354, 1216, 1175, 1073; **HRMS** (ESI$^+$) *m/z*: [M + H] calcd for $C_{23}H_{22}NO^+$ 328.1696; found 328.1705.

Combined diastereomers: clear oil; **R$_f$** = 0.45 (2:8 EtOAc:petroleum ether); **^{1}H NMR** (300 MHz, $CDCl_3$) δ 7.47–6.91 (m, 15H), 5.06 (d, *J* = 14.9 Hz, 0.66H), 5.03 (d, *J* = 14.9 Hz, 0.33H), 4.65 (s, 0.66H), 4.44 (s, 0.33H), 4.03 (d, *J* = 14.9 Hz, 0.66H), 3.82 (d, *J* = 14.9 Hz, 0.33H), 1.77 (s, 1H), 1.23 (s, 2H); **^{13}C NMR** (101 MHz, $CDCl_3$) δ 172.1, 171.8, 142.2, 138.3, 135.8, 135.6, 135.6, 135.5, 128.9, 128.9, 128.8, 128.7, 128.5, 128.4, 128.2, 128.0, 128.0, 127.9, 127.8, 127.8, 127.4, 127.3, 127.2, 126.6, 126.0, 68.0, 67.0, 65.7, 63.4, 44.4, 44.3, 24.3, 19.5.

3-methyl-1,3,4-triphenylazetidin-2-one (5c)[16]

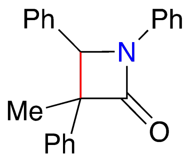

5c

Prepared according to general procedure B using **3c** (60 mg, 0.19 mmol) and **2-CTX** (4.72 mg, 0.02 mmol). Chromatography on silica gel with EtOAc:petroleum ether (1:9 to 3:7) afforded **5c** as 2 inseparable diastereomers (25 mg, 52% yield, 1:1.4 dr).

R$_f$ = 0.60 (2:8 EtOAc:petroleum ether); **IR** (film, ν_{max}/cm^{-1}) 3030, 2925, 1731, 1598, 1493, 1453, 1382, 1351, 1273, 1151, 1062, 1025; **^{1}H NMR** (300 MHz, $CDCl_3$) δ 7.57–7.54 (m, 2H), 7.43–7.29 (m, 14.25H), 7.10–6.99 (m, 10H), 5.24 (s, 1H), 5.06 (s, 0.75H), 1.92 (s, 2.25H), 1.23 (s, 3H); **^{13}C NMR** (101 MHz, $CDCl_3$) 169.4, 169.3, 142.0, 137.9, 137.7, 137.6, 135.4, 135.0, 131.1, 129.9, 129.2, 129.1, 129.0, 128.5, 128.4, 128.1, 128.1, 127.5, 127.4, 127.3, 127.1, 126.8, 126.1, 124.1, 68.8, 67.1, 64.8, 62.7, 24.6, 19.9 (overlapping peaks in the ^{13}C NMR spectrum meant that some of the signals in this diastereomeric mixture could not be reliably assigned); **HRMS** (ESI$^+$) *m/z*: [M + H] calcd for $C_{22}H_{20}NO^+$ 314.1539; found 314.1547. Reported data in accordance with previous literature.

3-methyl-3,4-diphenyl-1-tosylazetidin-2-one (5d)

Prepared according to general procedure B using **2d** (114 mg, 0.29 mmol) and **2-CTX** (7.2 mg, 0.03 mmol). Chromatography on silica gel with EtOAc:petroleum ether (0:1 to 1:7) afforded

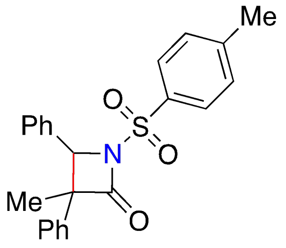

5d

5d as partially separable diastereomers (77.2 mg, 68% yield, 4.4:1 dr). **HRMS** (ESI+) *m/z*: [M + H] calcd for $C_{23}H_{22}NO_3S^+$ 392.1315; found 392.1329.

***trans*-Diastereomers (as a mixture with *cis-diastereomer*):** 24.9 mg (1.6:1 *cis*:*trans*); colourless oil; **R**$_f$ = 0.53 (2:8 EtOAc:petroleum ether); **IR** (film, ν_{max}/cm^{-1}) 3030, 2964, 2925, 1787, 1598, 1496, 1451, 1365, 1249, 1216, 1165, 1087, 1053, 1028; **^{1}H NMR** (300 MHz, $CDCl_3$) (only *trans* signals reported) δ 7.85 (d, *J* = 8.0 Hz, 2H), 7.39–7.15 (m, 13H), 5.18 (s, 1H), 2.46 (s, 3H), 1.15 (s, 3H); **^{13}C NMR** (101 MHz, $CDCl_3$) (only *trans* signals reported) δ 169.4, 145.5, 134.0, 135.7, 134.3, 130.0, 129.2, 128.8, 128.8, 127.9, 127.9, 127.0, 125.6, 69.8, 63.5, 29.8, 19.4.

***cis*-Diastereomer (completely separated):** 52.3 mg; white solid, **R**$_f$ = 0.45 (2:8 EtOAc:petroleum ether; **M.P.** 152–153 °C; **IR** (film, ν_{max}/cm^{-1}) 3061, 3032, 2965, 2926, 1786, 1597, 1496, 1451, 1364, 1247, 119, 1184, 1088, 1053, 1028; **^{1}H NMR** (300 MHz, $CDCl_3$) δ 7.76 (d, *J* = 7.9 Hz, 2H), 7.30–6.82 (m, 13H), 4.99 (s, 0.87H), 4.29 (s, 0.13H), 2.44 (s, 3H), 1.70 (s, 3H); **^{13}C NMR** (101 MHz, $CDCl_3$) δ 168.9, 145.4, 136.2, 135.5, 134.4, 130.0, 128.4, 128.2, 128.1, 127.8, 127.7, 127.3, 126.9, 70.7, 65.6, 24.6, 21.8.

13. Characterization data for β-lactams 6.

1,3-dimethyl-4-phenyl-3-(*p*-tolyl)azetidin-2-one (6a)

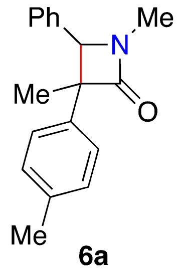

6a

Prepared according to general procedure B using **3a** (89 mg, 0.34 mmol) and **2-CTX** (8.3 mg, 0.03 mmol). Chromatography on silica gel with EtOAc:petroleum ether (1:9 to 2:8) afforded **6a** as 2 separable diastereomers (77 mg, 87% yield, 2.3:1 dr). **HRMS** (ESI+) *m/z*: [M + H] calcd for $C_{18}H_{20}NO^+$ 266.1539; found 266.1553.

***trans*-Diastereomer:** 0.054 g; colourless oil; **R**$_f$ = 0.27 (2:8 EtOAc:petroleum ether); **IR** (film, ν_{max}/cm^{-1}) 2969, 2921, 1745, 1636, 1514, 1452, 1420, 1387, 1352, 1179, 1024; **^{1}H NMR** (300 MHz, $CDCl_3$) δ 749–7.44 (m, 2H), 7.40–7.37 (m, 3H), 7.32–7.30 (m, 2H), 7.22–7.19 (m, 2H), 4.72 (s, 1H), 2.91 (s, 3H), 2.36 (s, 3H), 1.13 (s, 3H); **^{13}C NMR** (101 MHz, $CDCl_3$) δ 172.3, 139.4, 136.9, 135.8, 129.5, 128.9, 128.3, 127.2, 126.0, 69.3, 63.6, 27.1, 21.2, 19.7.

***cis*-Diastereomer:** 0.023 g; colourless oil; **R$_f$** = 0.13 (2:8 EtOAc:petroleum ether); **M.P.** 111–113 °C; **IR** (film, ν_{max}/cm^{-1}) 2959, 2921, 1744, 1633, 1452, 1420, 1390, 1361, 1280, 1260, 1067, 1026; **^{1}H NMR** (300 MHz, $CDCl_3$) δ 7.38–7.35 (m, 1H), 7.14–7.11 (m, 3H), 6.93–6.82 (m, 5H), 4.54 (s, 1H), 2.84 (s, 3H), 2.16 (s, 3H), 1.78 (s, 3H); **^{13}C NMR** (101 MHz, $CDCl_3$) δ 172.2, 136.1, 135.8, 135.3, 128.7, 128.3, 128.0, 127.6, 127.2, 70.8, 65.9, 27.1, 24.7, 21.1.

3-(4-methoxyphenyl)-1,3-dimethyl-4-phenylazetidin-2-one (6b)

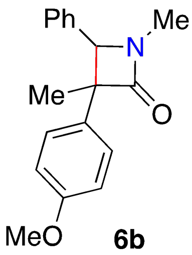

6b

Prepared according to general procedure B using **3b** (30.1 mg, 0.11 mmol) and **2-CTX** (2.6 mg, 0.01 mmol). Chromatography on silica gel with EtOAc:petroleum ether (0:1 to 1:7) afforded **6b** as 2 separable diastereomers (16.0 mg, 53% yield, 2.5:1 dr). **IR** (film, ν_{max}/cm^{-1}) 2957, 2924, 2858, 1745, 1611, 1513, 1456, 1391, 1296, 1249, 1181, 1030; **HRMS** (ESI^{+}) *m/z*: [M + H] calcd for $C_{18}H_{20}NO_2^{+}$ 282.1489; found 282.1486.

***trans*-Diastereomer:** 11.4 mg; colourless oil; **R$_f$** = 0.50 (3:7 EtOAc:petroleum ether); **^{1}H NMR** (300 MHz, $CDCl_3$) δ 7.45–7.28 (m, 7H), 6.92 (d, *J* = 9.1 Hz, 2H), 4.69 (s, 1H), 3.82 (s, 3H), 2.91 (s, 3H), 1.11 (s, 3H); **^{13}C NMR** (101 MHz, $CDCl_3$) δ 172.4, 158.8, 135.8, 134.6, 129.0, 128.4, 127.2 (2C), 114.3, 69.4, 63.3, 55.5, 27.2, 19.7.

***cis*-Diastereomer:** 4.6 mg; colourless oil; **R$_f$** = 0.39 (3:7 EtOAc:petroleum ether; **^{1}H NMR** (300 MHz, $CDCl_3$) δ 7.14–7.12 (m, 3H), 6.94–6.90 (m, 4H), 6.57 (d, *J* = 8.6 Hz, 2H), 4.53 (s, 1H), 3.67 (s, 3H), 2.84 (s, 3H), 1.77 (s, 3H); **^{13}C NMR** (101 MHz, $CDCl_3$) δ 172.1, 158.0, 135.6, 130.4, 128.3, 128.2, 127.9, 127.4, 113.2, 70.6, 65.4, 55.0, 26.9, 24.5.

3-(4-bromophenyl)-1,3-dimethyl-4-phenylazetidin-2-one (6c)

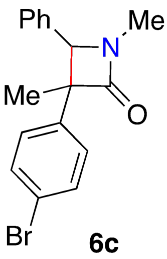

6c

Prepared according to general procedure B using **3c** (104 mg, 0.32 mmol) and **2-CTX** (7.8 mg, 0.03 mmol). Chromatography on silica gel with EtOAc:petroleum ether (0:1 to 1:7) afforded **6c** as 2 separable diastereomers (97.0 mg, 93% yield, 1.7:1 dr). **IR** (film, ν_{max}/cm^{-1}) 2956, 2923, 2857, 1750, 1644, 1490, 1455, 1422, 1392, 1076, 1012; **HRMS** (ESI^{+}) *m/z*: [M + H] calcd for $C_{17}H_{17}{}^{79}BrNO^{+}$ 330.0488; found 330.0497.

***trans*-Diastereomer:** 61.1 mg; clear oil; **R$_f$** = 0.56 (3:7 EtOAc:petroleum ether); **IR** (film, ν_{max}/cm^{-1}) 2923, 2856, 1751, 1489, 1455, 1422, 1391, 1354, 1178, 1077, 1012; **^{1}H NMR** (300

MHz, $CDCl_3$) δ 7.52–7.27 (m, 9H), 4.69 (s, 1H), 2.90 (s, 3H), 1.12 (s, 3H); **^{13}C NMR** (101 MHz, $CDCl_3$) δ 171.5, 141.4, 135.3, 132.0, 129.1, 128.6, 127.9, 127.2, 121.2, 69.0, 63.4, 27.2, 19.7.

***cis*-Diastereomer:** 35.9 mg; colourless oil; **R_f** = 0.39 (3:7 EtOAc:petroleum ether; **^{1}H NMR** (300 MHz, $CDCl_3$) δ 7.17–7.14 (m, 5H), 6.92–6.87 (d, 4H), 4.56 (s, 1H), 2.83 (s, 3H), 1.77 (s, 3H); **^{13}C NMR** (101 MHz, $CDCl_3$) δ 171.5, 137.6, 135.3, 131.1, 129.1, 128.5, 128.4, 127.5, 120.7, 70.5, 65.5, 27.1, 24.6.

3-(3,4-dichlorophenyl)-1,3-dimethyl-4-phenylazetidin-2-one (6d)

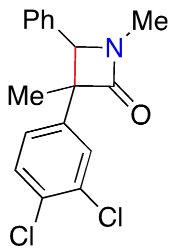

6d

Prepared according to general procedure B using **3d** (46 mg, 0.14 mmol) and **2-CTX** (3.5 mg, 0.014 mmol). Chromatography on silica gel with EtOAc:petroleum ether (1:9 to 2:8) afforded **6d** as 2 separable diastereomers (40 mg, 87% yield, 1.3:1 dr). **HRMS** (ESI$^+$) *m/z*: [M + H] calcd for $C_{17}H_{16}{}^{35}Cl_2NO^+$ 320.0603; found 320.0614.

***trans*-Diastereomer:** 0.022 g; oil; **R_f** = 0.32 (2:8 EtOAc:petroleum ether); **IR** (film, $ν_{max}/cm^{-1}$) 2971, 2923, 1747, 1643, 1461, 1423, 1386, 1354, 1134, 1026; **^{1}H NMR** (300 MHz, $CDCl_3$) δ 7.56 (s, 1H), 7.50–7.37 (m, 4H), 7.33–7.27 (m, 3H), 4.68 (s, 1H), 2.91 (s, 3H), 1.12 (s, 3H); **^{13}C NMR** (151 MHz, $CDCl_3$) δ 171.0, 142.5, 135.0, 133.1, 131.5, 130.9, 129.2, 128.8, 128.3, 127.2, 125.7, 69.0, 63.1, 27.3, 19.6.

***cis*-Diastereomer:** 0.018 g; oil; **R_f** = 0.26 (2:8 EtOAc:petroleum ether); **IR** (film, $ν_{max}/cm^{-1}$) 2964, 2924, 1745, 1643, 1468, 1422, 1389, 1136, 1028; **^{1}H NMR** (300 MHz, $CDCl_3$) δ 7.20–7.14 (m, 4H), 7.08 (d, *J* = 8.4 Hz, 1H), 6.94–6.91 (m, 2H), 6.83–6.79 (m, 1H), 4.57 (s, 1H), 2.85 (s, 3H), 1.77 (s, 3H); **^{13}C NMR** (151 MHz, $CDCl_3$) δ 170.9, 138.8, 135.1, 132.1, 130.8, 129.9, 129.5, 128.7, 128.6, 127.4, 126.8, 70.5, 65.1, 27.2, 24.5.

1,3-dimethyl-3-(naphthalen-2-yl)-4-phenylazetidin-2-one (6e)

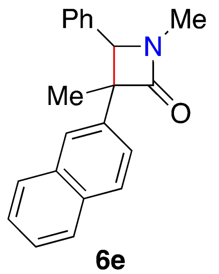

6e

Prepared according to general procedure B using **3e** (115 mg, 0.39 mmol) and **2-CTX** (9.65 mg, 0.04 mmol). Chromatography on silica gel with EtOAc:petroleum ether (1:9 to 2:8) afforded **6e** as 2 separable diastereomers (109 mg, 95% yield, 1.1:1 dr). **HRMS** (ESI$^+$) *m/z*: [M + H] calcd for $C_{21}H_{20}NO^+$ 302.1539; found 302.1548.

***trans*-Diastereomer:** 0.056 g; oil; **R_f** = 0.22 (2:8 EtOAc:petroleum ether); **IR** (film, $ν_{max}/cm^{-1}$) 2967, 2921, 1742, 1633, 1600, 1499, 1453, 1389, 1276, 1185, 1026;

^{1}H NMR (300 MHz, $CDCl_3$) δ 8.02 (s, 1H), 7.91–7.83 (m, 3H), 7.56–7.47 (m, 5H), 7.44–7.35 (m, 3H), 4.81 (s, 1H), 2.95 (s, 3H), 1.26 (s, 3H); **^{13}C NMR** (151 MHz, $CDCl_3$) δ 172.0, 139.6, 135.6, 133.5, 132.5, 129.0, 128.8, 128.4, 128.2, 127.7, 127.2, 126.5, 126.1, 124.6, 124.3, 69.1, 64.0, 27.1, 19.5.

***cis*-Diastereomer:** 0.053 g; white solid; **R_f** = 0.11 (2:8 EtOAc:petroleum ether); **M.P.** 128–130 °C; **IR** (film, ν_{max}/cm^{-1}) 2958, 2924, 1740, 1633, 1451, 1392, 1274, 1068, 1027; **^{1}H NMR** (300 MHz, $CDCl_3$) δ 7.87 (s, 1H), 7.75–7.72 (m, 1H), 7.64–7.61 (m, 1H), 7.43–7.36 (m, 3H), 7.08–7.03 (m, 3H), 6.98–6.94 (m, 2H), 6.81–6.78 (m 1H), 4.65 (s, 1H), 2.88 (s, 3H), 1.88 (s, 3H); **^{13}C NMR** (151 MHz, $CDCl_3$) δ 171.9, 135.9, 135.5, 133.0, 132.0, 128.3, 128.1, 128.1, 127.6, 127.5, 127.5, 126.1, 125.9, 125.7, 125.2, 70.6, 66.1, 27.0, 24.7.

14. References

1. Gaussian 09, Revision D.01, M. J. Frisch, G. W. Trucks, H. B. Schlegel, G. E. Scuseria, M. A. Robb, J. R. Cheeseman, G. Scalmani, V. Barone, B. Mennucci, G. A. Petersson, H. Nakatsuji, M. Caricato, X. Li, H. P. Hratchian, A. F. Izmaylov, J. Bloino, G. Zheng, J. L. Sonnenberg, M. Hada, M. Ehara, K. Toyota, R. Fukuda, J. Hasegawa, M. Ishida, T. Nakajima, Y. Honda, O. Kitao, H. Nakai, T. Vreven, J. A. Montgomery, Jr., J. E. Peralta, F. Ogliaro, M. Bearpark, J. J. Heyd, E. Brothers, K. N. Kudin, V. N. Staroverov, T. Keith, R. Kobayashi, J. Normand, K. Raghavachari, A. Rendell, J. C. Burant, S. S. Iyengar, J. Tomasi, M. Cossi, N. Rega, J. M. Millam, M. Klene, J. E. Knox, J. B. Cross, V. Bakken, C. Adamo, J. Jaramillo, R. Gomperts, R. E. Stratmann, O. Yazyev, A. J. Austin, R. Cammi, C. Pomelli, J. W. Ochterski, R. L. Martin, K. Morokuma, V. G. Zakrzewski, G. A. Voth, P. Salvador, J. J. Dannenberg, S. Dapprich, A. D. Daniels, O. Farkas, J. B. Foresman, J. V. Ortiz, J. Cioslowski, and D. J. Fox, Gaussian, Inc., Wallingford CT, 2013.
2. A. D. Becke, *J. Chem. Phys.,* 1993, **98**, 5648–5652.
3. B. Lee, W. Yang, R. G. Parr, *Phys. Rev., B*, 1988, **37**, 785–789.
4. S. H. Vosko, L. Wilk, M. Nusair, *Can. J. Phys.,* 1988, **58**, 1200–1211.
5. P. J. Stephens, F. J. Devlin, C. F. Chabalowski, M. J. Frisch, *J.Phys.Chem.,* 1994, **98**, 11623–11627.
6. F. Weigend, R. Ahlrichs, *Phys. Chem. Chem. Phys.,* 2005, **7**, 3297–3305.
7. S. Grimme, S. Ehrlich and L. Goerigk, *J. Comp. Chem*., 2011, **32***,* 1456–65.

8. For a review on continuum solvation models, see: J. Tomasi, B. Mennucci, and R. Cammi, *Chem. Rev.*, 2005, **105**, 2999–3093.
9. Orca 5.0.2: a) F. Neese, *WIREs Comput. Mol. Sci.*, 2012, **2**: 73–78; b) F. Neese, *WIREs Comput. Mol. Sci*., 2018, **8**, e1327; c) F. Neese, *WIREs Comput. Mol. Sci*., 2022, e1606, DOI: 10.1002/wcms.1606.
10. J. N. Harvey, M. Aschi, H. Schwarz, W. Koch, *Theor. Chem. Acc.*, 1998, **99**, 95–99.
    V. Barone, M. Cossi, *J. Phys. Chem., A*, 1998, **102**, 1995-2001.
11. B. A. Sandoval, P. D. Clayman, D. G. Oblinsky, S. Oh, Y. Nakano, M. Bird, G. D. Scholes and T. K. Hyster, *J. Am. Chem. Soc.,* 2021, **143**, 1735–1739.
12. Q.-L. Yang, Y.-K. Xing, X.-Y. Wang, H.-X. Ma, X.-J. Weng, X. Yang, H.-M. Guo and T.-S. Mei, *J. Am. Chem. Soc.,* 2019, **141**, 18970–18976.
13. J. H. Reed, P. A. Donets, S. Miaskiewicz and N. Cramer, *Angew. Chem. Int. Ed.* 2019, **58**, 8893–8897.
14. X.-Y. Ye, Z.-Q. Liang, C. Jing, Q.-W. Lang, Q.-D. Chen, X. Zhang, *Chem. Commun.,* 2021, **57**, 195–198.
15. Z.-Y. Dai, Z.-S. Nong, S. Song, P.-S. Wang, *Org. Lett.,* 2021, **23**, 3157–3161.
16. S. Schunk, D. Enders, *J. Org. Chem.*, 2002, **67**, 8034–8042.

15. NMR Spectra.

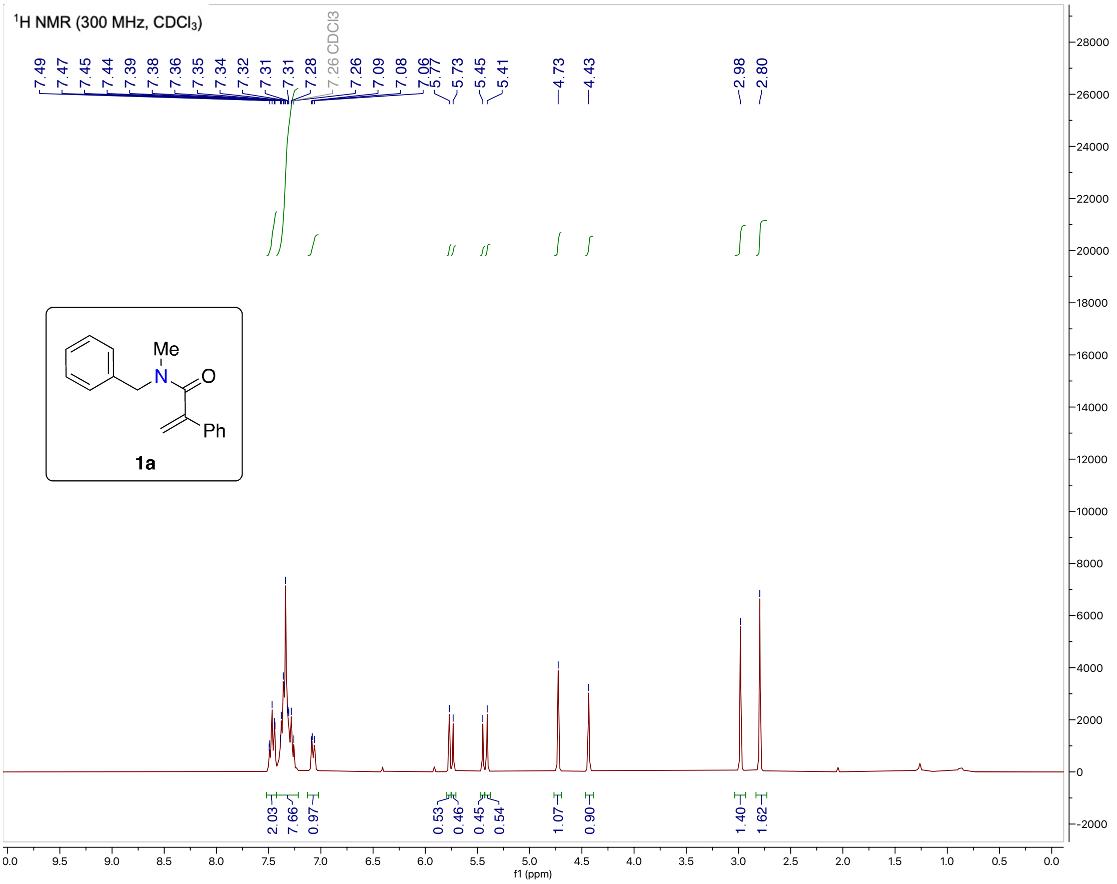

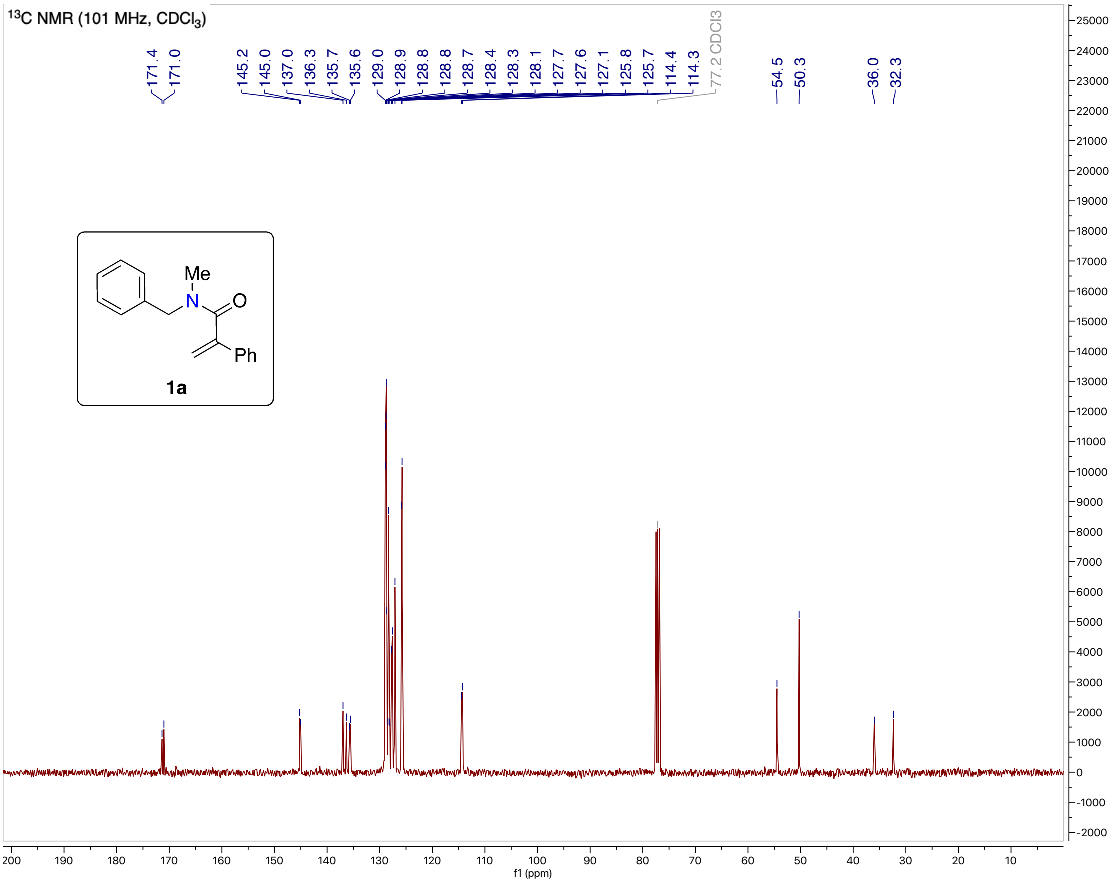

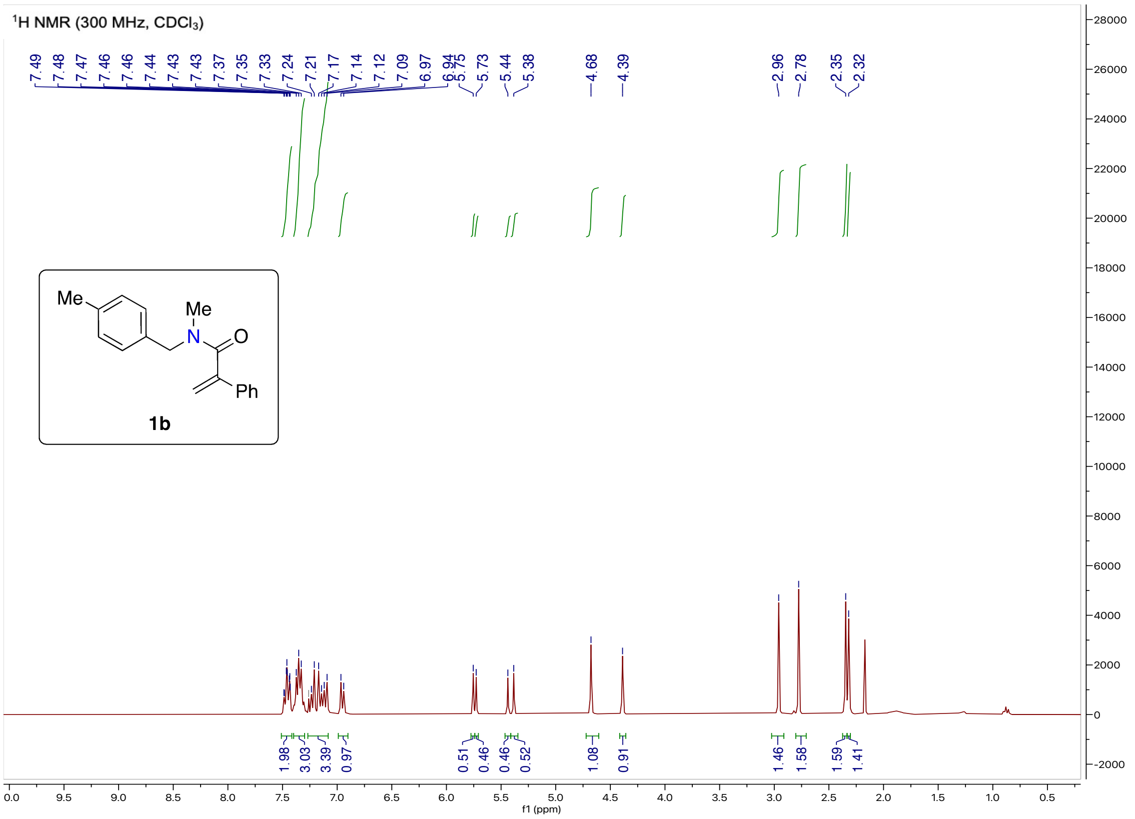

^{1}H NMR (300 MHz, CDCl$_3$)
Me
Me
N
O
Ph
1b
f1 (ppm)

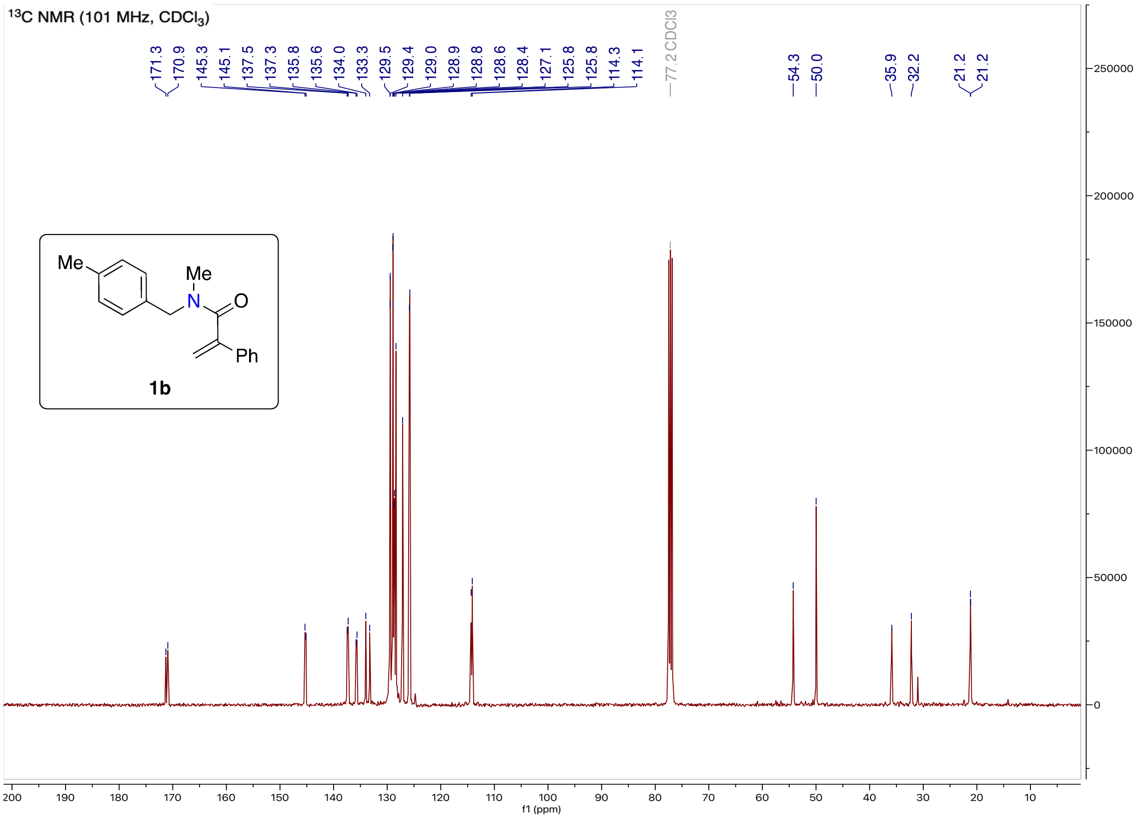

^{13}C NMR (101 MHz, CDCl$_3$)
77.2 CDCl3
Me
Me
N
O
Ph
1b
f1 (ppm)

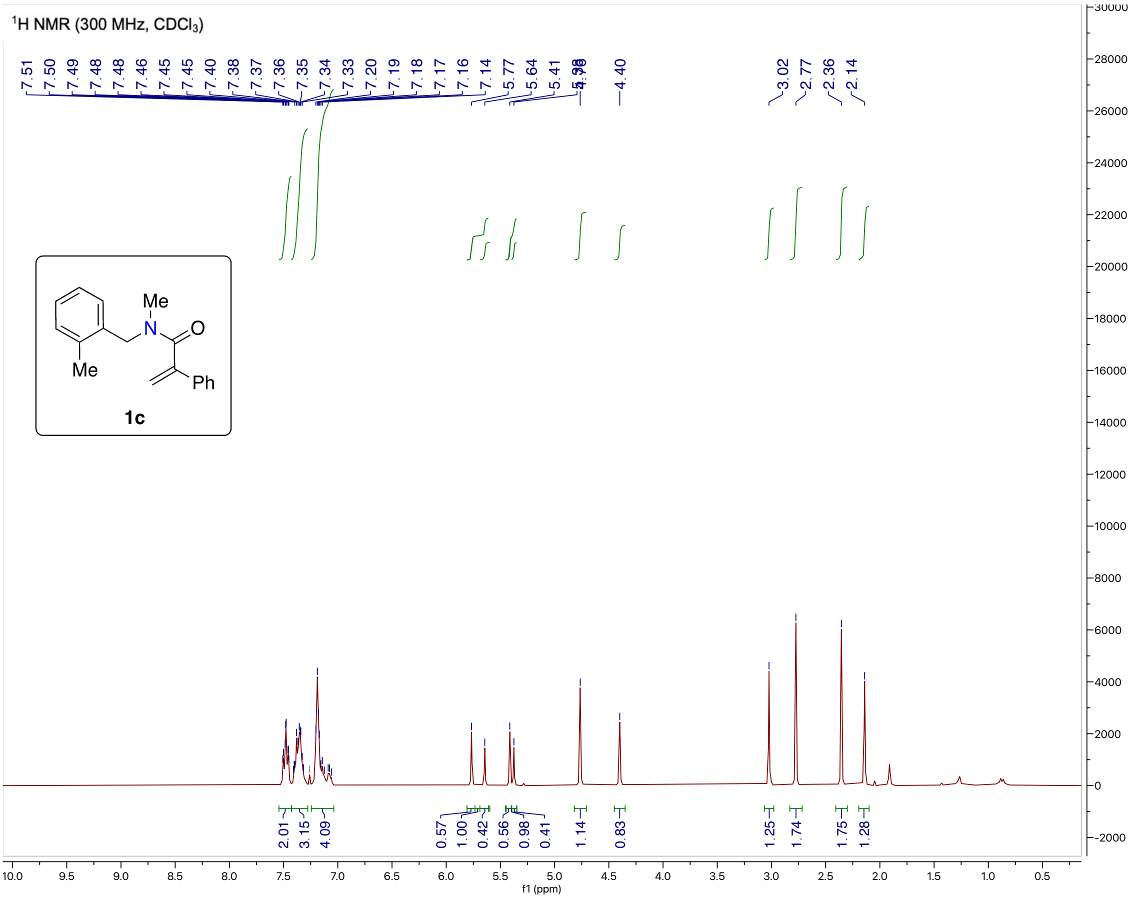

1H NMR (300 MHz, CDCl3)
7.51 7.50 7.49 7.48 7.48 7.46 7.45 7.45 7.40 7.38 7.37 7.36 7.35 7.34 7.33 7.20 7.19 7.18 7.17 7.16 7.14 5.77 5.64 5.41 4.40
3.02 2.77 2.36 2.14
2.01 3.15 4.09 0.57 1.00 0.42 0.56 0.98 0.41 1.14 0.83 1.25 1.74 1.75 1.28
Me
N
O
Me
Ph
1c
f1 (ppm)

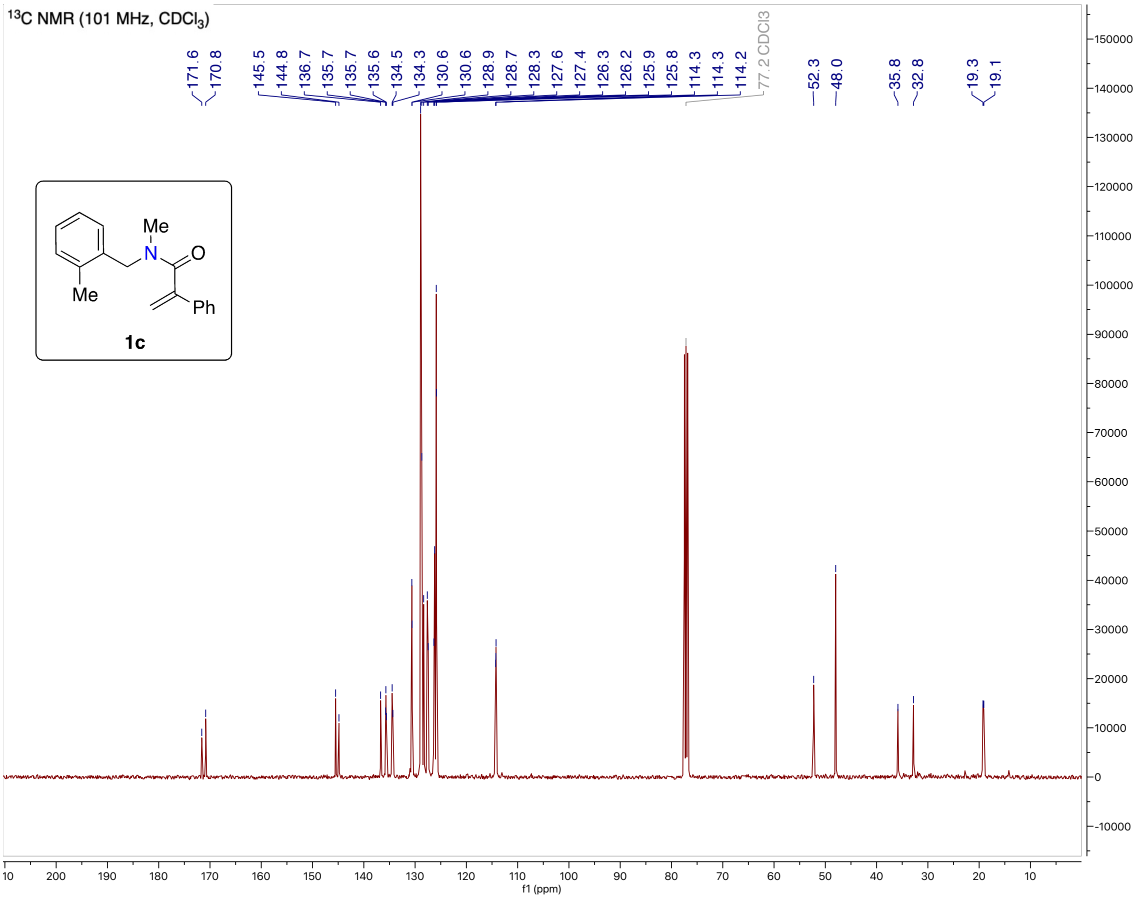

13C NMR (101 MHz, CDCl3)
171.6 170.8 145.5 144.8 136.7 135.7 135.6 134.5 134.3 130.6 130.6 128.9 128.7 128.3 127.6 127.4 126.3 126.2 125.9 125.8 114.3 114.3 114.2
77.2 CDCl3
52.3 48.0 35.8 32.8 19.3 19.1
Me
N
O
Me
Ph
1c
f1 (ppm)

^1H NMR (300 MHz, $CDCl_3$)

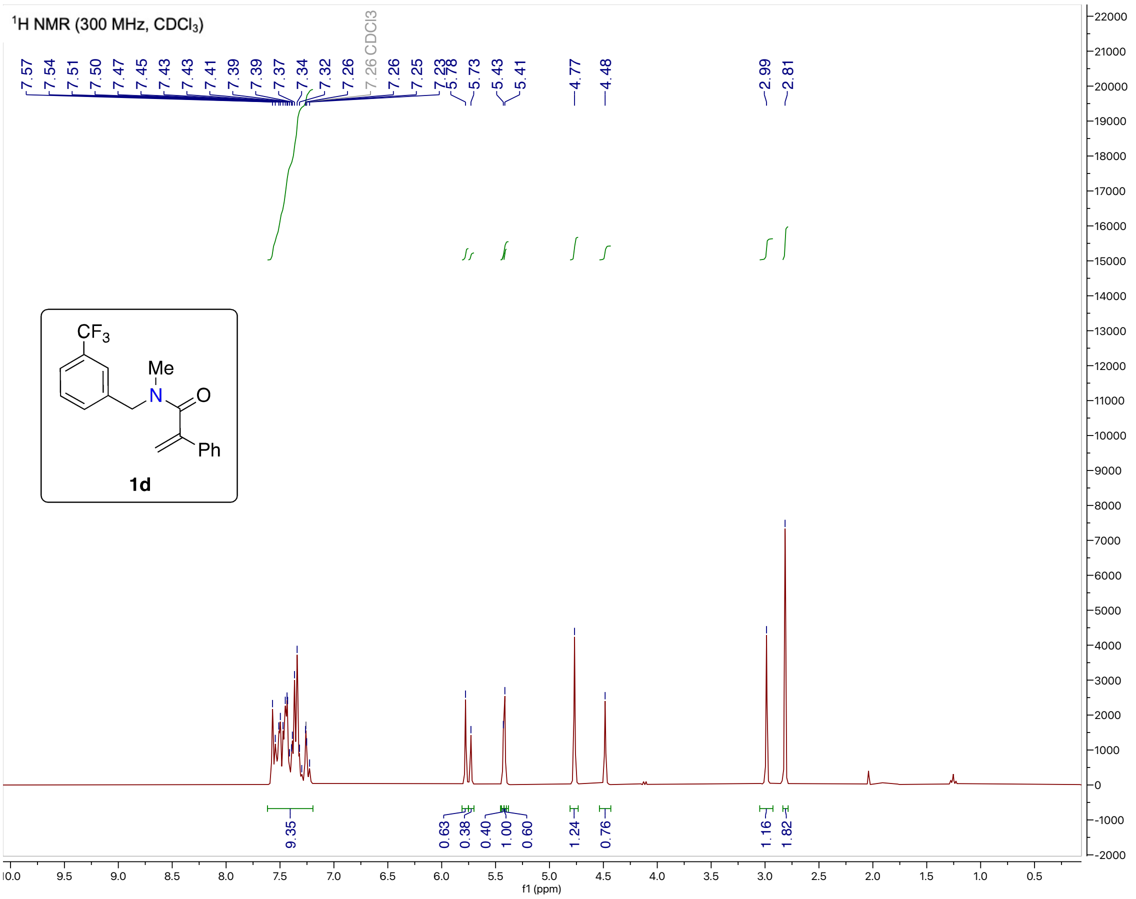

^{13}C NMR (151 MHz, $CDCl_3$)

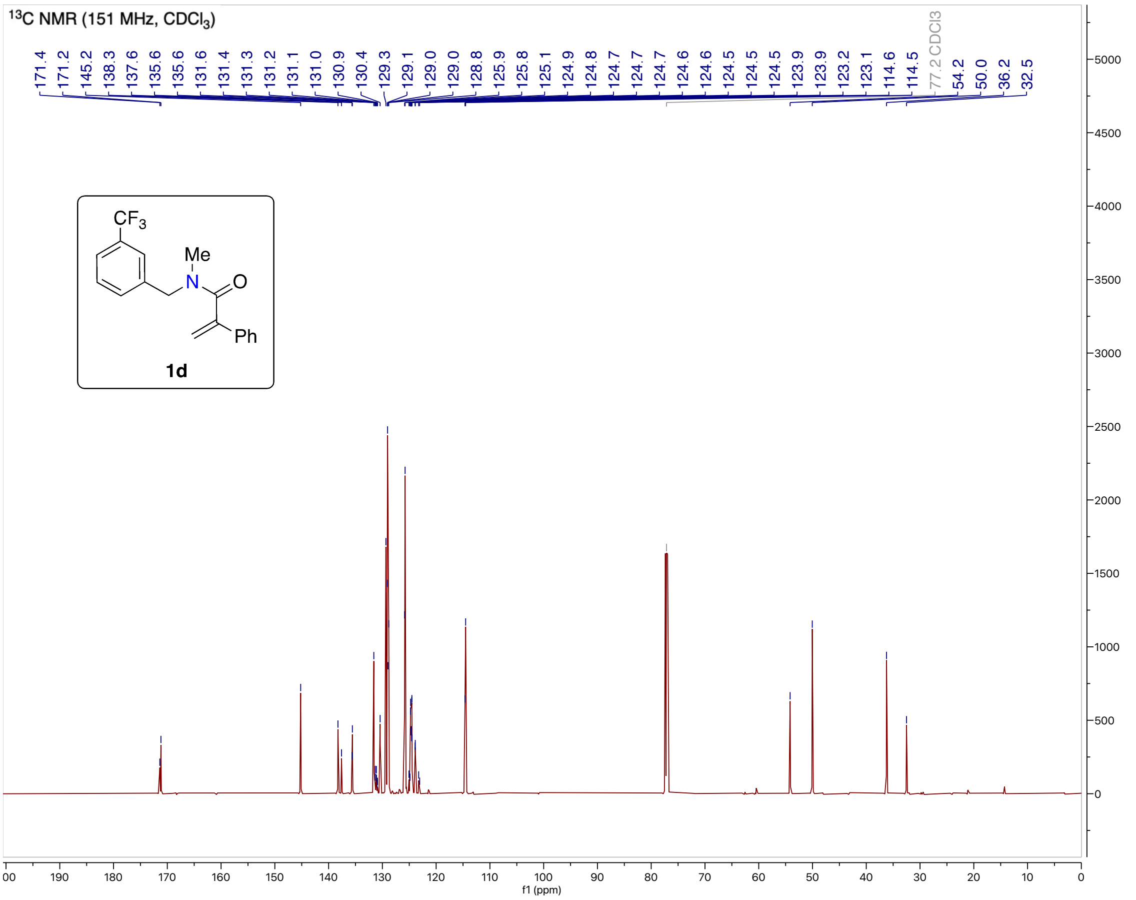

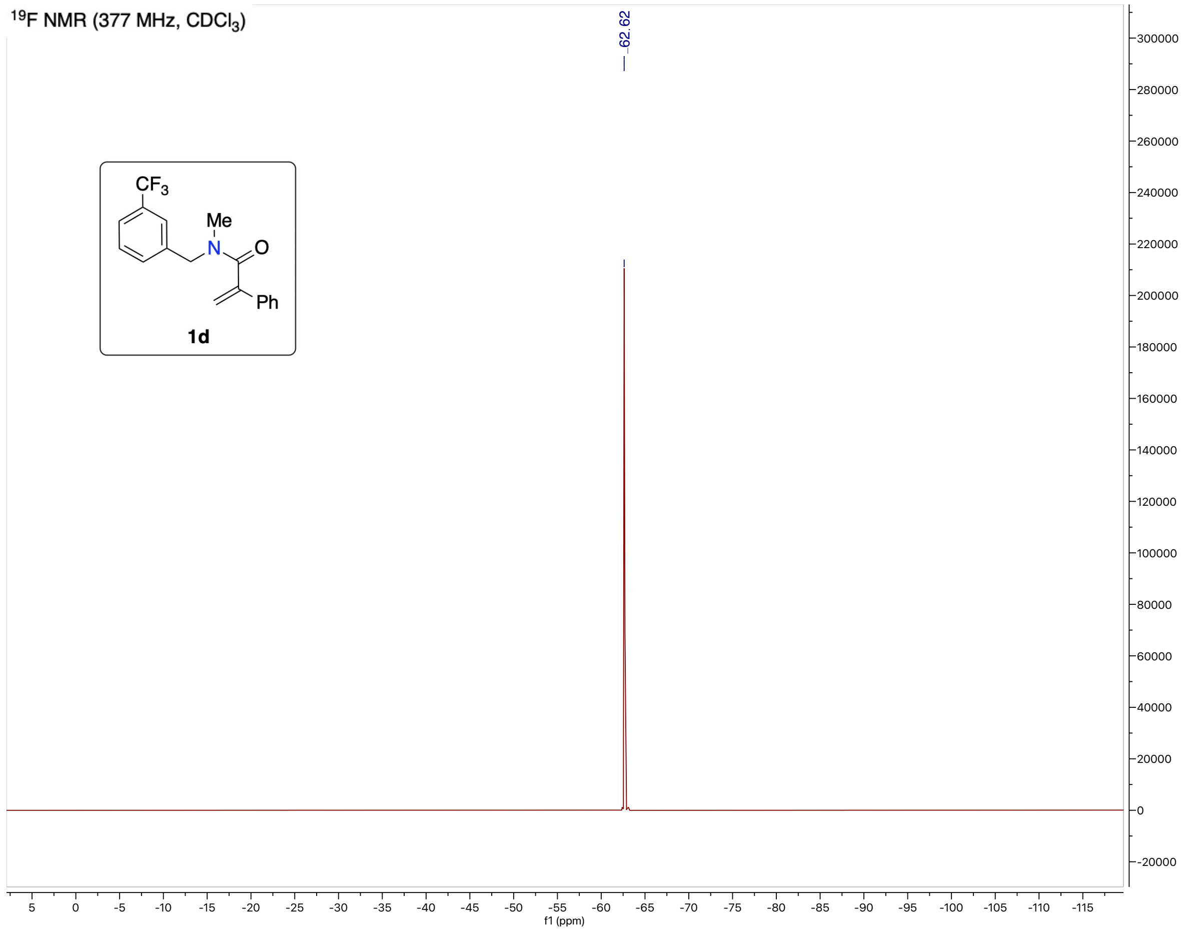

^{19}F NMR (377 MHz, CDCl$_3$)
-62.62
CF$_3$
Me
N
O
Ph
1d
f1 (ppm)

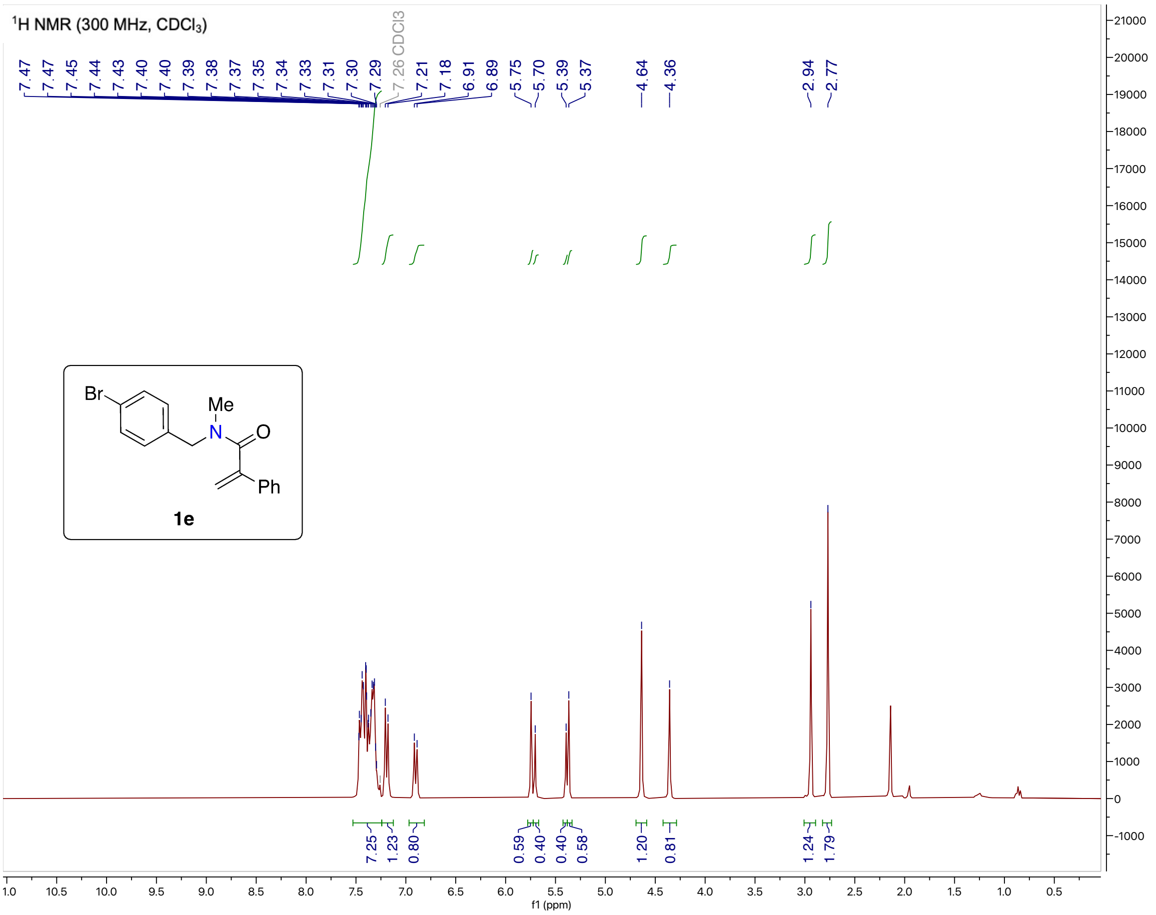
^{1}H NMR (300 MHz, CDCl$_3$)
1e
Br
Me
N
O
Ph
f1 (ppm)

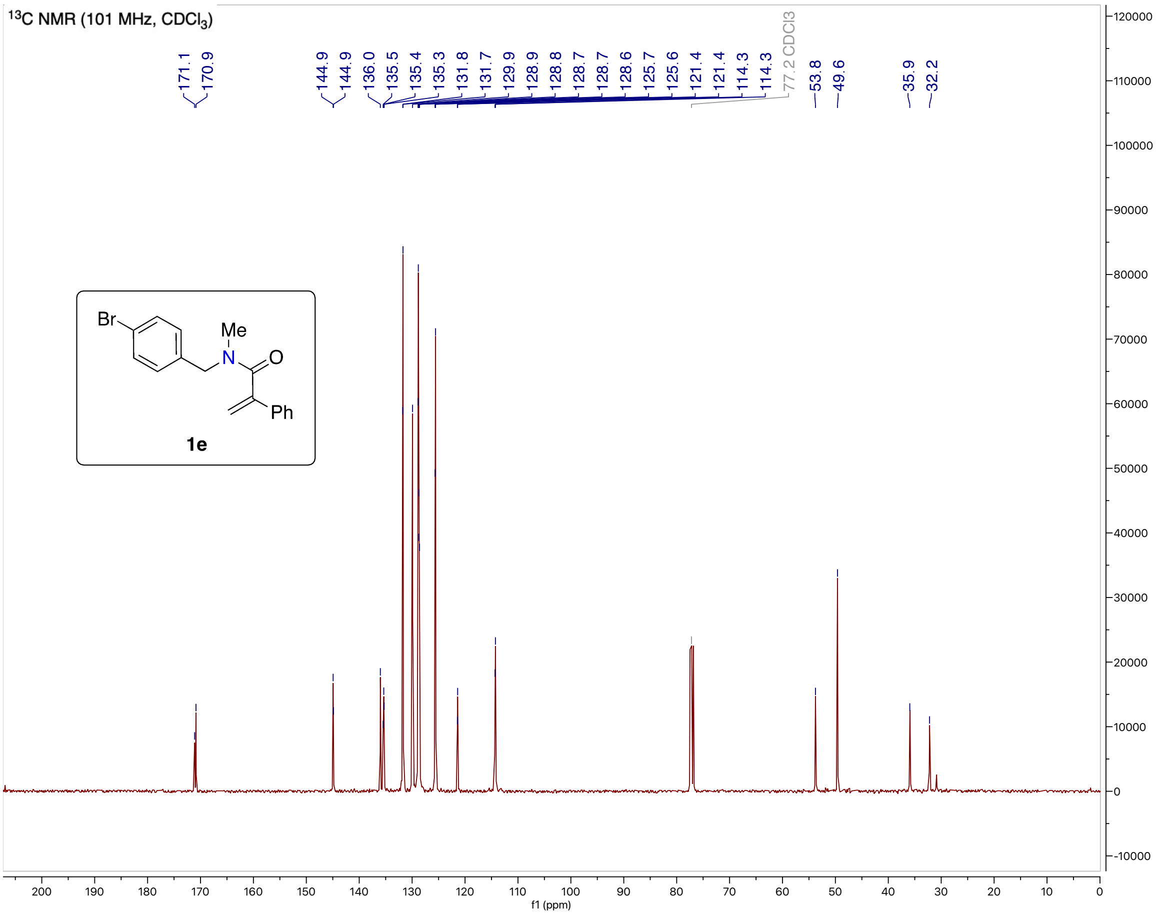
^{13}C NMR (101 MHz, CDCl$_3$)
1e
Br
Me
N
O
Ph
f1 (ppm)

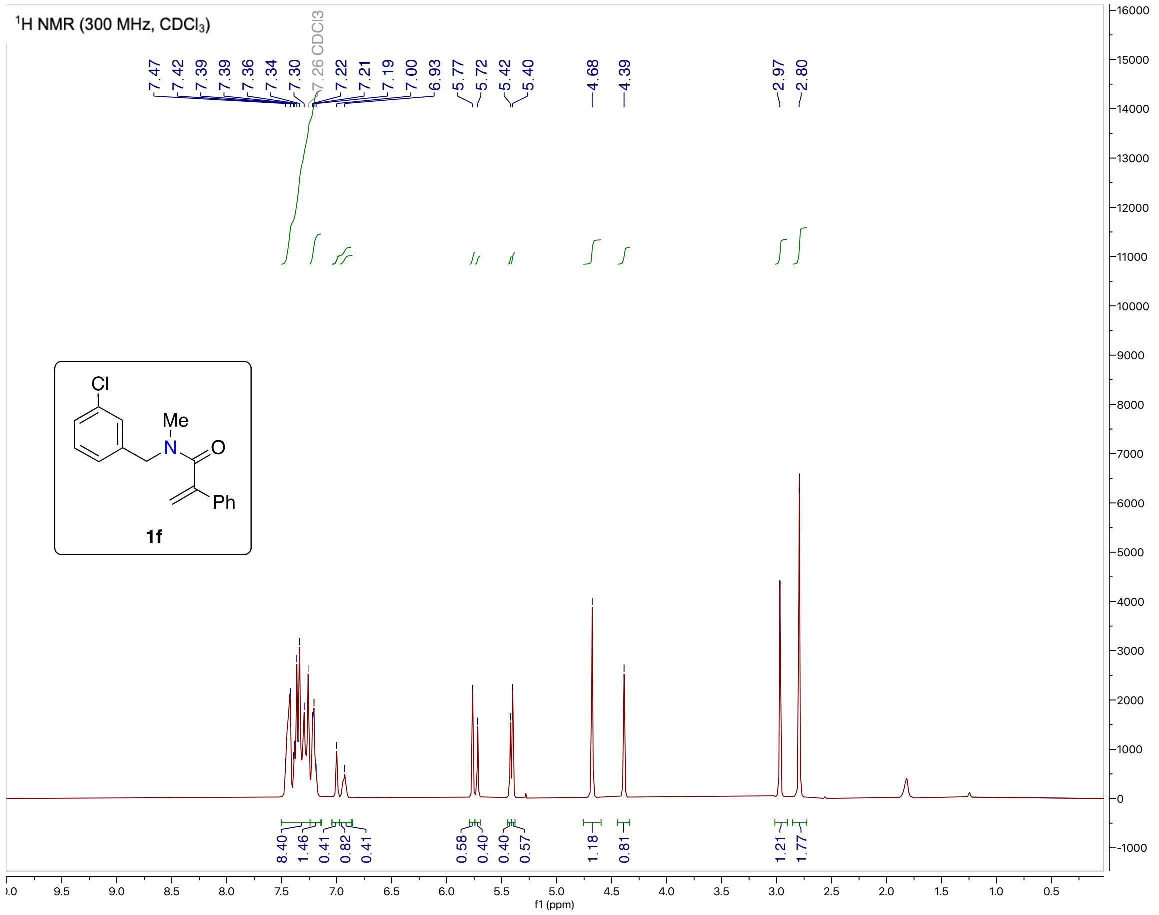

^{1}H NMR (300 MHz, $CDCl_3$)
7.47
7.42
7.39
7.39
7.36
7.34
7.30
7.26 CDCl3
7.22
7.21
7.19
7.00
6.93
5.77
5.72
5.42
5.40
4.68
4.39
2.97
2.80
8.40
1.46
0.41
0.82
0.41
0.58
0.40
0.40
0.57
1.18
0.81
1.21
1.77
Cl
Me
N
O
Ph
1f
f1 (ppm)

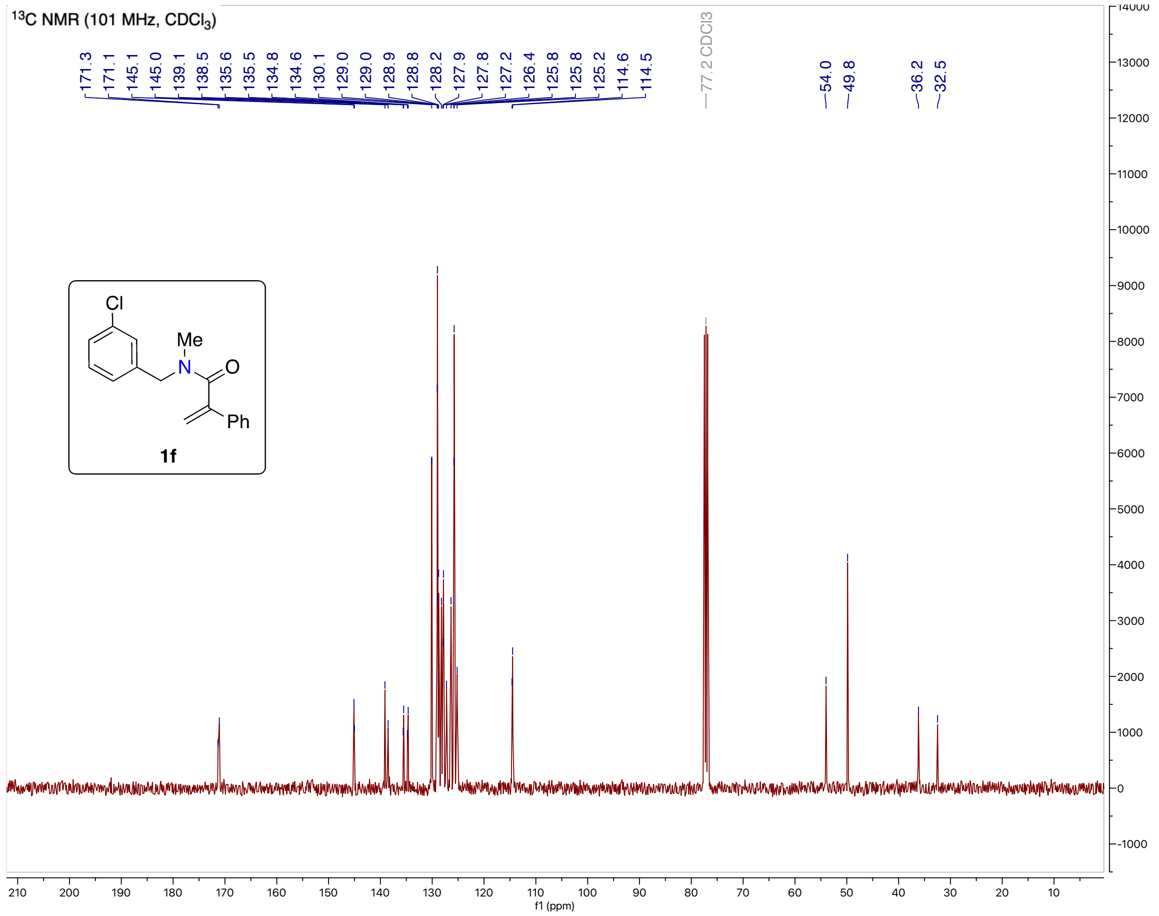

^{13}C NMR (101 MHz, $CDCl_3$)
171.3
171.1
145.1
145.0
139.1
138.5
135.6
135.5
134.8
134.6
130.1
129.0
129.0
128.9
128.8
128.2
127.9
127.8
127.2
126.4
125.8
125.8
125.2
114.6
114.5
77.2 CDCl3
54.0
49.8
36.2
32.5
Cl
Me
N
O
Ph
1f
f1 (ppm)

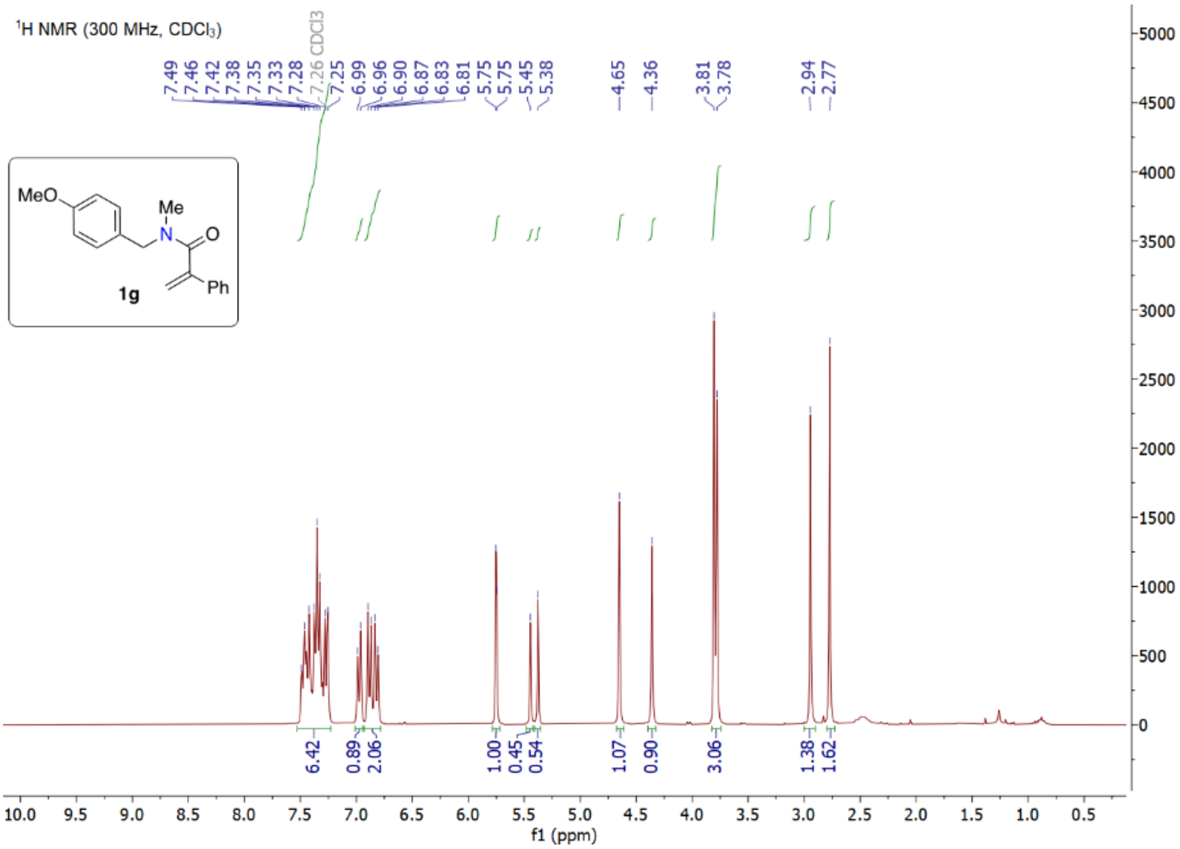

1H NMR (300 MHz, CDCl3)
7.49 7.46 7.42 7.38 7.35 7.33 7.28 7.26 CDCl3 7.25 6.99 6.96 6.90 6.87 6.83 6.81 5.75 5.75 5.45 5.38 4.65 4.36 3.81 3.78 2.94 2.77
MeO
Me
N
O
Ph
1g
6.42 0.89 2.06 1.00 0.45 0.54 1.07 0.90 3.06 1.38 1.62
f1 (ppm)

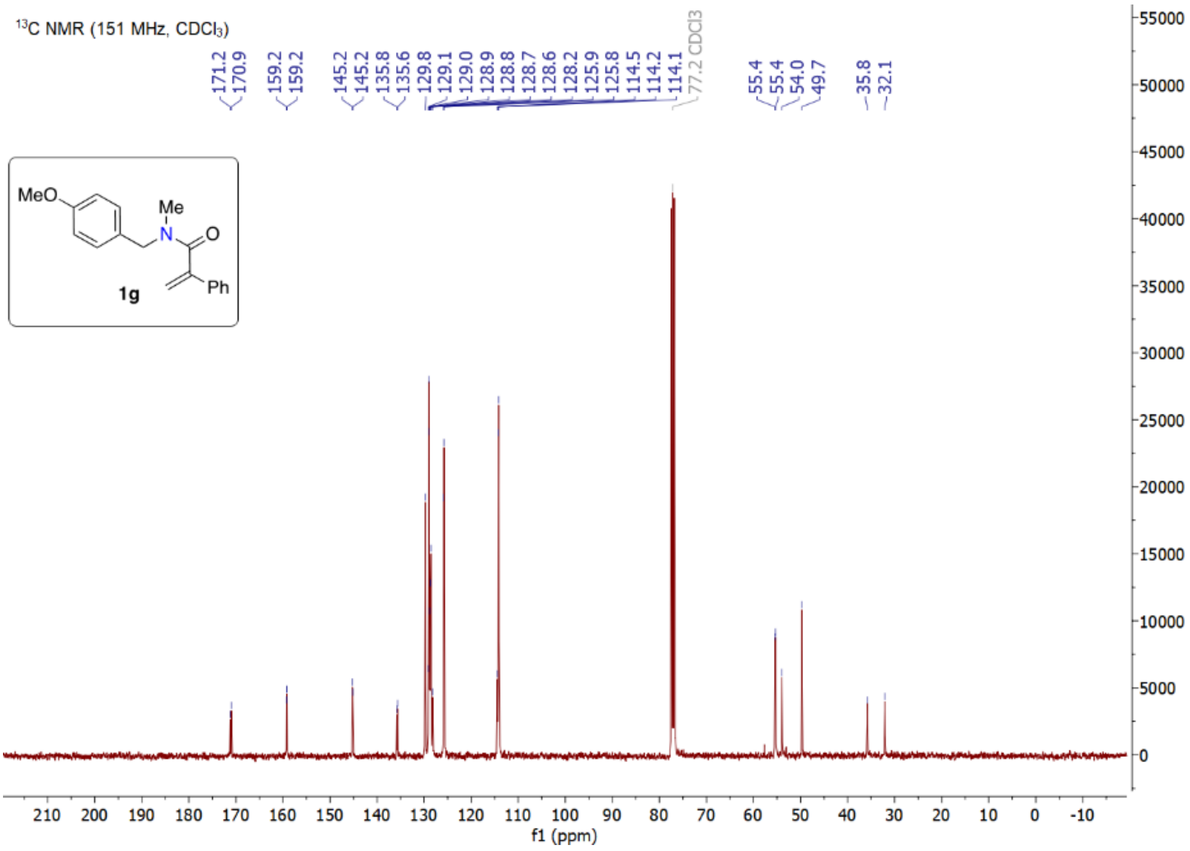

13C NMR (151 MHz, CDCl3)
171.2 170.9 159.2 159.2 145.2 145.2 135.8 135.6 129.8 129.1 129.0 128.9 128.8 128.7 128.6 128.2 125.9 125.8 114.5 114.2 114.1 77.2 CDCl3 55.4 55.4 54.0 49.7 35.8 32.1
MeO
Me
N
O
Ph
1g
f1 (ppm)

^{1}H NMR (300 MHz, $CDCl_3$)

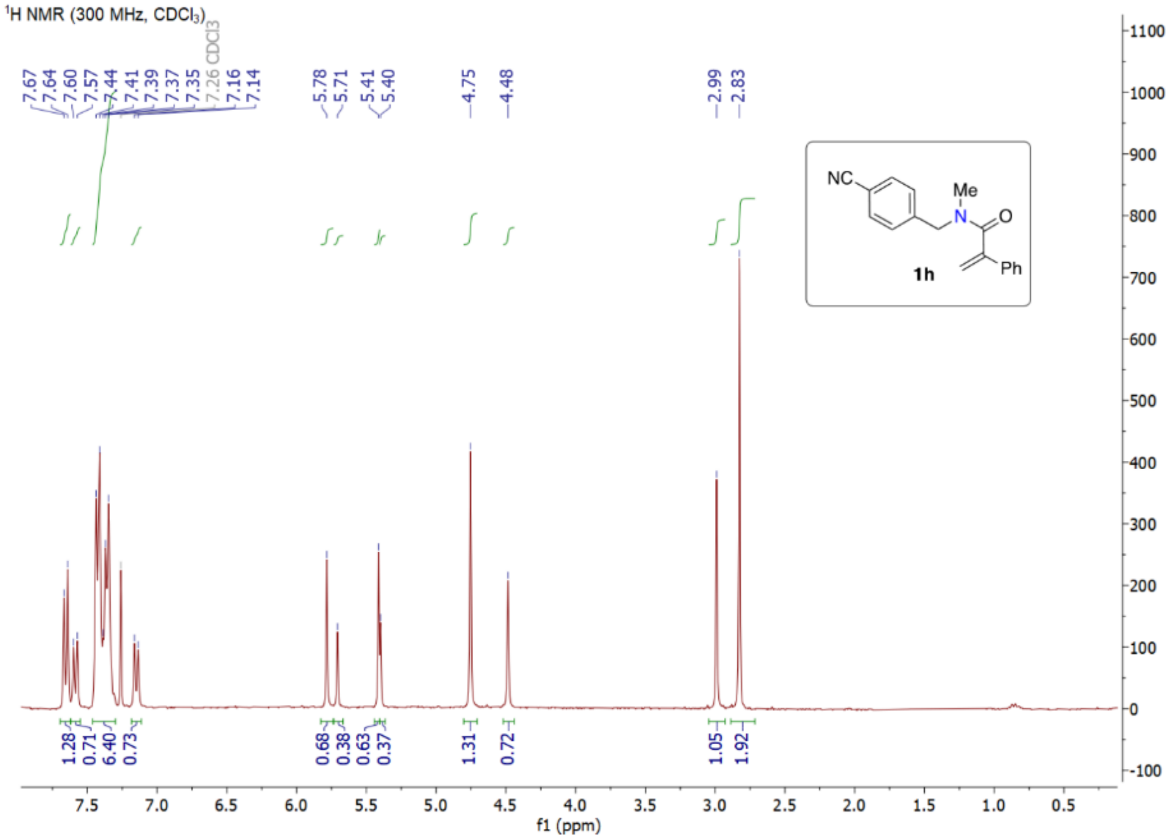

^{13}C NMR (151 MHz, $CDCl_3$)

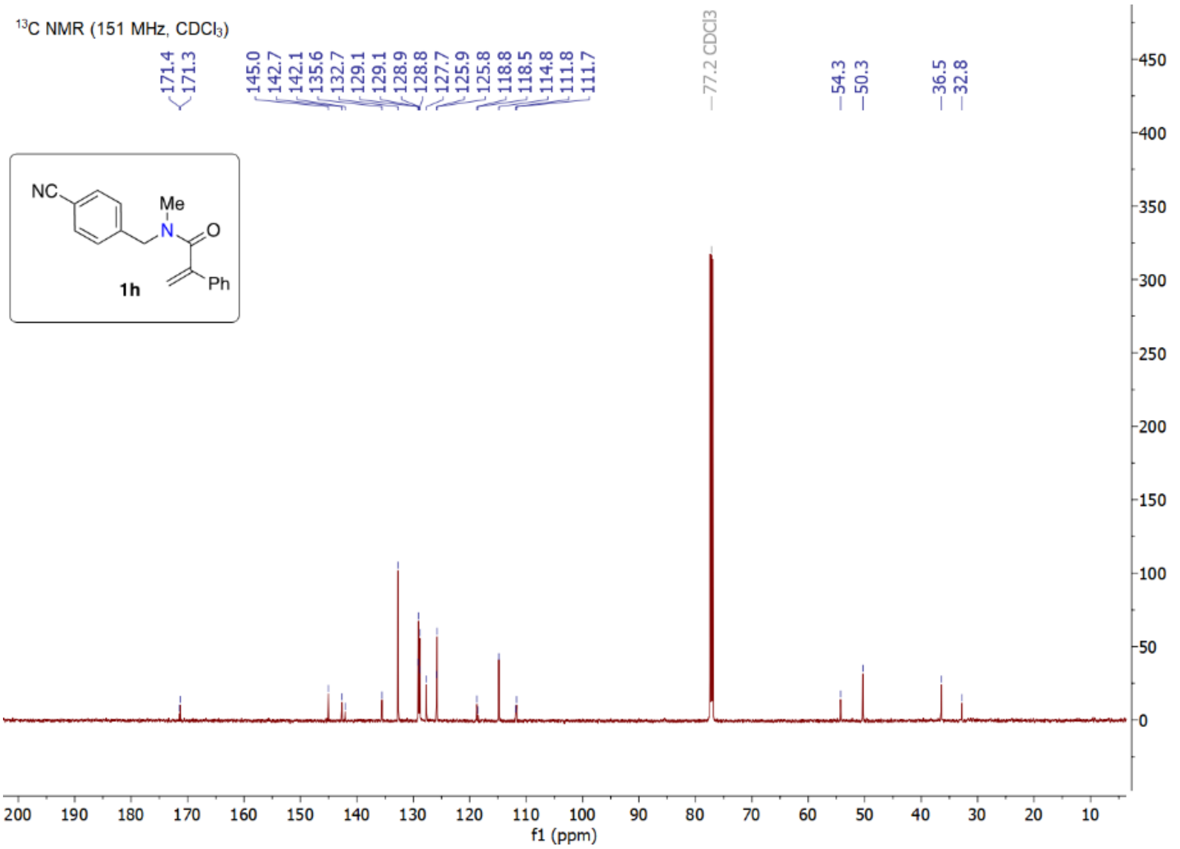

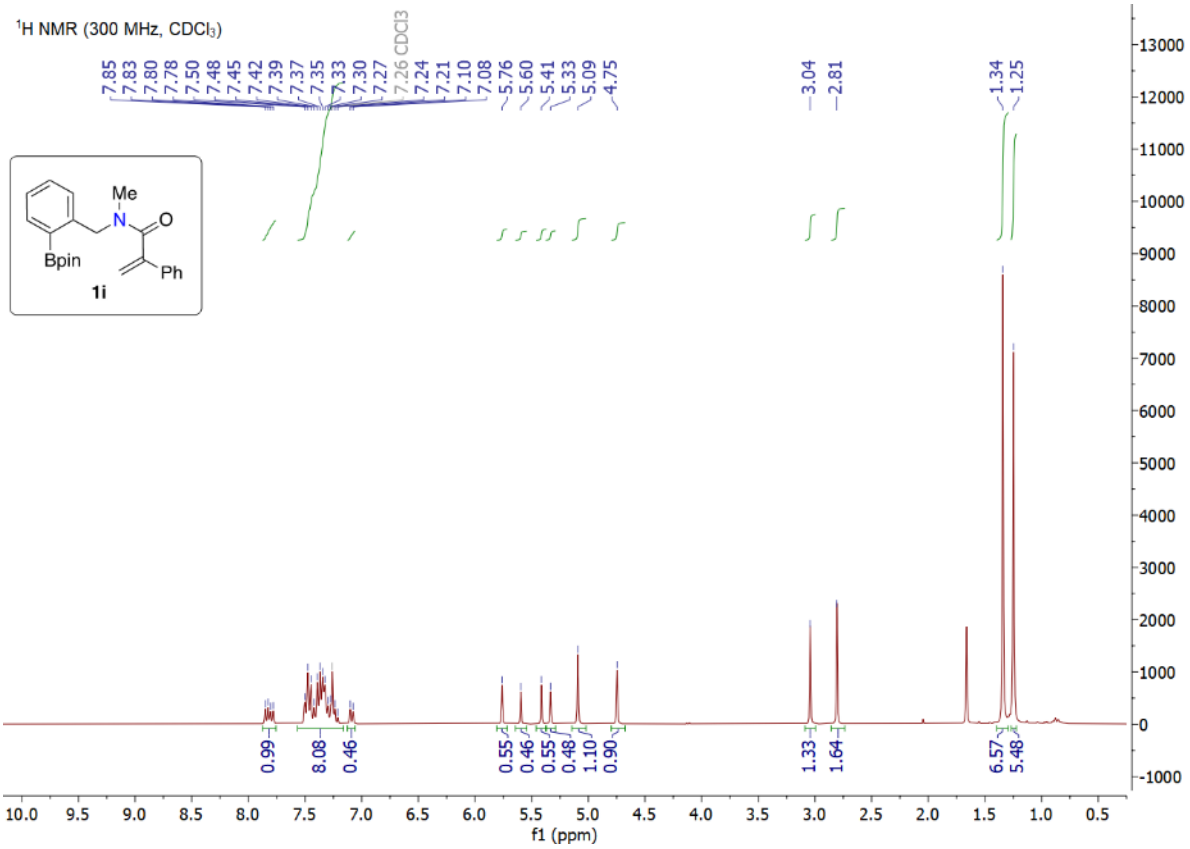

^{1}H NMR (300 MHz, CDCl$_3$)
7.85 7.83 7.80 7.78 7.50 7.48 7.45 7.42 7.39 7.37 7.35 7.33 7.30 7.27 7.26 CDCl3 7.24 7.21 7.10 7.08 5.76 5.60 5.41 5.33 5.09 4.75
3.04 2.81
1.34 1.25
Me
N
O
Bpin
Ph
1i
0.99 8.08 0.46 0.55 0.46 0.55 0.48 1.10 0.90 1.33 1.64 6.57 5.48
f1 (ppm)

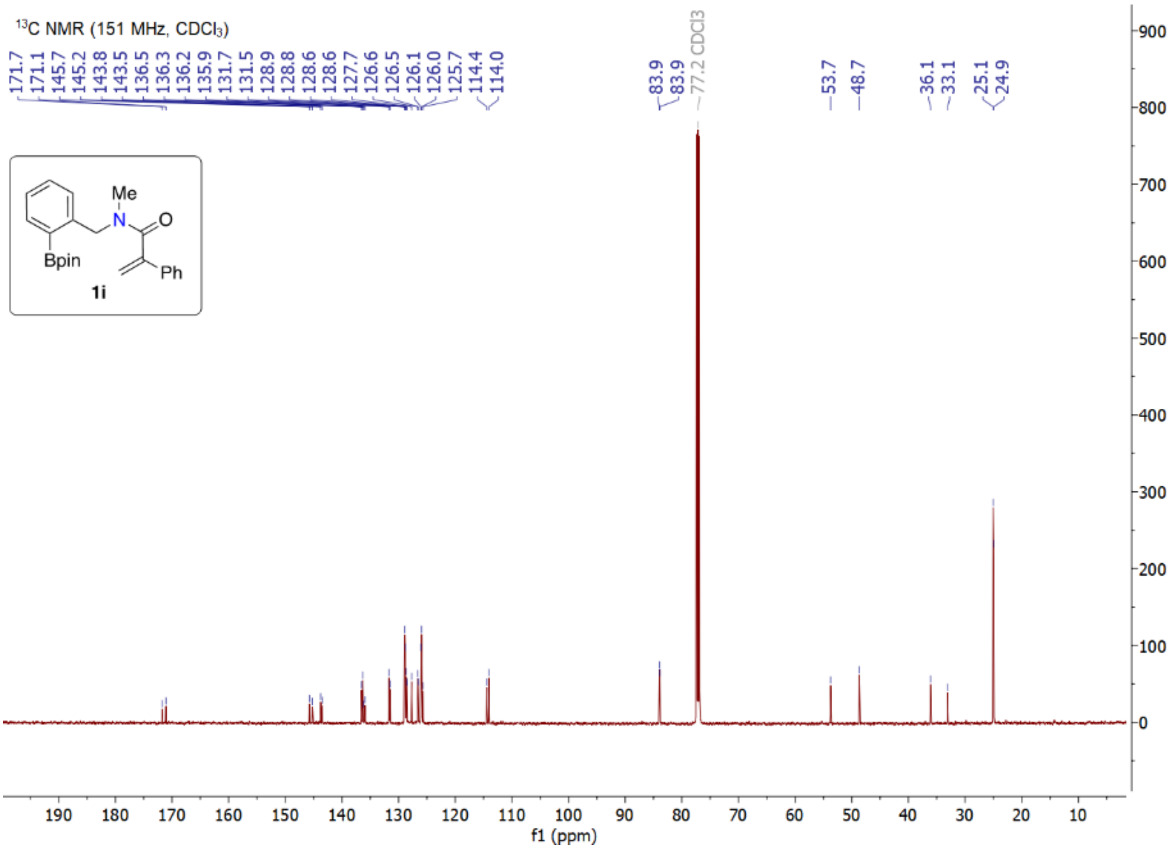

^{13}C NMR (151 MHz, CDCl$_3$)
171.7 171.1 145.7 145.2 143.8 143.5 136.5 136.3 136.2 135.9 131.7 131.5 128.9 128.8 128.6 128.6 127.7 126.6 126.5 126.1 126.0 125.7 114.4 114.0
83.9 83.9 77.2 CDCl3
53.7 48.7 36.1 33.1 25.1 24.9
Me
N
O
Bpin
Ph
1i
f1 (ppm)

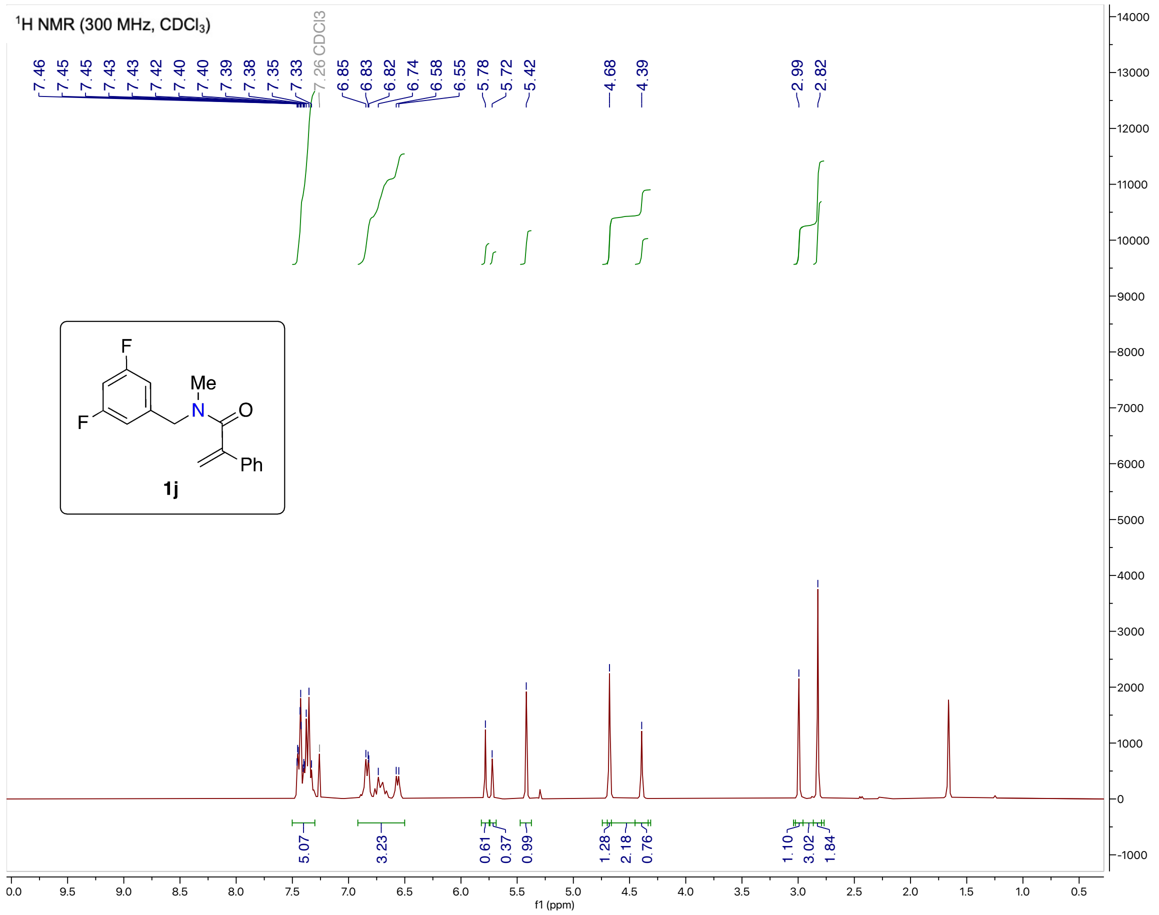

1H NMR (300 MHz, CDCl3)
1j
f1 (ppm)

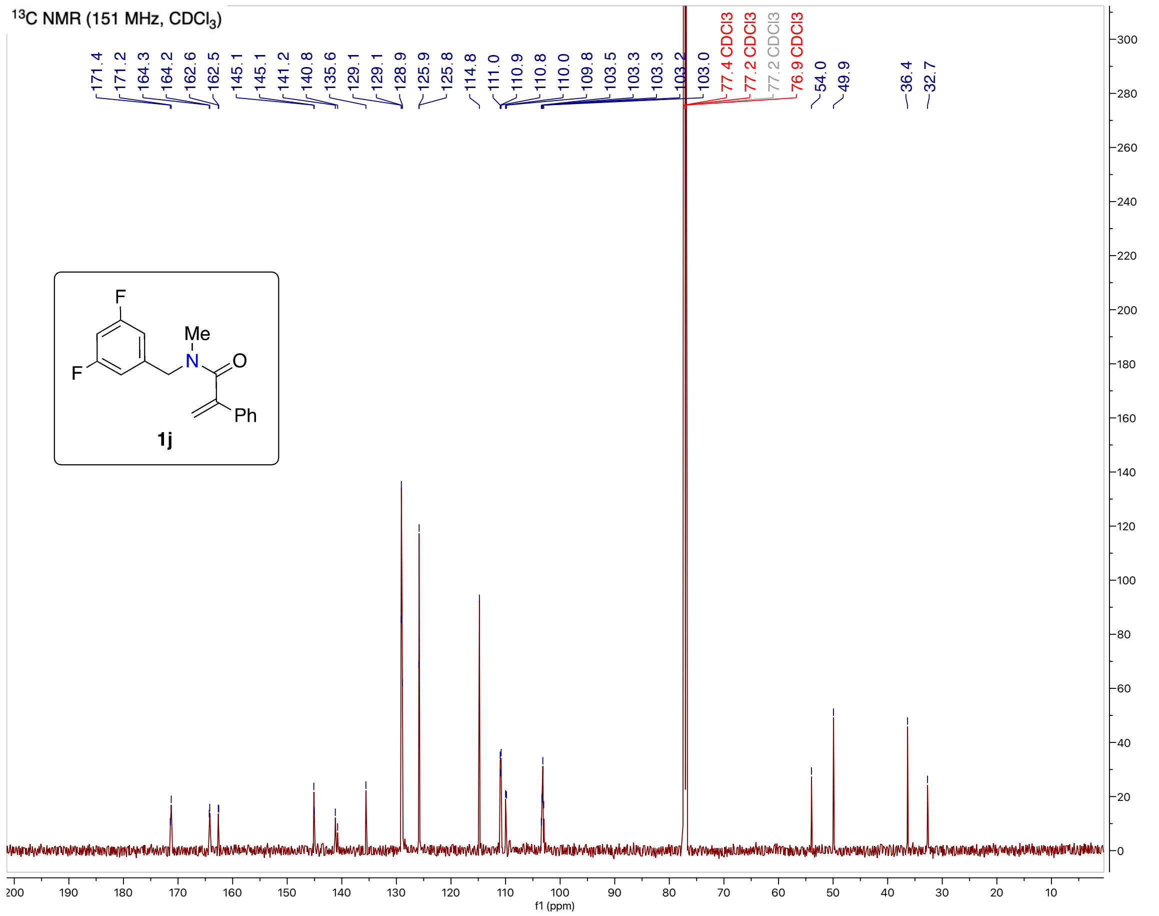

13C NMR (151 MHz, CDCl3)
1j
f1 (ppm)

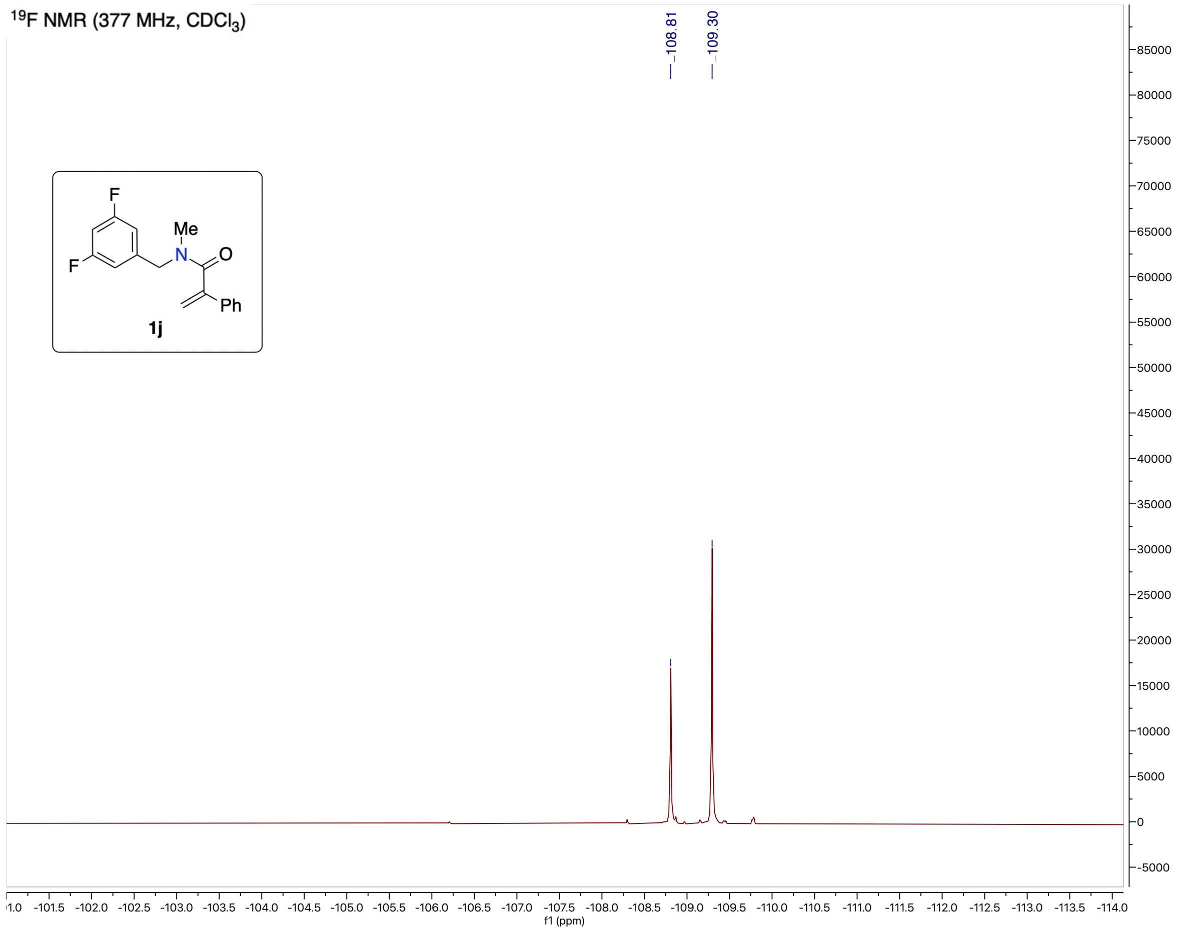

^{19}F NMR (377 MHz, CDCl$_3$)
-108.81
-109.30
F
F
Me
N
O
Ph
1j
f1 (ppm)

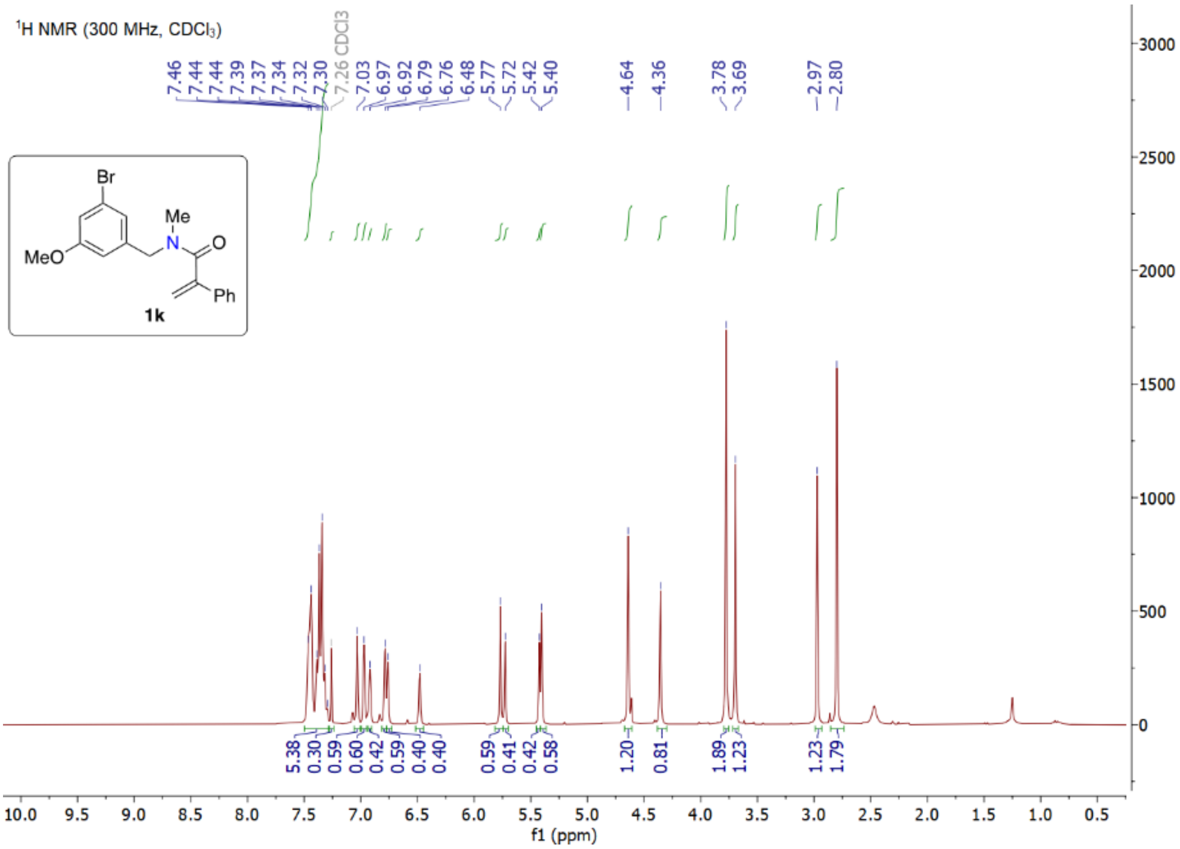

1H NMR (300 MHz, CDCl3)
7.46 7.44 7.44 7.39 7.37 7.34 7.32 7.30 7.26 CDCl3 7.03 6.97 6.92 6.79 6.76 6.48 5.77 5.72 5.42 5.40 4.64 4.36 3.78 3.69 2.97 2.80
Br
Me
N
O
MeO
Ph
1k
5.38 0.30 0.59 0.60 0.42 0.59 0.40 0.40 0.59 0.41 0.42 0.58 1.20 0.81 1.89 1.23 1.23 1.79
f1 (ppm)

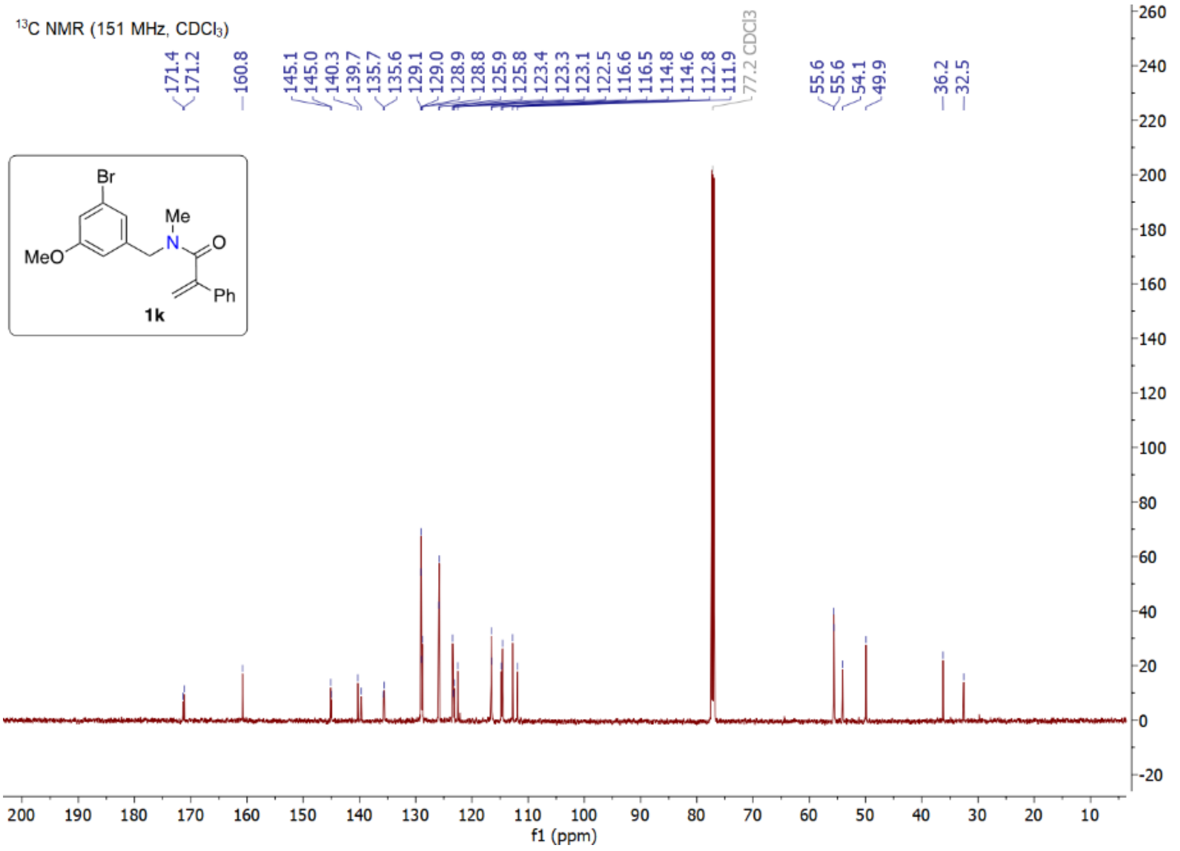

13C NMR (151 MHz, CDCl3)
171.4 171.2 160.8 145.1 145.0 140.3 139.7 135.7 135.6 129.1 129.0 128.9 128.8 125.9 125.8 123.4 123.3 123.1 122.5 116.6 116.5 114.8 114.6 112.8 111.9 77.2 CDCl3 55.6 55.6 54.1 49.9 36.2 32.5
Br
Me
N
O
MeO
Ph
1k
f1 (ppm)

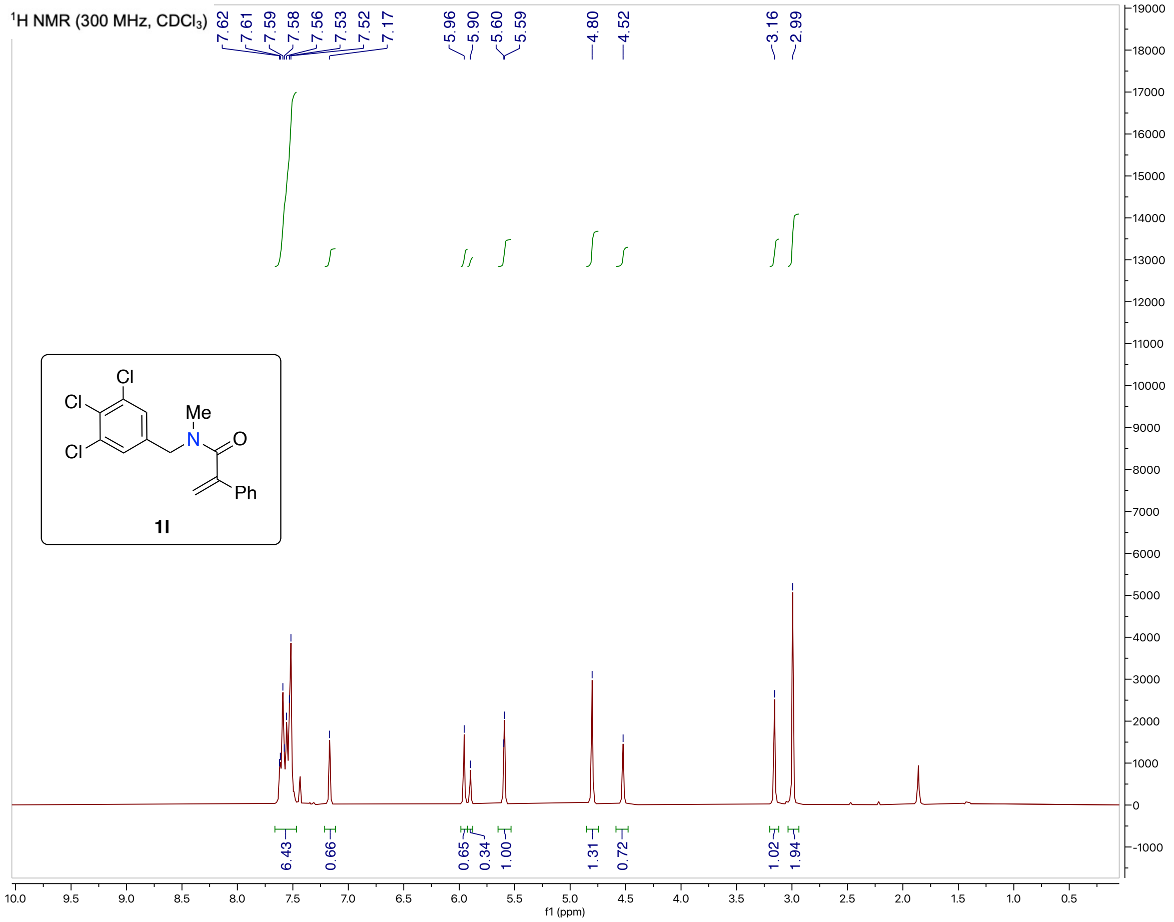

1H NMR (300 MHz, CDCl3)
7.62
7.61
7.59
7.58
7.56
7.53
7.52
7.17
5.96
5.90
5.60
5.59
4.80
4.52
3.16
2.99
6.43
0.66
0.65
0.34
1.00
1.31
0.72
1.02
1.94
Cl
Cl
Cl
Me
N
O
Ph
1l
f1 (ppm)

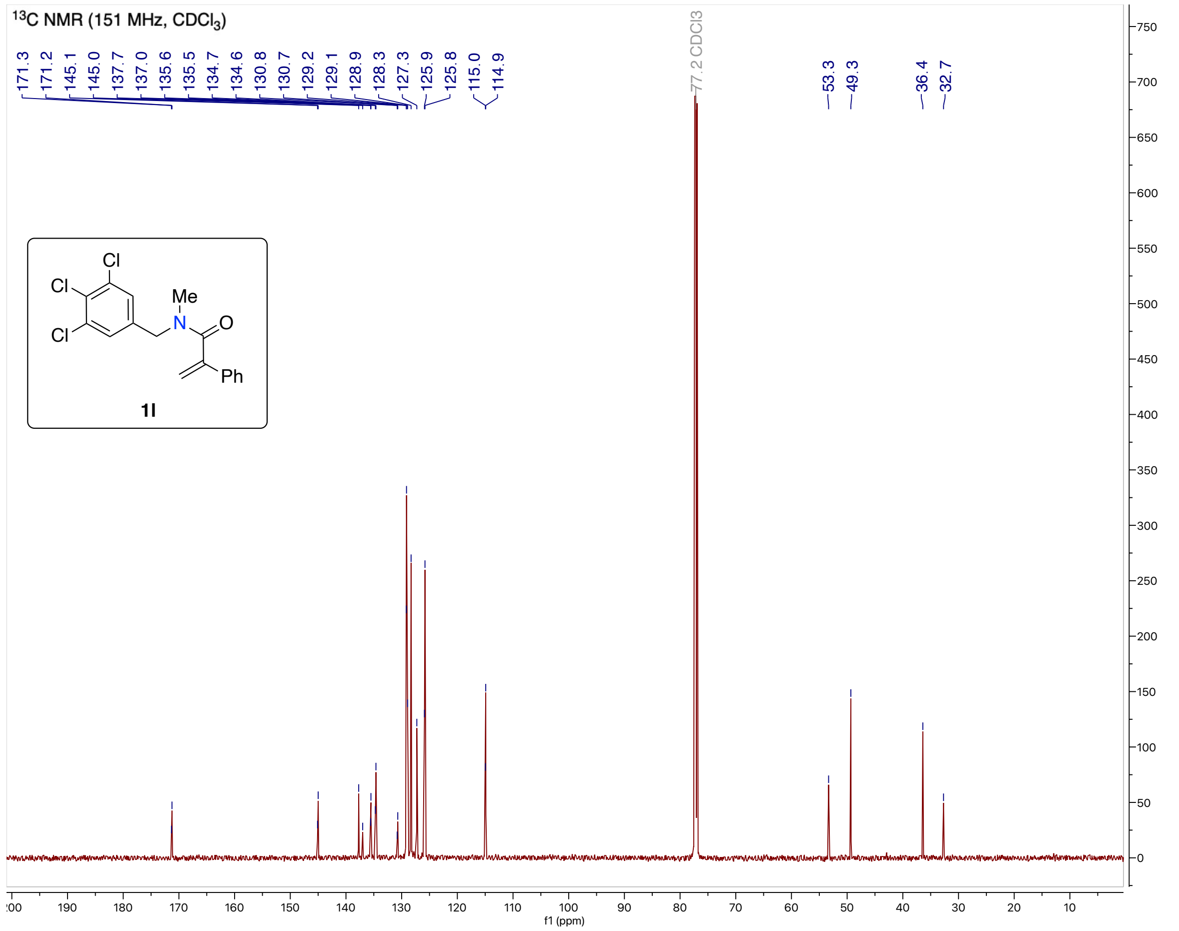

13C NMR (151 MHz, CDCl3)
171.3
171.2
145.1
145.0
137.7
137.0
135.6
135.5
134.7
134.6
130.8
130.7
129.2
129.1
128.9
128.3
127.3
125.9
125.8
115.0
114.9
77.2 CDCl3
53.3
49.3
36.4
32.7
Cl
Cl
Cl
Me
N
O
Ph
1l
f1 (ppm)

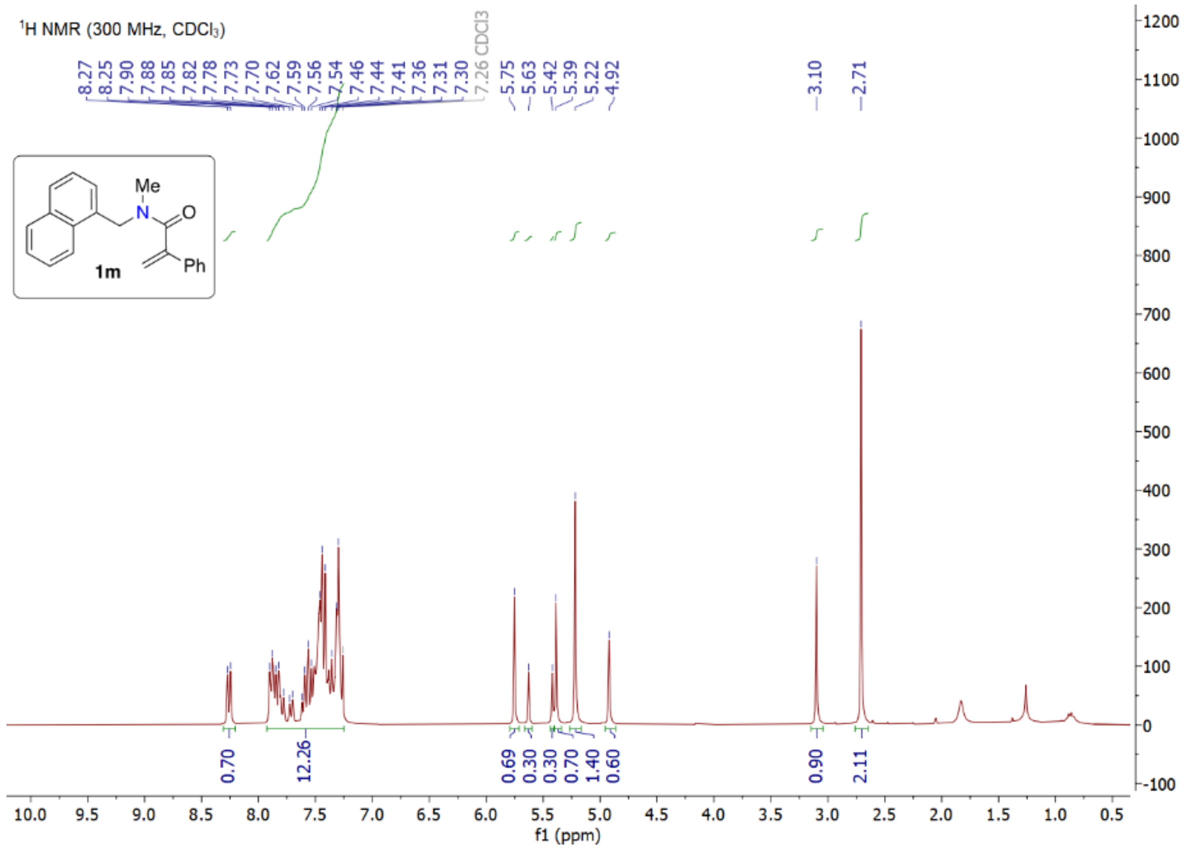
1H NMR (300 MHz, CDCl3)
Me
N
O
Ph
1m
f1 (ppm)

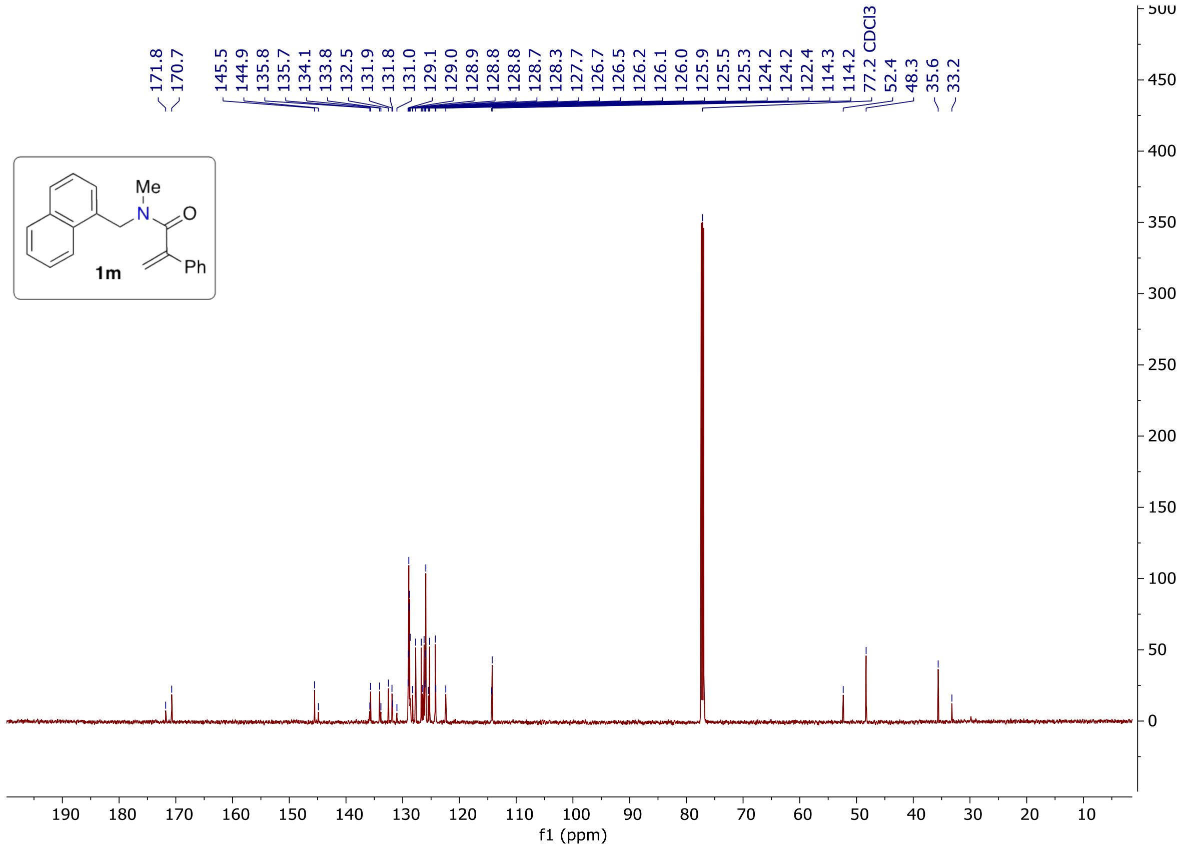
Me
N
O
Ph
1m
f1 (ppm)

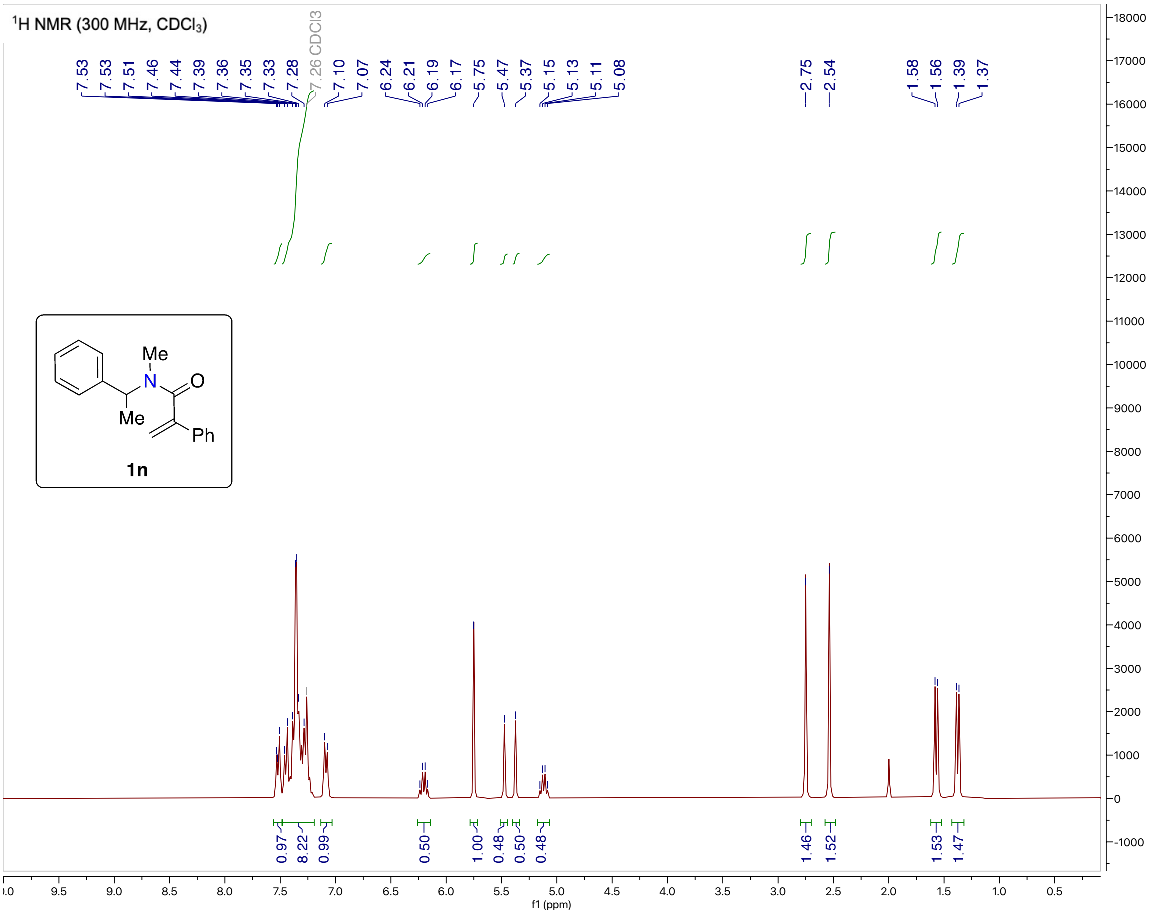

^{1}H NMR (300 MHz, CDCl$_3$)
7.53 7.53 7.51 7.46 7.44 7.39 7.36 7.35 7.33 7.28 7.26 CDCl3 7.10 7.07 6.24 6.21 6.19 6.17 5.75 5.47 5.37 5.15 5.13 5.11 5.08 2.75 2.54 1.58 1.56 1.39 1.37
Me
N
O
Me
Ph
1n
0.97 8.22 0.99 0.50 1.00 0.48 0.50 0.48 1.46 1.52 1.53 1.47
f1 (ppm)

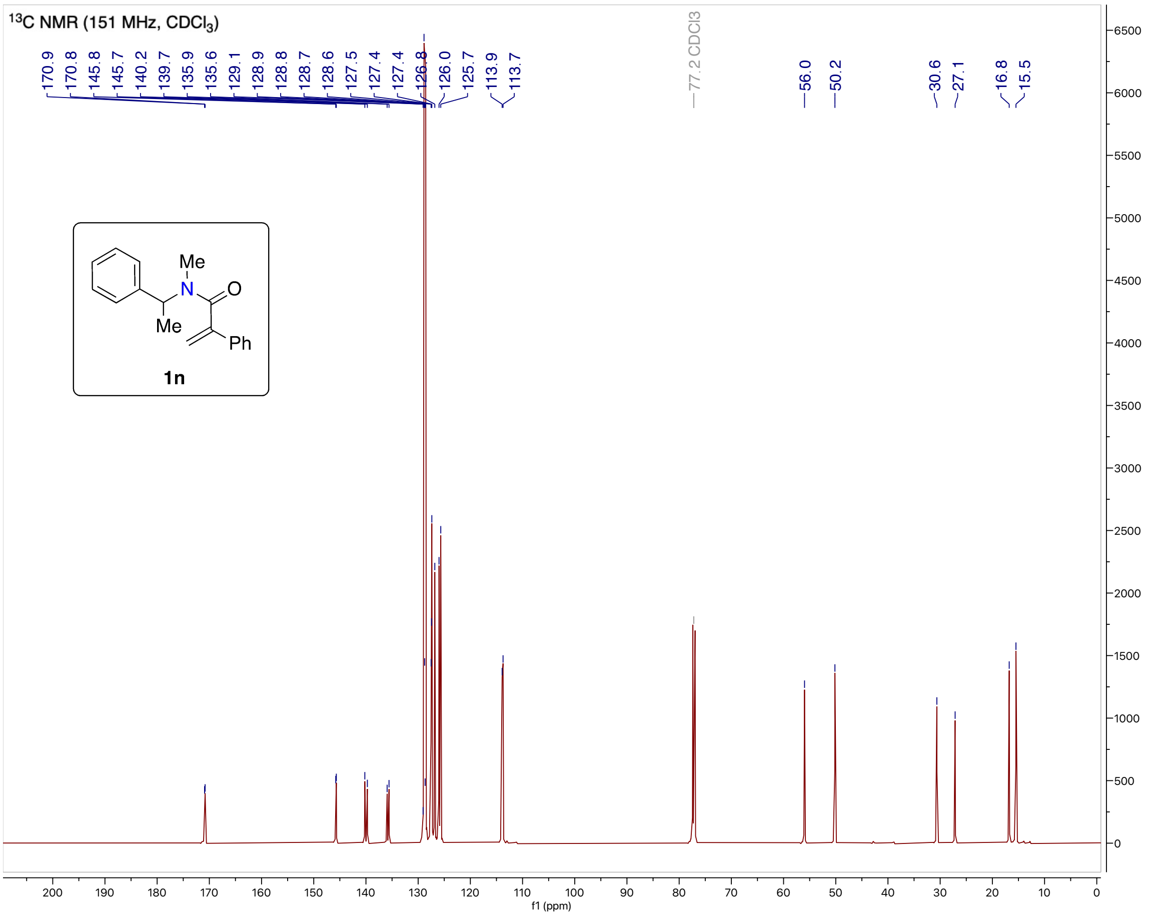

^{13}C NMR (151 MHz, CDCl$_3$)
170.9 170.8 145.8 145.7 140.2 139.7 135.9 135.6 129.1 128.9 128.8 128.7 128.6 127.5 127.4 127.4 126.8 126.0 125.7 113.9 113.7 77.2 CDCl3 56.0 50.2 30.6 27.1 16.8 15.5
Me
N
O
Me
Ph
1n
f1 (ppm)

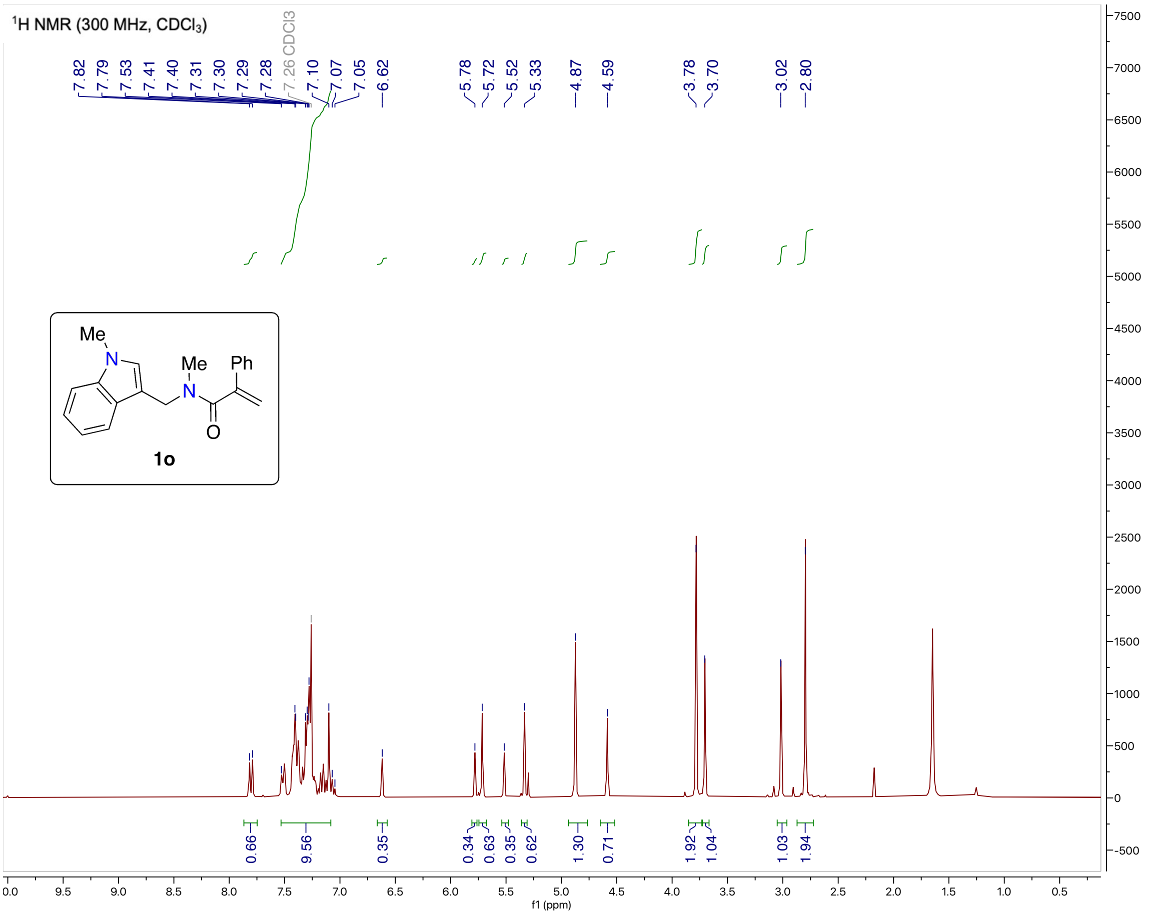
^{1}H NMR (300 MHz, CDCl$_3$)
1o
f1 (ppm)

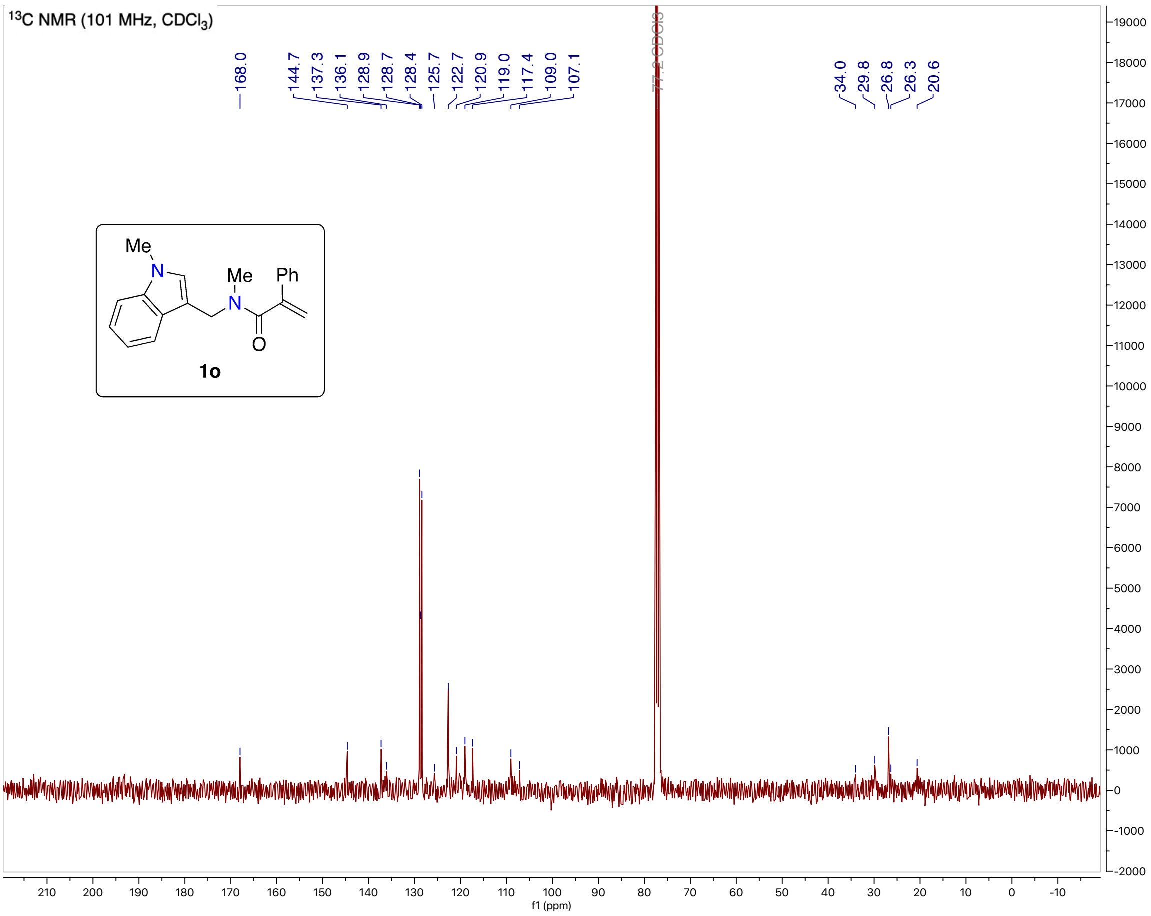
^{13}C NMR (101 MHz, CDCl$_3$)
1o
f1 (ppm)

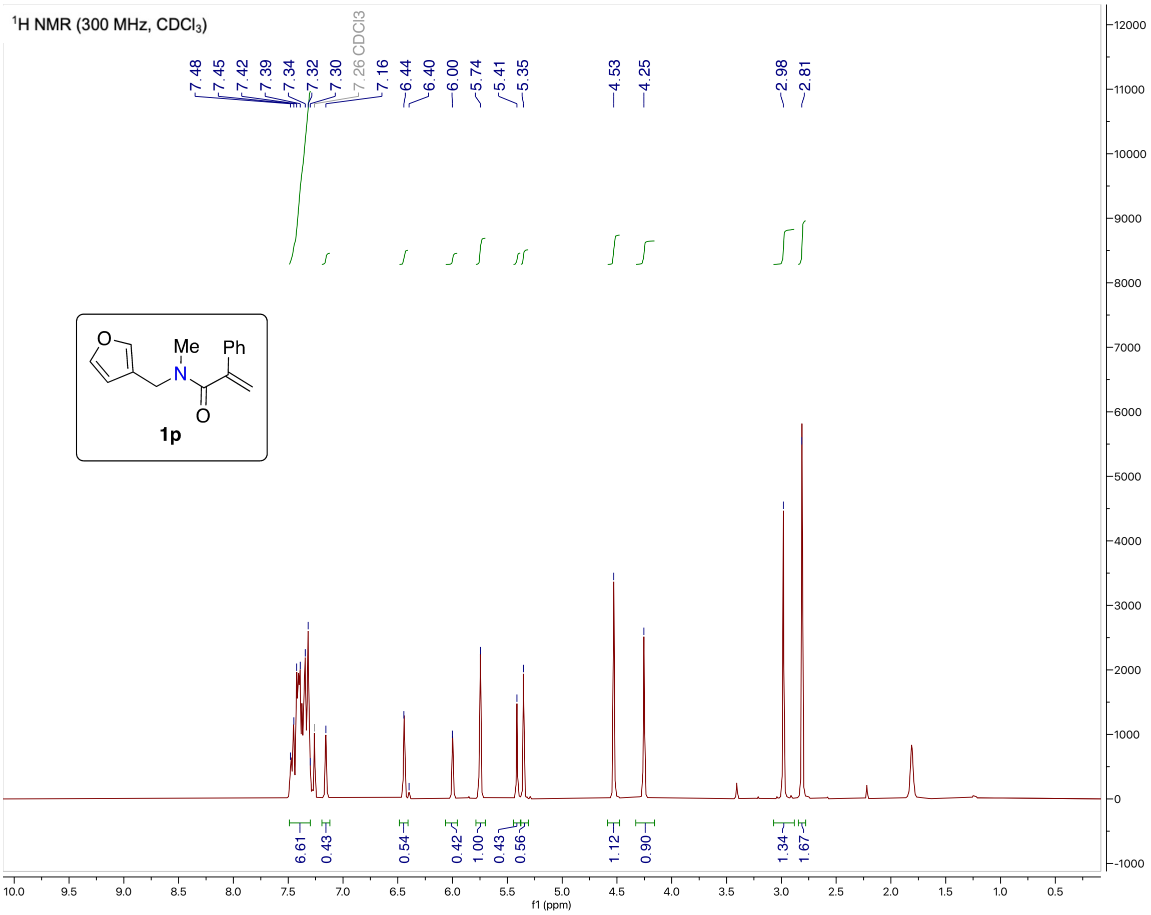

1H NMR (300 MHz, CDCl3)
7.48 7.45 7.42 7.39 7.34 7.32 7.30 7.26 CDCl3 7.16 6.44 6.40 6.00 5.74 5.41 5.35 4.53 4.25 2.98 2.81
6.61 0.43 0.54 0.42 1.00 0.43 0.56 1.12 0.90 1.34 1.67
Me
Ph
N
O
1p
f1 (ppm)

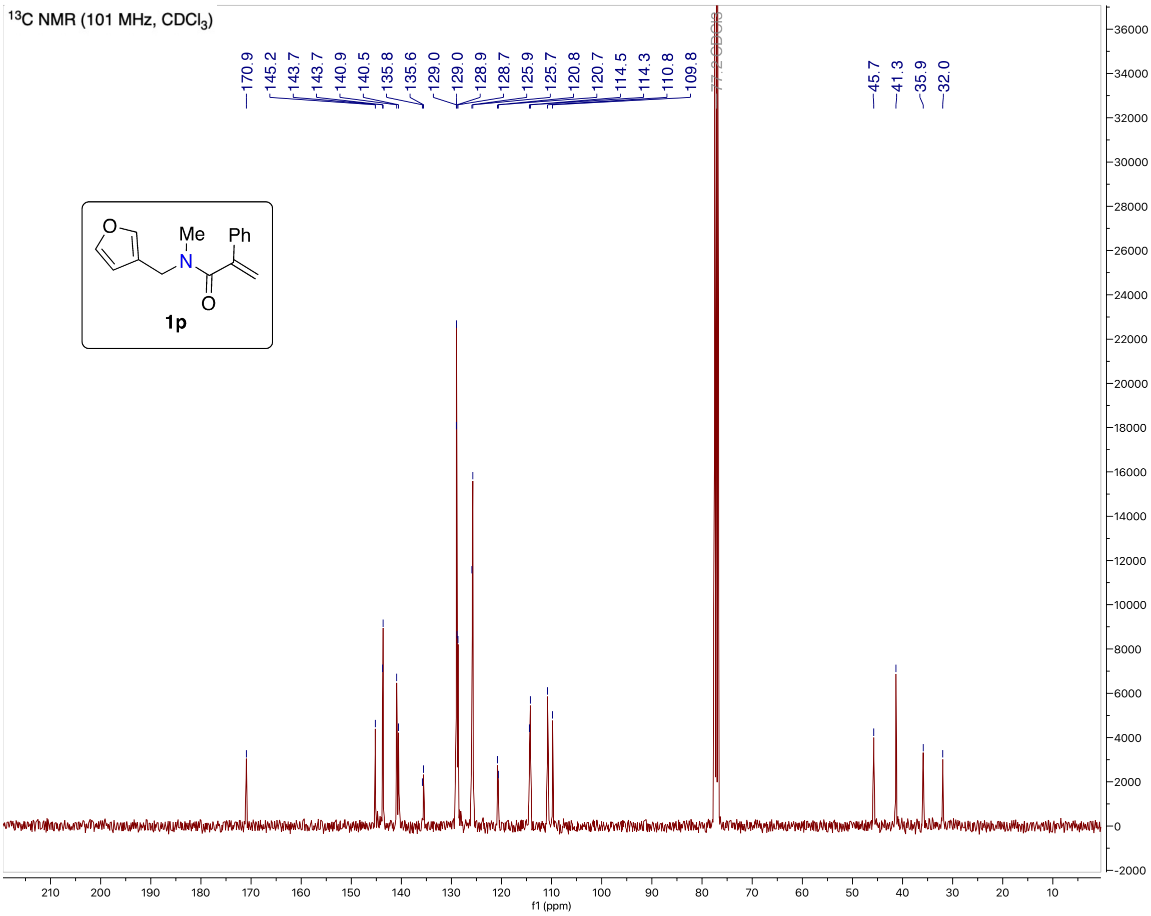

13C NMR (101 MHz, CDCl3)
170.9 145.2 143.7 143.7 140.9 140.5 135.8 135.6 129.0 129.0 128.9 128.7 125.9 125.7 120.8 120.7 114.5 114.3 110.8 109.8 45.7 41.3 35.9 32.0
Me
Ph
N
O
1p
f1 (ppm)

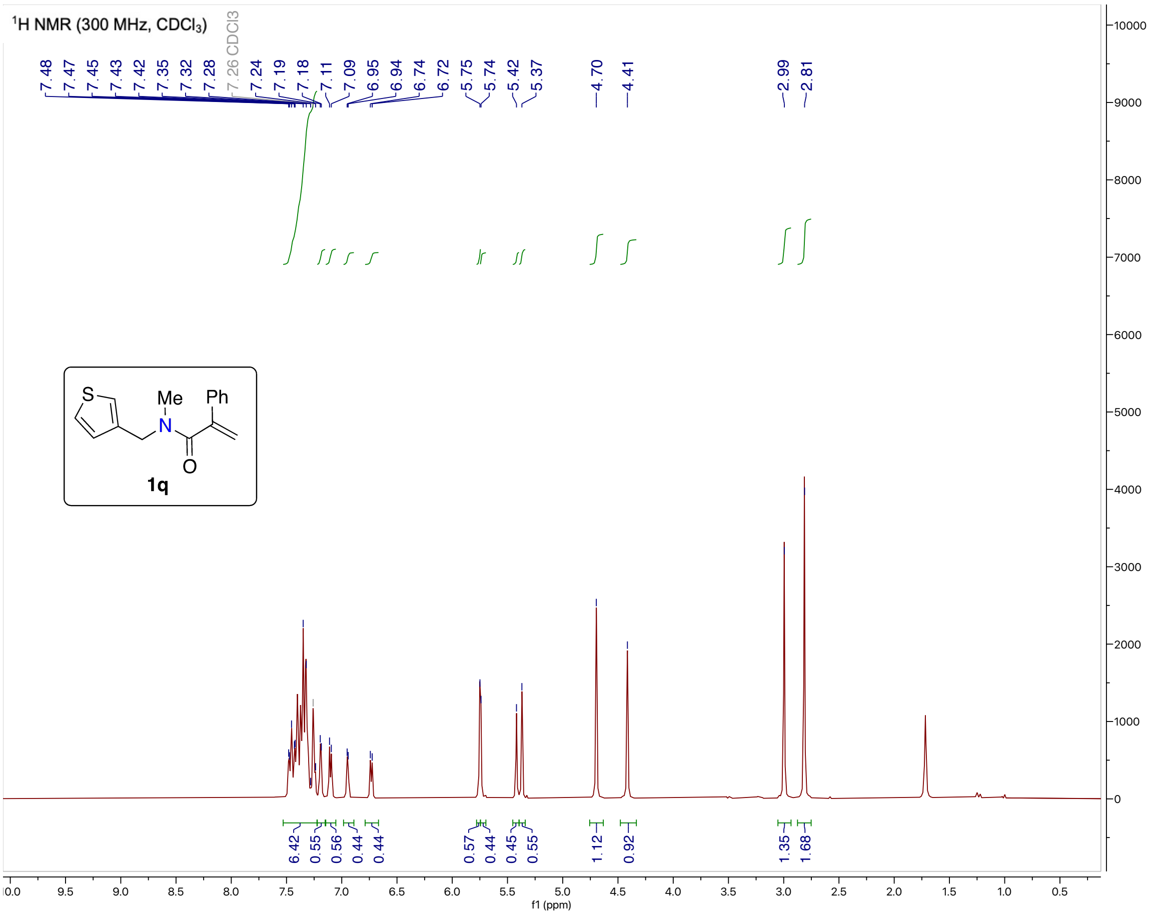

1H NMR (300 MHz, CDCl3)
7.48 7.47 7.45 7.43 7.42 7.35 7.32 7.28 7.26 CDCl3 7.24 7.19 7.18 7.11 7.09 6.95 6.94 6.74 6.72 5.75 5.74 5.42 5.37 4.70 4.41 2.99 2.81
Me
Ph
N
S
O
1q
6.42 0.55 0.56 0.44 0.44 0.57 0.44 0.45 0.55 1.12 0.92 1.35 1.68
f1 (ppm)

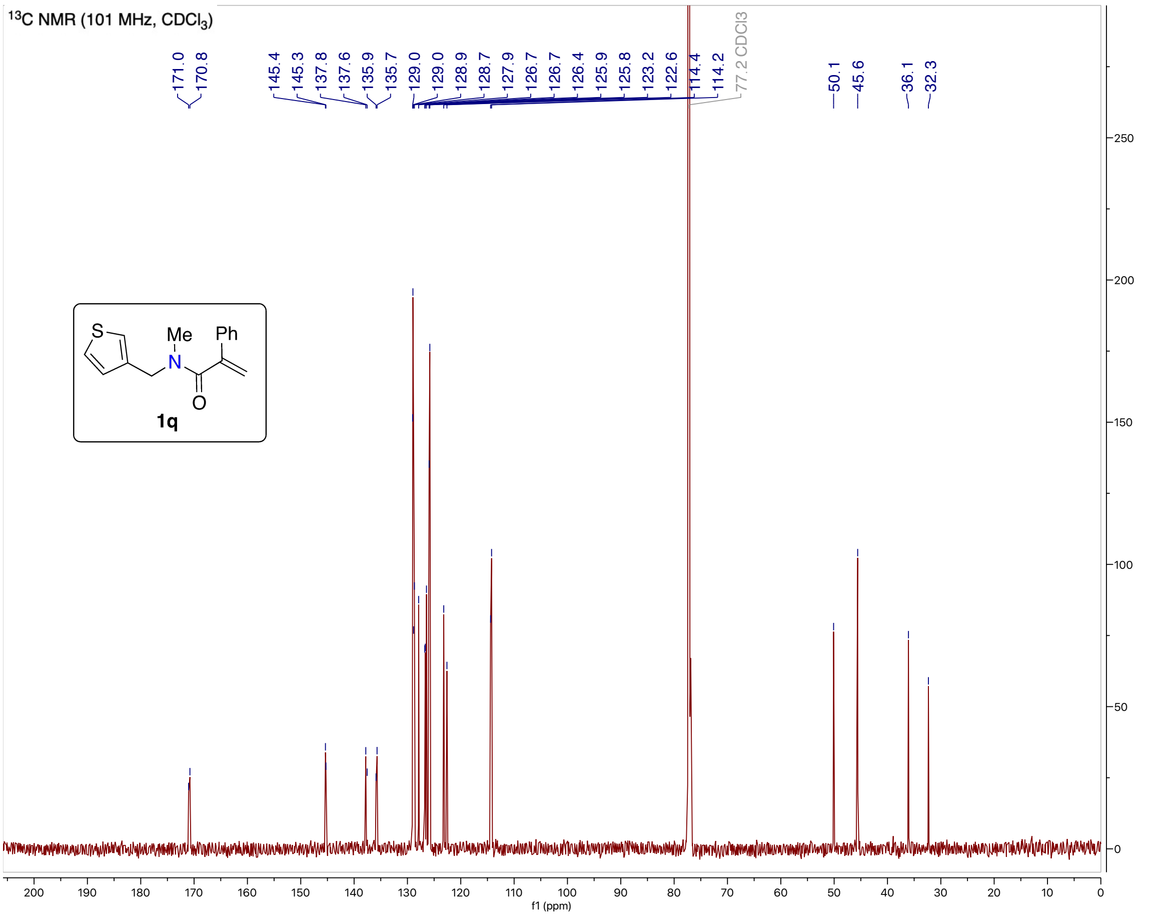

13C NMR (101 MHz, CDCl3)
171.0 170.8 145.4 145.3 137.8 137.6 135.9 135.7 129.0 129.0 128.9 128.7 127.9 126.7 126.7 126.4 125.9 125.8 123.2 122.6 114.4 114.2 77.2 CDCl3 50.1 45.6 36.1 32.3
Me
Ph
N
S
O
1q
f1 (ppm)

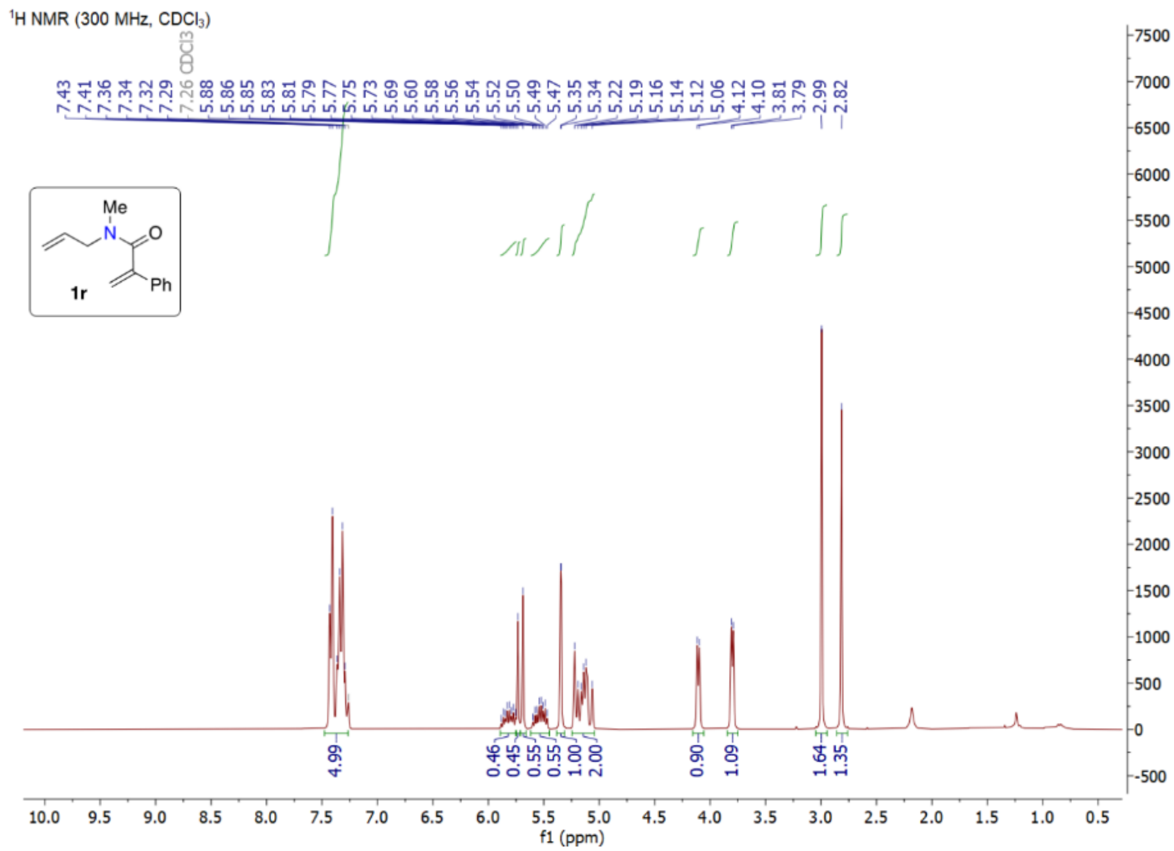
1H NMR (300 MHz, CDCl3)
1r

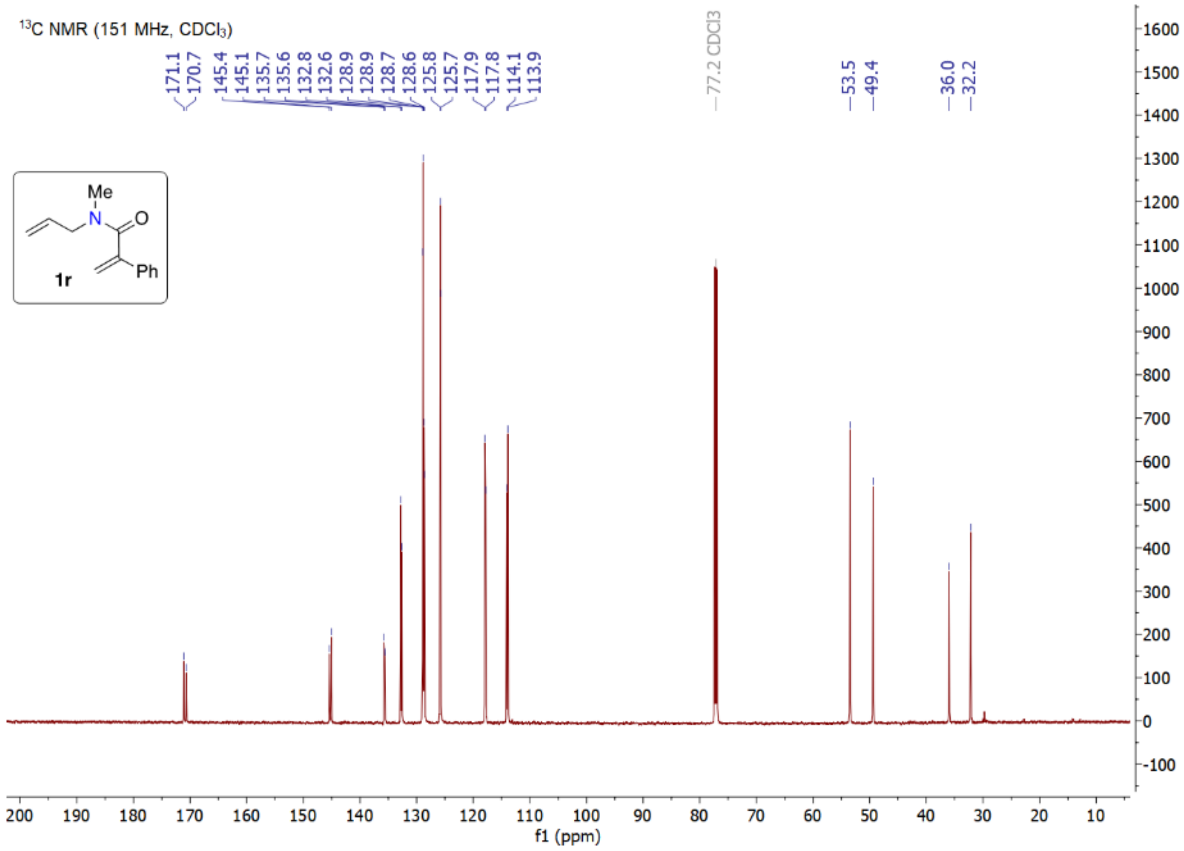
13C NMR (151 MHz, CDCl3)
1r

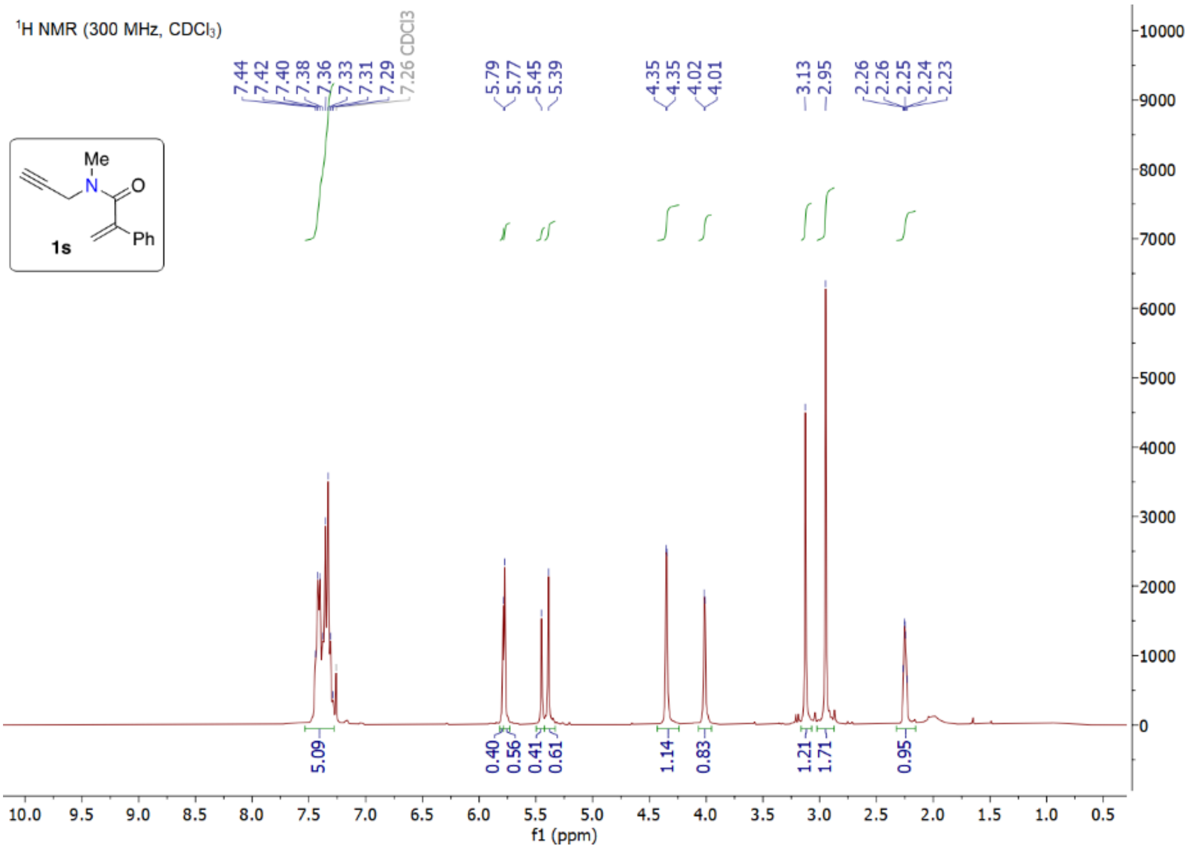

1H NMR (300 MHz, CDCl3)
7.44
7.42
7.40
7.38
7.36
7.33
7.31
7.29
7.26 CDCl3
5.79
5.77
5.45
5.39
4.35
4.35
4.02
4.01
3.13
2.95
2.26
2.26
2.25
2.24
2.23
Me
N
O
Ph
1s
5.09
0.40
0.56
0.41
0.61
1.14
0.83
1.21
1.71
0.95
f1 (ppm)

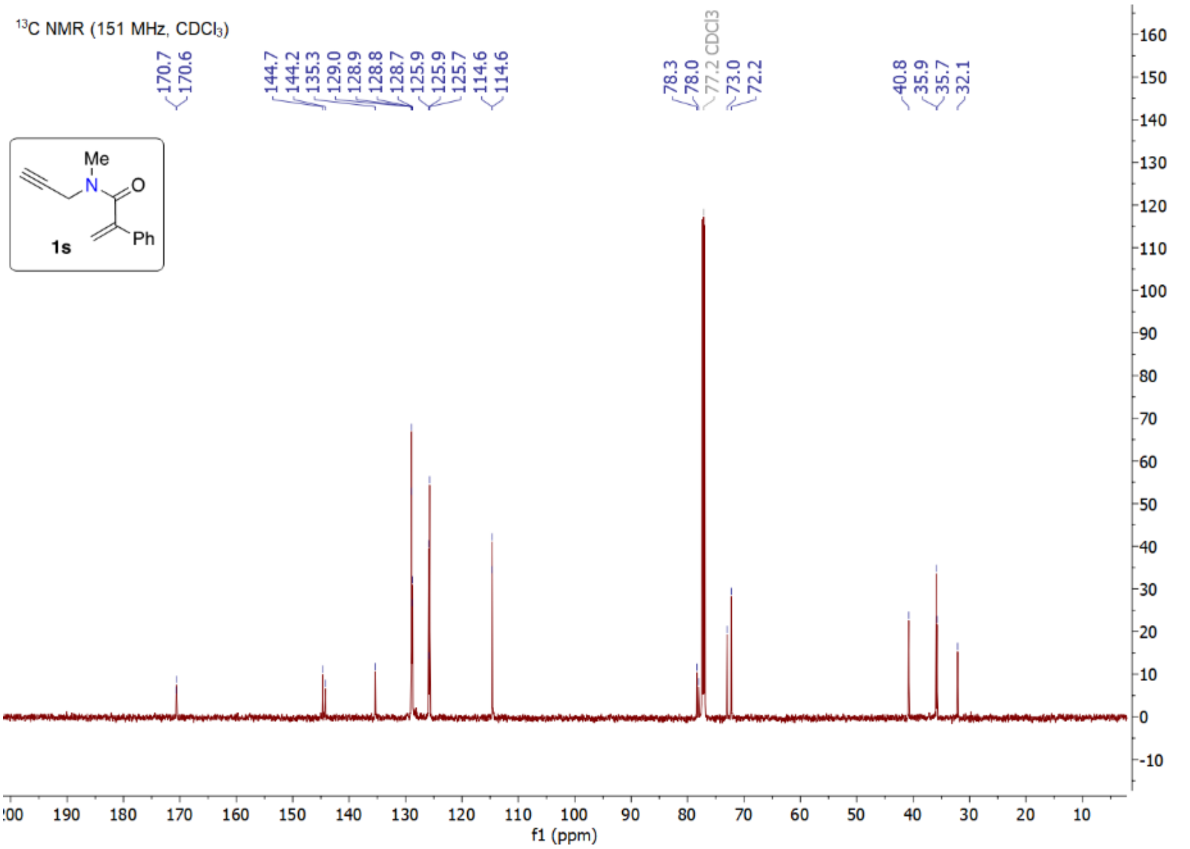

13C NMR (151 MHz, CDCl3)
170.7
170.6
144.7
144.2
135.3
129.0
128.9
128.8
128.7
125.9
125.9
125.7
114.6
114.6
78.3
78.0
77.2 CDCl3
73.0
72.2
40.8
35.9
35.7
32.1
Me
N
O
Ph
1s
f1 (ppm)

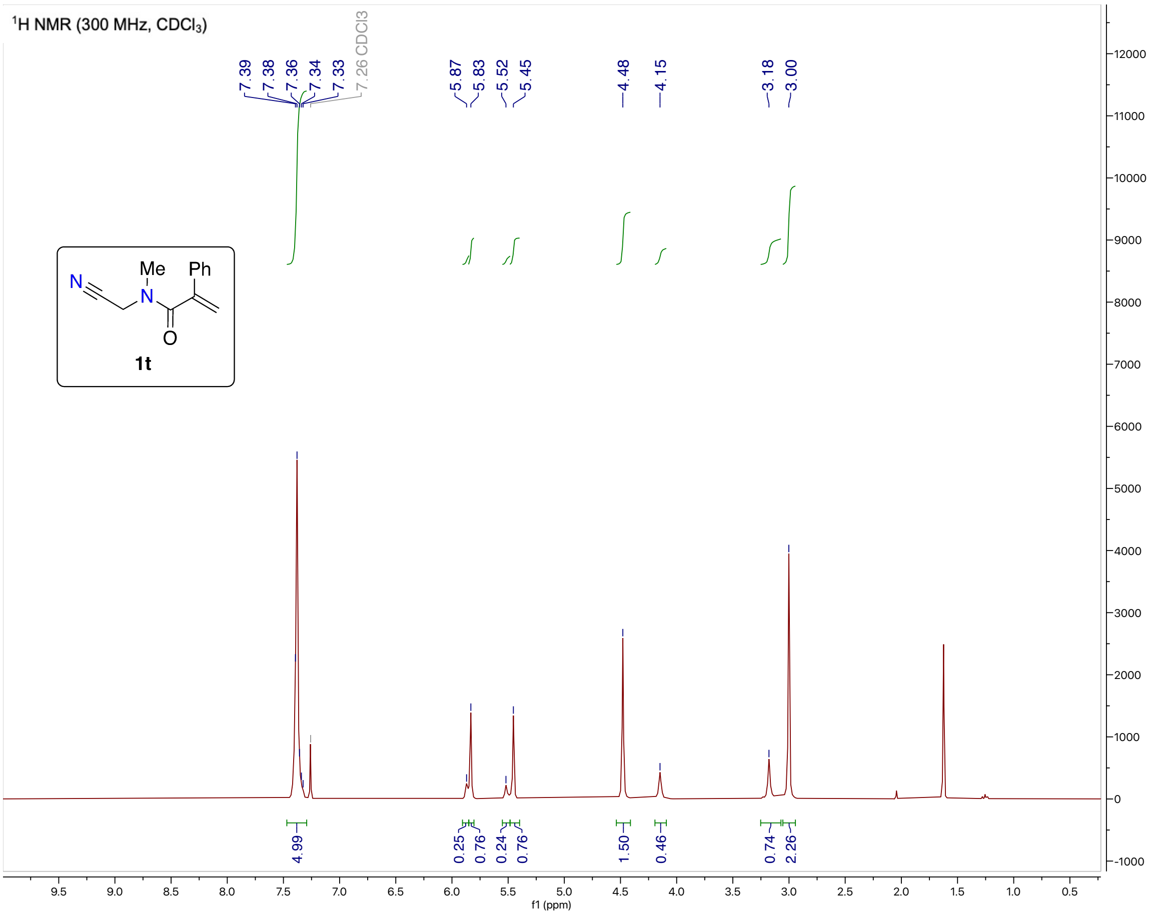

1H NMR (300 MHz, CDCl3)
7.39
7.38
7.36
7.34
7.33
7.26 CDCl3
5.87
5.83
5.52
5.45
4.48
4.15
3.18
3.00
Me
Ph
N
N
O
1t
4.99
0.25
0.76
0.24
0.76
1.50
0.46
0.74
2.26
f1 (ppm)

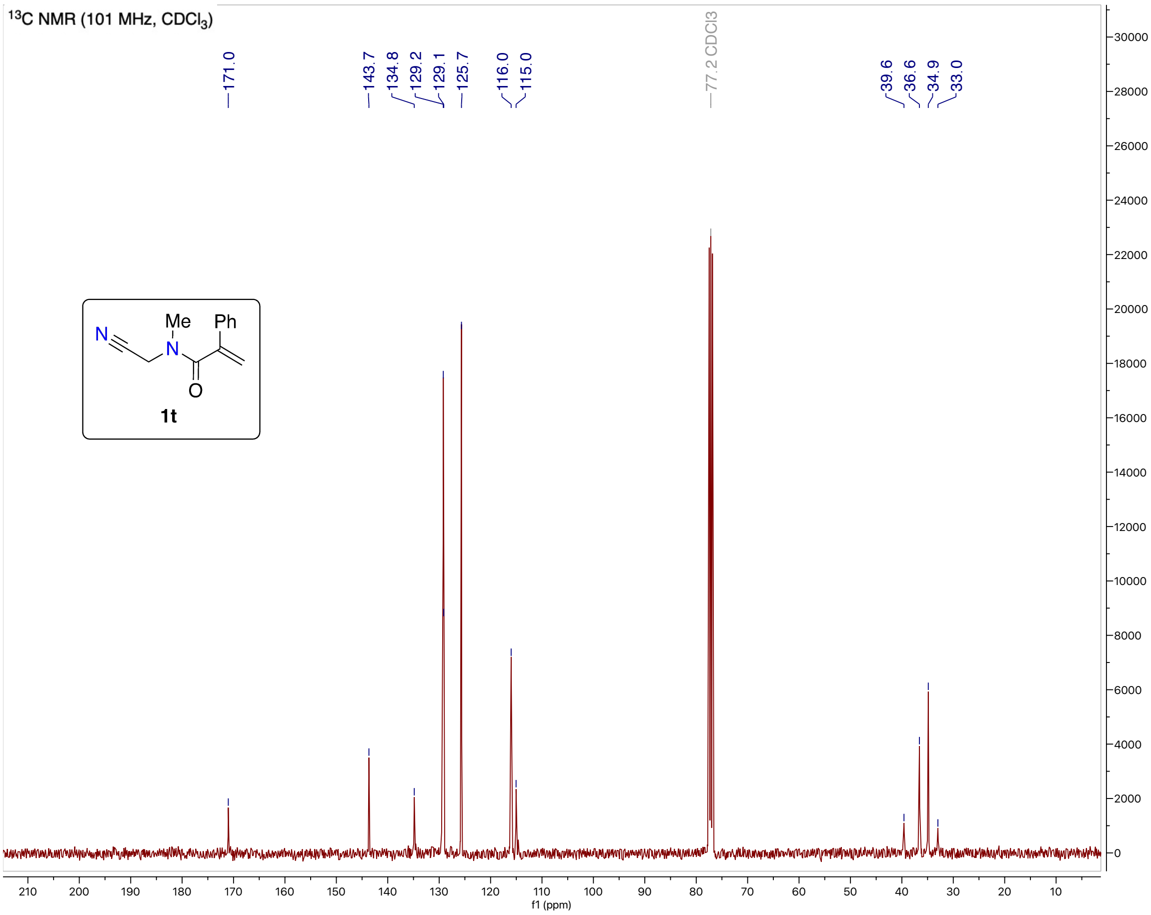

13C NMR (101 MHz, CDCl3)
171.0
143.7
134.8
129.2
129.1
125.7
116.0
115.0
77.2 CDCl3
39.6
36.6
34.9
33.0
Me
Ph
N
N
O
1t
f1 (ppm)

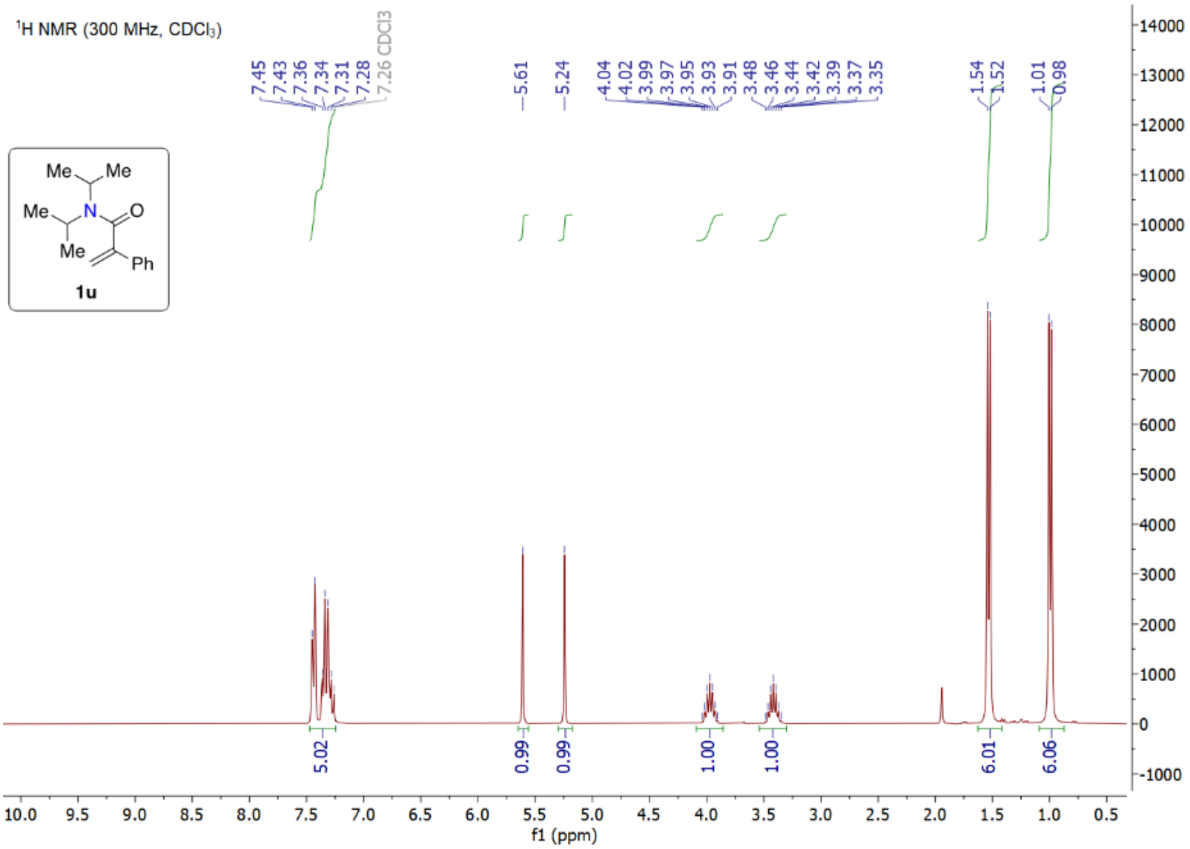
1H NMR (300 MHz, CDCl3)
7.45
7.43
7.36
7.34
7.31
7.28
7.26 CDCl3
5.61
5.24
4.04
4.02
3.99
3.97
3.95
3.93
3.91
3.48
3.46
3.44
3.42
3.39
3.37
3.35
1.54
1.52
1.01
0.98
Me
Me
Me
N
O
Me
Ph
1u
5.02
0.99
0.99
1.00
1.00
6.01
6.06
f1 (ppm)

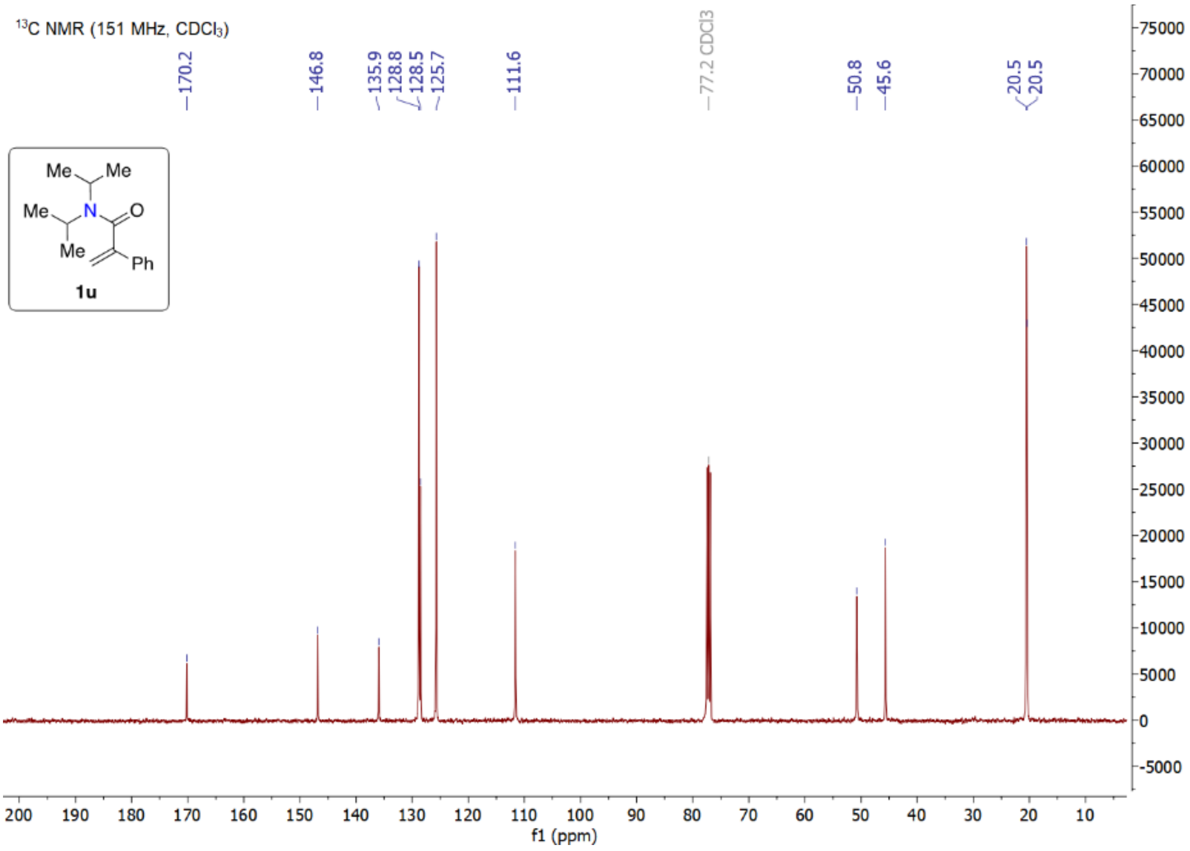
13C NMR (151 MHz, CDCl3)
170.2
146.8
135.9
128.8
128.5
125.7
111.6
77.2 CDCl3
50.8
45.6
20.5
20.5
Me
Me
Me
N
O
Me
Ph
1u
f1 (ppm)

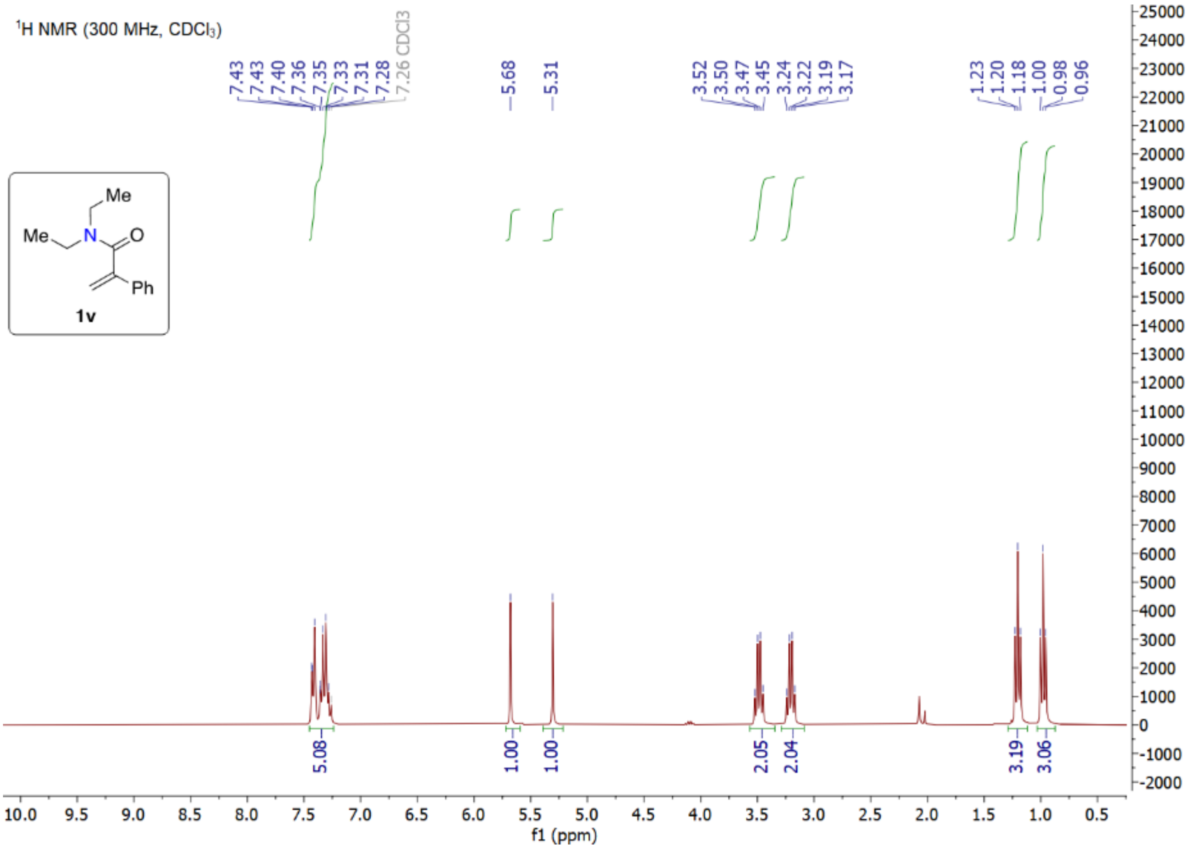

1H NMR (300 MHz, CDCl3)
7.43
7.43
7.40
7.36
7.35
7.33
7.31
7.28
7.26 CDCl3
5.68
5.31
3.52
3.50
3.47
3.45
3.24
3.22
3.19
3.17
1.23
1.20
1.18
1.00
0.98
0.96
Me
Me
N
O
Ph
1v
5.08
1.00
1.00
2.05
2.04
3.19
3.06
f1 (ppm)

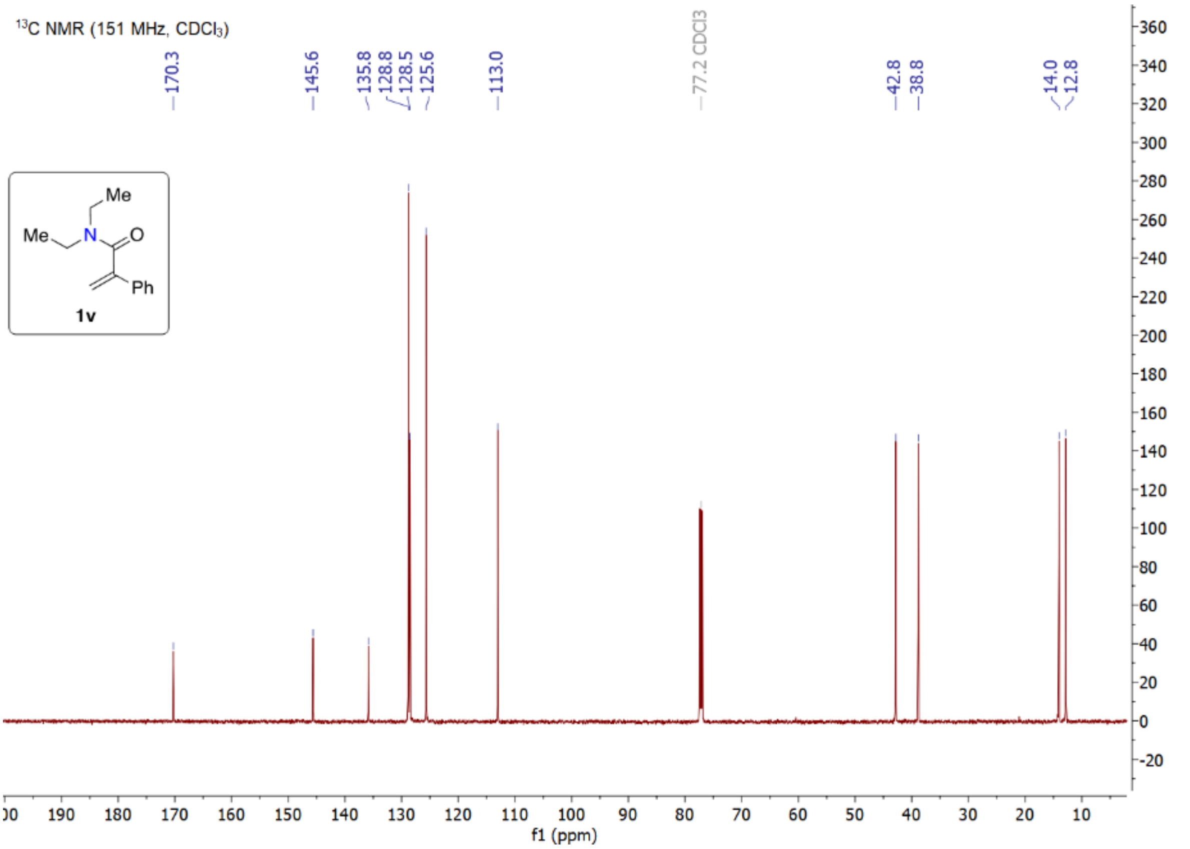

13C NMR (151 MHz, CDCl3)
170.3
145.6
135.8
128.8
128.5
125.6
113.0
77.2 CDCl3
42.8
38.8
14.0
12.8
Me
Me
N
O
Ph
1v
f1 (ppm)

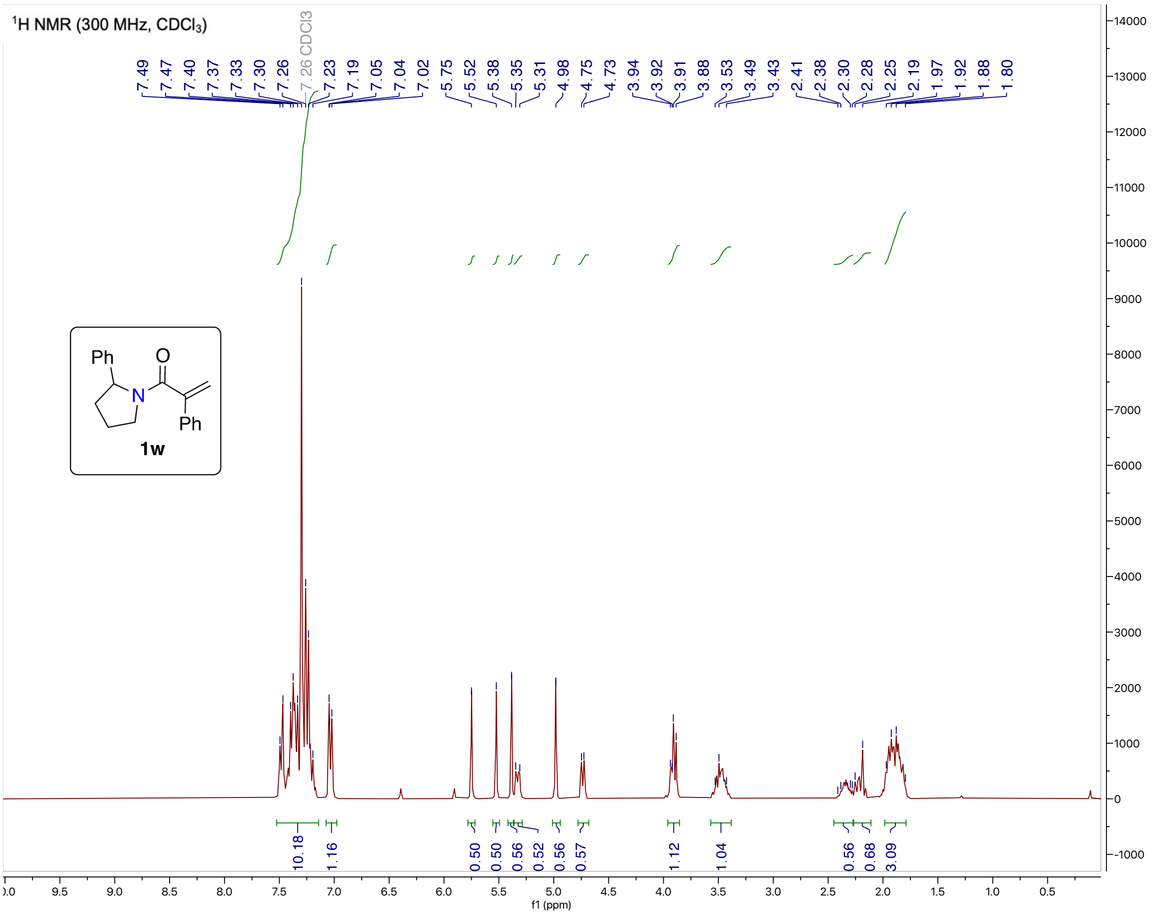
1H NMR (300 MHz, CDCl3)
7.49
7.47
7.40
7.37
7.33
7.30
7.26
7.26 CDCl3
7.23
7.19
7.05
7.04
7.02
5.75
5.52
5.38
5.35
5.31
4.98
4.75
4.73
3.94
3.92
3.91
3.88
3.53
3.49
3.43
2.41
2.38
2.30
2.28
2.25
2.19
1.97
1.92
1.88
1.80
Ph
O
N
Ph
1w
10.18
1.16
0.50
0.50
0.56
0.52
0.56
0.57
1.12
1.04
0.56
0.68
3.09
f1 (ppm)

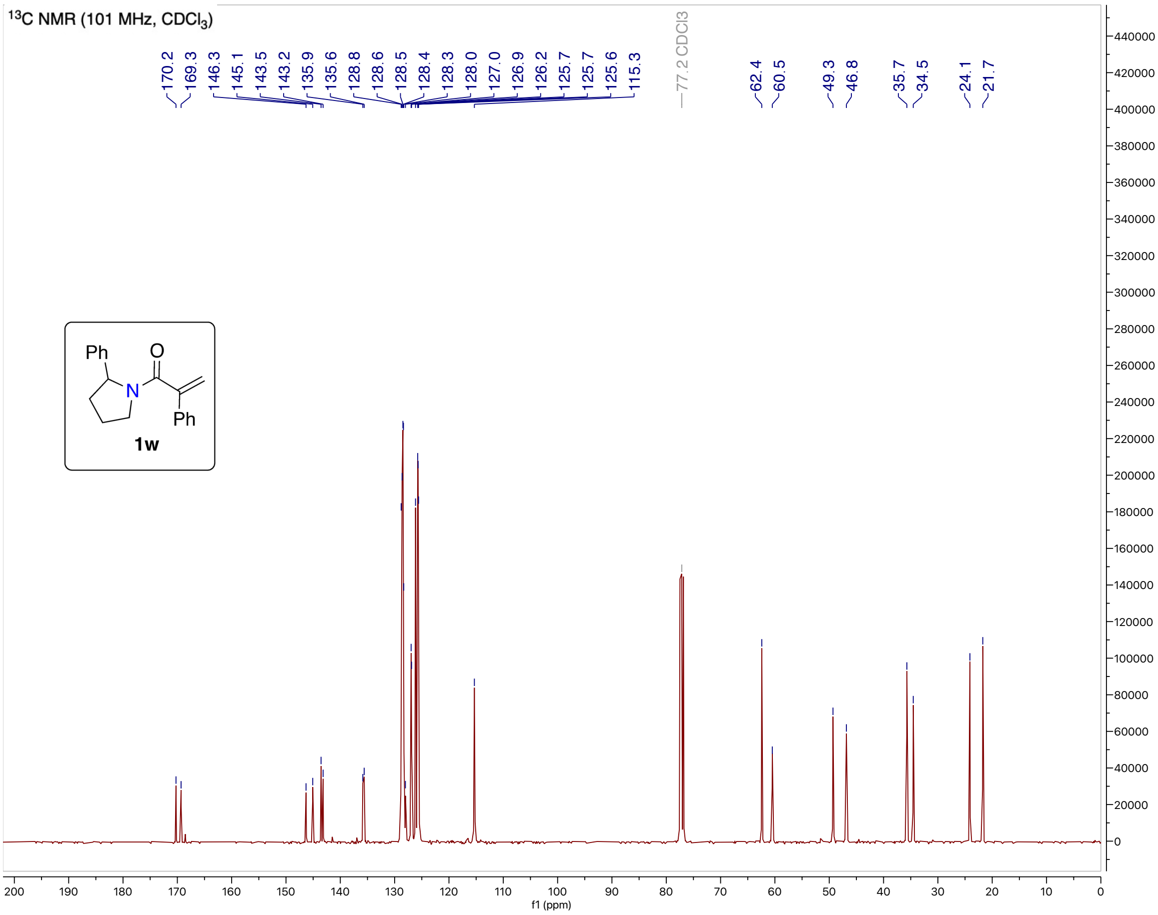
13C NMR (101 MHz, CDCl3)
170.2
169.3
146.3
145.1
143.5
143.2
135.9
135.6
128.8
128.6
128.5
128.4
128.3
128.0
127.0
126.9
126.2
125.7
125.7
125.6
115.3
77.2 CDCl3
62.4
60.5
49.3
46.8
35.7
34.5
24.1
21.7
Ph
O
N
Ph
1w
f1 (ppm)

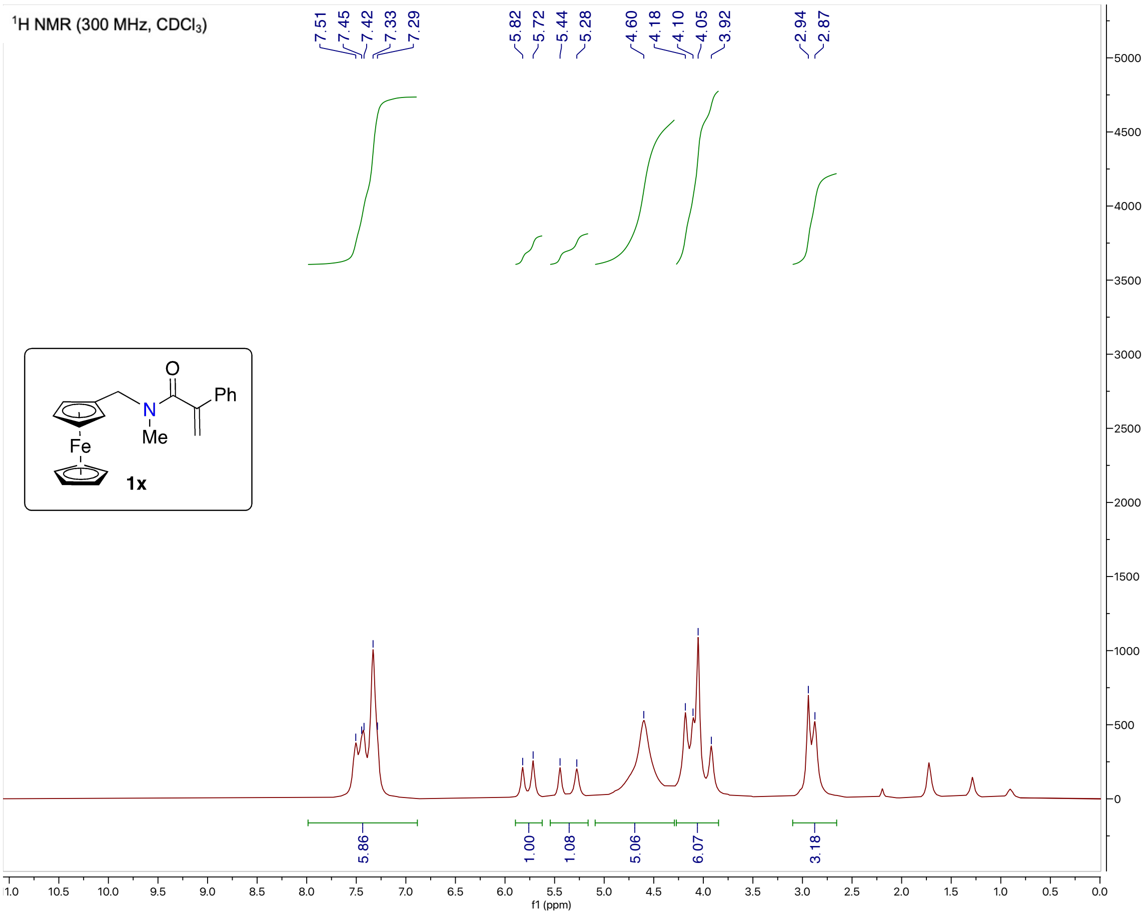

^{1}H NMR (300 MHz, CDCl$_3$)
7.51
7.45
7.42
7.33
7.29
5.82
5.72
5.44
5.28
4.60
4.18
4.10
4.05
3.92
2.94
2.87
Ph
O
N
Me
Fe
1x
5.86
1.00
1.08
5.06
6.07
3.18
f1 (ppm)

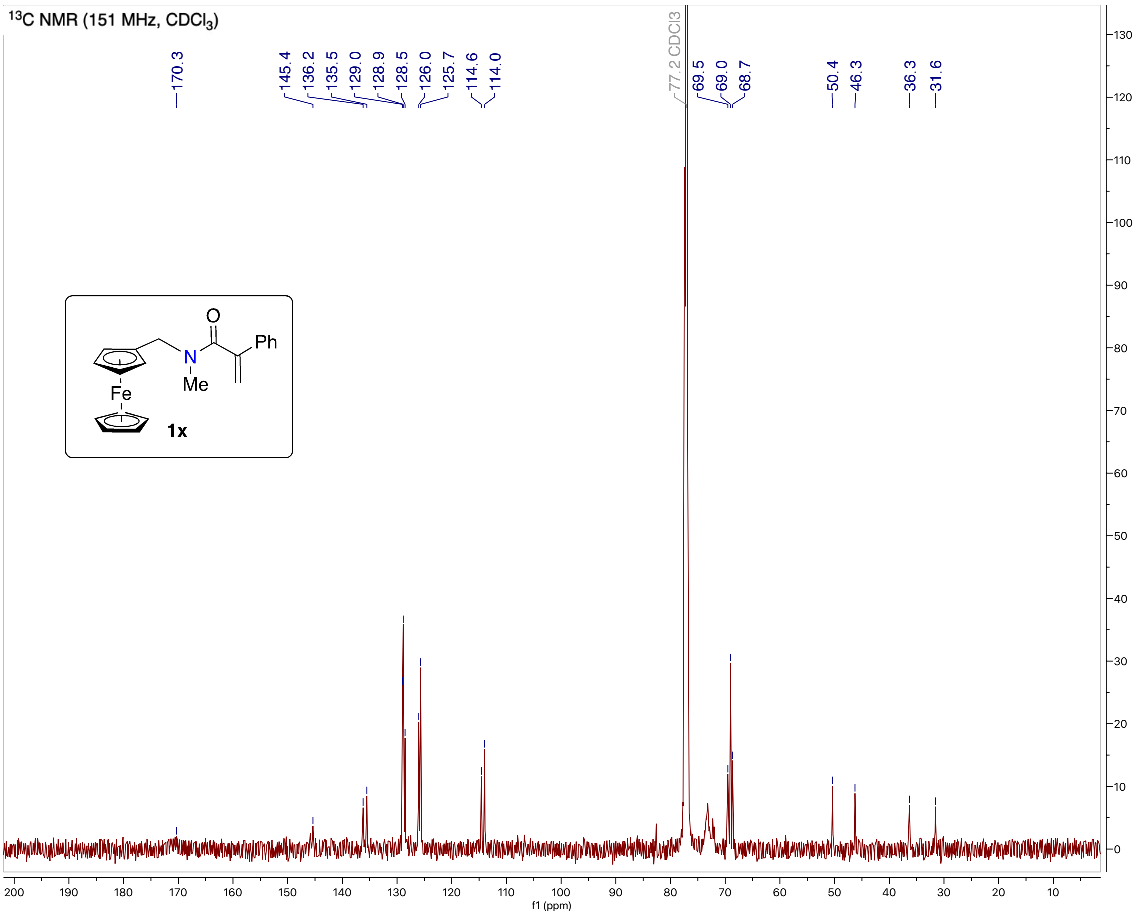

^{13}C NMR (151 MHz, CDCl$_3$)
170.3
145.4
136.2
135.5
129.0
128.9
128.5
126.0
125.7
114.6
114.0
77.2 CDCl3
69.5
69.0
68.7
50.4
46.3
36.3
31.6
Ph
O
N
Me
Fe
1x
f1 (ppm)

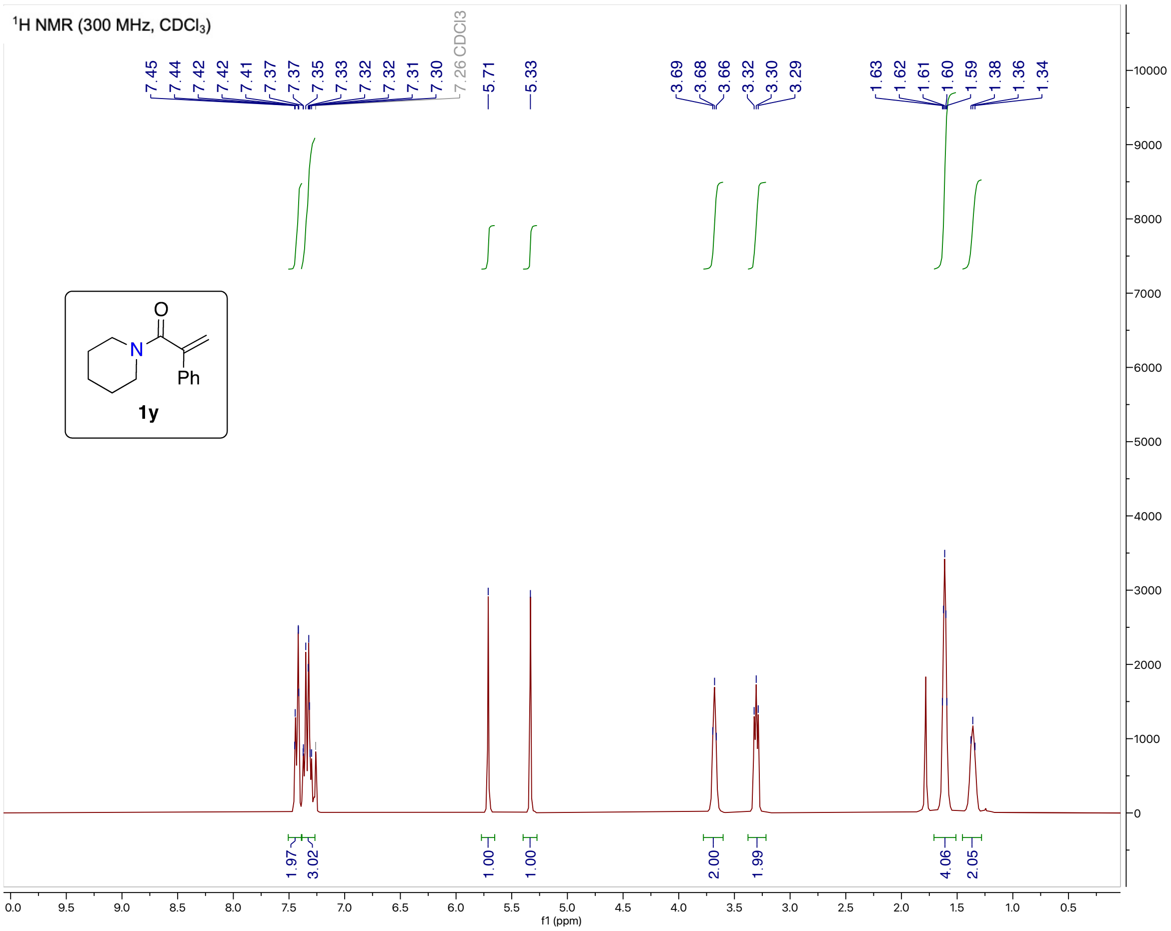

^{1}H NMR (300 MHz, CDCl3)
7.45 7.44 7.42 7.42 7.41 7.37 7.37 7.35 7.33 7.32 7.32 7.31 7.30 7.26 CDCl3 5.71 5.33 3.69 3.68 3.66 3.32 3.30 3.29 1.63 1.62 1.61 1.60 1.59 1.38 1.36 1.34
1.97 3.02 1.00 1.00 2.00 1.99 4.06 2.05
O
N
Ph
1y
f1 (ppm)

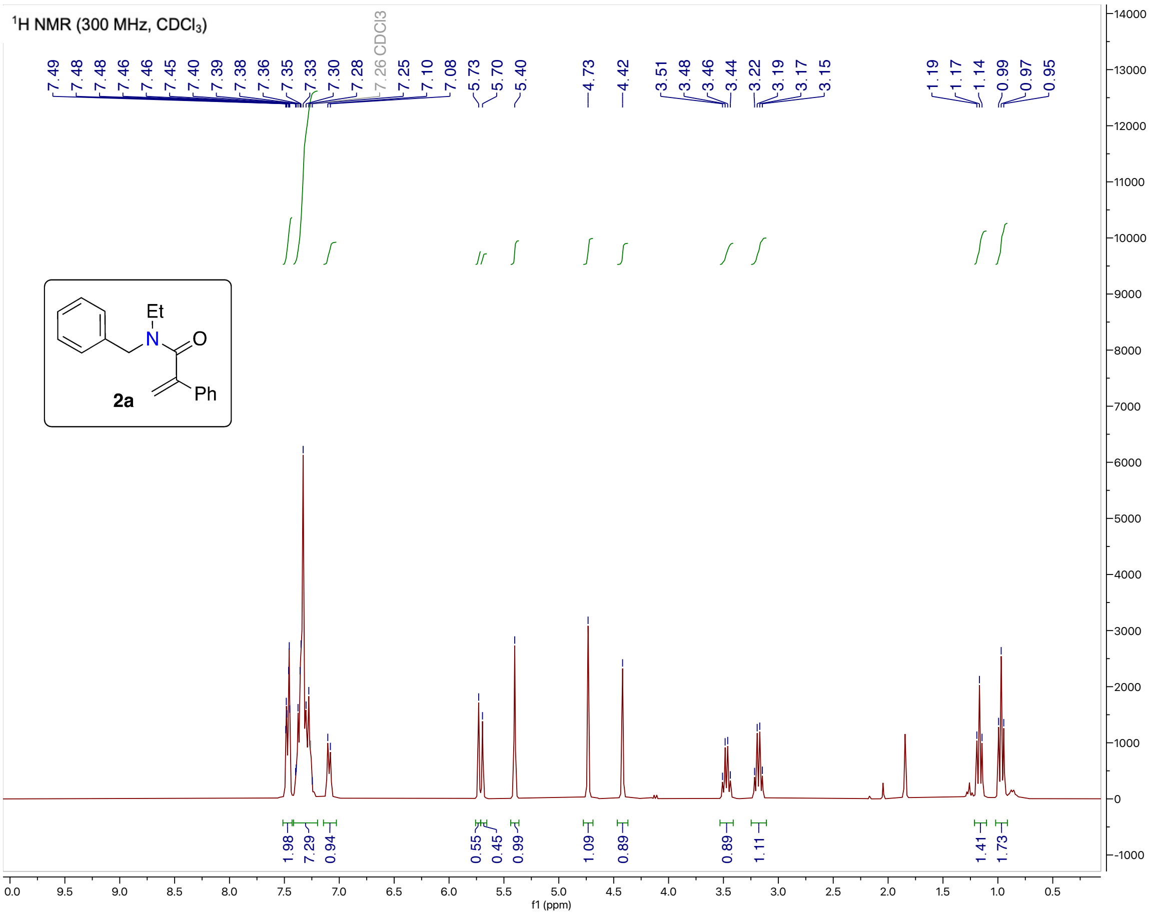
^{1}H NMR (300 MHz, CDCl3)
2a
Et
N
O
Ph
f1 (ppm)

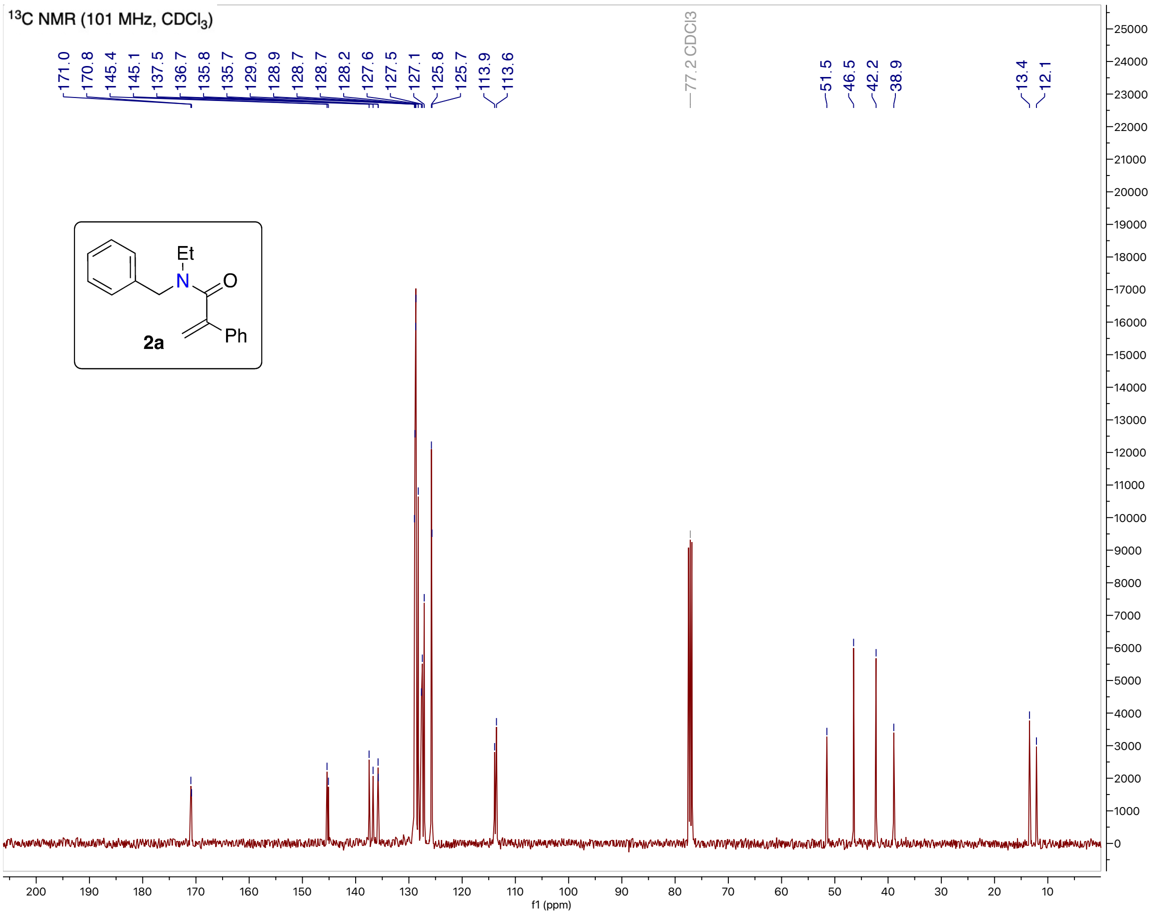
^{13}C NMR (101 MHz, CDCl3)
2a
Et
N
O
Ph
f1 (ppm)

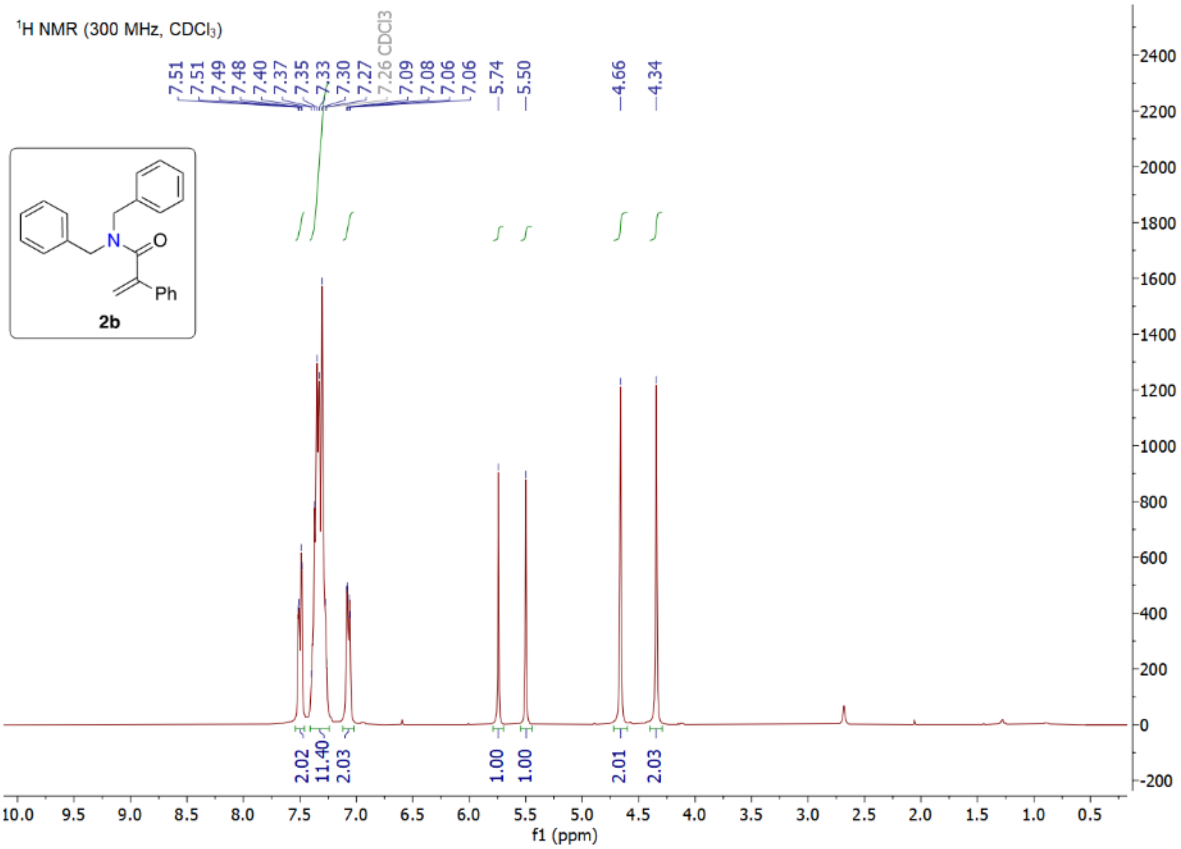

1H NMR (300 MHz, CDCl3)
7.51 7.51 7.49 7.48 7.40 7.37 7.35 7.33 7.30 7.27 7.26 CDCl3 7.09 7.08 7.06 7.06
5.74
5.50
4.66
4.34
N
O
Ph
2b
2.02
11.40
2.03
1.00
1.00
2.01
2.03
f1 (ppm)

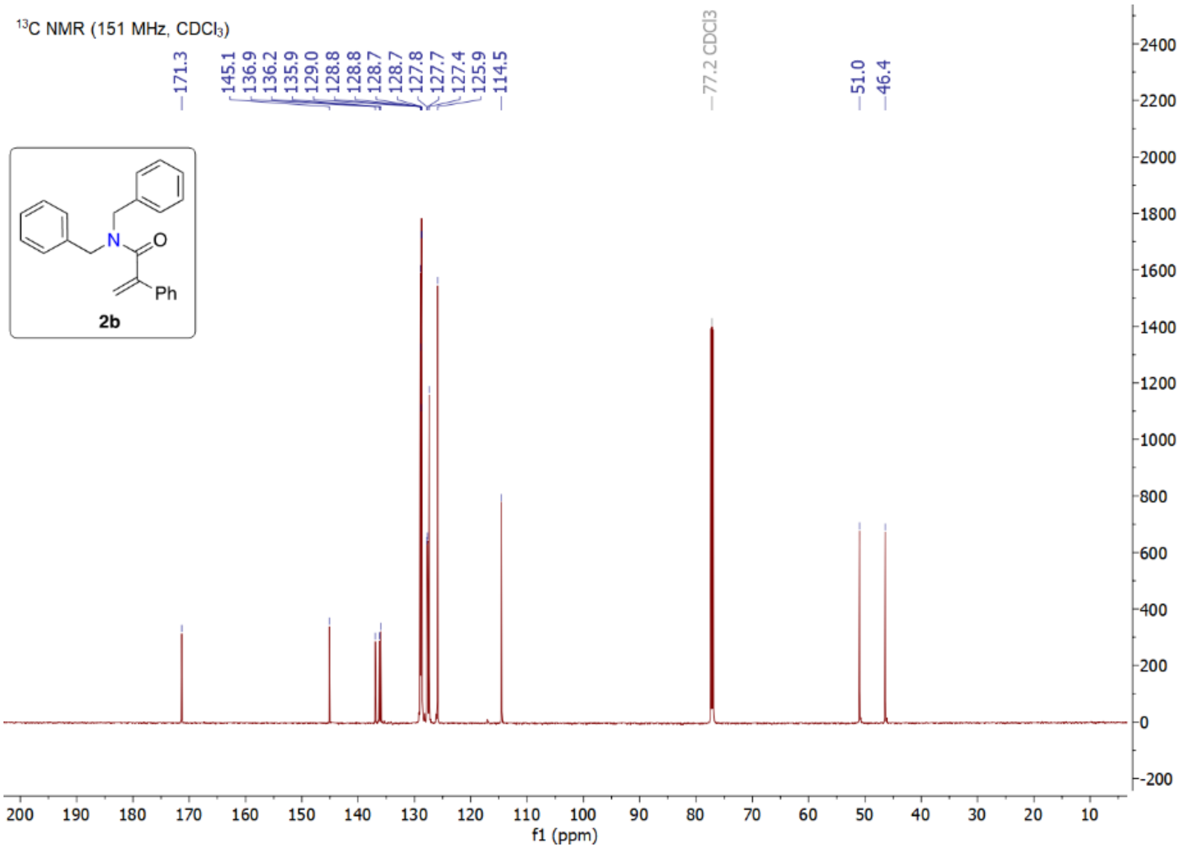

13C NMR (151 MHz, CDCl3)
171.3
145.1 136.9 136.2 135.9 129.0 128.8 128.8 128.7 128.7 127.8 127.7 127.4 125.9
114.5
77.2 CDCl3
51.0
46.4
N
O
Ph
2b
f1 (ppm)

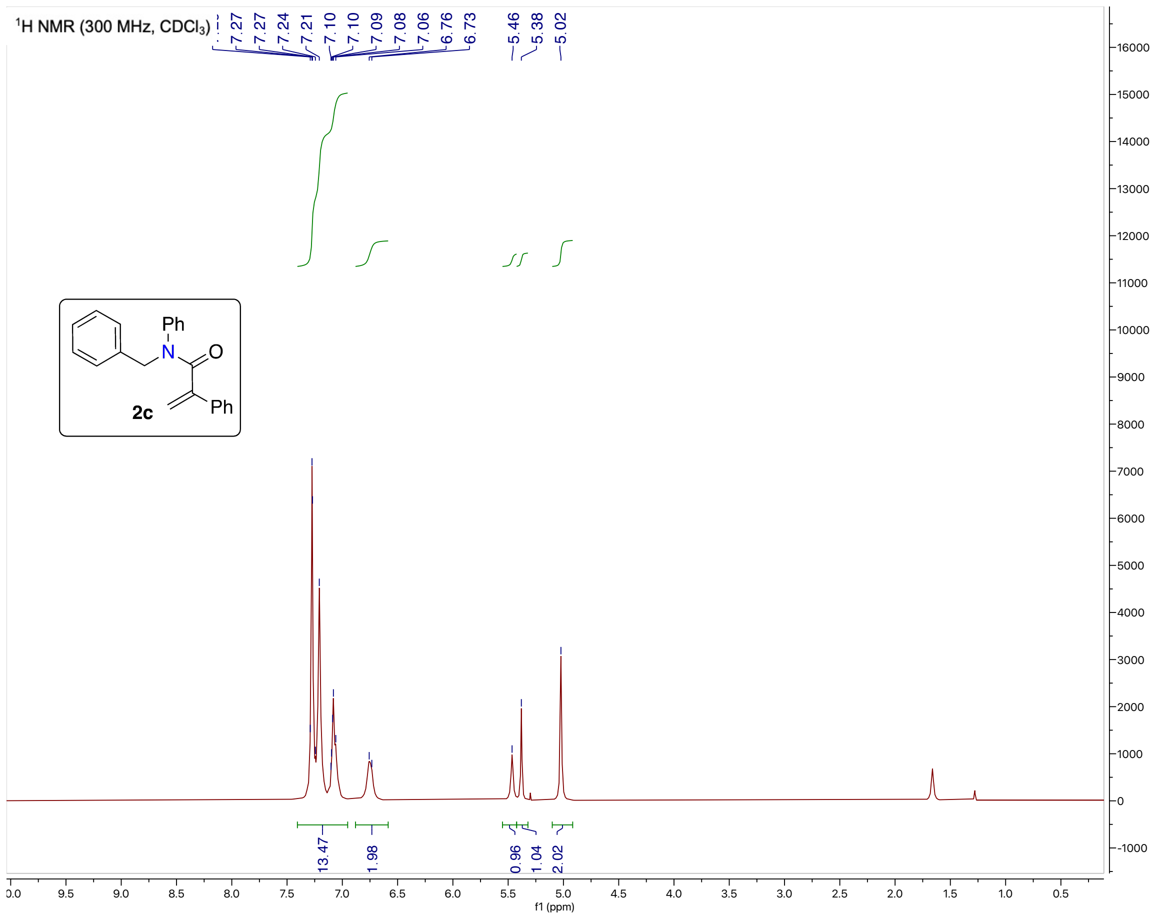
1H NMR (300 MHz, CDCl3)
7.27
7.27
7.24
7.21
7.10
7.10
7.09
7.08
7.06
6.76
6.73
5.46
5.38
5.02
Ph
N
O
2c
Ph
13.47
1.98
0.96
1.04
2.02
f1 (ppm)

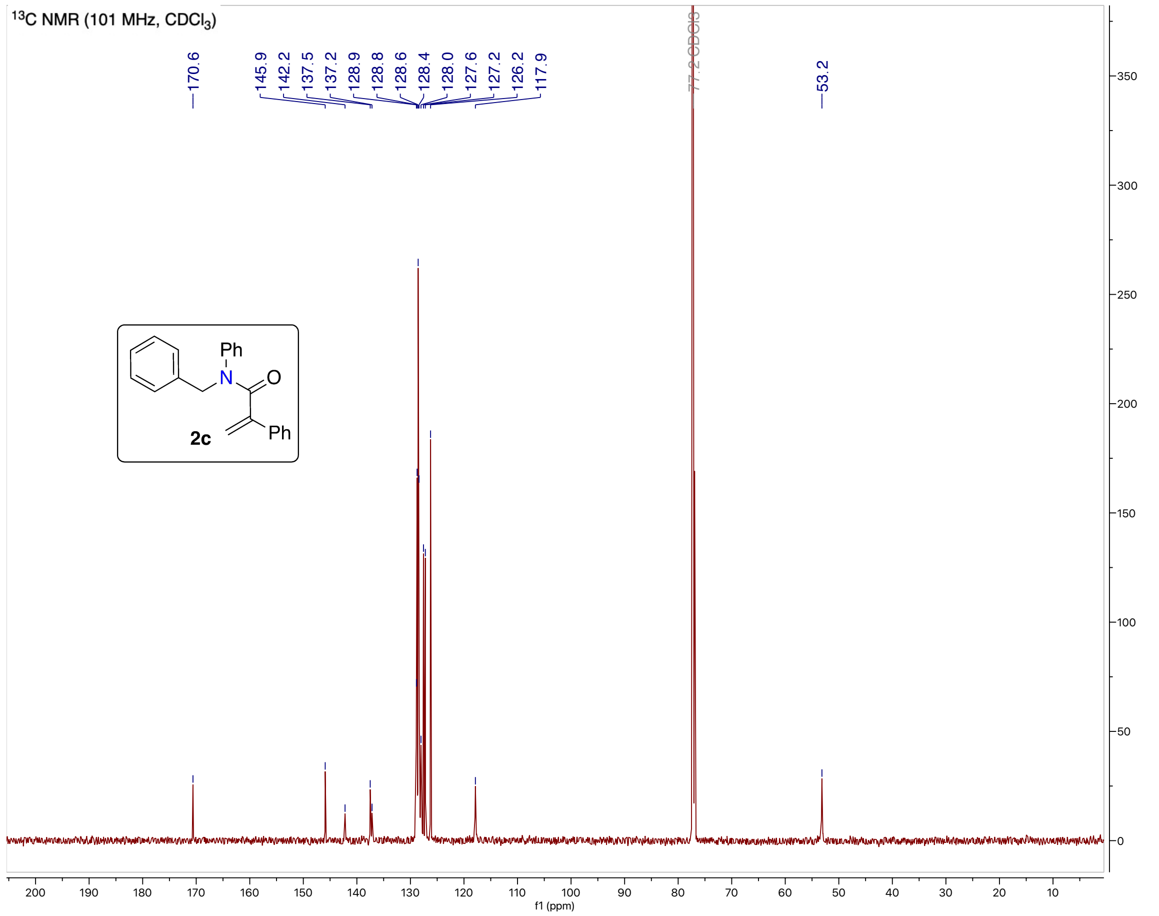
13C NMR (101 MHz, CDCl3)
170.6
145.9
142.2
137.5
137.2
128.9
128.8
128.6
128.4
128.0
127.6
127.2
126.2
117.9
77.2 CDCl3
53.2
Ph
N
O
2c
Ph
f1 (ppm)

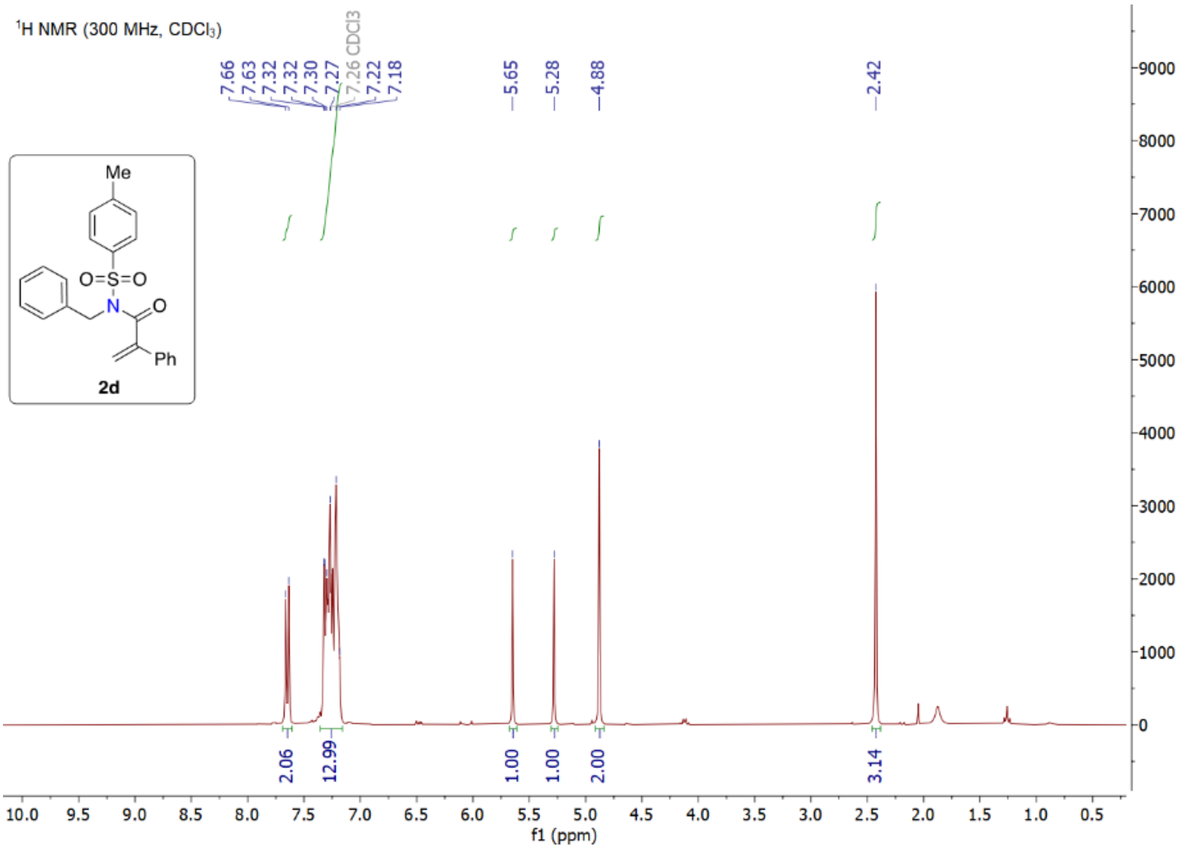

1H NMR (300 MHz, CDCl3)
7.66 7.63 7.32 7.32 7.30 7.27 7.26 CDCl3 7.22 7.18
5.65
5.28
4.88
2.42
Me
O=S=O
N
O
Ph
2d
2.06
12.99
1.00
1.00
2.00
3.14
f1 (ppm)

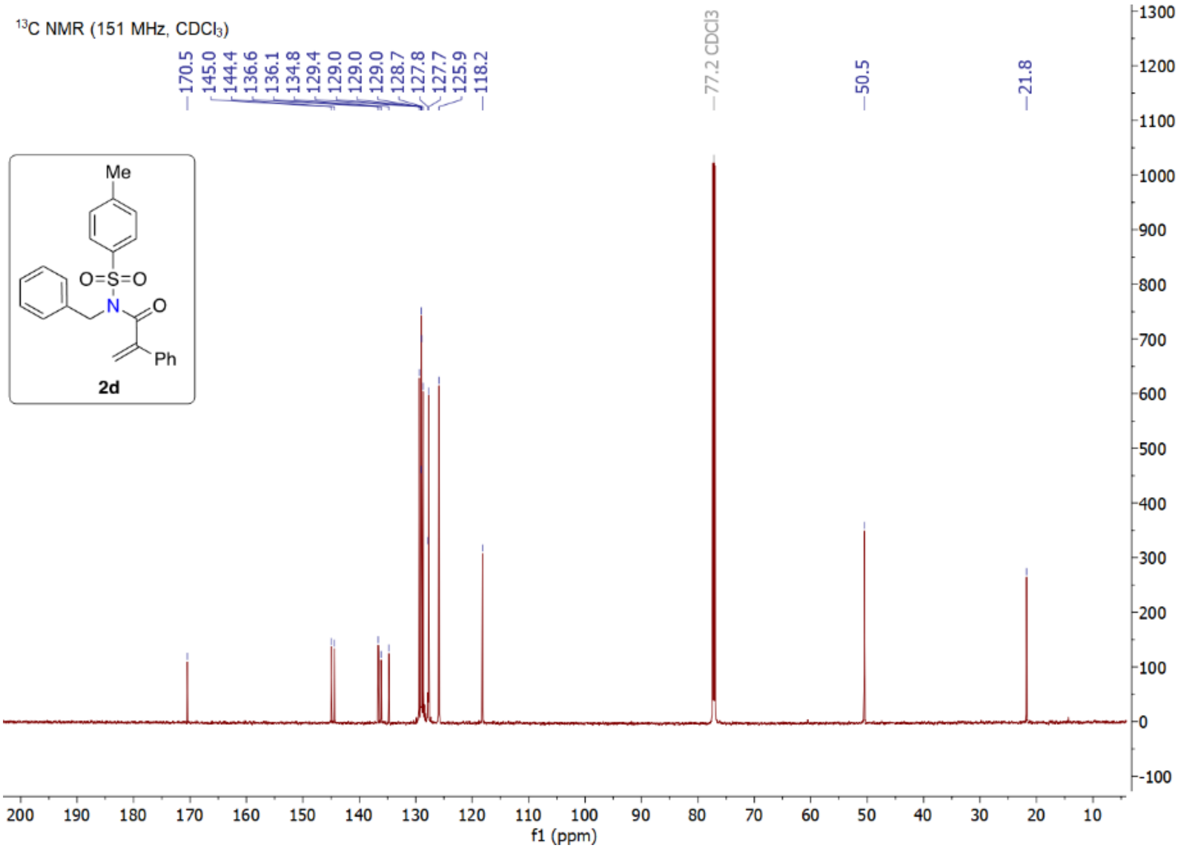

13C NMR (151 MHz, CDCl3)
170.5 145.0 144.4 136.6 136.1 134.8 129.4 129.0 129.0 129.0 128.7 127.8 127.7 125.9 118.2
77.2 CDCl3
50.5
21.8
Me
O=S=O
N
O
Ph
2d
f1 (ppm)

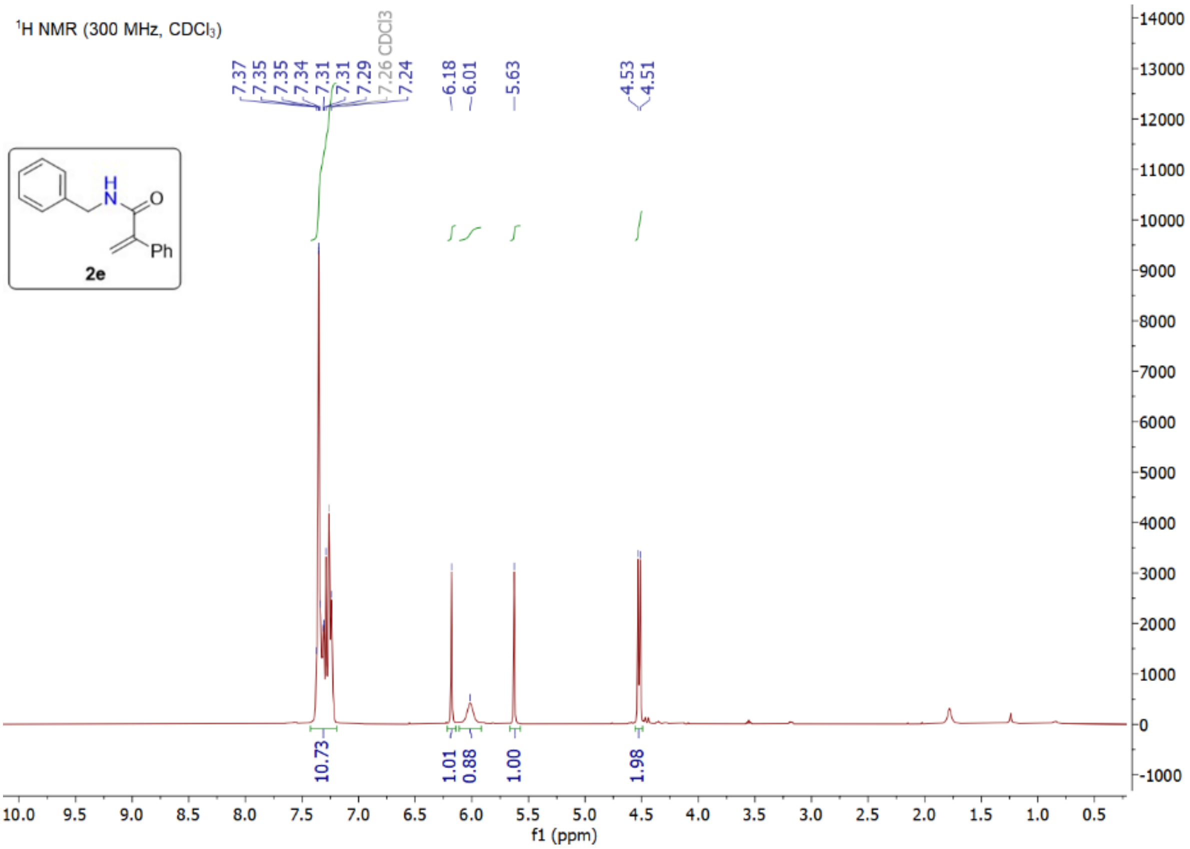

1H NMR (300 MHz, CDCl3)
7.37
7.35
7.35
7.34
7.31
7.31
7.29
7.26 CDCl3
7.24
6.18
6.01
5.63
4.53
4.51
2e
Ph
10.73
1.01
0.88
1.00
1.98
f1 (ppm)

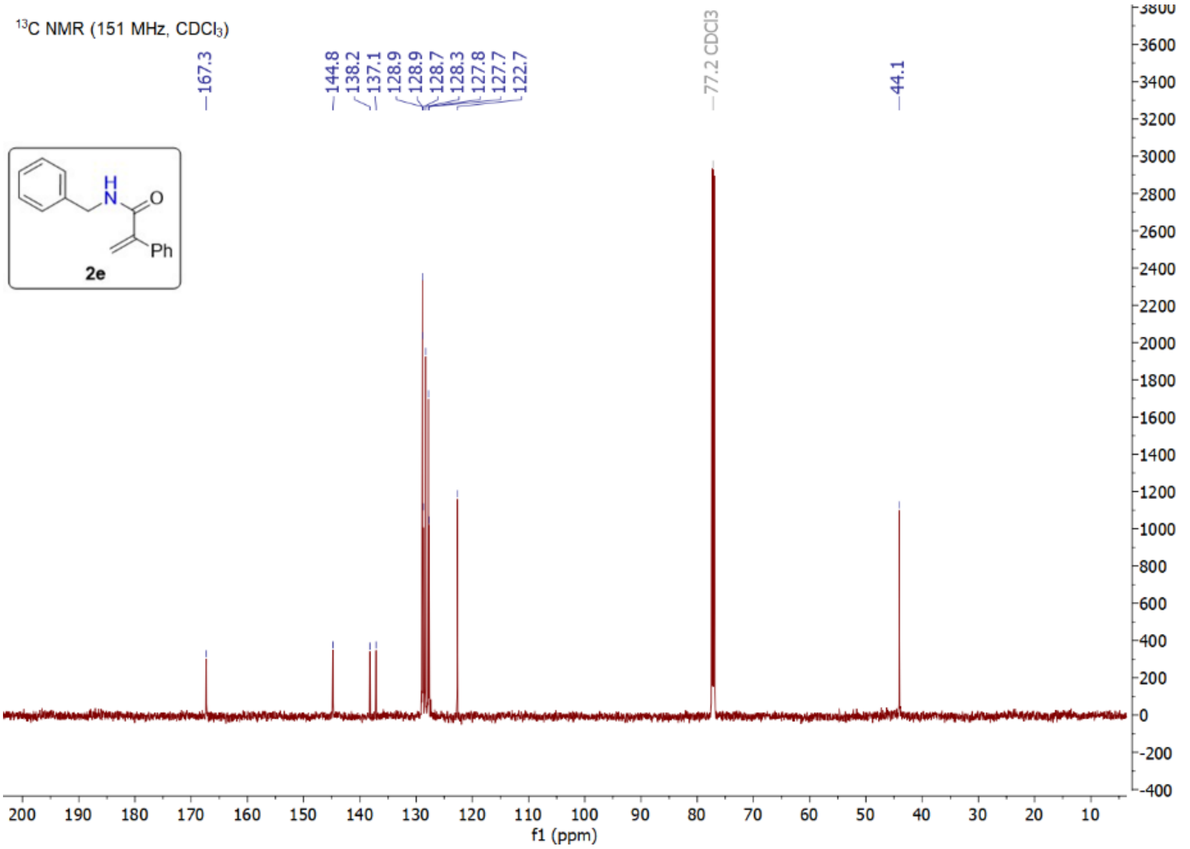

13C NMR (151 MHz, CDCl3)
167.3
144.8
138.2
137.1
128.9
128.9
128.7
128.3
127.8
127.7
122.7
77.2 CDCl3
44.1
2e
Ph
f1 (ppm)

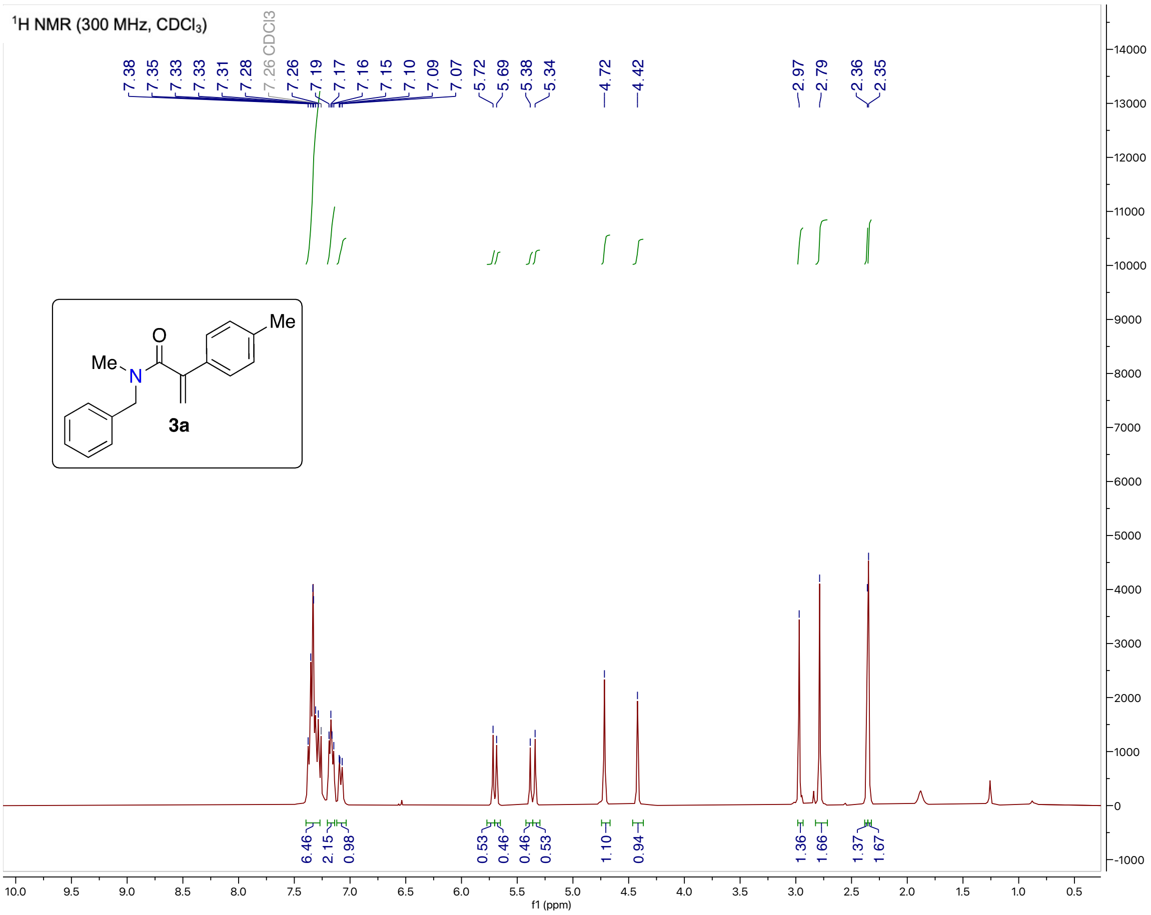

1H NMR (300 MHz, CDCl3)
7.38 7.35 7.33 7.33 7.31 7.28 7.26 CDCl3 7.26 7.19 7.17 7.16 7.15 7.10 7.09 7.07 5.72 5.69 5.38 5.34 4.72 4.42 2.97 2.79 2.36 2.35
6.46 2.15 0.98 0.53 0.46 0.46 0.53 1.10 0.94 1.36 1.66 1.37 1.67
Me
O
Me
N
3a
f1 (ppm)

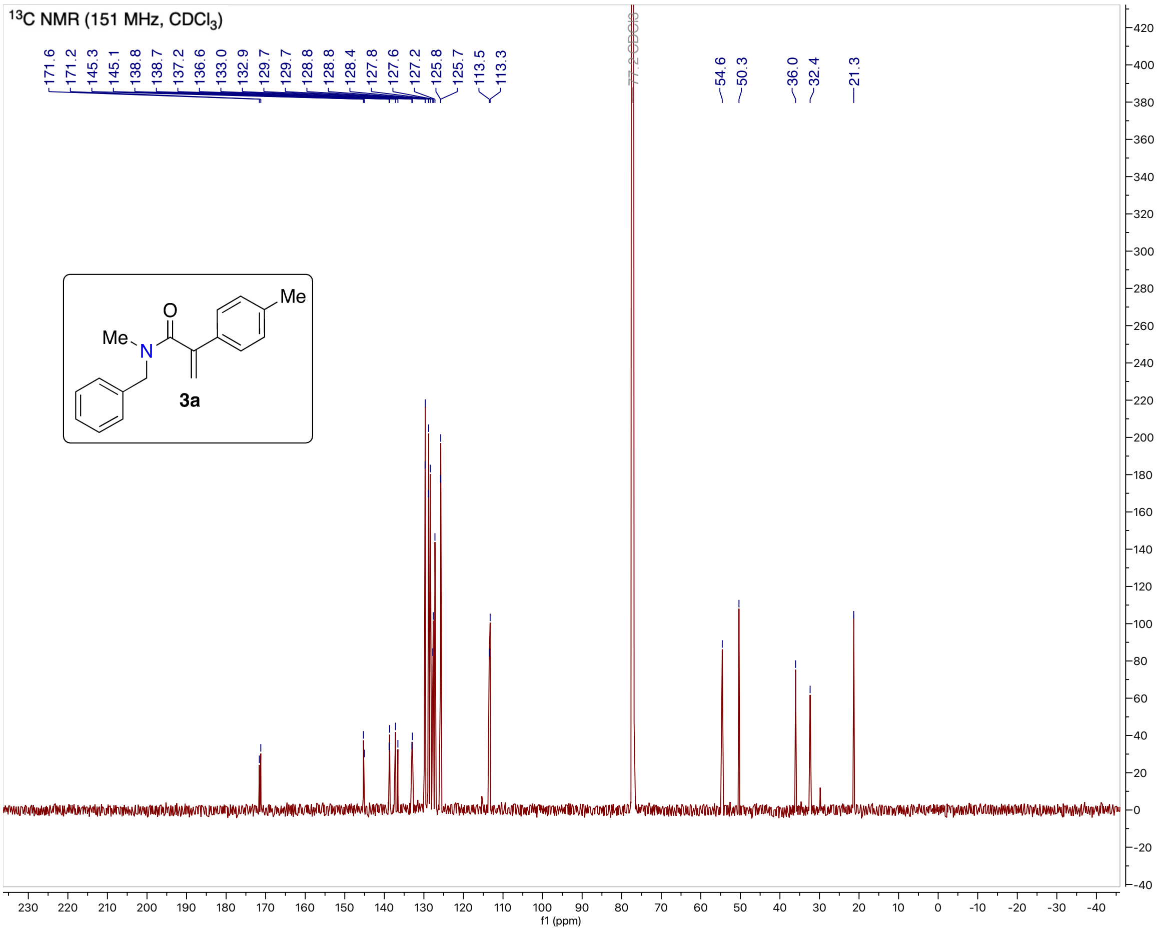

13C NMR (151 MHz, CDCl3)
171.6 171.2 145.3 145.1 138.8 138.7 137.2 136.6 133.0 132.9 129.7 129.7 128.8 128.8 128.4 127.8 127.6 127.2 125.8 125.7 113.5 113.3
77.2 CDCl3
54.6 50.3 36.0 32.4 21.3
Me
O
Me
N
3a
f1 (ppm)

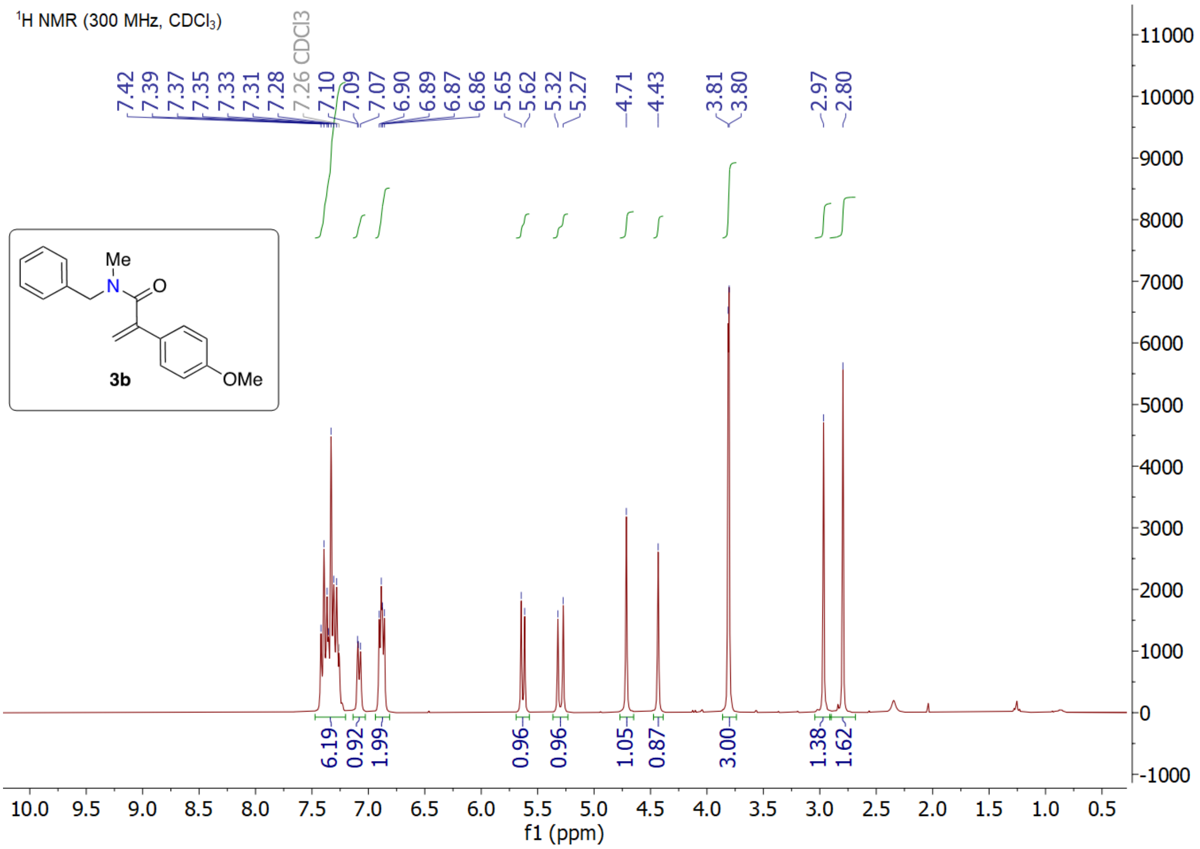

1H NMR (300 MHz, CDCl3)
7.42
7.39
7.37
7.35
7.33
7.31
7.28
7.26 CDCl3
7.10
7.09
7.07
6.90
6.89
6.87
6.86
5.65
5.62
5.32
5.27
4.71
4.43
3.81
3.80
2.97
2.80
Me
N
O
3b
OMe
6.19
0.92
1.99
0.96
0.96
1.05
0.87
3.00
1.38
1.62
f1 (ppm)

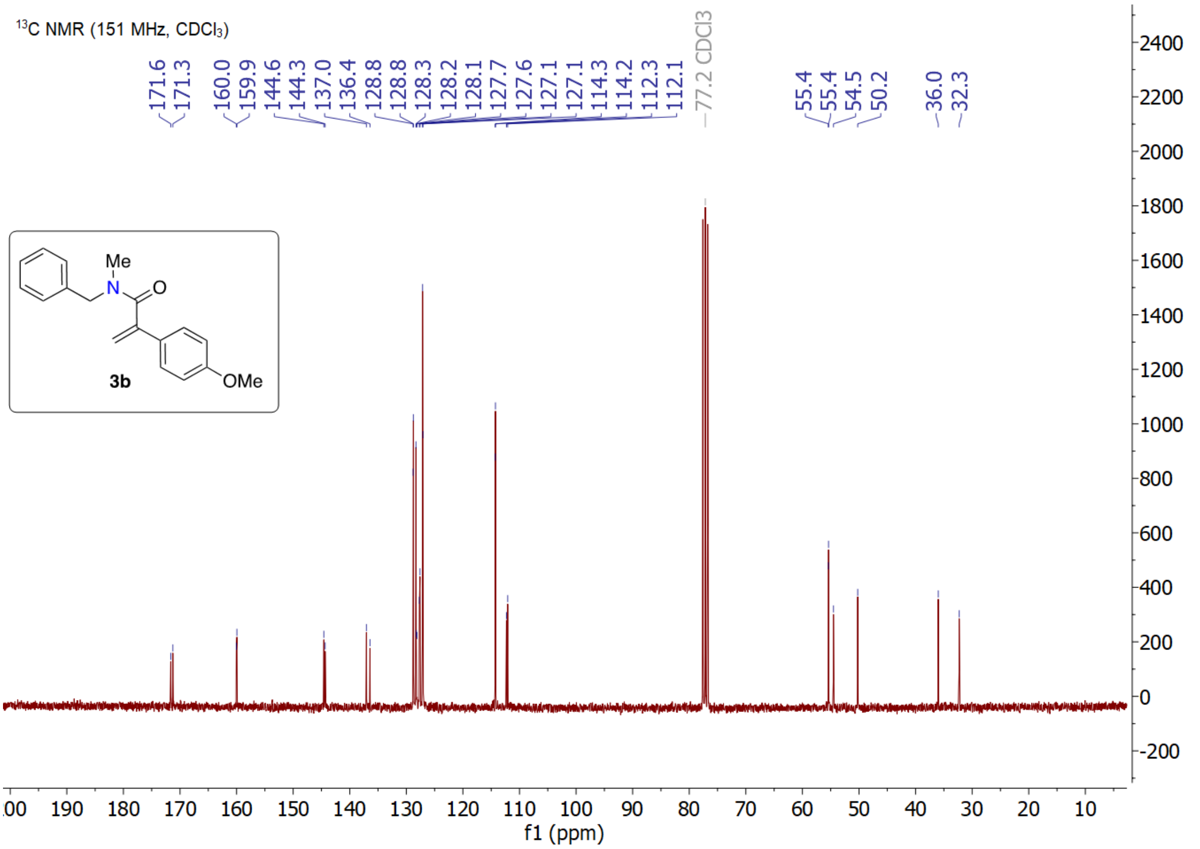

13C NMR (151 MHz, CDCl3)
171.6
171.3
160.0
159.9
144.6
144.3
137.0
136.4
128.8
128.8
128.3
128.2
128.1
127.7
127.6
127.1
127.1
114.3
114.2
112.3
112.1
77.2 CDCl3
55.4
55.4
54.5
50.2
36.0
32.3
Me
N
O
3b
OMe
f1 (ppm)

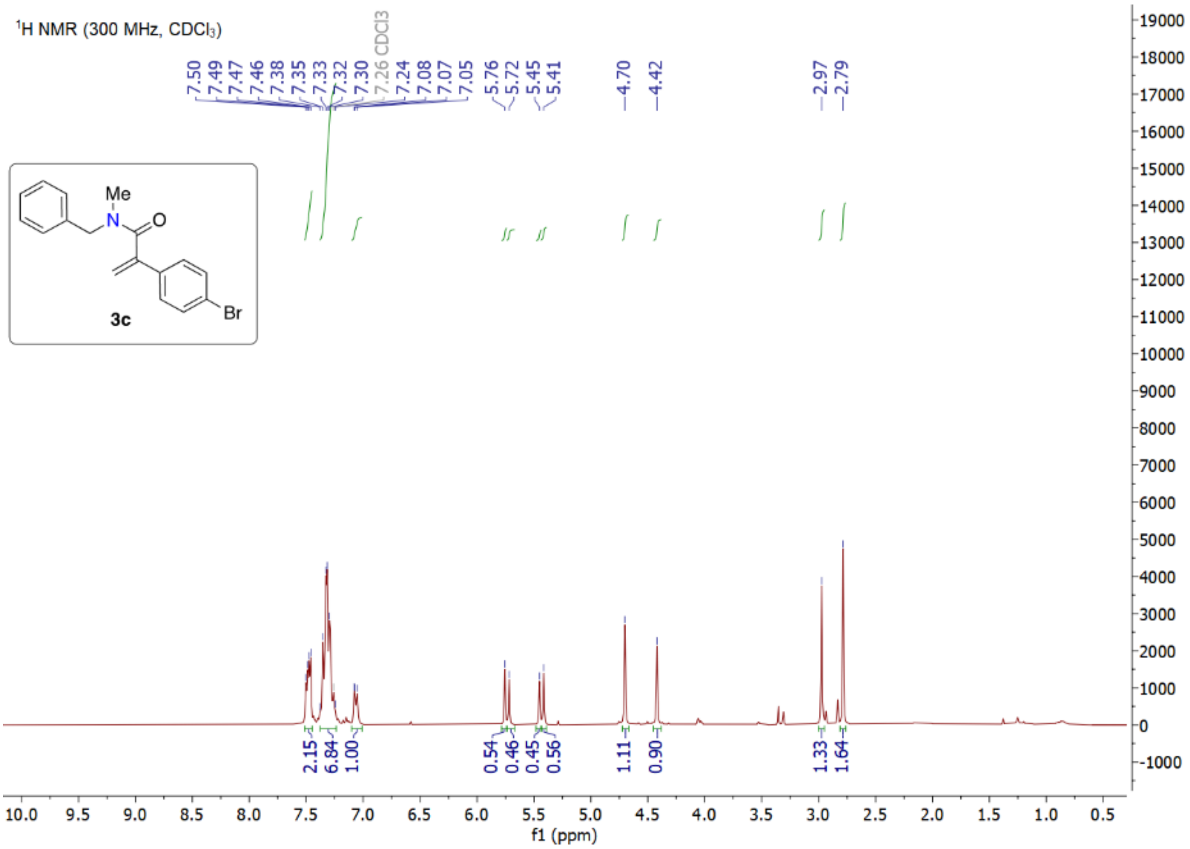

^{1}H NMR (300 MHz, CDCl3)
7.50
7.49
7.47
7.46
7.38
7.35
7.33
7.32
7.30
7.26 CDCl3
7.24
7.08
7.07
7.05
5.76
5.72
5.45
5.41
4.70
4.42
2.97
2.79
Me
N
O
Br
3c
2.15
6.84
1.00
0.54
0.46
0.45
0.56
1.11
0.90
1.33
1.64
f1 (ppm)

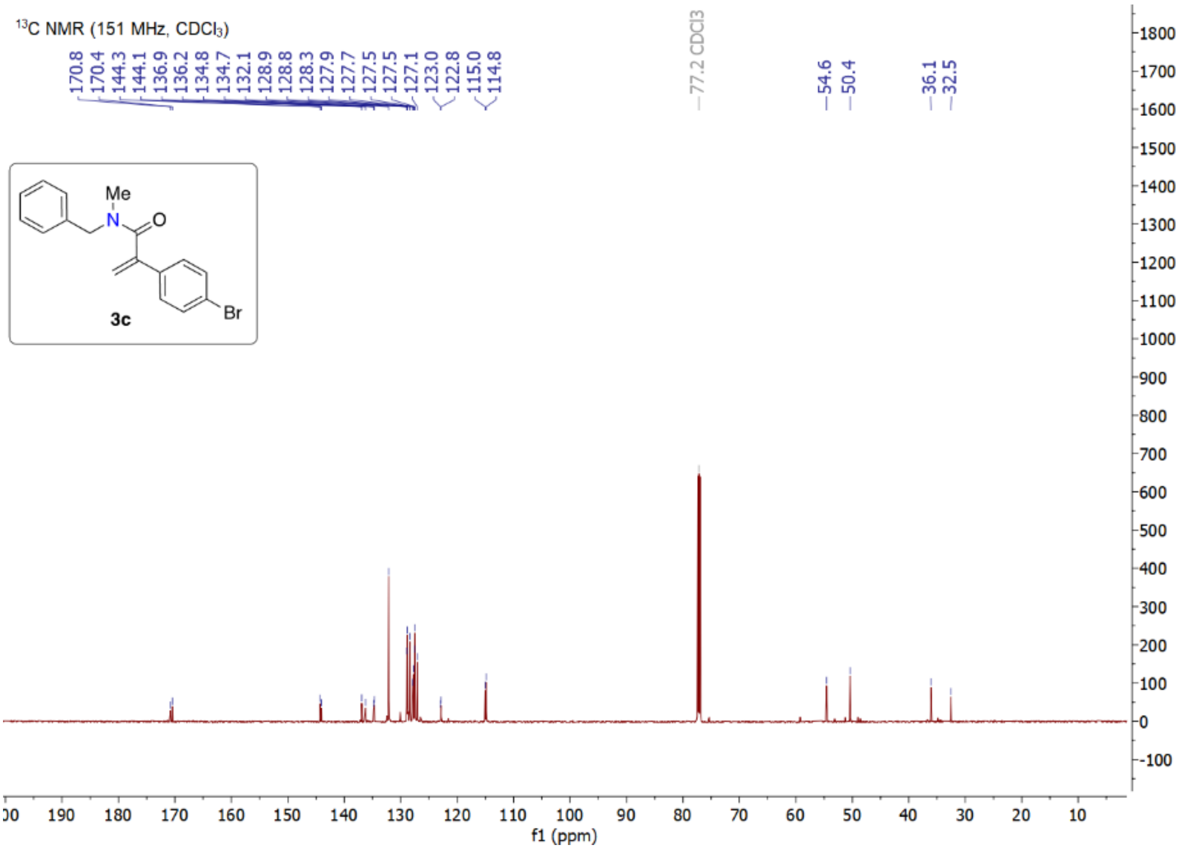

^{13}C NMR (151 MHz, CDCl3)
170.8
170.4
144.3
144.1
136.9
136.2
134.8
134.7
132.1
128.9
128.8
128.3
127.9
127.7
127.5
127.5
127.1
123.0
122.8
115.0
114.8
77.2 CDCl3
54.6
50.4
36.1
32.5
Me
N
O
Br
3c
f1 (ppm)

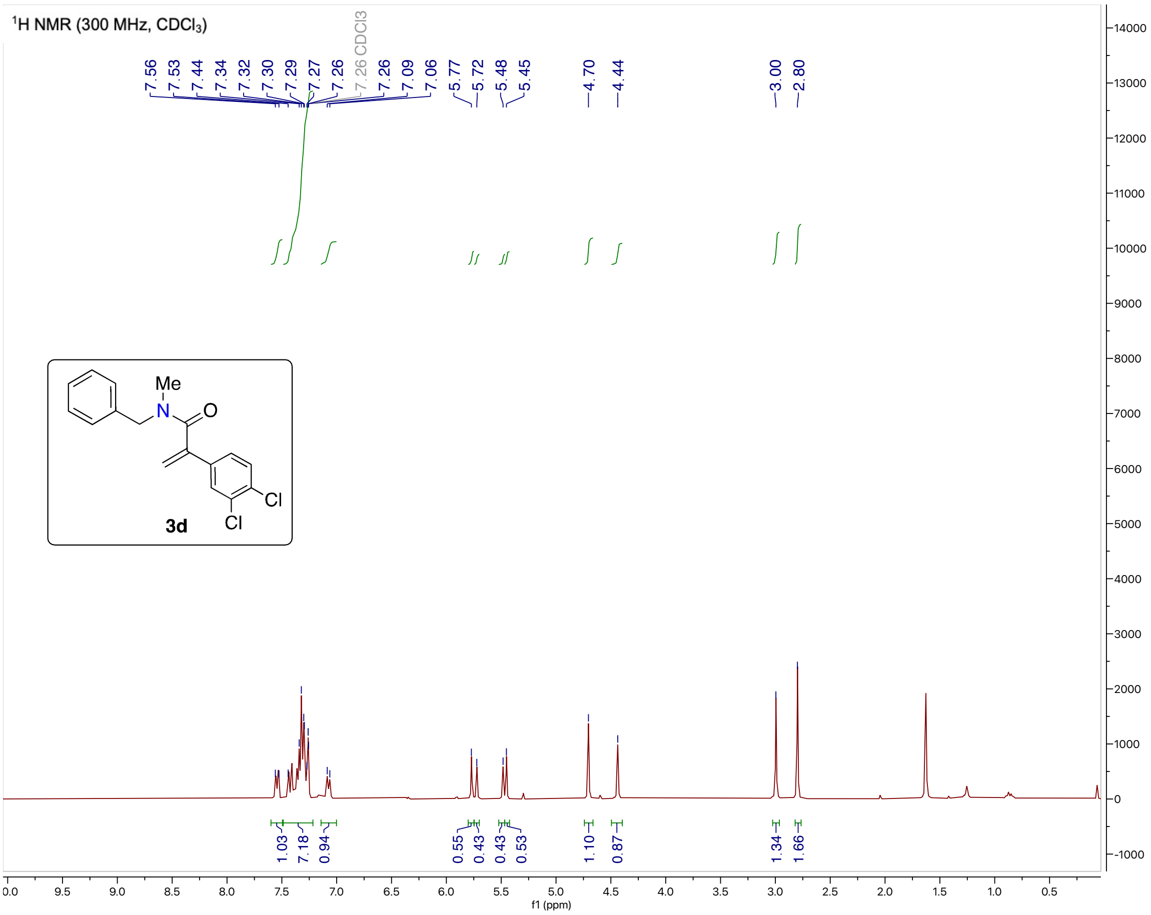

^{1}H NMR (300 MHz, CDCl$_3$)
7.56
7.53
7.44
7.34
7.32
7.30
7.29
7.27
7.26
7.26 CDCl3
7.26
7.09
7.06
5.77
5.72
5.48
5.45
4.70
4.44
3.00
2.80
1.03
7.18
0.94
0.55
0.43
0.43
0.53
1.10
0.87
1.34
1.66
Me
N
O
Cl
Cl
3d
f1 (ppm)

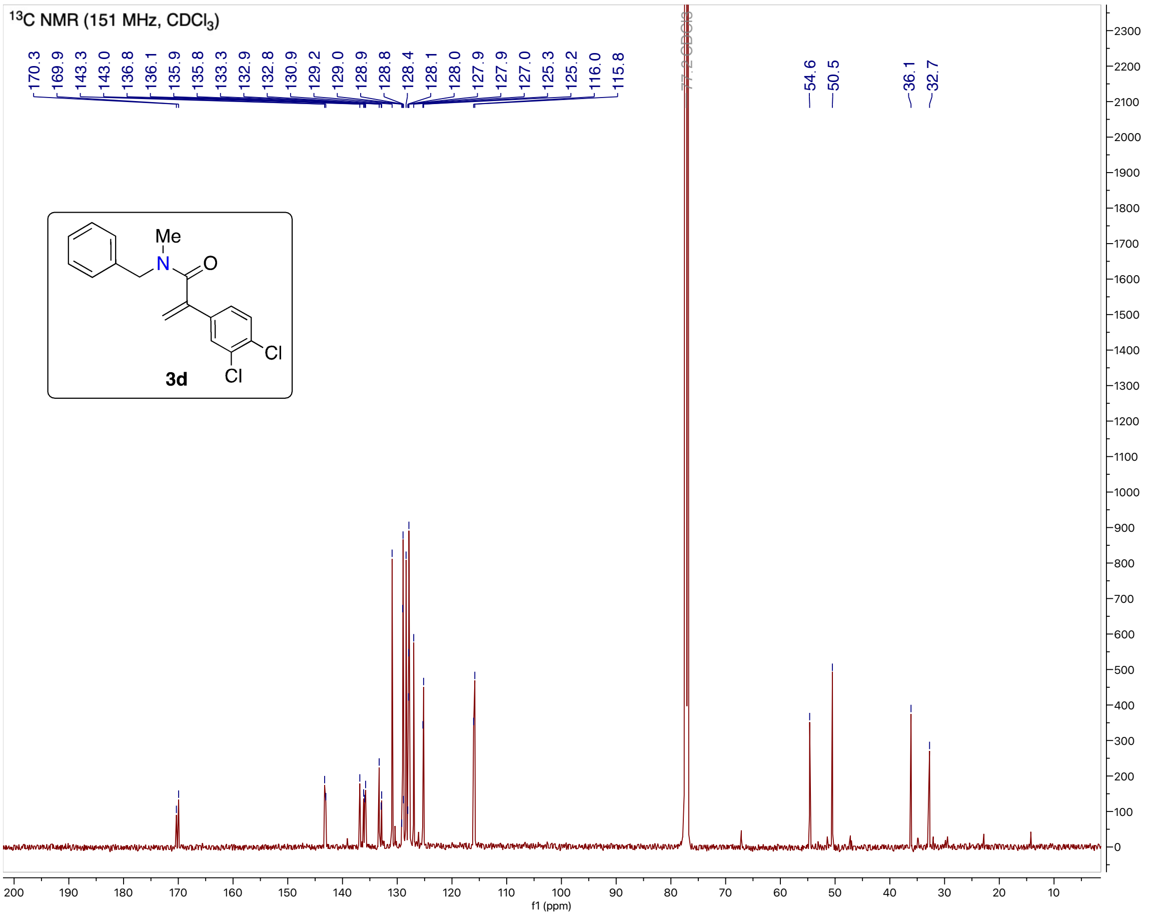

^{13}C NMR (151 MHz, CDCl$_3$)
170.3
169.9
143.3
143.0
136.8
136.1
135.9
135.8
133.3
132.9
132.8
130.9
129.2
129.0
128.9
128.8
128.4
128.1
128.0
127.9
127.9
127.0
125.3
125.2
116.0
115.8
77.2 CDCl3
54.6
50.5
36.1
32.7
Me
N
O
Cl
Cl
3d
f1 (ppm)

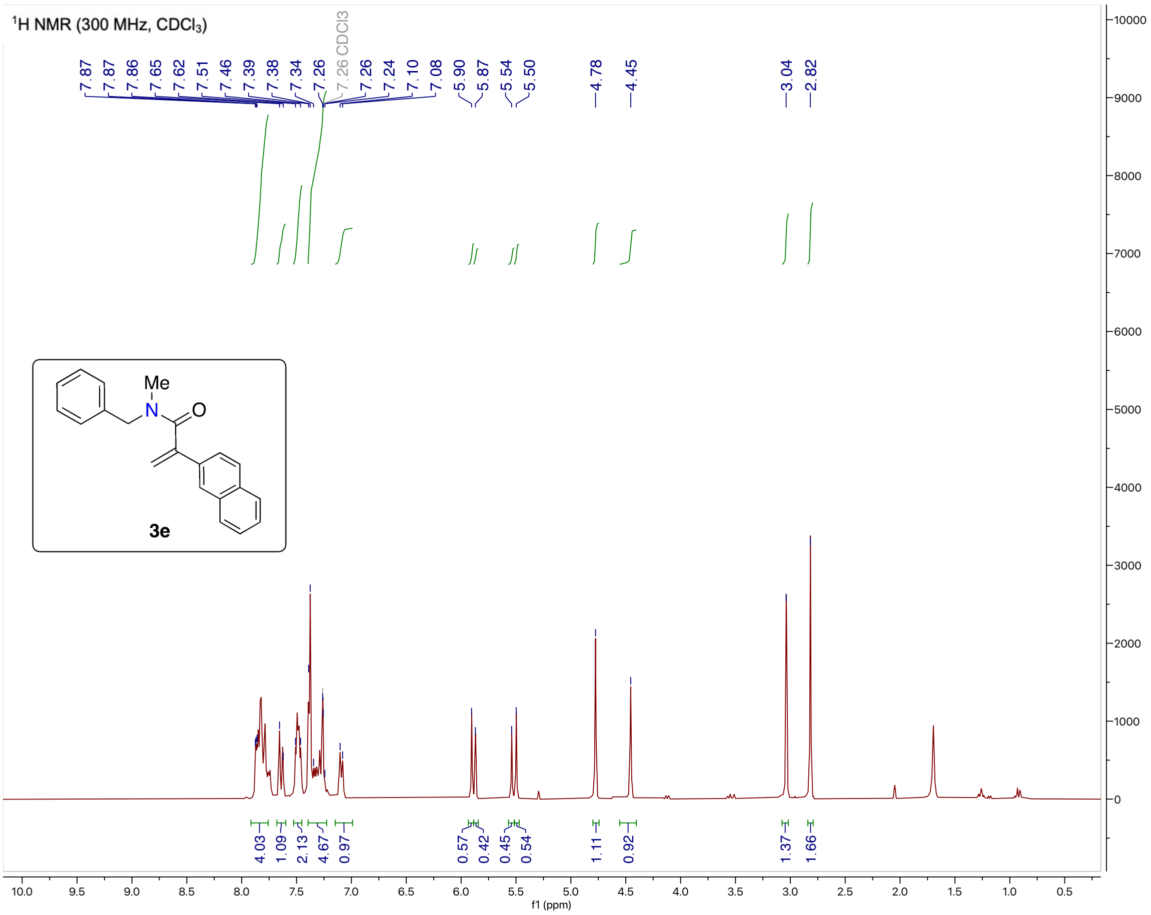

1H NMR (300 MHz, CDCl3)
7.26 CDCl3
7.87 7.87 7.86 7.65 7.62 7.51 7.46 7.39 7.38 7.34 7.26 7.26 7.24 7.10 7.08 5.90 5.87 5.54 5.50 4.78 4.45 3.04 2.82
4.03 1.09 2.13 4.67 0.97 0.57 0.42 0.45 0.54 1.11 0.92 1.37 1.66
Me
N
O
3e
f1 (ppm)

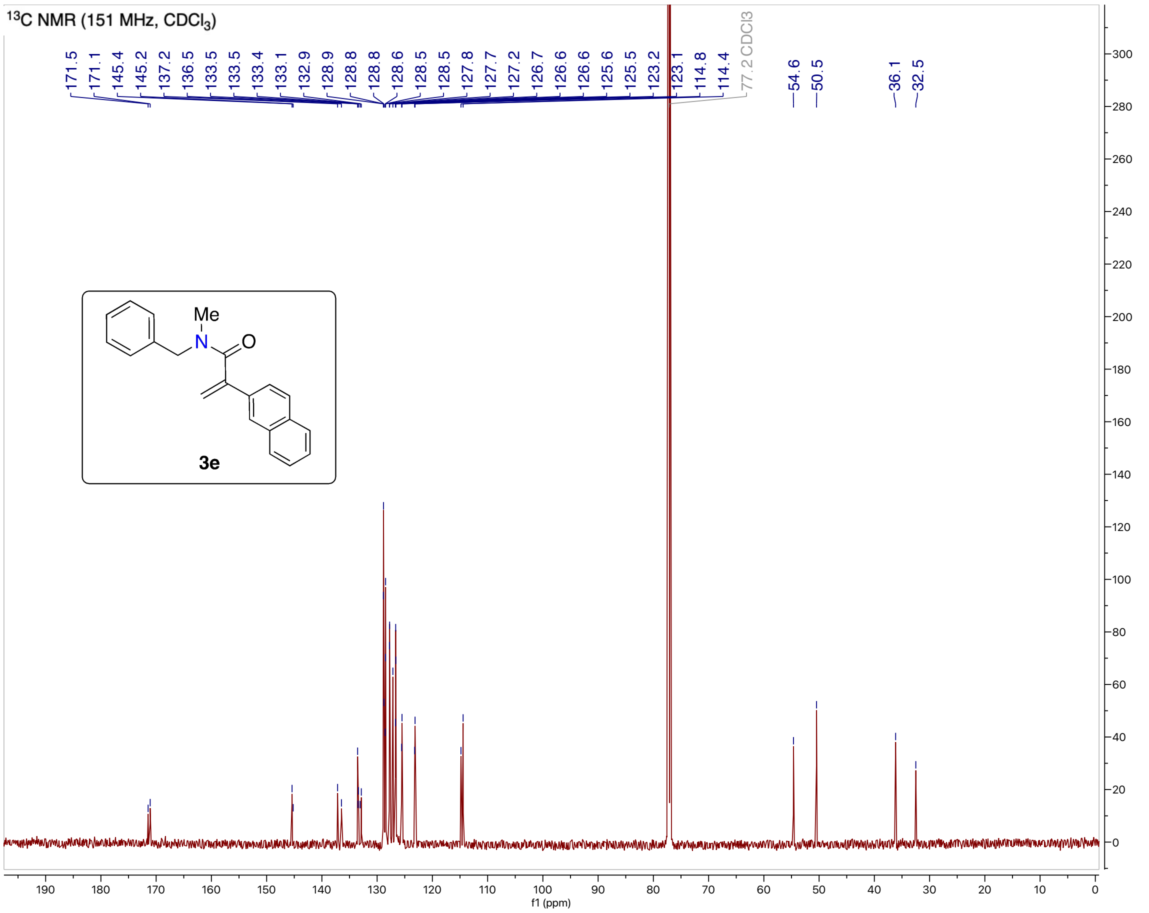

13C NMR (151 MHz, CDCl3)
77.2 CDCl3
171.5 171.1 145.4 145.2 137.2 136.5 133.5 133.5 133.4 133.1 132.9 128.9 128.8 128.8 128.6 128.5 128.5 127.8 127.7 127.2 126.7 126.6 126.6 125.6 125.5 123.2 123.1 114.8 114.4 54.6 50.5 36.1 32.5
Me
N
O
3e
f1 (ppm)

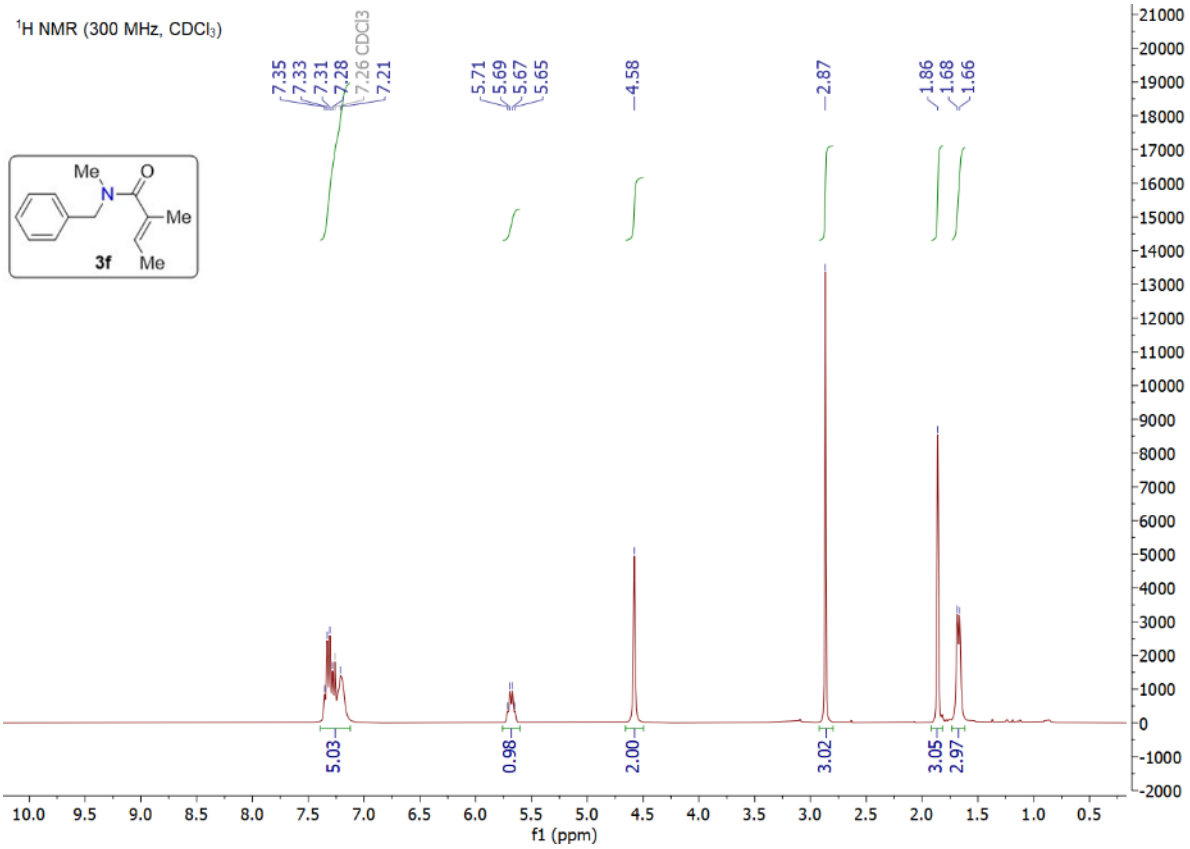

1H NMR (300 MHz, CDCl3)
7.35
7.33
7.31
7.28
7.26 CDCl3
7.21
5.71
5.69
5.67
5.65
4.58
2.87
1.86
1.68
1.66
Me
O
N
Me
3f
Me
5.03
0.98
2.00
3.02
3.05
2.97
f1 (ppm)

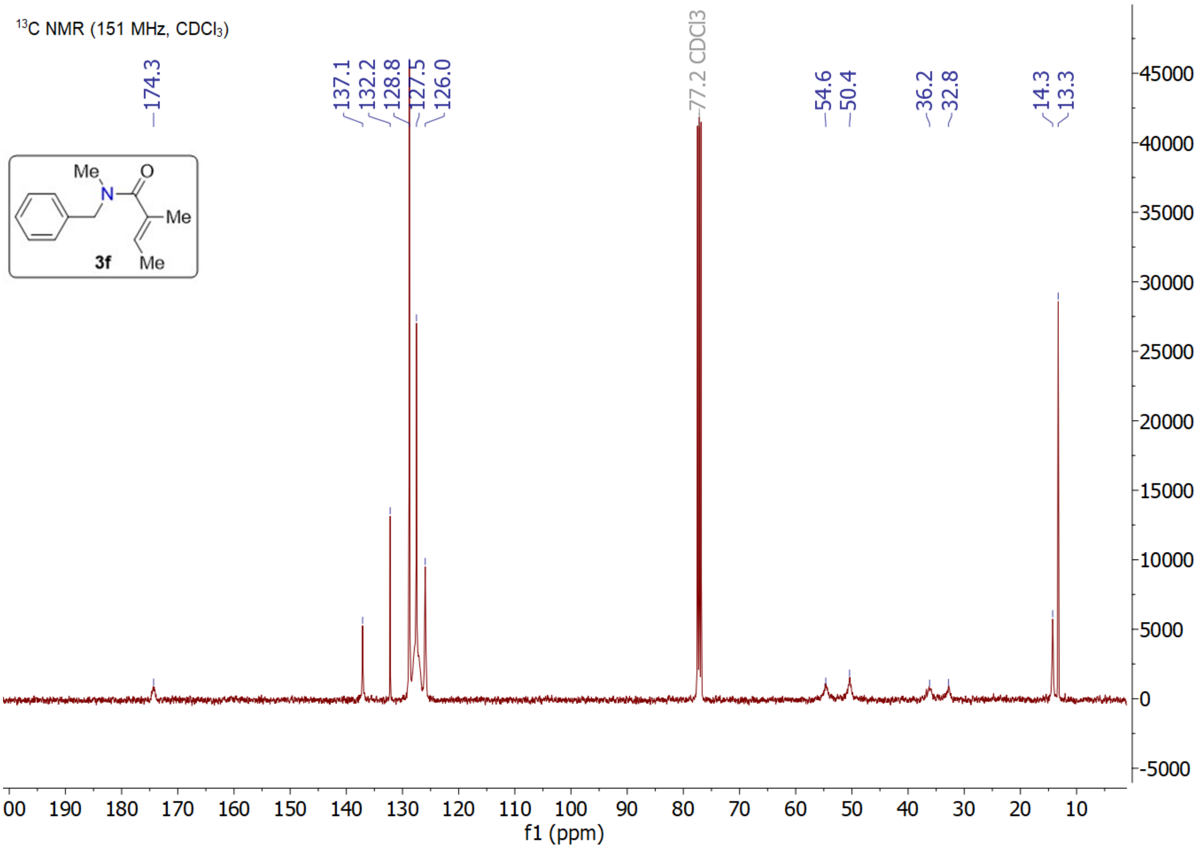

13C NMR (151 MHz, CDCl3)
174.3
137.1
132.2
128.8
127.5
126.0
77.2 CDCl3
54.6
50.4
36.2
32.8
14.3
13.3
Me
O
N
Me
3f
Me
f1 (ppm)

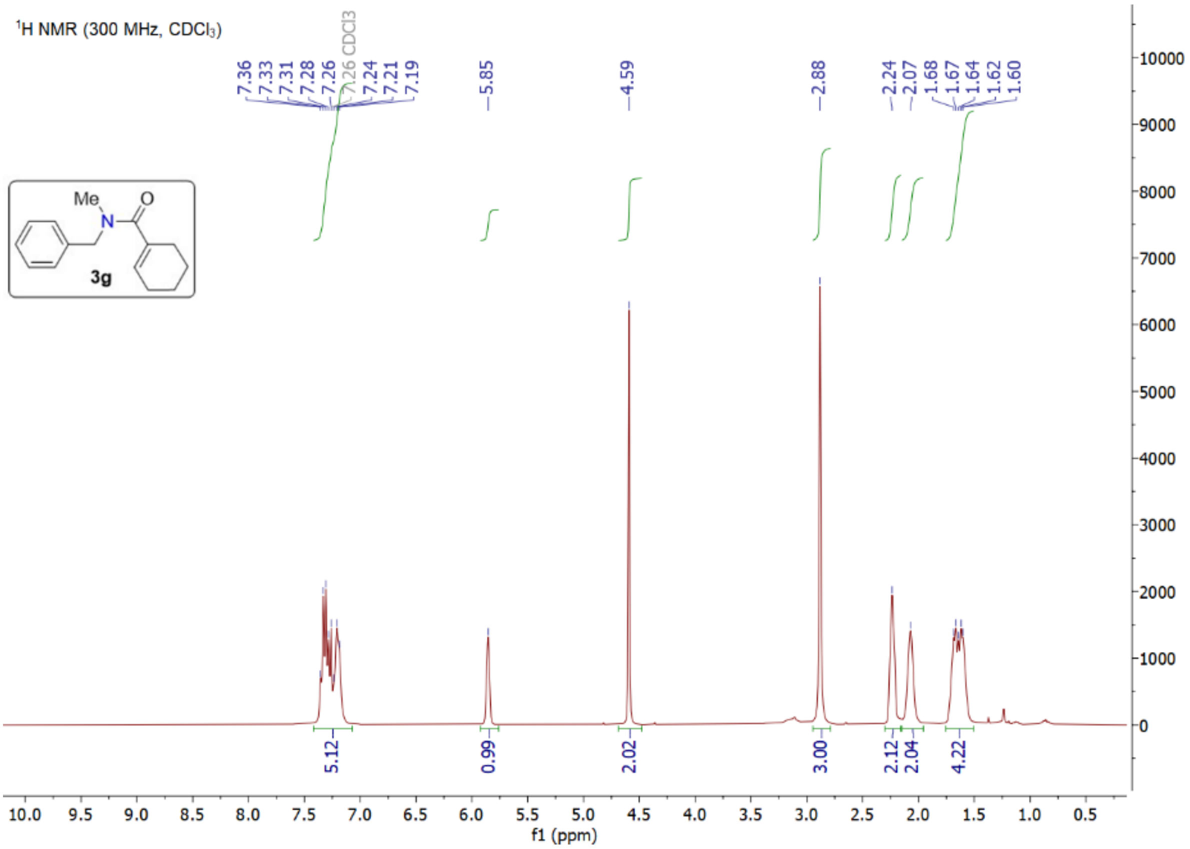

1H NMR (300 MHz, CDCl3)
7.36
7.33
7.31
7.28
7.26
7.26 CDCl3
7.24
7.21
7.19
5.85
4.59
2.88
2.24
2.07
1.68
1.67
1.64
1.62
1.60
Me
N
O
3g
5.12
0.99
2.02
3.00
2.12
2.04
4.22
f1 (ppm)

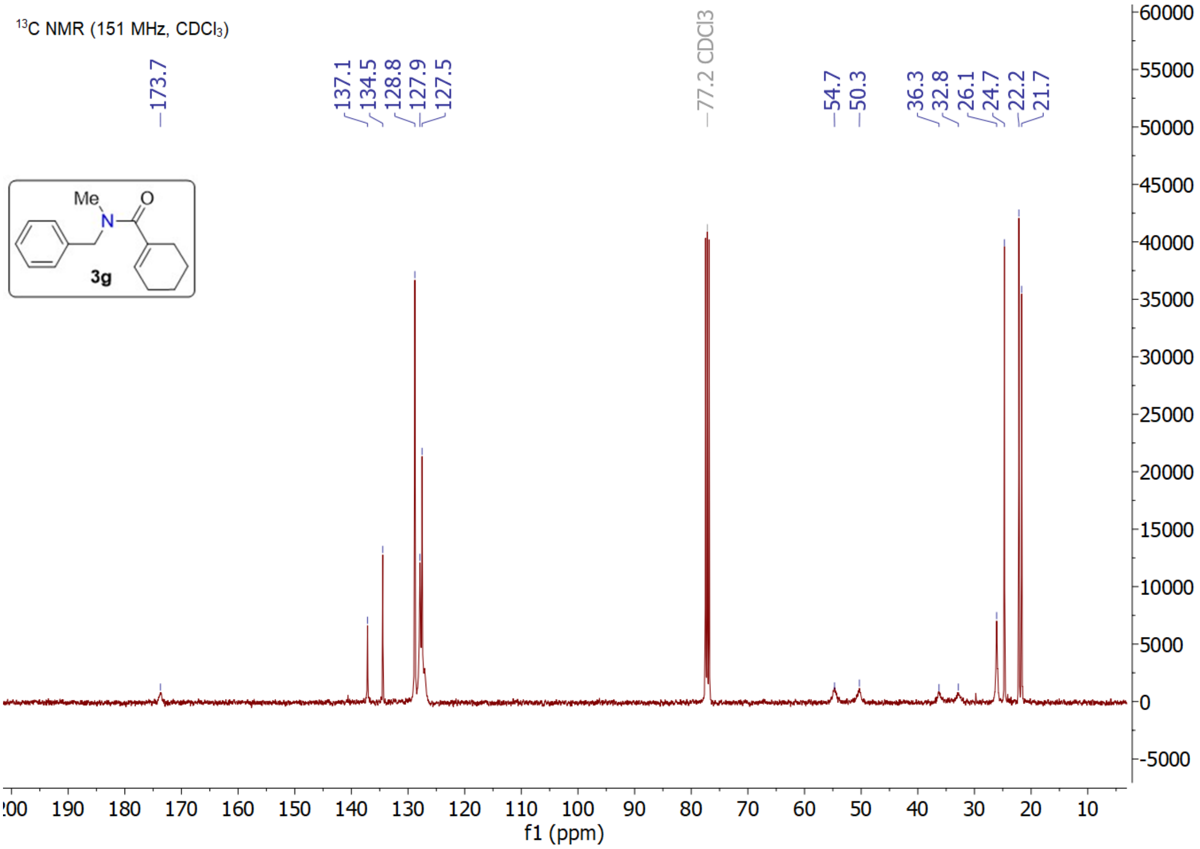

13C NMR (151 MHz, CDCl3)
173.7
137.1
134.5
128.8
127.9
127.5
77.2 CDCl3
54.7
50.3
36.3
32.8
26.1
24.7
22.2
21.7
Me
N
O
3g
f1 (ppm)

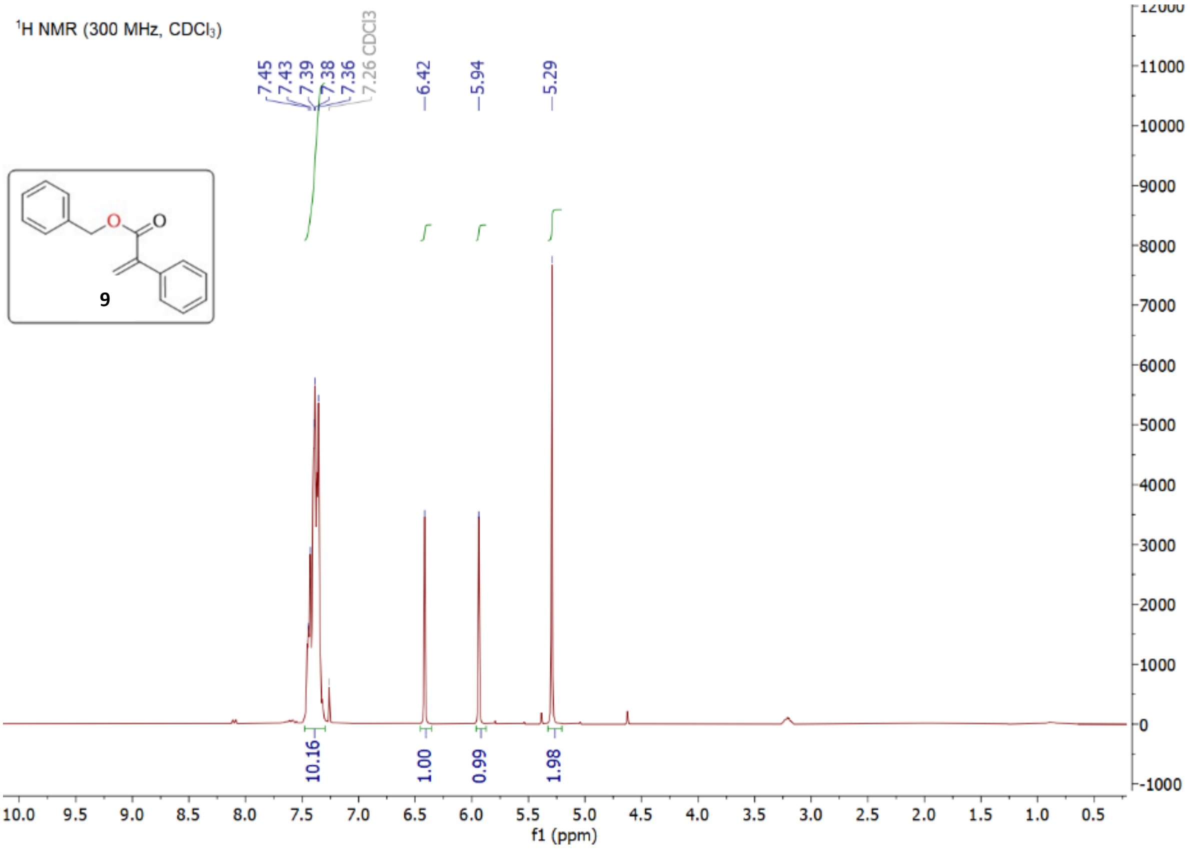

1H NMR (300 MHz, CDCl3)
7.45
7.43
7.39
7.38
7.36
7.26 CDCl3
6.42
5.94
5.29
9
10.16
1.00
0.99
1.98
f1 (ppm)

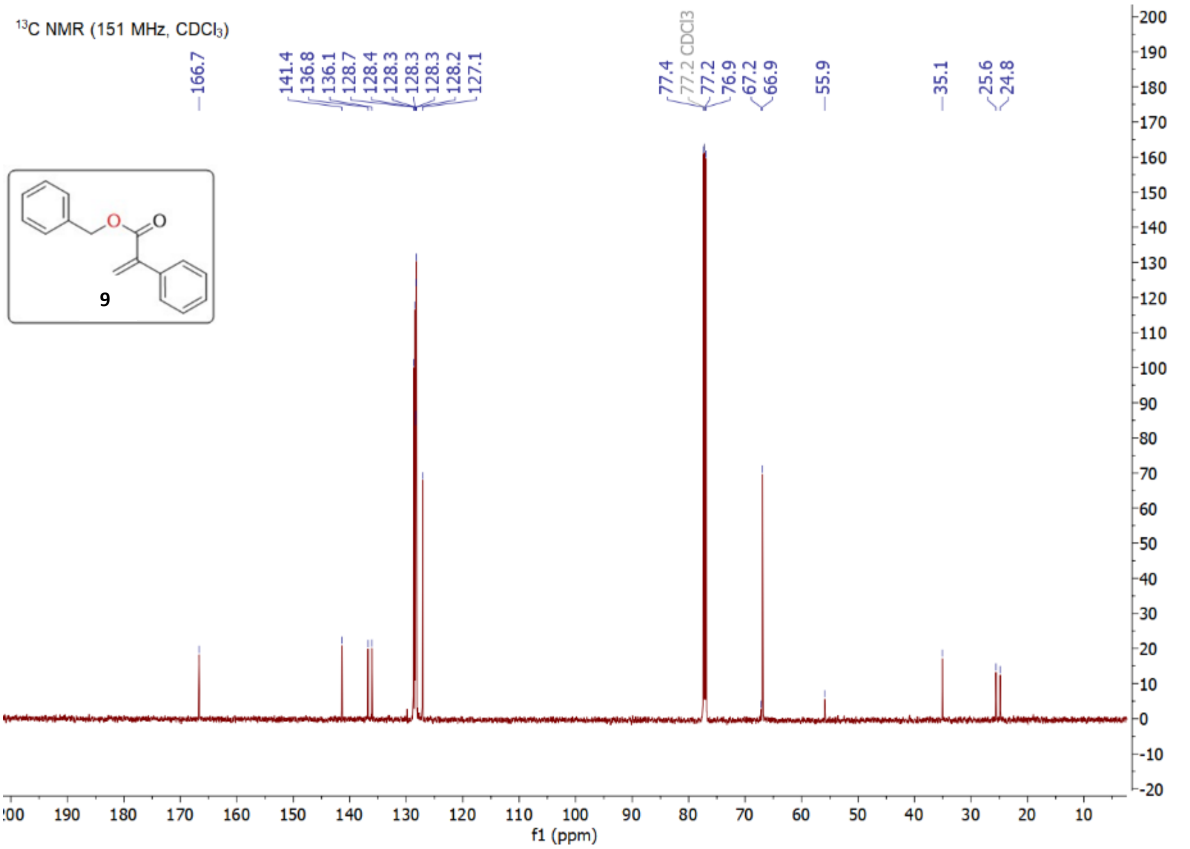

13C NMR (151 MHz, CDCl3)
166.7
141.4
136.8
136.1
128.7
128.4
128.3
128.3
128.3
128.2
127.1
77.4
77.2 CDCl3
77.2
76.9
67.2
66.9
55.9
35.1
25.6
24.8
9
f1 (ppm)

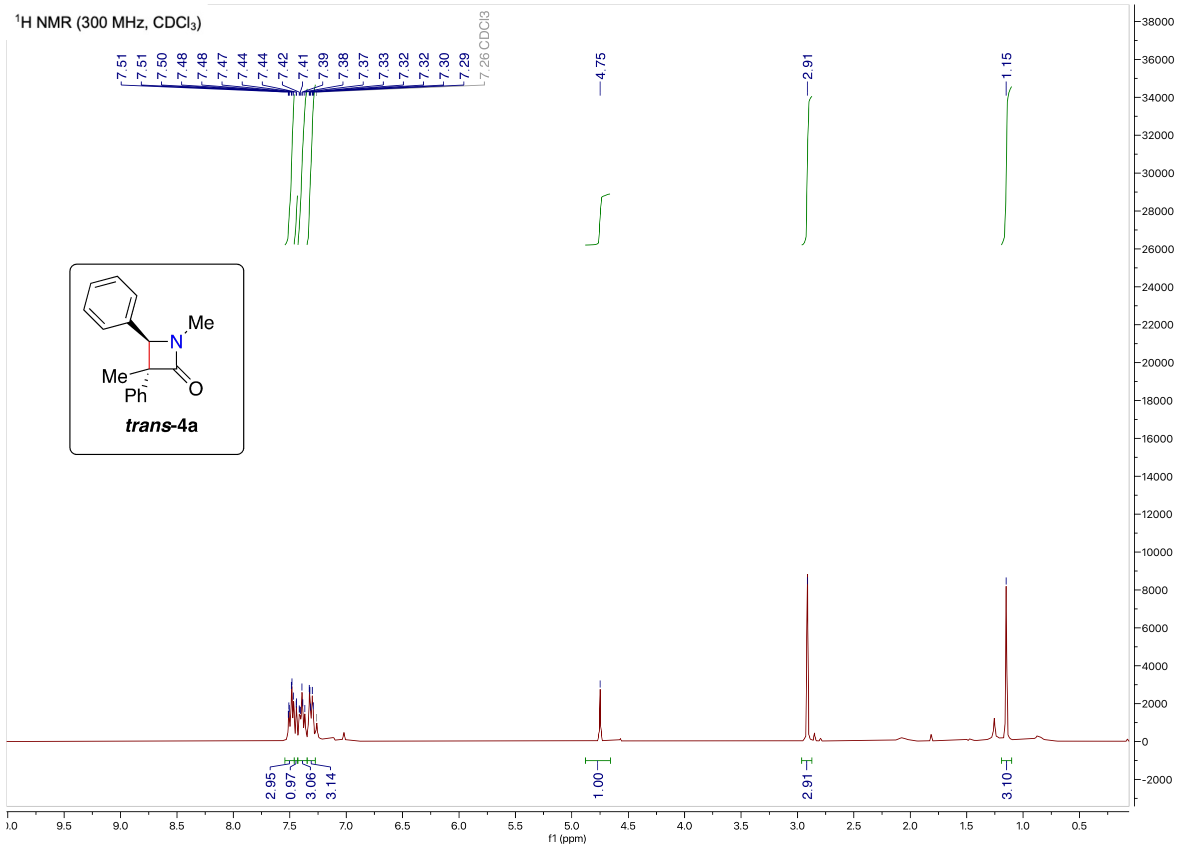

1H NMR (300 MHz, CDCl3)
7.51 7.51 7.50 7.48 7.48 7.47 7.44 7.44 7.42 7.41 7.39 7.38 7.37 7.33 7.32 7.32 7.30 7.29
7.26 CDCl3
4.75
2.91
1.15
Me
N
Me
Ph
O
trans-4a
2.95 0.97 3.06 3.14
1.00
2.91
3.10
f1 (ppm)

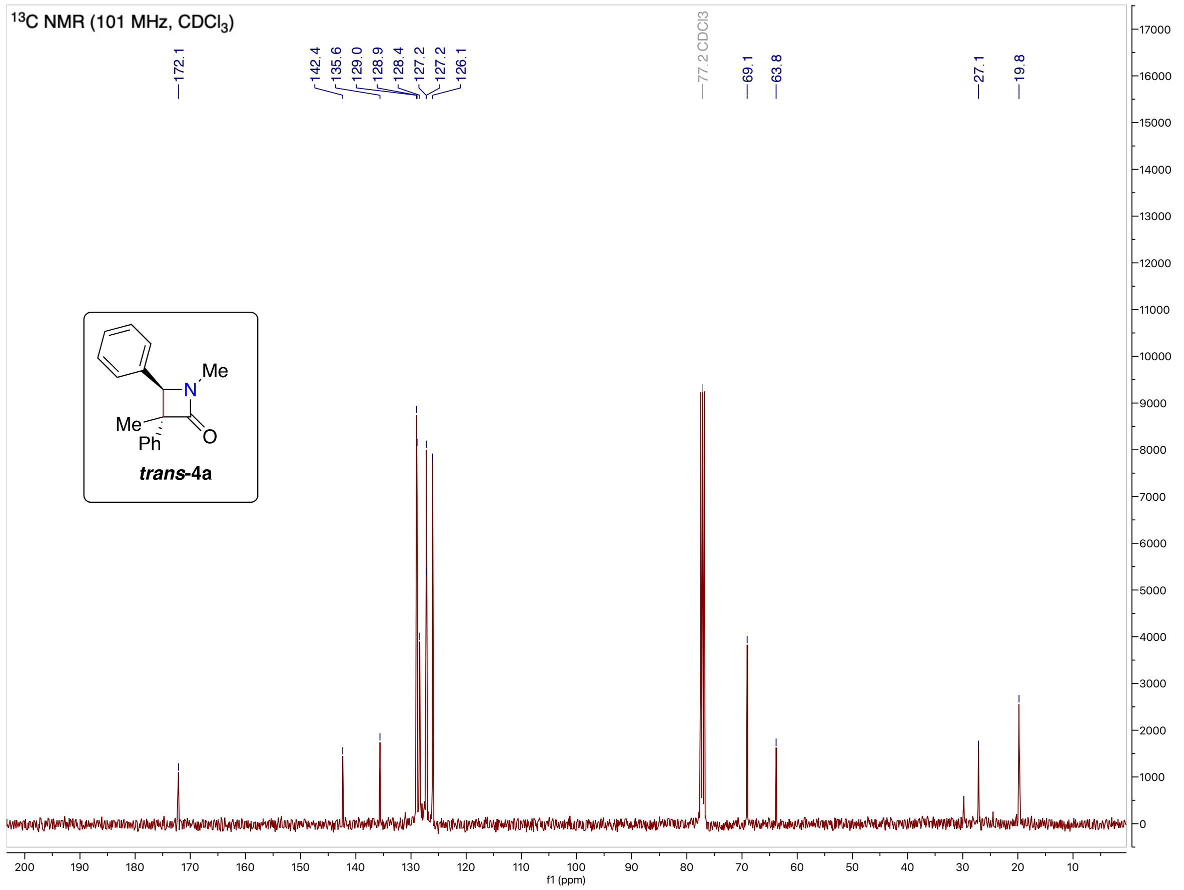

13C NMR (101 MHz, CDCl3)
172.1
142.4 135.6 129.0 128.9 128.4 127.2 127.2 126.1
77.2 CDCl3
69.1
63.8
27.1
19.8
Me
N
Me
Ph
O
trans-4a
f1 (ppm)

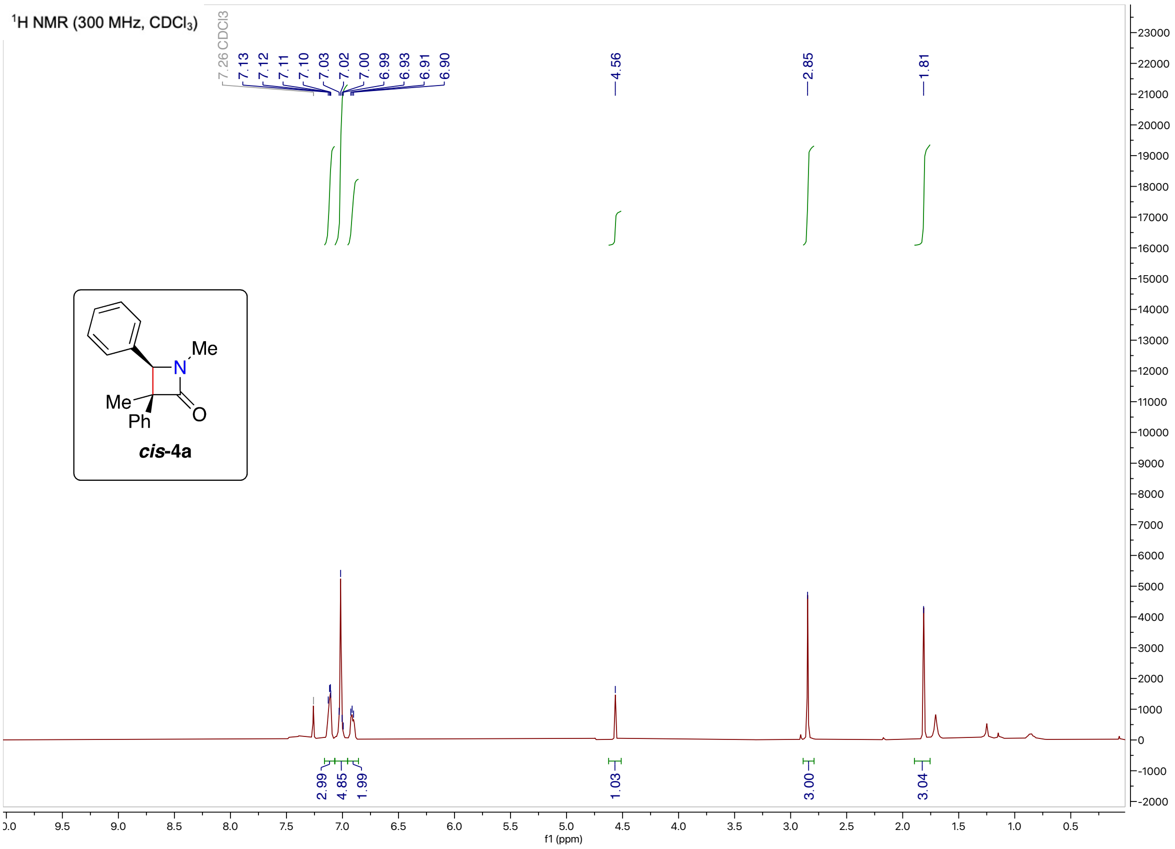

^{1}H NMR (300 MHz, CDCl$_3$)
7.26 CDCl3
7.13
7.12
7.11
7.10
7.03
7.02
7.00
6.99
6.93
6.91
6.90
4.56
2.85
1.81
2.99
4.85
1.99
1.03
3.00
3.04
Me
N
Me
Ph
O
cis-4a
f1 (ppm)

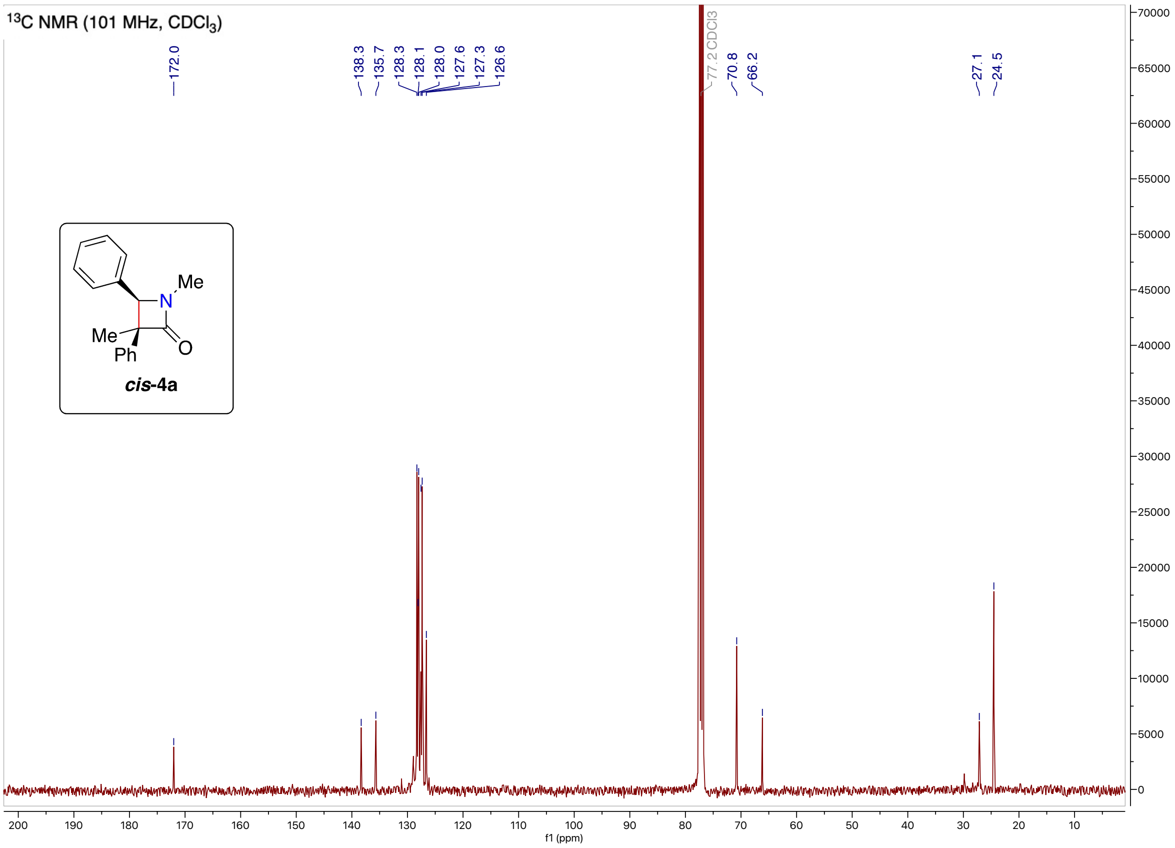

^{13}C NMR (101 MHz, CDCl$_3$)
172.0
138.3
135.7
128.3
128.1
128.0
127.6
127.3
126.6
77.2 CDCl3
70.8
66.2
27.1
24.5
Me
N
Me
Ph
O
cis-4a
f1 (ppm)

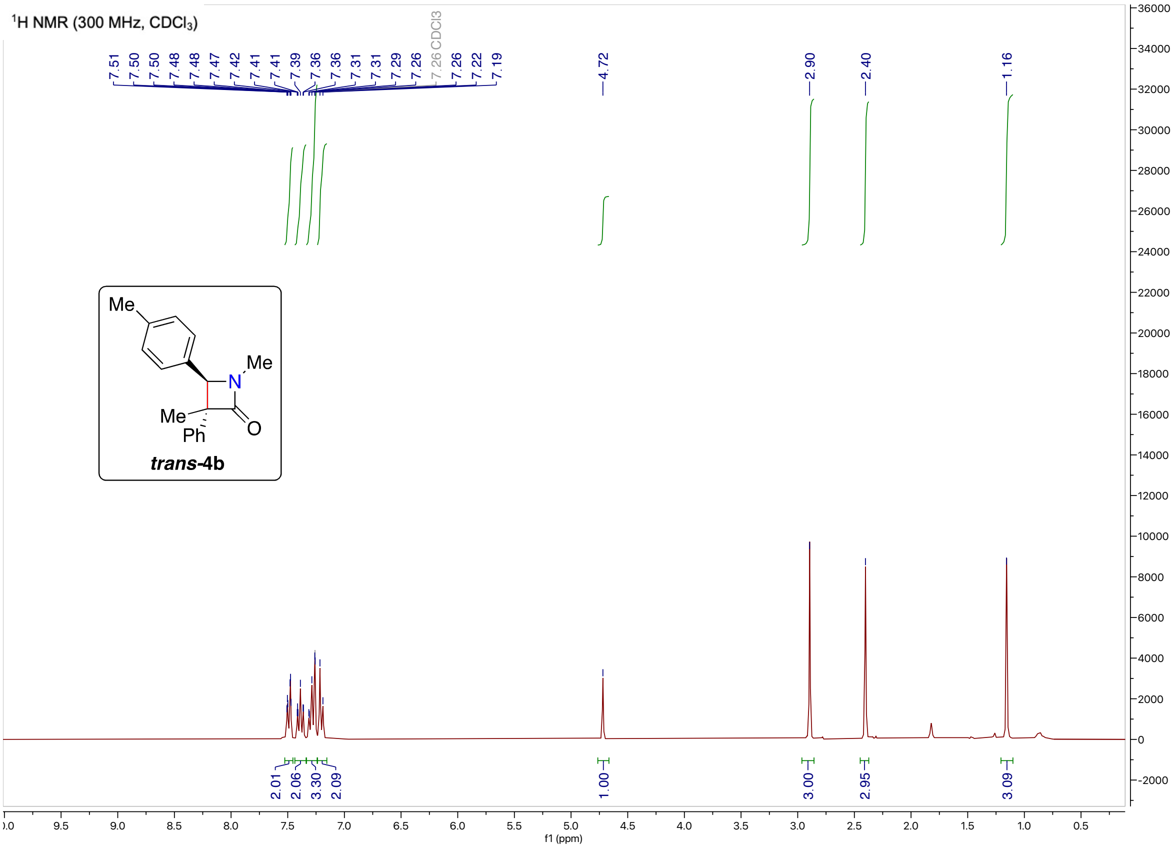

1H NMR (300 MHz, CDCl3)
7.51 7.50 7.50 7.48 7.48 7.47 7.42 7.41 7.41 7.39 7.36 7.36 7.31 7.31 7.29 7.26 7.26 CDCl3 7.26 7.22 7.19
4.72
2.90
2.40
1.16
Me
Me
N
Me
Ph
O
trans-4b
2.01 2.06 3.30 2.09
1.00
3.00
2.95
3.09
f1 (ppm)

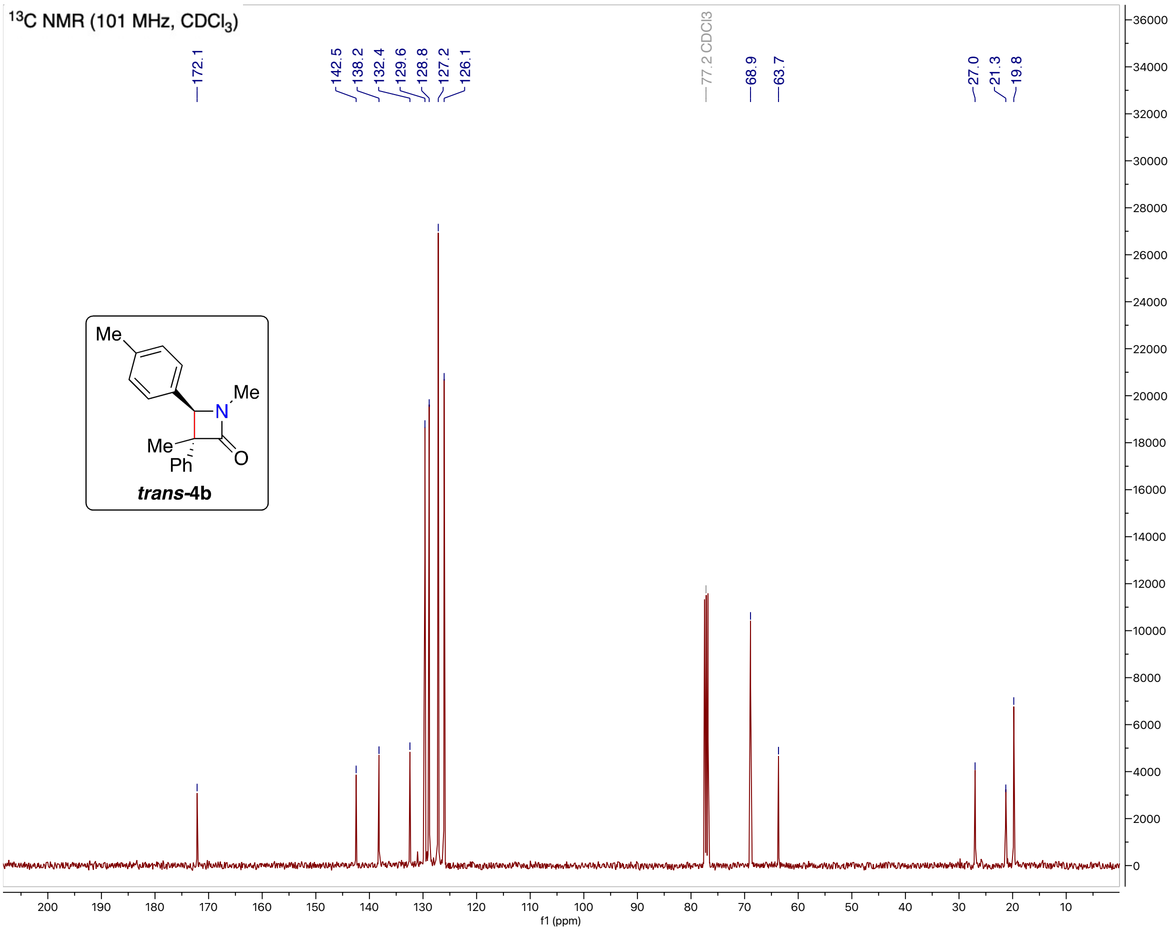

13C NMR (101 MHz, CDCl3)
172.1
142.5 138.2 132.4 129.6 128.8 127.2 126.1
77.2 CDCl3
68.9
63.7
27.0 21.3 19.8
Me
Me
N
Me
Ph
O
trans-4b
f1 (ppm)

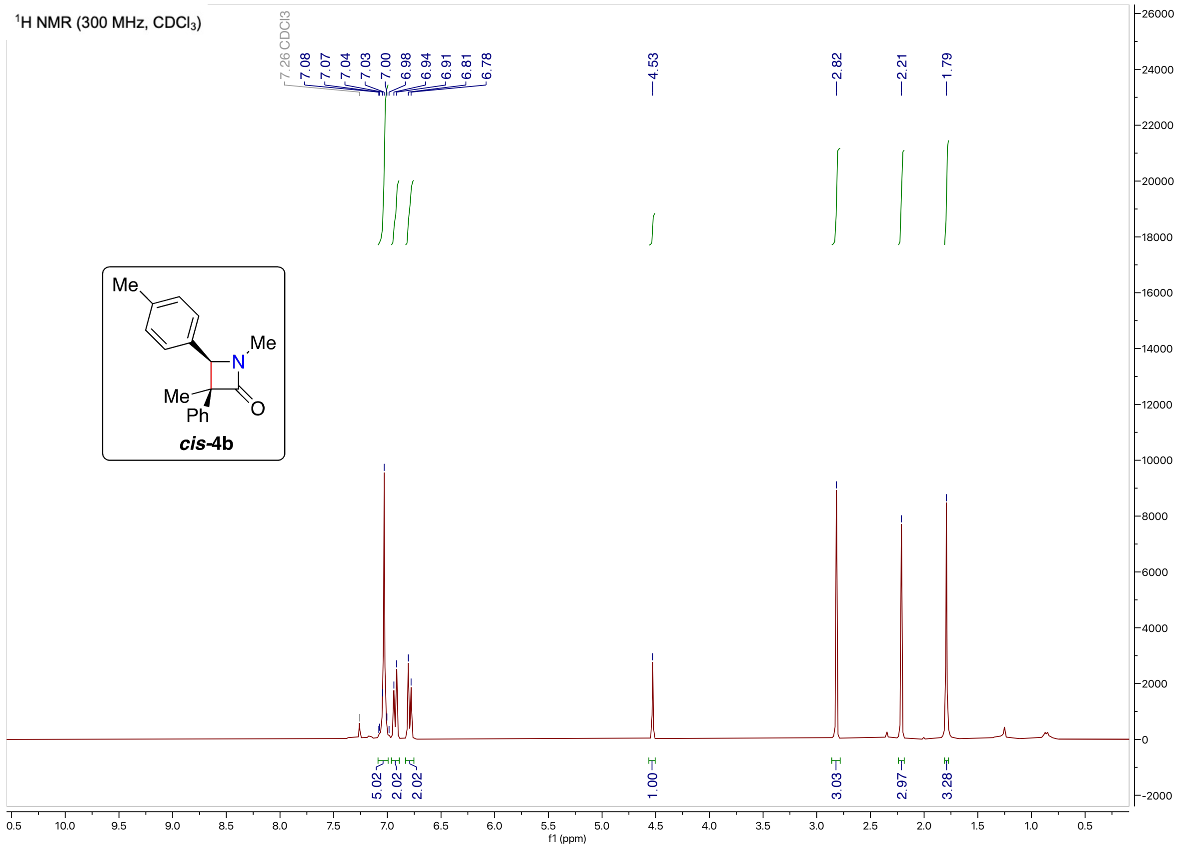

^{1}H NMR (300 MHz, CDCl$_3$)
7.26 CDCl3
7.08
7.07
7.04
7.03
7.00
6.98
6.94
6.91
6.81
6.78
4.53
2.82
2.21
1.79
Me
Me
N
Me
Ph
O
cis-4b
5.02
2.02
2.02
1.00
3.03
2.97
3.28
f1 (ppm)

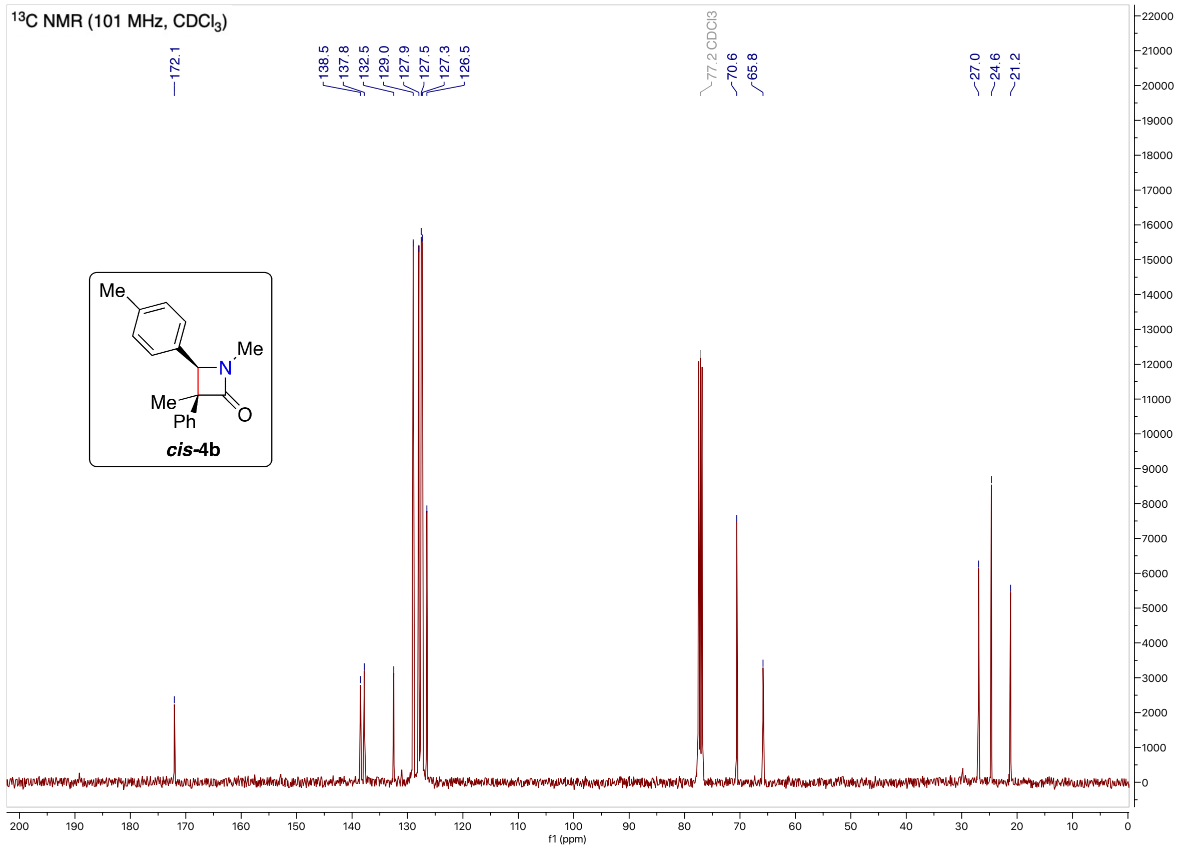

^{13}C NMR (101 MHz, CDCl$_3$)
172.1
138.5
137.8
132.5
129.0
127.9
127.5
127.3
126.5
77.2 CDCl3
70.6
65.8
27.0
24.6
21.2
Me
Me
N
Me
Ph
O
cis-4b
f1 (ppm)

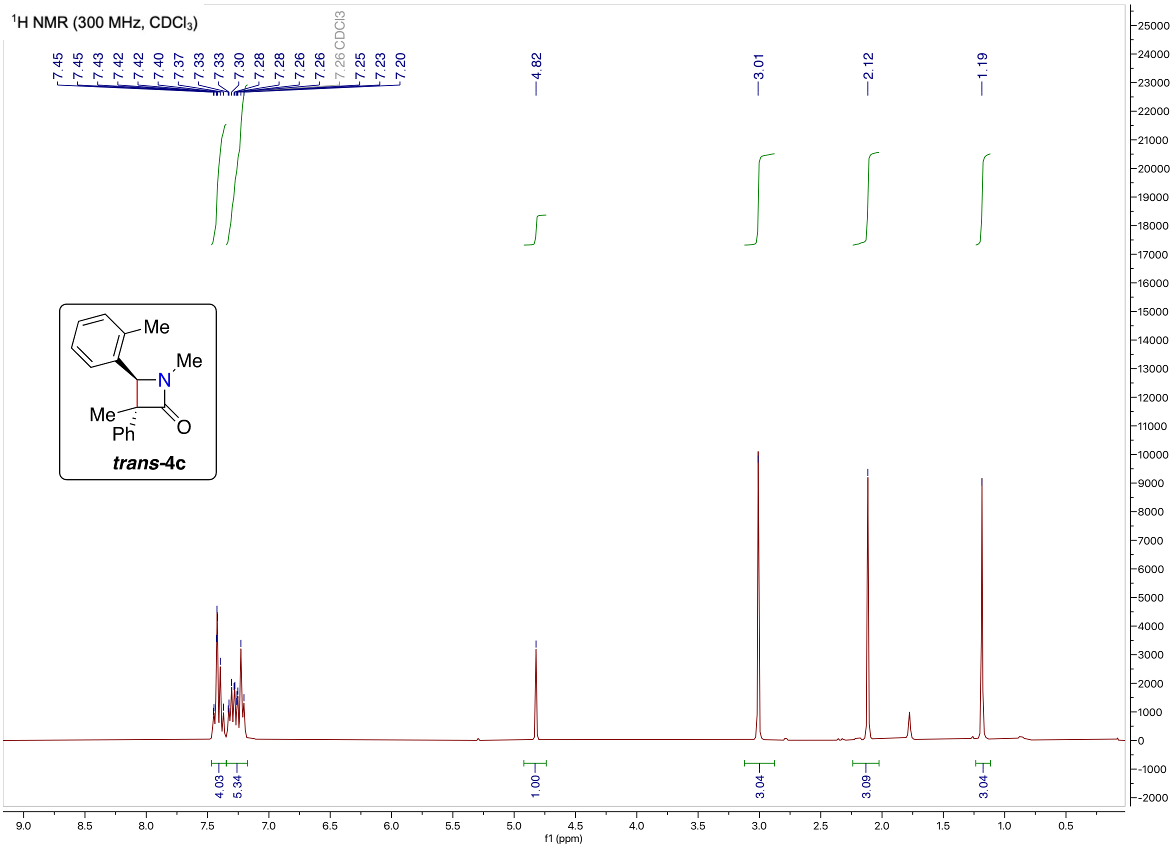

1H NMR (300 MHz, CDCl3)
7.45 7.45 7.43 7.42 7.42 7.40 7.37 7.33 7.33 7.30 7.28 7.28 7.26 7.26 7.26 CDCl3 7.25 7.23 7.20
4.82
3.01
2.12
1.19
4.03 5.34 1.00 3.04 3.09 3.04
Me
N
Me
Me
Ph
O
trans-4c
f1 (ppm)

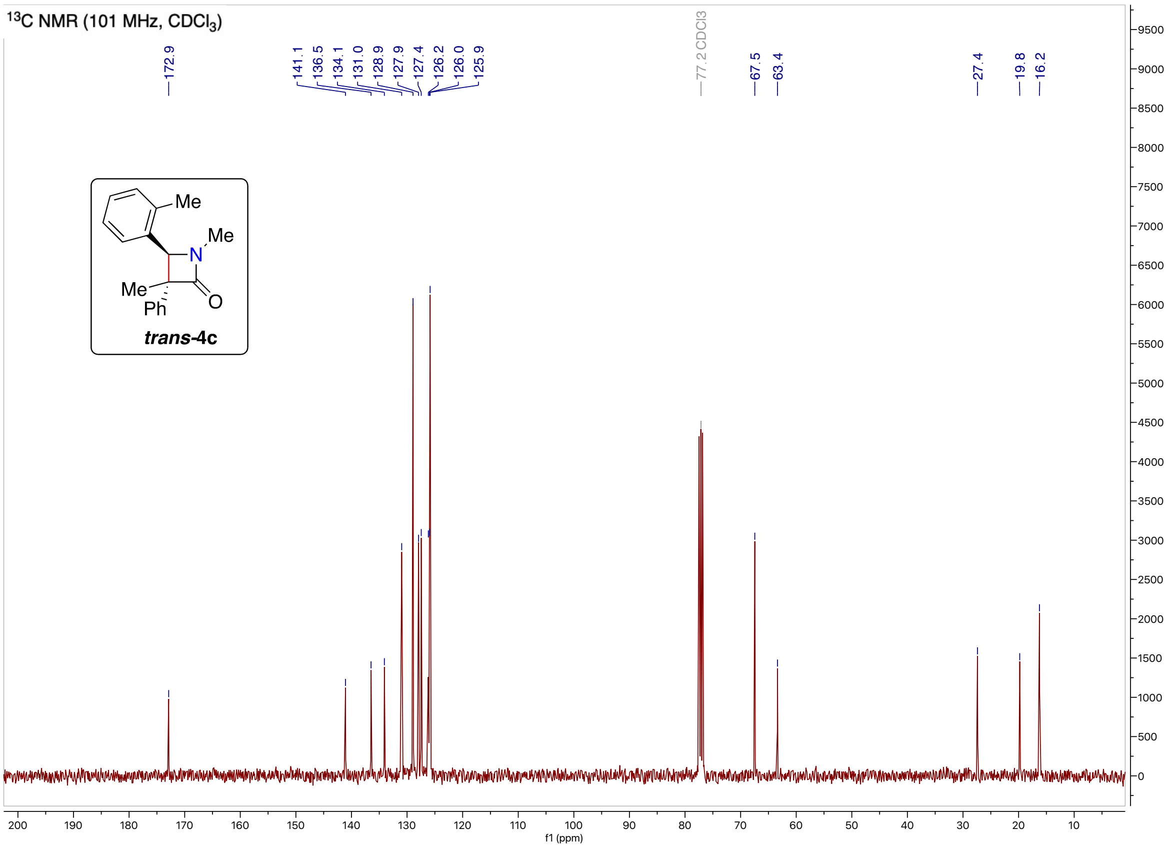

13C NMR (101 MHz, CDCl3)
172.9
141.1 136.5 134.1 131.0 128.9 127.9 127.4 126.2 126.0 125.9
77.2 CDCl3
67.5
63.4
27.4
19.8
16.2
Me
N
Me
Me
Ph
O
trans-4c
f1 (ppm)

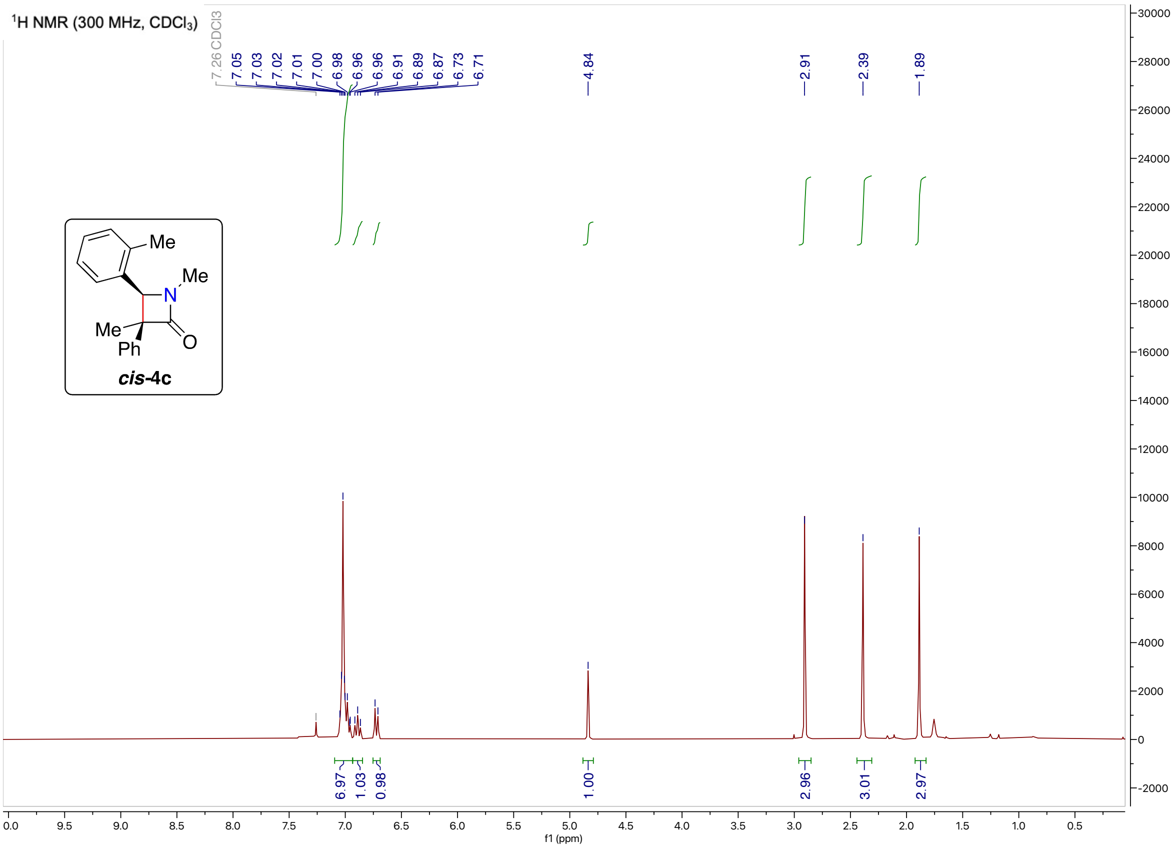

1H NMR (300 MHz, CDCl3)
7.26 CDCl3
7.05
7.03
7.02
7.01
7.00
6.98
6.96
6.96
6.91
6.89
6.87
6.73
6.71
4.84
2.91
2.39
1.89
Me
N
Me
Me
Ph
O
cis-4c
6.97
1.03
0.98
1.00
2.96
3.01
2.97
f1 (ppm)

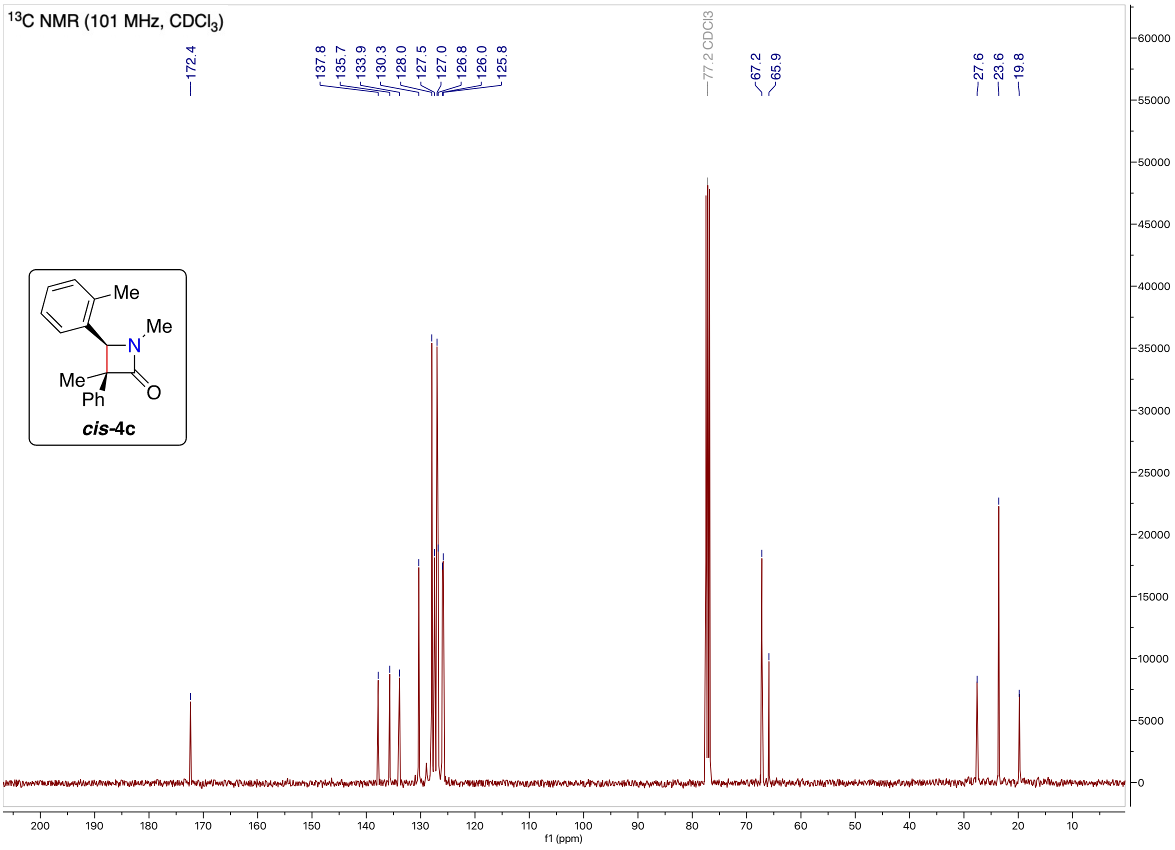

13C NMR (101 MHz, CDCl3)
172.4
137.8
135.7
133.9
130.3
128.0
127.5
127.0
126.8
126.0
125.8
77.2 CDCl3
67.2
65.9
27.6
23.6
19.8
Me
N
Me
Me
Ph
O
cis-4c
f1 (ppm)

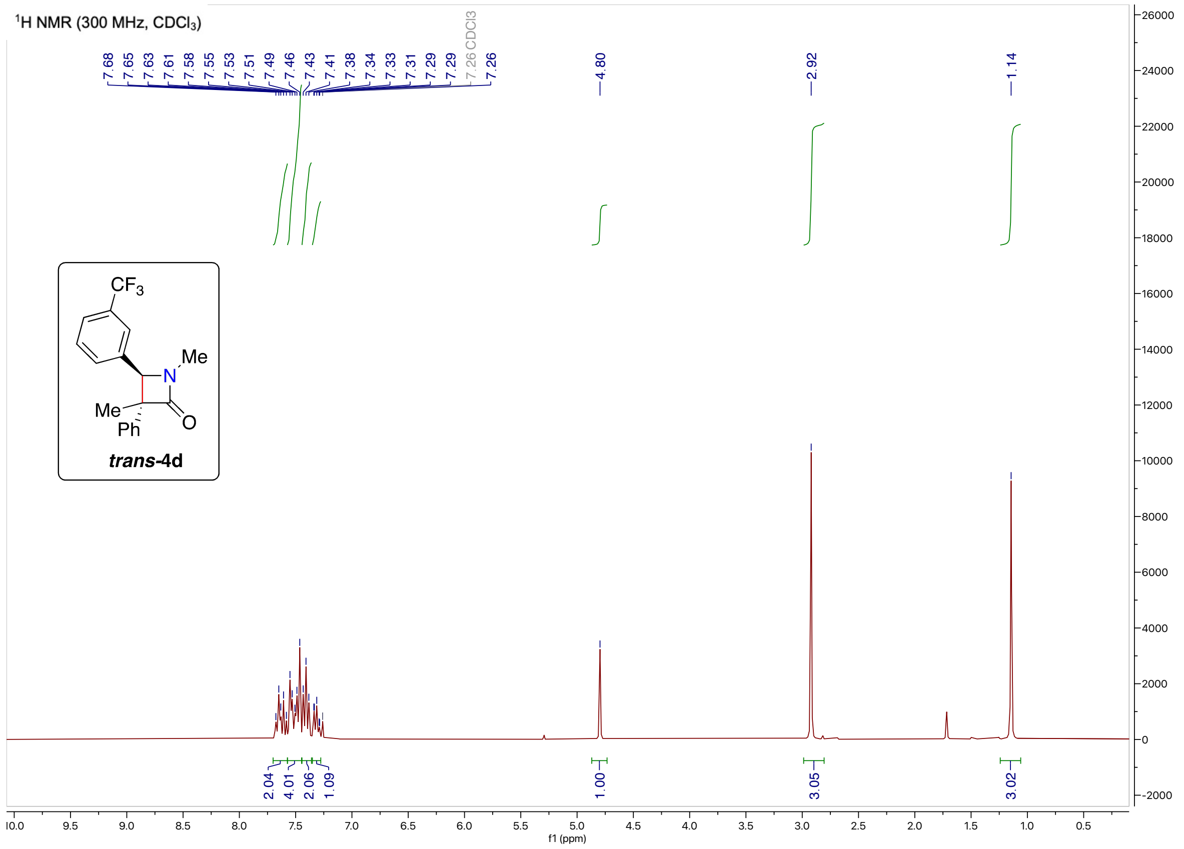

1H NMR (300 MHz, CDCl3)
7.68 7.65 7.63 7.61 7.58 7.55 7.53 7.51 7.49 7.46 7.43 7.41 7.38 7.34 7.33 7.31 7.29 7.29 7.26 CDCl3 7.26
4.80
2.92
1.14
2.04 4.01 2.06 1.09
1.00
3.05
3.02
CF3
Me
N
Me
Ph
O
trans-4d
f1 (ppm)

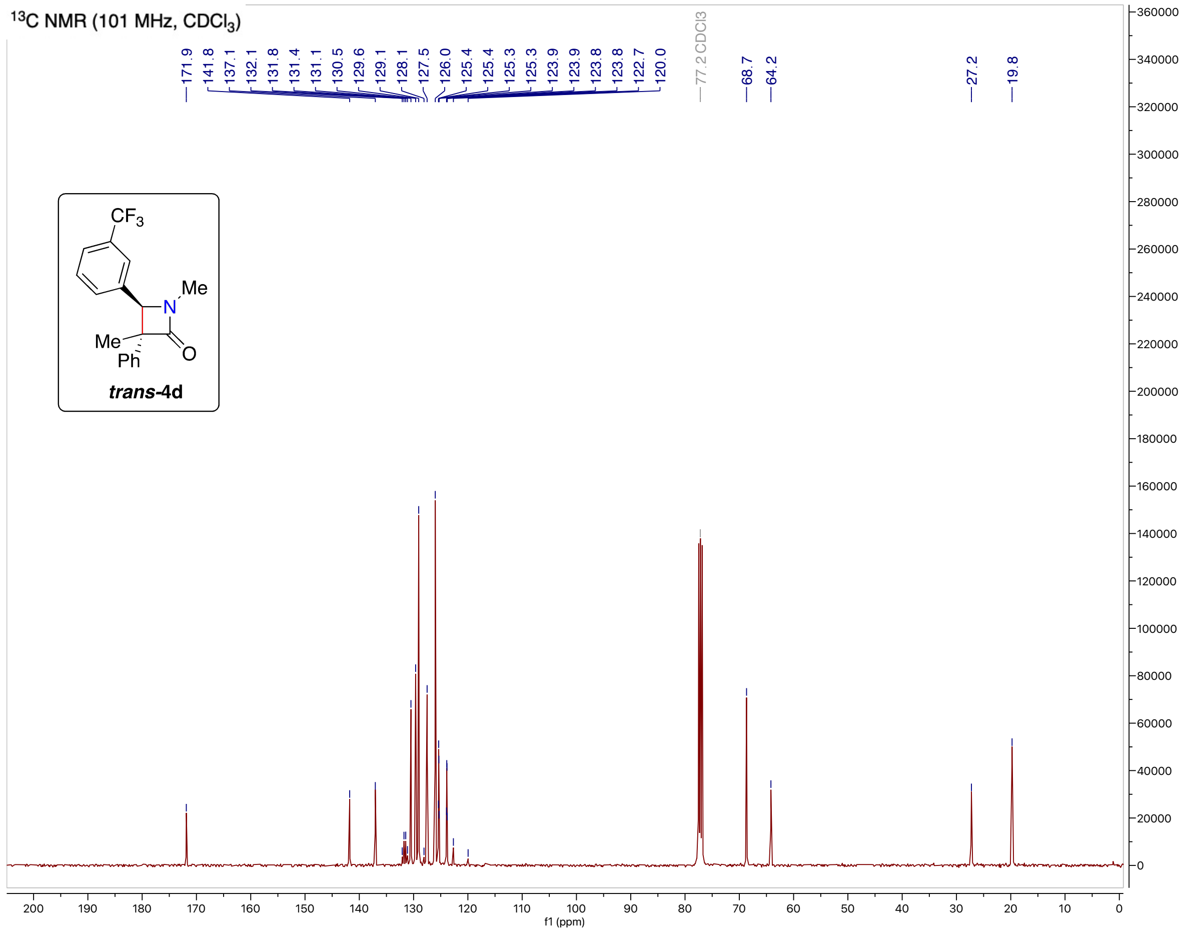

13C NMR (101 MHz, CDCl3)
171.9
141.8 137.1 132.1 131.8 131.4 131.1 130.5 129.6 129.1 128.1 127.5 126.0 125.4 125.3 125.3 123.9 123.8 123.8 122.7 120.0
77.2 CDCl3
68.7
64.2
27.2
19.8
CF3
Me
N
Me
Ph
O
trans-4d
f1 (ppm)

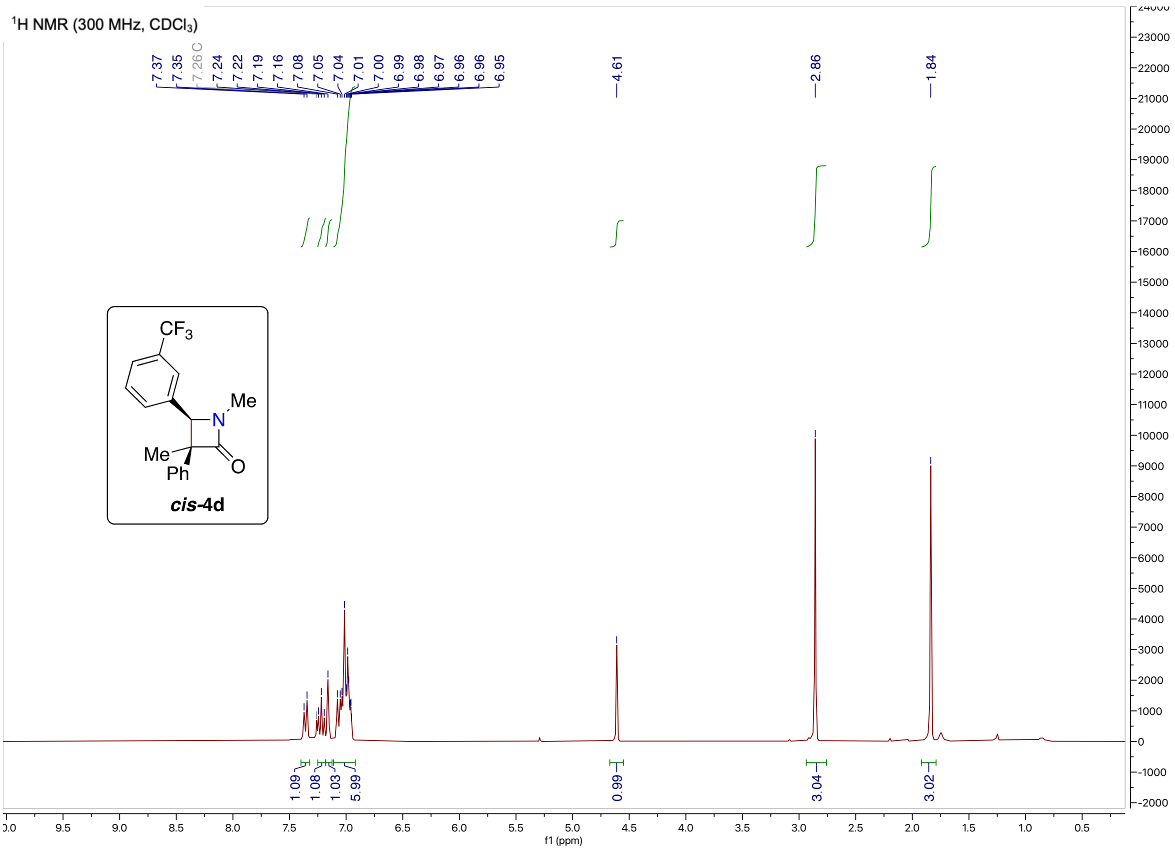

1H NMR (300 MHz, CDCl3)
7.37
7.35
7.26 C
7.24
7.22
7.19
7.16
7.08
7.05
7.04
7.01
7.00
6.99
6.98
6.97
6.96
6.96
6.95
4.61
2.86
1.84
1.09
1.08
1.03
5.99
0.99
3.04
3.02
CF3
Me
N
Me
Ph
O
cis-4d
f1 (ppm)

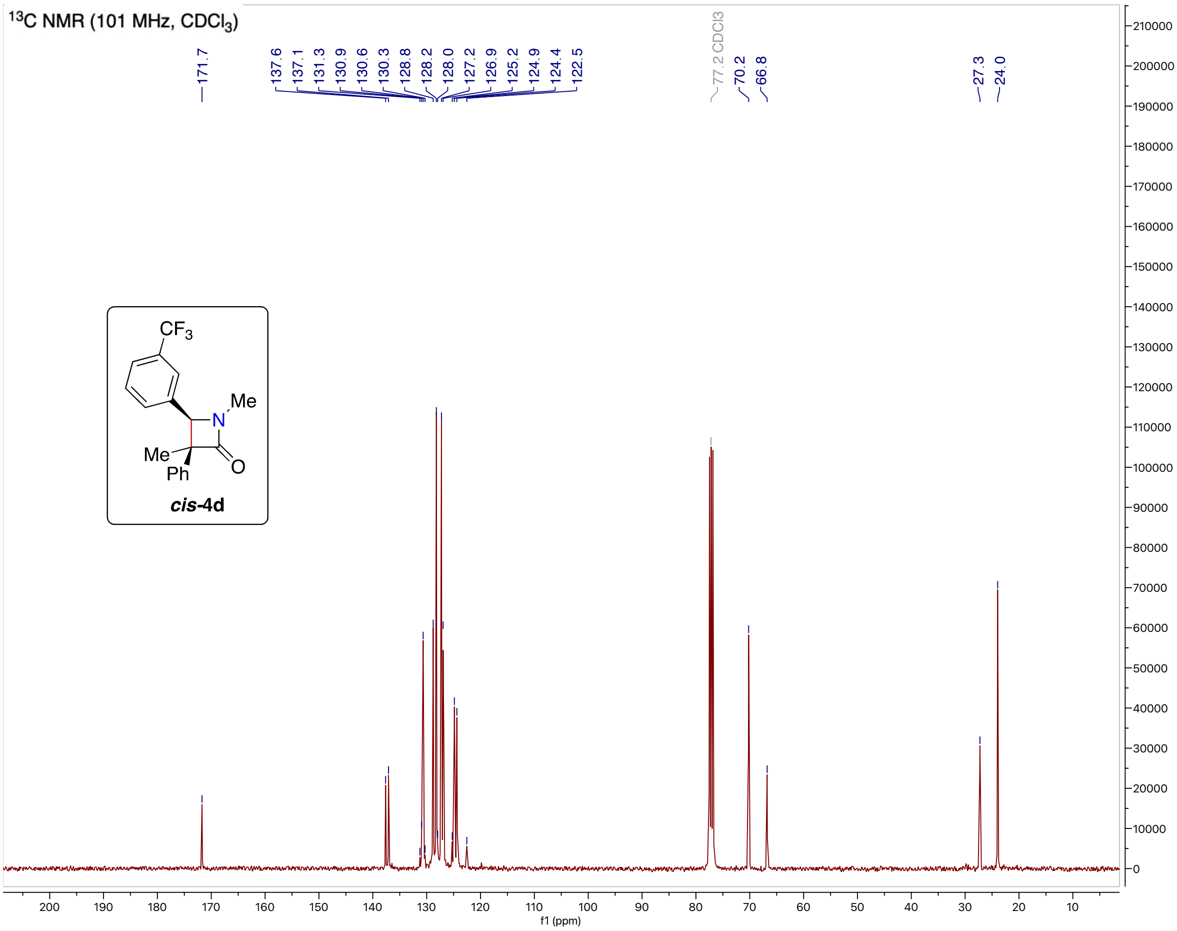

13C NMR (101 MHz, CDCl3)
171.7
137.6
137.1
131.3
130.9
130.6
130.3
128.8
128.2
128.0
127.2
126.9
125.2
124.9
124.4
122.5
77.2 CDCl3
70.2
66.8
27.3
24.0
CF3
Me
N
Me
Ph
O
cis-4d
f1 (ppm)

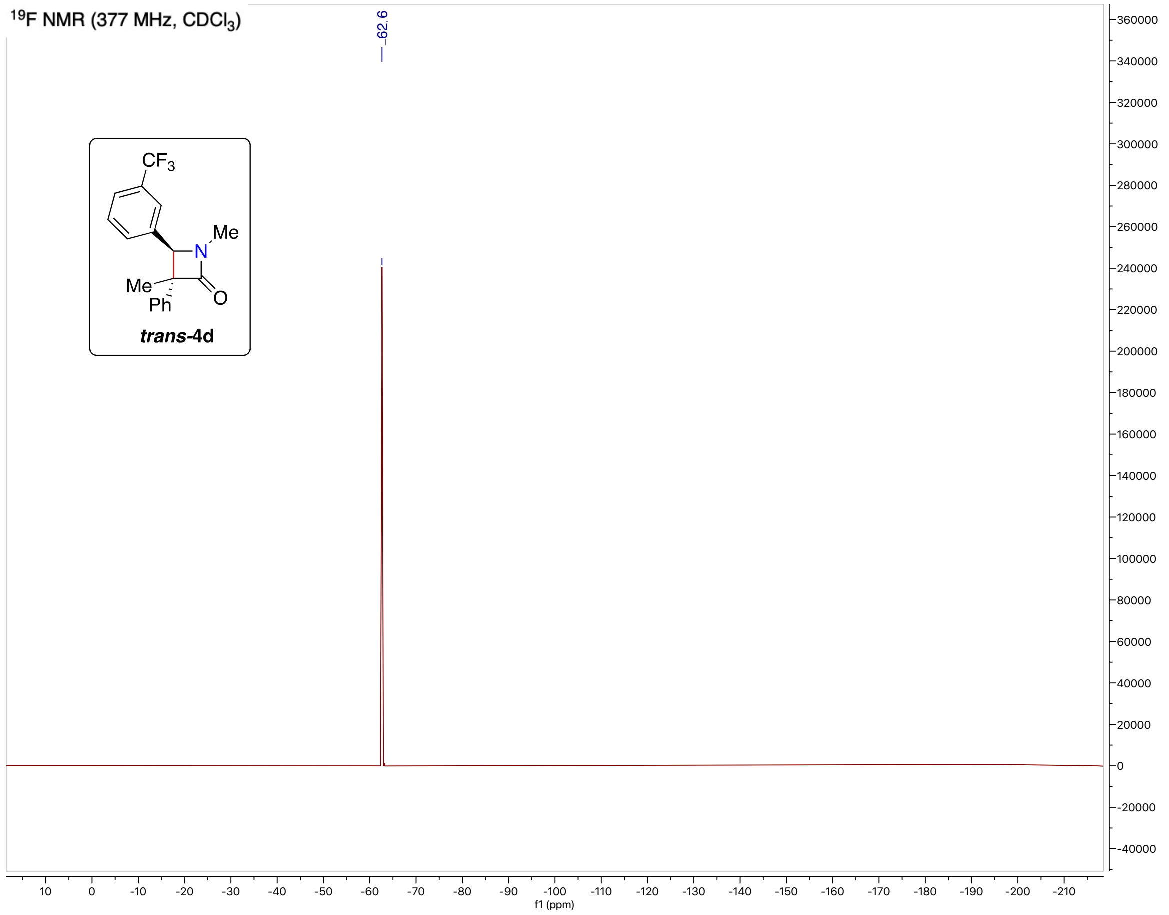

19F NMR (377 MHz, CDCl3)
-62.6
CF3
Me
N
Me
Ph
O
trans-4d
f1 (ppm)

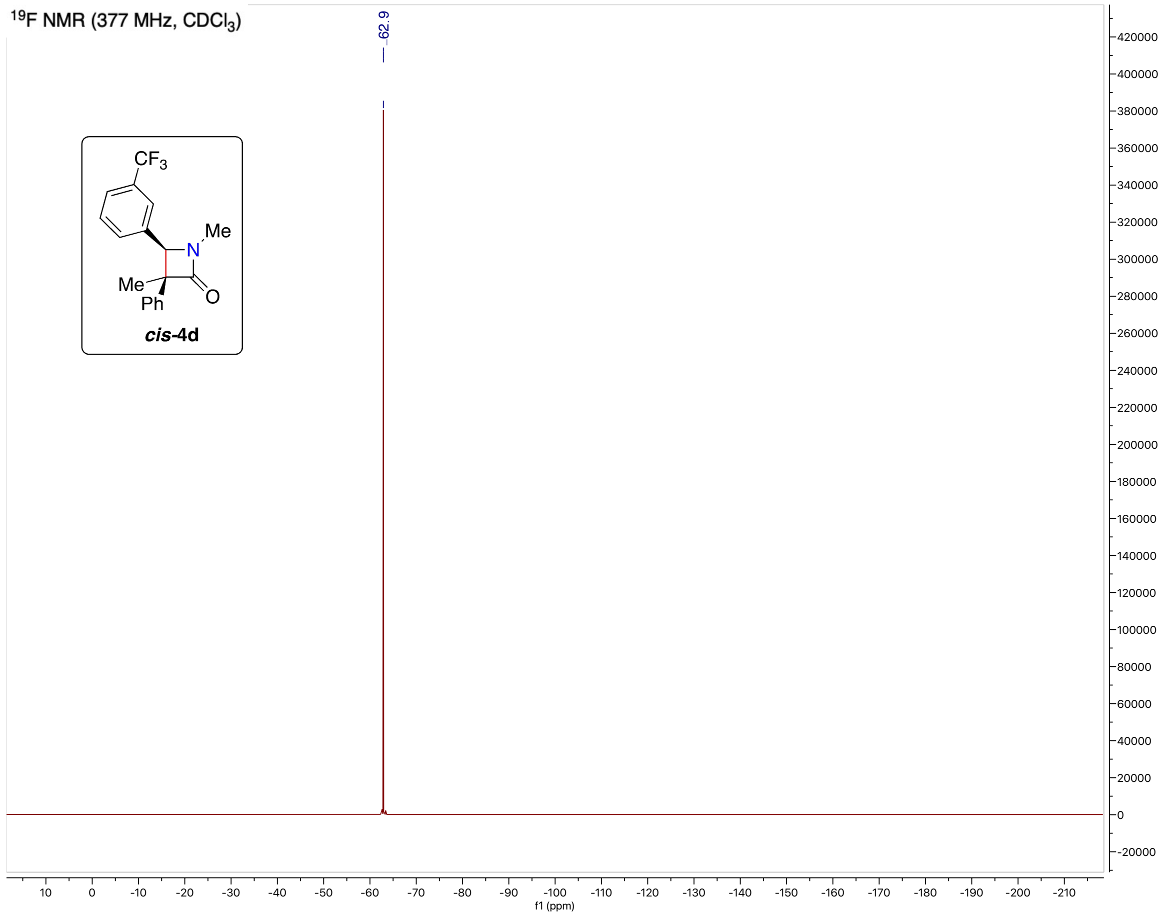

19F NMR (377 MHz, CDCl3)
-62.9
CF3
Me
N
Me
Ph
O
cis-4d
f1 (ppm)

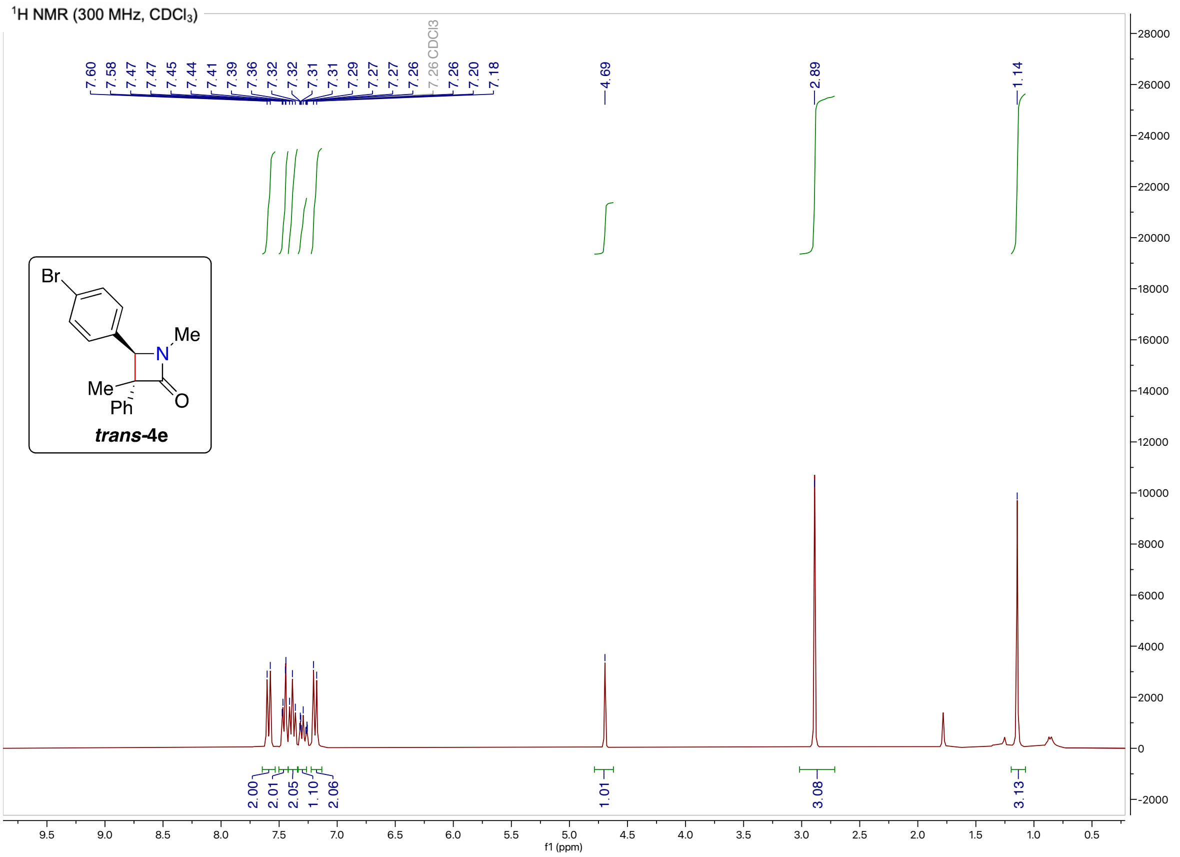

^{1}H NMR (300 MHz, CDCl$_3$)
7.60 7.58 7.47 7.47 7.45 7.44 7.41 7.39 7.36 7.32 7.32 7.31 7.31 7.29 7.27 7.27 7.26 7.26 CDCl3 7.26 7.20 7.18
4.69
2.89
1.14
2.00 2.01 2.05 1.10 2.06
1.01
3.08
3.13
trans-4e
f1 (ppm)

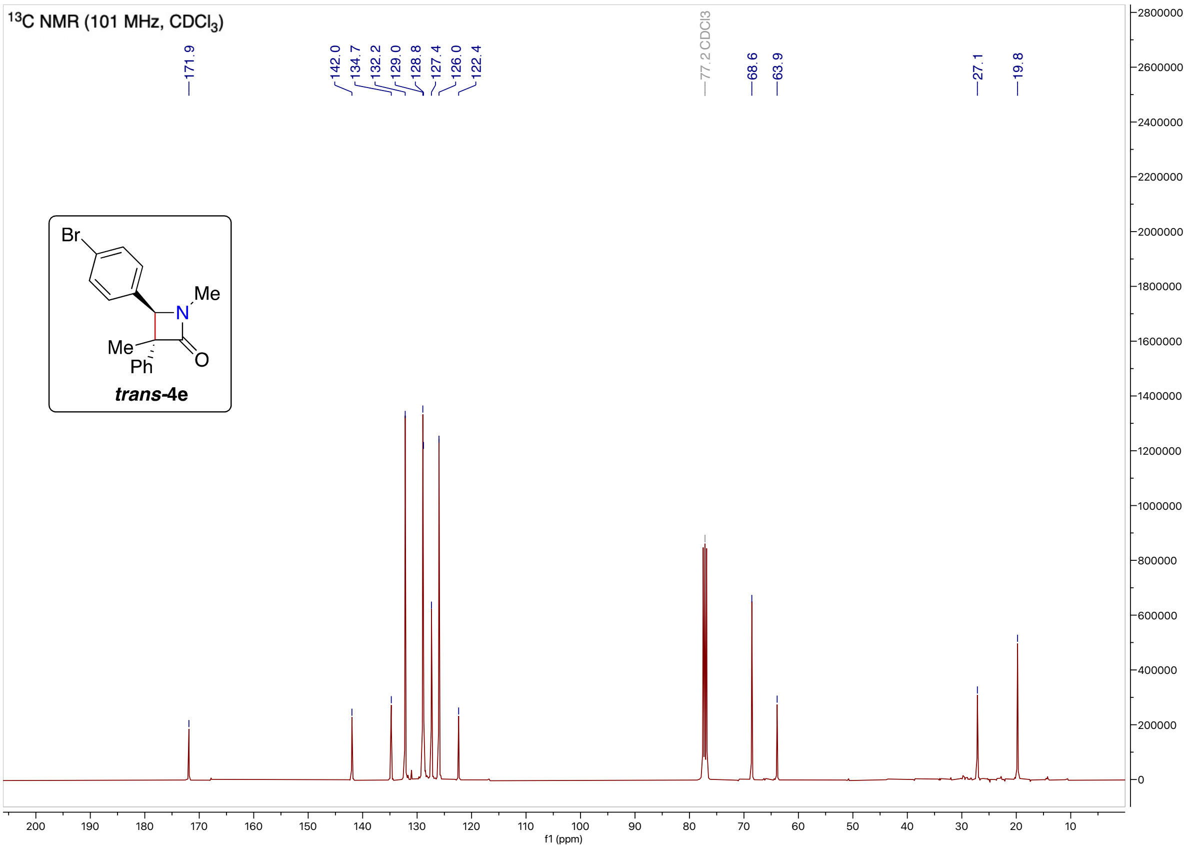

^{13}C NMR (101 MHz, CDCl$_3$)
171.9
142.0 134.7 132.2 129.0 128.8 127.4 126.0 122.4
77.2 CDCl3
68.6
63.9
27.1
19.8
trans-4e
f1 (ppm)

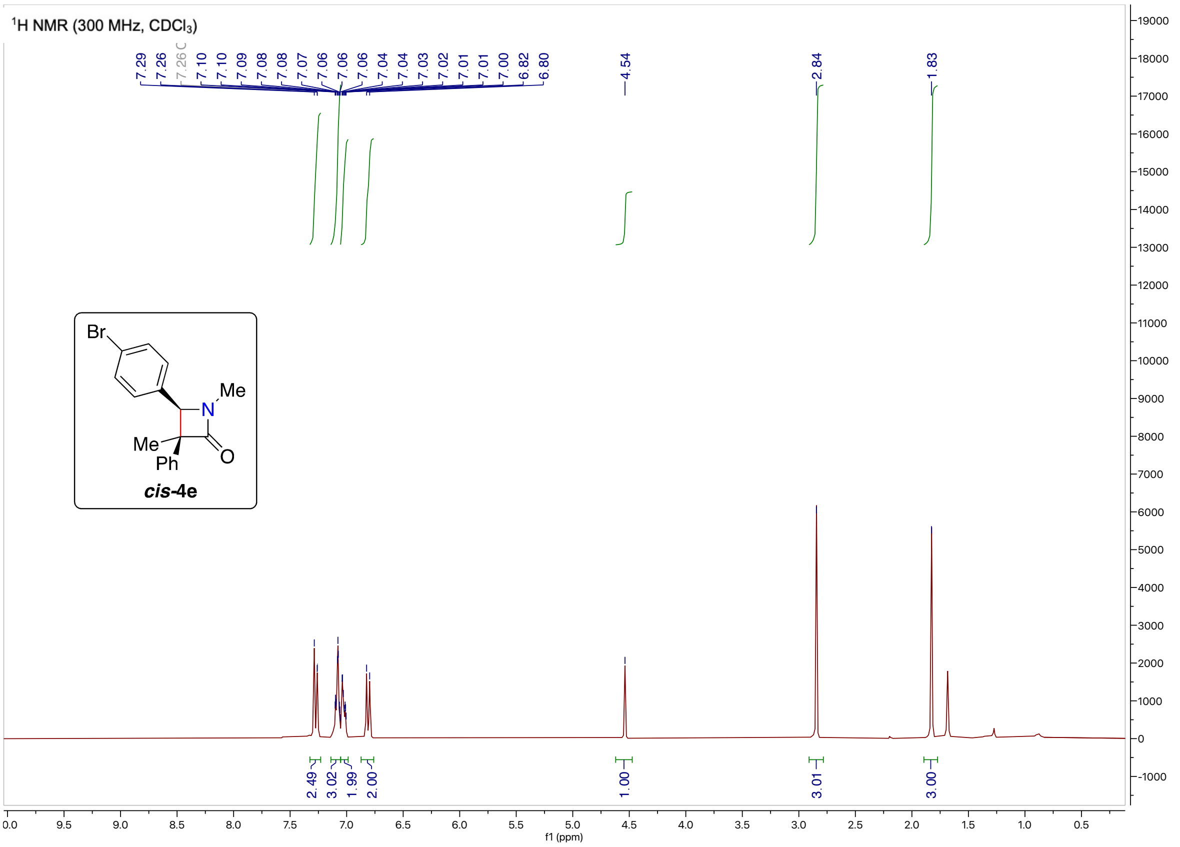

1H NMR (300 MHz, CDCl3)
7.29
7.26
7.26 C
7.10
7.10
7.09
7.08
7.08
7.07
7.06
7.06
7.06
7.04
7.04
7.03
7.02
7.01
7.01
7.00
6.82
6.80
4.54
2.84
1.83
2.49
3.02
1.99
2.00
1.00
3.01
3.00
Br
N
Me
Me
Ph
O
cis-4e
f1 (ppm)

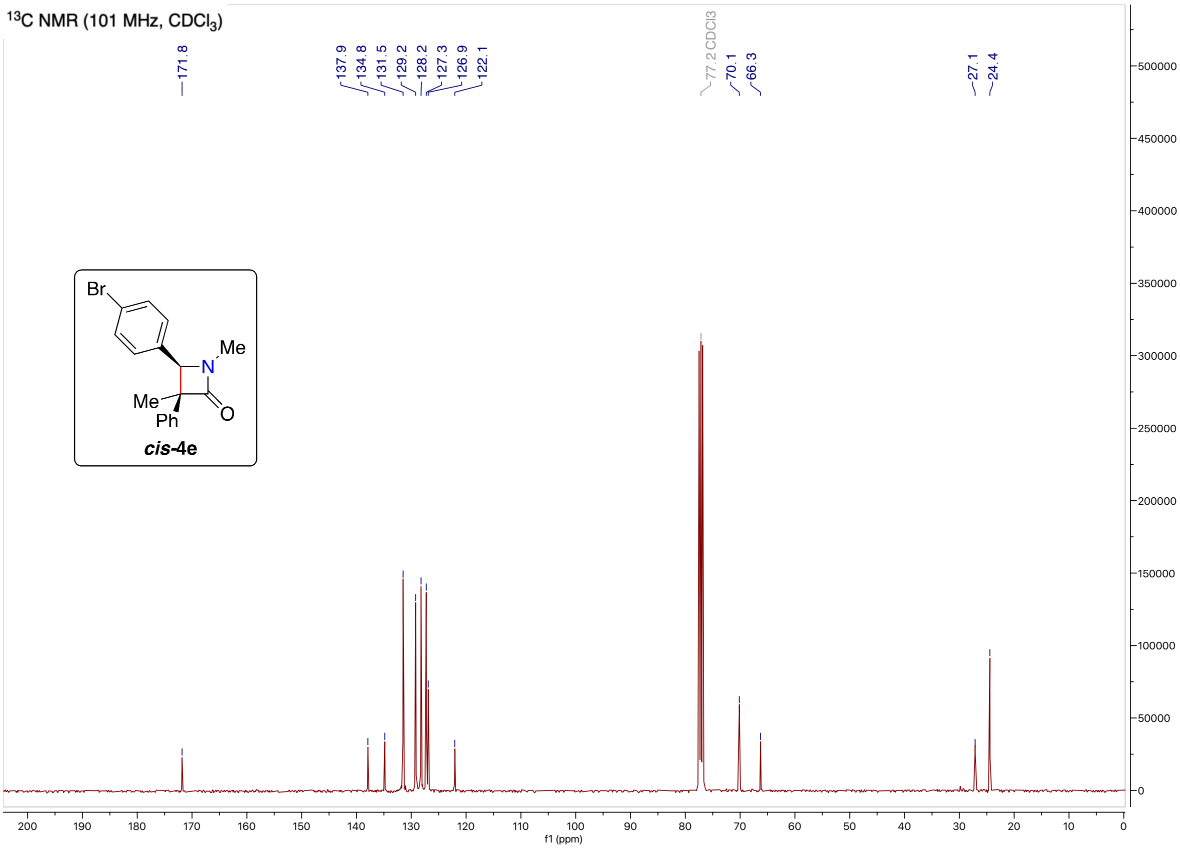

13C NMR (101 MHz, CDCl3)
171.8
137.9
134.8
131.5
129.2
128.2
127.3
126.9
122.1
77.2 CDCl3
70.1
66.3
27.1
24.4
Br
N
Me
Me
Ph
O
cis-4e
f1 (ppm)

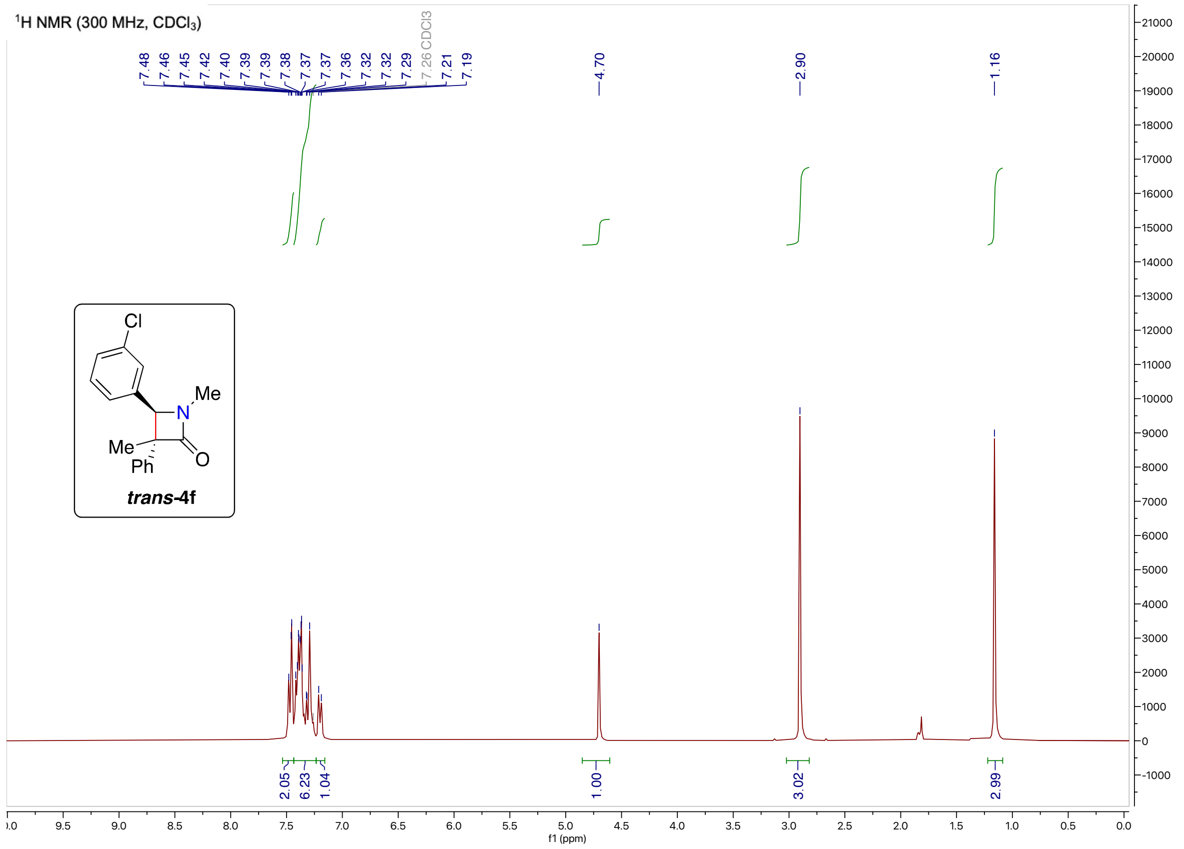

1H NMR (300 MHz, CDCl3)
7.48
7.46
7.45
7.42
7.40
7.39
7.38
7.37
7.37
7.36
7.32
7.32
7.29
7.26 CDCl3
7.21
7.19
4.70
2.90
1.16
Cl
N
Me
Me
Ph
O
trans-4f
2.05
6.23
1.04
1.00
3.02
2.99
f1 (ppm)

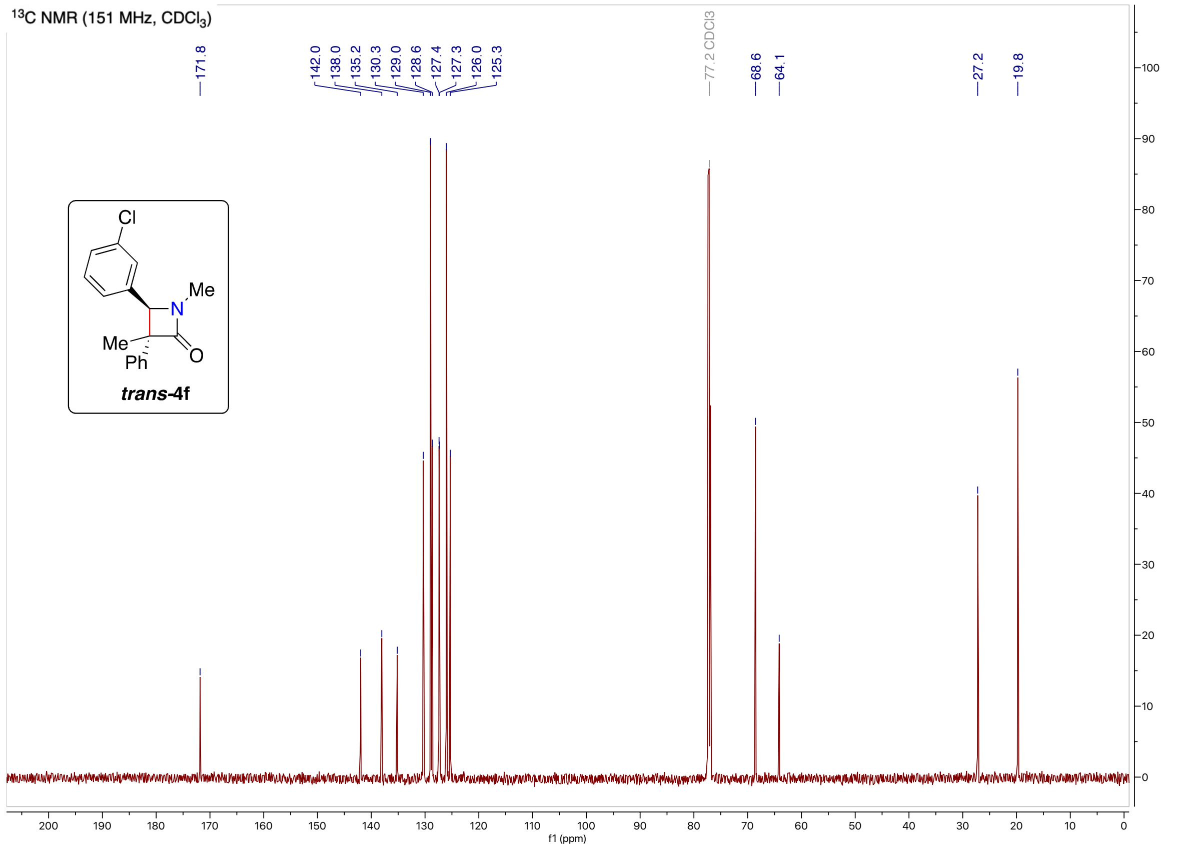

13C NMR (151 MHz, CDCl3)
171.8
142.0
138.0
135.2
130.3
129.0
128.6
127.4
127.3
126.0
125.3
77.2 CDCl3
68.6
64.1
27.2
19.8
Cl
N
Me
Me
Ph
O
trans-4f
f1 (ppm)

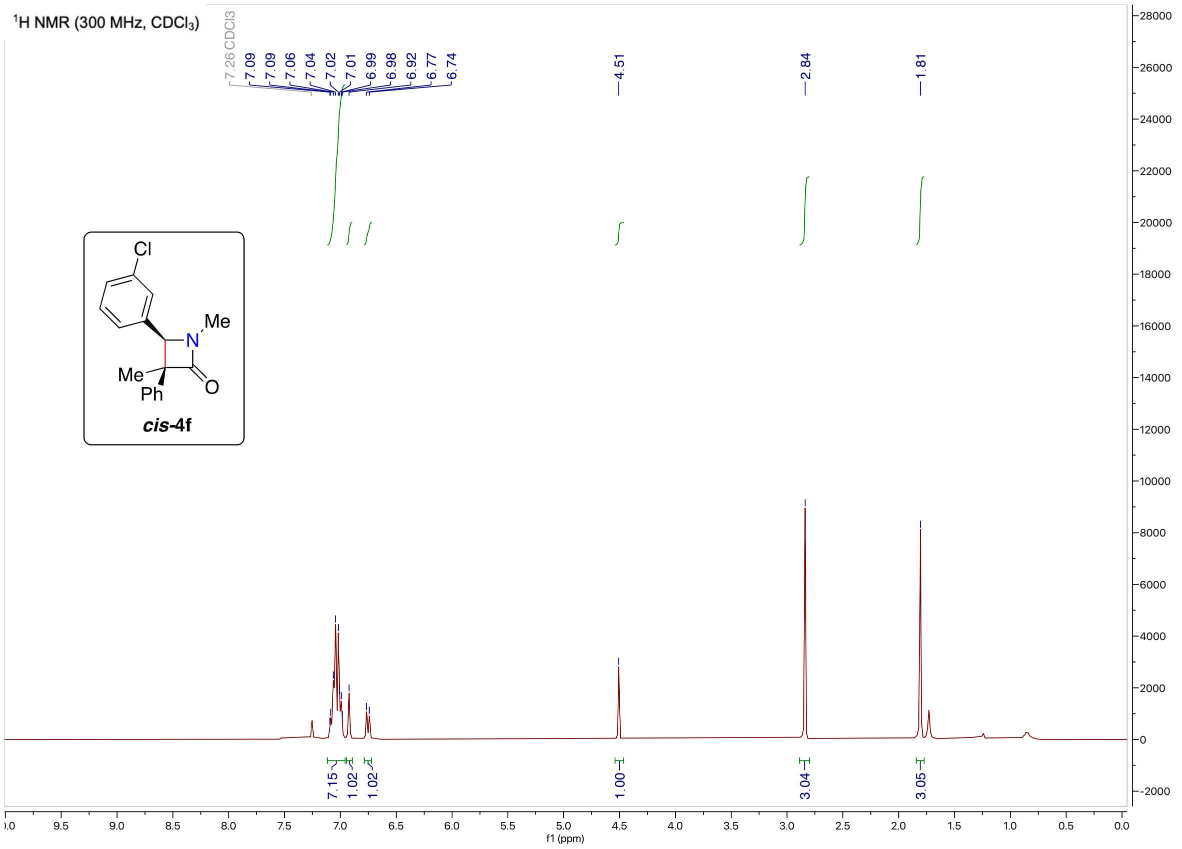

1H NMR (300 MHz, CDCl3)
7.26 CDCl3
7.09
7.09
7.06
7.04
7.02
7.01
6.99
6.98
6.92
6.77
6.74
4.51
2.84
1.81
7.15
1.02
1.02
1.00
3.04
3.05
cis-4f
f1 (ppm)

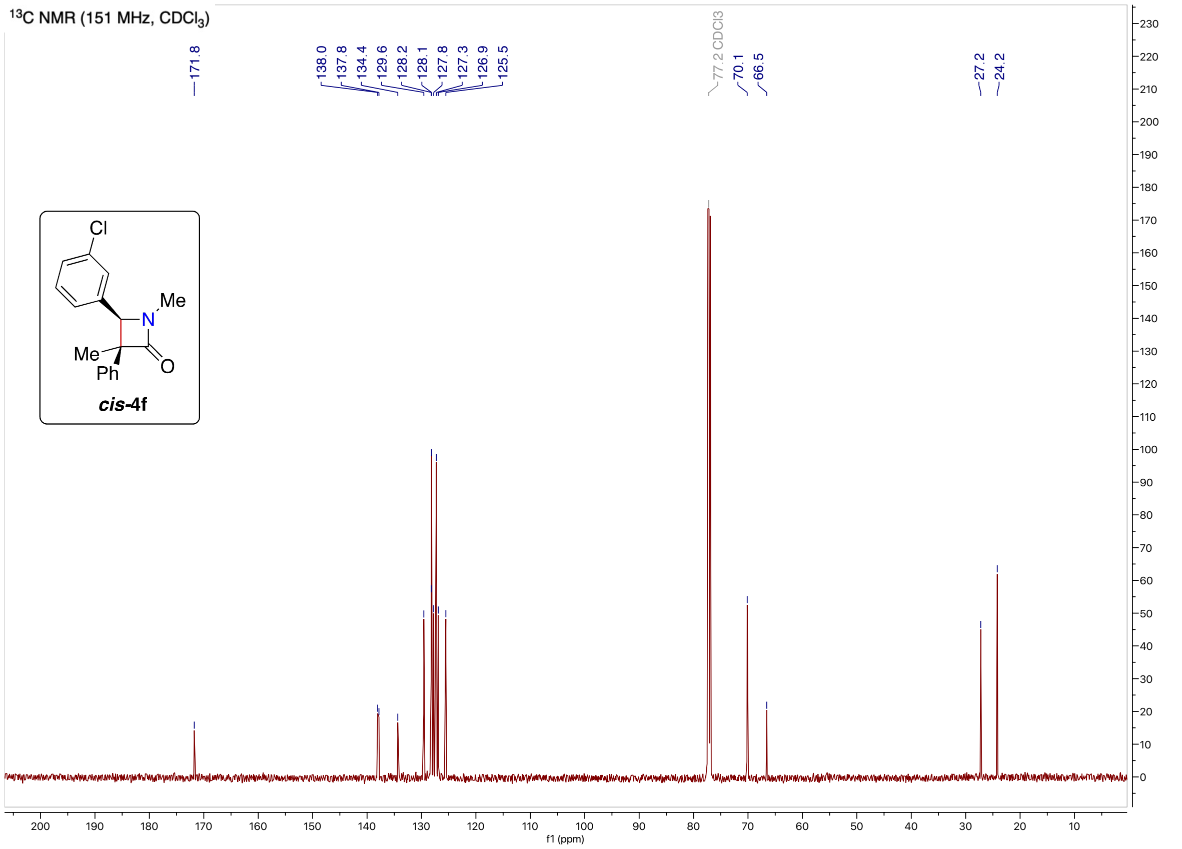

13C NMR (151 MHz, CDCl3)
171.8
138.0
137.8
134.4
129.6
128.2
128.1
127.8
127.3
126.9
125.5
77.2 CDCl3
70.1
66.5
27.2
24.2
cis-4f
f1 (ppm)

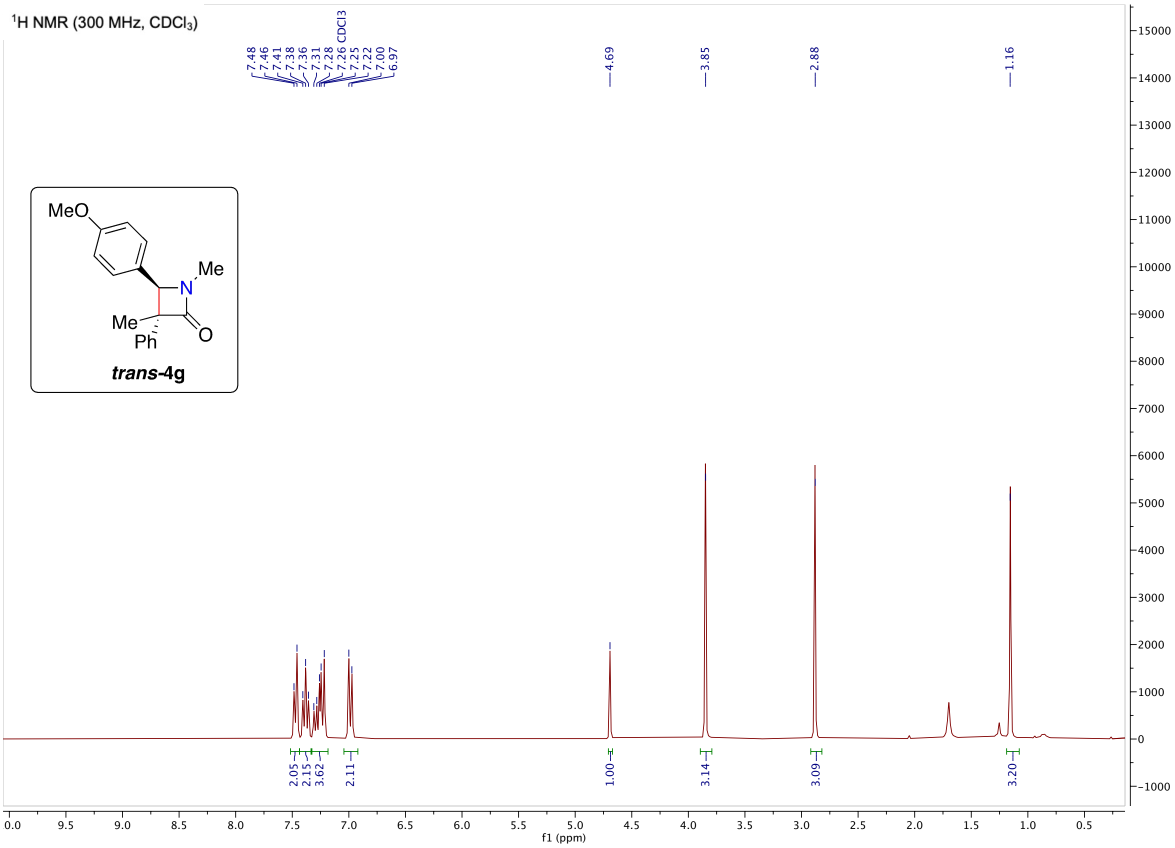

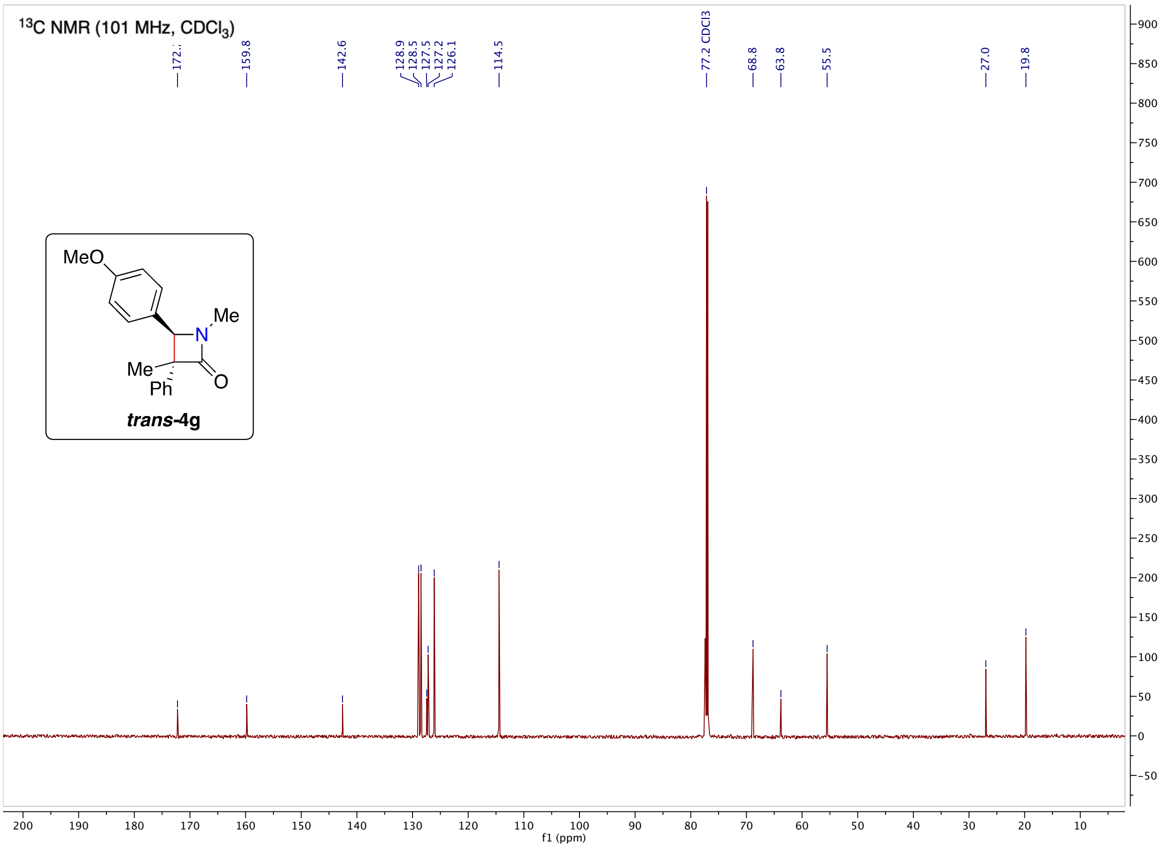

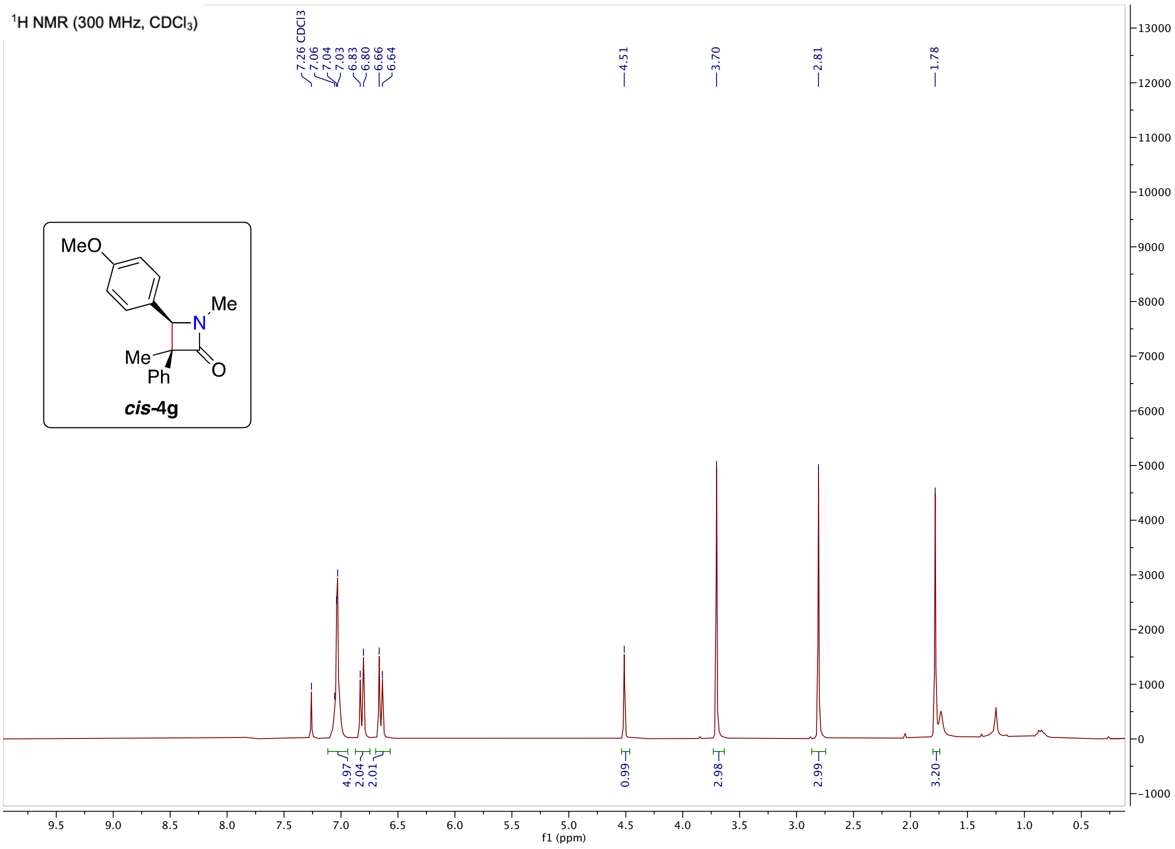

^{1}H NMR (300 MHz, CDCl$_3$)
7.26 CDCl3
7.06
7.04
7.03
6.83
6.80
6.66
6.64
4.51
3.70
2.81
1.78
MeO
Me
N
Me
Ph
O
cis-4g
4.97
2.04
2.01
0.99
2.98
2.99
3.20
f1 (ppm)

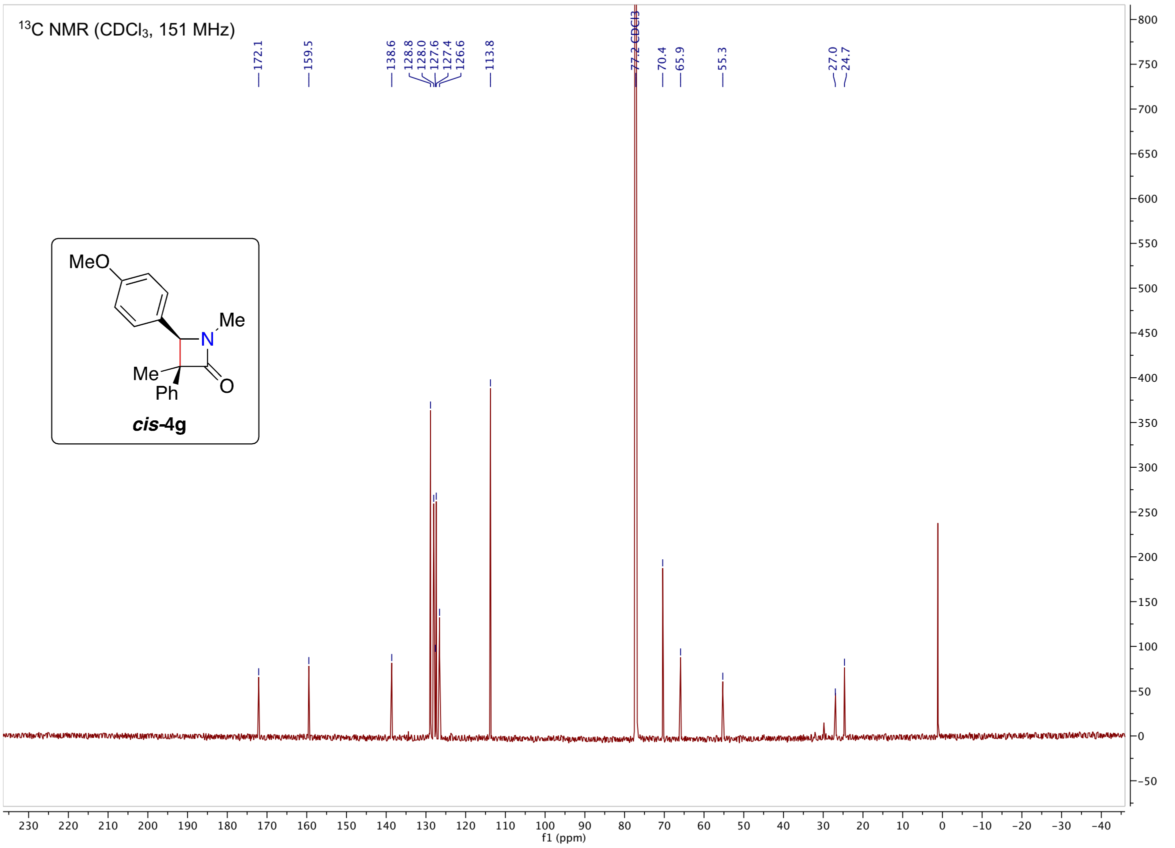

^{13}C NMR (CDCl$_3$, 151 MHz)
172.1
159.5
138.6
128.8
128.0
127.6
127.4
126.6
113.8
77.2 CDCl3
70.4
65.9
55.3
27.0
24.7
MeO
Me
N
Me
Ph
O
cis-4g
f1 (ppm)

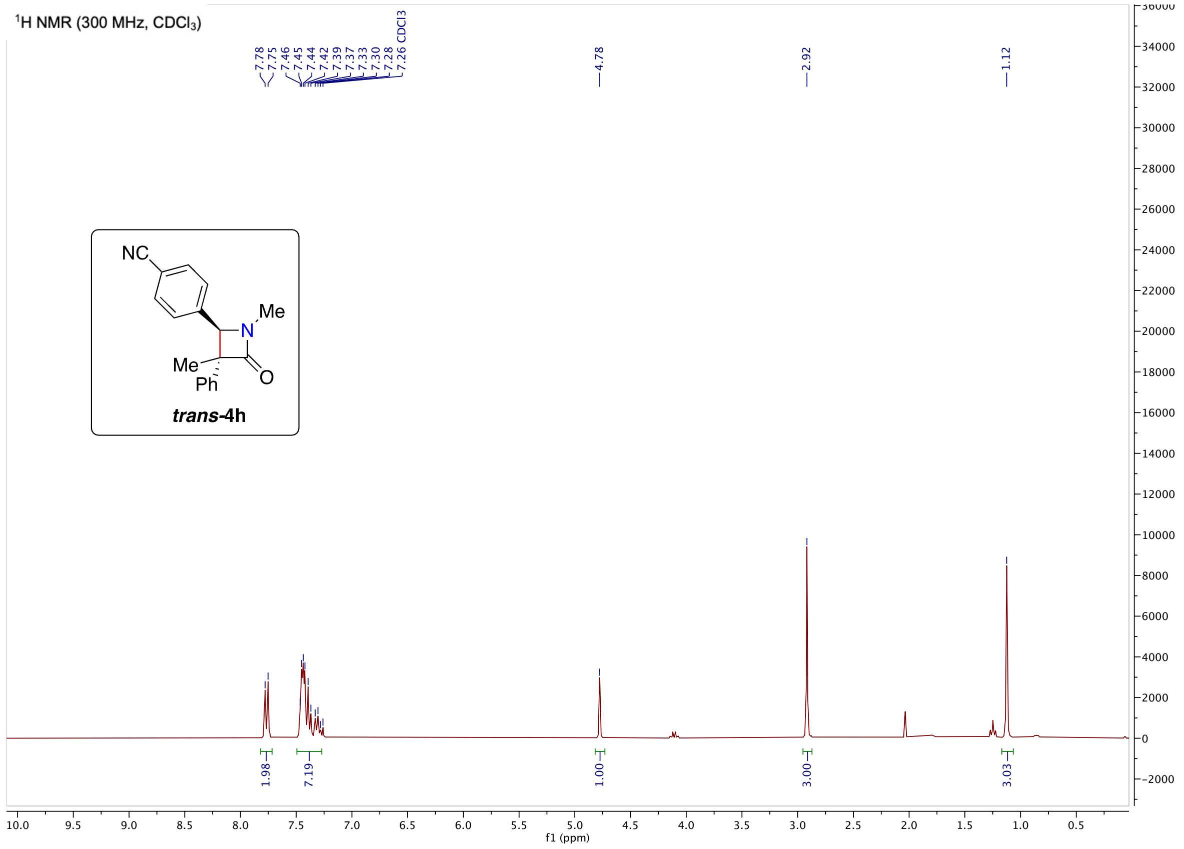

^{1}H NMR (300 MHz, CDCl$_3$)
7.78
7.75
7.46
7.45
7.44
7.42
7.39
7.37
7.33
7.30
7.28
7.26 CDCl3
4.78
2.92
1.12
NC
Me
N
Me
Ph
O
trans-4h
1.98
7.19
1.00
3.00
3.03
f1 (ppm)

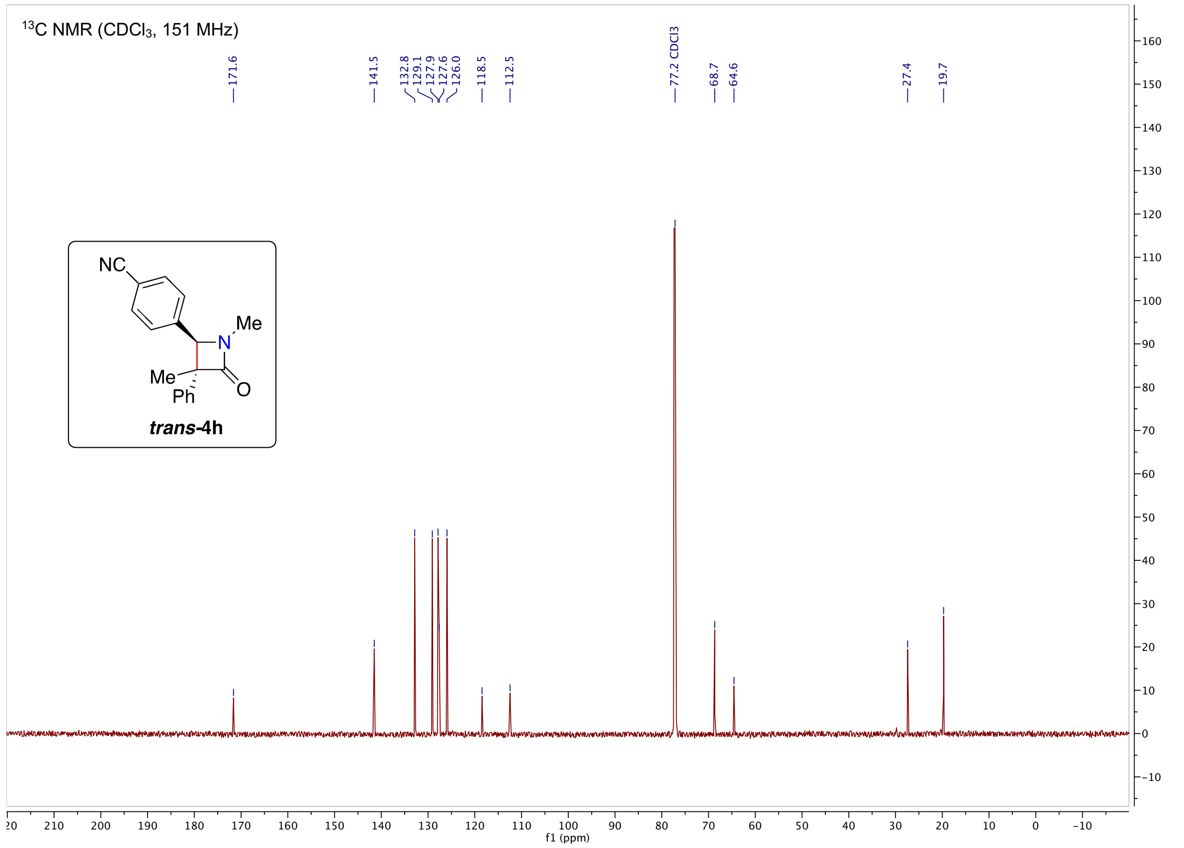

^{13}C NMR (CDCl$_3$, 151 MHz)
171.6
141.5
132.8
129.1
127.9
127.6
126.0
118.5
112.5
77.2 CDCl3
68.7
64.6
27.4
19.7
NC
Me
N
Me
Ph
O
trans-4h
f1 (ppm)

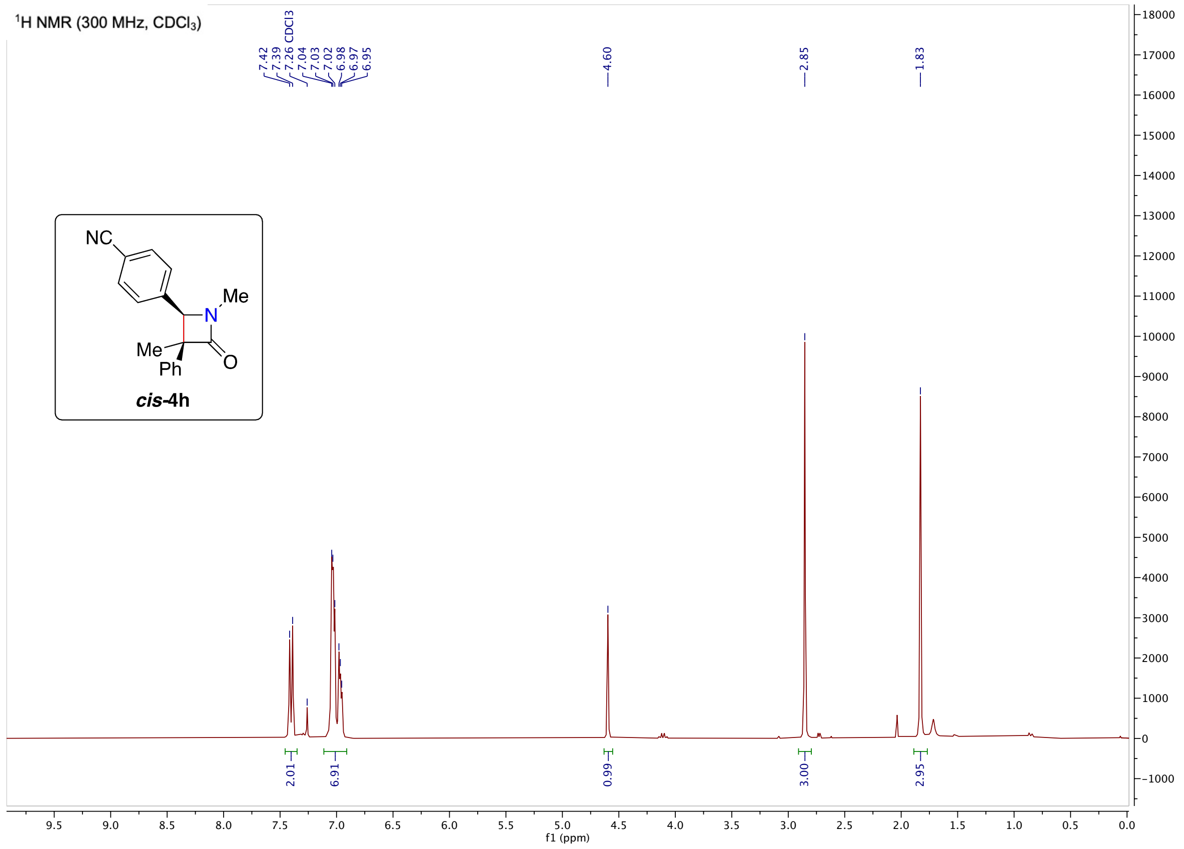

¹H NMR (300 MHz, CDCl₃)
7.42
7.39
7.26 CDCl3
7.04
7.03
7.02
6.98
6.97
6.95
4.60
2.85
1.83
NC
Me
N
Me
Ph
O
cis-4h
2.01
6.91
0.99
3.00
2.95
f1 (ppm)

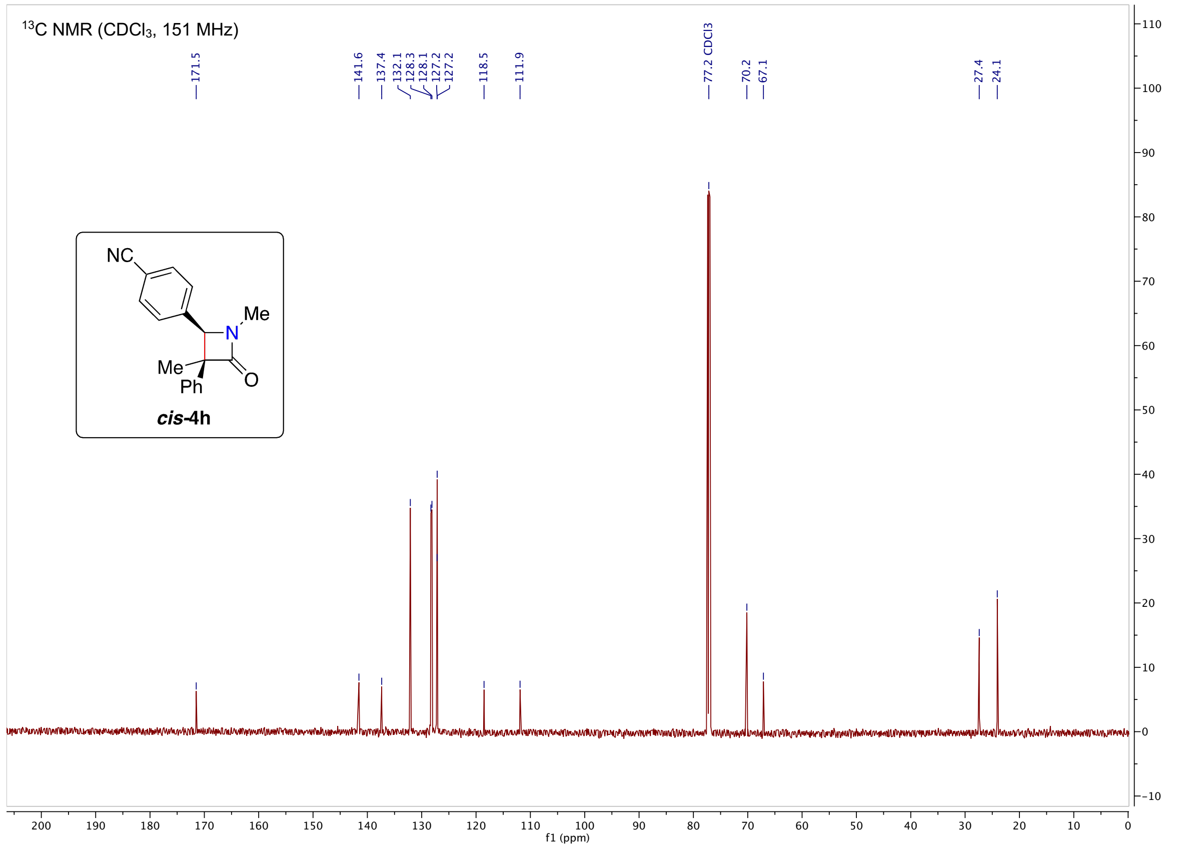

¹³C NMR (CDCl₃, 151 MHz)
171.5
141.6
137.4
132.1
128.3
128.1
127.2
127.2
118.5
111.9
77.2 CDCl3
70.2
67.1
27.4
24.1
NC
Me
N
Me
Ph
O
cis-4h
f1 (ppm)

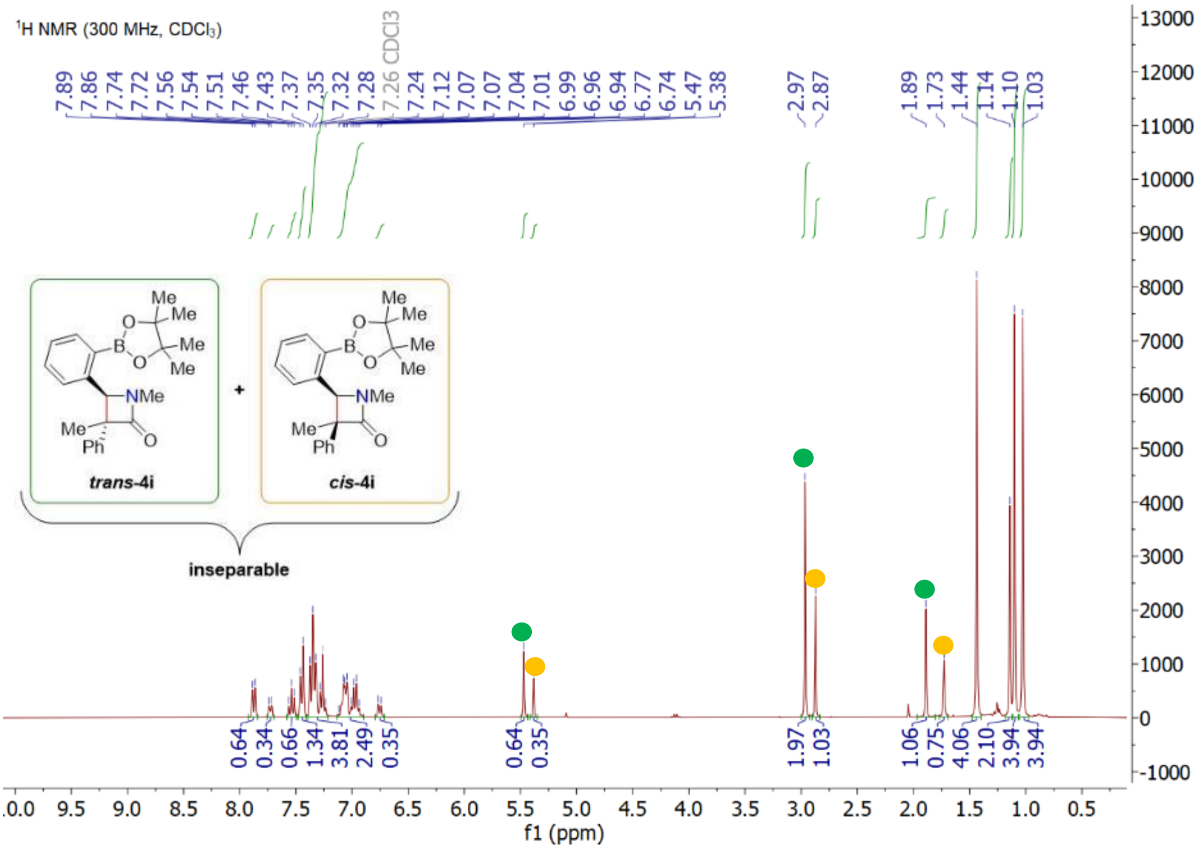
1H NMR (300 MHz, CDCl3)
trans-4i
cis-4i
inseparable
f1 (ppm)

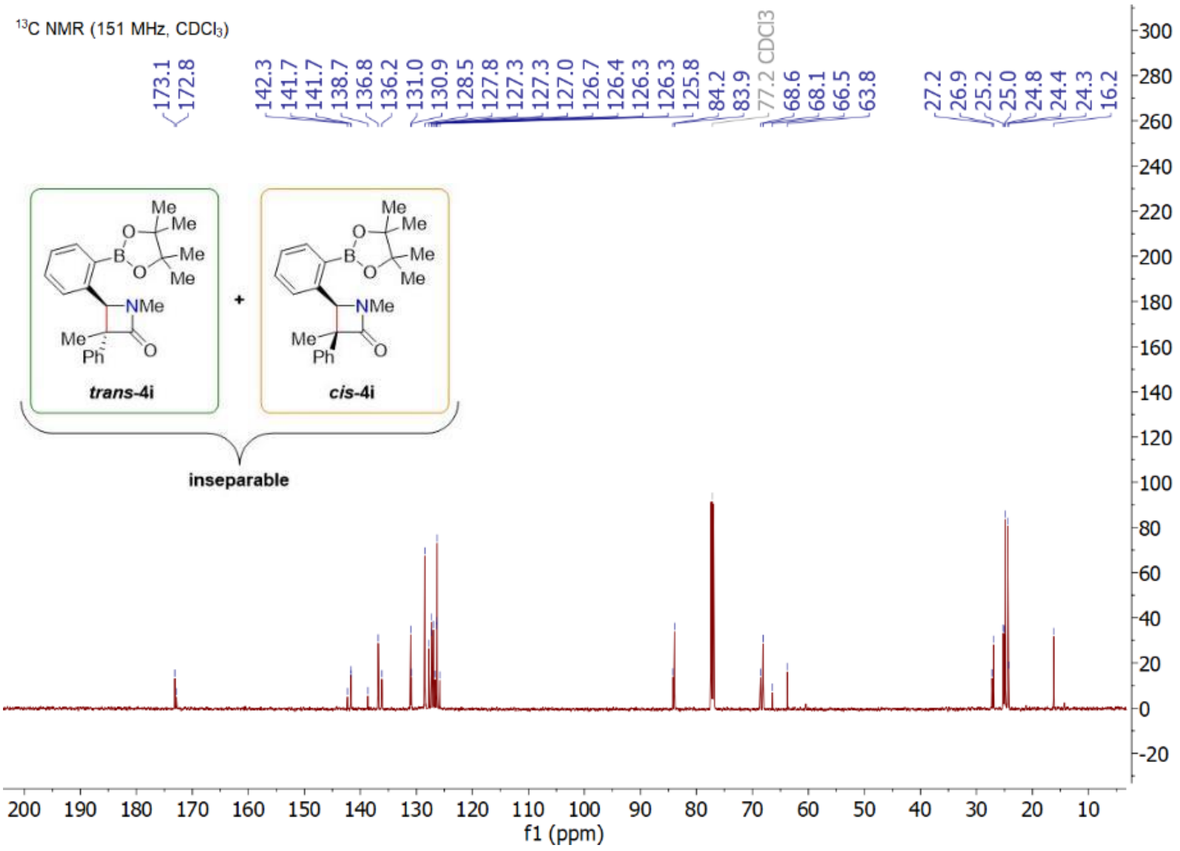
13C NMR (151 MHz, CDCl3)
trans-4i
cis-4i
inseparable
f1 (ppm)

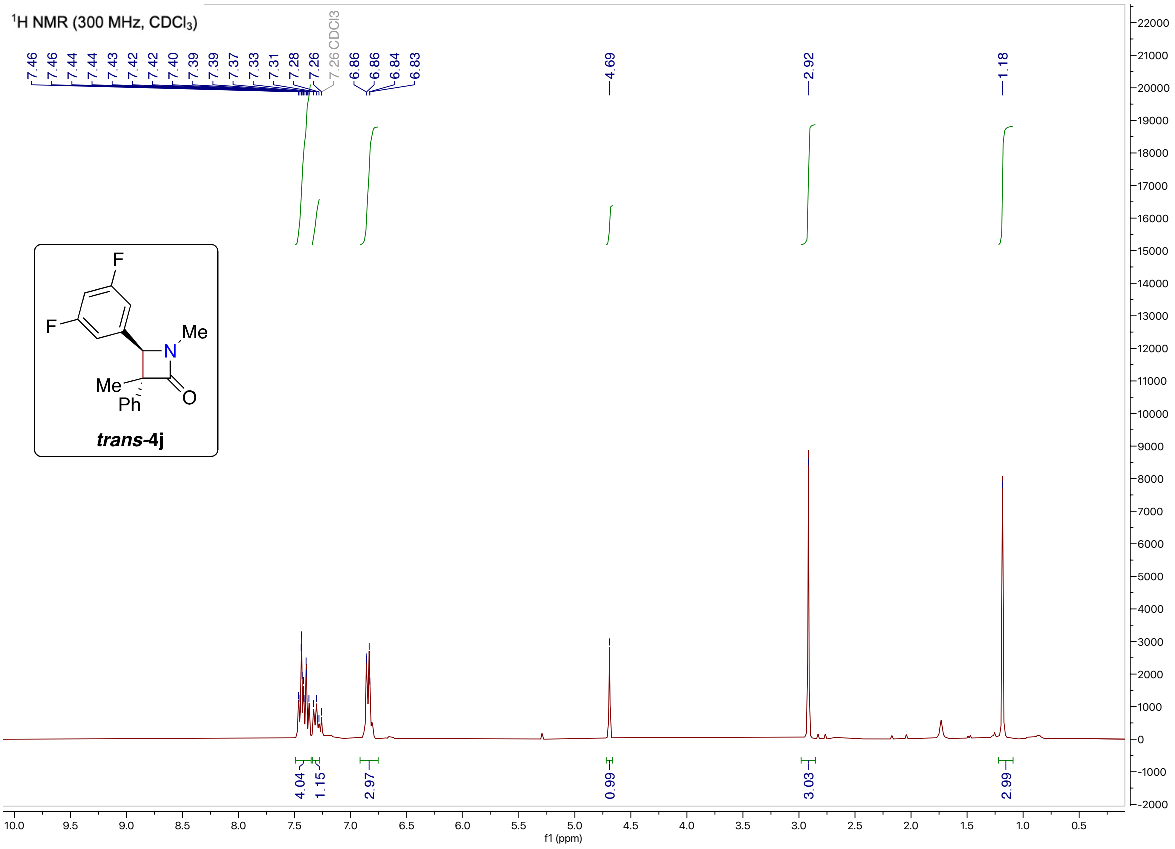

^{1}H NMR (300 MHz, CDCl$_3$)
7.46
7.46
7.44
7.44
7.43
7.42
7.42
7.40
7.39
7.39
7.37
7.33
7.31
7.28
7.26
7.26 CDCl3
6.86
6.86
6.84
6.83
4.69
2.92
1.18
F
F
N
Me
Me
Ph
O
trans-4j
4.04
1.15
2.97
0.99
3.03
2.99
f1 (ppm)

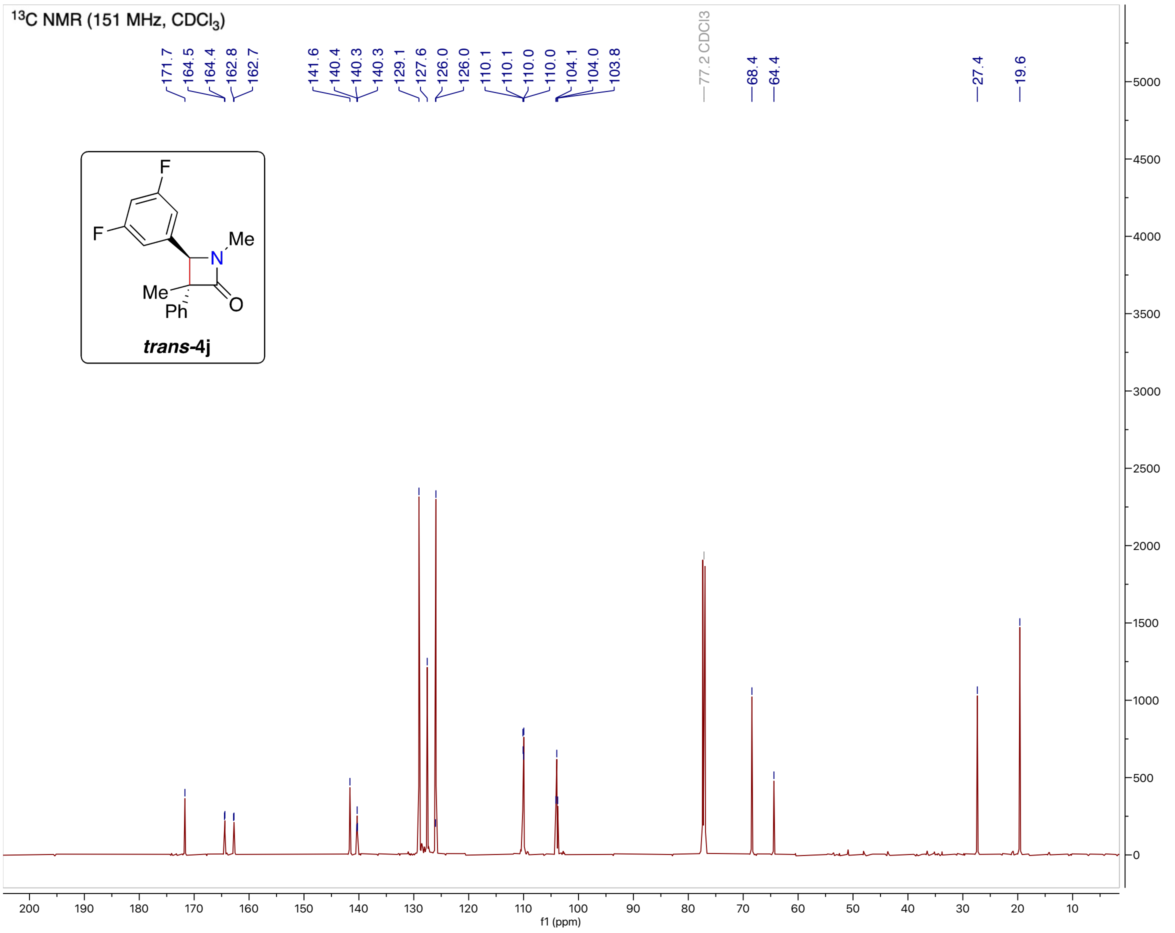

^{13}C NMR (151 MHz, CDCl$_3$)
171.7
164.5
164.4
162.8
162.7
141.6
140.4
140.3
140.3
129.1
127.6
126.0
126.0
110.1
110.1
110.0
110.0
104.1
104.0
103.8
77.2 CDCl3
68.4
64.4
27.4
19.6
F
F
N
Me
Me
Ph
O
trans-4j
f1 (ppm)

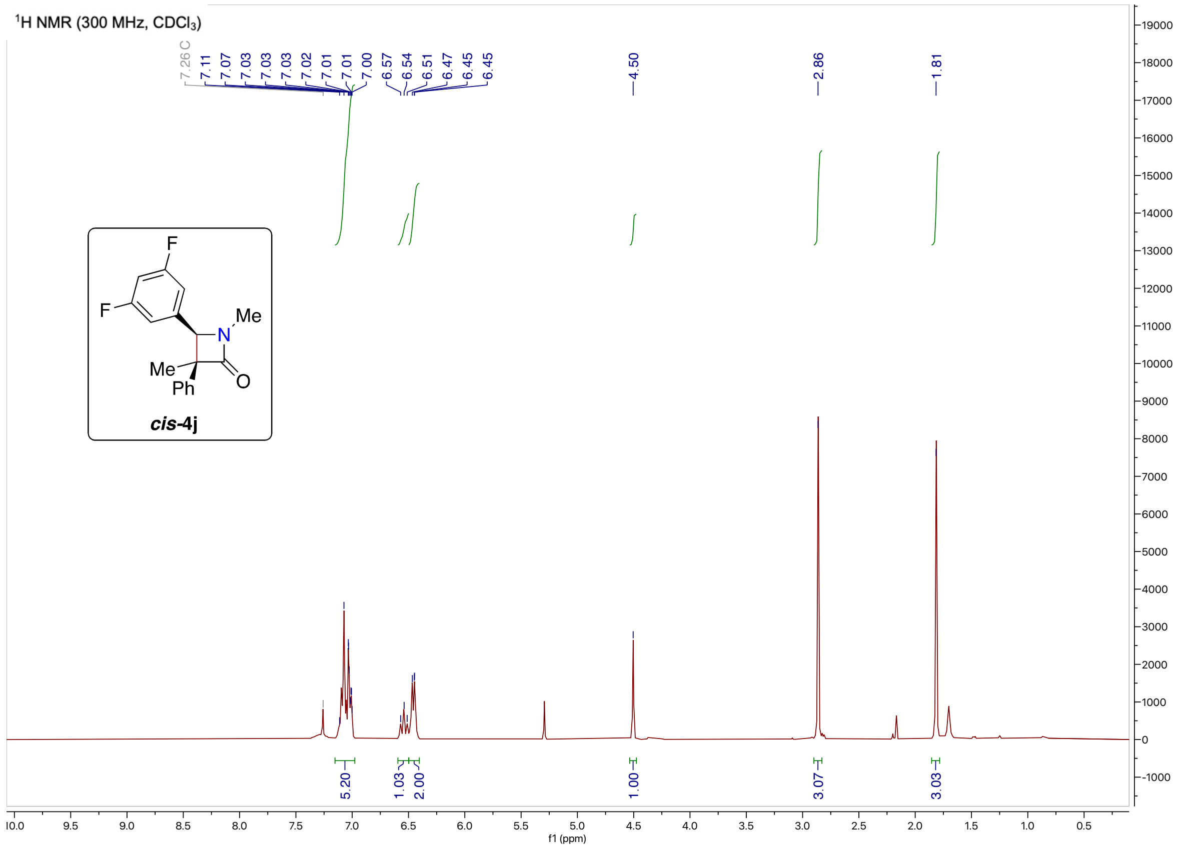
1H NMR (300 MHz, CDCl3)
7.26 C
7.11
7.07
7.03
7.03
7.03
7.02
7.01
7.01
7.00
6.57
6.54
6.51
6.47
6.45
6.45
4.50
2.86
1.81
5.20
1.03
2.00
1.00
3.07
3.03
cis-4j
f1 (ppm)

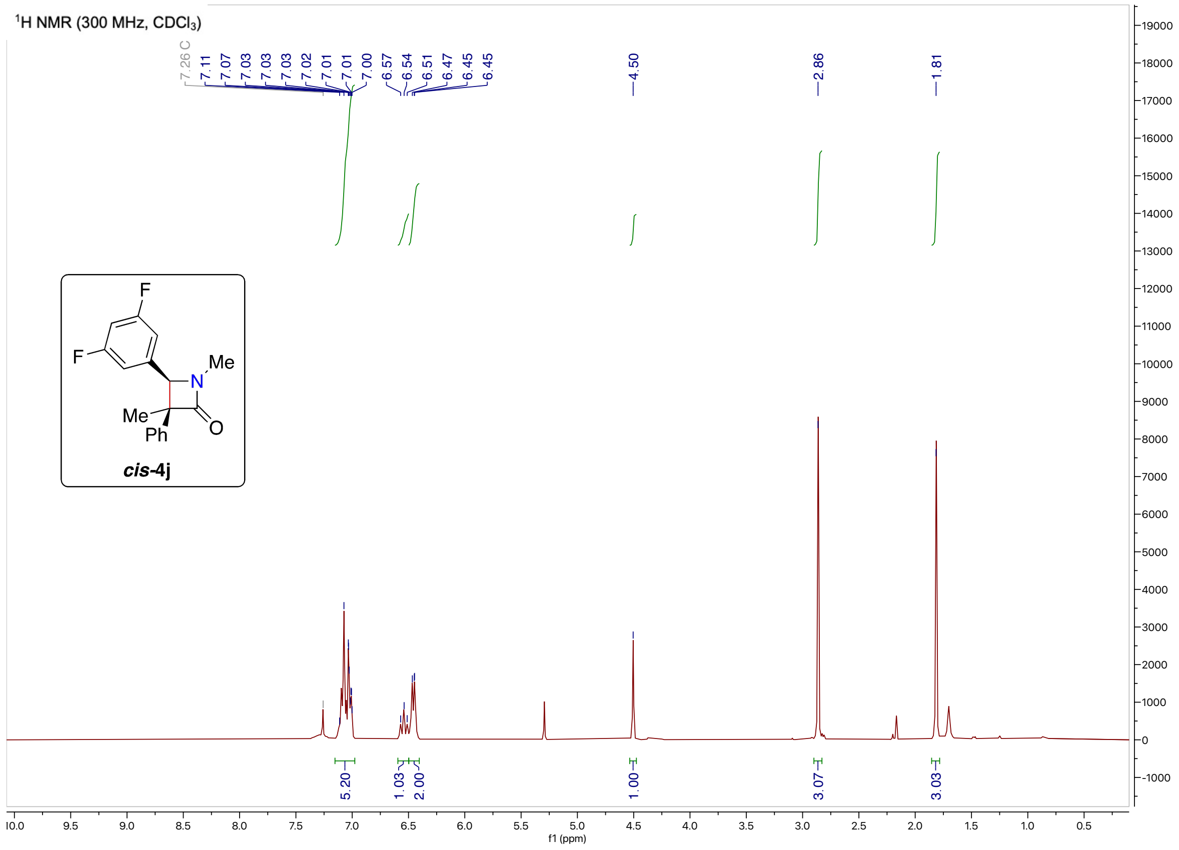
1H NMR (300 MHz, CDCl3)
7.26 C
7.11
7.07
7.03
7.03
7.03
7.02
7.01
7.01
7.00
6.57
6.54
6.51
6.47
6.45
6.45
4.50
2.86
1.81
5.20
1.03
2.00
1.00
3.07
3.03
cis-4j
f1 (ppm)

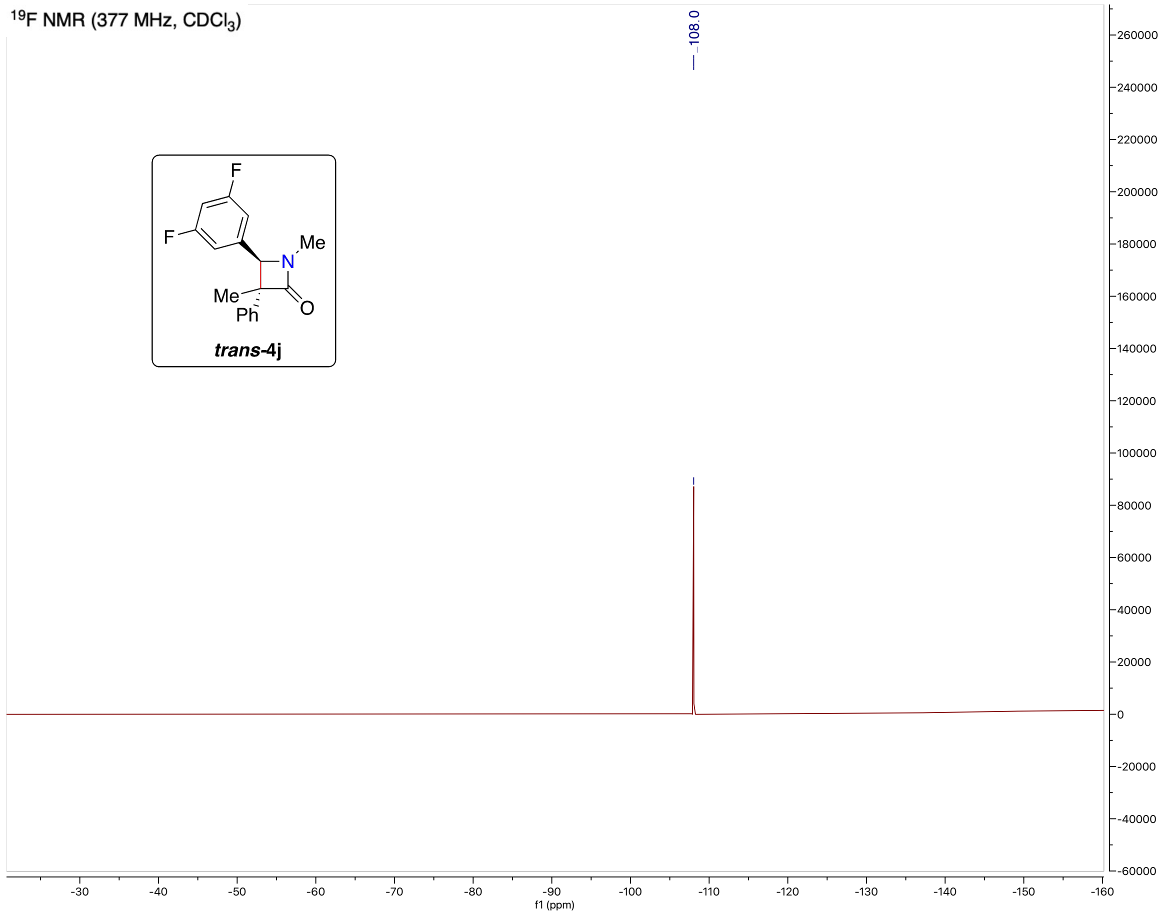
19F NMR (377 MHz, CDCl3)
-108.0
trans-4j
f1 (ppm)

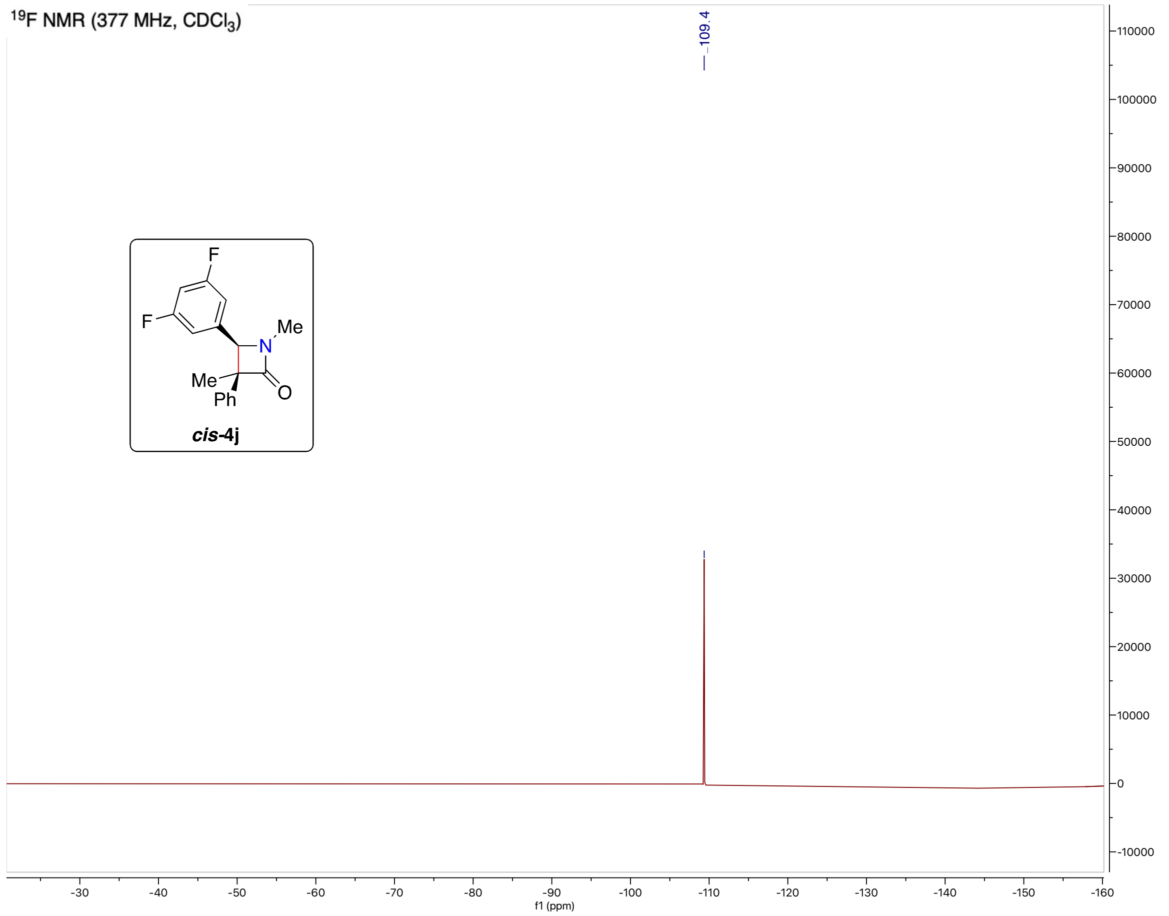
19F NMR (377 MHz, CDCl3)
-109.4
cis-4j
f1 (ppm)

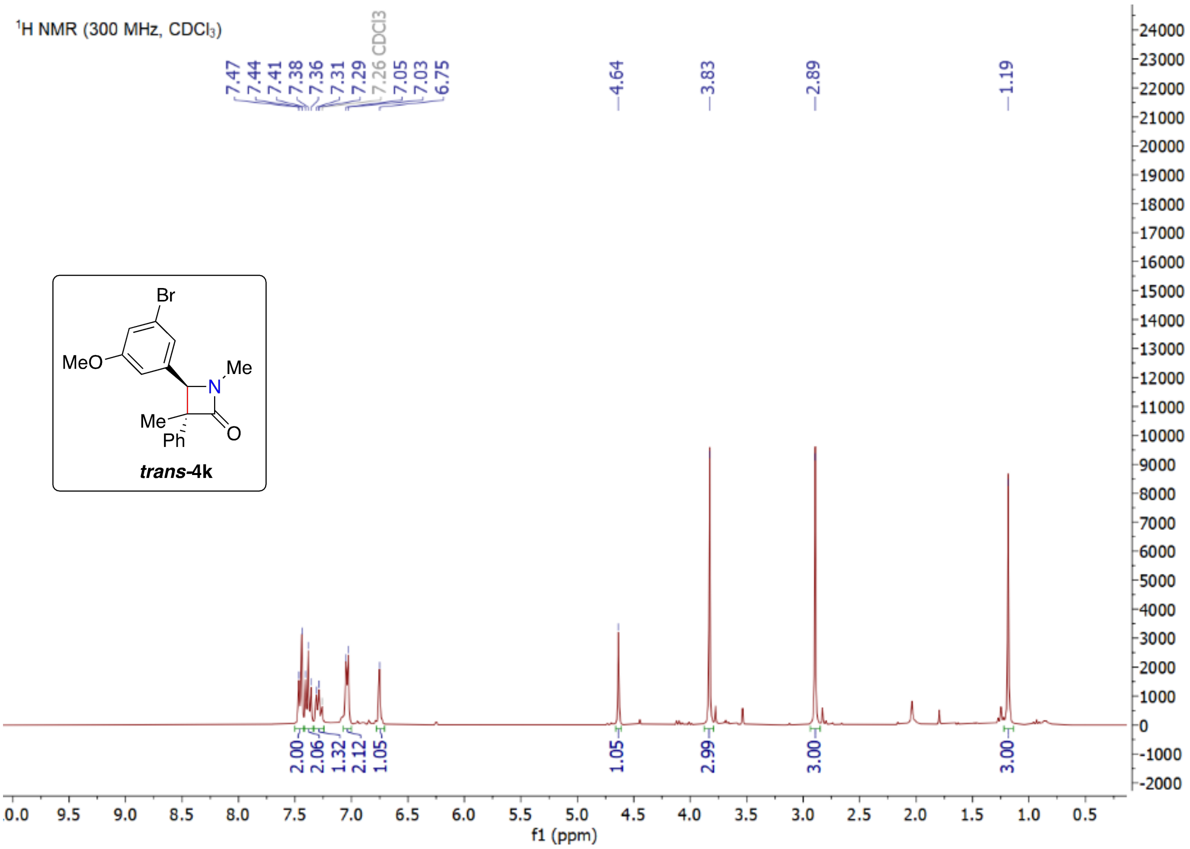

^{1}H NMR (300 MHz, $CDCl_3$)
7.47 7.44 7.41 7.38 7.36 7.31 7.29 7.26 CDCl3 7.05 7.03 6.75
4.64
3.83
2.89
1.19
2.00 2.06 1.32 2.12 1.05 1.05 2.99 3.00 3.00
Br
MeO
N
Me
Me
Ph
O
trans-4k
f1 (ppm)

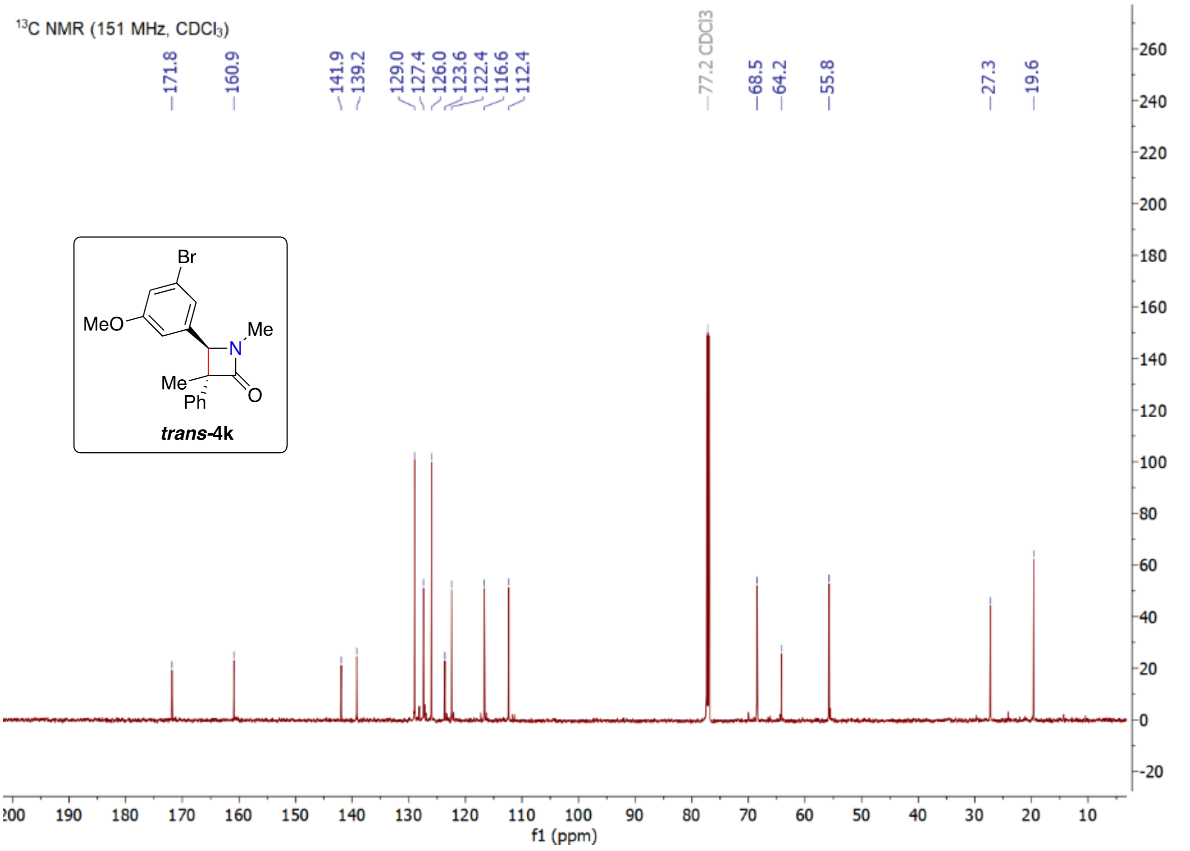

^{13}C NMR (151 MHz, $CDCl_3$)
171.8
160.9
141.9 139.2
129.0 127.4 126.0 123.6 122.4 116.6 112.4
77.2 CDCl3
68.5 64.2
55.8
27.3
19.6
Br
MeO
N
Me
Me
Ph
O
trans-4k
f1 (ppm)

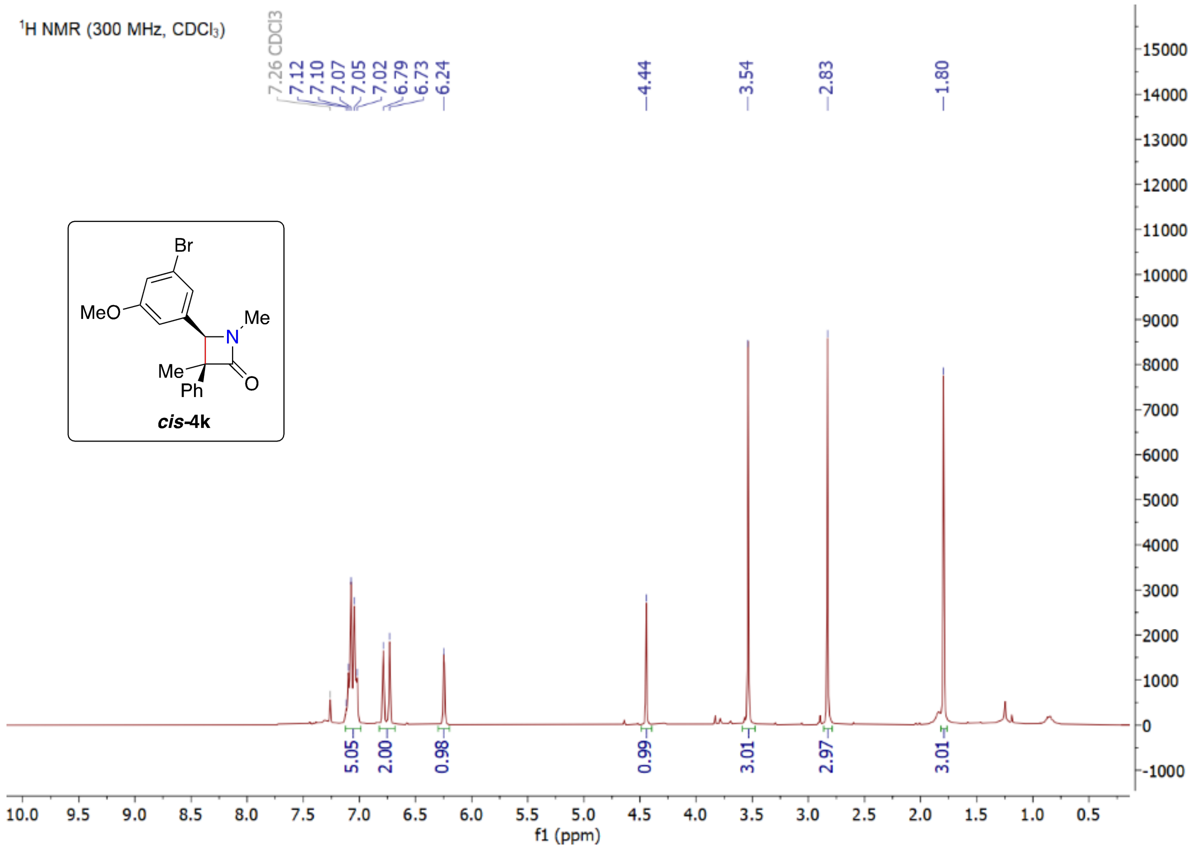

1H NMR (300 MHz, CDCl3)
7.26 CDCl3
7.12
7.10
7.07
7.05
7.02
6.79
6.73
6.24
4.44
3.54
2.83
1.80
5.05
2.00
0.98
0.99
3.01
2.97
3.01
Br
MeO
Me
N
Me
Ph
O
cis-4k
f1 (ppm)

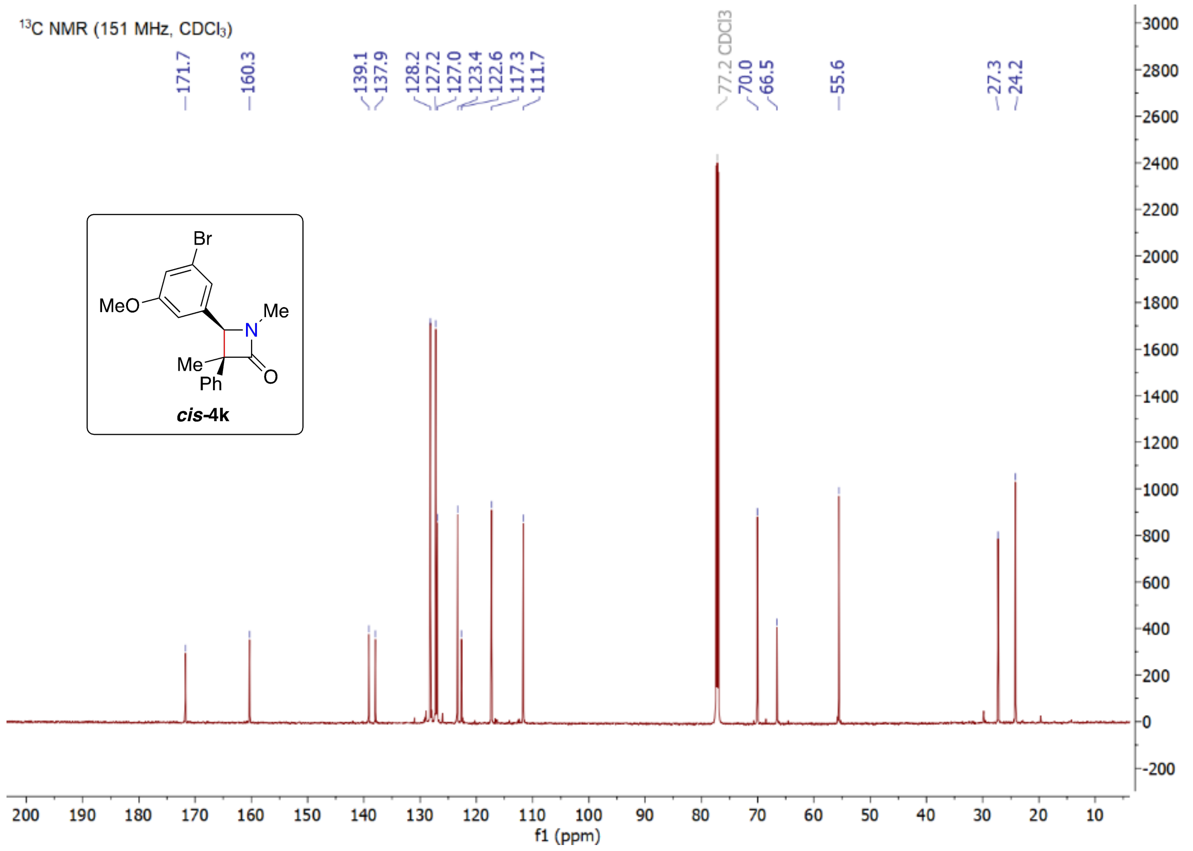

13C NMR (151 MHz, CDCl3)
171.7
160.3
139.1
137.9
128.2
127.2
127.0
123.4
122.6
117.3
111.7
77.2 CDCl3
70.0
66.5
55.6
27.3
24.2
Br
MeO
Me
N
Me
Ph
O
cis-4k
f1 (ppm)

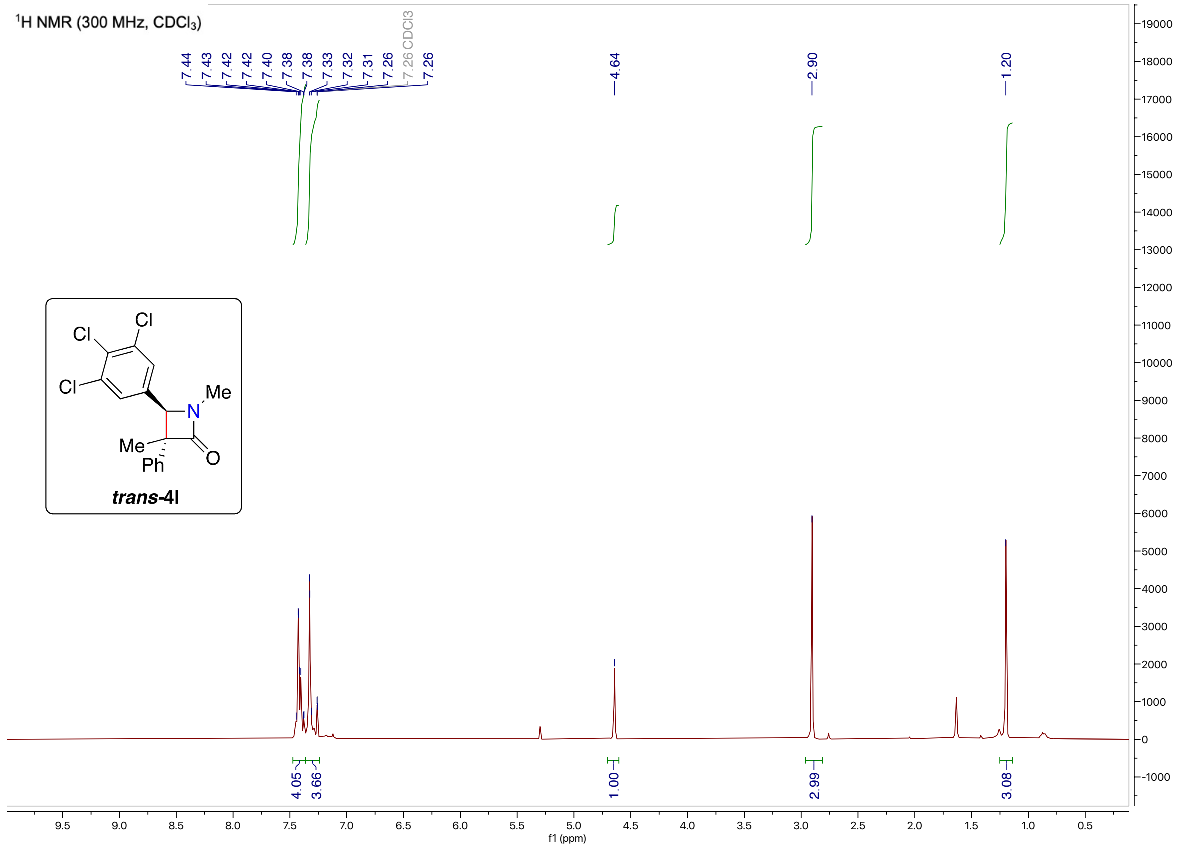

1H NMR (300 MHz, CDCl3)
7.44
7.43
7.42
7.42
7.40
7.38
7.38
7.33
7.32
7.31
7.26
7.26 CDCl3
7.26
4.64
2.90
1.20
4.05
3.66
1.00
2.99
3.08
f1 (ppm)
Cl
Cl
Cl
N
Me
Me
Ph
O
trans-4l

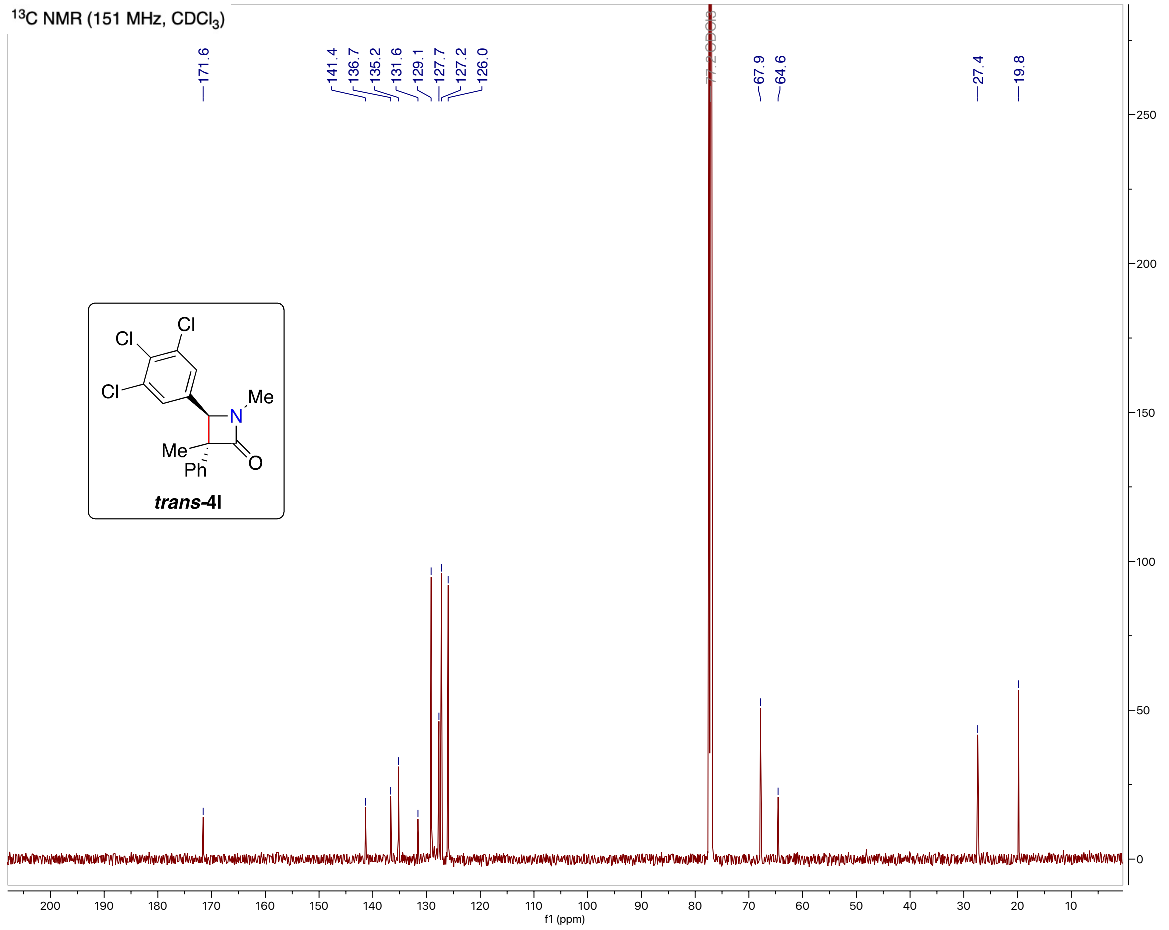

13C NMR (151 MHz, CDCl3)
171.6
141.4
136.7
135.2
131.6
129.1
127.7
127.2
126.0
77.2 CDCl3
67.9
64.6
27.4
19.8
f1 (ppm)
Cl
Cl
Cl
N
Me
Me
Ph
O
trans-4l

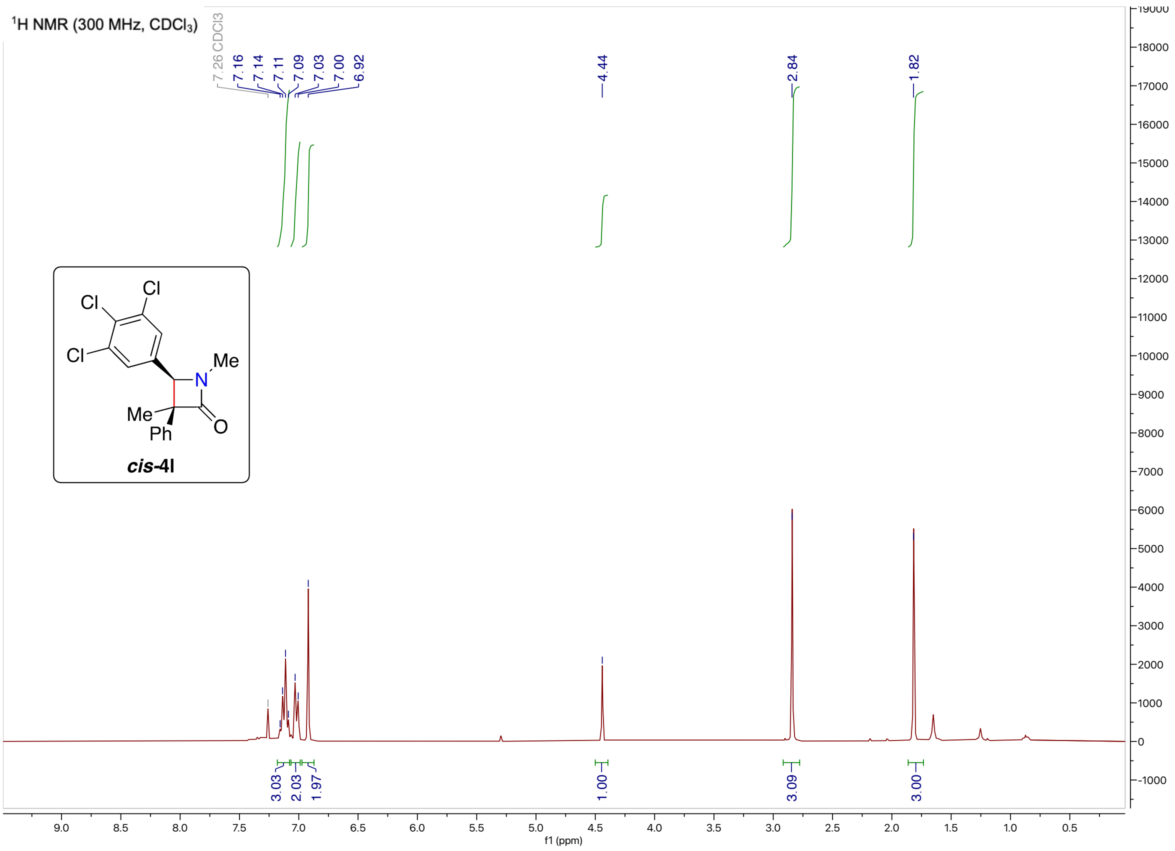

1H NMR (300 MHz, CDCl3)
7.26 CDCl3
7.16
7.14
7.11
7.09
7.03
7.00
6.92
4.44
2.84
1.82
3.03
2.03
1.97
1.00
3.09
3.00
f1 (ppm)
cis-4l

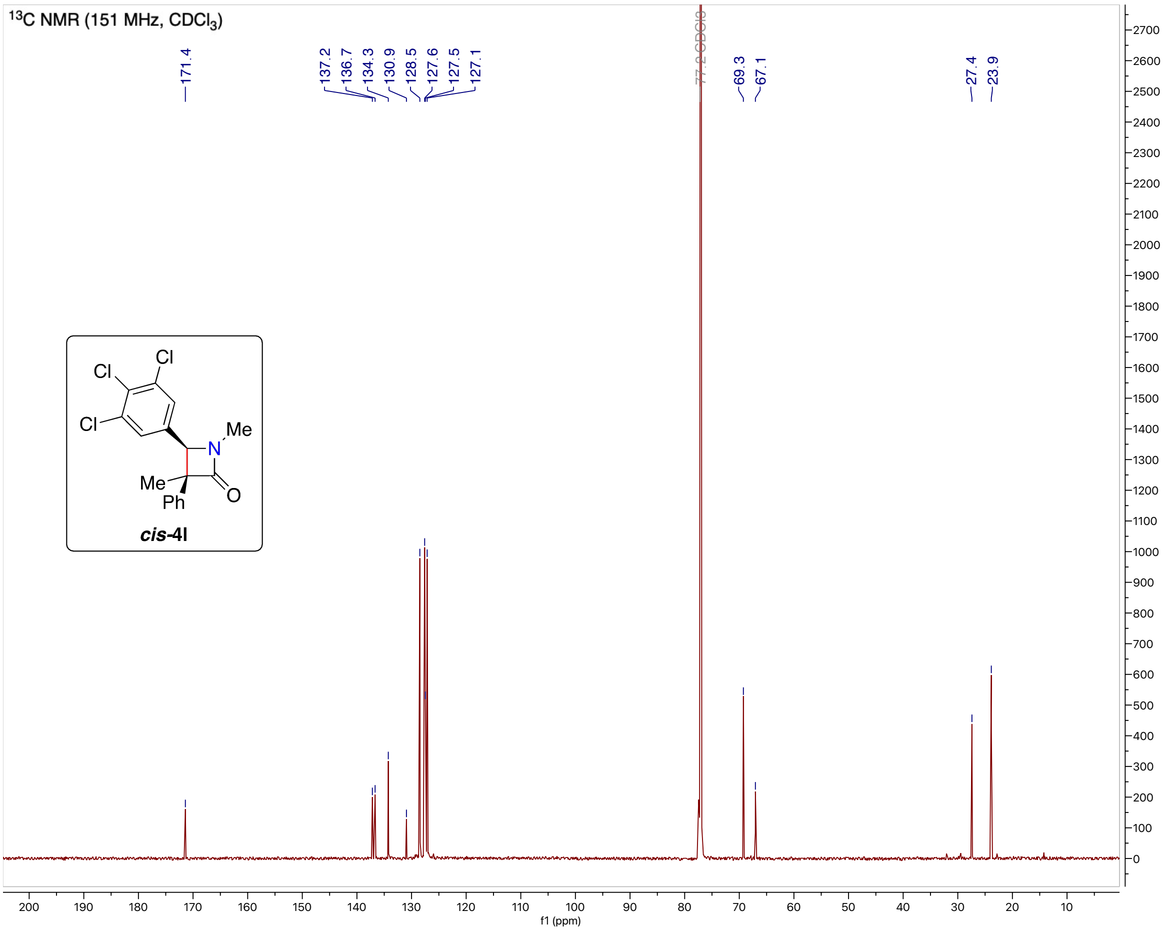

13C NMR (151 MHz, CDCl3)
171.4
137.2
136.7
134.3
130.9
128.5
127.6
127.5
127.1
77.2 CDCl3
69.3
67.1
27.4
23.9
f1 (ppm)
cis-4l

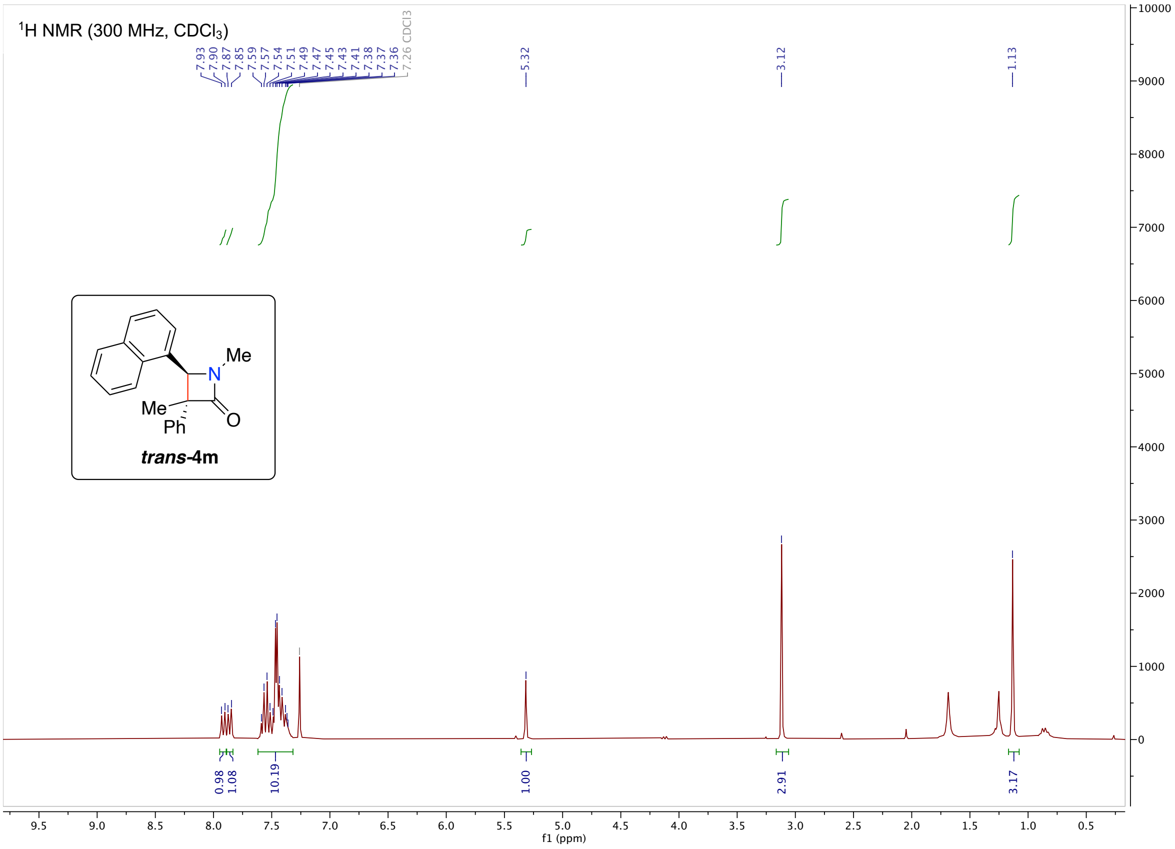
1H NMR (300 MHz, CDCl3)
7.93
7.90
7.87
7.85
7.59
7.57
7.54
7.51
7.49
7.47
7.45
7.43
7.41
7.38
7.37
7.36
7.26 CDCl3
5.32
3.12
1.13
Me
N
Me
Ph
O
trans-4m
0.98
1.08
10.19
1.00
2.91
3.17
f1 (ppm)

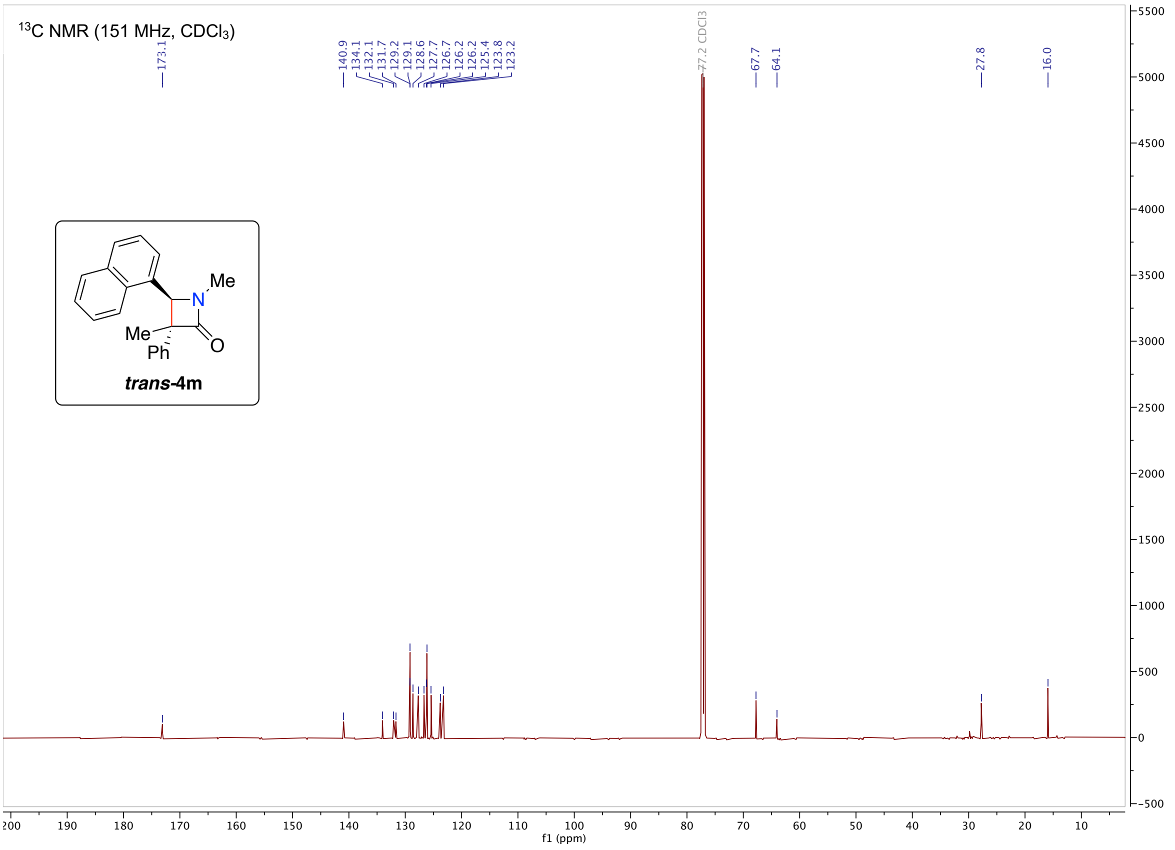
13C NMR (151 MHz, CDCl3)
173.1
140.9
134.1
132.1
131.7
129.2
129.1
128.6
127.7
126.7
126.2
126.2
125.4
123.8
123.2
77.2 CDCl3
67.7
64.1
27.8
16.0
Me
N
Me
Ph
O
trans-4m
f1 (ppm)

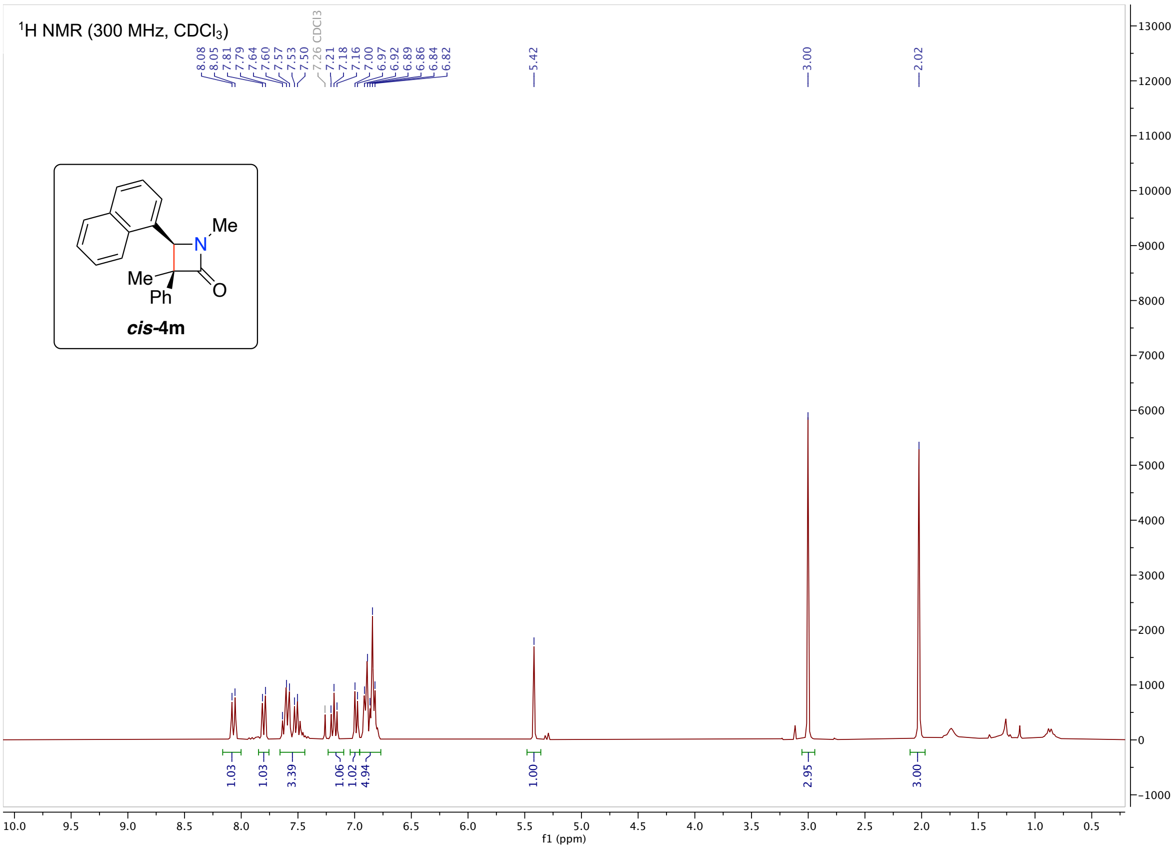

^{1}H NMR (300 MHz, CDCl$_3$)
8.08 8.05 7.81 7.79 7.64 7.60 7.57 7.53 7.50 7.26 CDCl3 7.21 7.18 7.16 7.00 6.97 6.92 6.89 6.86 6.84 6.82
5.42
3.00
2.02
Me
N
Me
Ph
O
cis-4m
1.03 1.03 3.39 1.06 1.02 4.94 1.00 2.95 3.00
f1 (ppm)

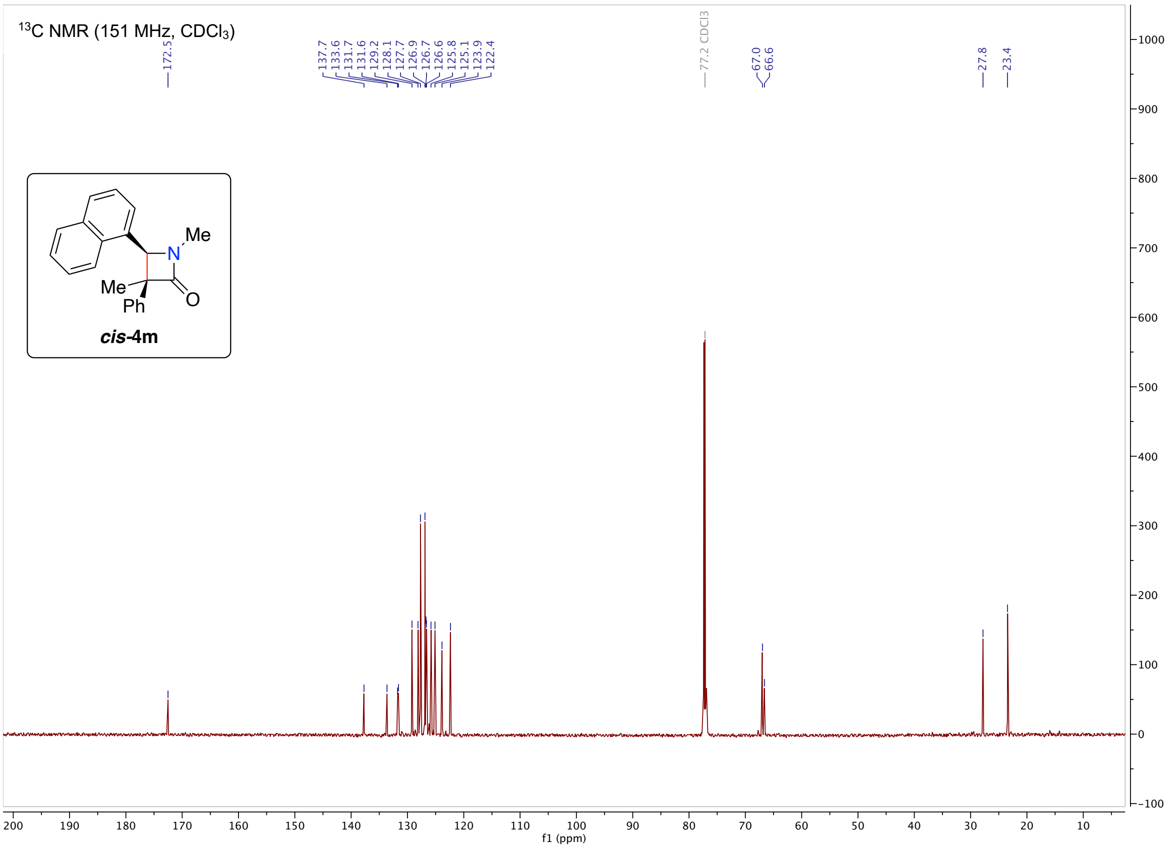

^{13}C NMR (151 MHz, CDCl$_3$)
172.5
137.7 133.6 131.7 131.6 129.2 128.1 127.7 126.9 126.7 126.6 125.8 125.1 123.9 122.4
77.2 CDCl3
67.0 66.6
27.8
23.4
Me
N
Me
Ph
O
cis-4m
f1 (ppm)

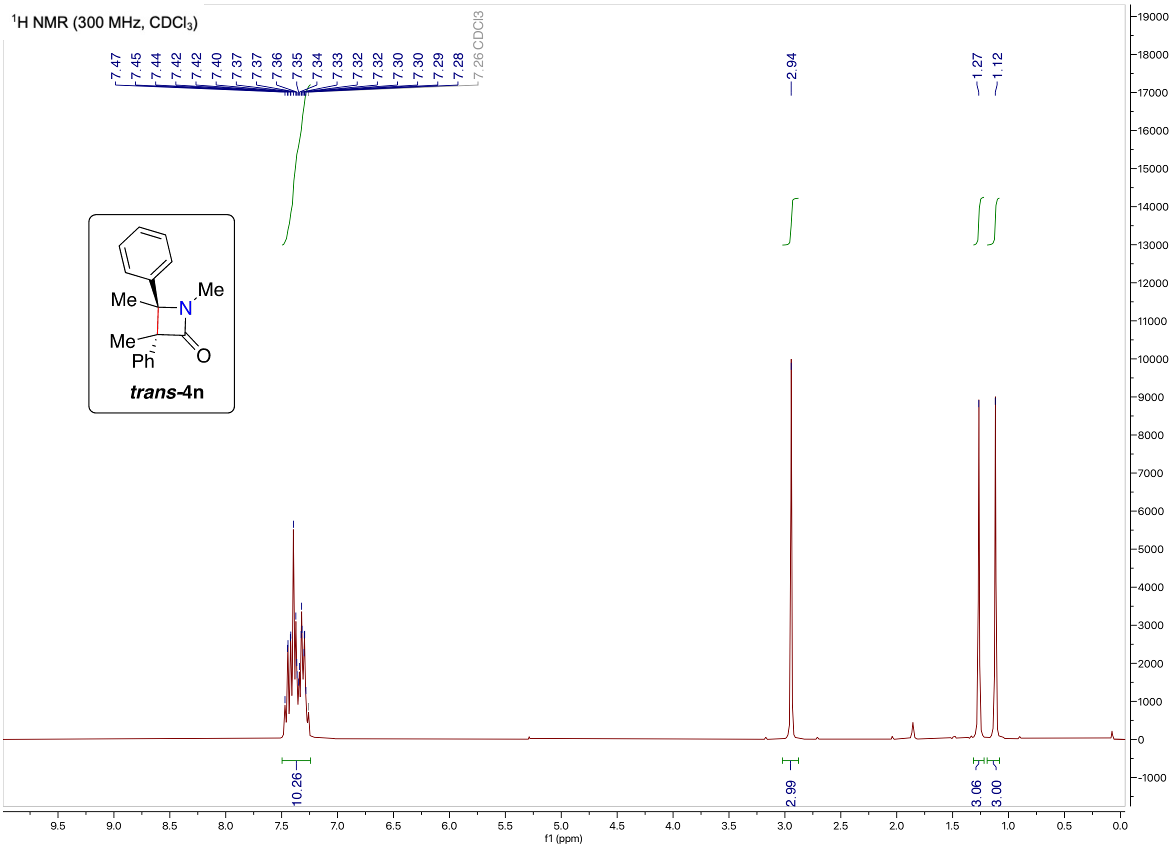

^{1}H NMR (300 MHz, CDCl$_3$)
7.47 7.45 7.44 7.42 7.42 7.40 7.37 7.37 7.36 7.35 7.34 7.33 7.32 7.32 7.30 7.30 7.29 7.28
7.26 CDCl3
2.94
1.27
1.12
Me
Me
N
Me
Ph
O
trans-4n
10.26
2.99
3.06
3.00
f1 (ppm)

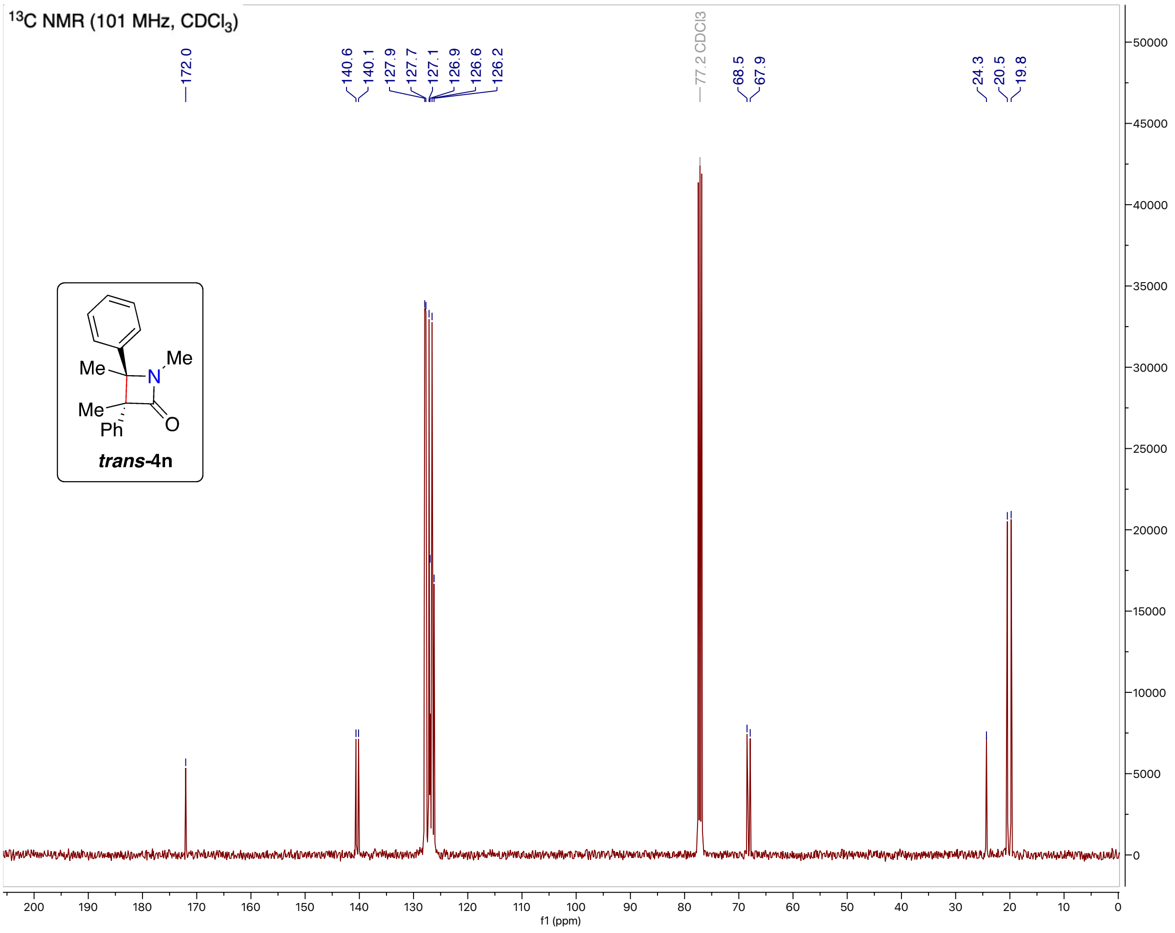

^{13}C NMR (101 MHz, CDCl$_3$)
172.0
140.6 140.1 127.9 127.7 127.1 126.9 126.6 126.2
77.2 CDCl3
68.5 67.9
24.3 20.5 19.8
Me
Me
N
Me
Ph
O
trans-4n
f1 (ppm)

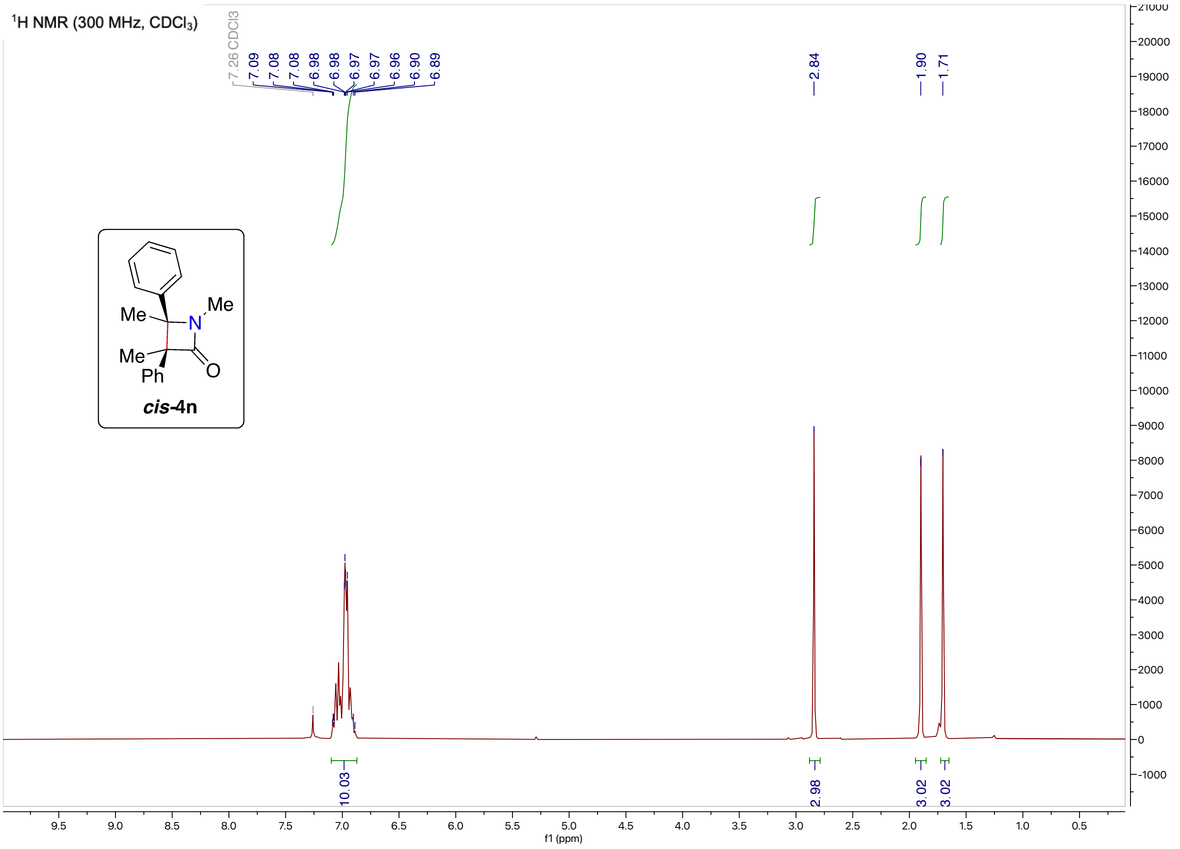

^{1}H NMR (300 MHz, CDCl$_3$)
7.26 CDCl3
7.09
7.08
7.08
6.98
6.98
6.97
6.97
6.96
6.90
6.89
2.84
1.90
1.71
10.03
2.98
3.02
3.02
Me
N
Me
Me
Ph
O
cis-4n
f1 (ppm)

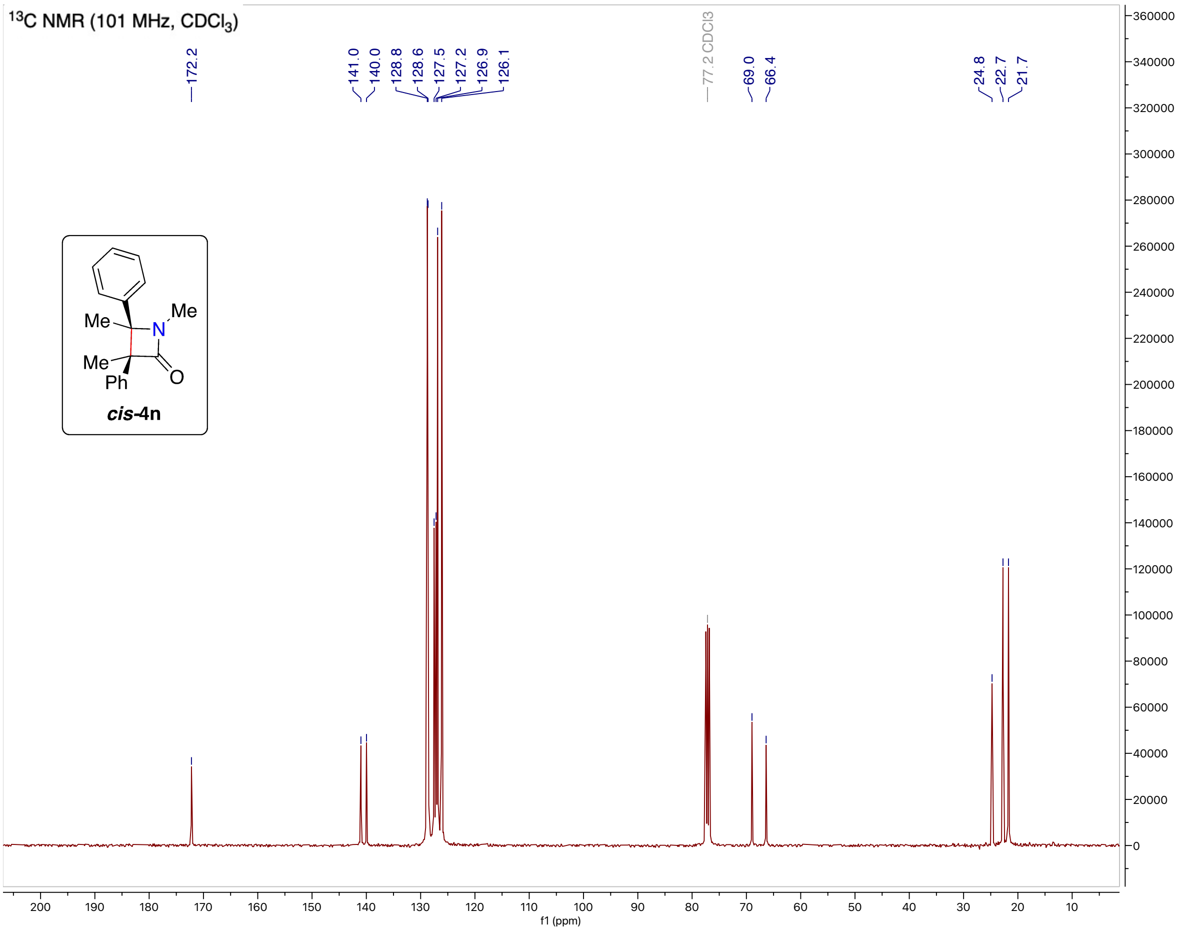

^{13}C NMR (101 MHz, CDCl$_3$)
172.2
141.0
140.0
128.8
128.6
127.5
127.2
126.9
126.1
77.2 CDCl3
69.0
66.4
24.8
22.7
21.7
Me
N
Me
Me
Ph
O
cis-4n
f1 (ppm)

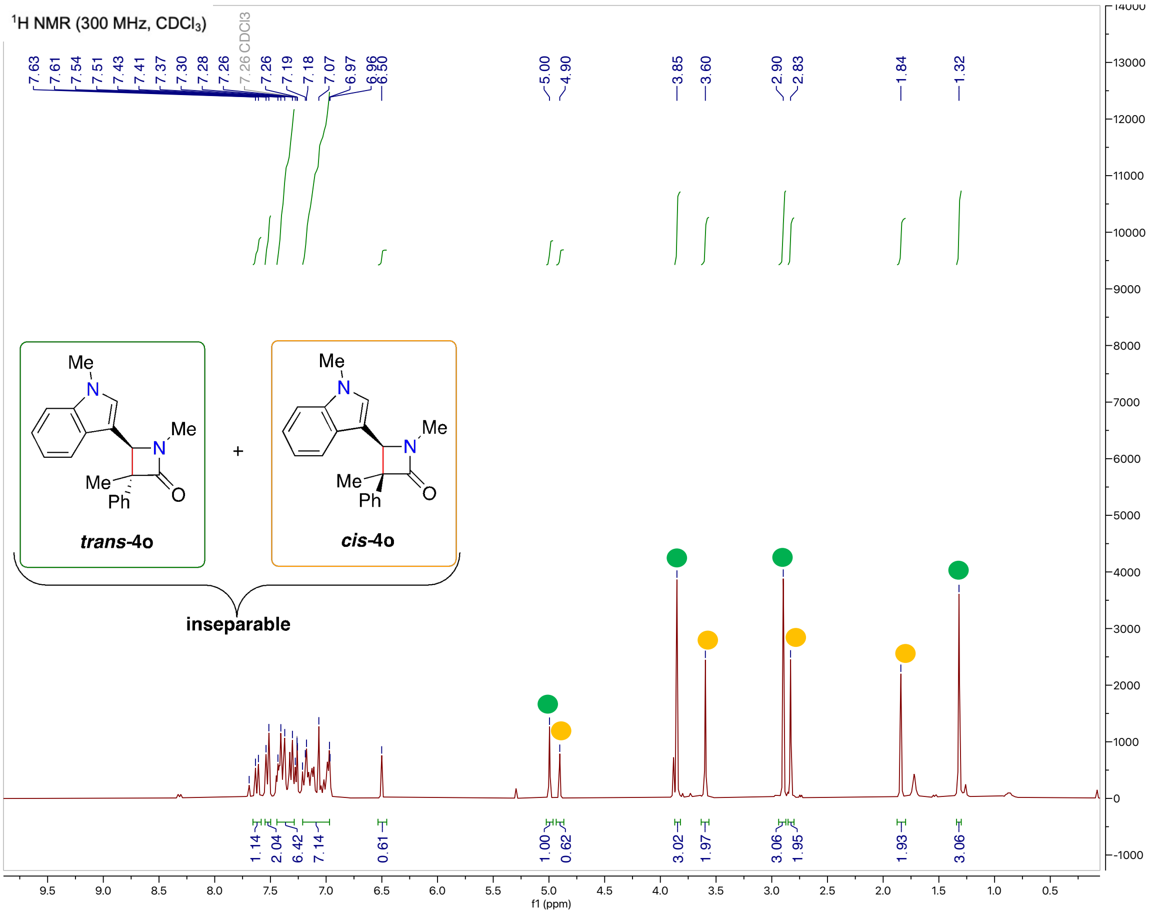

^{1}H NMR (300 MHz, CDCl$_3$)
trans-4o
cis-4o
inseparable
f1 (ppm)

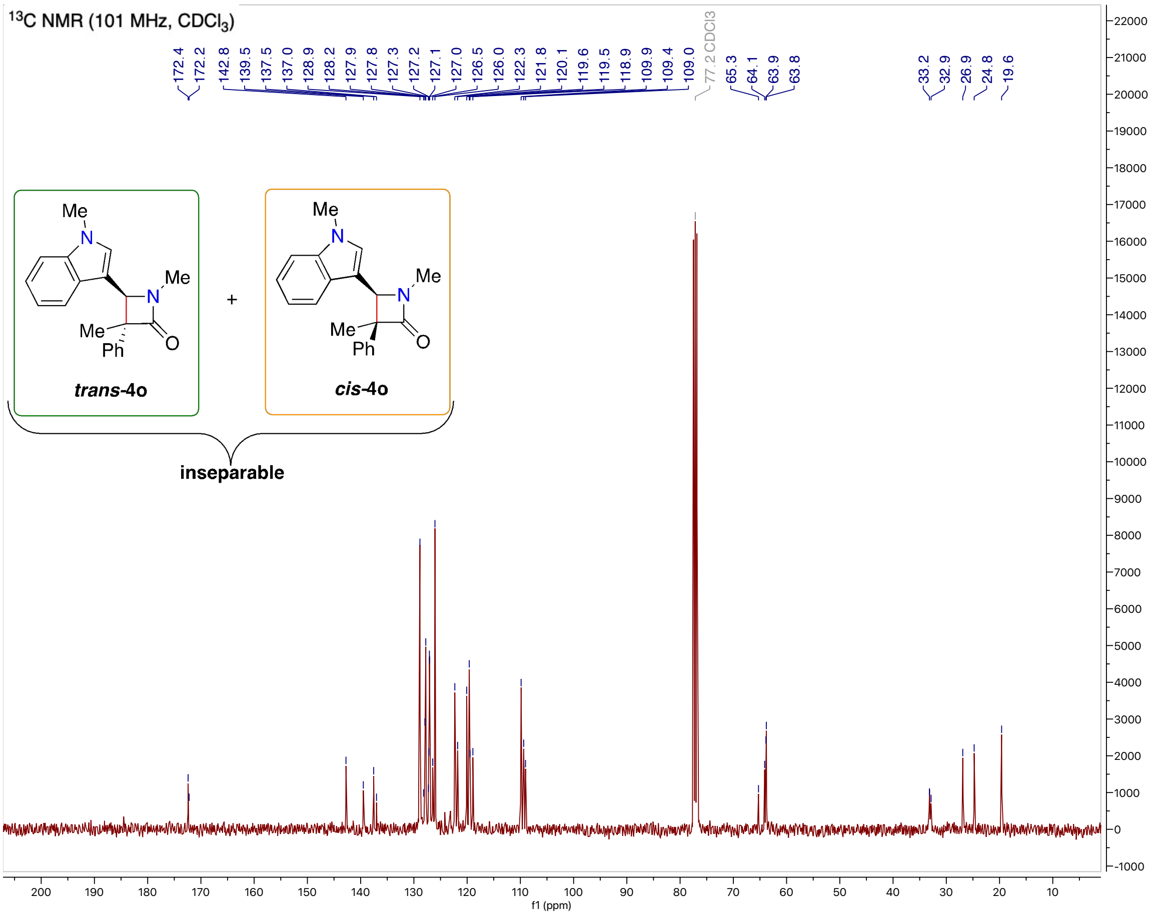

^{13}C NMR (101 MHz, CDCl$_3$)
trans-4o
cis-4o
inseparable
f1 (ppm)

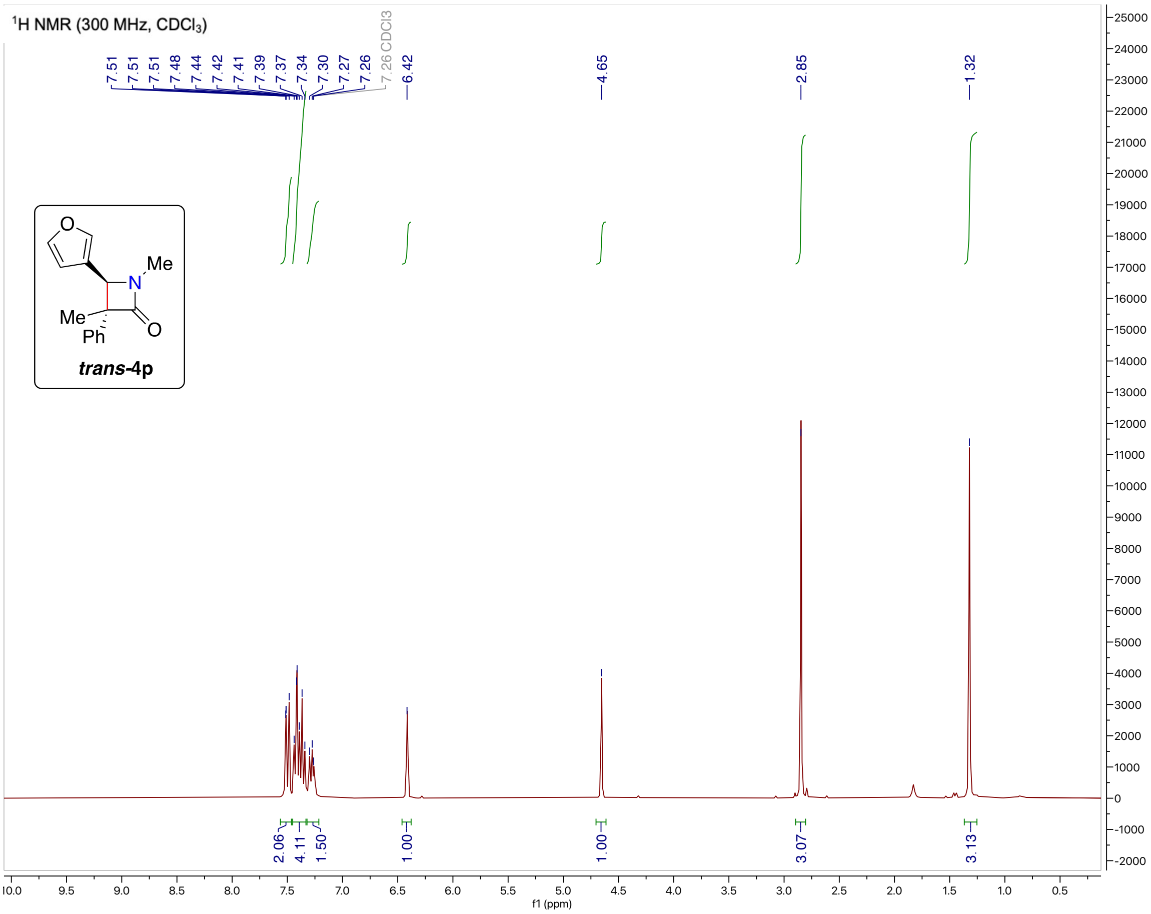

^{1}H NMR (300 MHz, CDCl$_3$)
7.51
7.51
7.51
7.48
7.44
7.42
7.41
7.39
7.37
7.34
7.30
7.27
7.26
7.26 CDCl3
6.42
4.65
2.85
1.32
2.06
4.11
1.50
1.00
1.00
3.07
3.13
Me
N
O
Me
Ph
trans-4p
f1 (ppm)

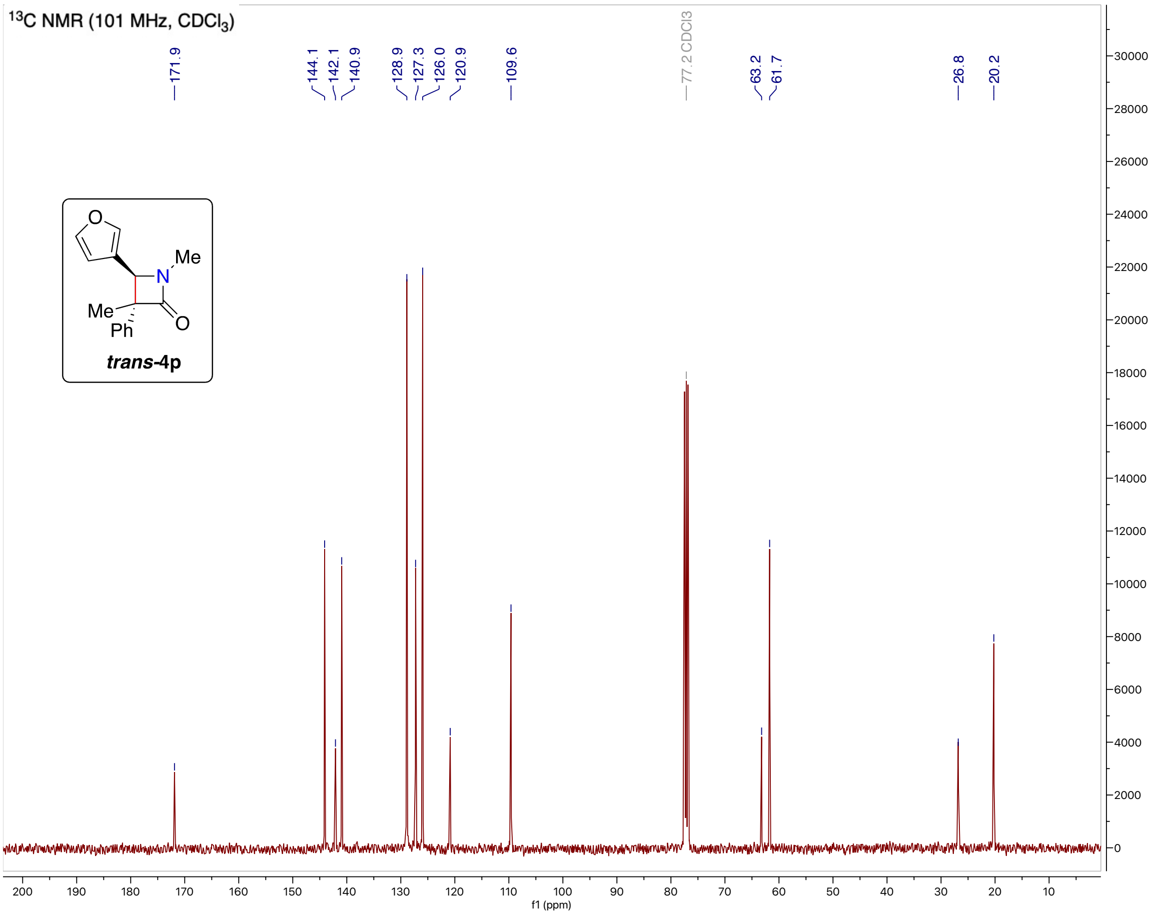

^{13}C NMR (101 MHz, CDCl$_3$)
171.9
144.1
142.1
140.9
128.9
127.3
126.0
120.9
109.6
77.2 CDCl3
63.2
61.7
26.8
20.2
Me
N
O
Me
Ph
trans-4p
f1 (ppm)

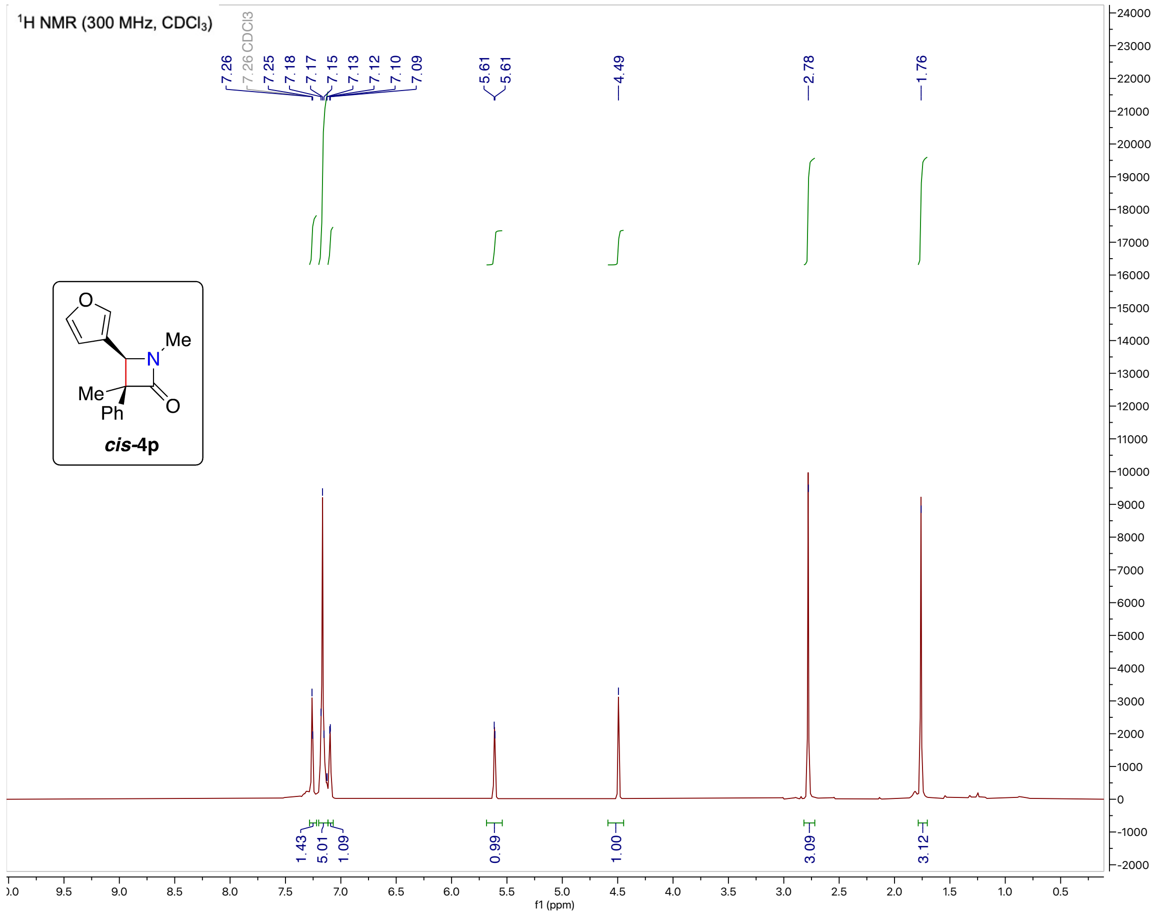

1H NMR (300 MHz, CDCl3)
7.26
7.26 CDCl3
7.25
7.18
7.17
7.15
7.13
7.12
7.10
7.09
5.61
5.61
4.49
2.78
1.76
Me
N
Me
O
Ph
O
cis-4p
1.43
5.01
1.09
0.99
1.00
3.09
3.12
f1 (ppm)

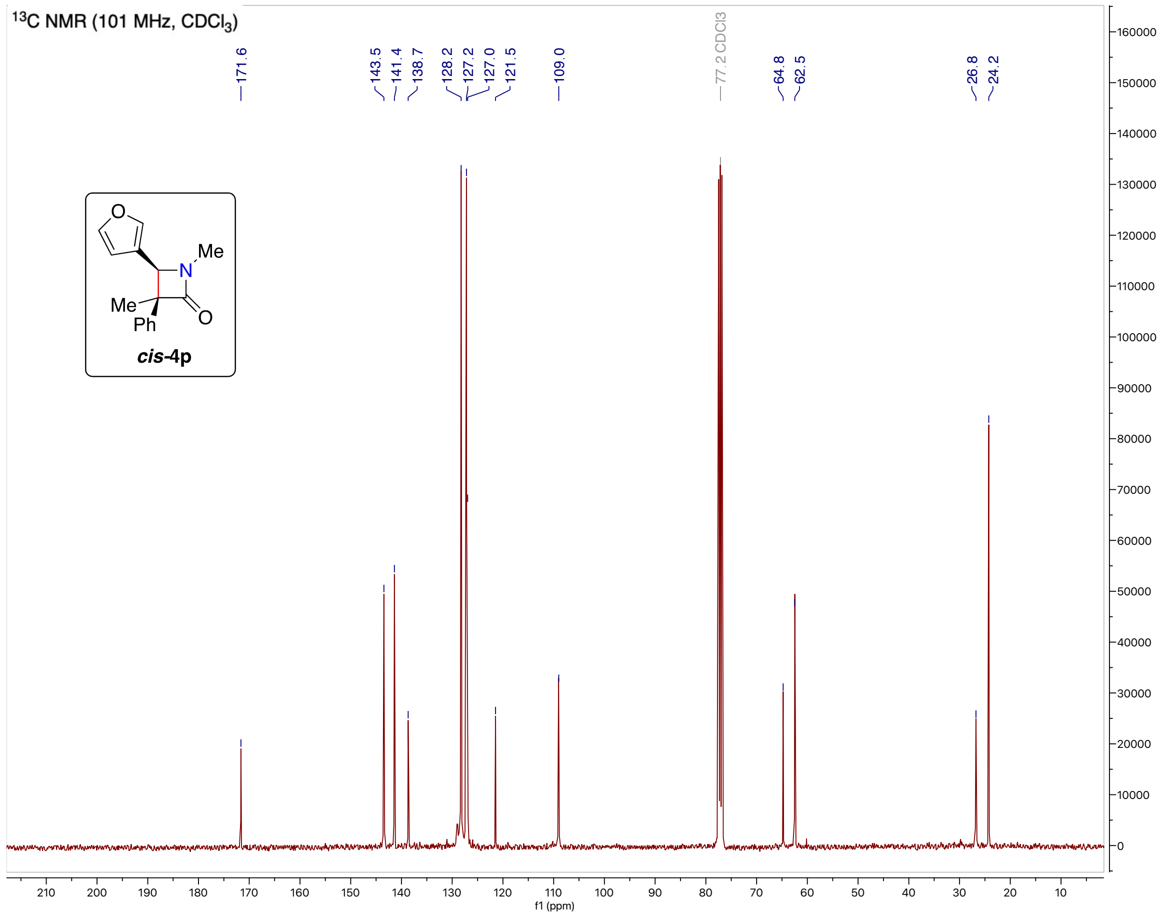

13C NMR (101 MHz, CDCl3)
171.6
143.5
141.4
138.7
128.2
127.2
127.0
121.5
109.0
77.2 CDCl3
64.8
62.5
26.8
24.2
Me
N
Me
O
Ph
O
cis-4p
f1 (ppm)

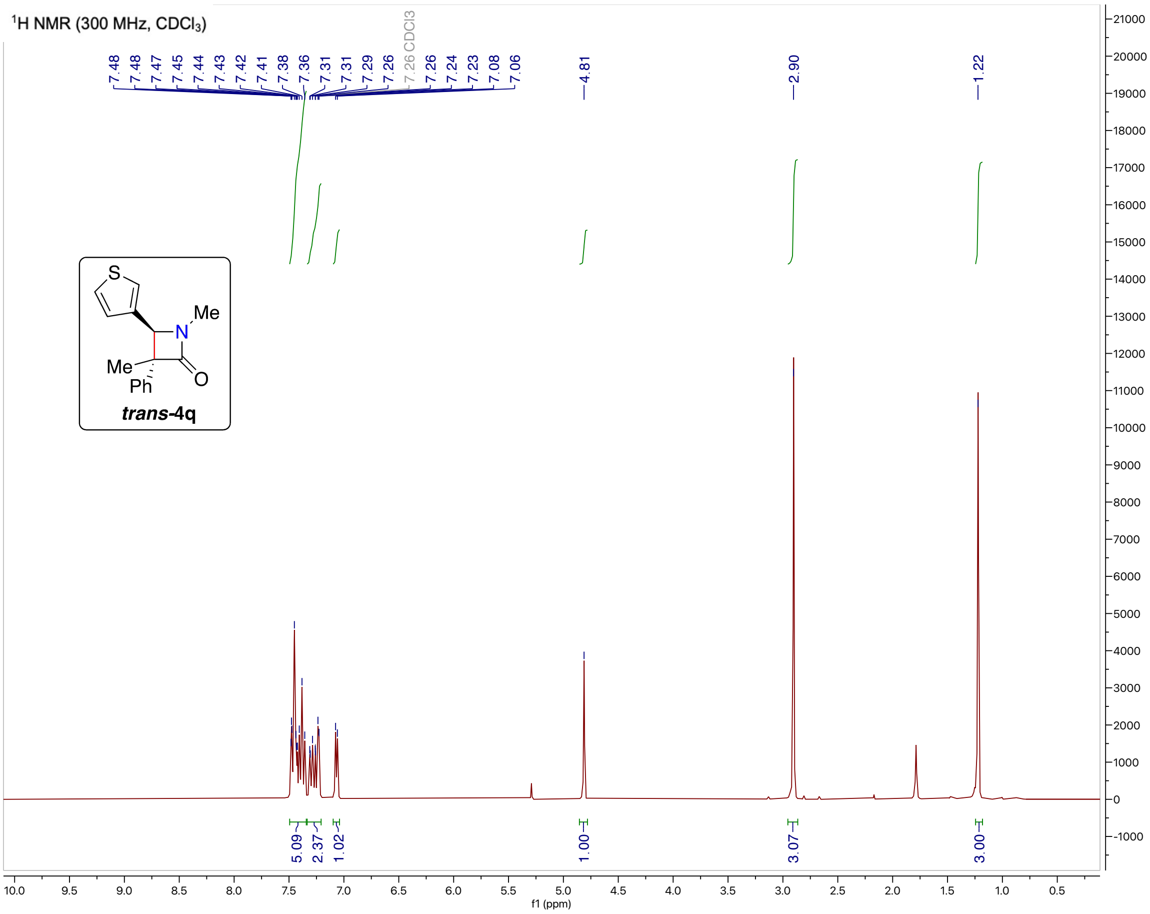

1H NMR (300 MHz, CDCl3)
7.48 7.48 7.47 7.45 7.44 7.43 7.42 7.41 7.38 7.36 7.31 7.31 7.29 7.26 7.26 CDCl3 7.26 7.24 7.23 7.08 7.06
4.81
2.90
1.22
trans-4q
5.09 2.37 1.02 1.00 3.07 3.00
f1 (ppm)

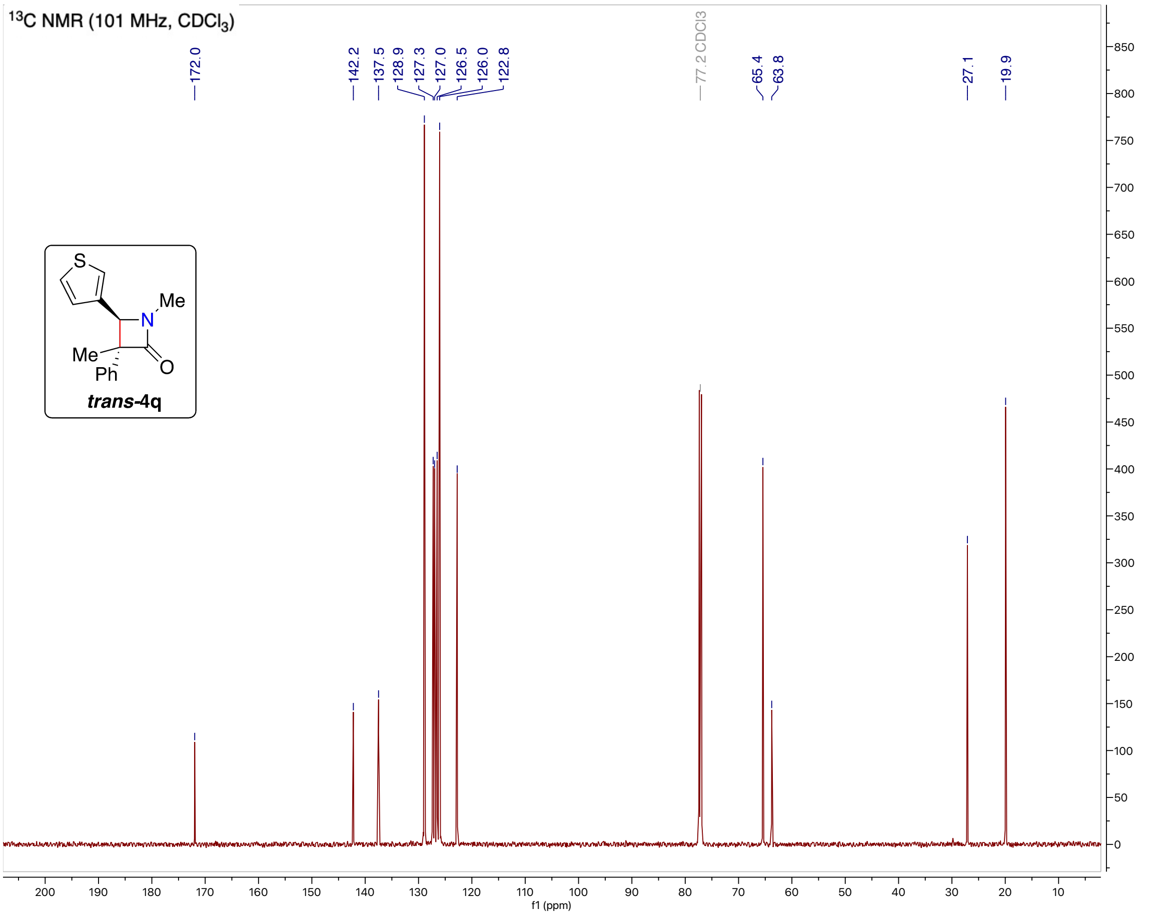

13C NMR (101 MHz, CDCl3)
172.0
142.2 137.5 128.9 127.3 127.0 126.5 126.0 122.8
77.2 CDCl3
65.4 63.8
27.1 19.9
trans-4q
f1 (ppm)

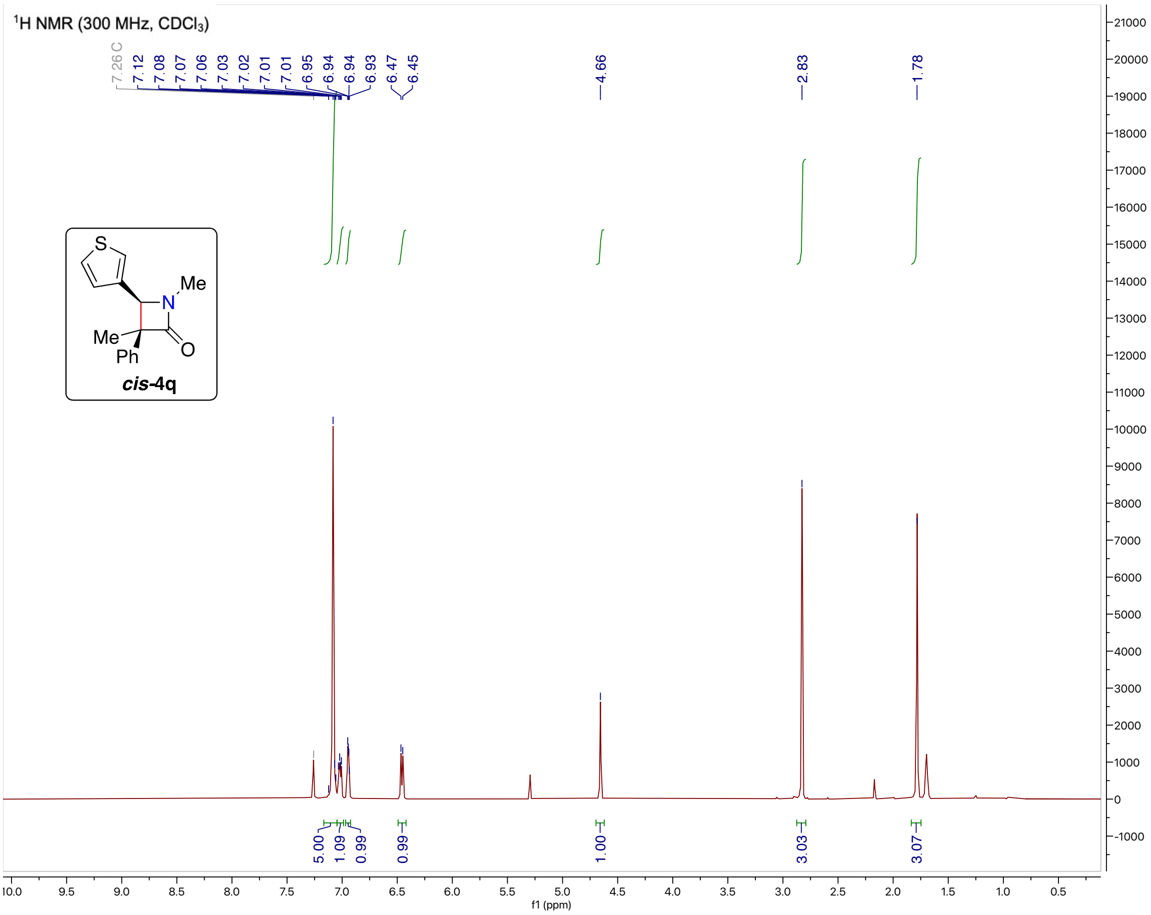

^{1}H NMR (300 MHz, CDCl$_3$)
7.26 C
7.12
7.08
7.07
7.06
7.03
7.02
7.01
7.01
6.95
6.94
6.94
6.93
6.47
6.45
4.66
2.83
1.78
S
N
Me
Me
Ph
O
cis-4q
5.00
1.09
0.99
0.99
1.00
3.03
3.07
f1 (ppm)

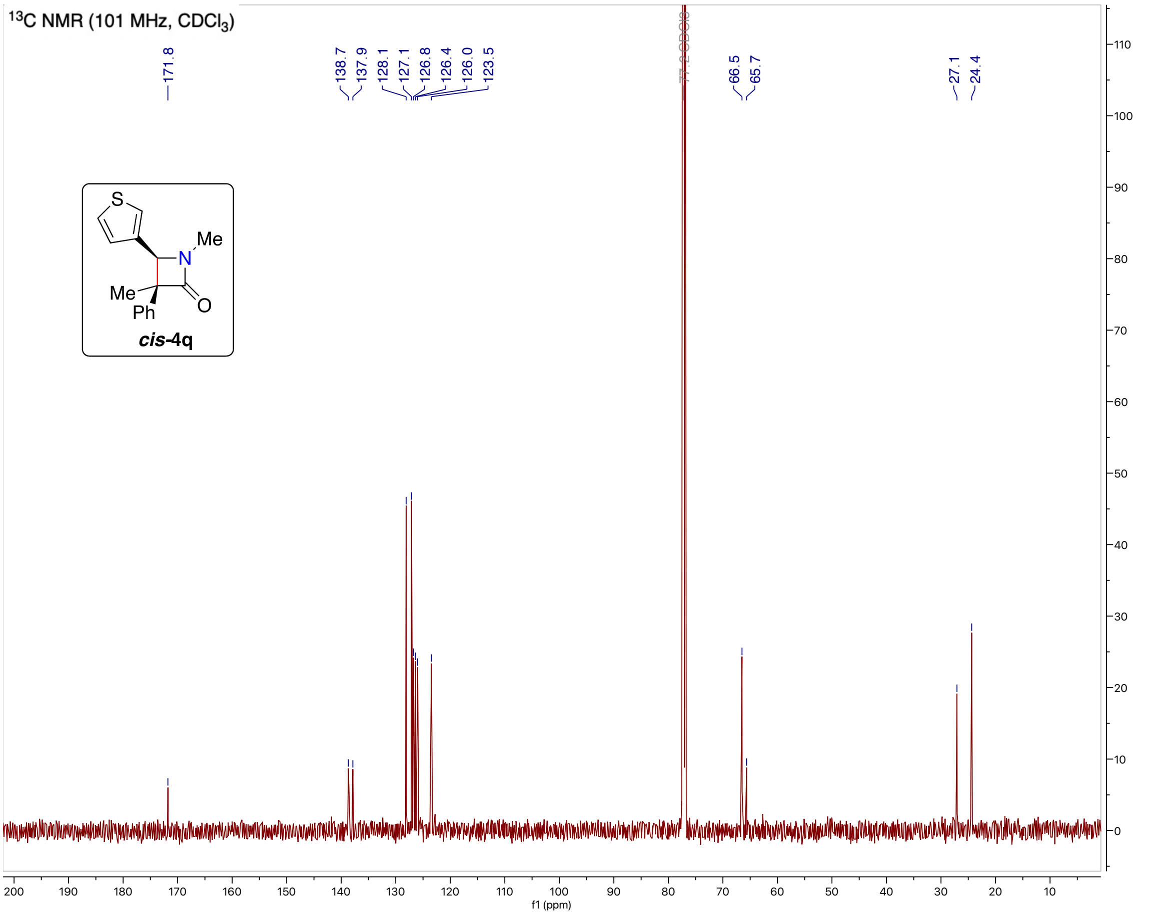

^{13}C NMR (101 MHz, CDCl$_3$)
171.8
138.7
137.9
128.1
127.1
126.8
126.4
126.0
123.5
66.5
65.7
27.1
24.4
S
N
Me
Me
Ph
O
cis-4q
f1 (ppm)

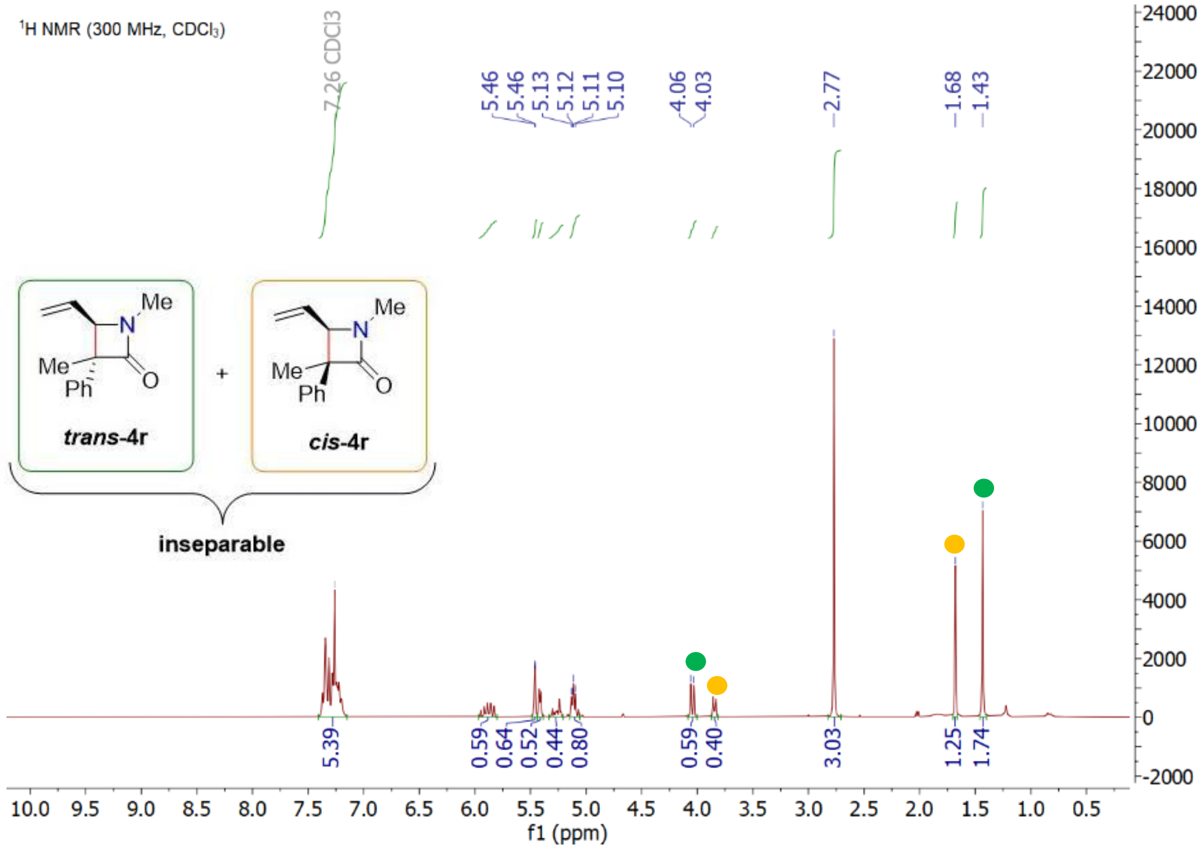

1H NMR (300 MHz, CDCl3)
trans-4r
cis-4r
inseparable
f1 (ppm)

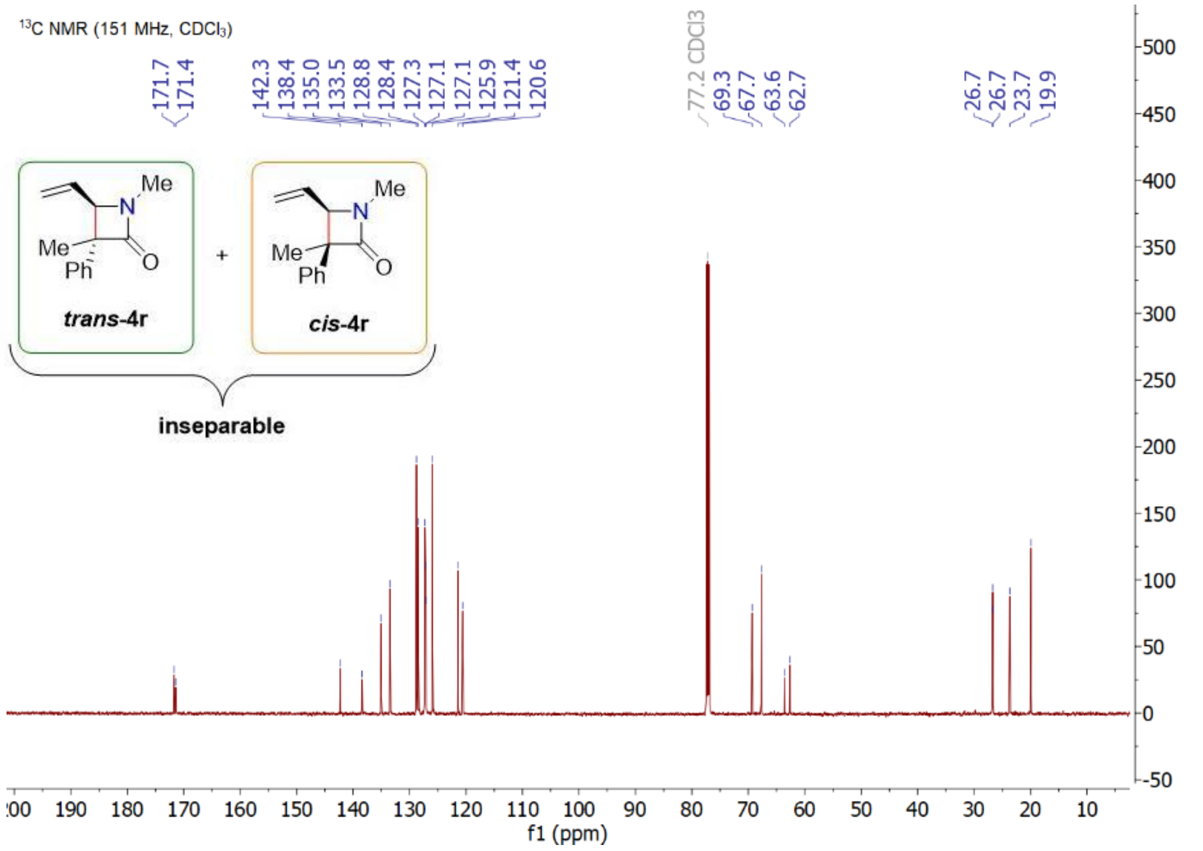

13C NMR (151 MHz, CDCl3)
trans-4r
cis-4r
inseparable
f1 (ppm)

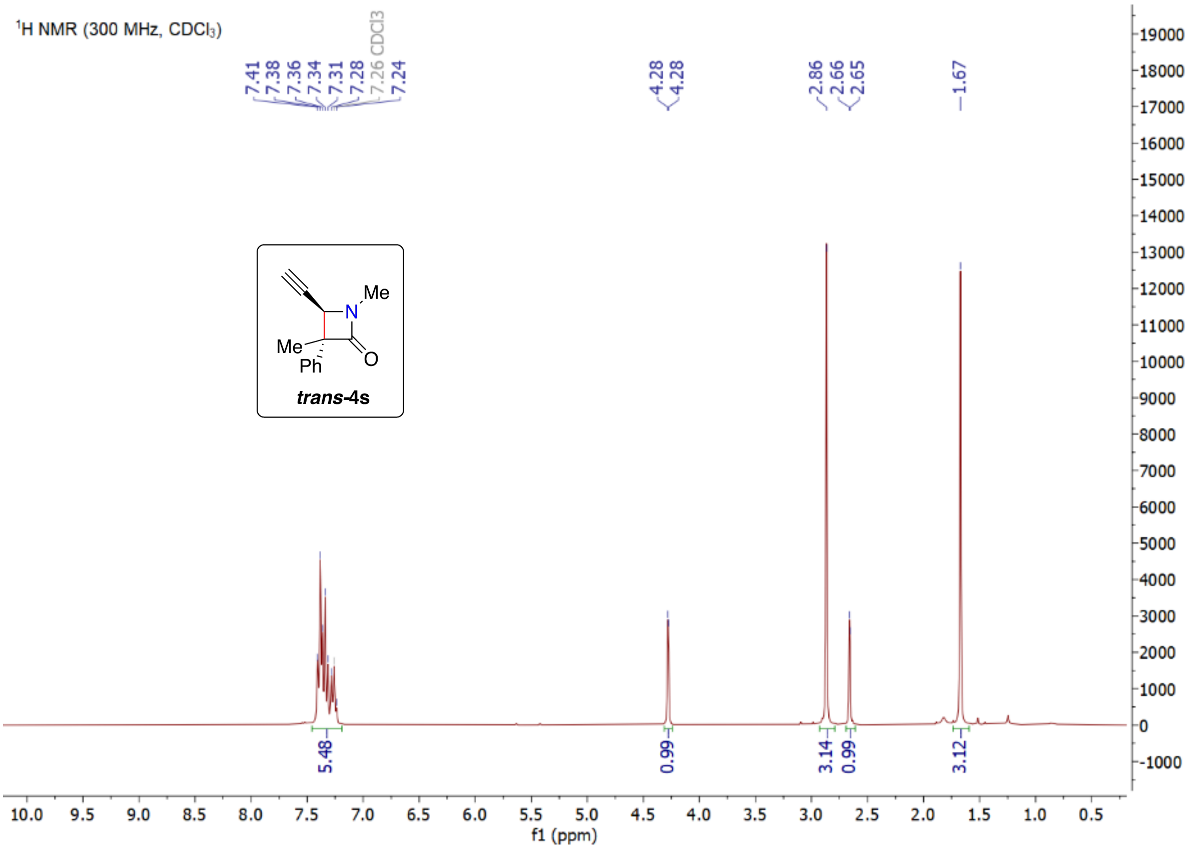

1H NMR (300 MHz, CDCl3)
7.41 7.38 7.36 7.34 7.31 7.28 7.26 CDCl3 7.24
4.28 4.28
2.86 2.66 2.65
1.67
Me
N
Me
Ph
O
trans-4s
5.48
0.99
3.14
0.99
3.12
f1 (ppm)

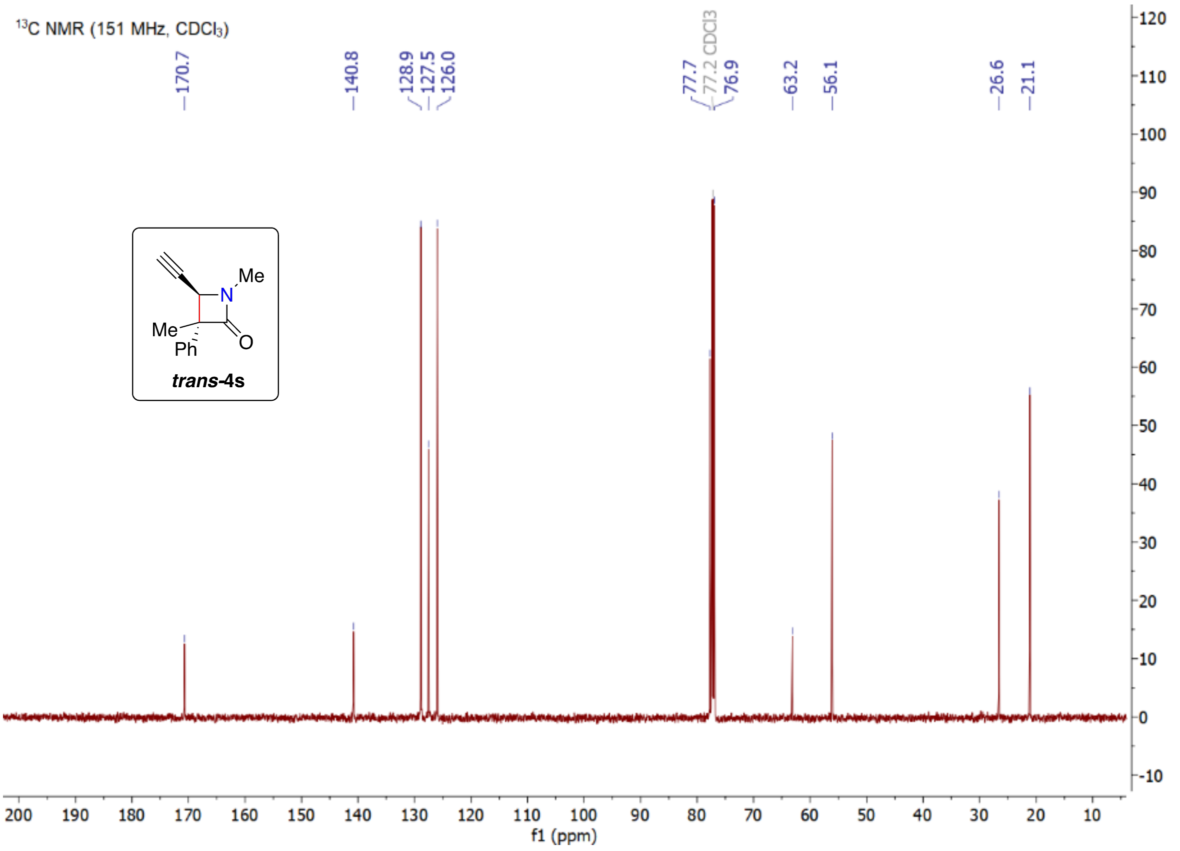

13C NMR (151 MHz, CDCl3)
170.7
140.8
128.9 127.5 126.0
77.7 77.2 CDCl3 76.9
63.2
56.1
26.6
21.1
Me
N
Me
Ph
O
trans-4s
f1 (ppm)

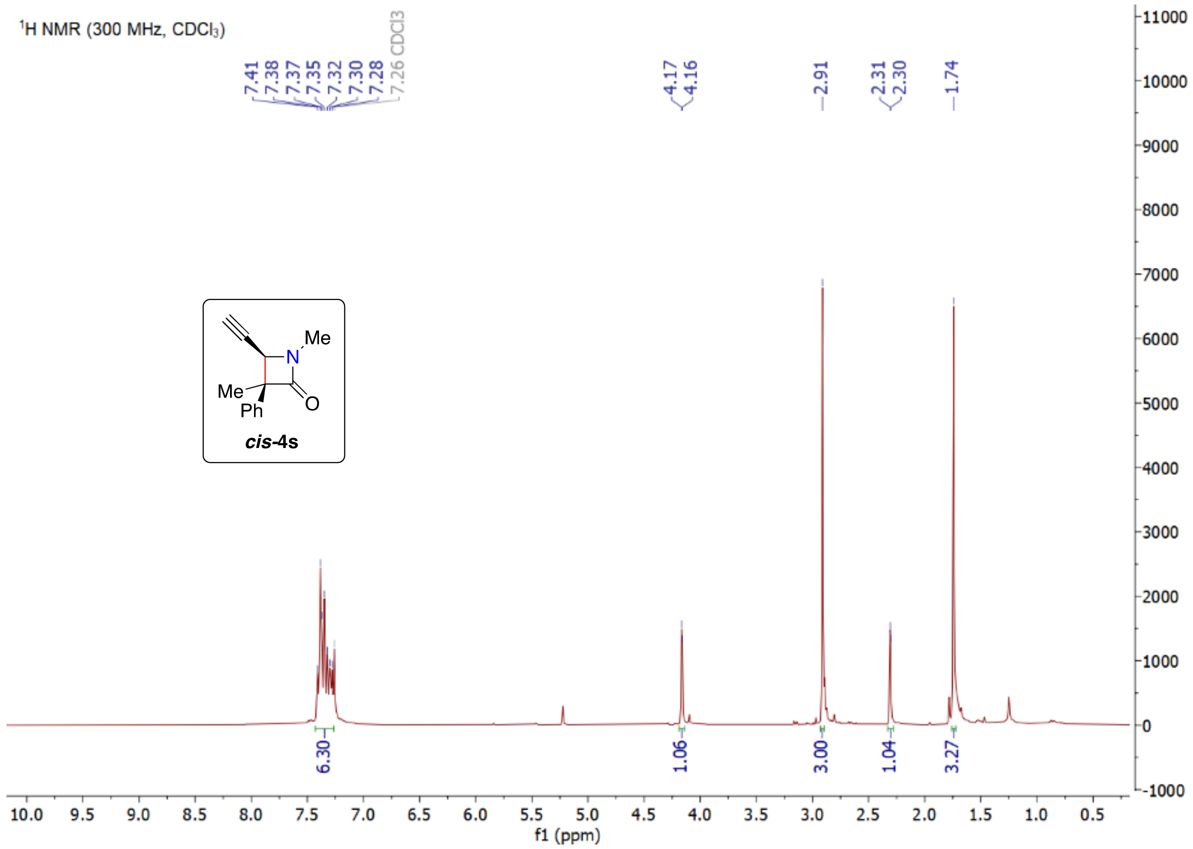

^{1}H NMR (300 MHz, CDCl$_3$)
7.41
7.38
7.37
7.35
7.32
7.30
7.28
7.26 CDCl3
4.17
4.16
2.91
2.31
2.30
1.74
6.30
1.06
3.00
1.04
3.27
Me
N
O
Me
Ph
cis-4s
f1 (ppm)

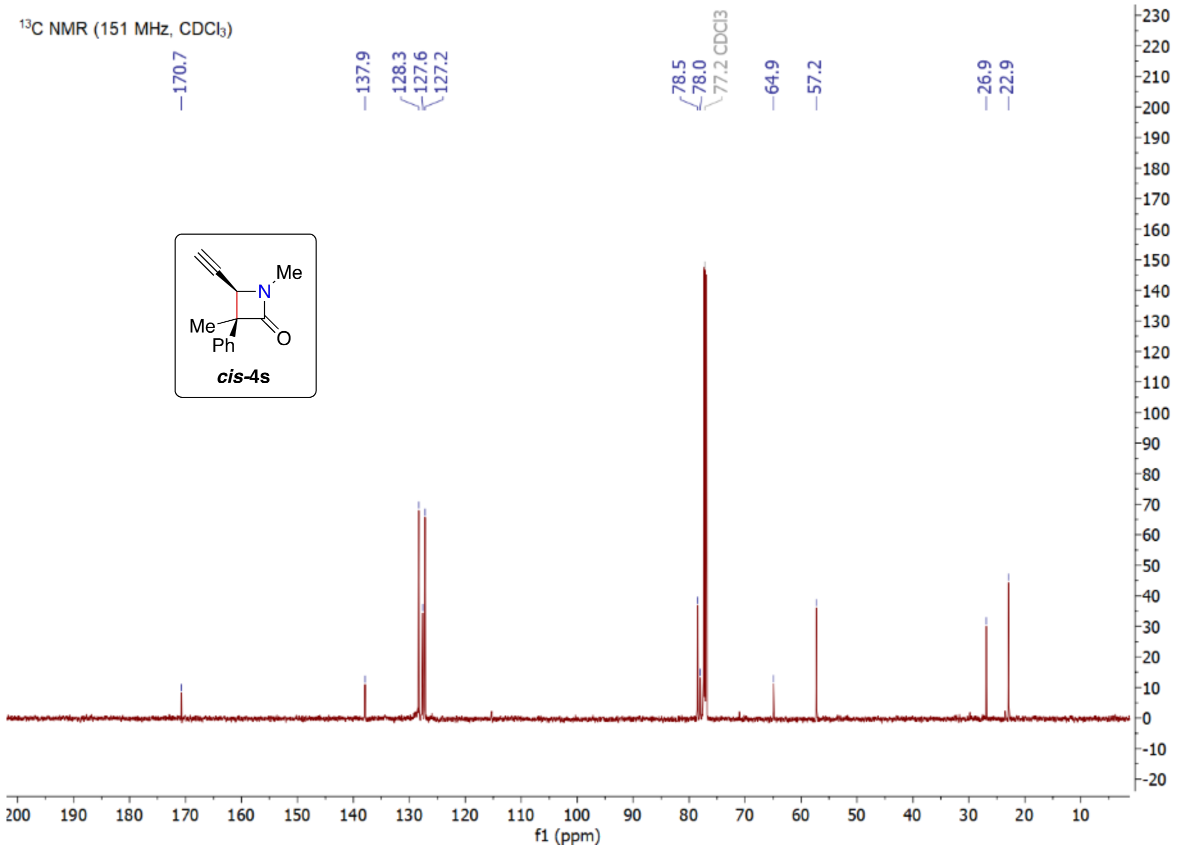

^{13}C NMR (151 MHz, CDCl$_3$)
170.7
137.9
128.3
127.6
127.2
78.5
78.0
77.2 CDCl3
64.9
57.2
26.9
22.9
Me
N
O
Me
Ph
cis-4s
f1 (ppm)

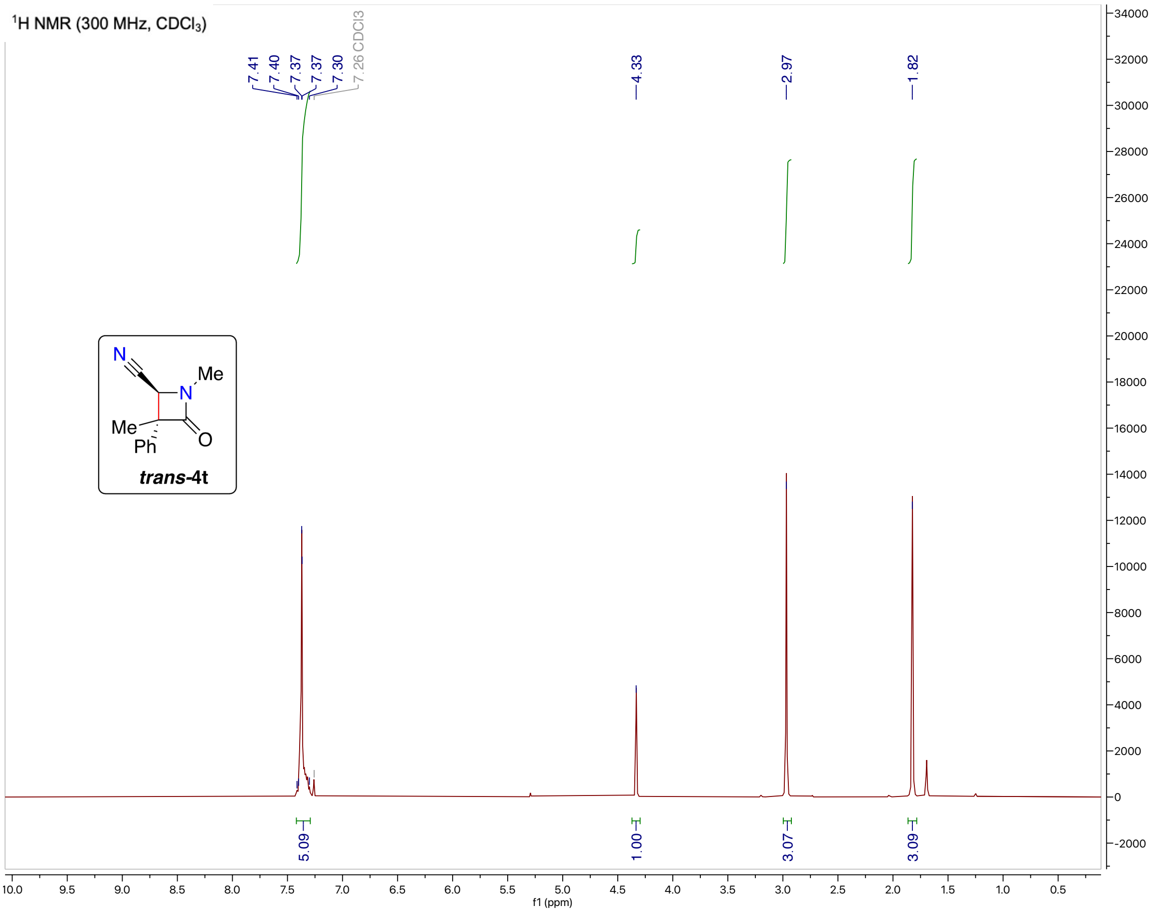

^{1}H NMR (300 MHz, CDCl$_3$)
7.41
7.40
7.37
7.37
7.30
7.26 CDCl3
4.33
2.97
1.82
trans-4t
5.09
1.00
3.07
3.09
f1 (ppm)

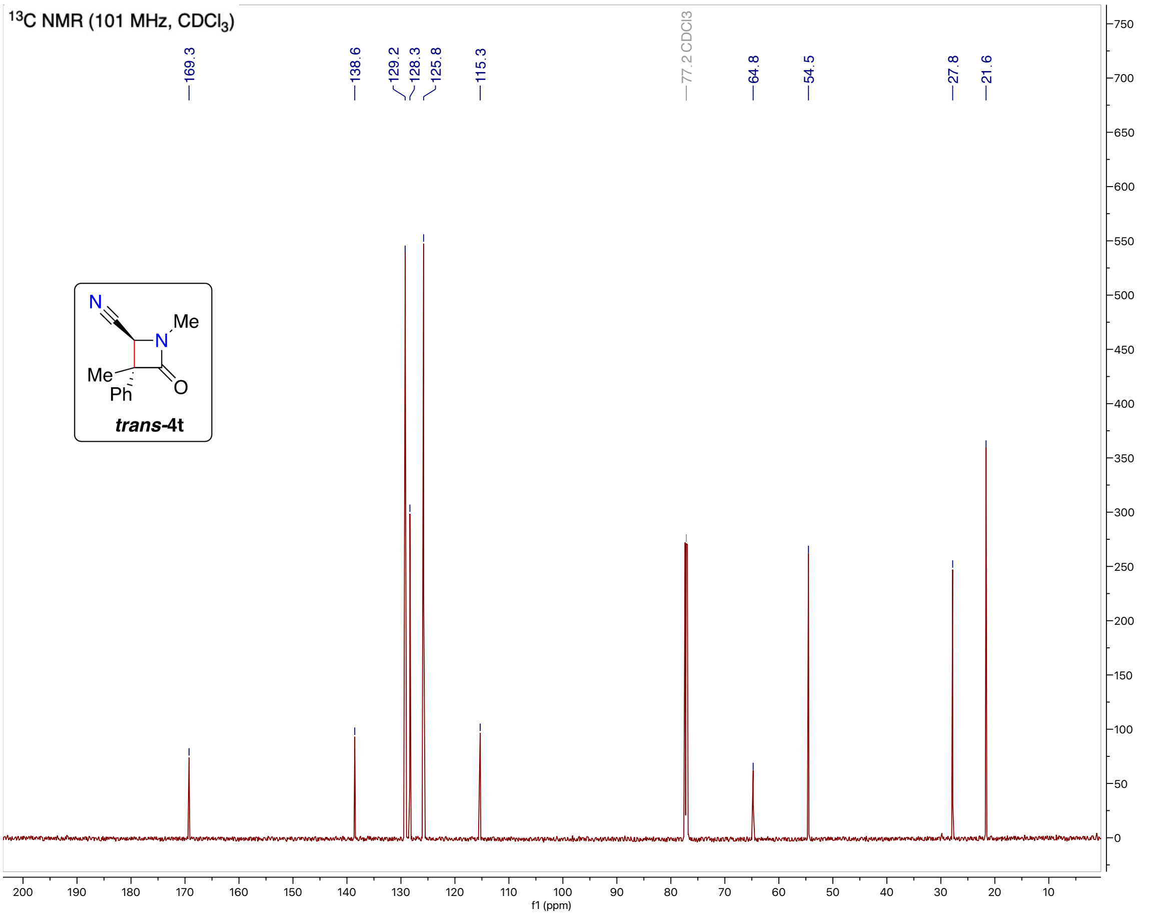

^{13}C NMR (101 MHz, CDCl$_3$)
169.3
138.6
129.2
128.3
125.8
115.3
77.2 CDCl3
64.8
54.5
27.8
21.6
trans-4t
f1 (ppm)

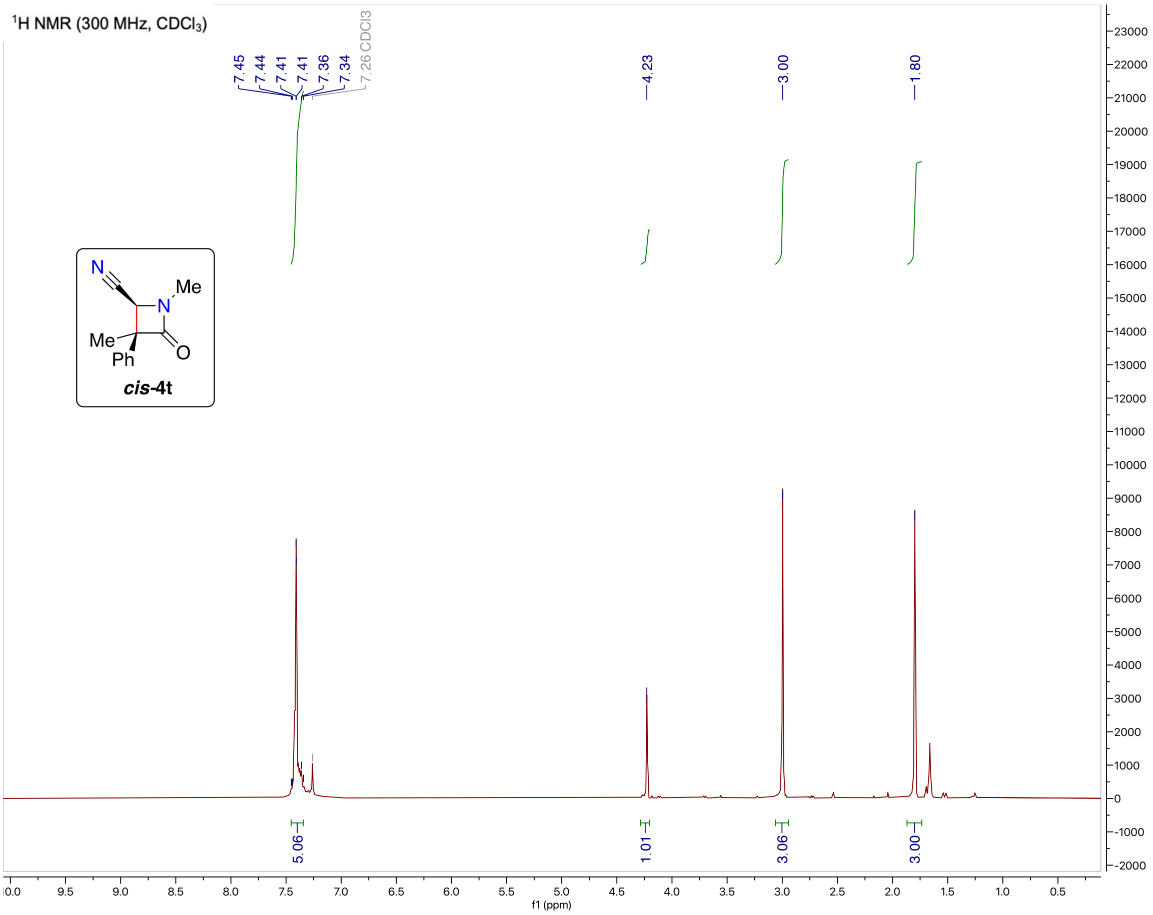

1H NMR (300 MHz, CDCl3)
7.45
7.44
7.41
7.41
7.36
7.34
7.26 CDCl3
4.23
3.00
1.80
5.06
1.01
3.06
3.00
cis-4t
f1 (ppm)

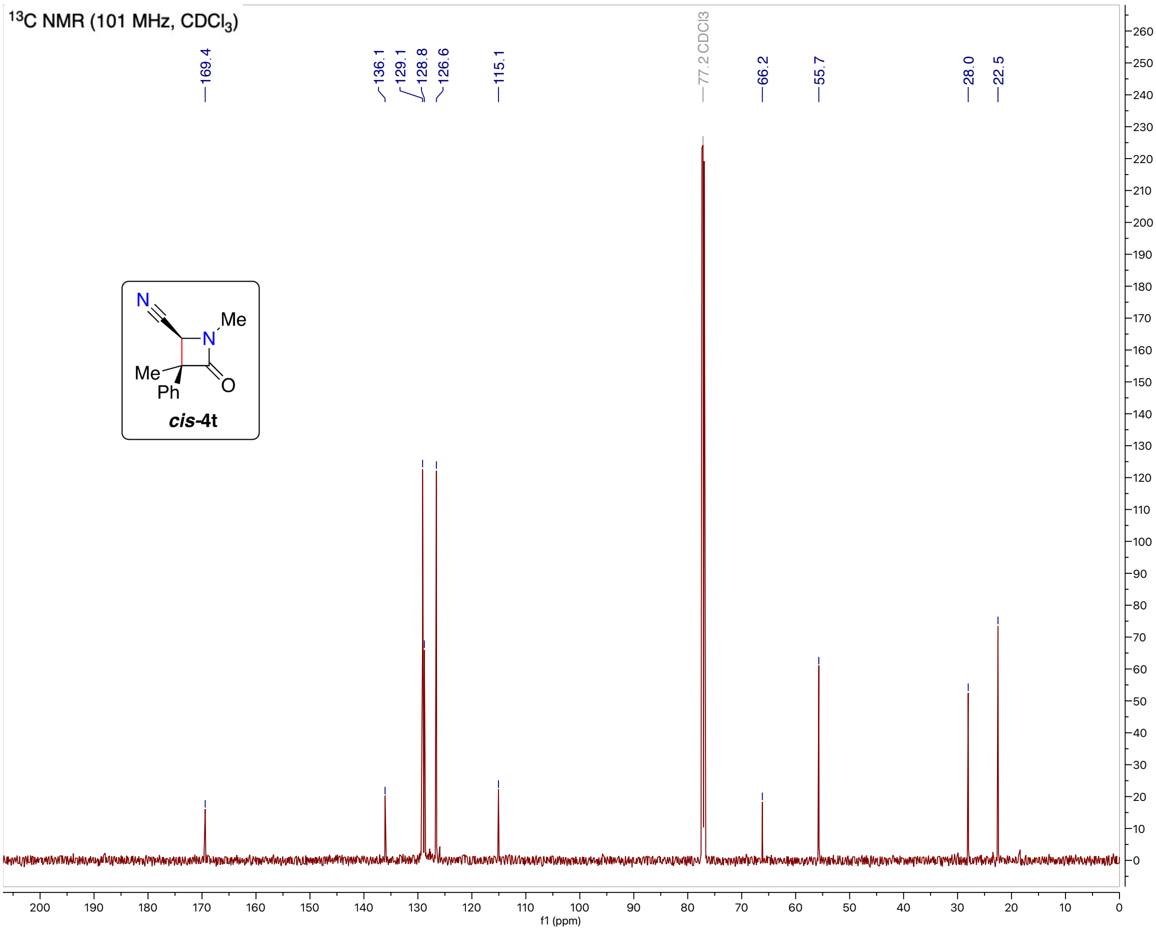

13C NMR (101 MHz, CDCl3)
169.4
136.1
129.1
128.8
126.6
115.1
77.2 CDCl3
66.2
55.7
28.0
22.5
cis-4t
f1 (ppm)

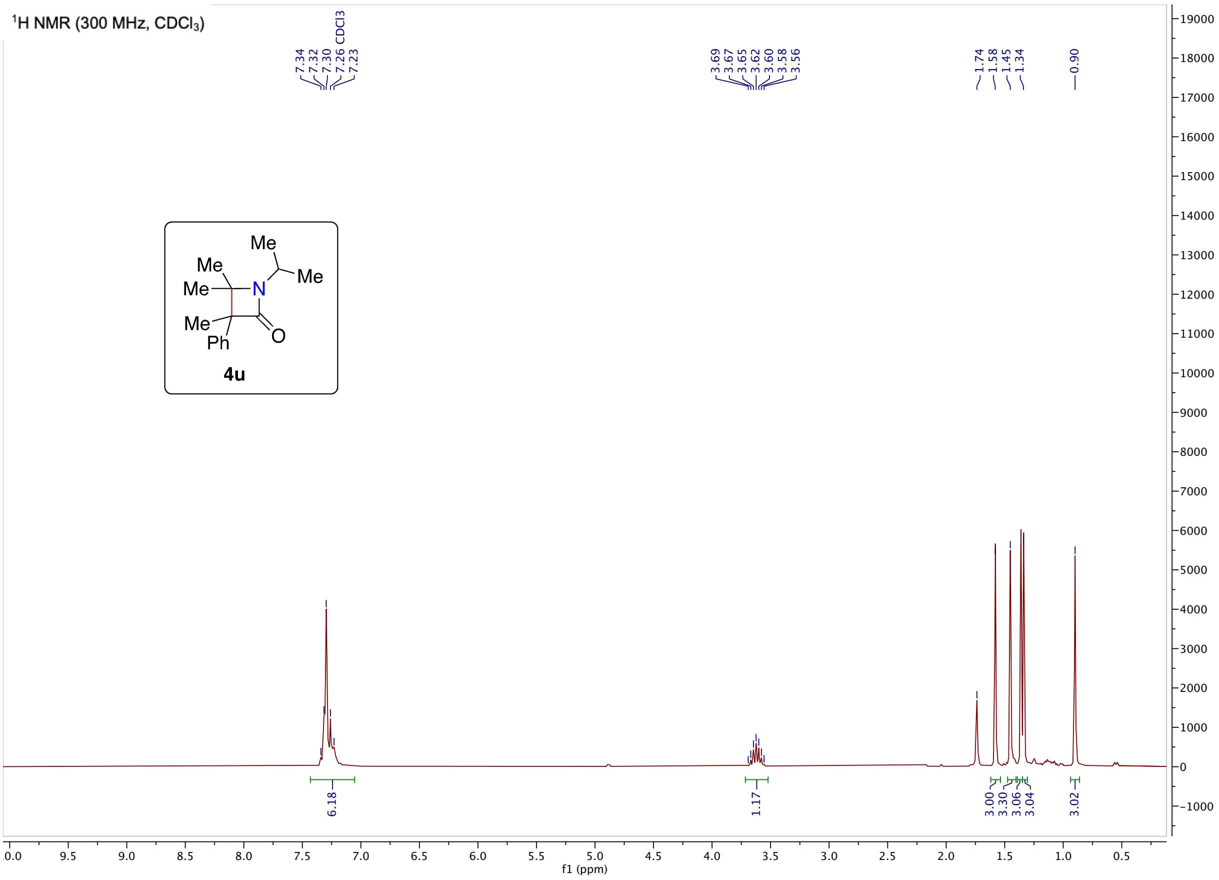
^{1}H NMR (300 MHz, $CDCl_3$)
7.34 7.32 7.30 7.26 CDCl3 7.23
3.69 3.67 3.65 3.62 3.60 3.58 3.56
1.74 1.58 1.45 1.34
0.90
6.18
1.17
3.00 3.30 3.06 3.04
3.02
Me
Me
Me
N
Me
Me
Ph
O
4u
f1 (ppm)

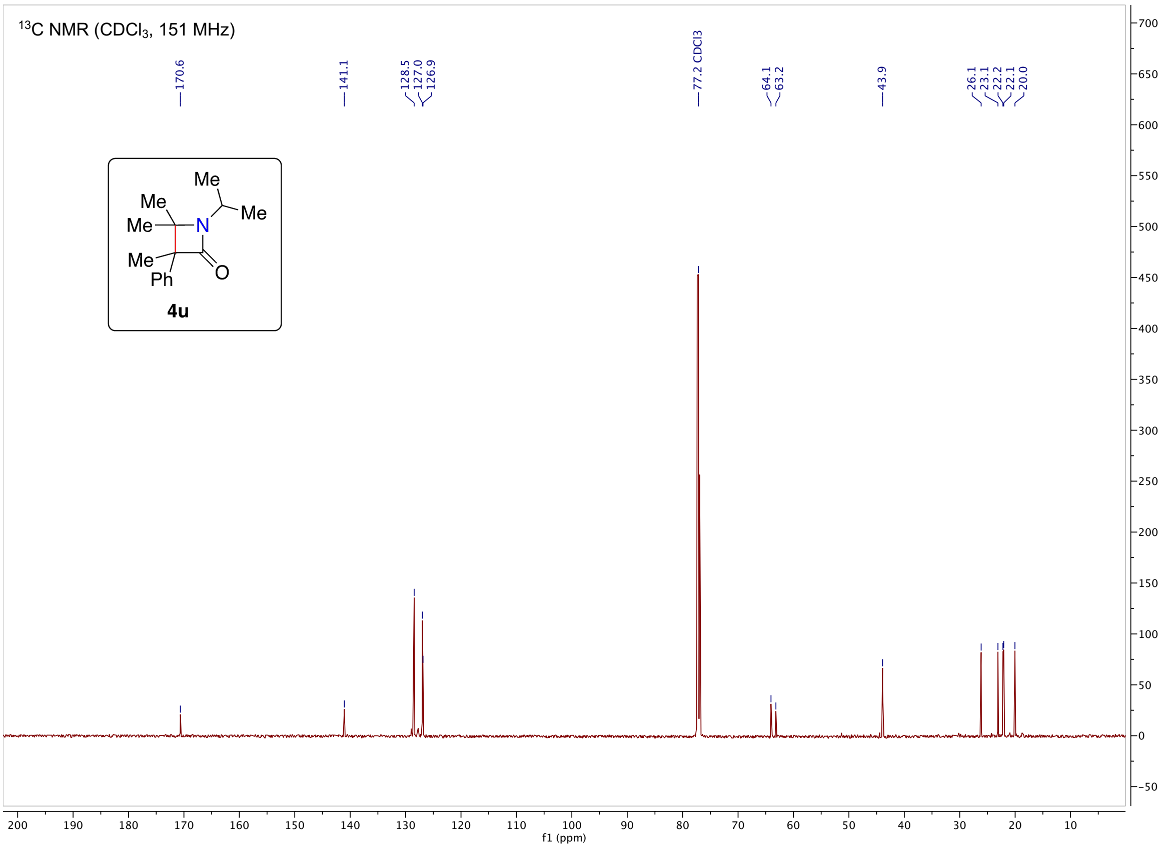
^{13}C NMR ($CDCl_3$, 151 MHz)
170.6
141.1
128.5 127.0 126.9
77.2 CDCl3
64.1 63.2
43.9
26.1 23.1 22.2 22.1 20.0
Me
Me
Me
N
Me
Me
Ph
O
4u
f1 (ppm)

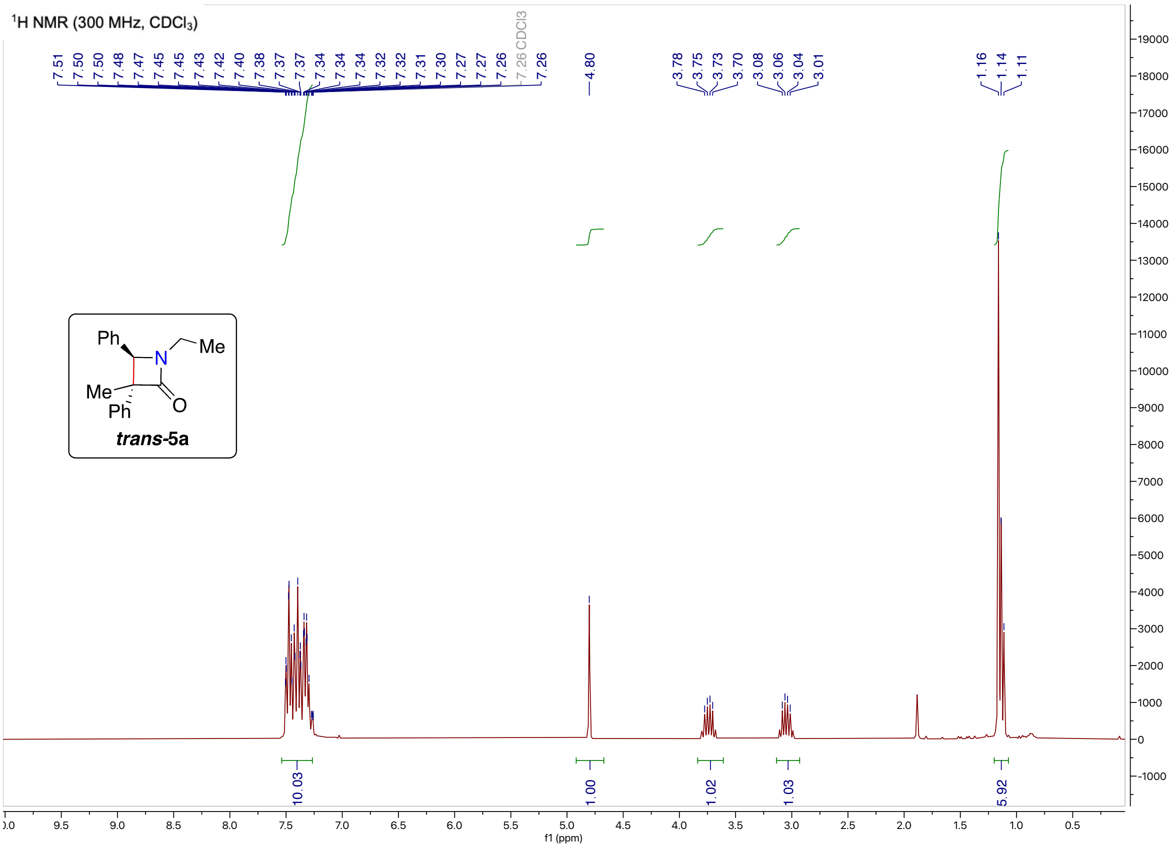
^{1}H NMR (300 MHz, CDCl$_3$)
trans-5a
10.03
1.00
1.02
1.03
5.92
f1 (ppm)

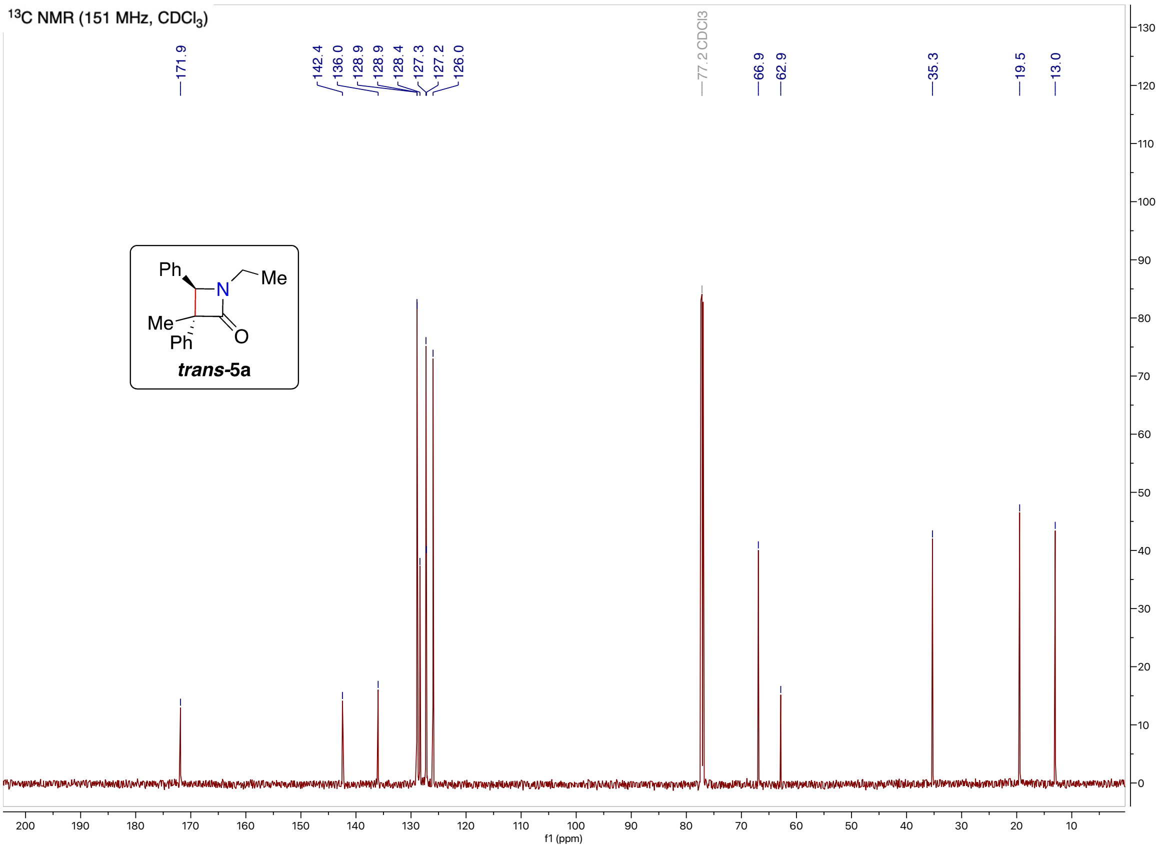
^{13}C NMR (151 MHz, CDCl$_3$)
171.9
142.4
136.0
128.9
128.9
128.4
127.3
127.2
126.0
77.2 CDCl3
66.9
62.9
35.3
19.5
13.0
trans-5a
f1 (ppm)

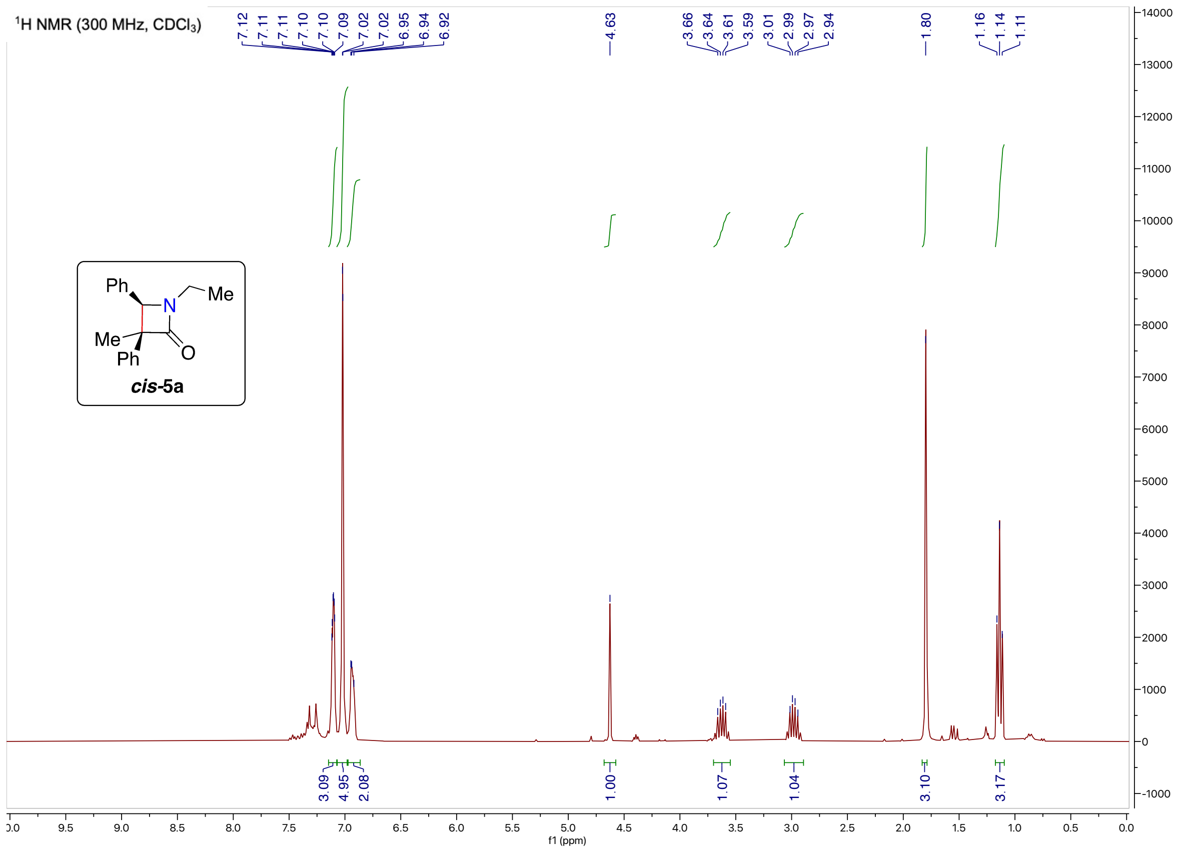

^{1}H NMR (300 MHz, CDCl$_3$)
7.12 7.11 7.11 7.10 7.10 7.09 7.02 7.02 6.95 6.94 6.92
4.63
3.66 3.64 3.61 3.59 3.01 2.99 2.97 2.94
1.80
1.16 1.14 1.11
3.09 4.95 2.08 1.00 1.07 1.04 3.10 3.17
Ph
N
Me
Me
Ph
O
cis-5a
f1 (ppm)

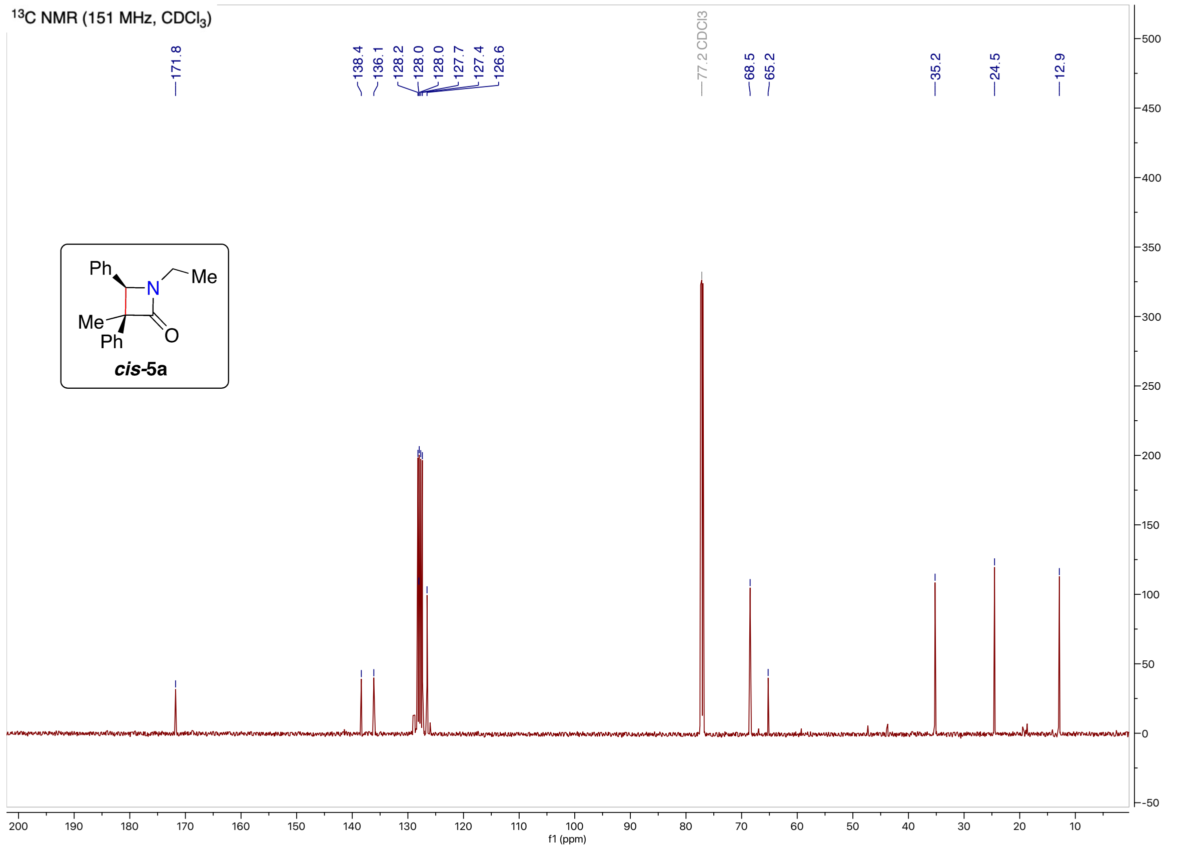

^{13}C NMR (151 MHz, CDCl$_3$)
171.8
138.4 136.1 128.2 128.0 128.0 127.7 127.4 126.6
77.2 CDCl3
68.5 65.2
35.2
24.5
12.9
Ph
N
Me
Me
Ph
O
cis-5a
f1 (ppm)

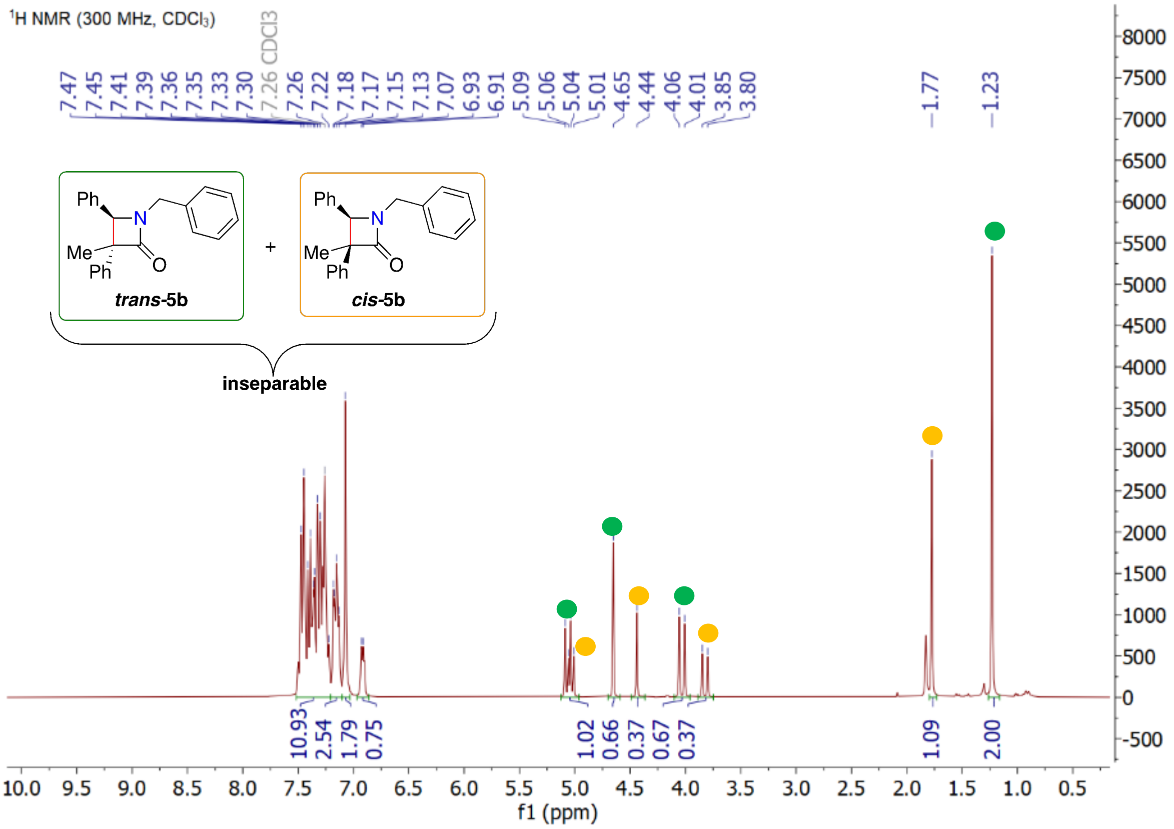

1H NMR (300 MHz, CDCl3)
7.47
7.45
7.41
7.39
7.36
7.35
7.33
7.30
7.26 CDCl3
7.26
7.22
7.18
7.17
7.15
7.13
7.07
6.93
6.91
5.09
5.06
5.04
5.01
4.65
4.44
4.06
4.01
3.85
3.80
1.77
1.23
Ph
N
Me
Ph
O
trans-5b
+
cis-5b
inseparable
10.93
2.54
1.79
0.75
1.02
0.66
0.37
0.67
0.37
1.09
2.00
f1 (ppm)

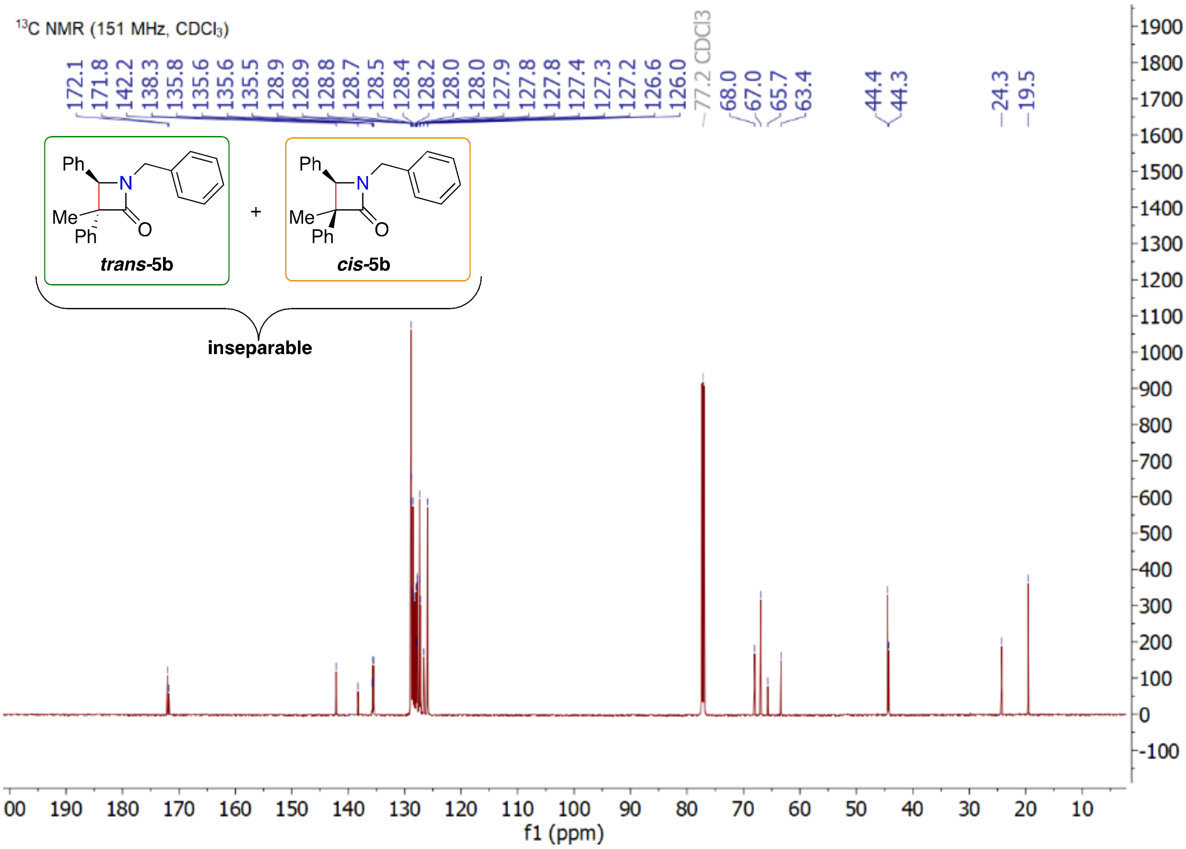

13C NMR (151 MHz, CDCl3)
172.1
171.8
142.2
138.3
135.8
135.6
135.6
135.5
128.9
128.9
128.8
128.7
128.5
128.4
128.2
128.0
128.0
127.9
127.8
127.8
127.4
127.3
127.2
126.6
126.0
77.2 CDCl3
68.0
67.0
65.7
63.4
44.4
44.3
24.3
19.5
Ph
N
Me
Ph
O
trans-5b
+
cis-5b
inseparable
f1 (ppm)

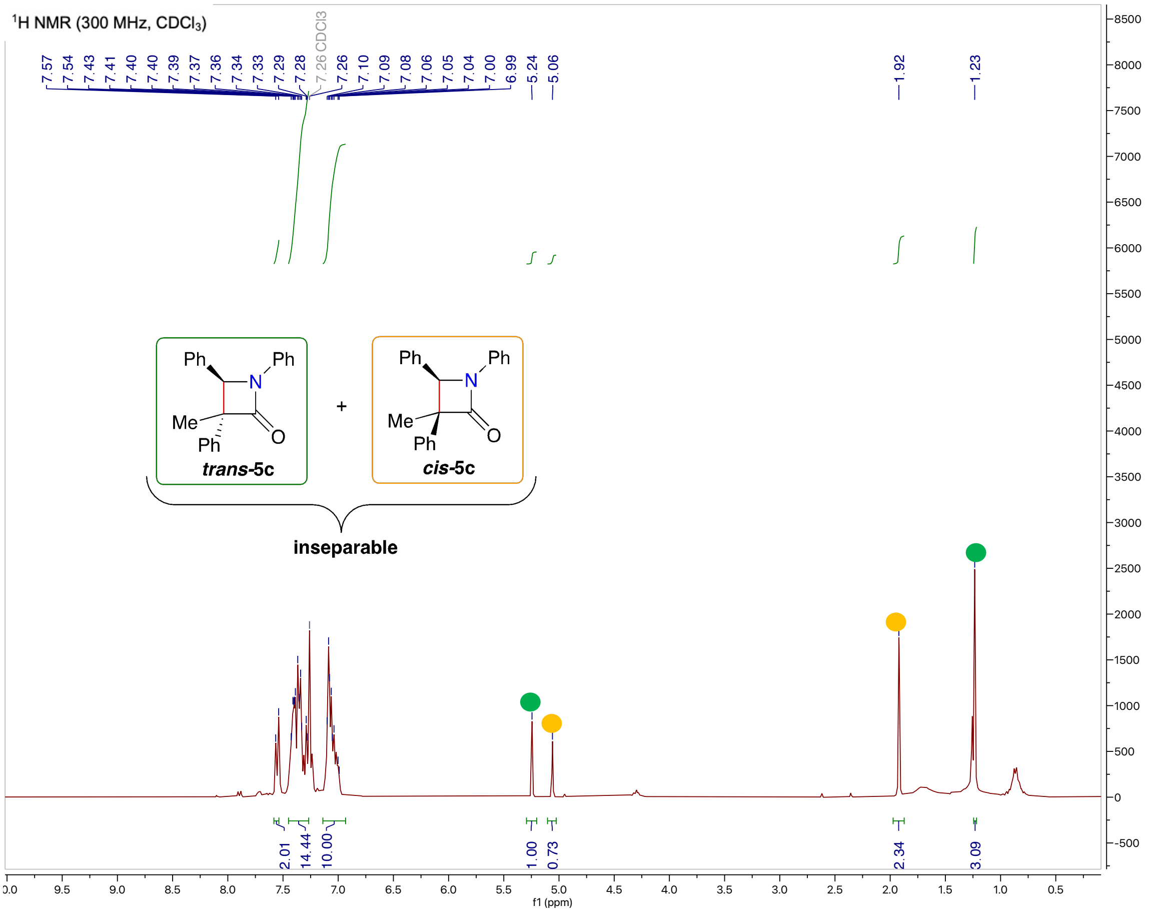

^{1}H NMR (300 MHz, CDCl$_3$)
7.57 7.54 7.43 7.41 7.40 7.40 7.39 7.37 7.36 7.34 7.33 7.29 7.28 7.26 CDCl3 7.26 7.10 7.09 7.08 7.06 7.05 7.04 7.00 6.99 5.24 5.06 1.92 1.23
Ph Ph N Me Ph O trans-5c
+
Ph Ph N Me Ph O cis-5c
inseparable
2.01 14.44 10.00 1.00 0.73 2.34 3.09
f1 (ppm)

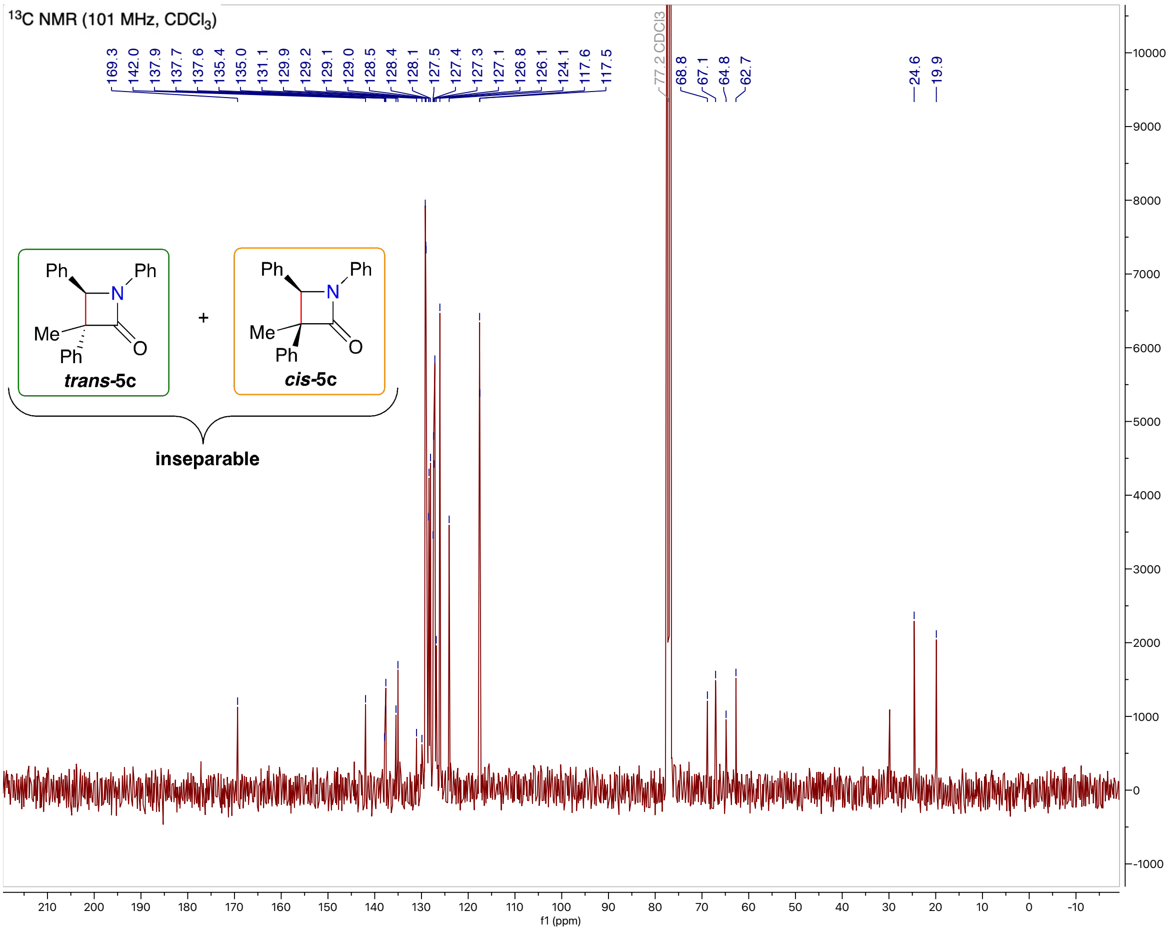

^{13}C NMR (101 MHz, CDCl$_3$)
169.3 142.0 137.9 137.7 137.6 135.4 135.0 131.1 129.9 129.2 129.1 129.0 128.5 128.4 128.1 127.5 127.4 127.3 127.1 126.8 126.1 124.1 117.6 117.5 77.2 CDCl3 68.8 67.1 64.8 62.7 24.6 19.9
trans-5c
cis-5c
inseparable
f1 (ppm)

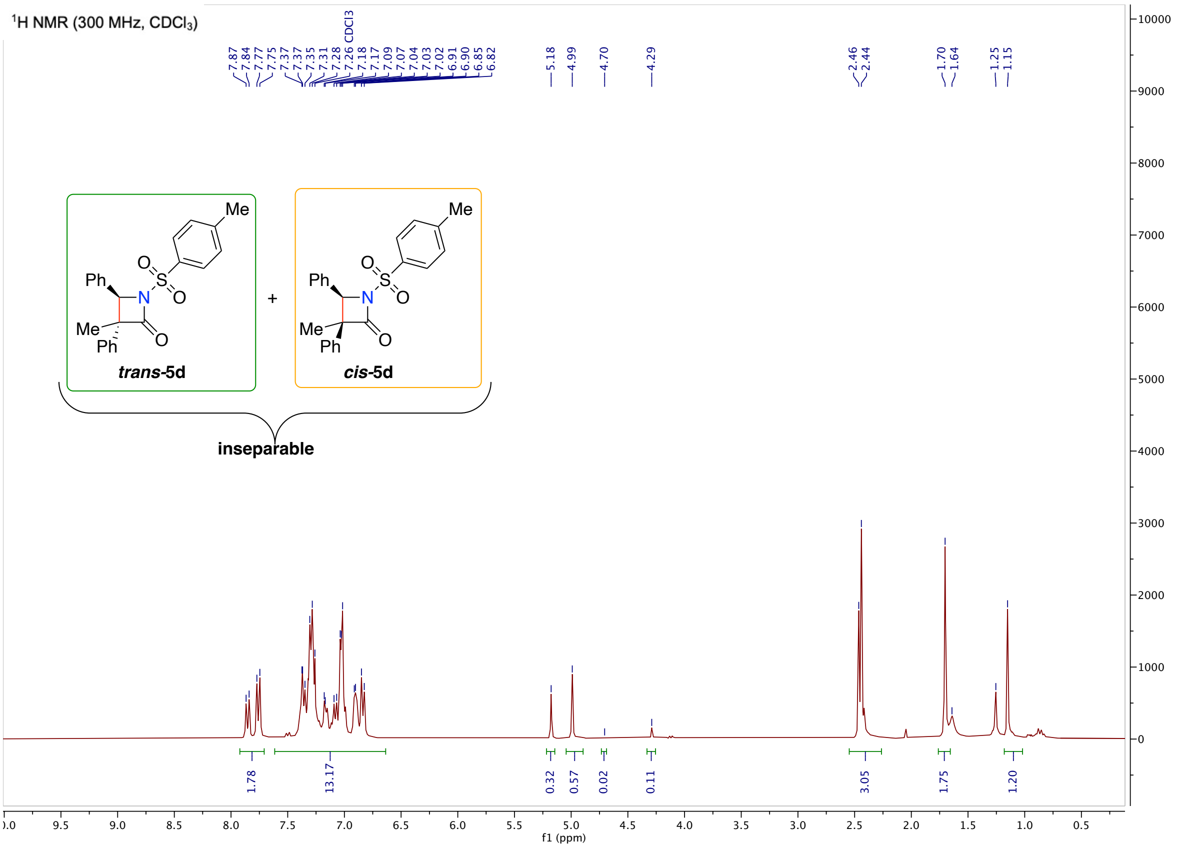
1H NMR (300 MHz, CDCl3)
7.87 7.84 7.77 7.75 7.37 7.37 7.35 7.31 7.28 7.26 CDCl3 7.18 7.17 7.09 7.07 7.04 7.03 7.02 6.91 6.90 6.85 6.82
5.18 4.99 4.70 4.29
2.46 2.44
1.70 1.64
1.25 1.15
1.78 13.17 0.32 0.57 0.02 0.11 3.05 1.75 1.20
trans-5d
cis-5d
inseparable
f1 (ppm)

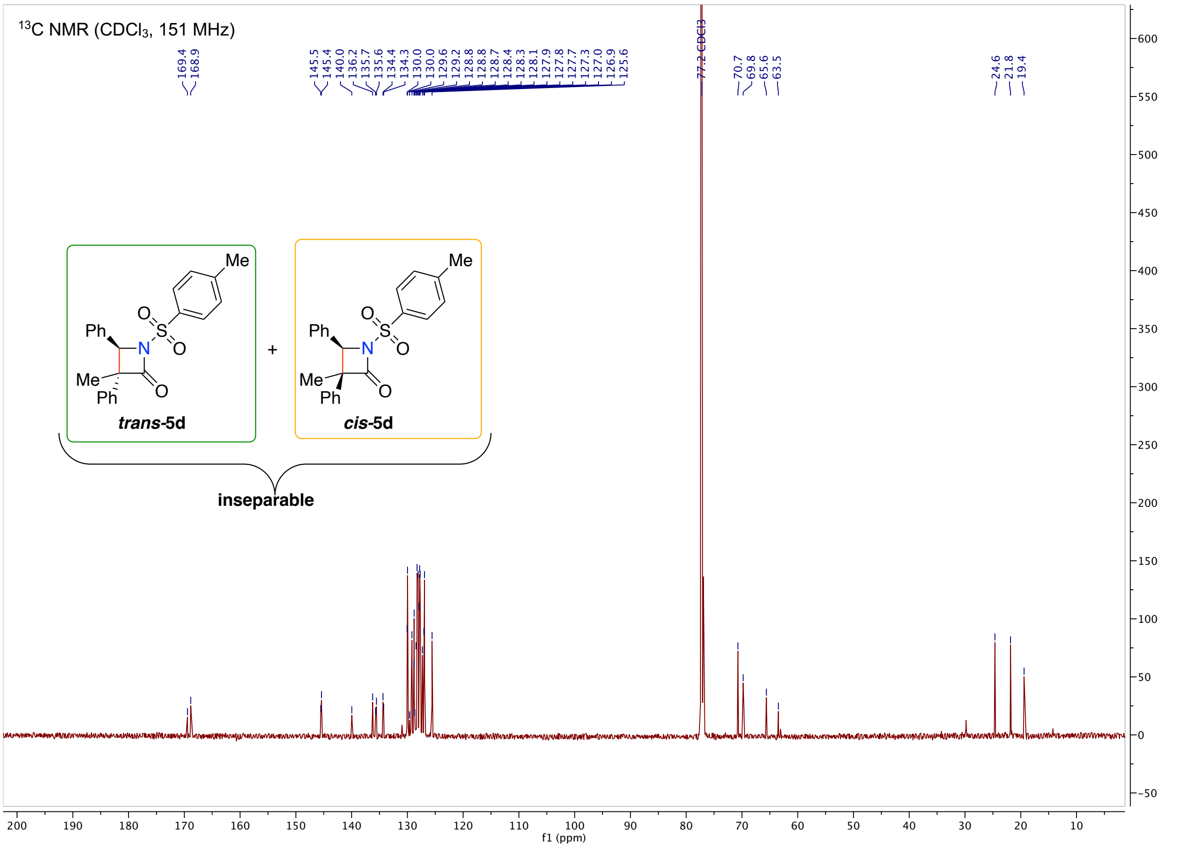
13C NMR (CDCl3, 151 MHz)
169.4 168.9
145.5 145.4 140.0 136.2 135.7 135.6 134.4 134.3 130.0 130.0 129.6 129.2 128.8 128.8 128.7 128.4 128.3 128.1 127.9 127.8 127.7 127.3 127.0 126.9 125.6
77.2 CDCl3
70.7 69.8 65.6 63.5
24.6 21.8 19.4
trans-5d
cis-5d
inseparable
f1 (ppm)

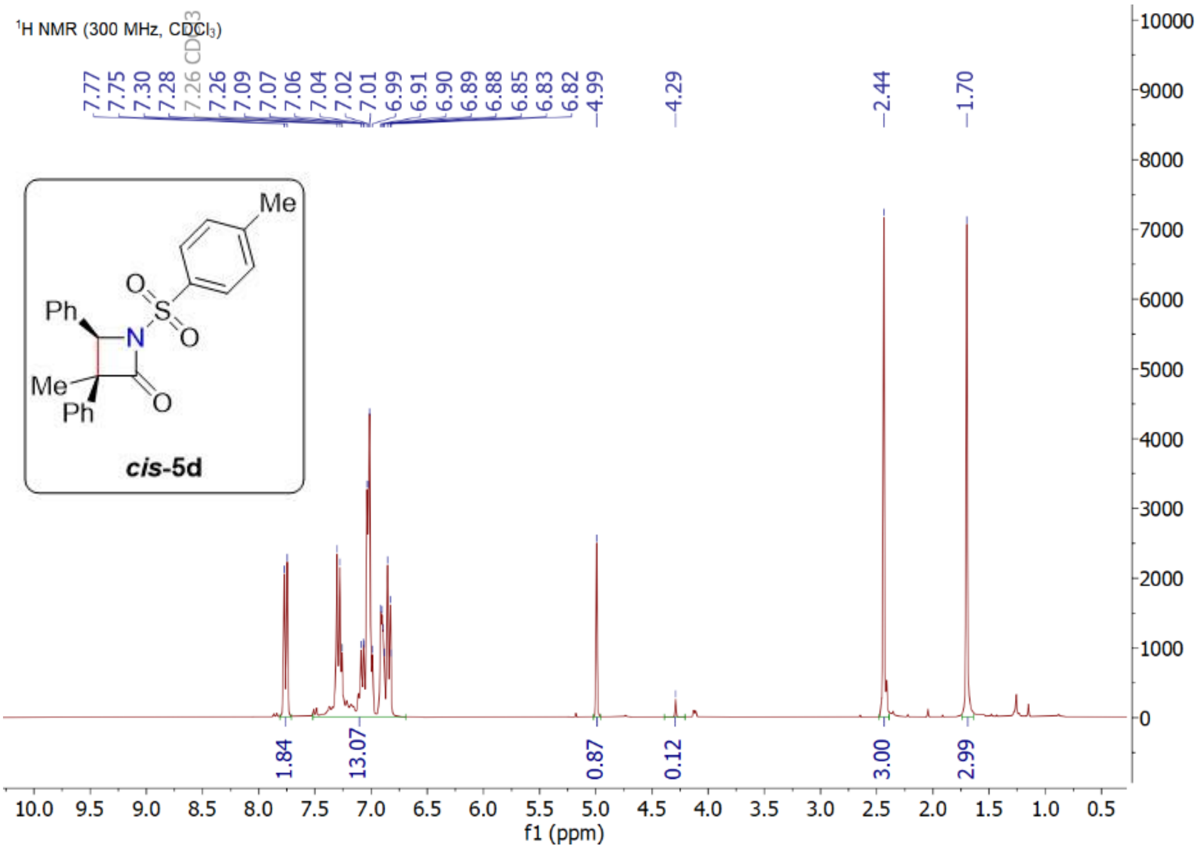

1H NMR (300 MHz, CDCl3)
7.77 7.75 7.30 7.28 7.26 CDCl3 7.26 7.09 7.07 7.06 7.04 7.02 7.01 6.99 6.91 6.90 6.89 6.88 6.85 6.83 6.82 4.99
4.29
2.44
1.70
Me
Ph
N
O
S
O
Me
Ph
O
cis-5d
1.84
13.07
0.87
0.12
3.00
2.99
f1 (ppm)

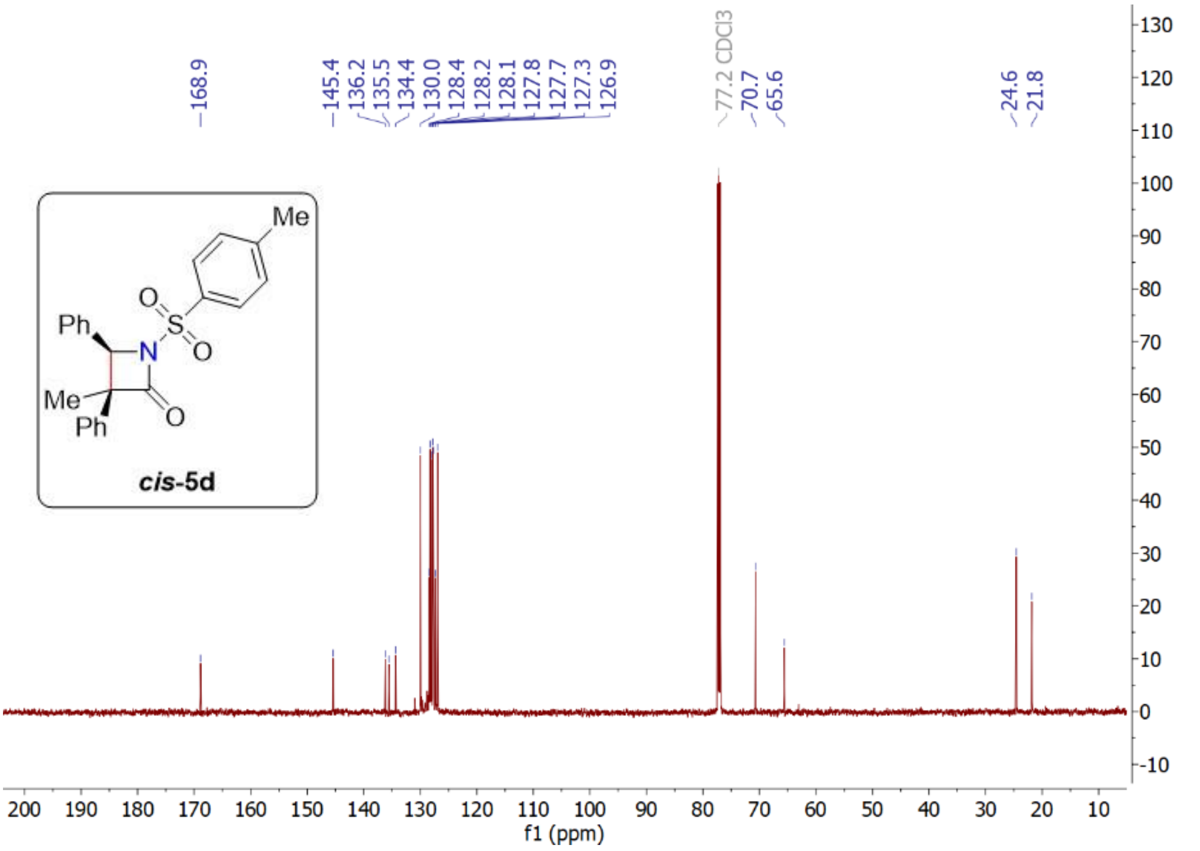

168.9
145.4 136.2 135.5 134.4 130.0 128.4 128.2 128.1 127.8 127.7 127.3 126.9
77.2 CDCl3 70.7 65.6
24.6 21.8
Me
Ph
N
O
S
O
Me
Ph
O
cis-5d
f1 (ppm)

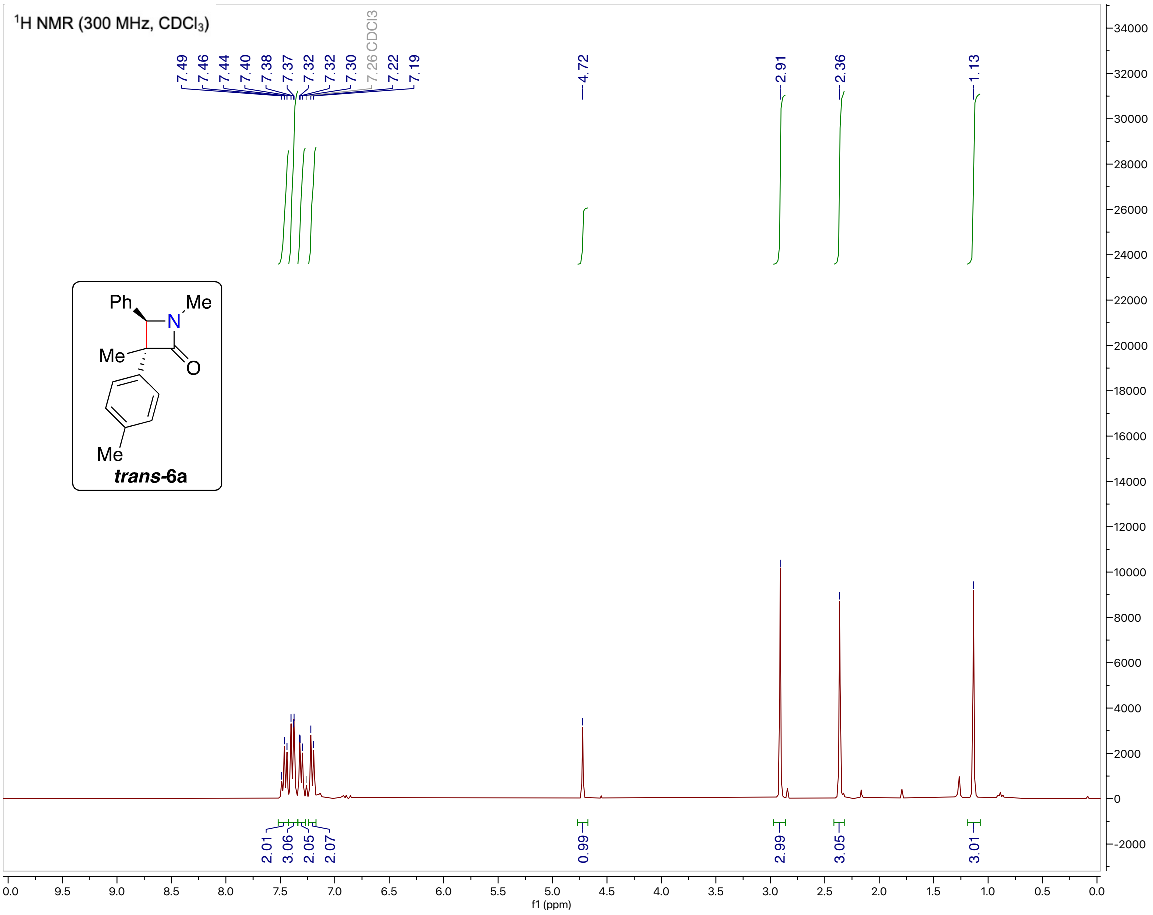

^{1}H NMR (300 MHz, CDCl$_3$)
7.49
7.46
7.44
7.40
7.38
7.37
7.32
7.32
7.30
7.26 CDCl3
7.22
7.19
4.72
2.91
2.36
1.13
Ph
N
Me
Me
O
Me
trans-6a
2.01
3.06
2.05
2.07
0.99
2.99
3.05
3.01
f1 (ppm)

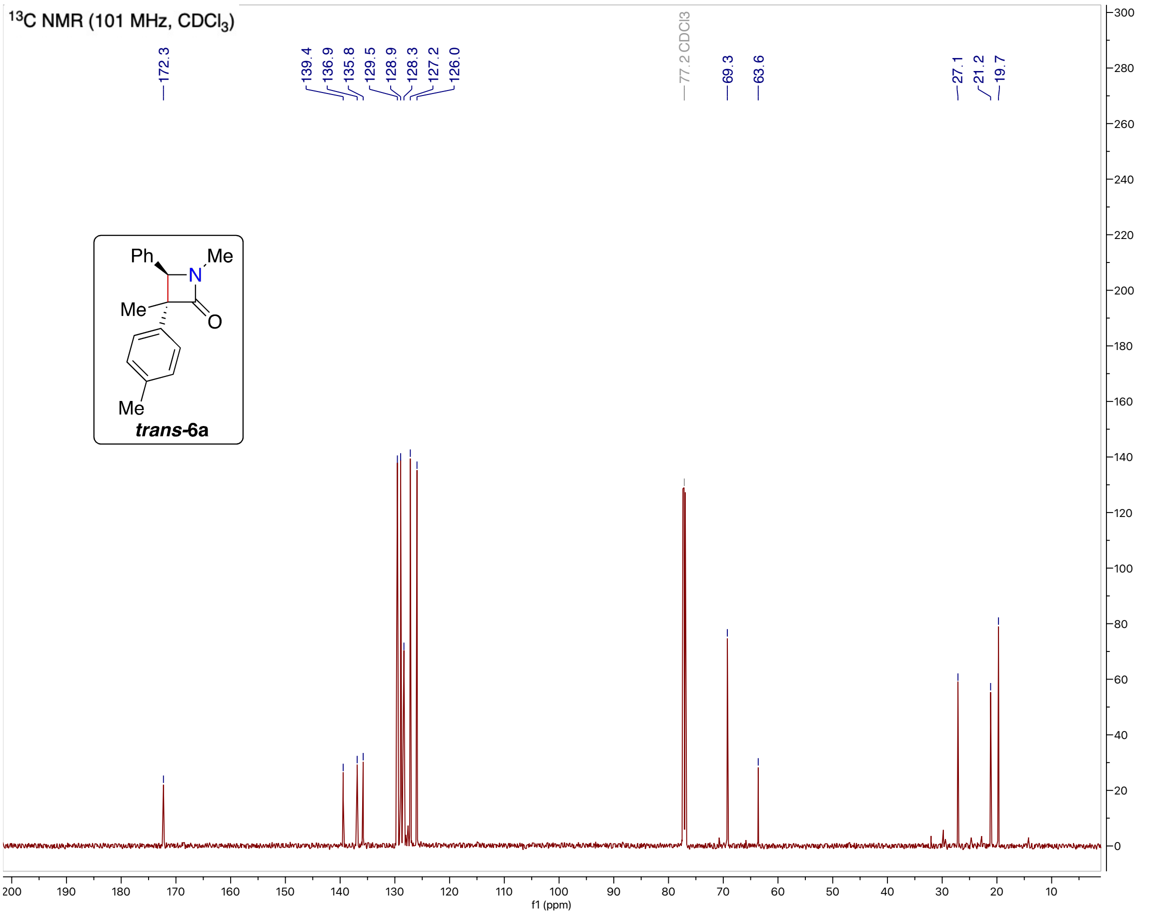

^{13}C NMR (101 MHz, CDCl$_3$)
172.3
139.4
136.9
135.8
129.5
128.9
128.3
127.2
126.0
77.2 CDCl3
69.3
63.6
27.1
21.2
19.7
Ph
N
Me
Me
O
Me
trans-6a
f1 (ppm)

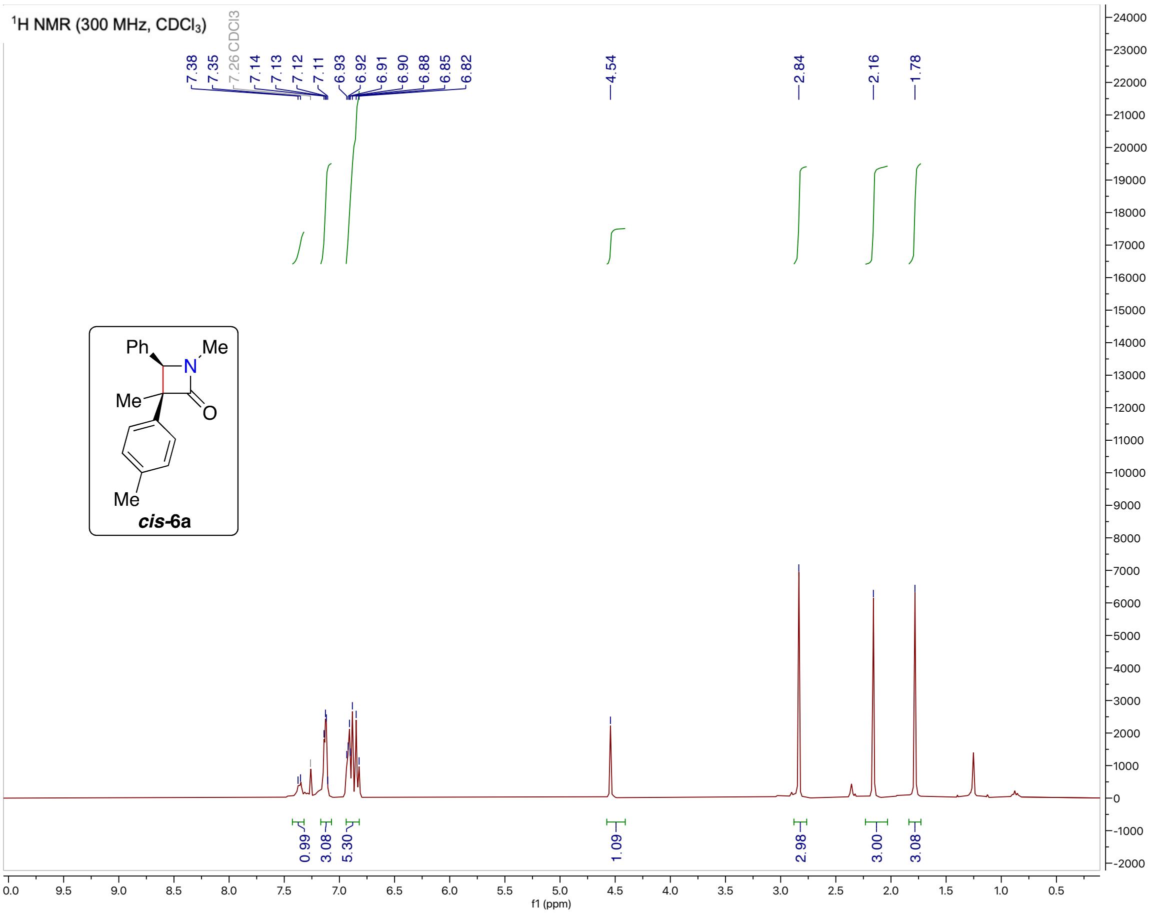

^{1}H NMR (300 MHz, CDCl$_3$)
7.38
7.35
7.26 CDCl3
7.14
7.13
7.12
7.11
6.93
6.92
6.91
6.90
6.88
6.85
6.82
4.54
2.84
2.16
1.78
Ph
Me
N
Me
O
Me
cis-6a
0.99
3.08
5.30
1.09
2.98
3.00
3.08
f1 (ppm)

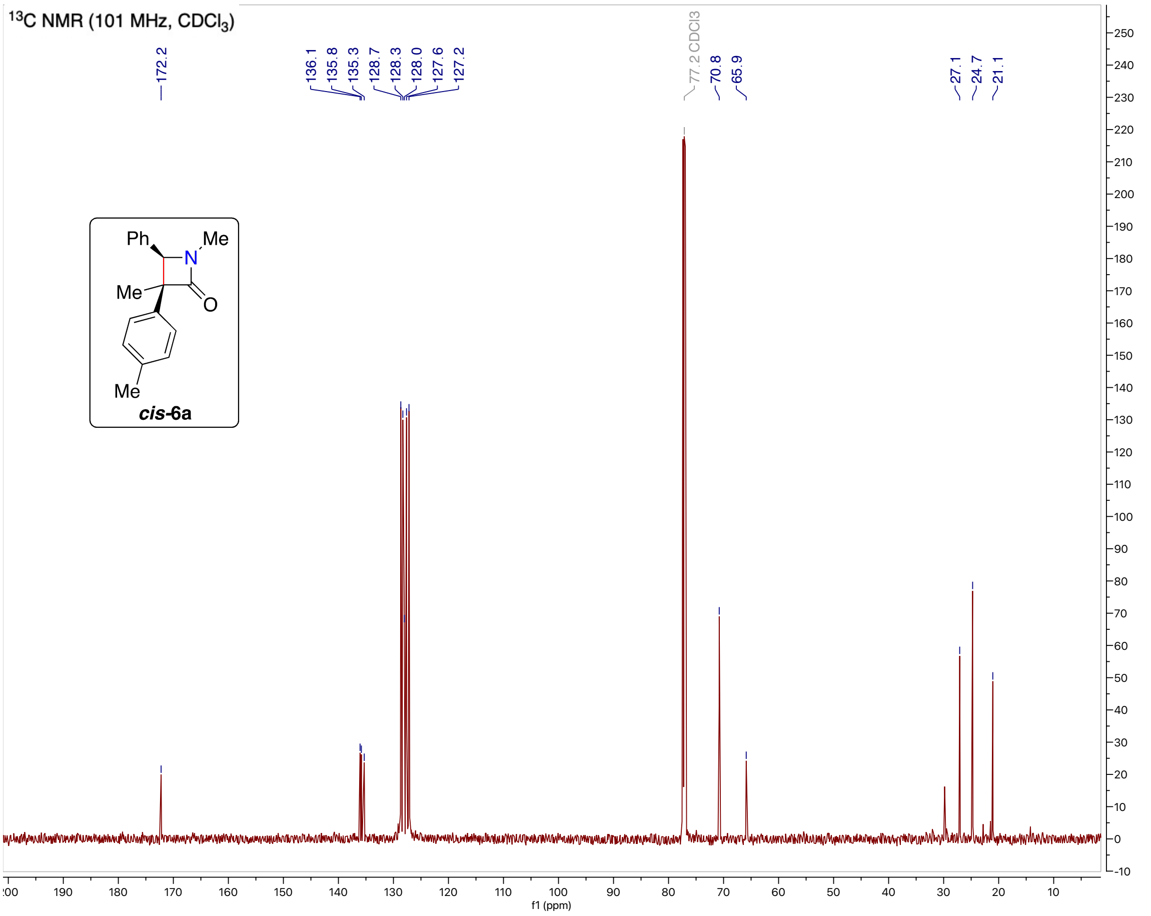

^{13}C NMR (101 MHz, CDCl$_3$)
172.2
136.1
135.8
135.3
128.7
128.3
128.0
127.6
127.2
77.2 CDCl3
70.8
65.9
27.1
24.7
21.1
Ph
Me
N
Me
O
Me
cis-6a
f1 (ppm)

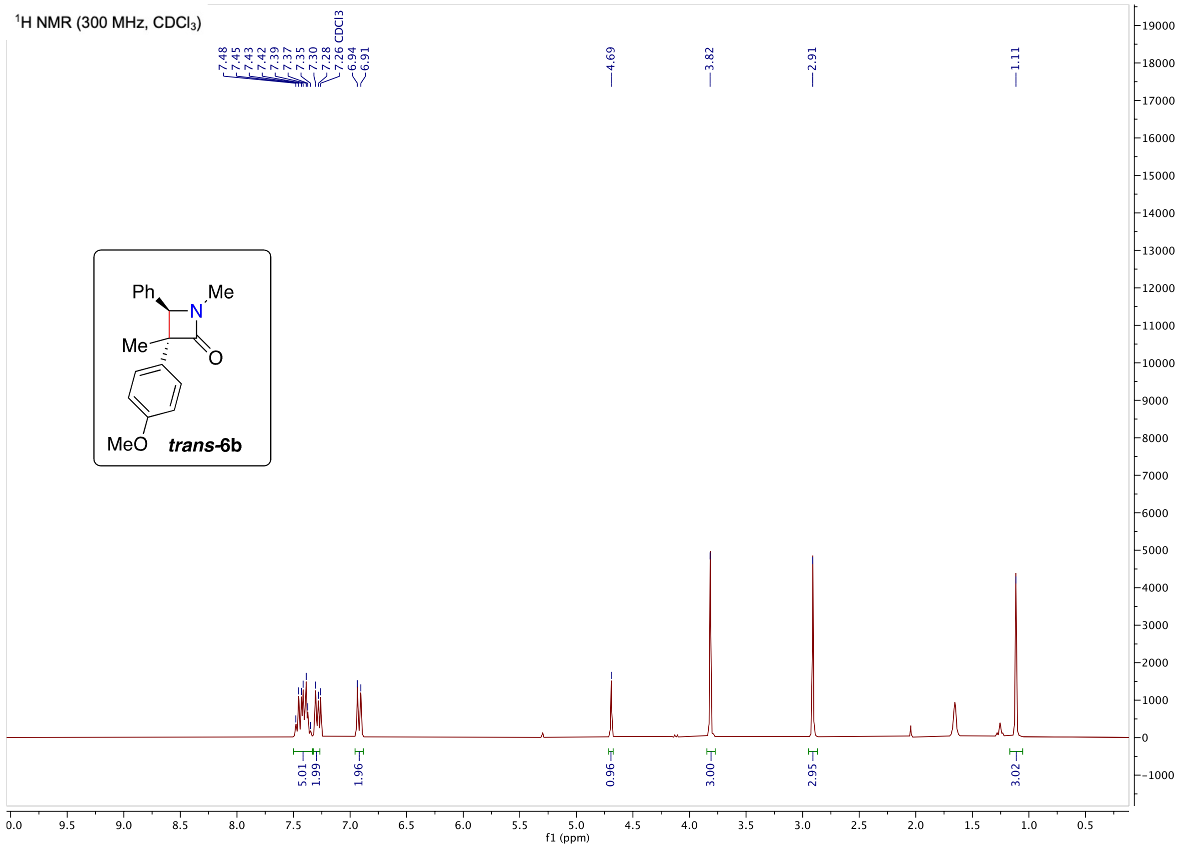

^{1}H NMR (300 MHz, CDCl$_3$)
7.48
7.45
7.43
7.42
7.39
7.37
7.35
7.30
7.28
7.26 CDCl3
6.94
6.91
4.69
3.82
2.91
1.11
Ph
Me
N
Me
O
MeO
trans-**6b**
5.01
1.99
1.96
0.96
3.00
2.95
3.02
f1 (ppm)

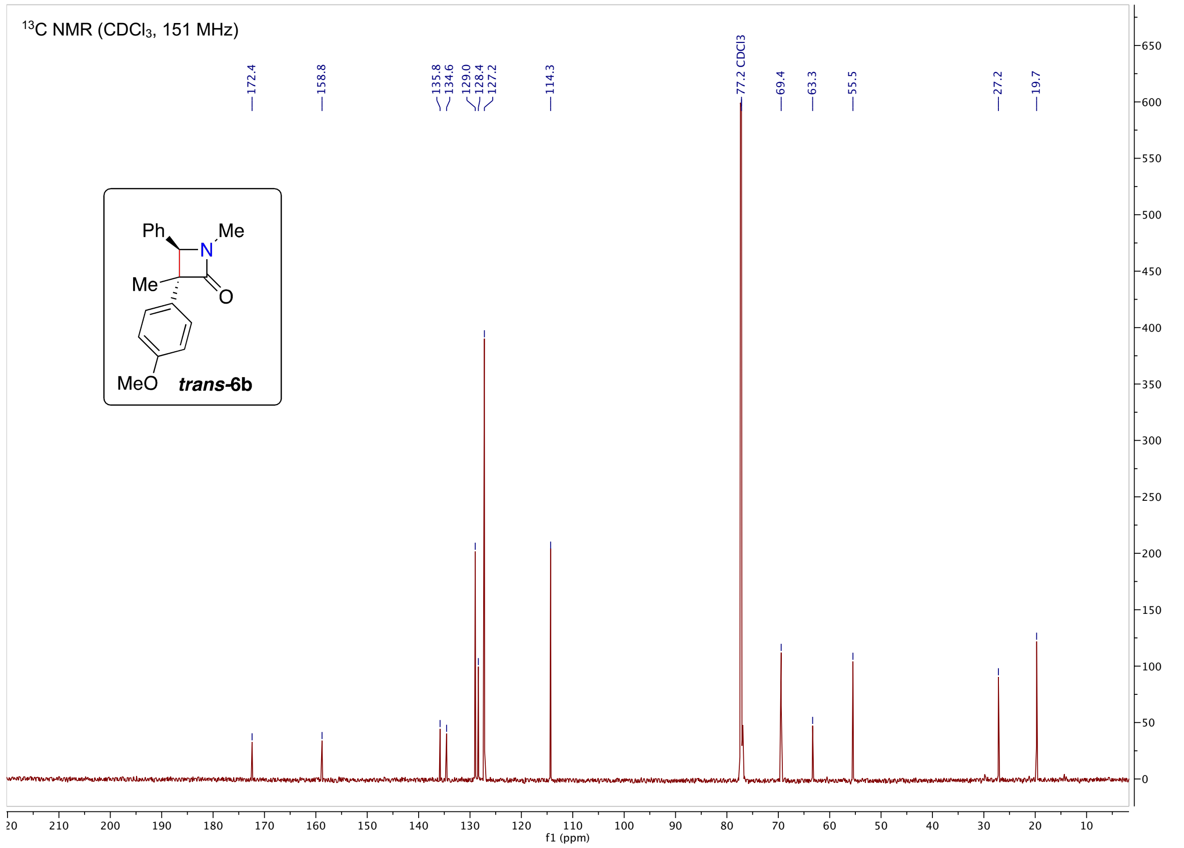

^{13}C NMR (CDCl$_3$, 151 MHz)
172.4
158.8
135.8
134.6
129.0
128.4
127.2
114.3
77.2 CDCl3
69.4
63.3
55.5
27.2
19.7
Ph
Me
N
Me
O
MeO
trans-**6b**
f1 (ppm)

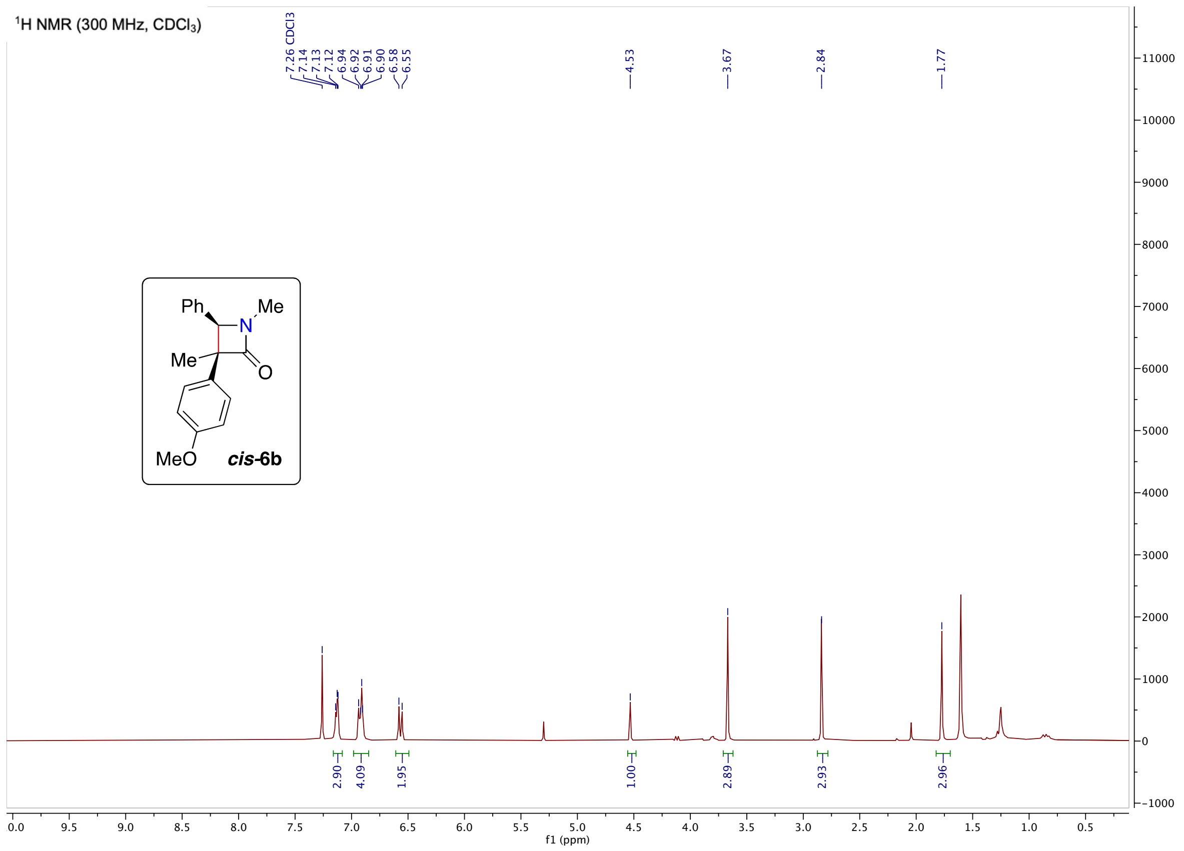

^{1}H NMR (300 MHz, CDCl$_3$)
7.26 CDCl3
7.14
7.13
7.12
6.94
6.92
6.91
6.90
6.58
6.55
4.53
3.67
2.84
1.77
2.90
4.09
1.95
1.00
2.89
2.93
2.96
Ph
N
Me
Me
O
MeO
cis-6b
f1 (ppm)

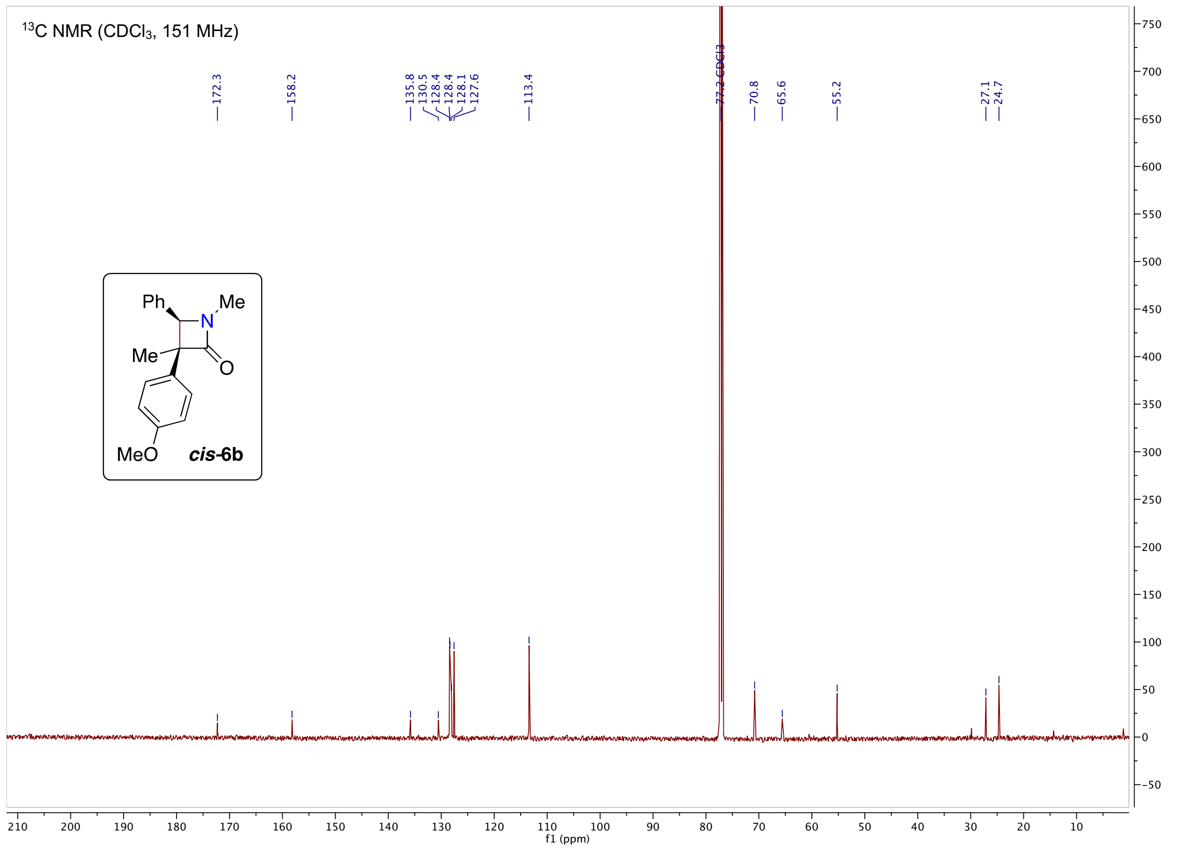

^{13}C NMR (CDCl$_3$, 151 MHz)
172.3
158.2
135.8
130.5
128.4
128.4
128.1
127.6
113.4
77.2 CDCl3
70.8
65.6
55.2
27.1
24.7
Ph
N
Me
Me
O
MeO
cis-6b
f1 (ppm)

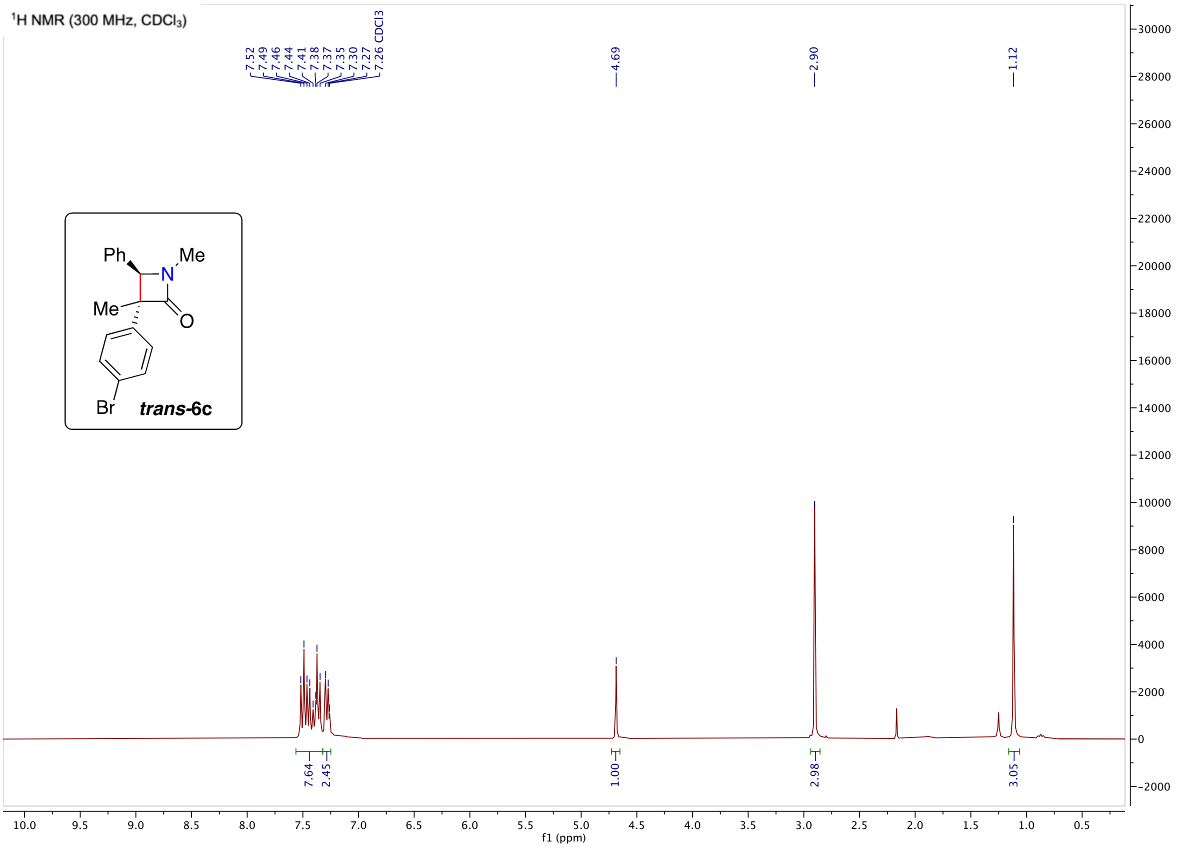

^{1}H NMR (300 MHz, CDCl$_3$)
7.52
7.49
7.46
7.44
7.41
7.38
7.37
7.35
7.30
7.27
7.26 CDCl3
4.69
2.90
1.12
Ph
N
Me
Me
O
Br
trans-6c
7.64
2.45
1.00
2.98
3.05
f1 (ppm)

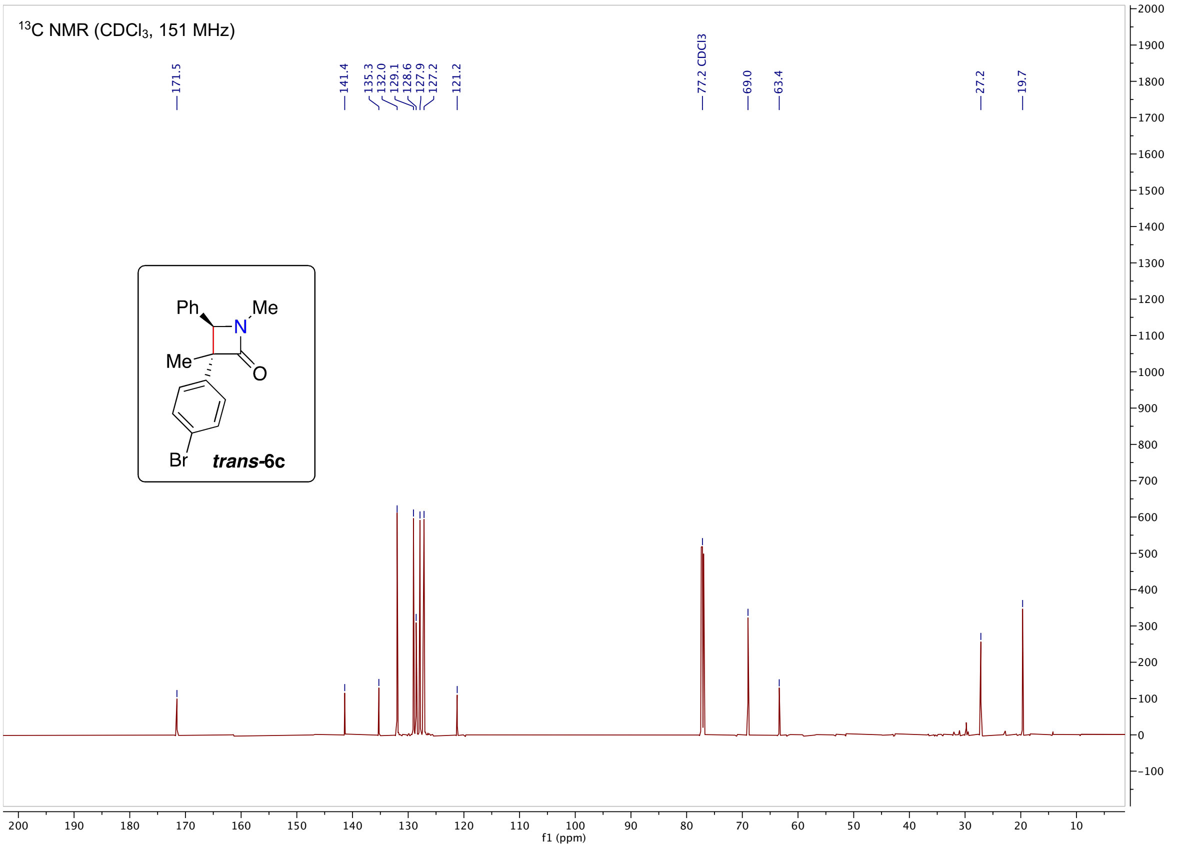

^{13}C NMR (CDCl$_3$, 151 MHz)
171.5
141.4
135.3
132.0
129.1
128.6
127.9
127.2
121.2
77.2 CDCl3
69.0
63.4
27.2
19.7
Ph
N
Me
Me
O
Br
trans-6c
f1 (ppm)

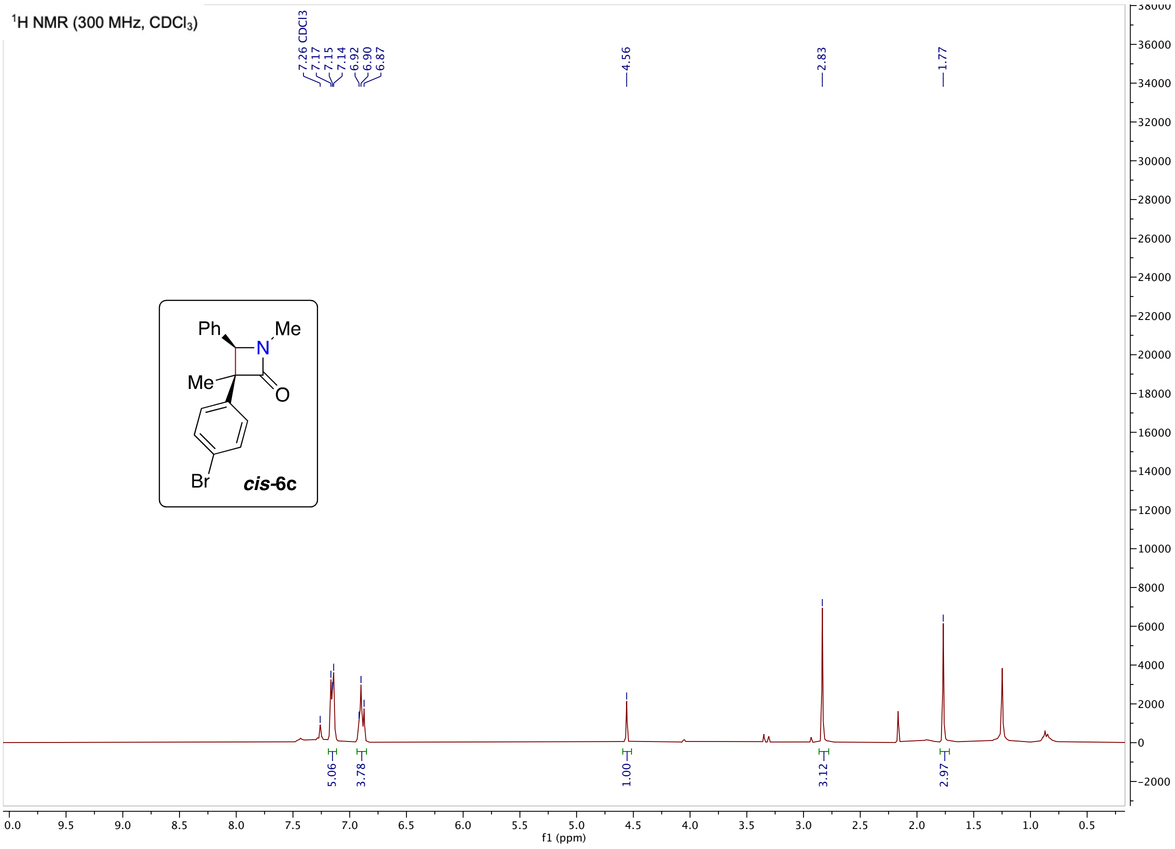

^{1}H NMR (300 MHz, CDCl$_3$)
7.26 CDCl3
7.17
7.15
7.14
6.92
6.90
6.87
4.56
2.83
1.77
Ph
N
Me
Me
O
Br
cis-6c
5.06
3.78
1.00
3.12
2.97
f1 (ppm)

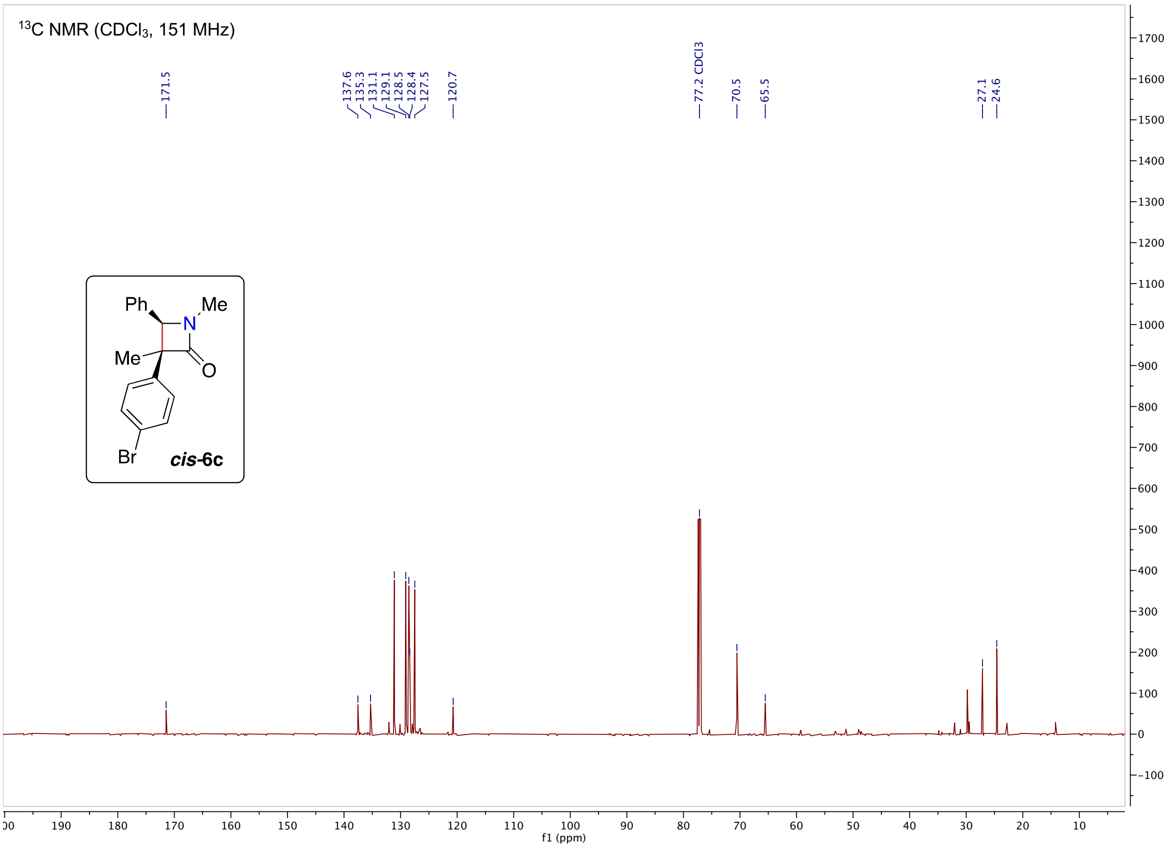

^{13}C NMR (CDCl$_3$, 151 MHz)
171.5
137.6
135.3
131.1
129.1
128.5
128.4
127.5
120.7
77.2 CDCl3
70.5
65.5
27.1
24.6
Ph
N
Me
Me
O
Br
cis-6c
f1 (ppm)

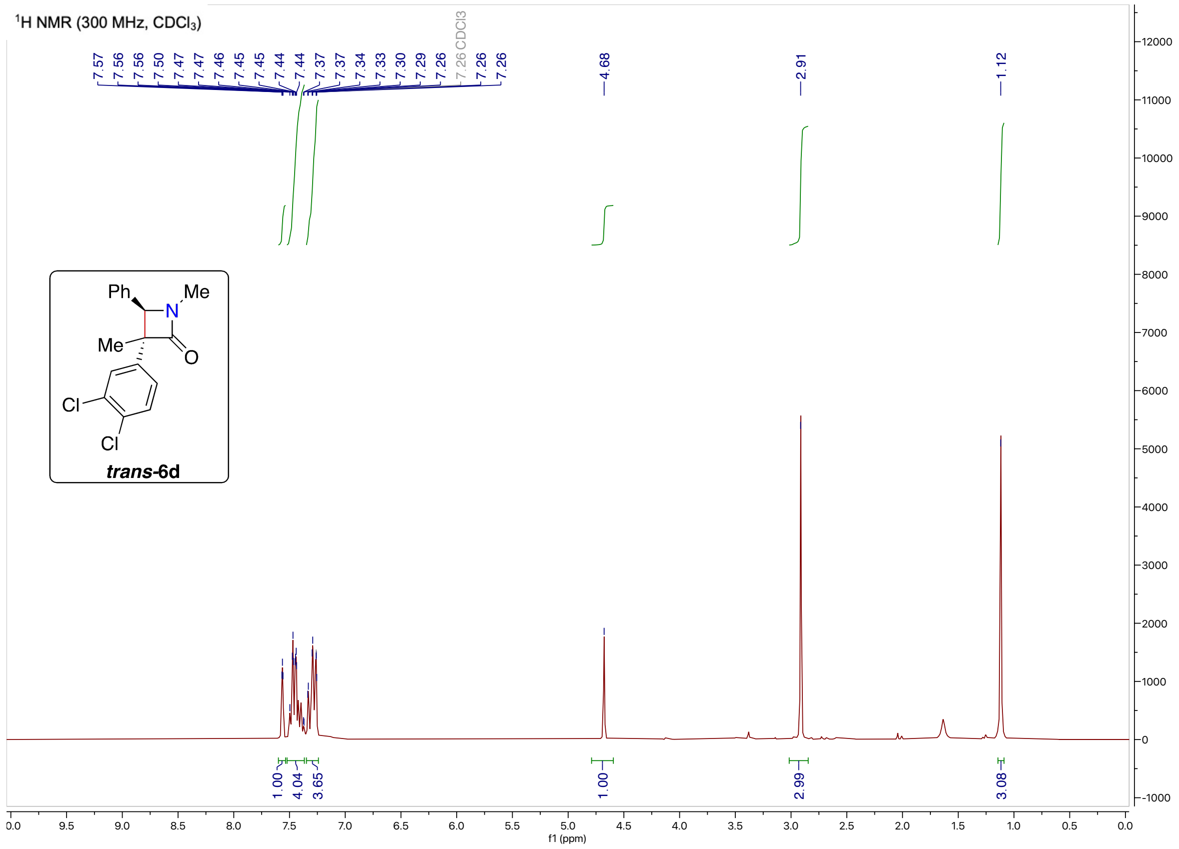

^{1}H NMR (300 MHz, CDCl$_3$)
7.57 7.56 7.56 7.50 7.47 7.47 7.46 7.45 7.45 7.44 7.44 7.37 7.37 7.34 7.33 7.30 7.29 7.26 7.26 CDCl3 7.26 7.26
4.68
2.91
1.12
Ph
N
Me
Me
O
Cl
Cl
trans-6d
1.00 4.04 3.65
1.00
2.99
3.08
f1 (ppm)

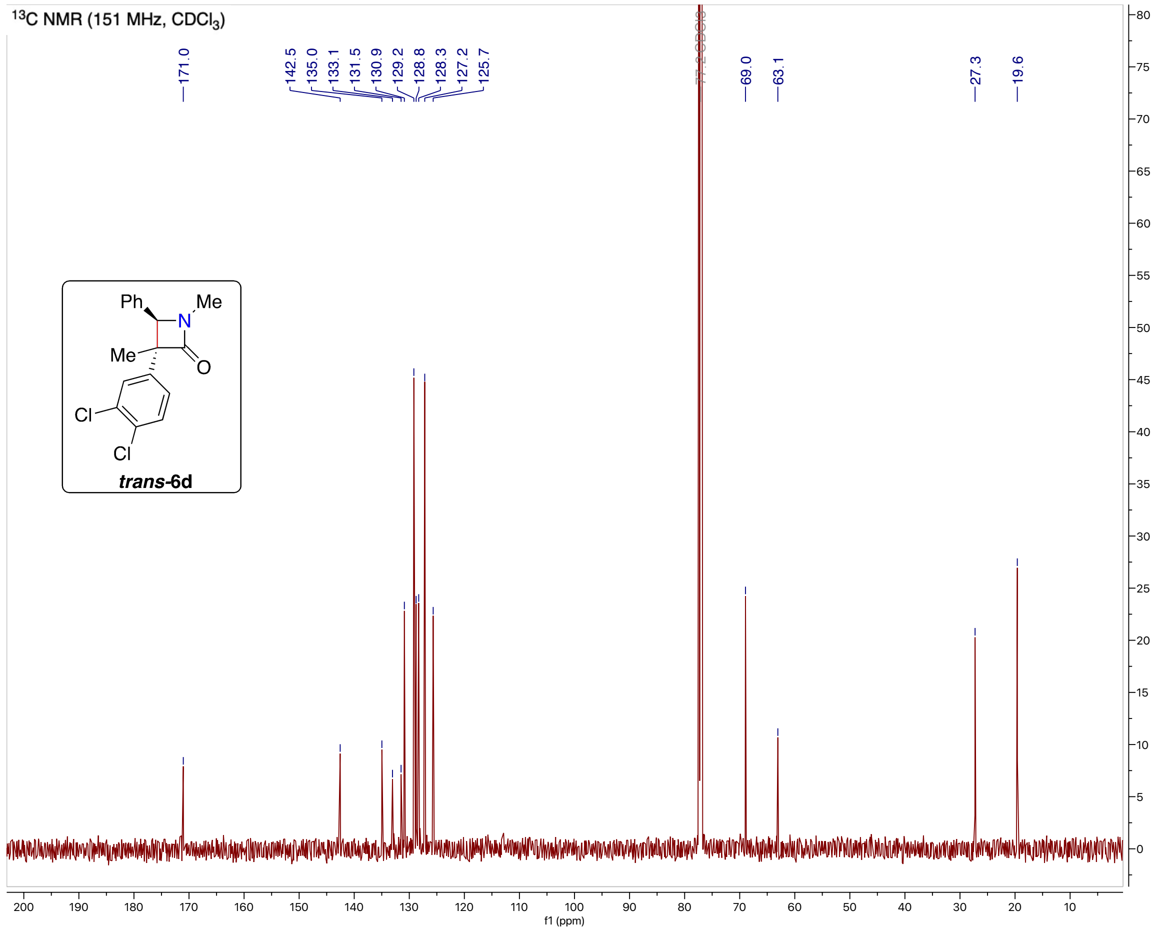

^{13}C NMR (151 MHz, CDCl$_3$)
171.0
142.5 135.0 133.1 131.5 130.9 129.2 128.8 128.3 127.2 125.7
69.0
63.1
27.3
19.6
Ph
N
Me
Me
O
Cl
Cl
trans-6d
f1 (ppm)

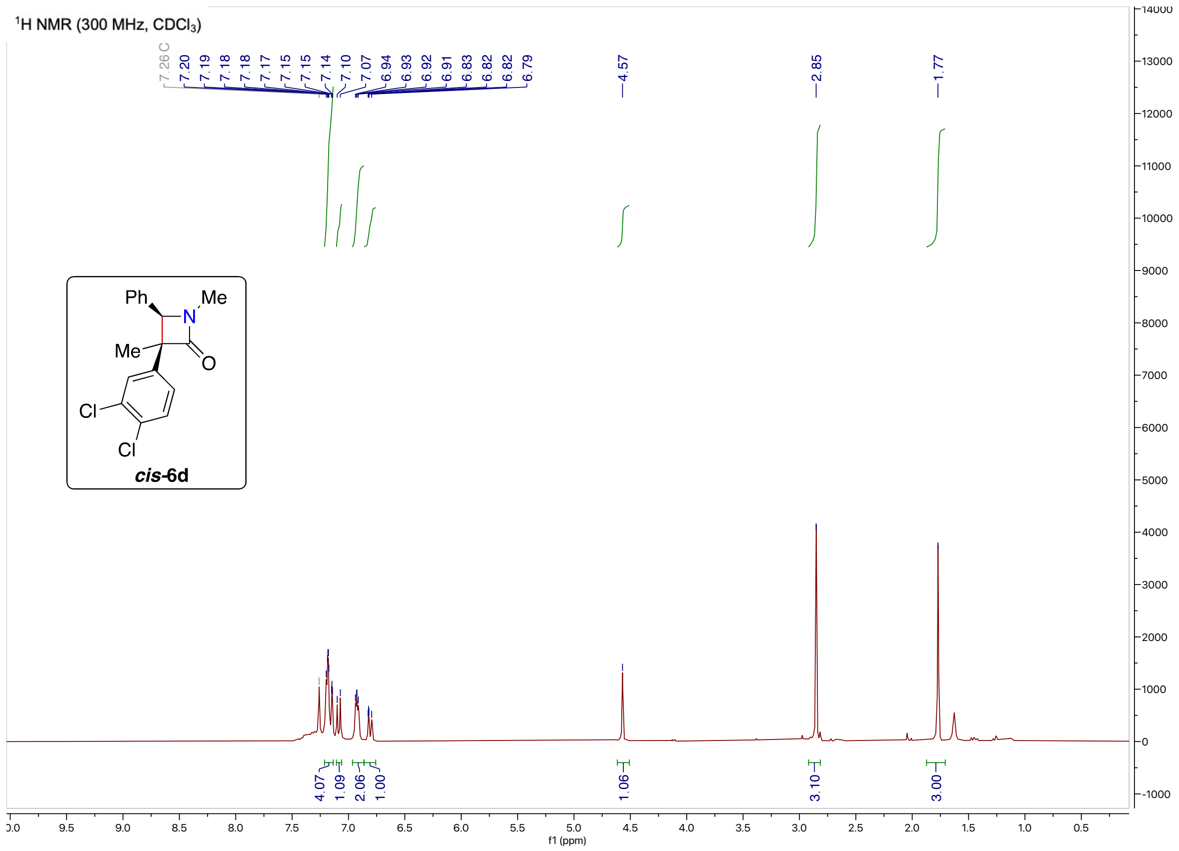

^{1}H NMR (300 MHz, CDCl$_3$)
7.26 C
7.20
7.19
7.18
7.18
7.17
7.15
7.15
7.14
7.10
7.07
6.94
6.93
6.92
6.91
6.83
6.82
6.82
6.79
4.57
2.85
1.77
Ph
Me
N
Me
O
Cl
Cl
cis-6d
4.07
1.09
2.06
1.00
1.06
3.10
3.00
f1 (ppm)

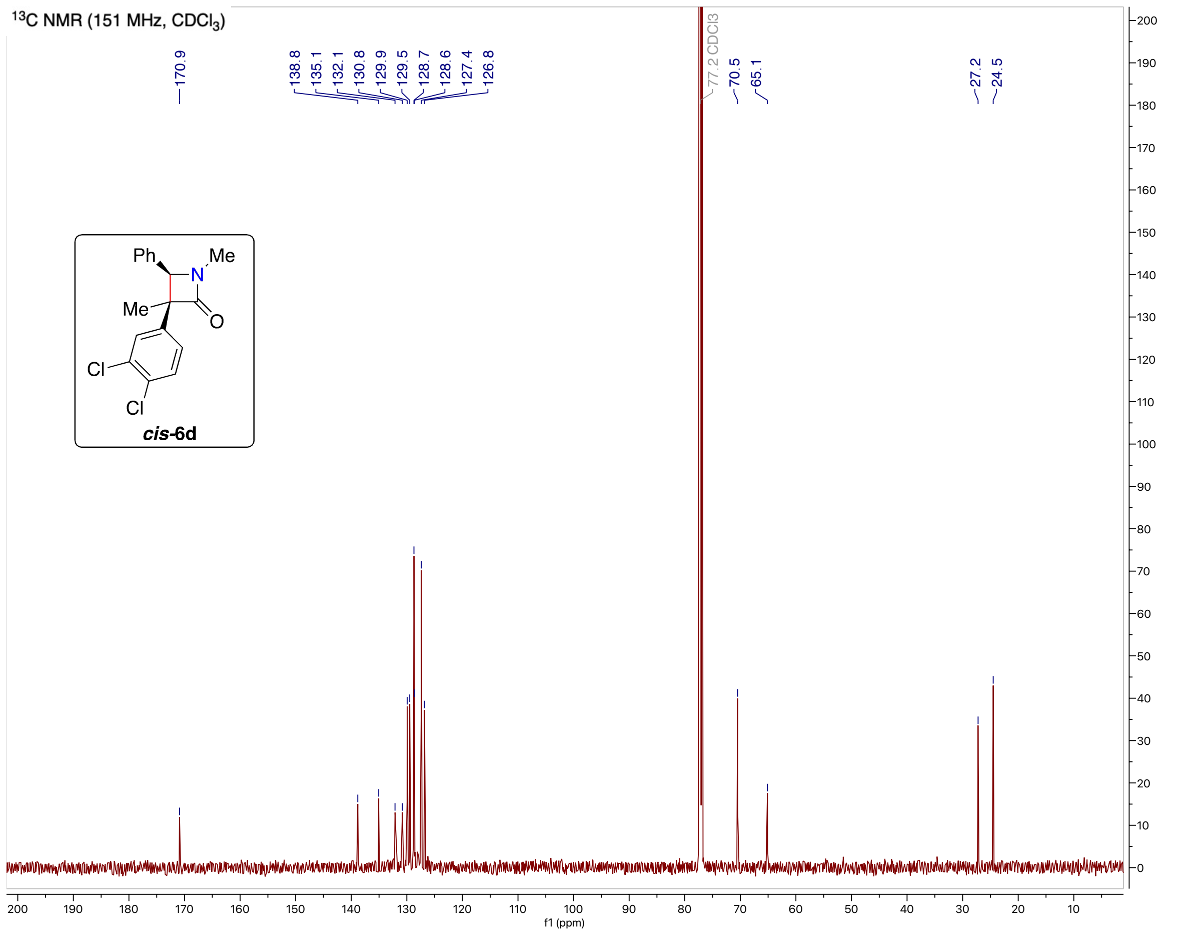

^{13}C NMR (151 MHz, CDCl$_3$)
170.9
138.8
135.1
132.1
130.8
129.9
129.5
128.7
128.6
127.4
126.8
77.2 CDCl3
70.5
65.1
27.2
24.5
Ph
Me
N
Me
O
Cl
Cl
cis-6d
f1 (ppm)

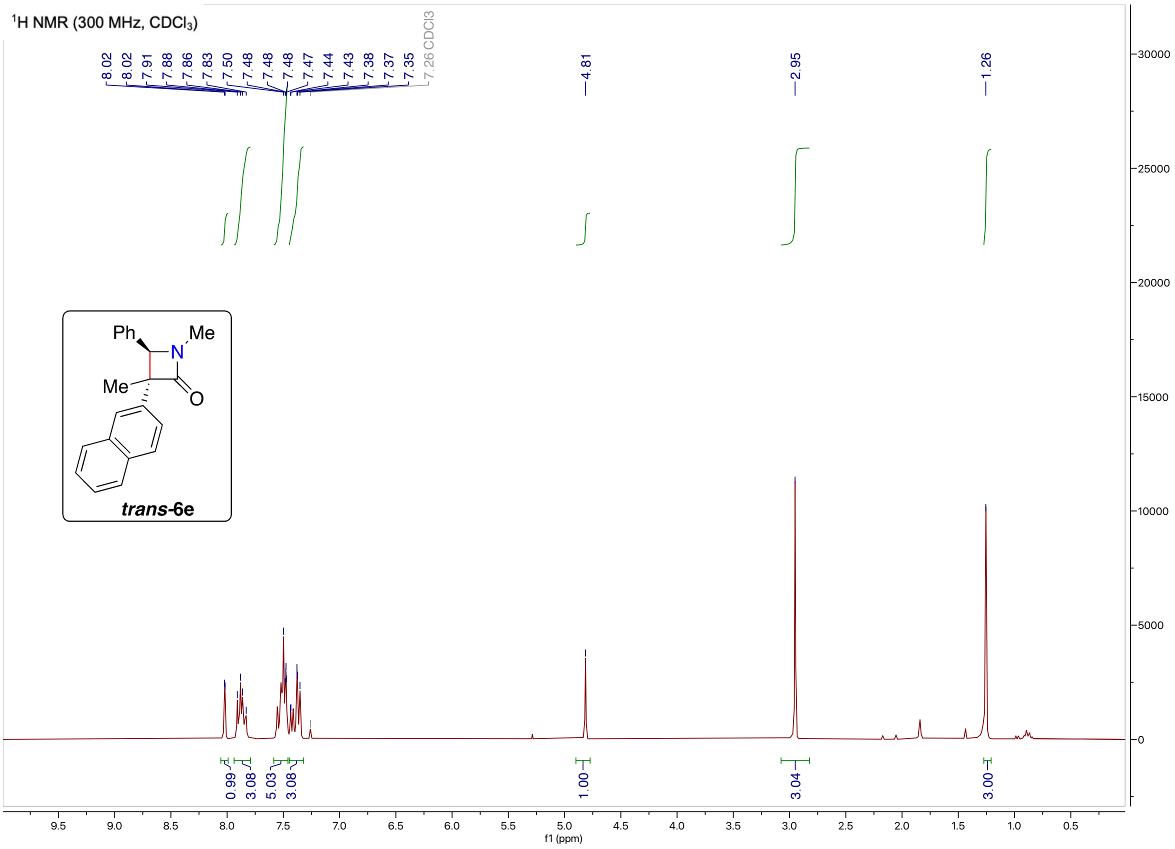
^{1}H NMR (300 MHz, CDCl$_3$)
8.02 8.02 7.91 7.88 7.86 7.83 7.50 7.48 7.48 7.48 7.47 7.44 7.43 7.38 7.37 7.35 7.26 CDCl3
4.81
2.95
1.26
Ph
N
Me
Me
O
trans-6e
0.99 3.08 5.03 3.08
1.00
3.04
3.00
f1 (ppm)

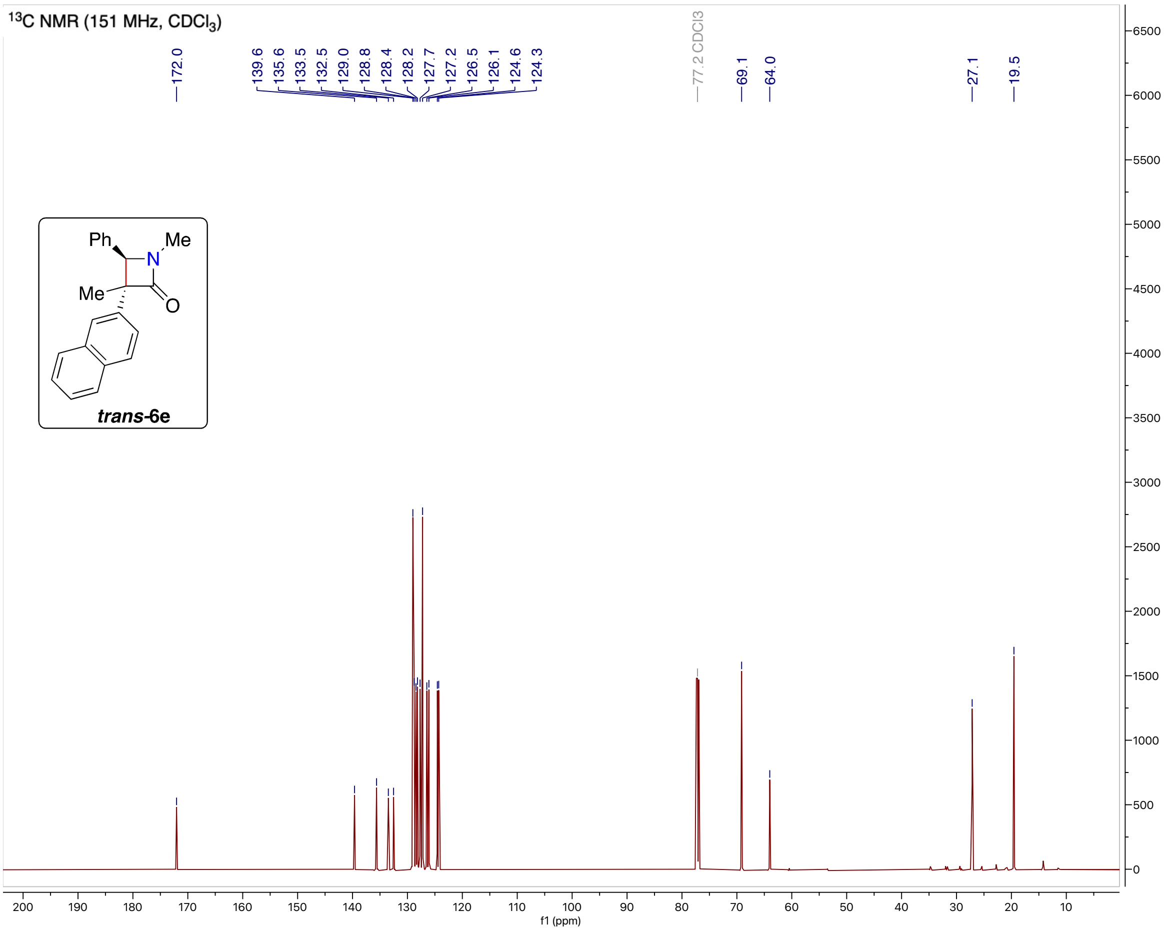
^{13}C NMR (151 MHz, CDCl$_3$)
172.0
139.6 135.6 133.5 132.5 129.0 128.8 128.4 128.2 127.7 127.2 126.5 126.1 124.6 124.3
77.2 CDCl3
69.1
64.0
27.1
19.5
Ph
N
Me
Me
O
trans-6e
f1 (ppm)

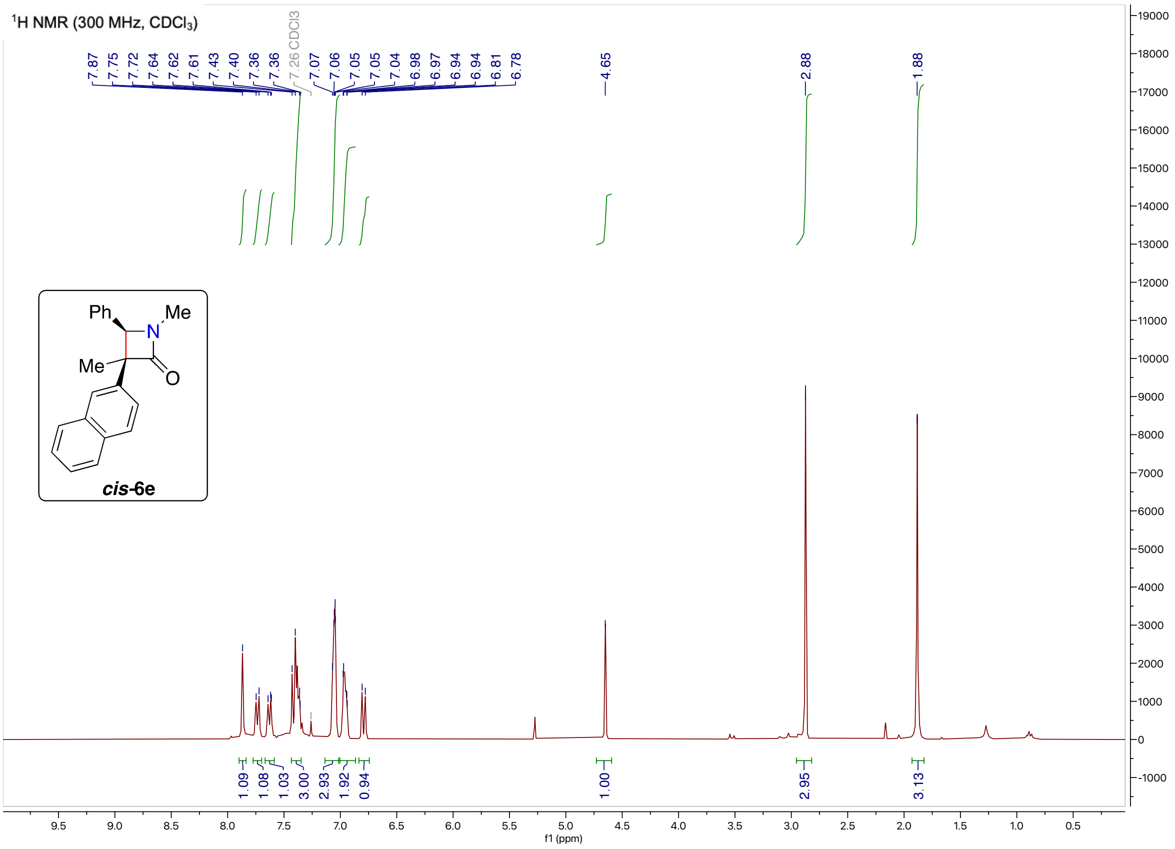

^{1}H NMR (300 MHz, CDCl3)
7.87 7.75 7.72 7.64 7.62 7.61 7.43 7.40 7.36 7.36 7.26 CDCl3 7.07 7.06 7.05 7.05 7.04 6.98 6.97 6.94 6.94 6.81 6.78
4.65
2.88
1.88
1.09 1.08 1.03 3.00 2.93 1.92 0.94 1.00 2.95 3.13
Ph
Me
N
Me
O
cis-6e
f1 (ppm)

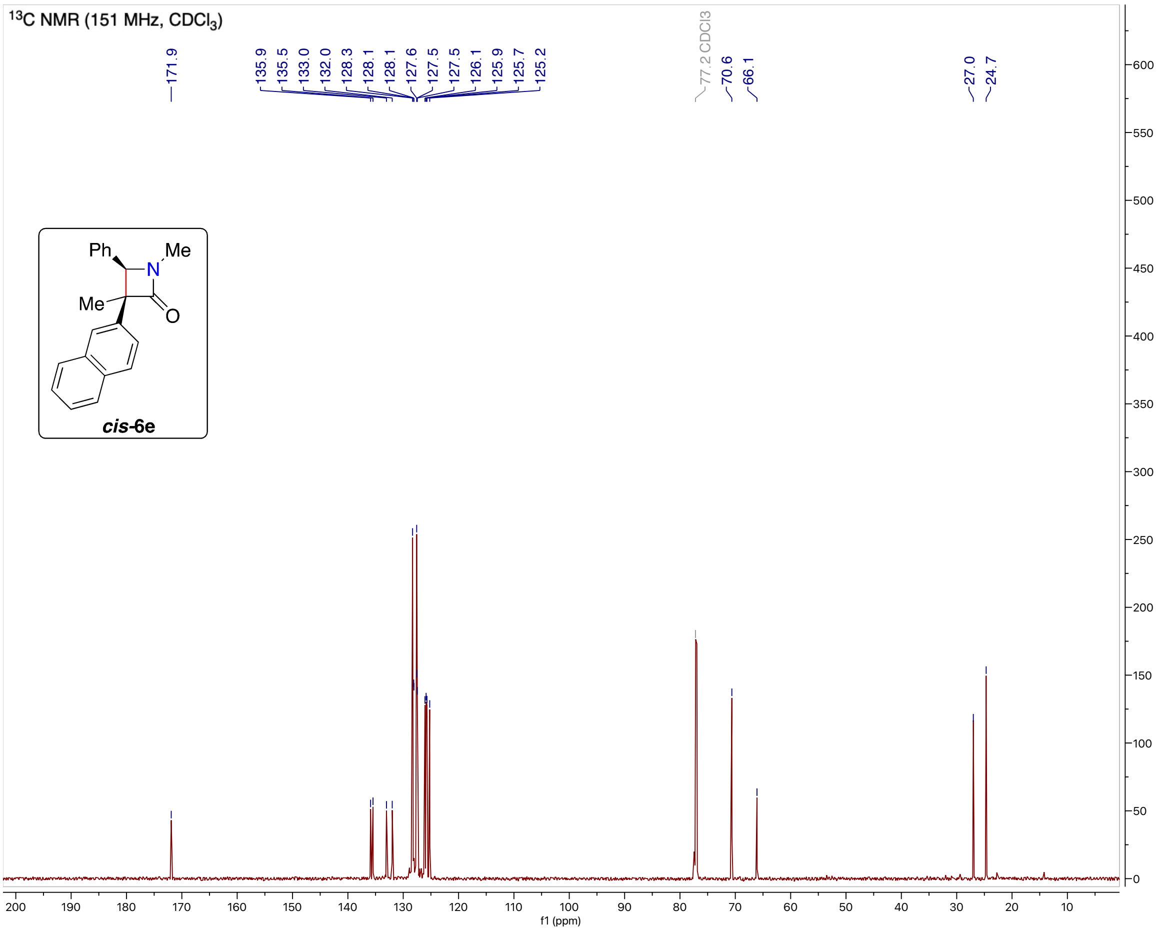

^{13}C NMR (151 MHz, CDCl3)
171.9
135.9 135.5 133.0 132.0 128.3 128.1 128.1 127.6 127.5 126.1 125.9 125.7 125.2
77.2 CDCl3 70.6 66.1
27.0 24.7
Ph
Me
N
Me
O
cis-6e
f1 (ppm)
